# Supplementary material for: Assessment of medical information on irritable bowel syndrome information in Wikipedia and Baidu Encyclopedia: comparative study
Source: PeerJ. 2024 May 24;12:e17264. doi: 10.7717/peerj.17264 (PMC11129691; doi:10.7717/peerj.17264)
Supplement: Data S1 [file peerj-12-17264-s001.zip › σÄƒσoïμò░μì«/Wikipedia/2-Functional constipation - Wikipedia.pdf]

WIKIPEDIA

Functional constipation

Functional constipation	
**Other names**	Chronic idiopathic constipation

Functional constipation, known as **chronic idiopathic constipation** (**CIC**), is constipation that does not have a physical (anatomical) or physiological (hormonal or other body chemistry) cause. It may have a neurological, psychological or psychosomatic cause. A person with functional constipation may be healthy, yet has difficulty defecating.

Contents

Symptoms and diagnosis
Treatment
Research
See also
References

Symptoms and diagnosis

Chronic idiopathic constipation is similar to constipation-predominant irritable bowel syndrome (IBS-C); however, people with CIC do not have other symptoms of IBS, such as abdominal pain.[1] Diagnosing CIC can be difficult as other syndromes must be ruled out as there is no physiological cause for CIC. Doctors will typically look for other symptoms, such as blood in stool, weight loss, low blood count, or other symptoms.

To be considered functional constipation, symptoms must be present at least a fourth of the time.[1] Possible causes are:

- Anismus
- Descending perineum syndrome
- Other inability or unwillingness to control the external anal sphincter, which normally is under voluntary control
- A poor diet
- An unwillingness to defecate
- Nervous reactions, including prolonged and/or chronic stress and anxiety, that close the internal anal sphincter, a muscle that is not under voluntary control
- Deeper psychosomatic disorders which sometimes affect digestion and the absorption of water in the colon

There is also possibility of presentation with other comorbid symptoms such as headache, especially in children.[2]

Treatment

Treatment options appear similar and include prucalopride, lubiprostone, linaclotide, tegaserod, velusetrag, elobixibat, bisacodyl, sodium picosulphate,[3] and most recently, plecanatide.

Research

A 2014 meta-analysis of three small trials evaluating probiotics showed a slight improvement in management of chronic idiopathic constipation, but well-designed studies are necessary to know the true efficacy of probiotics in treating this condition.[4]

Children with functional constipation often claim to lack the sensation of the urge to defecate, and may be conditioned to avoid doing so due to a previous painful experience.[5] One retrospective study showed that these children did indeed have the urge to defecate using colonic manometry, and suggested behavioral modification as a treatment for functional constipation.[6]

See also

- Functional symptom
- Sacral nerve stimulation

References

1. Americal College of Gastroenterology: Fuinctional Bowel Disorders (http://www.acg.gi.org/patients/gihealth/pdf/functional.pdf)
2. Inaloo S, Dehghani SM, Hashemi SM, Heydari M, Heydari ST (2014). "Comorbidity of headache and functional constipation in children: a cross-sectional survey". *Turk J Gastroenterol*. **25** (5): 508–11. doi:10.5152/tjg.2014.6183 (https://doi.org/10.5152%2Ftjg.2014.6183). PMID 25417610 (https://pubmed.ncbi.nlm.nih.gov/25417610).
3. Nelson, AD; Camilleri, M; Chirapongsathorn, S; Vijayvargiya, P; Valentin, N; Shin, A; Erwin, PJ; Wang, Z; Murad, MH (September 2017). "Comparison of efficacy of pharmacological treatments for chronic idiopathic constipation: a systematic review and network meta-analysis". *Gut*. **66** (9): 1611–1622. doi:10.1136/gutjnl-2016-311835 (https://doi.org/10.1136%2Fgutjnl-2016-311835). hdl:1805/12164 (https://hdl.handle.net/1805%2F12164). PMID 27287486 (https://pubmed.ncbi.nlm.nih.gov/27287486).
4. Ford, Alexander C; Quigley, Eamonn M M; Lacy, Brian E; Lembo, Anthony J; Saito, Yuri A; Schiller, Lawrence R; Soffer, Edy E; Spiegel, Brennan M R; Moayyedi, Paul (2014). "Efficacy of Prebiotics, Probiotics, and Synbiotics in Irritable Bowel Syndrome and Chronic Idiopathic Constipation: Systematic Review and Meta-analysis". *The American Journal of Gastroenterology*. **109** (10): 1547–1561. doi:10.1038/ajg.2014.202 (https://doi.org/10.1038%2Fajg.2014.202). ISSN 0002-9270 (https://www.worldcat.org/issn/0002-9270). PMID 25070051 (https://pubmed.ncbi.nlm.nih.gov/25070051).
5. Fleisher, DR (November 1976). "Diagnosis and treatment of disorders of defecation in children". *Pediatric Annals*. **5** (11): 71–101. PMID 980548 (https://pubmed.ncbi.nlm.nih.gov/980548).
6. Firestone Baum, C; John A; Srinivasan K; Harrison P; Kolomensky A; Monagas J; Cocjin J; Hyman PE (January 2013). "Colon manometry proves that perception of the urge to defecate is present in children with functional constipation who deny sensation".

Journal of Pediatric Gastroenterology and Nutrition. **56** (1): 19–22. doi:10.1097/MPG.0b013e31826f2740 (https://doi.org/10.1097%2FMPG.0b013e31826f2740). PMID 22922371 (https://pubmed.ncbi.nlm.nih.gov/22922371).

Retrieved from "https://en.wikipedia.org/w/index.php?title=Functional_constipation&oldid=987539533"

This page was last edited on 7 November 2020, at 18:22 (UTC).

Text is available under the Creative Commons Attribution-ShareAlike License 3.0; additional terms may apply. By using this site, you agree to the Terms of Use and Privacy Policy. Wikipedia® is a registered trademark of the Wikimedia Foundation, Inc., a non-profit organization.
