# Supplementary material for: Assessment of medical information on irritable bowel syndrome information in Wikipedia and Baidu Encyclopedia: comparative study
Source: PeerJ. 2024 May 24;12:e17264. doi: 10.7717/peerj.17264 (PMC11129691; doi:10.7717/peerj.17264)
Supplement: Data S1 [file peerj-12-17264-s001.zip › σÄƒσoïμò░μì«/Wikipedia/Neurogenic bowel dysfunction - Wikipedia.pdf]

WIKIPEDIA

Neurogenic bowel dysfunction

Neurogenic bowel dysfunction (NBD) is the inability to control defecation due to a deterioration of or injury to the nervous system, resulting in faecal incontinence or constipation.[1] It is common in people with spinal cord injury (SCI), multiple sclerosis (MS) or spina bifida.[2]

Neurogenic bowel dysfunction

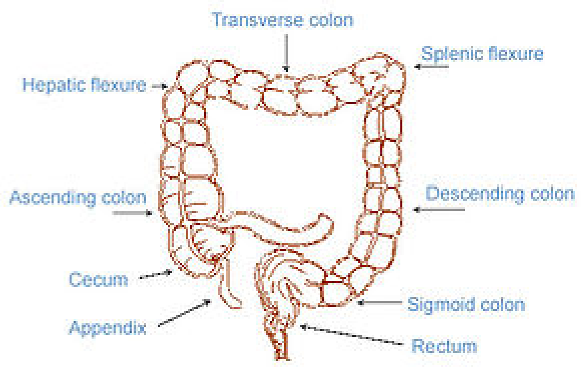

The image shows the 4 parts of the colon (ascending, transverse, descending and sigmoid) and the rectum. Faeces are transported along and stored in the rectum before excretion

**Specialty**	Gastroenterology

The gastrointestinal tract has a complex control that relies on coordinated interaction between muscular contractions and neuronal impulses.[3] Faecal incontinence or constipation occurs when there is a problem with the normal bowel functioning; this could be for a variety of reasons. The normal defecation pathway involves contractions of the colon in order to help mix the contents, absorb water and propel the contents along the intestine. This results in the faeces moving from the colon to the rectum.[4] The presence of stool in the rectum causes a reflexive relaxation of the internal anal sphincter, so the contents of the rectum can move in to the anal canal. This causes the conscious feeling of the need to defecate. At a suitable time our brain can send signals causing the external anal sphincter and puborectalis muscles to relax as these are under voluntary control and this allows defecation to take place.[4][5]

Spinal cord injury and other neurological problems mostly affect the lower GI tract leading to symptoms of incontinence or constipation. However, the upper GI tract may also be affected and patients with neurogenic bowel often present with multiple symptoms.[6][7] Research shows there is a high prevalence of upper abdominal complaints, for example a study showed that approximately 22% of SCI patients reported feeling bloated [6][8] and about 31% of SCI patients' experienced abdominal distension.[6][9]

Contents

Signs and symptoms
Causes
- Spinal cord injury
- Spina bifida
- Multiple sclerosis
- Brain lesion
- Parkinson's disease
- Diabetes mellitus

Mechanism
Diagnosis

Management
References

Signs and symptoms

Neurogenic bowel dysfunction can have an impact on a person's life as it often leads to difficulties with self-esteem, personal relationships, social life and can also reduce a person's independence.[5] There is also evidence from studies showing that faecal incontinence can increase the risk of depression and anxiety.[10]

Causes

Different neurological disorders affect the gastrointestinal tract in different ways:

Spinal cord injury

Bowel dysfunction caused by a spinal cord injury will vary greatly depending on the severity and level of the spinal cord lesion. In complete spinal cord injury both sensory and motor functions are completely lost below the level of the lesion so there is a loss of voluntary control and loss of sensation of the need to defecate.[11] An incomplete spinal cord injury is one where there may still be some sensation or motor function below the level of the lesion.[11]

Colorectal dysfunction due to spinal cord injury can be classified in to two types: an upper motor neuron lesion or lower motor neuron lesion. Problems with the upper motor neuron in a neurogenic bowel results in a hypertonic and spastic bowel because the defecation reflex centre, which causes the involuntary contraction of muscles of the rectum and anus, remains intact.[5] However, the nerve damage results in disruption to the nerve signals and therefore there is an inability to relax the anal sphincters and defecate, often leading to constipation.[5] An upper motor neuron lesion is one that is above the conus medullaris of the spinal cord and therefore above the vertebral level T12.[12] On the other hand, a lower motor neuron lesion can cause areflexia and a flaccid external anal sphincter so most commonly leading to incontinence. Lower motor neuron lesions are damage to nerves that are at the level of or below the conus medullaris and below vertebral level T12. However, both upper and lower motor neuron disorders can lead to constipation and/ or incontinence.[13][12]

Spina bifida

Patients with Spina Bifida have a neural tube that has failed to completely form. This is most commonly in the lower back area in the region of the conus medullaris or cauda equina. It, therefore, affects the bowel similarly to a spinal cord injury affecting the lower motor neuron resulting in a flaccid unreactive rectal wall and means the anal sphincter doesn't contract and close therefore leading to stool leakage.[11] Most patients with spina bifida also have hydrocephalus this can result in intellectual deficits so can contribute to faecal incontinence.[5]

Multiple sclerosis

There are a variety of symptoms associated with this condition that are all caused by a loss of myelin, the insulating layer surrounding the neurones. This means the nerve signals are interrupted and slower which will then cause muscle contractions to be fewer and irregular

resulting in an increased colon transit time.[11] The faeces being in the colon for a longer time will mean that more water is absorbed leading to harder stools and therefore increasing the symptoms of constipation. This neurological problem can also lead to reduced sensation of rectal filling and weakness of the anal sphincter because of weak muscular contraction so can cause stool leakage.[11] In patients with multiple sclerosis constipation and faecal incontinence often coexist and they can be acute, chronic or intermittent due to the fluctuating pattern of MS.[5]

Brain lesion

Damage to the defecation centre within the medulla oblongata of the brain can lead to bowel dysfunction. A stroke or acquired brain injury may lead to damage to this centre in the brain. Damage to the defecation centre can lead to a loss of coordination between rectal and anal contractions and also a loss of awareness of the need to defecate.[11]

Parkinson's disease

This condition differs as it affects both the extrinsic and enteric nervous systems due to the decreased dopamine levels in both. This results in less smooth muscle contraction of the colon, increasing the colon transit time.[11] The reduced dopamine levels also causes dystonia of the striated muscles of the pelvic floor and external anal sphincter. This explains how Parkinson's disease can lead to constipation.[13]

Diabetes mellitus

Twenty percent of people with diabetes mellitus experience faecal incontinence due to irreversible autonomic neuropathy. This is due to the high blood glucose levels over time damaging the nerves, which can lead to impaired rectal sensation.[11]

Mechanism

There are different types of neurons involved in innervating the lower GI tract these include: the enteric nervous system; located within the wall of the gut, and the extrinsic nervous system; comprising sympathetic and parasympathetic innervation.[3] The enteric nervous system directly controls the gut motility, whereas, the extrinsic nerve pathways influence gut contractility indirectly through modifying this enteric innervation.[3] In almost all cases of neurogenic bowel dysfunction it is the extrinsic nervous supply affected and the enteric nervous supply remains intact. The only exception being Parkinson's disease, as this can affect both the enteric and extrinsic innervation.[11]

Defecation involves conscious and subconscious processes, when the extrinsic nervous system is damaged either of these can be affected. Conscious processes are controlled by the somatic nervous system, these are voluntary movements for example the contraction of the striated muscle of the external anal sphincter is instructed to do so by the brain, which sends signals along the nerves innervating this muscle.[14][15] Subconscious processes are controlled by the autonomic nervous system; these are involuntary movements such as contraction of the smooth muscle of the internal anal sphincter or the colon. The autonomic nervous system also provides sensory information; this could be about the level of distension within the colon or rectum.[14][15]

Diagnosis

In order to correctly manage neurogenic bowel dysfunction it is important to accurately diagnose it. This can be done by a variety of methods, the most commonly used would be taking a clinical history and carrying out physical examinations which may include: abdominal, neurological and rectal examinations. [16] Patients may use the Bristol Stool Chart to help them describe and characterise the morphological features of their stool, this is useful as it gives an indication of the transit time.[17] An objective method used to evaluate the motility of the colon and help with diagnosis is the colon transit time.[18] Another helpful test to diagnose this condition may be an abdominal X- ray as this can show the distribution of the faeces and show any abnormalities with the colon for example a megacolon.[13] Methods used for diagnosis may vary depending on if the patient is incontinent or constipated

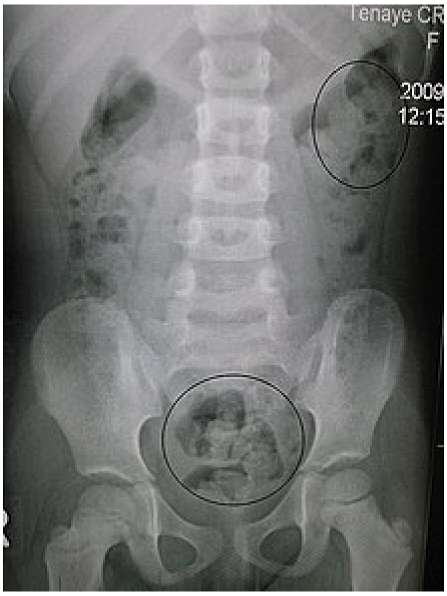

This image shows constipation in a young child as seen on X– ray.

Management

Management and treatment for neurogenic bowel dysfunction depends on symptoms and biomedical diagnosis for cause of the condition.[13] General practitioners will often refer patients to gastroenterologist to effectively manage the neurogenic bowel dysfunction. Research has been conducted on a variety of therapy and treatments for neurogenic bowel dysfunction including: diet modification, laxatives, magnetic and electrical stimulation, manual evacuation of feces and abdominal massage, enemas, and pulsed irrigation evacuation (PIE).[14][19][20] Efficacy studies for pulsed irrigation evacuation with PIEMED demonstrated favorable results, removing stool of 98% of patients who used it for ineffective bowel routine, symptomatic impaction, or asymptomatic impaction.[19] In the most severe of cases of neurogenic bowel dysfunction induced fecal impaction, surgical interventions like colostomy are used to disrupt the dense mass of stool.[21][22]

References

1. "Sample records for neurogenic bowel dysfunction" (https://www.science.gov/topicpages/n/neurogenic%2Bbowel%2Bdysfunction). *science.gov*. Science.gov. Archived (https://web.archive.org/web/20180929114707/https://www.science.gov/topicpages/n/neurogenic%2Bbowel%2Bdysfunction) from the original on 2018-09-29. Retrieved 2018-09-28.
2. Emmanuel, A (2010-02-09). "Review of the efficacy and safety of transanal irrigation for neurogenic bowel dysfunction" (https://doi.org/10.1038%2Fsc.2010.5). *Spinal Cord*. **48** (9): 664–673. doi:10.1038/sc.2010.5 (https://doi.org/10.1038%2Fsc.2010.5). PMID 20142830 (https://pubmed.ncbi.nlm.nih.gov/20142830).
3. Brookes, SJ; Dinning, PG; Gladman, MA (December 2009). "Neuroanatomy and physiology of colorectal function and defaecation: from basic science to human clinical studies". *Neurogastroenterol Motil*. **21**: 9–19. doi:10.1111/j.1365-2982.2009.01400.x (https://doi.org/10.1111%2Fj.1365-2982.2009.01400.x). PMID 19824934 (https://pubmed.ncbi.nlm.nih.gov/19824934). S2CID 33885542 (https://api.semanticscholar.org/CorpusID:33885542).
4. Palit, Somnath; Lunniss, Peter J.; Scott, S. Mark (2012-02-26). "The Physiology of Human Defecation". *Dig Dis Sci*. **57** (6): 1445–1464. doi:10.1007/s10620-012-2071-1 (https://doi.org/10.1007%2Fs10620-012-2071-1). PMID 22367113 (https://pubmed.ncbi.nlm.nih.gov/22367113). S2CID 25173457 (https://api.semanticscholar.org/CorpusID:25173457).

5. Krogh, K; Christensen, P; Laurberg, S (June 2001). "Colorectal symptoms in patients with neurological diseases". *Acta Neurol Scand*. **103** (6): 335–343. doi:10.1034/j.1600-0404.2001.103006335.x (https://doi.org/10.1034%2Fj.1600-0404.2001.103006335.x). PMID 11421845 (https://pubmed.ncbi.nlm.nih.gov/11421845). S2CID 28090580 (https://api.semanticscholar.org/CorpusID:28090580).
6. Qi, Zhengyan; Middleton, James W; Malcolm, Allison (2018-08-29). "Bowel Dysfunction in Spinal Cord Injury". *Curr Gastroenterol Rep*. **20** (10): 47. doi:10.1007/s11894-018-0655-4 (https://doi.org/10.1007%2Fs11894-018-0655-4). PMID 30159690 (https://pubmed.ncbi.nlm.nih.gov/30159690). S2CID 52129284 (https://api.semanticscholar.org/CorpusID:52129284).
7. Nielsen, S D; Faaborg, P M; Christensen, P; Krogh, K; Finnerup, N B (2016-08-09). "Chronic abdominal pain in long-term spinal cord injury: a follow-up study" (https://doi.org/10.1038%2Fsc.2016.124). *Spinal Cord*. **55** (3): 290–293. doi:10.1038/sc.2016.124 (https://doi.org/10.1038%2Fsc.2016.124). PMID 27502843 (https://pubmed.ncbi.nlm.nih.gov/27502843).
8. Ng, Clinton; Prott, Gillian; Rutkowski, Susan; Li, Yueming; Hansen, Ross; Kellow, John; Malcolm, Allison (2005). "Gastrointestinal Symptoms in Spinal Cord Injury: Relationships With Level of Injury and Psychologic Factors". *Dis Colon Rectum*. **48** (8): 1562–1568. doi:10.1007/s10350-005-0061-5 (https://doi.org/10.1007%2Fs10350-005-0061-5). PMID 15981066 (https://pubmed.ncbi.nlm.nih.gov/15981066). S2CID 22026816 (https://api.semanticscholar.org/CorpusID:22026816).
9. Coggrave, M; Norton, C; Wilson-Barnett, J (2008-11-18). "Management of neurogenic bowel dysfunction in the community after spinal cord injury: a postal survey in the United Kingdom" (https://doi.org/10.1038%2Fsc.2008.137). *Spinal Cord*. **47** (4): 323–333. doi:10.1038/sc.2008.137 (https://doi.org/10.1038%2Fsc.2008.137). PMID 19015665 (https://pubmed.ncbi.nlm.nih.gov/19015665).
10. Branagan, G; Tromans, A; Finnis, D (2003). "Effect of stoma formation on bowel care and quality of life in patients with spinal cord injury" (https://doi.org/10.1038%2Fsj.sc.3101529). *Spinal Cord*. **41** (12): 680–683. doi:10.1038/sj.sc.3101529 (https://doi.org/10.1038%2Fsj.sc.3101529). PMID 14639447 (https://pubmed.ncbi.nlm.nih.gov/14639447).
11. Pellatt, Glynis Collis (2008). "Neurogenic continence. Part 1: pathophysiology and quality of life". *Br J Nurs*. **17** (13): 836–841. doi:10.12968/bjon.2008.17.13.30534 (https://doi.org/10.12968%2Fbjon.2008.17.13.30534). PMID 18856146 (https://pubmed.ncbi.nlm.nih.gov/18856146).
12. Cotterill, Nikki; Madersbacher, Helmut; Wyndaele, Jean J.; Apostolidis, Apostolos; Drake, Marcus J.; Gajewski, Jerzy; Heesakkers, John; Panicker, Jalesh; Radziszewski, Piotr (2017-06-22). "Neurogenic bowel dysfunction: Clinical management recommendations of the Neurologic Incontinence Committee of the Fifth International Consultation on Incontinence 2013" (http://discovery.ucl.ac.uk/1562627/). *Neurourol Urodyn*. **37** (1): 46–53. doi:10.1002/nau.23289 (https://doi.org/10.1002%2Fnau.23289). PMID 28640977 (https://pubmed.ncbi.nlm.nih.gov/28640977). S2CID 206304249 (https://api.semanticscholar.org/CorpusID:206304249).
13. den Braber-Ymker, Marjanne; Lammens, Martin; van Putten, Michel J.A.M.; Nagtegaal, Iris D. (2017-01-06). "The enteric nervous system and the musculature of the colon are altered in patients with spina bifida and spinal cord injury" (https://www.ncbi.nlm.nih.gov/pmc/articles/PMC5306076). *Virchows Archiv*. **470** (2): 175–184. doi:10.1007/s00428-016-2060-4 (https://doi.org/10.1007%2Fs00428-016-2060-4). PMC 5306076 (https://www.ncbi.nlm.nih.gov/pmc/articles/PMC5306076). PMID 28062917 (https://pubmed.ncbi.nlm.nih.gov/28062917).
14. Preziosi, Giuseppe; Emmanuel, Anton (2009). "Neurogenic bowel dysfunction: pathophysiology, clinical manifestations and treatment". *Expert Rev Gastroenterol Hepatol*. **3** (4): 417–423. doi:10.1586/egh.09.31 (https://doi.org/10.1586%2Fegh.09.31). PMID 19673628 (https://pubmed.ncbi.nlm.nih.gov/19673628). S2CID 37109139 (https://api.semanticscholar.org/CorpusID:37109139).

15. Awad, Richard A (2011). "Neurogenic bowel dysfunction in patients with spinal cord injury, myelomeningocele, multiple sclerosis and Parkinson's disease" (https://www.ncbi.nlm.nih.gov/pmc/articles/PMC3235587). *World Journal of Gastroenterology*. **17** (46): 5035–48. doi:10.3748/wjg.v17.i46.5035 (https://doi.org/10.3748%2Fwjg.v17.i46.5035). PMC 3235587 (https://www.ncbi.nlm.nih.gov/pmc/articles/PMC3235587). PMID 22171138 (https://pubmed.ncbi.nlm.nih.gov/22171138).
16. Krogh, Klaus; Christensen, Peter (2009). "Neurogenic colorectal and pelvic floor dysfunction". *Best Pract Res Clin Gastroenterol*. **23** (4): 531–543. doi:10.1016/j.bpg.2009.04.012 (https://doi.org/10.1016%2Fj.bpg.2009.04.012). PMID 19647688 (https://pubmed.ncbi.nlm.nih.gov/19647688).
17. Amarenco, G. (2014). "Bristol Stool Chart : étude prospective et monocentrique de " l'introspection fécale " chez des sujets volontaires". *Progrès en Urol*. **24** (11): 708–713. doi:10.1016/j.purol.2014.06.008 (https://doi.org/10.1016%2Fj.purol.2014.06.008). PMID 25214452 (https://pubmed.ncbi.nlm.nih.gov/25214452).
18. Faaborg, P M; Christensen, P; Rosenkilde, M; Laurberg, S; Krogh, K (2010-11-23). "Do gastrointestinal transit times and colonic dimensions change with time since spinal cord injury?" (https://doi.org/10.1038%2Fsc.2010.162). *Spinal Cord*. **49** (4): 549–553. doi:10.1038/sc.2010.162 (https://doi.org/10.1038%2Fsc.2010.162). PMID 21102573 (https://pubmed.ncbi.nlm.nih.gov/21102573).
19. Gramlich, T.; Puet, T. (August 1998). "Long-term safety of pulsed irrigation evacuation (PIE) use with chronic bowel conditions" (https://pubmed.ncbi.nlm.nih.gov/9724176). *Digestive Diseases and Sciences*. **43** (8): 1831–1834. doi:10.1023/a:1018808408880 (https://doi.org/10.1023%2Fa%3A1018808408880). ISSN 0163-2116 (https://www.worldcat.org/issn/0163-2116). PMID 9724176 (https://pubmed.ncbi.nlm.nih.gov/9724176). S2CID 35212840 (https://api.semanticscholar.org/CorpusID:35212840).
20. McClurg, Doreen; Norton, Christine (2016-07-27). "What is the best way to manage neurogenic bowel dysfunction?" (https://www.bmj.com/content/354/bmj.i3931). *BMJ*. **354**: i3931. doi:10.1136/bmj.i3931 (https://doi.org/10.1136%2Fbmj.i3931). PMID 27815246 (https://pubmed.ncbi.nlm.nih.gov/27815246). S2CID 28028731 (https://api.semanticscholar.org/CorpusID:28028731). Archived (https://web.archive.org/web/20180929155512/https://www.bmj.com/content/354/bmj.i3931) from the original on 2018-09-29. Retrieved 2018-09-18.
21. Ray, Kausik; McFall, Malcolm (April 2010). "Large bowel enema through colostomy with a Foley catheter: a simple and effective technique" (https://www.ncbi.nlm.nih.gov/pmc/articles/PMC3080060). *Annals of the Royal College of Surgeons of England*. **92** (3): 263. doi:10.1308/rcsann.2010.92.3.263 (https://doi.org/10.1308%2Frcsann.2010.92.3.263). ISSN 1478-7083 (https://www.worldcat.org/issn/1478-7083). PMC 3080060 (https://www.ncbi.nlm.nih.gov/pmc/articles/PMC3080060). PMID 20425893 (https://pubmed.ncbi.nlm.nih.gov/20425893).
22. Facts about Neurogenic Bladder Treatment (https://www.spinabifida.net/5-facts-about-neurogenic-bladder-treatment/)

Retrieved from "https://en.wikipedia.org/w/index.php?title=Neurogenic_bowel_dysfunction&oldid=1118483922"

This page was last edited on 27 October 2022, at 07:17 (UTC).

Text is available under the Creative Commons Attribution-ShareAlike License 3.0; additional terms may apply. By using this site, you agree to the Terms of Use and Privacy Policy. Wikipedia® is a registered trademark of the Wikimedia Foundation, Inc., a non-profit organization.
