# Supplementary material for: Assessment of medical information on irritable bowel syndrome information in Wikipedia and Baidu Encyclopedia: comparative study
Source: PeerJ. 2024 May 24;12:e17264. doi: 10.7717/peerj.17264 (PMC11129691; doi:10.7717/peerj.17264)
Supplement: Data S1 [file peerj-12-17264-s001.zip › σÄƒσoïμò░μì«/Wikipedia/Levator ani syndrome - Wikipedia.pdf]

WIKIPEDIA

Levator ani syndrome

Levator ani syndrome is a condition characterized by burning pain or tenesmus of the rectal or perineal area,[1] caused by spasm of the levator ani muscle.[2][3][4] The genesis of the syndrome is unknown; however, inflammation of the arcus tendon is a possible cause of levator ani syndrome.[5]

Levator ani syndrome	
Other names	Levator spasm, Puborectalis syndrome, Chronic proctalgia, Piriformis syndrome, Pelvic tension myalgia, Levator syndrome, and Proctodynia

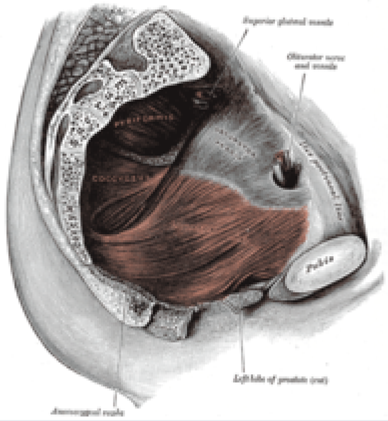

Left levator ani muscle seen from within

Specialty	Gastroenterology
Symptoms	burning anorectal pain or tenesmus
Causes	Painful spasm of the levator ani muscle
Treatment	Walking, pelvic relaxation techniques, massage, warm baths, muscle relaxant medications

Contents

Signs and symptoms
Cause
Diagnosis
Treatment
References
External links

Signs and symptoms

Symptoms include a dull ache more often to the left 2 inches above the anus or higher in the rectum and a feeling of constant rectal pressure or burning. The pain may last for 30 minutes or longer, and is usually described as chronic or intermittent with prolonged periods, in contrast to the brief pain of the related disorder proctalgia fugax. Pain may be worse when sitting than when standing or lying.[6] Precipitating factors include extended sitting, defecation, stress, sexual intercourse, childbirth, and surgery. Palpation of the levator ani muscle may find tenderness.[7]

Cause

Levator ani syndrome is characterized by painful spasm of the levator ani muscle.[2][3][4]

The genesis of the syndrome is unknown, however it has been suggested that inflammation of the arcus tendon is the possible cause of levator ani syndrome.[5] Proctalgia fugax and levator ani syndrome have not been found to be of psychosomatic origin, although stressful events may trigger

attacks.[3] Occurrence of levator ani syndrome is associated with "significant elevations on the hypochondriasis, depression, and hysteria scales of the Minnesota Multiphasic Personality Inventory," which is also the case in general among chronic pain sufferers.[4]

Diagnosis

The diagnosis of levator ani syndrome is clinical, based on the pattern of signs and symptoms. The diagnosis does not require any routine imaging or additional testing, though other causes of rectal pain must be excluded. Suspected levator ani syndrome is confirmed in the presence of chronic or recurrent rectal pain, occurring in episodes that last at least 30 minutes, with tenderness with posterior traction of the puborectalis muscle.

Treatment

The discomfort may be relieved by walking or pelvic relaxation techniques. Other treatments include massage of the muscle, warm baths, muscle relaxant medications such as cyclobenzaprine, therapeutic ultrasound and biofeedback. Electrical stimulation of the levator ani muscle has been used to try to break the spastic cycle. Injection of botulinum toxin A has also been used.

References

1. Rapini, Ronald P.; Bolognia, Jean L.; Jorizzo, Joseph L. (2007). *Dermatology: 2-Volume Set*. St. Louis: Mosby. ISBN 978-1-4160-2999-1.
2. Levator Syndrome, by Parswa Ansari, MD 7/2014, Merck Manuals
3. Giulio Aniello Santoro; Andrzej Paweł Wieczorek; Clive I. Bartram (27 October 2010). *Pelvic Floor Disorders: Imaging and Multidisciplinary Approach to Management* (https://books.google.com/books?id=35aUz5yx4SkC&pg=PA601). Springer. p. 601. ISBN 978-88-470-1542-5.
4. Bharucha AE, Trabuco E (September 2008). "Functional and chronic anorectal and pelvic pain disorders" (https://www.ncbi.nlm.nih.gov/pmc/articles/PMC2676775). *Gastroenterology Clinics of North America*. **37** (3): 685–96, ix. doi:10.1016/j.gtc.2008.06.002 (https://doi.org/10.1016%2Fj.gtc.2008.06.002). PMC 2676775 (https://www.ncbi.nlm.nih.gov/pmc/articles/PMC2676775). PMID 18794003 (https://pubmed.ncbi.nlm.nih.gov/18794003).
5. Park DH, Yoon SG, Kim KU, et al. (May 2005). "Comparison study between electrogalvanic stimulation and local injection therapy in levator ani syndrome". *International Journal of Colorectal Disease*. **20** (3): 272–6. doi:10.1007/s00384-004-0662-9 (https://doi.org/10.1007%2Fs00384-004-0662-9). PMID 15526112 (https://pubmed.ncbi.nlm.nih.gov/15526112).
6. Rao, SS; Bharucha, AE; Chiarioni, G; Felt-Bersma, R; Knowles, C; Malcolm, A; Wald, A (25 March 2016). "Functional Anorectal Disorders" (https://www.ncbi.nlm.nih.gov/pmc/articles/PMC5035713). *Gastroenterology*. doi:10.1053/j.gastro.2016.02.009 (https://doi.org/10.1053%2Fj.gastro.2016.02.009). PMC 5035713 (https://www.ncbi.nlm.nih.gov/pmc/articles/PMC5035713). PMID 27144630 (https://pubmed.ncbi.nlm.nih.gov/27144630).
7. Bharucha, Adil E.; Lee, Tae Hee (October 2016). "Anorectal and Pelvic Pain" (https://www.ncbi.nlm.nih.gov/pmc/articles/PMC5123821). *Mayo Clinic Proceedings* (review). **91** (10): 1471–1486. doi:10.1016/j.mayocp.2016.08.011 (https://doi.org/10.1016%2Fj.mayocp.2016.08.011). ISSN 1942-5546 (https://www.worldcat.org/issn/1942-5546). PMC 5123821 (http

s://www.ncbi.nlm.nih.gov/pmc/articles/PMC5123821). PMID 27712641 (https://pubmed.ncbi.nlm.nih.gov/27712641).

External links

Retrieved from "https://en.wikipedia.org/w/index.php?title=Levator_ani_syndrome&oldid=1069599692"

This page was last edited on 3 February 2022, at 04:03 (UTC).

Text is available under the Creative Commons Attribution-ShareAlike License 3.0; additional terms may apply. By using this site, you agree to the Terms of Use and Privacy Policy. Wikipedia® is a registered trademark of the Wikimedia Foundation, Inc., a non-profit organization.
