# Supplementary material for: Assessment of medical information on irritable bowel syndrome information in Wikipedia and Baidu Encyclopedia: comparative study
Source: PeerJ. 2024 May 24;12:e17264. doi: 10.7717/peerj.17264 (PMC11129691; doi:10.7717/peerj.17264)
Supplement: Data S1 [file peerj-12-17264-s001.zip › σÄƒσoïμò░μì«/Wikipedia/Intestinal malrotation - Wikipedia.pdf]

WIKIPEDIA

Intestinal malrotation

Intestinal malrotation	
Specialty	Medical genetics

Intestinal malrotation is a congenital anomaly of rotation of the midgut. It occurs during the first trimester as the fetal gut undergoes a complex series of growth and development. Malrotation can lead to a dangerous complication called volvulus. Malrotation can refer to a spectrum of abnormal intestinal positioning, often including:

- The small intestine found predominantly on the right side of the abdomen
- The cecum displaced from its usual position in the right lower quadrant into the epigastrium or right hypochondrium
- An absent or displaced ligament of Treitz
- Fibrous peritoneal bands called bands of Ladd running across the vertical portion of the duodenum
- An unusually narrow, stalk-like mesentery

The position of the intestines, narrow mesentery and Ladd's bands can contribute to several severe gastrointestinal conditions. The narrow mesentery predisposes some cases of malrotation to midgut volvulus, a twisting of the entire small bowel that can obstruct the mesenteric blood vessels leading to intestinal ischemia, necrosis, and death if not promptly treated. The fibrous Ladd's bands can constrict the duodenum, leading to intestinal obstruction.

Contents

Signs and symptoms
Complications
Causes
Diagnosis
Treatment
See also
References
External links

Signs and symptoms

Signs and symptoms of malrotation vary depending on if the patient is suffering from an acute volvulus or experiencing chronic symptoms.

- If the patient, most often an infant, presents acutely with midgut volvulus, it is usually manifested by bilious vomiting, crampy abdominal pain, abdominal distention, and in late cases, the passage of blood and mucus in their stools.

- Patients with chronic, uncorrected or undiagnosed malrotation can have recurrent abdominal pain and vomiting.
- Malrotation may be asymptomatic.

Complications

Intestinal malrotation can lead to a number of disease manifestations and complications such as:

- Acute midgut volvulus
- Chronic midgut volvulus
- Acute duodenal obstruction
- Chronic duodenal obstruction
- Short bowel syndrome, in cases of volvulus with intestinal necrosis
- Death, in cases of volvulus with pan-necrosis of the bowel, severe septic shock or hypovolemic shock
- Malabsorption
- Chronic motility issues
- Internal herniation
- Superior mesenteric artery syndrome

Causes

The exact cause of intestinal malrotation is unknown. It is not definitively associated with a particular gene, but there is some evidence of recurrence in families.[2]

Diagnosis

Malrotation is most often diagnosed during infancy, however, some cases are not discovered until later in childhood or even adulthood.[3]

With acutely ill patients, consider emergency surgery laparotomy if there is a high index of suspicion.

In cases of volvulus, plain radiography may demonstrate signs of duodenal obstruction with dilatation of the proximal duodenum and stomach but it is often non-specific. Ultrasonography may be useful in some cases of volvulus, depicting a "whirlpool sign" where the superior mesenteric artery and superior mesenteric vein have twisted.[4]

Upper gastrointestinal series is the modality of choice for the evaluation of malrotation, as it will often show an abnormal position of the duodenum and duodeno-jejunal flexure (ligament of Treitz). In cases of malrotation complicated with volvulus, upper GI demonstrates a corkscrew appearance of the distal duodenum and jejunum. In cases of obstructing Ladd's bands, upper GI may reveal a duodenal obstruction. Although upper GI series is regarded as the most reliable diagnostic test for intestinal malrotation, false negatives may occur in 5% of cases.[4] False negatives are most frequently attributed to radiographer error, uncooperative pediatric patients, or variations in intestinal positioning. In equivocal cases physicians may wish to repeat the upper GI

or consider additional diagnostic modalities. Lower gastrointestinal series, may be helpful in some patients by showing the caecum at an abnormal location. CT scan and magnetic resonance imaging may also aide in the diagnosis of equivocal cases.

The incidence of intestinal malrotation in infants with omphalocoele is low. Therefore, there is little evidence to support the screening for intestinal malrotation in infants with omphalocoele.[5]

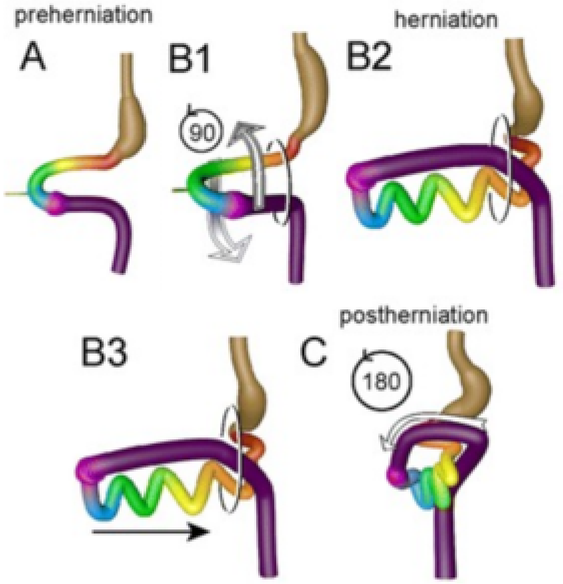

Diagram showing the process by which the intestine rotates and herniates during normal development. From panel A to B (left-sided views), the midgut loop rotates 90° in a counterclockwise direction, so that its position changes from midsagittal (A) to transverse (B1). The small intestine forms loops (B2) and slides back into the abdomen (B3) during resolution of the hernia. Meanwhile, the cecum moves from the left to the right side, which represents the additional 180° counterclockwise rotation of the intestine (C, central view).[1]

Treatment

Prompt surgical treatment is necessary for intestinal malrotation when volvulus has occurred:

- First, the patient is resuscitated with fluids to stabilize them for surgery
- The volvulus is corrected (counterclockwise rotation of the bowel),
- The fibrous Ladd's bands over the duodenum are cut,
- The mesenteric pedicle is widened by separation of the duodenum and cecum,
- The small and large bowels are placed in a position that reduces their risk of future volvulus

With this condition the appendix is often on the wrong side of the body and therefore removed as a precautionary measure during the surgical procedure.

This surgical technique is known as the "Ladd's procedure", after Dr. William Ladd.[6][7] Long-term research on the Ladd's procedure indicates that even after surgery, some patients are susceptible to GI issues and may need further surgery.[8]

See also

- Situs inversus, a congenital condition in which the major visceral organs are reversed or mirrored from their normal positions.

References

1. Soffers JH, Hikspoors JP, Mekonen HK, Koehler SE, Lamers WH (August 2015). "The growth pattern of the human intestine and its mesentery" (https://www.ncbi.nlm.nih.gov/pmc/articles/PMC4546136). *BMC Developmental Biology*. **15** (1): 31. doi:10.1186/s12861-015-0081-x (https://doi.org/10.1186%2Fs12861-015-0081-x). PMC 4546136 (https://www.ncbi.nlm.nih.gov/pmc/articles/PMC4546136). PMID 26297675 (https://pubmed.ncbi.nlm.nih.gov/26297675).
2. Stalker HJ, Chitayat D (September 1992). "Familial intestinal malrotation with midgut volvulus and facial anomalies: a disorder involving a gene controlling the normal gut rotation?". *American Journal of Medical Genetics*. **44** (1): 46—7.

doi:10.1002/ajmg.1320440111 (https://doi.org/10.1002%2Fajmg.1320440111). PMID 1519649 (https://pubmed.ncbi.nlm.nih.gov/1519649).

3. Dietz DW, Walsh RM, Grundfest-Broniatowski S, Lavery IC, Fazio VW, Vogt DP (October 2002). "Intestinal malrotation: a rare but important cause of bowel obstruction in adults". *Diseases of the Colon and Rectum*. **45** (10): 1381–6. doi:10.1007/s10350-004-6429-0 (https://doi.org/10.1007%2Fs10350-004-6429-0). PMID 12394439 (https://pubmed.ncbi.nlm.nih.gov/12394439). S2CID 23269321 (https://api.semanticscholar.org/CorpusID:23269321).
4. Yeh WC, Wang HP, Chen C, Wang HH, Wu MS, Lin JT (June 1999). "Preoperative sonographic diagnosis of midgut malrotation with volvulus in adults: the "whirlpool" sign". *Journal of Clinical Ultrasound*. **27** (5): 279–83. doi:10.1002/(SICI)1097-0096(199906)27:5<279::AID-JCU8>3.0.CO;2-G (https://doi.org/10.1002%2F%28SICI%291097-0096%28199906%2927%3A5%3C279%3A%3AAID-JCU8%3E3.0.CO%3B2-G). PMID 10355892 (https://pubmed.ncbi.nlm.nih.gov/10355892).
5. Lauriti, Giuseppe; Miscia, Maria Enrica; Cascini, Valentina; Chiesa, Pierluigi Lelli; Pierro, Agostino; Zani, Augusto (March 2019). "Intestinal malrotation in infants with omphalocele: A systematic review and meta-analysis". *Journal of Pediatric Surgery*. **54** (3): 378–382. doi:10.1016/j.jpedsurg.2018.09.010 (https://doi.org/10.1016%2Fj.jpedsurg.2018.09.010). ISSN 0022-3468 (https://www.worldcat.org/issn/0022-3468). PMID 30309732 (https://pubmed.ncbi.nlm.nih.gov/30309732). S2CID 52961238 (https://api.semanticscholar.org/CorpusID:52961238).
6. Ladd WE (1936). "Surgical Diseases of the Alimentary Tract in Infants". *N Engl J Med*. **215** (16): 705–8. doi:10.1056/NEJM193610152151604 (https://doi.org/10.1056%2FNEJM193610152151604).
7. Bass KD, Rothenberg SS, Chang JH (February 1998). "Laparoscopic Ladd's procedure in infants with malrotation". *Journal of Pediatric Surgery*. **33** (2): 279–81. doi:10.1016/S0022-3468(98)90447-X (https://doi.org/10.1016%2FS0022-3468%2898%2990447-X). PMID 9498402 (https://pubmed.ncbi.nlm.nih.gov/9498402).
8. Murphy FL, Sparnon AL (April 2006). "Long-term complications following intestinal malrotation and the Ladd's procedure: a 15 year review". *Pediatric Surgery International*. **22** (4): 326–9. doi:10.1007/s00383-006-1653-4 (https://doi.org/10.1007%2Fs00383-006-1653-4). PMID 16518597 (https://pubmed.ncbi.nlm.nih.gov/16518597). S2CID 22816456 (https://api.semanticscholar.org/CorpusID:22816456).

External links

Retrieved from "https://en.wikipedia.org/w/index.php?title=Intestinal_malrotation&oldid=1111263392"

This page was last edited on 20 September 2022, at 03:11 (UTC).

Text is available under the Creative Commons Attribution-ShareAlike License 3.0; additional terms may apply. By using this site, you agree to the Terms of Use and Privacy Policy. Wikipedia® is a registered trademark of the Wikimedia Foundation, Inc., a non-profit organization.
