# Supplementary material for: Assessment of medical information on irritable bowel syndrome information in Wikipedia and Baidu Encyclopedia: comparative study
Source: PeerJ. 2024 May 24;12:e17264. doi: 10.7717/peerj.17264 (PMC11129691; doi:10.7717/peerj.17264)
Supplement: Data S1 [file peerj-12-17264-s001.zip › σÄƒσoïμò░μì«/Wikipedia/1-Irritable bowel syndrome - Wikipedia.pdf]

WIKIPEDIA

Irritable bowel syndrome

Irritable bowel syndrome	
Other names	Spastic colon, nervous colon, mucous colitis, spastic bowel[1]
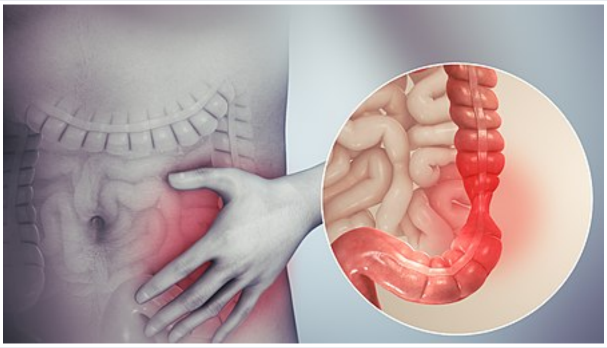 Drawing of the pain of IBS	
Specialty	Gastroenterology
Symptoms	Diarrhea, constipation, abdominal pain[1]
Usual onset	Before 45 years old[1]
Duration	Long term[2]
Causes	Unknown[2]
Diagnostic method	Based on symptoms, exclusion of other diseases[3]
Differential diagnosis	Celiac disease, giardiasis, non-celiac gluten sensitivity, microscopic colitis, inflammatory bowel disease, bile acid malabsorption, colon cancer[3][4]
Treatment	Symptomatic (dietary changes, medication, human milk oligosaccharides, probiotics, counseling)[5]
Prognosis	Normal life expectancy[6]
Frequency	10–15% (developed world)[1][7] and 15–45% (globally) [8]

Irritable bowel syndrome (**IBS**) is a "disorder of brain-gut interaction" characterized by a group of symptoms that commonly include abdominal pain and or abdominal bloating and changes in the consistency of bowel movements.[1] These symptoms may occur over a long time, sometimes for years.[2] IBS can negatively affect quality of life and may result in missed school or work (absenteeism) or reduced productivity at work (presenteeism).[9] Disorders such as anxiety, major depression, and chronic fatigue syndrome are common among people with IBS.[1][10][note 1]

The causes of IBS may well be multi-factorial.[2] Theories include combinations of "gut–brain axis" problems, alterations in gut motility, visceral hypersensitivity, infections including small intestinal bacterial overgrowth, neurotransmitters, genetic factors, and food sensitivity.[2] Onset may be triggered by an intestinal infection[11] ("post-infectious irritable bowel syndrome") or a stressful life event.[12]

Diagnosis is based on symptoms in the absence of worrisome features and once other potential conditions have been ruled out.[3] Worrisome or "alarm" features include onset at greater than 50 years of age, weight loss, blood in the stool, or a family history of inflammatory bowel disease.[3] Other conditions that may present similarly include celiac disease, microscopic colitis, inflammatory bowel disease, bile acid malabsorption, and colon cancer.[3]

Treatment of IBS is carried out to improve symptoms and can be very effective. This may include dietary changes, medication, probiotics, and counseling.[5][13] Dietary measures include increasing soluble fiber intake, or a diet low in fermentable oligosaccharides, disaccharides, monosaccharides, and polyols (FODMAPs). The "low FODMAP" diet is meant for short to medium term use and is not intended as a life-long therapy.[3][14][15] The medication loperamide may be used to help with diarrhea while laxatives may be used to help with constipation.[3] There is strong clinical-trial evidence for the use of antidepressants, often in lower doses than that used for depression or anxiety, even in patients without comorbid mood disorder. Tricyclic antidepressants

such as amitriptyline or nortriptyline and medications from the selective serotonin reuptake inhibitor (SSRI) group may improve overall symptoms and reduce pain.[3] Patient education and a good doctor–patient relationship are an important part of care.[3][16]

About 10–15% of people in the developed world are believed to be affected by IBS.[1][7] The prevalence varies according to country (from 1.1% to 45.0%) and criteria used to define IBS; however pooling the results of multiple studies gives an estimate of 11.2%.[8] It is more common in South America and less common in Southeast Asia.[3] In the Western world it is twice as common in women as men and typically occurs before age 45.[1] However, women in East Asia may not be more likely than men to have IBS, according to several studies.[17] The condition appears to become less common with age.[3] IBS does not affect life expectancy or lead to other serious diseases.[6] The first description of the condition was in 1820, while the current term *irritable bowel syndrome* came into use in 1944.[18]

Contents

Classification

Signs and symptoms

Cause
- Risk factors
- Stress
- Post-infectious
- Bacteria
- Fungus
- Protozoa
- Vitamin D
- Genetics

Mechanism

Diagnosis
- Rome criteria
- Differential diagnosis
- Investigations
- Misdiagnosis
- Comorbidities

Management
- Diet
- Physical Activity
- Medication
- Psychological therapies
- Vagus nerve stimulation
- Alternative medicine

Epidemiology
- Gender

History
Society and culture
Economics
Research
In other species
Notes
References
External links

Classification

IBS can be classified as diarrhea-predominant (IBS-D), constipation-predominant (IBS-C), with mixed/alternating stool pattern (IBS-M/IBS-A) or pain-predominant.[19] In some individuals, IBS may have an acute onset and develop after an infectious illness characterized by two or more of: fever, vomiting, diarrhea, or positive stool culture. This post-infective syndrome has consequently been termed "post-infectious IBS" (IBS-PI).[20][21][22][23]

Signs and symptoms

The primary symptoms of IBS are abdominal pain or discomfort in association with frequent diarrhea or constipation and a change in bowel habits.[24] Symptoms usually are experienced as acute attacks that subside within one day, but recurrent attacks are likely.[25] There may also be urgency for bowel movements, a feeling of incomplete evacuation (tenesmus) or bloating.[26] In some cases, the symptoms are relieved by bowel movements.[16] People with IBS, more commonly than others, have gastroesophageal reflux, symptoms relating to the genitourinary system, fibromyalgia, headache, backache, and psychiatric symptoms such as depression and anxiety.[10][26] About a third of adults who have IBS also report sexual dysfunction, typically in the form of a reduction in libido.[27]

Cause

While the causes of IBS are still unknown, it is believed that the entire gut–brain axis is affected.[28][29] Recent findings suggest that an allergy triggered peripheral immune mechanism may underlie the symptoms associated with abdominal pain in patients with irritable bowel syndrome.[30] IBS is more prevalent in obese patients.[31]

Risk factors

The risk of developing IBS increases six-fold after acute gastrointestinal infection.[32] Post-infection,[32] further risk factors are young age, prolonged fever, anxiety, and depression.[33] Psychological factors, such as depression or anxiety, have not been shown to cause or influence the onset of IBS, but may play a role in the persistence and perceived severity of symptoms.[34] Nevertheless, they may worsen IBS symptoms and quality of life.[34] Antibiotic use also appears to

increase the risk of developing IBS.[35] Research has found that genetic defects in innate immunity and epithelial homeostasis increase the risk of developing both post-infectious as well as other forms of IBS.[36]

Stress

Publications suggesting the role of the brain–gut axis appeared in the 1990s[37] and childhood physical and psychological abuse is often associated with the development of IBS.[38] It is believed that psychological stress may trigger IBS in predisposed individuals.[39]

Given the high levels of anxiety experienced by people with IBS and the overlap with conditions such as fibromyalgia and chronic fatigue syndrome, a potential explanation for IBS involves a disruption of the stress system. The stress response in the body involves the hypothalamic–pituitary–adrenal axis (HPA) and the sympathetic nervous system, both of which have been shown to operate abnormally in people with IBS. Psychiatric illness or anxiety precedes IBS symptoms in two-thirds of people with IBS, and psychological traits predispose previously healthy people to developing IBS after gastroenteritis.[40][41]

Post-infectious

Approximately 10 percent of IBS cases are triggered by an acute gastroenteritis infection.[42] The CdtB toxin is produced by bacteria causing gastroenteritis and the host may develop an autoimmunity when host antibodies to CdtB cross-react with vinculin.[43] Genetic defects relating to the innate immune system and epithelial barrier as well as high stress and anxiety levels appear to increase the risk of developing post-infectious IBS. Post-infectious IBS usually manifests itself as the diarrhea-predominant subtype. Evidence has demonstrated that the release of high levels of proinflammatory cytokines during acute enteric infection causes increased gut permeability leading to translocation of the commensal bacteria across the epithelial barrier; this in turn can result in significant damage to local tissues, which can develop into chronic gut abnormalities in sensitive individuals. However, increased gut permeability is strongly associated with IBS regardless of whether IBS was initiated by an infection or not.[36] A link between small intestinal bacterial overgrowth and tropical sprue has been proposed to be involved as a cause of post-infectious IBS.[44]

Bacteria

Small intestinal bacterial overgrowth (SIBO) occurs with greater frequency in people who have been diagnosed with IBS compared to healthy controls.[45] SIBO is most common in diarrhea-predominate IBS but also occurs in constipation-predominant IBS more frequently than healthy controls. Symptoms of SIBO include bloating, abdominal pain, diarrhea or constipation among others. IBS may be the result of the immune system interacting abnormally with gut microbiota resulting in an abnormal cytokine signalling profile.[46]

Certain bacteria are found in lower or higher abundance when compared with healthy individuals. Generally Bacteroidota, Bacillota, and Pseudomonadota are increased and Actinomycetota, *Bifidobacteria*, and *Lactobacillus* are decreased. Within the human gut, there are common phyla found. The most common is Bacillota. This includes *Lactobacillus*, which is found to have a decrease in people with IBS, and *Streptococcus*, which is shown to have an increase in abundance. Within this phylum, species in the class Clostridia are shown to have an increase, specifically *Ruminococcus* and *Dorea*. The family Lachnospiraceae presents an increase in IBS-D patients. The second most common phylum is Bacteroidota. In people with IBS, the Bacteroidota phylum

has been shown to have an overall decrease, but an increase in the genus *Bacteroides*. IBS-D shows a decrease for the phylum Actinomycetota and an increase in Pseudomonadota, specifically in the family Enterobacteriaceae.[47]

Fungus

There is growing evidence that alterations of gut microbiota (dysbiosis) are associated with the intestinal manifestations of IBS, but also with the psychiatric morbidity that coexists in up to 80% of people with IBS.[48] The role of the gut mycobiota, and especially of the abnormal proliferation of the yeast *Candida albicans* in some people with IBS, was under investigation as of 2005.[49]

Protozoa

Protozoal infections can cause symptoms that mirror specific IBS subtypes,[52] e.g., infection by certain substypes of *Blastocystis hominis* (blastocystosis).[53][54] Many people regard these organisms as incidental findings, and unrelated to symptoms of IBS.

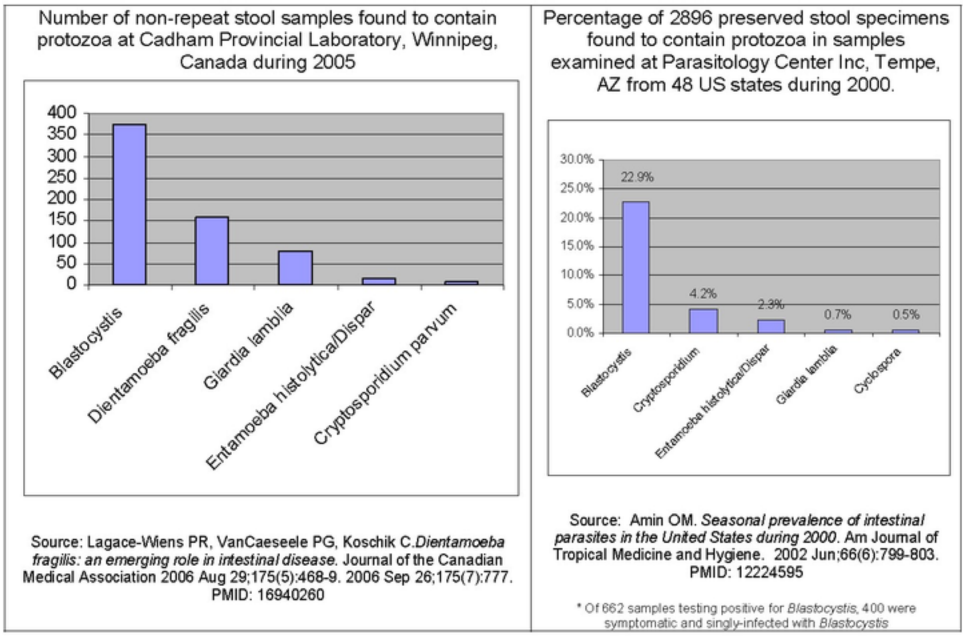

Prevalence of protozoal infections in industrialized countries (United States and Canada) in the 21st century[50][51]

As of 2017, evidence indicates that blastocystis colonisation occurs more commonly in IBS affected individuals and is a possible risk factor for developing IBS.[55] *Dientamoeba fragilis* has also been considered a possible organism to study, though it is also found in people without IBS.[56]

Vitamin D

Vitamin D deficiency is more common in individuals affected by irritable bowel syndrome.[57][58] Vitamin D is involved in regulating triggers for IBS including the gut microbiome, inflammatory processes and immune responses, as well as psychosocial factors.[59]

Genetics

SCN5A mutations are found in a small number of people who have IBS, particularly the constipation-predominant variant (IBS-C).[60][61] The resulting defect leads to disruption in bowel function, by affecting the Nav1.5 channel, in smooth muscle of the colon and pacemaker cells.

Mechanism

Genetic, environmental, and psychological factors seem to be important in the development of IBS. Studies have shown that IBS has a genetic component even though there is a predominant influence of environmental factors.[62]

Dysregulated brain-gut axis, abnormal serotonin/5-hydroxytryptamine (5-HT) metabolism, and high density of mucosal nerve fibers or neurites in the intestines have been implicated in the mechanisms of IBS. A number of 5-HT receptor subtypes were involved in the IBS symptoms, including 5-HT_3, 5-HT_4, and 5-HT_7 receptors. High levels of 5-HT_7 receptor-expressing mucosal nerve fibers were observed in the colon of IBS patients. A role of 5-HT7 receptor in intestinal hyperalgesia was demonstrated in mouse models with visceral hypersensitivity, of which a novel 5-HT_7 receptor antagonist administered perorally reduced intestinal pain levels.[63]

There is evidence that abnormalities occur in the gut flora of individuals who have IBS, such as reduced diversity, a decrease in bacteria belonging to the phylum Bacteroidota, and an increase in those belonging to the phylum Bacillota.[48] The changes in gut flora are most profound in individuals who have diarrhoea predominant IBS. Antibodies against common components (namely flagellin) of the commensal gut flora are a common occurrence in IBS affected individuals.[64]

Chronic low-grade inflammation commonly occurs in IBS affected individuals with abnormalities found including increased enterochromaffin cells, intraepithelial lymphocytes, and mast cells resulting in chronic immune-mediated inflammation of the gut mucosa.[28][65] IBS has been reported in greater quantities in multigenerational families with IBS than in the regular population.[66] It is believed that psychological stress can induce increased inflammation and thereby cause IBS to develop in predisposed individuals.[39]

Diagnosis

No specific laboratory or imaging tests can diagnose irritable bowel syndrome. Diagnosis should be based on symptoms, the exclusion of worrisome features, and the performance of specific investigations to rule out organic diseases that may present similar symptoms.[3][67]

The recommendations for physicians are to minimize the use of medical investigations.[68] Rome criteria (see below) are usually used. They allow the diagnosis to be based only on symptoms, but no criteria based solely on symptoms is sufficiently accurate to diagnose IBS.[69][70] Worrisome features include onset at greater than 50 years of age, weight loss, blood in the stool, iron-deficiency anemia, or a family history of colon cancer, celiac disease, or inflammatory bowel disease.[3] The criteria for selecting tests and investigations also depends on the level of available medical resources.[34]

Rome criteria

The Rome criteria are consensus guidelines, initially released in 1994 and updated periodically since then. These may pertain more closely to clinical trials as in practice, patient symptoms may vary considerably. The Rome IV criteria (2016) for IBS include recurrent abdominal pain, on

average, at least one day/week in the last three months, associated with two or more of the following further criteria:[71]

- Related to defecation
- Associated with a change in frequency of stool
- Associated with a change in form (appearance) of stool

The algorithm may include additional tests to guard against misdiagnosis of other diseases as IBS. Such "red flag" symptoms may include weight loss, gastrointestinal bleeding, anemia, or nocturnal symptoms. However, red flag conditions may not always contribute to accuracy in diagnosis; for instance, as many as 31% of people with IBS have blood in their stool, many possibly from hemorrhoidal bleeding.[72]

The diagnostic algorithm identifies a name that can be applied to the person's condition based on the combination of symptoms of diarrhea, abdominal pain, and constipation. For example, the statement "50% of returning travellers had developed functional diarrhea while 25% had developed IBS" would mean half the travellers had diarrhea while a quarter had diarrhea with abdominal pain. While some researchers believe this categorization system will help physicians understand IBS, others have questioned the value of the system and suggested all people with IBS have the same underlying disease but with different symptoms.[73]

Differential diagnosis

Colon cancer, inflammatory bowel disease, thyroid disorders (hyperthyroidism or hypothyroidism), and giardiasis can all feature abnormal defecation and abdominal pain. Less common causes of this symptom profile are carcinoid syndrome, microscopic colitis, bacterial overgrowth, and eosinophilic gastroenteritis; IBS is, however, a common presentation, and testing for these conditions would yield low numbers of positive results, so it is considered difficult to justify the expense.[74] Conditions that may present similarly include celiac disease, bile acid malabsorption, colon cancer, and dyssynergic defecation.[3]

Ruling out parasitic infections, lactose intolerance, small intestinal bacterial overgrowth, and coeliac disease is recommended before a diagnosis of irritable bowel syndrome is made.[67] An upper endoscopy with small bowel biopsies is necessary to identify the presence of celiac disease.[75] An ileocolonoscopy with biopsies is useful to exclude Crohn's disease and ulcerative colitis (Inflammatory bowel disease).[75]

Some people, managed for years for IBS, may have non-celiac gluten sensitivity (NCGS).[4] Gastrointestinal symptoms of IBS are clinically indistinguishable from those of NCGS, but the presence of any of the following non-intestinal manifestations suggest a possible NCGS: headache or migraine, "foggy mind", chronic fatigue,[76] fibromyalgia,[77][78][79] joint and muscle pain,[76][77][80] leg or arm numbness,[76][77][80] tingling of the extremities,[76][80] dermatitis (eczema or skin rash),[76][80] atopic disorders,[76] allergy to one or more inhalants, foods or metals[76][77] (such as mites, graminaceae, parietaria, cat or dog hair, shellfish, or nickel[77]), depression,[76][77][80] anxiety,[77] anemia,[76][80] iron-deficiency anemia, folate deficiency, asthma, rhinitis, eating disorders,[77] neuropsychiatric disorders (such as schizophrenia,[80][81] autism,[77][80][81] peripheral neuropathy,[80][81] ataxia,[81] attention deficit hyperactivity

disorder[76]) or autoimmune diseases.[76] An improvement with a gluten-free diet of immune-mediated symptoms, including autoimmune diseases, once having reasonably ruled out coeliac disease and wheat allergy, is another way to realize a differential diagnosis.[76]

Investigations

Investigations are performed to exclude other conditions:

- Stool microscopy and culture (to exclude infectious conditions)
- Blood tests: Full blood examination, liver function tests, erythrocyte sedimentation rate, and serological testing for coeliac disease
- Abdominal ultrasound (to exclude gallstones and other biliary tract diseases)
- Endoscopy and biopsies (to exclude peptic ulcer disease, coeliac disease, inflammatory bowel disease, and malignancies)
- Hydrogen breath testing (to exclude fructose and lactose malabsorption)

Misdiagnosis

People with IBS are at increased risk of being given inappropriate surgeries such as appendectomy, cholecystectomy, and hysterectomy due to being misdiagnosed as other medical conditions.[82] Some common examples of misdiagnosis include infectious diseases, coeliac disease,[83] *Helicobacter pylori*,[84][85] parasites (non-protozoal).[52][86][87] The American College of Gastroenterology recommends all people with symptoms of IBS be tested for coeliac disease.[88]

Bile acid malabsorption is also sometimes missed in people with diarrhea-predominant IBS. SeHCAT tests suggest around 30% of people with D-IBS have this condition, and most respond to bile acid sequestrants.[89]

Comorbidities

Several medical conditions, or comorbidities, appear with greater frequency in people with IBS.

- Neurological/psychiatric: A study of 97,593 individuals with IBS identified comorbidities such as headache, fibromyalgia, and depression.[90] IBS occurs in 51% of people with chronic fatigue syndrome and 49% of people with fibromyalgia, and psychiatric disorders occur in 94% of people with IBS.[10][note 1]
- Channelopathy and muscular dystrophy: IBS and functional GI diseases are comorbidities of genetic channelopathies that cause cardiac conduction defects and neuromuscular dysfunction, and result also in alterations in GI motility, secretion, and sensation.[91] Similarly, IBS and FBD are highly prevalent in myotonic muscle dystrophies. Digestive symptoms may be the first sign of dystrophic disease and may precede the musculo-skeletal features by up to 10 years.[92]
- Inflammatory bowel disease: IBS may be marginally associated with inflammatory bowel disease.[93] Researchers have found some correlation between IBS and IBD,[94] noting that people with IBD experience IBS-like symptoms when their IBD is in remission.[95][96] A three-year study found that patients diagnosed with IBS were 16.3 times more likely to be diagnosed with IBD during the study period, although this is likely due to an initial misdiagnosis.[97]

- Abdominal surgery: People with IBS were at increased risk of having unnecessary gall bladder removal surgery not due to an increased risk of gallstones, but rather to abdominal pain, awareness of having gallstones, and inappropriate surgical indications.[98] These people also are 87% more likely to undergo abdominal and pelvic surgery and three times more likely to undergo gallbladder surgery.[99] Also, people with IBS were twice as likely to undergo hysterectomy.[100]
- Endometriosis: One study reported a statistically significant link between migraine headaches, IBS, and endometriosis.[101]
- Other chronic disorders: Interstitial cystitis may be associated with other chronic pain syndromes, such as irritable bowel syndrome and fibromyalgia. The connection between these syndromes is unknown.[102]

Management

A number of treatments have been found to be effective, including fiber, talk therapy, antispasmodic and antidepressant medication, and peppermint oil.[103][104][105]

Diet

FODMAP

FODMAPs are short-chain carbohydrates that are poorly absorbed in the small intestine. A 2018 systematic review found that although there is evidence of improved IBS symptoms with a low FODMAP diet, the evidence is of very low quality.[106] Symptoms most likely to improve include urgency, flatulence, bloating,[107] abdominal pain, and altered stool output. One national guideline advises a low FODMAP diet for managing IBS when other dietary and lifestyle measures have been unsuccessful.[108] The diet restricts various carbohydrates which are poorly absorbed in the small intestine, as well as fructose and lactose, which are similarly poorly absorbed in those with intolerances to them. Reduction of fructose and fructan has been shown to reduce IBS symptoms in a dose-dependent manner in people with fructose malabsorption and IBS.[109]

FODMAPs are fermentable oligo-, di-, monosaccharides and polyols, which are poorly absorbed in the small intestine and subsequently fermented by the bacteria in the distal small and proximal large intestine. This is a normal phenomenon, common to everyone. The resultant production of gas potentially results in bloating and flatulence.[110] Although FODMAPs can produce certain digestive discomfort in some people, not only do they not cause intestinal inflammation, but they help avoid it, because they produce beneficial alterations in the intestinal flora that contribute to maintaining the good health of the colon.[111][112][113] FODMAPs are not the cause of irritable bowel syndrome nor other functional gastrointestinal disorders, but rather a person develops symptoms when the underlying bowel response is exaggerated or abnormal.[110]

A low-FODMAP diet consists of restricting them from the diet. They are globally trimmed, rather than individually, which is more successful than for example restricting only fructose and fructans, which are also FODMAPs, as is recommended for those with fructose malabsorption.[110]

A low-FODMAP diet might help to improve short-term digestive symptoms in adults with irritable bowel syndrome,[114][108][115][15] but its long-term follow-up can have negative effects because it causes a detrimental impact on the gut microbiota and metabolome.[116][108][15][117] It should only

be used for short periods of time and under the advice of a specialist.[118] A low-FODMAP diet is highly restrictive in various groups of nutrients and can be impractical to follow in the long-term.[119] More studies are needed to assess the true impact of this diet on health.[108][15]

In addition, the use of a low-FODMAP diet without verifying the diagnosis of IBS may result in misdiagnosis of other conditions such as celiac disease.[120] Since the consumption of gluten is suppressed or reduced with a low-FODMAP diet, the improvement of the digestive symptoms with this diet may not be related to the withdrawal of the FODMAPs, but of gluten, indicating the presence of unrecognized celiac disease, avoiding its diagnosis and correct treatment, with the consequent risk of several serious health complications, including various types of cancer.[120][121]

Fiber

Some evidence suggests soluble fiber supplementation (e.g., psyllium/ispagula husk) is effective.[14] It acts as a bulking agent, and for many people with IBS-D, allows for a more consistent stool. For people with IBS-C, it seems to allow for a softer, moister, more easily passable stool.

However, insoluble fiber (e.g., bran) has not been found to be effective for IBS.[122][123] In some people, insoluble fiber supplementation may aggravate symptoms.[124][125]

Fiber might be beneficial in those who have a predominance of constipation. In people who have IBS-C, soluble fiber can reduce overall symptoms but will not reduce pain. The research supporting dietary fiber contains conflicting small studies complicated by the heterogeneity of types of fiber and doses used.[126]

One meta-analysis found only soluble fiber improved global symptoms of irritable bowel, but neither type of fiber reduced pain.[126] An updated meta-analysis by the same authors also found soluble fiber reduced symptoms, while insoluble fiber worsened symptoms in some cases.[127] Positive studies have used 10–30 grams per day of ispaghula (psyllium).[128][129] One study specifically examined the effect of dose, and found 20 g of ispaghula (psyllium) were better than 10 g and equivalent to 30 g per day.[130]

Physical Activity

Recent studies have demonstrated the potential beneficial effects of Physical activity on irritable bowel syndrome. Some randomised controlled trials (RCTs) have demonstrated a beneficial effect of physical activity on IBS symptoms. Three RCTs showed a significant improvement in Irritable Bowel Syndrome – Severity Scoring System, while 1 RCT showed a significant improvement only in symptoms of constipation.[131] In light of this, the latest British Society of Gastroenterology guidelines on the management of IBS have stated that all patients with IBS should be advised to take regular exercise (strong recommendation, weak certainty evidence),[132] whereas the American College of Gastroenterology guidelines have suggested with a lower certainty of evidence.[133] Exercise is Medicine recently provided simple practical indications based on world health organization guidelines,[134] which should be followed when physicians prescribing exercise training. As shown by the previous studies, a good Physical activity prescription during the visit could significantly improve patients' adherence and, consequently, lead to a significant clinical benefit for symptoms of irritable bowel syndrome.[131]

Medication

Medications that may be useful include antispasmodics such as dicyclomine and antidepressants.[135] Both H1-antihistamines and mast cell stabilizers have shown efficacy in reducing pain associated with visceral hypersensitivity in IBS.[28]

Serotonergic agents

A number of 5-HT3 antagonists or 5-HT4 agonists were proposed clinically to treat diarrhea-predominant IBS and constipation-predominant IBS, respectively. However, severe side effects have resulted in its withdrawal by food and drug administration and are now prescribed under emergency investigational drug protocol.[136] Other 5-HT receptor subtypes, such as 5-HT7 receptor, have yet to be developed.

Laxatives

For people who do not adequately respond to dietary fiber, osmotic laxatives such as polyethylene glycol, sorbitol, and lactulose can help avoid "cathartic colon" which has been associated with stimulant laxatives.[137] Lubiprostone is a gastrointestinal agent used for the treatment of constipation-predominant IBS.[138]

Antispasmodics

The use of antispasmodic drugs (e.g., anticholinergics such as hyoscyamine or dicyclomine) may help people who have cramps or diarrhea. A meta-analysis by the Cochrane Collaboration concludes if seven people are treated with antispasmodics, one of them will benefit.[135] Antispasmodics can be divided into two groups: neurotropics and musculotropics. Musculotropics, such as mebeverine, act directly at the smooth muscle of the gastrointestinal tract, relieving spasm without affecting normal gut motility. Since this action is not mediated by the autonomic nervous system, the usual anticholinergic side effects are absent.[139] The antispasmodic otilonium may also be useful.[140]

Discontinuation of proton pump inhibitors

Proton-pump inhibitors (PPIs) used to suppress stomach acid production may cause small intestinal bacterial overgrowth (SIBO) leading to IBS symptoms.[141] Discontinuation of PPIs in selected individuals has been recommended as it may lead to an improvement or resolution of IBS symptoms.[142]

Antidepressants

Evidence is conflicting about the benefit of antidepressants in IBS. Some meta-analyses have found a benefit, while others have not.[143] There is good evidence that low doses of tricyclic antidepressants (TCAs) can be effective for IBS.[135][144] With TCAs, about one in three people improve.[145]

However, the evidence is less robust for the effectiveness of other antidepressant classes such as selective serotonin reuptake inhibitor antidepressants (SSRIs). Because of their serotonergic effect, SSRIs have been studied in IBS, especially for people who are constipation predominant. As

of 2015, the evidence indicates that SSRIs do not help.[146] Antidepressants are not effective for IBS in people with depression, possibly because lower doses of antidepressants than the doses used to treat depression are required for relief of IBS.[147]

Other agents

Magnesium aluminum silicates and alverine citrate drugs can be effective for IBS.[148][125]

Rifaximin may be useful as a treatment for IBS symptoms, including abdominal bloating and flatulence, although relief of abdominal distension is delayed.[39][149] It is especially useful where small intestinal bacterial overgrowth is involved.[39]

In individuals with IBS and low levels of vitamin D supplementation is recommended. Some evidence suggests that vitamin D supplementation may improve symptoms of IBS, but further research is needed before it can be recommended as a specific treatment for IBS.[57][58]

Psychological therapies

There is low quality evidence from studies with poor methodological quality that psychological therapies can be effective in the treatment of IBS.[147] Reducing stress may reduce the frequency and severity of IBS symptoms. Techniques that may be helpful include regular exercise, such as swimming, walking, or running.[150]

Vagus nerve stimulation

Vagus nerve stimulation has anti-inflammatory effects and its potential for the treatment of IBS is actively researched.[151][152][153]

Alternative medicine

A meta-analysis found no benefits of acupuncture relative to placebo for IBS symptom severity or IBS-related quality of life.[154]

Probiotics

Probiotics can be beneficial in the treatment of IBS; taking 10 billion to 100 billion beneficial bacteria per day is recommended for beneficial results. However, further research is needed on individual strains of beneficial bacteria for more refined recommendations.[149][155] Probiotics have positive effects such as enhancing the intestinal mucosal barrier, providing a physical barrier, bacteriocin production (resulting in reduced numbers of pathogenic and gas-producing bacteria), reducing intestinal permeability and bacterial translocation, and regulating the immune system both locally and systemically among other beneficial effects.[82] Probiotics may also have positive effects on the gut–brain axis by their positive effects countering the effects of stress on gut immunity and gut function.[156]

A number of probiotics have been found to be effective, including *Lactobacillus plantarum*,[82] and *Bifidobacteria infantis*;[157] but one review found only *Bifidobacteria infantis* showed efficacy.[158] *B. infantis* may have effects beyond the gut via it causing a reduction of proinflammatory cytokine activity and elevation of blood tryptophan levels, which may cause an

improvement in symptoms of depression.[159] Some yogurt is made using probiotics that may help ease symptoms of IBS.[160] A probiotic yeast called *Saccharomyces boulardii* has some evidence of effectiveness in the treatment of irritable bowel syndrome.[161]

Certain probiotics have different effects on certain symptoms of IBS. For example, *Bifidobacterium breve*, *B. longum,* and *Lactobacillus acidophilus* have been found to alleviate abdominal pain. *B. breve, B. infantis, L. casei*, or *L. plantarum* species alleviated distension symptoms. *B. breve, B. infantis, L. casei, L. plantarum, B. longum, L. acidophilus, L. bulgaricus*, and *Streptococcus salivarius* ssp. *thermophilus* have all been found to affect flatulence levels. Most clinical studies show probiotics do not improve straining, sense of incomplete evacuation, stool consistency, fecal urgency, or stool frequency, although a few clinical studies did find some benefit of probiotic therapy. The evidence is conflicting for whether probiotics improve overall quality of life scores.[162]

Probiotics may exert their beneficial effects on IBS symptoms via preserving the gut microbiota, normalisation of cytokine blood levels, improving the intestinal transit time, decreasing small intestine permeability, and by treating small intestinal bacterial overgrowth of fermenting bacteria.[162] A fecal transplant does not appear useful as of 2019.[163]

The most clinically researched multi-strain probiotic is the De Simone Formulation. Developed by a gastroenterologist medical doctor, the formulation has been studied in over 70 human clinical trials since the 1990s including for dysbiosis factors relating to irritable bowel syndrome in children and adults.[164][165]

Herbal remedies

Peppermint oil appears useful.[166] In a meta-analysis it was found to be superior to placebo for improvement of IBS symptoms, at least in the short term.[105] An earlier meta-analysis suggested the results of peppermint oil were tentative as the number of people studied was small and blinding of those receiving treatment was unclear.[103] Safety during pregnancy has not been established, however, and caution is required not to chew or break the enteric coating; otherwise, gastroesophageal reflux may occur as a result of lower esophageal sphincter relaxation. Occasionally, nausea and perianal burning occur as side effects.[123] Iberogast, a multi-herbal extract, was found to be superior in efficacy to placebo.[167] A comprehensive meta-analysis using twelve random trials resulted that the use of peppermint oil is an effective therapy for adults with irritable bowel syndrome.[168]

Research into cannabinoids as treatment for IBS is limited. GI propulsion, secretion, and inflammation in the gut are all modulated by the ECS (Endocannabinoid system), providing a rationale for cannabinoids as treatment candidates for IBS.[169]

Only limited evidence exists for the effectiveness of other herbal remedies for IBS. As with all herbs, it is wise to be aware of possible drug interactions and adverse effects.[123]

Epidemiology

The prevalence of IBS varies by country and by age range examined. The bar graph at right shows the percentage of the population reporting symptoms of IBS in studies from various geographic regions (see table below for references). The following table contains a list of studies performed in different countries that measured the prevalence of IBS and IBS-like symptoms:

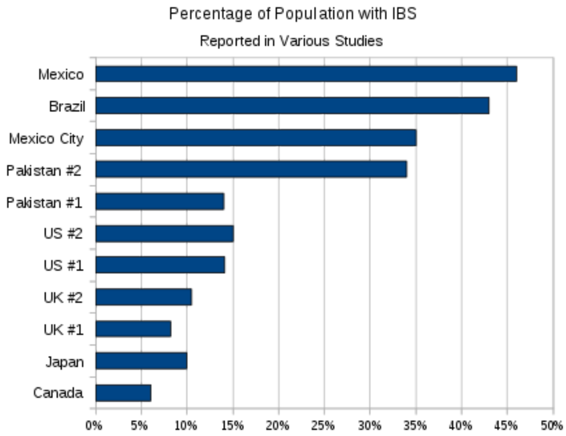

Percentage of population with IBS reported in various studies in different countries (see sources in the table)

Percentage of population reporting symptoms of IBS in various studies from various geographic areas			
**Location**	**Prevalence**	**Author/year**	**Notes**
Canada	6%[170]	Boivin, 2001	
Japan	10%[171]	Quigley, 2006	Study measured prevalence of GI abdominal pain/cramping
United Kingdom	8.2%[172]<br>10.5%[173]	Ehlin, 2003<br>Wilson, 2004	Prevalence increased substantially 1970–2004
United States	14.1%[174]	Hungin, 2005	Most undiagnosed
United States	15%[170]	Boivin, 2001	Estimate
Pakistan	14%[175]	Jafri, 2007	Much more common in 16–30 age range. 56% male, 44% female
Pakistan	34%[176]	Jafri, 2005	College students
Mexico City	35%[177]	Schmulson, 2006	n=324. Also measured functional diarrhea and functional vomiting. High rates attributed to "stress of living in a populated city."
Brazil	43%[171]	Quigley, 2006	Study measured prevalence of GI abdominal pain/cramping
Mexico	46%[171]	Quigley, 2006	Study measured prevalence of GI abdominal pain/cramping

Gender

In western countries, women are around two to three times more likely to be diagnosed with IBS and four to five times more likely to seek specialty care for it than men.[178] However, women in East Asian countries are not more likely than men to have irritable bowel syndrome, and there are

conflicting reports about the female predominance of the disease in Africa and other parts of Asia.[179] People diagnosed with IBS are usually younger than 45 years old.[1] Studies of females with IBS show symptom severity often fluctuates with the menstrual cycle, suggesting hormonal differences may play a role.[180] Endorsement of gender-related traits has been associated with quality of life and psychological adjustment in IBS.[181] The increase in gastrointestinal symptoms during menses or early menopause may be related to declining or low estrogen and progesterone, suggesting that estrogen withdrawal may play a role in IBS.[182] Gender differences in healthcare-seeking may also play a role.[183] Gender differences in trait anxiety may contribute to lower pain thresholds in women, putting them at greater risk for a number of chronic pain disorders.[184] Finally, sexual trauma is a major risk factor for IBS, as are other forms of abuse.[185] Because women are at higher risk of sexual abuse than men, sex-related risk of abuse may contribute to the higher rate of IBS in women.[186]

History

The concept of an "irritable bowel" was introduced by P.W. Brown, first in The Journal Of The Kansas Medical Society in 1947[187] and later in the *Rocky Mountain Medical Journal* in 1950.[188] The term was used to categorize people who developed symptoms of diarrhea, abdominal pain, and constipation, but where no well-recognized infective cause could be found. Early theories suggested the irritable bowel was caused by a psychosomatic or mental disorder.[189]

Society and culture

Economics

United States

The aggregate cost of irritable bowel syndrome in the United States has been estimated at $1.7–10 billion in direct medical costs, with an additional $20 billion in indirect costs, for a total of $21.7–30 billion.[9] A study by a managed care company comparing medical costs for people with IBS to non-IBS controls identified a 49% annual increase in medical costs associated with a diagnosis of IBS.[190] People with IBS incurred average annual direct costs of $5,049 and $406 in out-of-pocket expenses in 2007.[191] A study of workers with IBS found that they reported a 34.6% loss in productivity, corresponding to 13.8 hours lost per 40 hour week.[192] A study of employer-related health costs from a Fortune 100 company conducted with data from the 1990s found people with IBS incurred US$4527 in claims costs vs. $3276 for controls.[193] A study on Medicaid costs conducted in 2003 by the University of Georgia College of Pharmacy and Novartis found IBS was associated in an increase of $962 in Medicaid costs in California, and $2191 in North Carolina. People with IBS had higher costs for physician visits, outpatients visits, and prescription drugs. The study suggested the costs associated with IBS were comparable to those found for people with asthma.[194]

Research

Individuals with IBS have been found to have decreased diversity and numbers of Bacteroidota microbiota. Preliminary research into the effectiveness of fecal microbiota transplant in the treatment of IBS has been very favourable with a 'cure' rate of between 36 percent and 60 percent with remission of core IBS symptoms persisting at 9 and 19 months follow up.[195][196] Treatment

with probiotic strains of bacteria has shown to be effective, though not all strains of microorganisms confer the same benefit and adverse side effects have been documented in a minority of cases.[197]

There is increasing evidence for the effectiveness of mesalazine (5-aminosalicylic acid) in the treatment of IBS.[198] Mesalazine is a drug with anti-inflammatory properties that has been reported to significantly reduce immune mediated inflammation in the gut of IBS affected individuals with mesalazine therapy resulting in improved IBS symptoms as well as feelings of general wellness in IBS affected people. It has also been observed that mesalazine therapy helps to normalise the gut flora which is often abnormal in people who have IBS. The therapeutic benefits of mesalazine may be the result of improvements to the epithelial barrier function.[199] Treatment based on "abnormally" high IgG antibodies cannot be recommended.[200]

Differences in visceral sensitivity and intestinal physiology have been noted in IBS. Mucosal barrier reinforcement in response to oral 5-HTP was absent in IBS compared to controls.[201] IBS/IBD individuals are less often HLA DQ2/8 positive than in upper functional gastrointestinal disease and healthy populations.[202]

In other species

A similar syndrome is found in rats (*Rattus* spp.).[203] In rats a short-chain fatty acid receptor is involved.[203] Karaki *et al.*, 2006 finds a free fatty acid receptor 2 subtype – GPR43 – that is expressed in both enteroendocrine cells and mucosal mast cells.[203] These cells then respond in an exaggerated way to the IBS rat's own large quantity of maldigestion products.[203]

Notes

1. The cited review is based on sources ranging from 1988 to 2001 and is probably biased relative to a more recent research.

References

1. "Definition and Facts for Irritable Bowel Syndrome" (https://web.archive.org/web/20160402144132/http://www.niddk.nih.gov/health-information/health-topics/digestive-diseases/irritable-bowel-syndrome/Pages/definition-facts.aspx). *NIDDKD*. February 23, 2015. Archived from the original (http://www.niddk.nih.gov/health-information/health-topics/digestive-diseases/irritable-bowel-syndrome/Pages/definition-facts.aspx) on April 2, 2016. Retrieved March 29, 2016.
2. "Symptoms and Causes of Irritable Bowel Syndrome" (https://web.archive.org/web/20160405092200/http://www.niddk.nih.gov/health-information/health-topics/digestive-diseases/irritable-bowel-syndrome/Pages/symptoms-causes.aspx). *NIDDK*. February 23, 2015. Archived from the original (http://www.niddk.nih.gov/health-information/health-topics/digestive-diseases/irritable-bowel-syndrome/Pages/symptoms-causes.aspx) on April 5, 2016. Retrieved March 29, 2016.
3. Chey WD, Kurlander J, Eswaran S (March 2015). "Irritable bowel syndrome: a clinical review". *JAMA*. **313** (9): 949–58. doi:10.1001/jama.2015.0954 (https://doi.org/10.1001%2Fjama.2015.0954). PMID 25734736 (https://pubmed.ncbi.nlm.nih.gov/25734736). S2CID 205062386 (https://api.semanticscholar.org/CorpusID:205062386).
4. Levy J, Bernstein L, Silber N (December 2014). "Celiac disease: an immune dysregulation syndrome". *Current Problems in Pediatric and Adolescent Health Care*. **44** (11): 324–7. doi:10.1016/j.cppeds.2014.10.002 (https://doi.org/10.1016%2Fj.cppeds.2014.10.002). PMID 25499458 (https://pubmed.ncbi.nlm.nih.gov/25499458).

5. "Treatment for Irritable Bowel Syndrome" (https://web.archive.org/web/20160406205101/http://www.niddk.nih.gov/health-information/health-topics/digestive-diseases/irritable-bowel-syndrome/Pages/treatment.aspx). *NIDDK*. February 23, 2015. Archived from the original (http://www.niddk.nih.gov/health-information/health-topics/digestive-diseases/irritable-bowel-syndrome/Pages/treatment.aspx) on April 6, 2016. Retrieved March 29, 2016.
6. Quigley EM (2013). "Treatment level 1" (https://books.google.com/books?id=qSAruYVwLLcC&pg=PT295). *Irritable Bowel Syndrome: Diagnosis and Clinical Management* (First ed.). Chichester, West Sussex: Wiley-Blackwell. ISBN 9781118444740. Archived (https://web.archive.org/web/20170908144619/https://books.google.com/books?id=qSAruYVwLLcC&pg=PT295) from the original on September 8, 2017.
7. Maxion-Bergemann S, Thielecke F, Abel F, Bergemann R (2006). "Costs of irritable bowel syndrome in the UK and US". *PharmacoEconomics*. **24** (1): 21–37. doi:10.2165/00019053-200624010-00002 (https://doi.org/10.2165%2F00019053-200624010-00002). PMID 16445300 (https://pubmed.ncbi.nlm.nih.gov/16445300). S2CID 45376327 (https://api.semanticscholar.org/CorpusID:45376327).
8. Lovell RM, Ford AC (July 2012). "Global prevalence of and risk factors for irritable bowel syndrome: a meta-analysis". *Clinical Gastroenterology and Hepatology*. **10** (7): 712–721.e4. doi:10.1016/j.cgh.2012.02.029 (https://doi.org/10.1016%2Fj.cgh.2012.02.029). PMID 22426087 (https://pubmed.ncbi.nlm.nih.gov/22426087). "Pooled prevalence in all studies was 11.2% (95% CI, 9.8%-12.8%). The prevalence varied according to country (from 1.1% to 45.0%) and criteria used to define IBS... Women are at slightly higher risk for IBS than men."
9. Hulisz D (2004). "The burden of illness of irritable bowel syndrome: current challenges and hope for the future". *Journal of Managed Care Pharmacy*. **10** (4): 299–309. doi:10.18553/jmcp.2004.10.4.299 (https://doi.org/10.18553%2Fjmcp.2004.10.4.299). PMID 15298528 (https://pubmed.ncbi.nlm.nih.gov/15298528). S2CID 9413379 (https://api.semanticscholar.org/CorpusID:9413379).
10. Whitehead WE, Palsson O, Jones KR (April 2002). "Systematic review of the comorbidity of irritable bowel syndrome with other disorders: what are the causes and implications?" (https://cdr.lib.unc.edu/downloads/zs25xj11x). *Gastroenterology*. **122** (4): 1140–56. doi:10.1053/gast.2002.32392 (https://doi.org/10.1053%2Fgast.2002.32392). PMID 11910364 (https://pubmed.ncbi.nlm.nih.gov/11910364).
11. Spiller R, Garsed K (May 2009). "Postinfectious irritable bowel syndrome". *Gastroenterology*. **136** (6): 1979–88. doi:10.1053/j.gastro.2009.02.074 (https://doi.org/10.1053%2Fj.gastro.2009.02.074). PMID 19457422 (https://pubmed.ncbi.nlm.nih.gov/19457422).
12. Chang L (March 2011). "The role of stress on physiologic responses and clinical symptoms in irritable bowel syndrome" (https://www.ncbi.nlm.nih.gov/pmc/articles/PMC3039211). *Gastroenterology*. **140** (3): 761–5. doi:10.1053/j.gastro.2011.01.032 (https://doi.org/10.1053%2Fj.gastro.2011.01.032). PMC 3039211 (https://www.ncbi.nlm.nih.gov/pmc/articles/PMC3039211). PMID 21256129 (https://pubmed.ncbi.nlm.nih.gov/21256129).
13. Palsson, Olafur S.; Peery, Anne; Seitzberg, Dorthe; Amundsen, Ingvild Dybdrodt; McConnell, Bruce; Simrén, Magnus (December 7, 2020). "Human Milk Oligosaccharides Support Normal Bowel Function and Improve Symptoms of Irritable Bowel Syndrome: A Multicenter, Open-Label Trial" (https://www.ncbi.nlm.nih.gov/pmc/articles/PMC7721220). *Clinical and Translational Gastroenterology*. **11** (12): e00276. doi:10.14309/ctg.0000000000000276 (https://doi.org/10.14309%2Fctg.0000000000000276). ISSN 2155-384X (https://www.worldcat.org/issn/2155-384X). PMC 7721220 (https://www.ncbi.nlm.nih.gov/pmc/articles/PMC7721220). PMID 33512807 (https://pubmed.ncbi.nlm.nih.gov/33512807).

14. Moayyedi P, Quigley EM, Lacy BE, Lembo AJ, Saito YA, Schiller LR, Soffer EE, Spiegel BM, Ford AC (September 2014). "The effect of fiber supplementation on irritable bowel syndrome: a systematic review and meta-analysis". *The American Journal of Gastroenterology*. **109** (9): 1367–74. doi:10.1038/ajg.2014.195 (https://doi.org/10.1038%2Fajg.2014.195). PMID 25070054 (https://pubmed.ncbi.nlm.nih.gov/25070054). S2CID 8076372 (https://api.semanticscholar.org/CorpusID:8076372).
15. Rao SS, Yu S, Fedewa A (June 2015). "Systematic review: dietary fibre and FODMAP-restricted diet in the management of constipation and irritable bowel syndrome". *Alimentary Pharmacology & Therapeutics*. **41** (12): 1256–70. doi:10.1111/apt.13167 (https://doi.org/10.1111%2Fapt.13167). PMID 25903636 (https://pubmed.ncbi.nlm.nih.gov/25903636). S2CID 27558785 (https://api.semanticscholar.org/CorpusID:27558785).
16. Mayer EA (April 2008). "Clinical practice. Irritable bowel syndrome" (https://www.ncbi.nlm.nih.gov/pmc/articles/PMC3816529). *The New England Journal of Medicine*. **358** (16): 1692–9. doi:10.1056/NEJMcp0801447 (https://doi.org/10.1056%2FNEJMcp0801447). PMC 3816529 (https://www.ncbi.nlm.nih.gov/pmc/articles/PMC3816529). PMID 18420501 (https://pubmed.ncbi.nlm.nih.gov/18420501).
17. Jung, Hye-Kyung (April 2011). "Is There True Gender Difference of Irritable Bowel Syndrome in Asia?" (https://www.ncbi.nlm.nih.gov/pmc/articles/PMC3093021). *Journal of Neurogastroenterology and Motility*. **17** (2): 206–207. doi:10.5056/jnm.2011.17.2.206 (https://doi.org/10.5056%2Fjnm.2011.17.2.206). PMC 3093021 (https://www.ncbi.nlm.nih.gov/pmc/articles/PMC3093021). PMID 21603006 (https://pubmed.ncbi.nlm.nih.gov/21603006). "However, some Asian studies fail to report significant gender differences in the prevalence of IBS.6"
18. Hatch MC (2000). *Women and Health* (https://books.google.com/books?id=zSCgj8LXFHAC&pg=PA1098). San Diego, Calif: Academic Press. p. 1098. ISBN 9780122881459. Archived (https://web.archive.org/web/20170908144619/https://books.google.com/books?id=zSCgj8LXFHAC&pg=PA1098) from the original on September 8, 2017.
19. Holten KB, Wetherington A, Bankston L (May 2003). "Diagnosing the patient with abdominal pain and altered bowel habits: is it irritable bowel syndrome?" (http://www.aafp.org/afp/20030515/2157.html). *American Family Physician*. **67** (10): 2157–2162. PMID 12776965 (https://pubmed.ncbi.nlm.nih.gov/12776965). Archived (https://web.archive.org/web/20080515204942/http://www.aafp.org/afp/20030515/2157.html) from the original on May 15, 2008.
20. DuPont AW (February 2008). "Postinfectious irritable bowel syndrome". *Clinical Infectious Diseases*. Oxford University Press. **46** (4): 594–599. doi:10.1086/526774 (https://doi.org/10.1086%2F526774). PMID 18205536 (https://pubmed.ncbi.nlm.nih.gov/18205536).
21. Spiller R, Lam C (July 2012). "An Update on Post-infectious Irritable Bowel Syndrome: Role of Genetics, Immune Activation, Serotonin and Altered Microbiome" (https://www.ncbi.nlm.nih.gov/pmc/articles/PMC3400813). *Journal of Neurogastroenterology and Motility*. **18** (3): 258–268. doi:10.5056/jnm.2012.18.3.258 (https://doi.org/10.5056%2Fjnm.2012.18.3.258). PMC 3400813 (https://www.ncbi.nlm.nih.gov/pmc/articles/PMC3400813). PMID 22837873 (https://pubmed.ncbi.nlm.nih.gov/22837873).
22. Spiller RC (May 2003). "Postinfectious irritable bowel syndrome". *Gastroenterology*. **124** (6): 1662–1671. doi:10.1016/S0016-5085(03)00324-X (https://doi.org/10.1016%2FS0016-5085%2803%2900324-X). PMID 12761724 (https://pubmed.ncbi.nlm.nih.gov/12761724).
23. Iacob T, Ţăţulescu DF, Dumitraşcu DL (2017). "Therapy of the postinfectious irritable bowel syndrome: an update" (https://www.ncbi.nlm.nih.gov/pmc/articles/PMC5433563). *Clujul Medical*. **90** (2): 133–138. doi:10.15386/cjmed-752 (https://doi.org/10.15386%2Fcjmed-752). PMC 5433563 (https://www.ncbi.nlm.nih.gov/pmc/articles/PMC5433563). PMID 28559695 (https://pubmed.ncbi.nlm.nih.gov/28559695).

24. Schmulson MW, Chang L (November 1999). "Diagnostic approach to the patient with irritable bowel syndrome". *The American Journal of Medicine*. **107** (5A): 20S–26S. doi:10.1016/S0002-9343(99)00278-8 (https://doi.org/10.1016%2FS0002-9343%2899%2900278-8). PMID 10588169 (https://pubmed.ncbi.nlm.nih.gov/10588169).
25. Tamparo C (2011). *Fifth Edition: Diseases of the Human Body*. Philadelphia, PA: F.A. Davis Company. p. 407. ISBN 978-0-8036-2505-1.
26. Talley NJ (November 2006). "Irritable bowel syndrome" (https://www.ncbi.nlm.nih.gov/pmc/articles/PMC1761148). *Internal Medicine Journal*. **36** (11): 724–8. doi:10.1111/j.1445-5994.2006.01217.x (https://doi.org/10.1111%2Fj.1445-5994.2006.01217.x). PMC 1761148 (https://www.ncbi.nlm.nih.gov/pmc/articles/PMC1761148). PMID 17040359 (https://pubmed.ncbi.nlm.nih.gov/17040359).
27. Sperber AD, Dekel R (April 2010). "Irritable Bowel Syndrome and Co-morbid Gastrointestinal and Extra-gastrointestinal Functional Syndromes" (https://www.ncbi.nlm.nih.gov/pmc/articles/PMC2879857). *Journal of Neurogastroenterology and Motility*. **16** (2): 113–9. doi:10.5056/jnm.2010.16.2.113 (https://doi.org/10.5056%2Fjnm.2010.16.2.113). PMC 2879857 (https://www.ncbi.nlm.nih.gov/pmc/articles/PMC2879857). PMID 20535341 (https://pubmed.ncbi.nlm.nih.gov/20535341).
28. Wouters MM, Vicario M, Santos J (January 2016). "The role of mast cells in functional GI disorders" (https://doi.org/10.1136%2Fgutjnl-2015-309151). *Gut*. **65** (1): 155–68. doi:10.1136/gutjnl-2015-309151 (https://doi.org/10.1136%2Fgutjnl-2015-309151). PMID 26194403 (https://pubmed.ncbi.nlm.nih.gov/26194403). S2CID 3456082 (https://api.semanticscholar.org/CorpusID:3456082).
29. Ohman L, Simrén M (March 2010). "Pathogenesis of IBS: role of inflammation, immunity and neuroimmune interactions". *Nature Reviews. Gastroenterology & Hepatology*. **7** (3): 163–173. doi:10.1038/nrgastro.2010.4 (https://doi.org/10.1038%2Fnrgastro.2010.4). PMID 20101257 (https://pubmed.ncbi.nlm.nih.gov/20101257). S2CID 20898797 (https://api.semanticscholar.org/CorpusID:20898797).
30. Rothenberg ME (June 2021). "An Allergic Basis for Abdominal Pain". *The New England Journal of Medicine*. **384** (22): 2156–2158. doi:10.1056/NEJMcibr2104146 (https://doi.org/10.1056%2FNEJMcibr2104146). PMID 34077648 (https://pubmed.ncbi.nlm.nih.gov/34077648). S2CID 235322218 (https://api.semanticscholar.org/CorpusID:235322218).
31. Kim JH, Yi DY, Lee YM, Choi YJ, Kim JY, Hong YH, Park JY, Kim SY, Lee NM, Yun SW, Chae SA, Lim IS, Choi ES, Jeong IS (August 2022). "Association between body mass index and fecal calprotectin levels in children and adolescents with irritable bowel syndrome" (https://www.ncbi.nlm.nih.gov/pmc/articles/PMC9371505). *Medicine*. **101** (32): e29968. doi:10.1097/MD.0000000000029968 (https://doi.org/10.1097%2FMD.0000000000029968). PMC 9371505 (https://www.ncbi.nlm.nih.gov/pmc/articles/PMC9371505). PMID 35960084 (https://pubmed.ncbi.nlm.nih.gov/35960084).
32. Enck P, Aziz Q, Barbara G, Farmer AD, Fukudo S, Mayer EA, et al. (March 2016). "Irritable bowel syndrome" (https://www.ncbi.nlm.nih.gov/pmc/articles/PMC5001845). *Nature Reviews. Disease Primers*. **2**: 16014. doi:10.1038/nrdp.2016.14 (https://doi.org/10.1038%2Fnrdp.2016.14). PMC 5001845 (https://www.ncbi.nlm.nih.gov/pmc/articles/PMC5001845). PMID 27159638 (https://pubmed.ncbi.nlm.nih.gov/27159638).
33. Thabane M, Kottachchi DT, Marshall JK (August 2007). "Systematic review and meta-analysis: The incidence and prognosis of post-infectious irritable bowel syndrome" (https://archive.today/20130713045629/http://www3.interscience.wiley.com/cgi-bin/fulltext/117987841/HTMLSTART). *Alimentary Pharmacology & Therapeutics*. **26** (4): 535–544. doi:10.1111/j.1365-2036.2007.03399.x (https://doi.org/10.1111%2Fj.1365-2036.2007.03399.x). PMID 17661757 (https://pubmed.ncbi.nlm.nih.gov/17661757). S2CID 41136248 (https://api.semanticscholar.org/CorpusID:41136248). Archived from the original (http://www3.interscience.wiley.com/cgi-bin/fulltext/117987841/HTMLSTART) on July 13, 2013.

34. "World Gastroenterology Organisation Global Guidelines. Irritable Bowel Syndrome: a Global Perspective" (http://www.worldgastroenterology.org/UserFiles/file/guidelines/irritable-bowel-syndrome-english-2015.pdf) (PDF). World Gastroenterology Organisation. September 2015. Archived (https://web.archive.org/web/20160527194952/http://www.worldgastroenterology.org/UserFiles/file/guidelines/irritable-bowel-syndrome-english-2015.pdf) (PDF) from the original on May 27, 2016. Retrieved April 24, 2016.
35. Shanahan F, Quigley EM (May 2014). "Manipulation of the microbiota for treatment of IBS and IBD-challenges and controversies". *Gastroenterology*. **146** (6): 1554–1563. doi:10.1053/j.gastro.2014.01.050 (https://doi.org/10.1053%2Fj.gastro.2014.01.050). PMID 24486051 (https://pubmed.ncbi.nlm.nih.gov/24486051).
36. Beatty JK, Bhargava A, Buret AG (April 2014). "Post-infectious irritable bowel syndrome: mechanistic insights into chronic disturbances following enteric infection" (https://www.ncbi.nlm.nih.gov/pmc/articles/PMC3983453). *World Journal of Gastroenterology*. **20** (14): 3976–85. doi:10.3748/wjg.v20.i14.3976 (https://doi.org/10.3748%2Fwjg.v20.i14.3976). PMC 3983453 (https://www.ncbi.nlm.nih.gov/pmc/articles/PMC3983453). PMID 24744587 (https://pubmed.ncbi.nlm.nih.gov/24744587).
37. Fukudo S, Nomura T, Muranaka M, Taguchi F (September 1993). "Brain-gut response to stress and cholinergic stimulation in irritable bowel syndrome. A preliminary study". *Journal of Clinical Gastroenterology*. **17** (2): 133–41. doi:10.1097/00004836-199309000-00009 (https://doi.org/10.1097%2F00004836-199309000-00009). PMID 8031340 (https://pubmed.ncbi.nlm.nih.gov/8031340).
38. Barreau F, Ferrier L, Fioramonti J, Bueno L (September 2007). "New insights in the etiology and pathophysiology of irritable bowel syndrome: contribution of neonatal stress models" (https://doi.org/10.1203%2FPDR.0b013e3180db2949). *Pediatric Research*. **62** (3): 240–5. doi:10.1203/PDR.0b013e3180db2949 (https://doi.org/10.1203%2FPDR.0b013e3180db2949). PMID 17622962 (https://pubmed.ncbi.nlm.nih.gov/17622962). S2CID 26538682 (https://api.semanticscholar.org/CorpusID:26538682).
39. Li J, Zhu W, Liu W, Wu Y, Wu B (January 2016). "Rifaximin for Irritable Bowel Syndrome: A Meta-Analysis of Randomized Placebo-Controlled Trials" (https://www.ncbi.nlm.nih.gov/pmc/articles/PMC5291563). *Medicine*. **95** (4): e2534. doi:10.1097/MD.0000000000002534 (https://doi.org/10.1097%2FMD.0000000000002534). PMC 5291563 (https://www.ncbi.nlm.nih.gov/pmc/articles/PMC5291563). PMID 26825893 (https://pubmed.ncbi.nlm.nih.gov/26825893).
40. Spiller R, Aziz Q, Creed F, Emmanuel A, Houghton L, Hungin P, Jones R, Kumar D, Rubin G, Trudgill N, Whorwell P (December 2007). "Guidelines on the irritable bowel syndrome: mechanisms and practical management" (https://www.ncbi.nlm.nih.gov/pmc/articles/PMC2095723). *Gut*. **56** (12): 1770–98. doi:10.1136/gut.2007.119446 (https://doi.org/10.1136%2Fgut.2007.119446). PMC 2095723 (https://www.ncbi.nlm.nih.gov/pmc/articles/PMC2095723). PMID 17488783 (https://pubmed.ncbi.nlm.nih.gov/17488783).
41. Fukudo S (January 2007). "Role of corticotropin-releasing hormone in irritable bowel syndrome and intestinal inflammation". *Journal of Gastroenterology*. **42** (Suppl 17): 48–51. doi:10.1007/s00535-006-1942-7 (https://doi.org/10.1007%2Fs00535-006-1942-7). PMID 17238026 (https://pubmed.ncbi.nlm.nih.gov/17238026). S2CID 26067985 (https://api.semanticscholar.org/CorpusID:26067985).
42. "Post-infectious IBS" (https://aboutibs.org/what-is-ibs-sidenav/post-infectious-ibs.html). *aboutibs.org*. March 8, 2021. Retrieved April 2, 2021.
43. Barbara G, Grover M, Bercik P, Corsetti M, Ghoshal UC, Ohman L, Rajilić-Stojanović M (January 2019). "Rome Foundation Working Team Report on Post-Infection Irritable Bowel Syndrome" (https://www.gastrojournal.org/article/S0016-5085(18)34766-8/abstract). *Gastroenterology*. **156** (1): 46–58.e7. doi:10.1053/j.gastro.2018.07.011 (https://doi.org/10.1053%2Fj.gastro.2018.07.011). PMC 6309514 (https://www.ncbi.nlm.nih.gov/pmc/articles/PMC6309514). PMID 30009817 (https://pubmed.ncbi.nlm.nih.gov/30009817).

44. Ghoshal UC, Gwee KA (July 2017). "Post-infectious IBS, tropical sprue and small intestinal bacterial overgrowth: the missing link". *Nature Reviews. Gastroenterology & Hepatology*. **14** (7): 435–441. doi:10.1038/nrgastro.2017.37 (https://doi.org/10.1038%2Fnrgastro.2017.37). PMID 28513629 (https://pubmed.ncbi.nlm.nih.gov/28513629). S2CID 33660302 (https://api.semanticscholar.org/CorpusID:33660302).
45. Chen B, Kim JJ, Zhang Y, Du L, Dai N (July 2018). "Prevalence and predictors of small intestinal bacterial overgrowth in irritable bowel syndrome: a systematic review and meta-analysis". *Journal of Gastroenterology*. **53** (7): 807–818. doi:10.1007/s00535-018-1476-9 (https://doi.org/10.1007%2Fs00535-018-1476-9). PMID 29761234 (https://pubmed.ncbi.nlm.nih.gov/29761234). S2CID 46889298 (https://api.semanticscholar.org/CorpusID:46889298).
46. Ghoshal UC, Srivastava D (March 2014). "Irritable bowel syndrome and small intestinal bacterial overgrowth: meaningful association or unnecessary hype" (https://www.ncbi.nlm.nih.gov/pmc/articles/PMC3949258). *World Journal of Gastroenterology*. **20** (10): 2482–91. doi:10.3748/wjg.v20.i10.2482 (https://doi.org/10.3748%2Fwjg.v20.i10.2482). PMC 3949258 (https://www.ncbi.nlm.nih.gov/pmc/articles/PMC3949258). PMID 24627585 (https://pubmed.ncbi.nlm.nih.gov/24627585).
47. Bennet SM, Ohman L, Simren M (May 2015). "Gut microbiota as potential orchestrators of irritable bowel syndrome" (https://www.ncbi.nlm.nih.gov/pmc/articles/PMC4413965). *Gut and Liver*. **9** (3): 318–31. doi:10.5009/gnl14344 (https://doi.org/10.5009%2Fgnl14344). PMC 4413965 (https://www.ncbi.nlm.nih.gov/pmc/articles/PMC4413965). PMID 25918261 (https://pubmed.ncbi.nlm.nih.gov/25918261).
48. Collins SM (August 2014). "A role for the gut microbiota in IBS". *Nature Reviews. Gastroenterology & Hepatology*. **11** (8): 497–505. doi:10.1038/nrgastro.2014.40 (https://doi.org/10.1038%2Fnrgastro.2014.40). PMID 24751910 (https://pubmed.ncbi.nlm.nih.gov/24751910). S2CID 10676400 (https://api.semanticscholar.org/CorpusID:10676400).
49. Santelmann H, Howard JM (January 2005). "Yeast metabolic products, yeast antigens and yeasts as possible triggers for irritable bowel syndrome". *European Journal of Gastroenterology & Hepatology*. **17** (1): 21–6. CiteSeerX 10.1.1.567.6030 (https://citeseerx.ist.psu.edu/viewdoc/summary?doi=10.1.1.567.6030). doi:10.1097/00042737-200501000-00005 (https://doi.org/10.1097%2F00042737-200501000-00005). PMID 15647635 (https://pubmed.ncbi.nlm.nih.gov/15647635). S2CID 35882838 (https://api.semanticscholar.org/CorpusID:35882838).
50. Lagacé-Wiens PR, VanCaeseele PG, Koschik C (August 2006). "Dientamoeba fragilis: an emerging role in intestinal disease" (https://www.ncbi.nlm.nih.gov/pmc/articles/PMC1550747). *CMAJ*. **175** (5): 468–9. doi:10.1503/cmaj.060265 (https://doi.org/10.1503%2Fcmaj.060265). PMC 1550747 (https://www.ncbi.nlm.nih.gov/pmc/articles/PMC1550747). PMID 16940260 (https://pubmed.ncbi.nlm.nih.gov/16940260).
51. Amin OM (June 2002). "Seasonal prevalence of intestinal parasites in the United States during 2000" (https://doi.org/10.4269%2Fajtmh.2002.66.799). *The American Journal of Tropical Medicine and Hygiene*. **66** (6): 799–803. doi:10.4269/ajtmh.2002.66.799 (https://doi.org/10.4269%2Fajtmh.2002.66.799). PMID 12224595 (https://pubmed.ncbi.nlm.nih.gov/12224595).
52. Stark D, van Hal S, Marriott D, Ellis J, Harkness J (January 2007). "Irritable bowel syndrome: a review on the role of intestinal protozoa and the importance of their detection and diagnosis". *International Journal for Parasitology*. **37** (1): 11–20. doi:10.1016/j.ijpara.2006.09.009 (https://doi.org/10.1016%2Fj.ijpara.2006.09.009). PMID 17070814 (https://pubmed.ncbi.nlm.nih.gov/17070814).

53. Wawrzyniak I, Poirier P, Viscogliosi E, Dionigia M, Texier C, Delbac F, Alaoui HE (October 2013). "Blastocystis, an unrecognized parasite: an overview of pathogenesis and diagnosis" (https://www.ncbi.nlm.nih.gov/pmc/articles/PMC4040727). *Therapeutic Advances in Infectious Disease*. **1** (5): 167–78. doi:10.1177/2049936113504754 (https://doi.org/10.1177%2F2049936113504754). PMC 4040727 (https://www.ncbi.nlm.nih.gov/pmc/articles/PMC4040727). PMID 25165551 (https://pubmed.ncbi.nlm.nih.gov/25165551). "Recent in vitro and in vivo studies have shed new light on the pathogenic power of this parasite, suggesting that Blastocystis sp. infection is associated with a variety of gastrointestinal disorders, may play a significant role in irritable bowel syndrome, and may be linked with cutaneous lesions (urticaria)."
54. Roberts T, Stark D, Harkness J, Ellis J (2014). "Update on the pathogenic potential and treatment options for Blastocystis sp" (https://www.ncbi.nlm.nih.gov/pmc/articles/PMC4039988). *Gut Pathogens*. **6**: 17. doi:10.1186/1757-4749-6-17 (https://doi.org/10.1186%2F1757-4749-6-17). PMC 4039988 (https://www.ncbi.nlm.nih.gov/pmc/articles/PMC4039988). PMID 24883113 (https://pubmed.ncbi.nlm.nih.gov/24883113).
55. Rostami A, Riahi SM, Haghighi A, Saber V, Armon B, Seyyedtabaei SJ (September 2017). "The role of Blastocystis sp. and Dientamoeba fragilis in irritable bowel syndrome: a systematic review and meta-analysis". *Parasitology Research*. **116** (9): 2361–2371. doi:10.1007/s00436-017-5535-6 (https://doi.org/10.1007%2Fs00436-017-5535-6). PMID 28668983 (https://pubmed.ncbi.nlm.nih.gov/28668983). S2CID 32999514 (https://api.semanticscholar.org/CorpusID:32999514).
56. Windsor JJ, Macfarlane L (May 2005). "Irritable bowel syndrome: the need to exclude Dientamoeba fragilis" (http://www.ajtmh.org/cgi/content/full/72/5/501). *The American Journal of Tropical Medicine and Hygiene*. **72** (5): 501, author reply 501–2. doi:10.4269/ajtmh.2005.72.5.0720501 (https://doi.org/10.4269%2Fajtmh.2005.72.5.0720501). PMID 15891119 (https://pubmed.ncbi.nlm.nih.gov/15891119). Archived (https://web.archive.org/web/20100717141228/http://www.ajtmh.org/cgi/content/full/72/5/501) from the original on July 17, 2010.
57. Williams CE, Williams EA, Corfe BM (October 2018). "Vitamin D status in irritable bowel syndrome and the impact of supplementation on symptoms: what do we know and what do we need to know?" (http://eprints.whiterose.ac.uk/127402/3/Williams%20et%20al%20EJCN%20Body%20Text%20R2%20Clean.pdf) (PDF). *European Journal of Clinical Nutrition*. **72** (10): 1358–1363. doi:10.1038/s41430-017-0064-z (https://doi.org/10.1038%2Fs41430-017-0064-z). PMID 29367731 (https://pubmed.ncbi.nlm.nih.gov/29367731). S2CID 19291568 (https://api.semanticscholar.org/CorpusID:19291568).
58. Ferguson LR, Laing B, Marlow G, Bishop K (January 2016). "The role of vitamin D in reducing gastrointestinal disease risk and assessment of individual dietary intake needs: Focus on genetic and genomic technologies". *Molecular Nutrition & Food Research*. **60** (1): 119–33. doi:10.1002/mnfr.201500243 (https://doi.org/10.1002%2Fmnfr.201500243). PMID 26251177 (https://pubmed.ncbi.nlm.nih.gov/26251177).
59. Barbalho SM, Goulart RA, Araújo AC, Guiguer ÉL, Bechara MD (April 2019). "Irritable bowel syndrome: a review of the general aspects and the potential role of vitamin D". *Expert Rev Gastroenterol Hepatol*. **13** (4): 345–359. doi:10.1080/17474124.2019.1570137 (https://doi.org/10.1080%2F17474124.2019.1570137). PMID 30791775 (https://pubmed.ncbi.nlm.nih.gov/30791775). S2CID 73484679 (https://api.semanticscholar.org/CorpusID:73484679).
60. Beyder A, Farrugia G (2016). "Ion channelopathies in functional GI disorders" (https://www.ncbi.nlm.nih.gov/pmc/articles/PMC5142191). *American Journal of Physiology. Gastrointestinal and Liver Physiology*. **311** (4): G581–G586. doi:10.1152/ajpgi.00237.2016 (https://doi.org/10.1152%2Fajpgi.00237.2016). PMC 5142191 (https://www.ncbi.nlm.nih.gov/pmc/articles/PMC5142191). PMID 27514480 (https://pubmed.ncbi.nlm.nih.gov/27514480).

61. Verstraelen TE, Ter Bekke RM, Volders PG, Masclee AA, Kruimel JW (2015). "The role of the SCN5A-encoded channelopathy in irritable bowel syndrome and other gastrointestinal disorders". *Neurogastroenterology & Motility*. **27** (7): 906–13. doi:10.1111/nmo.12569 (https://doi.org/10.1111%2Fnmo.12569). PMID 25898860 (https://pubmed.ncbi.nlm.nih.gov/25898860). S2CID 5055360 (https://api.semanticscholar.org/CorpusID:5055360).
62. Talley NJ (December 2006). "Genes and environment in irritable bowel syndrome: one step forward" (https://www.ncbi.nlm.nih.gov/pmc/articles/PMC1856457). *Gut*. **55** (12): 1694–6. doi:10.1136/gut.2006.108837 (https://doi.org/10.1136%2Fgut.2006.108837). PMC 1856457 (https://www.ncbi.nlm.nih.gov/pmc/articles/PMC1856457). PMID 17124153 (https://pubmed.ncbi.nlm.nih.gov/17124153).
63. Chang WY, Yang YT, She MP, Tu CH, Lee TC, Wu MS, Sun CH, Hsin LW, Yu LC (2022). "5-HT 7 receptor-dependent intestinal neurite outgrowth contributes to visceral hypersensitivity in irritable bowel syndrome" (https://www.ncbi.nlm.nih.gov/pmc/articles/PMC9420680). *Laboratory Investigation*. **102** (9): 1023–1037. doi:10.1038/s41374-022-00800-z (https://doi.org/10.1038%2Fs41374-022-00800-z). PMC 9420680 (https://www.ncbi.nlm.nih.gov/pmc/articles/PMC9420680). PMID 35585132 (https://pubmed.ncbi.nlm.nih.gov/35585132). S2CID 248867188 (https://api.semanticscholar.org/CorpusID:248867188).
64. Cremon C, Carini G, De Giorgio R, Stanghellini V, Corinaldesi R, Barbara G (May 2010). "Intestinal dysbiosis in irritable bowel syndrome: etiological factor or epiphenomenon?". *Expert Review of Molecular Diagnostics*. **10** (4): 389–93. doi:10.1586/erm.10.33 (https://doi.org/10.1586%2Ferm.10.33). PMID 20465494 (https://pubmed.ncbi.nlm.nih.gov/20465494). S2CID 207219334 (https://api.semanticscholar.org/CorpusID:207219334).
65. Schmulson M, Bielsa MV, Carmona-Sánchez R, Hernández A, López-Colombo A, López Vidal Y, Peláez-Luna M, Remes-Troche JM, Tamayo JL, Valdovinos MA (2014). "Microbiota, gastrointestinal infections, low-grade inflammation, and antibiotic therapy in irritable bowel syndrome: an evidence-based review" (https://doi.org/10.1016%2Fj.rgmx.2014.01.004). *Revista de Gastroenterologia de Mexico* (in Spanish). **79** (2): 96–134. doi:10.1016/j.rgmx.2014.01.004 (https://doi.org/10.1016%2Fj.rgmx.2014.01.004). PMID 24857420 (https://pubmed.ncbi.nlm.nih.gov/24857420).
66. Saito YA (March 2011). "The role of genetics in IBS" (https://www.ncbi.nlm.nih.gov/pmc/articles/PMC3056499). *Gastroenterology Clinics of North America*. **40** (1): 45–67. doi:10.1016/j.gtc.2010.12.011 (https://doi.org/10.1016%2Fj.gtc.2010.12.011). PMC 3056499 (https://www.ncbi.nlm.nih.gov/pmc/articles/PMC3056499). PMID 21333900 (https://pubmed.ncbi.nlm.nih.gov/21333900).
67. Yawn BP, Lydick E, Locke GR, Wollan PC, Bertram SL, Kurland MJ (2001). "Do published guidelines for evaluation of irritable bowel syndrome reflect practice?" (https://www.ncbi.nlm.nih.gov/pmc/articles/PMC59674). *BMC Gastroenterology*. **1**: 11. doi:10.1186/1471-230X-1-11 (https://doi.org/10.1186%2F1471-230X-1-11). PMC 59674 (https://www.ncbi.nlm.nih.gov/pmc/articles/PMC59674). PMID 11701092 (https://pubmed.ncbi.nlm.nih.gov/11701092).
68. Irvine AJ, Chey WD, Ford AC (January 2017). "Screening for Celiac Disease in Irritable Bowel Syndrome: An Updated Systematic Review and Meta-analysis" (http://eprints.whiterose.ac.uk/106483/3/AJG-16-1318R1%20CLEAN.pdf) (PDF). *The American Journal of Gastroenterology* (Review). **112** (1): 65–76. doi:10.1038/ajg.2016.466 (https://doi.org/10.1038%2Fajg.2016.466). PMID 27753436 (https://pubmed.ncbi.nlm.nih.gov/27753436). S2CID 269053 (https://api.semanticscholar.org/CorpusID:269053). "Although IBS is not a diagnosis of exclusion, with physicians advised to minimize the use of investigations, the gastrointestinal (GI) tract has a limited repertoire of symptoms, meaning that abdominal pain and a change in bowel habit is not specific to the disorder."

69. Drossman DA (February 2016). "Functional Gastrointestinal Disorders: History, Pathophysiology, Clinical Features and Rome IV" (https://cdr.lib.unc.edu/downloads/12579z328). *Gastroenterology*. **150** (6): 1262–1279.e2. doi:10.1053/j.gastro.2016.02.032 (https://doi.org/10.1053%2Fj.gastro.2016.02.032). PMID 27144617 (https://pubmed.ncbi.nlm.nih.gov/27144617). S2CID 6441439 (https://api.semanticscholar.org/CorpusID:6441439).
70. Saha L (June 2014). "Irritable bowel syndrome: pathogenesis, diagnosis, treatment, and evidence-based medicine" (https://www.ncbi.nlm.nih.gov/pmc/articles/PMC4051916). *World Journal of Gastroenterology* (Review). **20** (22): 6759–73. doi:10.3748/wjg.v20.i22.6759 (https://doi.org/10.3748%2Fwjg.v20.i22.6759). PMC 4051916 (https://www.ncbi.nlm.nih.gov/pmc/articles/PMC4051916). PMID 24944467 (https://pubmed.ncbi.nlm.nih.gov/24944467).
71. "Rome IV Criteria" (https://theromefoundation.org/rome-iv/rome-iv-criteria/). *Rome Foundation*. Retrieved April 14, 2022.
72. Fass R, Longstreth GF, Pimentel M, Fullerton S, Russak SM, Chiou CF, Reyes E, Crane P, Eisen G, McCarberg B, Ofman J (September 2001). "Evidence- and consensus-based practice guidelines for the diagnosis of irritable bowel syndrome" (https://doi.org/10.1001%2Farchinte.161.17.2081). *Archives of Internal Medicine*. **161** (17): 2081–8. doi:10.1001/archinte.161.17.2081 (https://doi.org/10.1001%2Farchinte.161.17.2081). PMID 11570936 (https://pubmed.ncbi.nlm.nih.gov/11570936).
73. Talley NJ (2006). "A unifying hypothesis for the functional gastrointestinal disorders: really multiple diseases or one irritable gut?". *Reviews in Gastroenterological Disorders*. **6** (2): 72–8. PMID 16699476 (https://pubmed.ncbi.nlm.nih.gov/16699476).
74. Hauser C (2005). *Mayo Clinic Gastroenterology and Hepatology Board Review* (https://books.google.com/books?id=nStxzRQlNaAC&pg=PA225). CRC Press. p. 225–. ISBN 978-0-203-50274-7. Archived (https://web.archive.org/web/20130510090901/http://books.google.com/books?id=nStxzRQlNaAC&pg=PA225) from the original on May 10, 2013. Retrieved October 24, 2010.
75. El-Salhy M (October 2012). "Irritable bowel syndrome: diagnosis and pathogenesis" (https://www.ncbi.nlm.nih.gov/pmc/articles/PMC3468846). *World Journal of Gastroenterology*. **18** (37): 5151–63. doi:10.3748/wjg.v18.i37.5151 (https://doi.org/10.3748%2Fwjg.v18.i37.5151). PMC 3468846 (https://www.ncbi.nlm.nih.gov/pmc/articles/PMC3468846). PMID 23066308 (https://pubmed.ncbi.nlm.nih.gov/23066308).
76. Fasano A, Sapone A, Zevallos V, Schuppan D (May 2015). "Nonceliac gluten sensitivity". *Gastroenterology* (Review). **148** (6): 1195–204. doi:10.1053/j.gastro.2014.12.049 (https://doi.org/10.1053%2Fj.gastro.2014.12.049). PMID 25583468 (https://pubmed.ncbi.nlm.nih.gov/25583468).
77. Volta U, Caio G, De Giorgio R, Henriksen C, Skodje G, Lundin KE (June 2015). "Non-celiac gluten sensitivity: a work-in-progress entity in the spectrum of wheat-related disorders". *Best Practice & Research. Clinical Gastroenterology*. **29** (3): 477–91. doi:10.1016/j.bpg.2015.04.006 (https://doi.org/10.1016%2Fj.bpg.2015.04.006). PMID 26060112 (https://pubmed.ncbi.nlm.nih.gov/26060112).
78. Rossi A, Di Lollo AC, Guzzo MP, Giacomelli C, Atzeni F, Bazzichi L, Di Franco M (2015). "Fibromyalgia and nutrition: what news?". *Clinical and Experimental Rheumatology*. **33** (1 Suppl 88): S117-25. PMID 25786053 (https://pubmed.ncbi.nlm.nih.gov/25786053).
79. San Mauro Martín I, Garicano Vilar E, Collado Yurrutia L, Ciudad Cabañas MJ (December 2014). "[Is gluten the great etiopathogenic agent of disease in the XXI century?]". *Nutricion Hospitalaria*. **30** (6): 1203–10. doi:10.3305/nh.2014.30.6.7866 (https://doi.org/10.3305%2Fnh.2014.30.6.7866). PMID 25433099 (https://pubmed.ncbi.nlm.nih.gov/25433099).

80. Catassi C, Bai JC, Bonaz B, Bouma G, Calabrò A, Carroccio A, Castillejo G, Ciacci C, Cristofori F, Dolinsek J, Francavilla R, Elli L, Green P, Holtmeier W, Koehler P, Koletzko S, Meinhold C, Sanders D, Schumann M, Schuppan D, Ullrich R, Vécsei A, Volta U, Zevallos V, Sapone A, Fasano A (September 2013). "Non-Celiac Gluten sensitivity: the new frontier of gluten related disorders" (https://www.ncbi.nlm.nih.gov/pmc/articles/PMC3820047). *Nutrients* (Review). **5** (10): 3839–53. doi:10.3390/nu5103839 (https://doi.org/10.3390%2Fnu5103839). PMC 3820047 (https://www.ncbi.nlm.nih.gov/pmc/articles/PMC3820047). PMID 24077239 (https://pubmed.ncbi.nlm.nih.gov/24077239).
81. Lebwohl B, Ludvigsson JF, Green PH (October 2015). "Celiac disease and non-celiac gluten sensitivity" (https://www.ncbi.nlm.nih.gov/pmc/articles/PMC4596973). *BMJ* (Review). **351**: h4347. doi:10.1136/bmj.h4347 (https://doi.org/10.1136%2Fbmj.h4347). PMC 4596973 (https://www.ncbi.nlm.nih.gov/pmc/articles/PMC4596973). PMID 26438584 (https://pubmed.ncbi.nlm.nih.gov/26438584).
82. Bixquert Jiménez M (August 2009). "Treatment of irritable bowel syndrome with probiotics. An etiopathogenic approach at last?" (https://doi.org/10.4321%2Fs1130-01082009000800006). *Revista Española de Enfermedades Digestivas*. **101** (8): 553–64. doi:10.4321/s1130-01082009000800006 (https://doi.org/10.4321%2Fs1130-01082009000800006). PMID 19785495 (https://pubmed.ncbi.nlm.nih.gov/19785495).
83. Spiegel BM, DeRosa VP, Gralnek IM, Wang V, Dulai GS (June 2004). "Testing for celiac sprue in irritable bowel syndrome with predominant diarrhea: a cost-effectiveness analysis" (https://zenodo.org/record/1235988). *Gastroenterology*. **126** (7): 1721–32. doi:10.1053/j.gastro.2004.03.012 (https://doi.org/10.1053%2Fj.gastro.2004.03.012). PMID 15188167 (https://pubmed.ncbi.nlm.nih.gov/15188167).
84. Su YC, Wang WM, Wang SY, Lu SN, Chen LT, Wu DC, Chen CY, Jan CM, Horowitz M (August 2000). "The association between Helicobacter pylori infection and functional dyspepsia in patients with irritable bowel syndrome". *The American Journal of Gastroenterology*. **95** (8): 1900–5. doi:10.1111/j.1572-0241.2000.02252.x (https://doi.org/10.1111%2Fj.1572-0241.2000.02252.x). PMID 10950033 (https://pubmed.ncbi.nlm.nih.gov/10950033). S2CID 22496041 (https://api.semanticscholar.org/CorpusID:22496041).
85. Gerards C, Leodolter A, Glasbrenner B, Malfertheiner P (2001). "H. pylori infection and visceral hypersensitivity in patients with irritable bowel syndrome". *Digestive Diseases*. **19** (2): 170–3. doi:10.1159/000050673 (https://doi.org/10.1159%2F000050673). PMID 11549828 (https://pubmed.ncbi.nlm.nih.gov/11549828). S2CID 6877270 (https://api.semanticscholar.org/CorpusID:6877270).
86. Grazioli B, Matera G, Laratta C, Schipani G, Guarnieri G, Spiniello E, Imeneo M, Amorosi A, Focà A, Luzza F (March 2006). "Giardia lamblia infection in patients with irritable bowel syndrome and dyspepsia: a prospective study" (http://www.wjgnet.com/1007-9327/12/1941.asp). *World Journal of Gastroenterology*. **12** (12): 1941–4. doi:10.3748/wjg.v12.i12.1941 (https://doi.org/10.3748%2Fwjg.v12.i12.1941). PMC 4087522 (https://www.ncbi.nlm.nih.gov/pmc/articles/PMC4087522). PMID 16610003 (https://pubmed.ncbi.nlm.nih.gov/16610003). Archived (https://web.archive.org/web/20090815101352/http://www.wjgnet.com/1007-9327/12/1941.asp) from the original on August 15, 2009.
87. Vernia P, Ricciardi MR, Frandina C, Bilotta T, Frieri G (April 1995). "Lactose malabsorption and irritable bowel syndrome. Effect of a long-term lactose-free diet". *The Italian Journal of Gastroenterology*. **27** (3): 117–21. PMID 7548919 (https://pubmed.ncbi.nlm.nih.gov/7548919).

88. Brandt LJ, Chey WD, Foxx-Orenstein AE, Schiller LR, Schoenfeld PS, Spiegel BM, Talley NJ, Quigley EM (January 2009). "An evidence-based position statement on the management of irritable bowel syndrome" (http://www.nature.com/ajg/journal/v104/n1s/pdf/ajg2008122a.pdf) (PDF). *The American Journal of Gastroenterology*. **104** (Suppl 1): S1-35. doi:10.1038/ajg.2008.122 (https://doi.org/10.1038%2Fajg.2008.122). PMID 19521341 (https://pubmed.ncbi.nlm.nih.gov/19521341). S2CID 12556370 (https://api.semanticscholar.org/CorpusID:12556370). Archived (https://web.archive.org/web/20101205005043/http://www.nature.com/ajg/journal/v104/n1s/pdf/ajg2008122a.pdf) (PDF) from the original on December 5, 2010.
89. Wedlake L, A'Hern R, Russell D, Thomas K, Walters JR, Andreyev HJ (October 2009). "Systematic review: the prevalence of idiopathic bile acid malabsorption as diagnosed by SeHCAT scanning in patients with diarrhoea-predominant irritable bowel syndrome" (https://doi.org/10.1111%2Fj.1365-2036.2009.04081.x). *Alimentary Pharmacology & Therapeutics*. **30** (7): 707–17. doi:10.1111/j.1365-2036.2009.04081.x (https://doi.org/10.1111%2Fj.1365-2036.2009.04081.x). PMID 19570102 (https://pubmed.ncbi.nlm.nih.gov/19570102). S2CID 11327665 (https://api.semanticscholar.org/CorpusID:11327665).
90. Cole JA, Rothman KJ, Cabral HJ, Zhang Y, Farraye FA (September 2006). "Migraine, fibromyalgia, and depression among people with IBS: a prevalence study" (https://www.ncbi.nlm.nih.gov/pmc/articles/PMC1592499). *BMC Gastroenterology*. **6**: 26. doi:10.1186/1471-230X-6-26 (https://doi.org/10.1186%2F1471-230X-6-26). PMC 1592499 (https://www.ncbi.nlm.nih.gov/pmc/articles/PMC1592499). PMID 17007634 (https://pubmed.ncbi.nlm.nih.gov/17007634).
91. Beyder A, Farrugia G (October 2016). "Ion channelopathies in functional GI disorders" (https://www.ncbi.nlm.nih.gov/pmc/articles/PMC5142191). *Am J Physiol Gastrointest Liver Physiol*. **311** (4): G581–G586. doi:10.1152/ajpgi.00237.2016 (https://doi.org/10.1152%2Fajpgi.00237.2016). PMC 5142191 (https://www.ncbi.nlm.nih.gov/pmc/articles/PMC5142191). PMID 27514480 (https://pubmed.ncbi.nlm.nih.gov/27514480).
92. Bellini M, Biagi S, Stasi C, Costa F, Mumolo MG, Ricchiuti A, Marchi S (March 2006). "Gastrointestinal manifestations in myotonic muscular dystrophy" (https://www.ncbi.nlm.nih.gov/pmc/articles/PMC4087506). *World J Gastroenterol*. **12** (12): 1821–1828. doi:10.3748/wjg.v12.i12.1821 (https://doi.org/10.3748%2Fwjg.v12.i12.1821). PMC 4087506 (https://www.ncbi.nlm.nih.gov/pmc/articles/PMC4087506). PMID 16609987 (https://pubmed.ncbi.nlm.nih.gov/16609987).
93. Bercik P, Verdu EF, Collins SM (June 2005). "Is irritable bowel syndrome a low-grade inflammatory bowel disease?". *Gastroenterology Clinics of North America*. **34** (2): 235–45, vi–vii. doi:10.1016/j.gtc.2005.02.007 (https://doi.org/10.1016%2Fj.gtc.2005.02.007). PMID 15862932 (https://pubmed.ncbi.nlm.nih.gov/15862932).
94. Quigley EM (2005). "Irritable bowel syndrome and inflammatory bowel disease: interrelated diseases?". *Chinese Journal of Digestive Diseases*. **6** (3): 122–32. doi:10.1111/j.1443-9573.2005.00202.x (https://doi.org/10.1111%2Fj.1443-9573.2005.00202.x). PMID 16045602 (https://pubmed.ncbi.nlm.nih.gov/16045602).
95. Simrén M, Axelsson J, Gillberg R, Abrahamsson H, Svedlund J, Björnsson ES (February 2002). "Quality of life in inflammatory bowel disease in remission: the impact of IBS-like symptoms and associated psychological factors". *The American Journal of Gastroenterology*. **97** (2): 389–96. doi:10.1016/S0002-9270(01)04037-0 (https://doi.org/10.1016%2FS0002-9270%2801%2904037-0). PMID 11866278 (https://pubmed.ncbi.nlm.nih.gov/11866278).
96. Minderhoud IM, Oldenburg B, Wismeijer JA, van Berge Henegouwen GP, Smout AJ (March 2004). "IBS-like symptoms in patients with inflammatory bowel disease in remission; relationships with quality of life and coping behavior". *Digestive Diseases and Sciences*. **49** (3): 469–74. doi:10.1023/B:DDAS.0000020506.84248.f9 (https://doi.org/10.1023%2FB%3ADDAS.0000020506.84248.f9). PMID 15139501 (https://pubmed.ncbi.nlm.nih.gov/15139501). S2CID 7853070 (https://api.semanticscholar.org/CorpusID:7853070).

97. García Rodríguez LA, Ruigómez A, Wallander MA, Johansson S, Olbe L (March 2000). "Detection of colorectal tumor and inflammatory bowel disease during follow-up of patients with initial diagnosis of irritable bowel syndrome". *Scandinavian Journal of Gastroenterology*. **35** (3): 306–11. doi:10.1080/003655200750024191 (https://doi.org/10.1080%2F003655200750024191). PMID 10766326 (https://pubmed.ncbi.nlm.nih.gov/10766326). S2CID 218911104 (https://api.semanticscholar.org/CorpusID:218911104).
98. Corazziari E, Attili AF, Angeletti C, De Santis A (December 2008). "Gallstones, cholecystectomy and irritable bowel syndrome (IBS) MICOL population-based study". *Digestive and Liver Disease*. **40** (12): 944–50. doi:10.1016/j.dld.2008.02.013 (https://doi.org/10.1016%2Fj.dld.2008.02.013). PMID 18406218 (https://pubmed.ncbi.nlm.nih.gov/18406218).
99. Cole JA, Yeaw JM, Cutone JA, Kuo B, Huang Z, Earnest DL, Walker AM (December 2005). "The incidence of abdominal and pelvic surgery among patients with irritable bowel syndrome". *Digestive Diseases and Sciences*. **50** (12): 2268–75. doi:10.1007/s10620-005-3047-1 (https://doi.org/10.1007%2Fs10620-005-3047-1). PMID 16416174 (https://pubmed.ncbi.nlm.nih.gov/16416174). S2CID 687380 (https://api.semanticscholar.org/CorpusID:687380).
100. Longstreth GF, Yao JF (June 2004). "Irritable bowel syndrome and surgery: a multivariable analysis". *Gastroenterology*. **126** (7): 1665–73. doi:10.1053/j.gastro.2004.02.020 (https://doi.org/10.1053%2Fj.gastro.2004.02.020). PMID 15188159 (https://pubmed.ncbi.nlm.nih.gov/15188159).
101. Tietjen GE, Bushnell CD, Herial NA, Utley C, White L, Hafeez F (2007). "Endometriosis is associated with prevalence of comorbid conditions in migraine" (https://doi.org/10.1111%2Fj.1526-4610.2007.00784.x). *Headache*. **47** (7): 1069–78. doi:10.1111/j.1526-4610.2007.00784.x (https://doi.org/10.1111%2Fj.1526-4610.2007.00784.x). PMID 17635599 (https://pubmed.ncbi.nlm.nih.gov/17635599). S2CID 34972425 (https://api.semanticscholar.org/CorpusID:34972425).
102. "Interstitial cystitis: Risk factors" (http://www.mayoclinic.com/health/interstitial-cystitis/DS00497/DSECTION=4). *Mayo Clinic*. January 20, 2009. Archived (https://web.archive.org/web/20080516123144/http://www.mayoclinic.com/health/interstitial-cystitis/DS00497/DSECTION%3D4) from the original on May 16, 2008.
103. Ford AC, Talley NJ, Spiegel BM, Foxx-Orenstein AE, Schiller L, Quigley EM, Moayyedi P (November 2008). "Effect of fibre, antispasmodics, and peppermint oil in the treatment of irritable bowel syndrome: systematic review and meta-analysis" (https://www.ncbi.nlm.nih.gov/pmc/articles/PMC2583392). *BMJ*. **337**: a2313. doi:10.1136/bmj.a2313 (https://doi.org/10.1136%2Fbmj.a2313). PMC 2583392 (https://www.ncbi.nlm.nih.gov/pmc/articles/PMC2583392). PMID 19008265 (https://pubmed.ncbi.nlm.nih.gov/19008265).
104. Ford AC, Quigley EM, Lacy BE, Lembo AJ, Saito YA, Schiller LR, Soffer EE, Spiegel BM, Moayyedi P (September 2014). "Effect of antidepressants and psychological therapies, including hypnotherapy, in irritable bowel syndrome: systematic review and meta-analysis". *The American Journal of Gastroenterology*. **109** (9): 1350–65, quiz 1366. doi:10.1038/ajg.2014.148 (https://doi.org/10.1038%2Fajg.2014.148). PMID 24935275 (https://pubmed.ncbi.nlm.nih.gov/24935275). S2CID 205100444 (https://api.semanticscholar.org/CorpusID:205100444).
105. Khanna R, MacDonald JK, Levesque BG (July 2014). "Peppermint oil for the treatment of irritable bowel syndrome: a systematic review and meta-analysis". *Journal of Clinical Gastroenterology*. **48** (6): 505–12. doi:10.1097/MCG.0b013e3182a88357 (https://doi.org/10.1097%2FMCG.0b013e3182a88357). PMID 24100754 (https://pubmed.ncbi.nlm.nih.gov/24100754). S2CID 22520810 (https://api.semanticscholar.org/CorpusID:22520810).

106. Dionne J, Ford AC, Yuan Y, Chey WD, Lacy BE, Saito YA, Quigley EM, Moayyedi P (September 2018). "A Systematic Review and Meta-Analysis Evaluating the Efficacy of a Gluten-Free Diet and a Low FODMAPs Diet in Treating Symptoms of Irritable Bowel Syndrome" (http://eprints.whiterose.ac.uk/134755/). *The American Journal of Gastroenterology*. **113** (9): 1290–1300. doi:10.1038/s41395-018-0195-4 (https://doi.org/10.1038%2Fs41395-018-0195-4). PMID 30046155 (https://pubmed.ncbi.nlm.nih.gov/30046155). S2CID 50786768 (https://api.semanticscholar.org/CorpusID:50786768).
107. Pessarelli, T., Sorge, A., Elli, L., & Costantino, A. The Gluten-free Diet and the Low-FODMAP Diet in the Management of Functional Abdominal Bloating and Distension. Frontiers in Nutrition, 2680.
108. Staudacher HM, Irving PM, Lomer MC, Whelan K (April 2014). "Mechanisms and efficacy of dietary FODMAP restriction in IBS". *Nature Reviews. Gastroenterology & Hepatology* (Review). **11** (4): 256–66. doi:10.1038/nrgastro.2013.259 (https://doi.org/10.1038%2Fnrgastro.2013.259). PMID 24445613 (https://pubmed.ncbi.nlm.nih.gov/24445613). S2CID 23001679 (https://api.semanticscholar.org/CorpusID:23001679). "An emerging body of research now demonstrates the efficacy of fermentable carbohydrate restriction in IBS. [...] However, further work is urgently needed both to confirm clinical efficacy of fermentable carbohydrate restriction in a variety of clinical subgroups and to fully characterize the effect on the gut microbiota and the colonic environ¬ment. Whether the effect on luminal bifidobacteria is clinically relevant, preventable, or long lasting, needs to be investigated. The influence on nutrient intake, dietary diversity, which might also affect the gut microbiota,137 and quality of life also requires further exploration as does the possible economic effects due to reduced physician contact and need for medication. Although further work is required to confirm its place in IBS and functional bowel disorder clinical pathways, fermentable carbohydrate restriction is an important consideration for future national and international IBS guidelines."
109. Fedewa A, Rao SS (January 2014). "Dietary fructose intolerance, fructan intolerance and FODMAPs" (https://www.ncbi.nlm.nih.gov/pmc/articles/PMC3934501). *Current Gastroenterology Reports*. **16** (1): 370. doi:10.1007/s11894-013-0370-0 (https://doi.org/10.1007%2Fs11894-013-0370-0). PMC 3934501 (https://www.ncbi.nlm.nih.gov/pmc/articles/PMC3934501). PMID 24357350 (https://pubmed.ncbi.nlm.nih.gov/24357350).
110. Gibson PR, Shepherd SJ (February 2010). "Evidence-based dietary management of functional gastrointestinal symptoms: The FODMAP approach" (https://doi.org/10.1111%2Fj.1440-1746.2009.06149.x). *Journal of Gastroenterology and Hepatology*. **25** (2): 252–8. doi:10.1111/j.1440-1746.2009.06149.x (https://doi.org/10.1111%2Fj.1440-1746.2009.06149.x). PMID 20136989 (https://pubmed.ncbi.nlm.nih.gov/20136989). S2CID 20666740 (https://api.semanticscholar.org/CorpusID:20666740).
111. Makharia A, Catassi C, Makharia GK (December 2015). "The Overlap between Irritable Bowel Syndrome and Non-Celiac Gluten Sensitivity: A Clinical Dilemma" (https://www.ncbi.nlm.nih.gov/pmc/articles/PMC4690093). *Nutrients* (Review). **7** (12): 10417–26. doi:10.3390/nu7125541 (https://doi.org/10.3390%2Fnu7125541). PMC 4690093 (https://www.ncbi.nlm.nih.gov/pmc/articles/PMC4690093). PMID 26690475 (https://pubmed.ncbi.nlm.nih.gov/26690475).
112. Greer JB, O'Keefe SJ (2011). "Microbial induction of immunity, inflammation, and cancer" (https://www.ncbi.nlm.nih.gov/pmc/articles/PMC3059938). *Frontiers in Physiology* (Review). **1**: 168. doi:10.3389/fphys.2010.00168 (https://doi.org/10.3389%2Ffphys.2010.00168). PMC 3059938 (https://www.ncbi.nlm.nih.gov/pmc/articles/PMC3059938). PMID 21423403 (https://pubmed.ncbi.nlm.nih.gov/21423403).
113. Andoh A, Tsujikawa T, Fujiyama Y (2003). "Role of dietary fiber and short-chain fatty acids in the colon". *Current Pharmaceutical Design* (Review). **9** (4): 347–58. doi:10.2174/1381612033391973 (https://doi.org/10.2174%2F1381612033391973). PMID 12570825 (https://pubmed.ncbi.nlm.nih.gov/12570825).

114. Turco R, Salvatore S, Miele E, Romano C, Marseglia GL, Staiano A (May 2018). "Does a low FODMAPs diet reduce symptoms of functional abdominal pain disorders? A systematic review in adult and paediatric population, on behalf of Italian Society of Pediatrics" (https://www.ncbi.nlm.nih.gov/pmc/articles/PMC5952847). *Italian Journal of Pediatrics* (Systematic Review). **44** (1): 53. doi:10.1186/s13052-018-0495-8 (https://doi.org/10.1186%2Fs13052-018-0495-8). PMC 5952847 (https://www.ncbi.nlm.nih.gov/pmc/articles/PMC5952847). PMID 29764491 (https://pubmed.ncbi.nlm.nih.gov/29764491).
115. Marsh A, Eslick EM, Eslick GD (April 2016). "Does a diet low in FODMAPs reduce symptoms associated with functional gastrointestinal disorders? A comprehensive systematic review and meta-analysis". *European Journal of Nutrition*. **55** (3): 897–906. doi:10.1007/s00394-015-0922-1 (https://doi.org/10.1007%2Fs00394-015-0922-1). PMID 25982757 (https://pubmed.ncbi.nlm.nih.gov/25982757). S2CID 206969839 (https://api.semanticscholar.org/CorpusID:206969839).
116. Tuck CJ, Muir JG, Barrett JS, Gibson PR (September 2014). "Fermentable oligosaccharides, disaccharides, monosaccharides and polyols: role in irritable bowel syndrome". *Expert Review of Gastroenterology & Hepatology*. **8** (7): 819–34. doi:10.1586/17474124.2014.917956 (https://doi.org/10.1586%2F17474124.2014.917956). PMID 24830318 (https://pubmed.ncbi.nlm.nih.gov/24830318). S2CID 28811344 (https://api.semanticscholar.org/CorpusID:28811344).
117. Heiman ML, Greenway FL (May 2016). "A healthy gastrointestinal microbiome is dependent on dietary diversity" (https://www.ncbi.nlm.nih.gov/pmc/articles/PMC4837298). *Molecular Metabolism* (Review). **5** (5): 317–320. doi:10.1016/j.molmet.2016.02.005 (https://doi.org/10.1016%2Fj.molmet.2016.02.005). PMC 4837298 (https://www.ncbi.nlm.nih.gov/pmc/articles/PMC4837298). PMID 27110483 (https://pubmed.ncbi.nlm.nih.gov/27110483).
118. Staudacher HM, Whelan K (August 2017). "The low FODMAP diet: recent advances in understanding its mechanisms and efficacy in IBS" (https://kclpure.kcl.ac.uk/portal/en/publications/the-low-fodmap-diet(c7f6c885-e206-4fa4-8206-576e70bd3d59).html). *Gut* (Review). **66** (8): 1517–1527. doi:10.1136/gutjnl-2017-313750 (https://doi.org/10.1136%2Fgutjnl-2017-313750). PMID 28592442 (https://pubmed.ncbi.nlm.nih.gov/28592442). S2CID 3492917 (https://api.semanticscholar.org/CorpusID:3492917).
119. Hou JK, Lee D, Lewis J (October 2014). "Diet and inflammatory bowel disease: review of patient-targeted recommendations" (https://www.ncbi.nlm.nih.gov/pmc/articles/PMC4021001). *Clinical Gastroenterology and Hepatology* (Review). **12** (10): 1592–600. doi:10.1016/j.cgh.2013.09.063 (https://doi.org/10.1016%2Fj.cgh.2013.09.063). PMC 4021001 (https://www.ncbi.nlm.nih.gov/pmc/articles/PMC4021001). PMID 24107394 (https://pubmed.ncbi.nlm.nih.gov/24107394). "Even less evidence exists for the efficacy of the SCD, FODMAP, or Paleo diet. Furthermore, the practicality of maintaining these interventions over long periods of time is doubtful. At a practical level, adherence to defined diets may result in an unnecessary financial burden or reduction in overall caloric intake in people who are already at risk for protein-calorie malnutrition."

120. Barrett JS (March 2017). "How to institute the low-FODMAP diet" (https://doi.org/10.1111%2Fjgh.13686). *Journal of Gastroenterology and Hepatology* (Review). **32** (Suppl 1): 8–10. doi:10.1111/jgh.13686 (https://doi.org/10.1111%2Fjgh.13686). PMID 28244669 (https://pubmed.ncbi.nlm.nih.gov/28244669). S2CID 24990614 (https://api.semanticscholar.org/CorpusID:24990614). "Common symptoms of IBS are bloating, abdominal pain, excessive flatus, constipation, diarrhea, or alternating bowel habit. These symptoms, however, are also common in the presentation of coeliac disease, inflammatory bowel disease, defecatory disorders, and colon cancer. Confirming the diagnosis is crucial so that appropriate therapy can be undertaken. Unfortunately, even in these alternate diagnoses, a change in diet restricting FODMAPs may improve symptoms and mask the fact that the correct diagnosis has not been made. This is the case with coeliac disease where a low-FODMAP diet can concurrently reduce dietary gluten, improving symptoms, and also affecting coeliac diagnostic indices.3,4 Misdiagnosis of intestinal diseases can lead to secondary problems such as nutritional deficiencies, cancer risk, or even mortality in the case of colon cancer."
121. "Celiac disease" (https://web.archive.org/web/20170317123604/http://www.worldgastroenterology.org/guidelines/global-guidelines/celiac-disease/celiac-disease-english). World Gastroenterology Organisation Global Guidelines. July 2016. Archived from the original (http://www.worldgastroenterology.org/guidelines/global-guidelines/celiac-disease/celiac-disease-english) on 17 March 2017. Retrieved 4 June 2018.
122. Francis CY, Whorwell PJ (July 1994). "Bran and irritable bowel syndrome: time for reappraisal". *Lancet*. **344** (8914): 39–40. doi:10.1016/S0140-6736(94)91055-3 (https://doi.org/10.1016%2FS0140-6736%2894%2991055-3). PMID 7912305 (https://pubmed.ncbi.nlm.nih.gov/7912305). S2CID 34156816 (https://api.semanticscholar.org/CorpusID:34156816).
123. Shen YH, Nahas R (February 2009). "Complementary and alternative medicine for treatment of irritable bowel syndrome" (https://www.ncbi.nlm.nih.gov/pmc/articles/PMC2642499). *Canadian Family Physician*. **55** (2): 143–8. PMC 2642499 (https://www.ncbi.nlm.nih.gov/pmc/articles/PMC2642499). PMID 19221071 (https://pubmed.ncbi.nlm.nih.gov/19221071).
124. Bijkerk CJ, de Wit NJ, Muris JW, Whorwell PJ, Knottnerus JA, Hoes AW (August 2009). "Soluble or insoluble fibre in irritable bowel syndrome in primary care? Randomised placebo controlled trial" (https://www.ncbi.nlm.nih.gov/pmc/articles/PMC3272664). *BMJ*. **339** (b3154): b3154. doi:10.1136/bmj.b3154 (https://doi.org/10.1136%2Fbmj.b3154). PMC 3272664 (https://www.ncbi.nlm.nih.gov/pmc/articles/PMC3272664). PMID 19713235 (https://pubmed.ncbi.nlm.nih.gov/19713235).
125. Ducrotté P (November 2007). "[Irritable bowel syndrome: current treatment options]". *Presse Médicale*. **36** (11 Pt 2): 1619–26. doi:10.1016/j.lpm.2007.03.008 (https://doi.org/10.1016%2Fj.lpm.2007.03.008). PMID 17490849 (https://pubmed.ncbi.nlm.nih.gov/17490849).
126. Bijkerk CJ, Muris JW, Knottnerus JA, Hoes AW, de Wit NJ (February 2004). "Systematic review: the role of different types of fibre in the treatment of irritable bowel syndrome". *Alimentary Pharmacology & Therapeutics*. **19** (3): 245–51. doi:10.1111/j.0269-2813.2004.01862.x (https://doi.org/10.1111%2Fj.0269-2813.2004.01862.x). PMID 14984370 (https://pubmed.ncbi.nlm.nih.gov/14984370). S2CID 38345912 (https://api.semanticscholar.org/CorpusID:38345912).
127. Bijkerk CJ, de Wit NJ, Muris JW, Whorwell PJ, Knottnerus JA, Hoes AW (August 2009). "Soluble or insoluble fibre in irritable bowel syndrome in primary care? Randomised placebo controlled trial" (https://www.ncbi.nlm.nih.gov/pmc/articles/PMC3272664). *BMJ*. **339** (b): b3154. doi:10.1136/bmj.b3154 (https://doi.org/10.1136%2Fbmj.b3154). PMC 3272664 (https://www.ncbi.nlm.nih.gov/pmc/articles/PMC3272664). PMID 19713235 (https://pubmed.ncbi.nlm.nih.gov/19713235).

128. Prior A, Whorwell PJ (November 1987). "Double blind study of ispaghula in irritable bowel syndrome" (https://www.ncbi.nlm.nih.gov/pmc/articles/PMC1433676). *Gut*. **28** (11): 1510–3. doi:10.1136/gut.28.11.1510 (https://doi.org/10.1136%2Fgut.28.11.1510). PMC 1433676 (https://www.ncbi.nlm.nih.gov/pmc/articles/PMC1433676). PMID 3322956 (https://pubmed.ncbi.nlm.nih.gov/3322956).
129. Jalihal A, Kurian G (1990). "Ispaghula therapy in irritable bowel syndrome: improvement in overall well-being is related to reduction in bowel dissatisfaction". *Journal of Gastroenterology and Hepatology*. **5** (5): 507–13. doi:10.1111/j.1440-1746.1990.tb01432.x (https://doi.org/10.1111%2Fj.1440-1746.1990.tb01432.x). PMID 2129822 (https://pubmed.ncbi.nlm.nih.gov/2129822). S2CID 4666481 (https://api.semanticscholar.org/CorpusID:4666481).
130. Kumar A, Kumar N, Vij JC, Sarin SK, Anand BS (February 1987). "Optimum dosage of ispaghula husk in patients with irritable bowel syndrome: correlation of symptom relief with whole gut transit time and stool weight" (https://www.ncbi.nlm.nih.gov/pmc/articles/PMC1432983). *Gut*. **28** (2): 150–5. doi:10.1136/gut.28.2.150 (https://doi.org/10.1136%2Fgut.28.2.150). PMC 1432983 (https://www.ncbi.nlm.nih.gov/pmc/articles/PMC1432983). PMID 3030900 (https://pubmed.ncbi.nlm.nih.gov/3030900).
131. Costantino A, Pessarelli T, Vecchiato M, Vecchi M, Basilisco G, Ermolao A. A practical guide to the proper prescription of physical activity in patients with irritable bowel syndrome [published online ahead of print, 2022 Sep 21]. Dig Liver Dis. 2022;S1590-8658(22)00663-6. doi:10.1016/j.dld.2022.08.034
132. Vasant DH, Paine PA, Black CJ, et al. British Society of Gastroenterology guidelines on the management of irritable bowel syndrome. Gut. 2021;70(7):1214-1240. doi:10.1136/gutjnl-2021-324598
133. Lacy BE, Pimentel M, Brenner DM, et al. ACG Clinical Guideline: Management of Irritable Bowel Syndrome. Am J Gastroenterol. 2021;116(1):17-44. doi:10.14309/ajg.0000000000001036
134. WHO Guidelines on Physical Activity and Sedentary Behaviour. Geneva: World Health Organization; 2020.
135. Ruepert L, Quartero AO, de Wit NJ, van der Heijden GJ, Rubin G, Muris JW (August 2011). "Bulking agents, antispasmodics and antidepressants for the treatment of irritable bowel syndrome" (https://www.ncbi.nlm.nih.gov/pmc/articles/PMC8745618). *The Cochrane Database of Systematic Reviews*. **2013** (8): CD003460. doi:10.1002/14651858.CD003460.pub3 (https://doi.org/10.1002%2F14651858.CD003460.pub3). PMC 8745618 (https://www.ncbi.nlm.nih.gov/pmc/articles/PMC8745618). PMID 21833945 (https://pubmed.ncbi.nlm.nih.gov/21833945). S2CID 22977015 (https://api.semanticscholar.org/CorpusID:22977015).
136. Ford AC, Brandt LJ, Young C, Chey WD, Foxx-Orenstein AE, Moayyedi P (2009). "Efficacy of 5-HT3 antagonists and 5-HT4 agonists in irritable bowel syndrome: systematic review and meta-analysis". *American Journal of Gastroenterology*. **104** (7): 1831–1843. doi:10.1038/ajg.2009.223 (https://doi.org/10.1038%2Fajg.2009.223). PMID 19471254 (https://pubmed.ncbi.nlm.nih.gov/19471254). S2CID 8042629 (https://api.semanticscholar.org/CorpusID:8042629).
137. Joo JS, Ehrenpreis ED, Gonzalez L, Kaye M, Breno S, Wexner SD, Zaitman D, Secrest K (June 1998). "Alterations in colonic anatomy induced by chronic stimulant laxatives: the cathartic colon revisited". *Journal of Clinical Gastroenterology*. **26** (4): 283–6. doi:10.1097/00004836-199806000-00014 (https://doi.org/10.1097%2F00004836-199806000-00014). PMID 9649012 (https://pubmed.ncbi.nlm.nih.gov/9649012).

138. ▪ Li F, Fu T, Tong WD, Liu BH, Li CX, Gao Y, et al. (April 2016). "Lubiprostone Is Effective in the Treatment of Chronic Idiopathic Constipation and Irritable Bowel Syndrome: A Systematic Review and Meta-Analysis of Randomized Controlled Trials". *Mayo Clinic Proceedings*. **91** (4): 456–468. doi:10.1016/j.mayocp.2016.01.015 (https://doi.org/10.1016%2Fj.mayocp.2016.01.015). PMID 27046523 (https://pubmed.ncbi.nlm.nih.gov/27046523). "Lubiprostone is a safe and efficacious drug for the treatment of chronic idiopathic constipation and irritable bowel syndrome with constipation, with limited adverse effects in 3 months of follow-up."
   ▪ "Lubiprostone: MedlinePlus Drug Information" (https://medlineplus.gov/druginfo/meds/a607034.html). *medlineplus.gov*. 2017. "Lubiprostone is also used to treat irritable bowel syndrome with constipation... in women who are at least 18 years of age."
   ▪ "Lubiprostone Oral: Uses, Side Effects, Interactions, Pictures, Warnings & Dosing - WebMD" (https://www.webmd.com/drugs/2/drug-95017/lubiprostone-oral/details). *www.webmd.com*.
   ▪ "Lubiprostone (Oral Route) Side Effects - Mayo Clinic" (https://www.mayoclinic.org/drugs-supplements/lubiprostone-oral-route/side-effects/drg-20069057?p=1). *www.mayoclinic.org*. 2021.
139. Barber P, Parkes J, Blundell D (June 1, 2012). *Further Essentials of Pharmacology for Nurses* (https://books.google.com/books?id=BjhFBgAAQBAJ&pg=PA34). McGraw-Hill Education (UK). pp. 34–. ISBN 978-0-335-24398-3. Archived (https://web.archive.org/web/20170216212139/https://books.google.com/books?id=BjhFBgAAQBAJ&pg=PA34) from the original on February 16, 2017.
140. Annaházi A, Róka R, Rosztóczy A, Wittmann T (May 2014). "Role of antispasmodics in the treatment of irritable bowel syndrome" (https://www.ncbi.nlm.nih.gov/pmc/articles/PMC4033443). *World Journal of Gastroenterology*. **20** (20): 6031–43. doi:10.3748/wjg.v20.i20.6031 (https://doi.org/10.3748%2Fwjg.v20.i20.6031). PMC 4033443 (https://www.ncbi.nlm.nih.gov/pmc/articles/PMC4033443). PMID 24876726 (https://pubmed.ncbi.nlm.nih.gov/24876726).
141. Ghoshal UC, Shukla R, Ghoshal U (March 2017). "Small Intestinal Bacterial Overgrowth and Irritable Bowel Syndrome: A Bridge between Functional Organic Dichotomy" (https://www.ncbi.nlm.nih.gov/pmc/articles/PMC5347643). *Gut and Liver*. **11** (2): 196–208. doi:10.5009/gnl16126 (https://doi.org/10.5009%2Fgnl16126). PMC 5347643 (https://www.ncbi.nlm.nih.gov/pmc/articles/PMC5347643). PMID 28274108 (https://pubmed.ncbi.nlm.nih.gov/28274108).
142. Simrén M, Barbara G, Flint HJ, Spiegel BM, Spiller RC, Vanner S, et al. (January 2013). "Intestinal microbiota in functional bowel disorders: a Rome foundation report" (https://www.ncbi.nlm.nih.gov/pmc/articles/PMC3551212). *Gut*. **62** (1): 159–76. doi:10.1136/gutjnl-2012-302167 (https://doi.org/10.1136%2Fgutjnl-2012-302167). PMC 3551212 (https://www.ncbi.nlm.nih.gov/pmc/articles/PMC3551212). PMID 22730468 (https://pubmed.ncbi.nlm.nih.gov/22730468).
143. Wald A. Talley NJ, Grover S (eds.). "Treatment of irritable bowel syndrome in adults" (http://www.uptodate.com/online/content/topic.do?topicKey=gi_dis/5811&selectedTitle=1~148&source=search_result#9). *UpToDate Inc*.
144. Ford AC, Lacy BE, Harris LA, Quigley EM, Moayyedi P (January 2019). "Effect of Antidepressants and Psychological Therapies in Irritable Bowel Syndrome: An Updated Systematic Review and Meta-Analysis" (http://eprints.whiterose.ac.uk/135430/2/AJG-18-087R1%20CLEAN.pdf) (PDF). *The American Journal of Gastroenterology*. **114** (1): 21–39. doi:10.1038/s41395-018-0222-5 (https://doi.org/10.1038%2Fs41395-018-0222-5). PMID 30177784 (https://pubmed.ncbi.nlm.nih.gov/30177784). S2CID 52151689 (https://api.semanticscholar.org/CorpusID:52151689).

145. Jackson JL, O'Malley PG, Tomkins G, Balden E, Santoro J, Kroenke K (January 2000). "Treatment of functional gastrointestinal disorders with antidepressant medications: a meta-analysis". *The American Journal of Medicine*. **108** (1): 65–72. doi:10.1016/S0002-9343(99)00299-5 (https://doi.org/10.1016%2FS0002-9343%2899%2900299-5). PMID 11059442 (https://pubmed.ncbi.nlm.nih.gov/11059442).
146. Xie C, Tang Y, Wang Y, Yu T, Wang Y, Jiang L, Lin L (August 7, 2015). "Efficacy and Safety of Antidepressants for the Treatment of Irritable Bowel Syndrome: A Meta-Analysis" (https://www.ncbi.nlm.nih.gov/pmc/articles/PMC4529302). *PLOS ONE*. **10** (8): e0127815. Bibcode:2015PLoSO..1027815X (https://ui.adsabs.harvard.edu/abs/2015PLoSO..1027815X). doi:10.1371/journal.pone.0127815 (https://doi.org/10.1371%2Fjournal.pone.0127815). PMC 4529302 (https://www.ncbi.nlm.nih.gov/pmc/articles/PMC4529302). PMID 26252008 (https://pubmed.ncbi.nlm.nih.gov/26252008).
147. Song KH, Jung HK, Kim HJ, Koo HS, Kwon YH, Shin HD, Lim HC, Shin JE, Kim SE, Cho DH, Kim JH, Kim HJ (April 2018). "Clinical Practice Guidelines for Irritable Bowel Syndrome in Korea, 2017 Revised Edition" (https://www.ncbi.nlm.nih.gov/pmc/articles/PMC5885719). *Journal of Neurogastroenterology and Motility*. **24** (2): 197–215. doi:10.5056/jnm17145 (https://doi.org/10.5056%2Fjnm17145). PMC 5885719 (https://www.ncbi.nlm.nih.gov/pmc/articles/PMC5885719). PMID 29605976 (https://pubmed.ncbi.nlm.nih.gov/29605976).
148. Lee KJ (October 2015). "Pharmacologic Agents for Chronic Diarrhea" (https://www.ncbi.nlm.nih.gov/pmc/articles/PMC4641856). *Intestinal Research*. **13** (4): 306–12. doi:10.5217/ir.2015.13.4.306 (https://doi.org/10.5217%2Fir.2015.13.4.306). PMC 4641856 (https://www.ncbi.nlm.nih.gov/pmc/articles/PMC4641856). PMID 26576135 (https://pubmed.ncbi.nlm.nih.gov/26576135).
149. Ford AC, Harris LA, Lacy BE, Quigley EM, Moayyedi P (November 2018). "Systematic review with meta-analysis: the efficacy of prebiotics, probiotics, synbiotics and antibiotics in irritable bowel syndrome" (https://doi.org/10.1111%2Fapt.15001). *Alimentary Pharmacology & Therapeutics*. **48** (10): 1044–1060. doi:10.1111/apt.15001 (https://doi.org/10.1111%2Fapt.15001). PMID 30294792 (https://pubmed.ncbi.nlm.nih.gov/30294792). S2CID 52933693 (https://api.semanticscholar.org/CorpusID:52933693).
150. "Irritable Bowel Syndrome (IBS) – Treatment" (http://www.nhs.uk/Conditions/Irritable-bowel-syndrome/Pages/Treatment.aspx). *NHS Choices*. National Health Service. Archived (https://web.archive.org/web/20121018175323/http://www.nhs.uk/Conditions/Irritable-bowel-syndrome/Pages/Treatment.aspx) from the original on October 18, 2012. Retrieved October 21, 2012.
151. Bonaz B, Sinniger V, Pellissier S (2021). "Therapeutic Potential of Vagus Nerve Stimulation for Inflammatory Bowel Diseases" (https://www.ncbi.nlm.nih.gov/pmc/articles/PMC8019822). *Frontiers in Neuroscience*. **15**: 650971. doi:10.3389/fnins.2021.650971 (https://doi.org/10.3389%2Ffnins.2021.650971). PMC 8019822 (https://www.ncbi.nlm.nih.gov/pmc/articles/PMC8019822). PMID 33828455 (https://pubmed.ncbi.nlm.nih.gov/33828455).
152. Payne SC, Furness JB, Burns O, Sedo A, Hyakumura T, Shepherd RK, Fallon JB (2019). "Anti-inflammatory Effects of Abdominal Vagus Nerve Stimulation on Experimental Intestinal Inflammation" (https://www.ncbi.nlm.nih.gov/pmc/articles/PMC6517481). *Frontiers in Neuroscience*. **13**: 418. doi:10.3389/fnins.2019.00418 (https://doi.org/10.3389%2Ffnins.2019.00418). PMC 6517481 (https://www.ncbi.nlm.nih.gov/pmc/articles/PMC6517481). PMID 31133776 (https://pubmed.ncbi.nlm.nih.gov/31133776).
153. "Non-Invasive Nerve Stimulation Shows Promise for Younger IBD Patients" (https://www.medpagetoday.com/meetingcoverage/aibd/96225). *www.medpagetoday.com*. December 15, 2021. Retrieved February 6, 2022.

154. Manheimer E, Cheng K, Wieland LS, Min LS, Shen X, Berman BM, Lao L (May 2012). "Acupuncture for treatment of irritable bowel syndrome" (https://www.ncbi.nlm.nih.gov/pmc/articles/PMC3718572). *The Cochrane Database of Systematic Reviews*. **5** (5): CD005111. doi:10.1002/14651858.CD005111.pub3 (https://doi.org/10.1002%2F14651858.CD005111.pub3). PMC 3718572 (https://www.ncbi.nlm.nih.gov/pmc/articles/PMC3718572). PMID 22592702 (https://pubmed.ncbi.nlm.nih.gov/22592702).
155. Nikfar S, Rahimi R, Rahimi F, Derakhshani S, Abdollahi M (December 2008). "Efficacy of probiotics in irritable bowel syndrome: a meta-analysis of randomized, controlled trials". *Diseases of the Colon and Rectum*. **51** (12): 1775–80. doi:10.1007/s10350-008-9335-z (https://doi.org/10.1007%2Fs10350-008-9335-z). PMID 18465170 (https://pubmed.ncbi.nlm.nih.gov/18465170). S2CID 42964491 (https://api.semanticscholar.org/CorpusID:42964491).
156. Konturek PC, Brzozowski T, Konturek SJ (December 2011). "Stress and the gut: pathophysiology, clinical consequences, diagnostic approach and treatment options". *Journal of Physiology and Pharmacology*. **62** (6): 591–9. PMID 22314561 (https://pubmed.ncbi.nlm.nih.gov/22314561).
157. "New Studies Examine the Evidence on Probiotics in IBS" (https://web.archive.org/web/20060210062320/http://www.acg.gi.org/media/releases/ACG05Release_ProbioticsinIBS.pdf) (PDF) (Press release). American College of Gastroenterology. October 31, 2005. Archived from the original (http://www.acg.gi.org/media/releases/ACG05Release_ProbioticsinIBS.pdf) (PDF) on February 10, 2006.
158. Brenner DM, Moeller MJ, Chey WD, Schoenfeld PS (April 2009). "The utility of probiotics in the treatment of irritable bowel syndrome: a systematic review". *The American Journal of Gastroenterology*. **104** (4): 1033–49, quiz 1050. doi:10.1038/ajg.2009.25 (https://doi.org/10.1038%2Fajg.2009.25). PMID 19277023 (https://pubmed.ncbi.nlm.nih.gov/19277023). S2CID 24789648 (https://api.semanticscholar.org/CorpusID:24789648).
159. Aragon G, Graham DB, Borum M, Doman DB (January 2010). "Probiotic therapy for irritable bowel syndrome" (https://www.ncbi.nlm.nih.gov/pmc/articles/PMC2886445). *Gastroenterology & Hepatology*. **6** (1): 39–44. PMC 2886445 (https://www.ncbi.nlm.nih.gov/pmc/articles/PMC2886445). PMID 20567539 (https://pubmed.ncbi.nlm.nih.gov/20567539).
160. "IBS diet: Can yogurt ease symptoms?" (http://www.mayoclinic.com/health/ibs-diet/AN01346). *Mayo Clinic*. May 21, 2008. Archived (https://web.archive.org/web/20100209073302/http://www.mayoclinic.com/health/ibs-diet/AN01346) from the original on February 9, 2010.
161. McFarland LV (May 2010). "Systematic review and meta-analysis of Saccharomyces boulardii in adult patients" (https://www.ncbi.nlm.nih.gov/pmc/articles/PMC2868213). *World Journal of Gastroenterology*. **16** (18): 2202–22. doi:10.3748/wjg.v16.i18.2202 (https://doi.org/10.3748%2Fwjg.v16.i18.2202). PMC 2868213 (https://www.ncbi.nlm.nih.gov/pmc/articles/PMC2868213). PMID 20458757 (https://pubmed.ncbi.nlm.nih.gov/20458757).
162. Ortiz-Lucas M, Tobías A, Saz P, Sebastián JJ (January 2013). "Effect of probiotic species on irritable bowel syndrome symptoms: A bring up to date meta-analysis" (http://scielo.isciii.es/pdf/diges/v105n1/original4.pdf) (PDF). *Revista Española de Enfermedades Digestivas*. **105** (1): 19–36. doi:10.4321/s1130-01082013000100005 (https://doi.org/10.4321%2Fs1130-01082013000100005). PMID 23548007 (https://pubmed.ncbi.nlm.nih.gov/23548007).

163. Xu D, Chen VL, Steiner CA, Berinstein JA, Eswaran S, Waljee AK, et al. (July 2019). "Efficacy of Fecal Microbiota Transplantation in Irritable Bowel Syndrome: A Systematic Review and Meta-Analysis" (https://www.ncbi.nlm.nih.gov/pmc/articles/PMC7257434). *The American Journal of Gastroenterology*. **114** (7): 1043–1050. doi:10.14309/ajg.0000000000000198 (https://doi.org/10.14309%2Fajg.0000000000000198). PMC 7257434 (https://www.ncbi.nlm.nih.gov/pmc/articles/PMC7257434). PMID 30908299 (https://pubmed.ncbi.nlm.nih.gov/30908299).
164. Guandalini, S.; Magazzù, G.; Chiaro, A.; La Balestra, V.; Di Nardo, G.; Gopalan, S.; Sibal, A.; Romano, C.; Canani, R. B.; Lionetti, P.; Setty, M. (2010). "VSL#3 improves symptoms in children with irritable bowel syndrome: a multicenter, randomized, placebo-controlled, double-blind, crossover study - PubMed" (https://pubmed.ncbi.nlm.nih.gov/20453678/). *Journal of Pediatric Gastroenterology and Nutrition*. Pubmed.ncbi.nlm.nih.gov. **51** (1): 24–30. doi:10.1097/MPG.0b013e3181ca4d95 (https://doi.org/10.1097%2FMPG.0b013e3181ca4d95). PMID 20453678 (https://pubmed.ncbi.nlm.nih.gov/20453678). S2CID 33659736 (https://api.semanticscholar.org/CorpusID:33659736). Retrieved August 2, 2022.
165. Kim, H. J.; Vazquez Roque, M. I.; Camilleri, M.; Stephens, D.; Burton, D. D.; Baxter, K.; Thomforde, G.; Zinsmeister, A. R. (2005). "A randomized controlled trial of a probiotic combination VSL# 3 and placebo in irritable bowel syndrome with bloating - PubMed" (https://pubmed.ncbi.nlm.nih.gov/16185307/). *Neurogastroenterology and Motility*. Pubmed.ncbi.nlm.nih.gov. **17** (5): 687–696. doi:10.1111/j.1365-2982.2005.00695.x (https://doi.org/10.1111%2Fj.1365-2982.2005.00695.x). PMID 16185307 (https://pubmed.ncbi.nlm.nih.gov/16185307). S2CID 1930115 (https://api.semanticscholar.org/CorpusID:1930115). Retrieved August 2, 2022.
166. Wilkins T, Pepitone C, Alex B, Schade RR (September 2012). "Diagnosis and management of IBS in adults". *American Family Physician*. **86** (5): 419–26. PMID 22963061 (https://pubmed.ncbi.nlm.nih.gov/22963061).
167. Rösch W, Liebregts T, Gundermann KJ, Vinson B, Holtmann G (2006). "Phytotherapy for functional dyspepsia: a review of the clinical evidence for the herbal preparation STW 5". *Phytomedicine*. **13** (Suppl 5): 114–21. doi:10.1016/j.phymed.2006.03.022 (https://doi.org/10.1016%2Fj.phymed.2006.03.022). PMID 16978851 (https://pubmed.ncbi.nlm.nih.gov/16978851).
168. Alammar N, Wang L, Saberi B, Nanavati J, Holtmann G, Shinohara RT, Mullin GE (January 2019). "The impact of peppermint oil on the irritable bowel syndrome: a meta-analysis of the pooled clinical data" (https://www.ncbi.nlm.nih.gov/pmc/articles/PMC6337770). *BMC Complementary and Alternative Medicine*. **19** (1): 21. doi:10.1186/s12906-018-2409-0 (https://doi.org/10.1186%2Fs12906-018-2409-0). PMC 6337770 (https://www.ncbi.nlm.nih.gov/pmc/articles/PMC6337770). PMID 30654773 (https://pubmed.ncbi.nlm.nih.gov/30654773).
169. Russo EB (July 1, 2016). "Clinical Endocannabinoid Deficiency Reconsidered: Current Research Supports the Theory in Migraine, Fibromyalgia, Irritable Bowel, and Other Treatment-Resistant Syndromes" (https://www.ncbi.nlm.nih.gov/pmc/articles/PMC5576607). *Cannabis and Cannabinoid Research*. **1** (1): 154–165. doi:10.1089/can.2016.0009 (https://doi.org/10.1089%2Fcan.2016.0009). PMC 5576607 (https://www.ncbi.nlm.nih.gov/pmc/articles/PMC5576607). PMID 28861491 (https://pubmed.ncbi.nlm.nih.gov/28861491).
170. Boivin M (October 2001). "Socioeconomic impact of irritable bowel syndrome in Canada" (https://doi.org/10.1155%2F2001%2F401309). *Canadian Journal of Gastroenterology*. **15** (Suppl B): 8B–11B. doi:10.1155/2001/401309 (https://doi.org/10.1155%2F2001%2F401309). PMID 11694908 (https://pubmed.ncbi.nlm.nih.gov/11694908).

171. Quigley EM, Locke GR, Mueller-Lissner S, Paulo LG, Tytgat GN, Helfrich I, Schaefer E (July 2006). "Prevalence and management of abdominal cramping and pain: a multinational survey" (https://doi.org/10.1111%2Fj.1365-2036.2006.02989.x). *Alimentary Pharmacology & Therapeutics*. **24** (2): 411–9. doi:10.1111/j.1365-2036.2006.02989.x (https://doi.org/10.1111%2Fj.1365-2036.2006.02989.x). PMID 16842469 (https://pubmed.ncbi.nlm.nih.gov/16842469). S2CID 35344863 (https://api.semanticscholar.org/CorpusID:35344863).
172. Ehlin AG, Montgomery SM, Ekbom A, Pounder RE, Wakefield AJ (August 2003). "Prevalence of gastrointestinal diseases in two British national birth cohorts" (https://www.ncbi.nlm.nih.gov/pmc/articles/PMC1773740). *Gut*. **52** (8): 1117–21. doi:10.1136/gut.52.8.1117 (https://doi.org/10.1136%2Fgut.52.8.1117). PMC 1773740 (https://www.ncbi.nlm.nih.gov/pmc/articles/PMC1773740). PMID 12865268 (https://pubmed.ncbi.nlm.nih.gov/12865268).
173. Wilson S, Roberts L, Roalfe A, Bridge P, Singh S (July 2004). "Prevalence of irritable bowel syndrome: a community survey" (https://www.ncbi.nlm.nih.gov/pmc/articles/PMC1324800). *The British Journal of General Practice*. **54** (504): 495–502. PMC 1324800 (https://www.ncbi.nlm.nih.gov/pmc/articles/PMC1324800). PMID 15239910 (https://pubmed.ncbi.nlm.nih.gov/15239910).
174. Hungin AP, Chang L, Locke GR, Dennis EH, Barghout V (June 2005). "Irritable bowel syndrome in the United States: prevalence, symptom patterns and impact" (https://doi.org/10.1111%2Fj.1365-2036.2005.02463.x). *Alimentary Pharmacology & Therapeutics*. **21** (11): 1365–75. doi:10.1111/j.1365-2036.2005.02463.x (https://doi.org/10.1111%2Fj.1365-2036.2005.02463.x). PMID 15932367 (https://pubmed.ncbi.nlm.nih.gov/15932367). S2CID 25719789 (https://api.semanticscholar.org/CorpusID:25719789).
175. Jafri W, Yakoob J, Jafri N, Islam M, Ali QM (June 2007). "Irritable bowel syndrome and health seeking behaviour in different communities of Pakistan". *The Journal of the Pakistan Medical Association*. **57** (6): 285–7. PMID 17629228 (https://pubmed.ncbi.nlm.nih.gov/17629228).
176. Jafri W, Yakoob J, Jafri N, Islam M, Ali QM (2005). "Frequency of irritable bowel syndrome in college students". *Journal of Ayub Medical College, Abbottabad*. **17** (4): 9–11. PMID 16599025 (https://pubmed.ncbi.nlm.nih.gov/16599025).
177. Schmulson M, Ortíz O, Santiago-Lomeli M, Gutiérrez-Reyes G, Gutiérrez-Ruiz MC, Robles-Díaz G, Morgan D (2006). "Frequency of functional bowel disorders among healthy volunteers in Mexico City". *Digestive Diseases*. **24** (3–4): 342–7. doi:10.1159/000092887 (https://doi.org/10.1159%2F000092887). PMID 16849861 (https://pubmed.ncbi.nlm.nih.gov/16849861). S2CID 3275233 (https://api.semanticscholar.org/CorpusID:3275233).
178. Payne S (August 2004). "Sex, gender, and irritable bowel syndrome: making the connections". *Gender Medicine*. **1** (1): 18–28. doi:10.1016/S1550-8579(04)80007-X (https://doi.org/10.1016%2FS1550-8579%2804%2980007-X). PMID 16115580 (https://pubmed.ncbi.nlm.nih.gov/16115580).
179. Kang, J. Y. (March 2005). "Systematic review: the influence of geography and ethnicity in irritable bowel syndrome" (https://onlinelibrary.wiley.com/doi/full/10.1111/j.1365-2036.2005.02396.x). *Alimentary Pharmacology and Therapeutics*. **21** (6): 663–676. doi:10.1111/j.1365-2036.2005.02396.x (https://doi.org/10.1111%2Fj.1365-2036.2005.02396.x). ISSN 0269-2813 (https://www.worldcat.org/issn/0269-2813). PMID 15771752 (https://pubmed.ncbi.nlm.nih.gov/15771752). S2CID 25990902 (https://api.semanticscholar.org/CorpusID:25990902). "A number of African and Asian authors have reported that the female predominance typical of western patients did not occur.5, 6, 8, 9, 12-16 Again, other authors did not concur with this finding.10"

180. Jackson NA, Houghton LA, Whorwell PJ, Currer B (December 1994). "Does the menstrual cycle affect anorectal physiology?". *Digestive Diseases and Sciences*. **39** (12): 2607–11. doi:10.1007/bf02087697 (https://doi.org/10.1007%2Fbf02087697). PMID 7995186 (https://pubmed.ncbi.nlm.nih.gov/7995186). S2CID 19400135 (https://api.semanticscholar.org/CorpusID:19400135).
181. Voci SC, Cramer KM (November 2009). "Gender-related traits, quality of life, and psychological adjustment among women with irritable bowel syndrome". *Quality of Life Research*. **18** (9): 1169–76. doi:10.1007/s11136-009-9532-9 (https://doi.org/10.1007%2Fs11136-009-9532-9). PMID 19728159 (https://pubmed.ncbi.nlm.nih.gov/19728159). S2CID 21629490 (https://api.semanticscholar.org/CorpusID:21629490).
182. Heitkemper, Margaret M.; Chang, Lin (January 1, 2009). "Do fluctuations in ovarian hormones affect gastrointestinal symptoms in women with irritable bowel syndrome?" (https://www.ncbi.nlm.nih.gov/pmc/articles/PMC3322543). *Gender Medicine*. **6**: 152–167. doi:10.1016/j.genm.2009.03.004 (https://doi.org/10.1016%2Fj.genm.2009.03.004). ISSN 1550-8579 (https://www.worldcat.org/issn/1550-8579). PMC 3322543 (https://www.ncbi.nlm.nih.gov/pmc/articles/PMC3322543). PMID 19406367 (https://pubmed.ncbi.nlm.nih.gov/19406367).
183. Drossman DA, Li Z, Andruzzi E, Temple RD, Talley NJ, Thompson WG, Whitehead WE, Janssens J, Funch-Jensen P, Corazziari E (September 1993). "U.S. householder survey of functional gastrointestinal disorders. Prevalence, sociodemography, and health impact". *Digestive Diseases and Sciences*. **38** (9): 1569–80. doi:10.1007/bf01303162 (https://doi.org/10.1007%2Fbf01303162). PMID 8359066 (https://pubmed.ncbi.nlm.nih.gov/8359066). S2CID 37966231 (https://api.semanticscholar.org/CorpusID:37966231).
184. Goffaux P, Michaud K, Gaudreau J, Chalaye P, Rainville P, Marchand S (September 2011). "Sex differences in perceived pain are affected by an anxious brain". *Pain*. **152** (9): 2065–73. doi:10.1016/j.pain.2011.05.002 (https://doi.org/10.1016%2Fj.pain.2011.05.002). PMID 21665365 (https://pubmed.ncbi.nlm.nih.gov/21665365). S2CID 241921 (https://api.semanticscholar.org/CorpusID:241921).
185. Sansone, Randy A.; Sansone, Lori A. (May 2, 2012). "IRRITABLE BOWEL SYNDROME: Relationships with Abuse in Childhood" (https://www.ncbi.nlm.nih.gov/pmc/articles/PMC4479362). *Innovations in Clinical Neuroscience*. **12** (5–6): 34–37. PMC 4479362 (https://www.ncbi.nlm.nih.gov/pmc/articles/PMC4479362). PMID 26155376 (https://pubmed.ncbi.nlm.nih.gov/26155376).
186. Walker EA, Katon WJ, Roy-Byrne PP, Jemelka RP, Russo J (October 1993). "Histories of sexual victimization in patients with irritable bowel syndrome or inflammatory bowel disease". *The American Journal of Psychiatry*. **150** (10): 1502–6. doi:10.1176/ajp.150.10.1502 (https://doi.org/10.1176%2Fajp.150.10.1502). PMID 8379554 (https://pubmed.ncbi.nlm.nih.gov/8379554). S2CID 21976238 (https://api.semanticscholar.org/CorpusID:21976238).
187. Brown, P. W. (July 1947). "The irritable bowel syndrome" (https://pubmed.ncbi.nlm.nih.gov/20250197/). *The Journal of the Kansas Medical Society*. **48** (7): 309–312. ISSN 0022-8699 (https://www.worldcat.org/issn/0022-8699). PMID 20250197 (https://pubmed.ncbi.nlm.nih.gov/20250197).
188. Brown, P. W. (May 5, 1950). "The irritable bowel syndrome" (https://pubmed.ncbi.nlm.nih.gov/15418074/). *Rocky Mountain Medical Journal*. **47** (5): 343–346. ISSN 0035-760X (https://www.worldcat.org/issn/0035-760X). PMID 15418074 (https://pubmed.ncbi.nlm.nih.gov/15418074).
189. Brown PW (May 1950). "The irritable bowel syndrome". *Rocky Mountain Medical Journal*. **47** (5): 343–6. PMID 15418074 (https://pubmed.ncbi.nlm.nih.gov/15418074).

190. Levy RL, Von Korff M, Whitehead WE, Stang P, Saunders K, Jhingran P, Barghout V, Feld AD (November 2001). "Costs of care for irritable bowel syndrome patients in a health maintenance organization". *The American Journal of Gastroenterology*. **96** (11): 3122–9. doi:10.1111/j.1572-0241.2001.05258.x (https://doi.org/10.1111%2Fj.1572-0241.2001.05258.x). PMID 11721759 (https://pubmed.ncbi.nlm.nih.gov/11721759). S2CID 31865692 (https://api.semanticscholar.org/CorpusID:31865692).
191. Nyrop KA, Palsson OS, Levy RL, Von Korff M, Feld AD, Turner MJ, Whitehead WE (July 2007). "Costs of health care for irritable bowel syndrome, chronic constipation, functional diarrhoea and functional abdominal pain" (https://doi.org/10.1111%2Fj.1365-2036.2007.03370.x). *Alimentary Pharmacology & Therapeutics*. **26** (2): 237–48. doi:10.1111/j.1365-2036.2007.03370.x (https://doi.org/10.1111%2Fj.1365-2036.2007.03370.x). PMID 17593069 (https://pubmed.ncbi.nlm.nih.gov/17593069). S2CID 31374266 (https://api.semanticscholar.org/CorpusID:31374266).
192. Paré P, Gray J, Lam S, Balshaw R, Khorasheh S, Barbeau M, Kelly S, McBurney CR (October 2006). "Health-related quality of life, work productivity, and health care resource utilization of subjects with irritable bowel syndrome: baseline results from LOGIC (Longitudinal Outcomes Study of Gastrointestinal Symptoms in Canada), a naturalistic study". *Clinical Therapeutics*. **28** (10): 1726–35, discussion 1710–1. doi:10.1016/j.clinthera.2006.10.010 (https://doi.org/10.1016%2Fj.clinthera.2006.10.010). PMID 17157129 (https://pubmed.ncbi.nlm.nih.gov/17157129).
193. Leong SA, Barghout V, Birnbaum HG, Thibeault CE, Ben-Hamadi R, Frech F, Ofman JJ (April 2003). "The economic consequences of irritable bowel syndrome: a US employer perspective" (https://doi.org/10.1001%2Farchinte.163.8.929). *Archives of Internal Medicine*. **163** (8): 929–35. doi:10.1001/archinte.163.8.929 (https://doi.org/10.1001%2Farchinte.163.8.929). PMID 12719202 (https://pubmed.ncbi.nlm.nih.gov/12719202).
194. Martin BC, Ganguly R, Pannicker S, Frech F, Barghout V (2003). "Utilization patterns and net direct medical cost to Medicaid of irritable bowel syndrome" (http://www.medscape.com/viewarticle/465472). *Current Medical Research and Opinion*. **19** (8): 771–80. doi:10.1185/030079903125002540 (https://doi.org/10.1185%2F030079903125002540). PMID 14687449 (https://pubmed.ncbi.nlm.nih.gov/14687449). S2CID 19353148 (https://api.semanticscholar.org/CorpusID:19353148). Archived (https://web.archive.org/web/20031220161925/http://www.medscape.com/viewarticle/465472) from the original on December 20, 2003.
195. Aroniadis OC, Brandt LJ (January 2013). "Fecal microbiota transplantation: past, present and future". *Current Opinion in Gastroenterology*. **29** (1): 79–84. doi:10.1097/MOG.0b013e32835a4b3e (https://doi.org/10.1097%2FMOG.0b013e32835a4b3e). PMID 23041678 (https://pubmed.ncbi.nlm.nih.gov/23041678). S2CID 39943619 (https://api.semanticscholar.org/CorpusID:39943619).
196. Smits LP, Bouter KE, de Vos WM, Borody TJ, Nieuwdorp M (November 2013). "Therapeutic potential of fecal microbiota transplantation". *Gastroenterology*. **145** (5): 946–53. doi:10.1053/j.gastro.2013.08.058 (https://doi.org/10.1053%2Fj.gastro.2013.08.058). PMID 24018052 (https://pubmed.ncbi.nlm.nih.gov/24018052).
197. Ford AC, Quigley EM, Lacy BE, Lembo AJ, Saito YA, Schiller LR, Soffer EE, Spiegel BM, Moayyedi P (October 2014). "Efficacy of prebiotics, probiotics, and synbiotics in irritable bowel syndrome and chronic idiopathic constipation: systematic review and meta-analysis". *The American Journal of Gastroenterology*. **109** (10): 1547–61, quiz 1546, 1562. doi:10.1038/ajg.2014.202 (https://doi.org/10.1038%2Fajg.2014.202). PMID 25070051 (https://pubmed.ncbi.nlm.nih.gov/25070051). S2CID 205100508 (https://api.semanticscholar.org/CorpusID:205100508).
198. Klotz U (February 2012). "The pharmacological profile and clinical use of mesalazine (5-aminosalicylic acid)". *Arzneimittel-Forschung*. **62** (2): 53–8. doi:10.1055/s-0031-1299685 (https://doi.org/10.1055%2Fs-0031-1299685). PMID 22344548 (https://pubmed.ncbi.nlm.nih.gov/22344548).

199. Barbara G, et al. (2009). "Aminosalicylates and other anti-inflammatory compounds for irritable bowel syndrome". *Digestive Diseases*. **27** (Suppl 1): 115–21. doi:10.1159/000268131 (https://doi.org/10.1159%2F000268131). PMID 20203507 (https://pubmed.ncbi.nlm.nih.gov/20203507). S2CID 5184633 (https://api.semanticscholar.org/CorpusID:5184633).
200. Philpott H, Nandurkar S, Lubel J, Gibson PR (January 2013). "Alternative investigations for irritable bowel syndrome". *Journal of Gastroenterology and Hepatology*. **28** (1): 73–7. doi:10.1111/j.1440-1746.2012.07291.x (https://doi.org/10.1111%2Fj.1440-1746.2012.07291.x). PMID 23033865 (https://pubmed.ncbi.nlm.nih.gov/23033865). S2CID 1877012 (https://api.semanticscholar.org/CorpusID:1877012).
201. Keszthelyi D, Troost FJ, Jonkers DM, van Eijk HM, Lindsey PJ, Dekker J, Buurman WA, Masclee AA (August 2014). "Serotonergic reinforcement of intestinal barrier function is impaired in irritable bowel syndrome". *Alimentary Pharmacology & Therapeutics*. **40** (4): 392–402. doi:10.1111/apt.12842 (https://doi.org/10.1111%2Fapt.12842). PMID 24943480 (https://pubmed.ncbi.nlm.nih.gov/24943480). S2CID 43740780 (https://api.semanticscholar.org/CorpusID:43740780).
202. DiGiacomo D, Santonicola A, Zingone F, Troncone E, Caria MC, Borgheresi P, Parrilli G, Ciacci C (April 2013). "Human leukocyte antigen DQ2/8 prevalence in non-celiac patients with gastrointestinal diseases" (https://www.ncbi.nlm.nih.gov/pmc/articles/PMC3646141). *World Journal of Gastroenterology*. **19** (16): 2507–13. doi:10.3748/wjg.v19.i16.2507 (https://doi.org/10.3748%2Fwjg.v19.i16.2507). PMC 3646141 (https://www.ncbi.nlm.nih.gov/pmc/articles/PMC3646141). PMID 23674852 (https://pubmed.ncbi.nlm.nih.gov/23674852).
203. Camilleri, Michael (2012). "Peripheral Mechanisms in Irritable Bowel Syndrome". *New England Journal of Medicine* *(NEJM)*. Massachusetts Medical Society (MMS). **367** (17): 1626–1635. doi:10.1056/nejmra1207068 (https://doi.org/10.1056%2Fnejmra1207068). ISSN 0028-4793 (https://www.worldcat.org/issn/0028-4793).

External links

- Irritable bowel syndrome (https://curlie.org/Health/Conditions_and_Diseases/Digestive_Disorders/Intestinal/Irritable_Bowel_Syndrome/) at Curlie

Retrieved from "https://en.wikipedia.org/w/index.php?title=Irritable_bowel_syndrome&oldid=1125037072"

This page was last edited on 1 December 2022, at 21:47 (UTC).

Text is available under the Creative Commons Attribution-ShareAlike License 3.0; additional terms may apply. By using this site, you agree to the Terms of Use and Privacy Policy. Wikipedia® is a registered trademark of the Wikimedia Foundation, Inc., a non-profit organization.
