# Supplementary material for: Assessment of medical information on irritable bowel syndrome information in Wikipedia and Baidu Encyclopedia: comparative study
Source: PeerJ. 2024 May 24;12:e17264. doi: 10.7717/peerj.17264 (PMC11129691; doi:10.7717/peerj.17264)
Supplement: Data S1 [file peerj-12-17264-s001.zip › σÄƒσoïμò░μì«/Wikipedia/Constipation - Wikipedia.pdf]

WIKIPEDIA

Constipation

Constipation is a bowel dysfunction that makes bowel movements infrequent or hard to pass.[2] The stool is often hard and dry.[4] Other symptoms may include abdominal pain, bloating, and feeling as if one has not completely passed the bowel movement.[3] Complications from constipation may include hemorrhoids, anal fissure or fecal impaction.[4] The normal frequency of bowel movements in adults is between three per day and three per week.[4] Babies often have three to four bowel movements per day while young children typically have two to three per day.[8]

Constipation has many causes.[4] Common causes include slow movement of stool within the colon, irritable bowel syndrome, and pelvic floor disorders.[4] Underlying associated diseases include hypothyroidism, diabetes, Parkinson's disease, celiac disease, non-celiac gluten sensitivity, colon cancer, diverticulitis, and inflammatory bowel disease.[4][5][6][9] Medications associated with constipation include opioids, certain antacids, calcium channel blockers, and anticholinergics.[4] Of those taking opioids about 90% develop constipation.[10] Constipation is more concerning when there is weight loss or anemia, blood is present in the stool, there is a history of inflammatory bowel disease or colon cancer in a person's family, or it is of new onset in someone who is older.[11]

Treatment of constipation depends on the underlying cause and the duration that it has been present.[4] Measures that may help include drinking enough fluids, eating more fiber, consumption of honey[12] and exercise.[4] If this is not effective, laxatives of the bulk forming agent, osmotic agent, stool softener, or lubricant type may be recommended.[4] Stimulant laxatives are generally reserved for when other types are not effective.[4] Other treatments may include biofeedback or in rare cases surgery.[4]

In the general population rates of constipation are 2–30 percent.[7] Among elderly people living in a care home the rate of constipation is 50–75

Constipation	
Other names	Costiveness,[1] dyschezia[2]

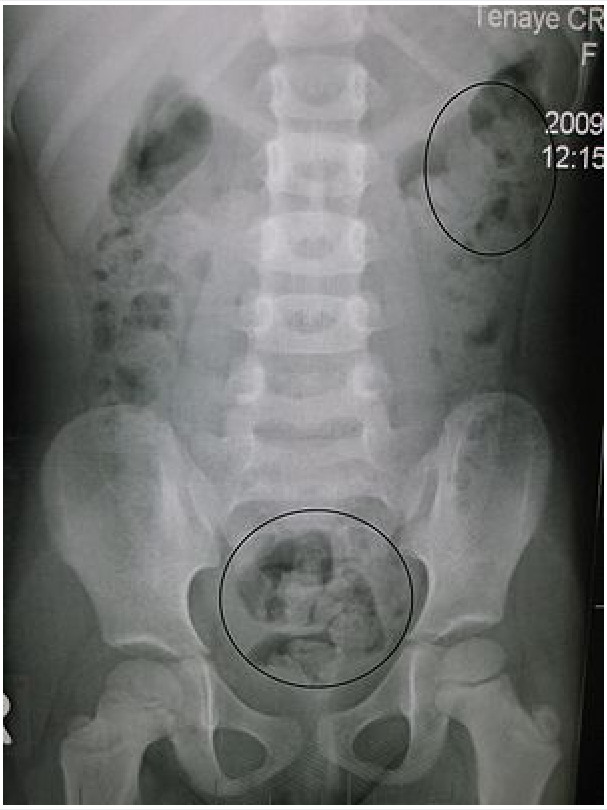

Constipation in a young child seen on X-ray. Circles represent areas of fecal matter (stool is white surrounded by black bowel gas).

Specialty	Gastroenterology
Symptoms	Infrequent or hard to pass bowel movements, abdominal pain, bloating[2][3]
Complications	Hemorrhoids, anal fissure, fecal impaction[4]
Causes	Slow movement of stool within the colon, irritable bowel syndrome, celiac disease, non-celiac gluten sensitivity, pelvic floor disorders[4][5][6]
Risk factors	Hypothyroidism, diabetes, Parkinson's disease, gluten-related disorders, colon cancer, ovarian cancer, diverticulitis, inflammatory

percent.[10] People spend, in the United States, more than US$250 million on medications for constipation a year.[13]

	bowel disease, certain medications[4][5][6]
**Treatment**	Drinking enough fluids, eating more fiber, exercise[4]
**Medication**	Laxatives of the bulk forming agent, osmotic agent, stool softener, or lubricant type[4]
**Frequency**	2–30%[7]

Contents

- Definition
- Causes
  - Diet
  - Medications
  - Medical conditions
  - Psychological
  - Congenital
- Pathophysiology
- Diagnostic approach
  - Description
  - Examination
  - Diagnostic tests
  - Criteria
- Prevention
- Treatment
  - Fiber supplements
  - Laxatives
  - Enemas
  - Physical intervention
  - Regular exercise
  - Surgical intervention
- Prognosis
- Epidemiology
- History
- Special populations
  - Children
  - Postpartum women
- See also
- References
- External links

Definition

Constipation is a symptom, not a disease. Most commonly, constipation is thought of as infrequent bowel movements, usually fewer than 3 stools per week.[14][15] However, people may have other complaints as well including:[3][16]

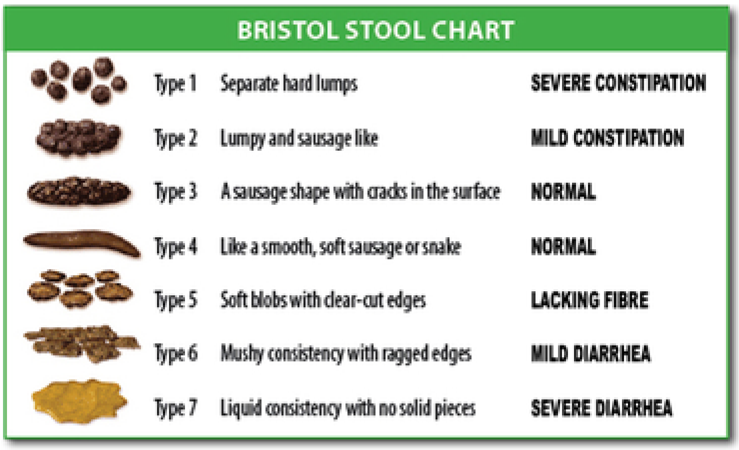

Bristol stool chart

- Straining with bowel movements
- Excessive time needed to pass a bowel movement
- Hard stools
- Pain with bowel movements secondary to straining
- Abdominal pain
- Abdominal bloating.
- the sensation of incomplete bowel evacuation.

The Rome III Criteria are a set of symptoms that help standardize the diagnosis of constipation in various age groups. These criteria help physicians to better define constipation in a standardized manner.

Causes

The causes of constipation can be divided into congenital, primary, and secondary.[2] The most common kind is primary and not life-threatening.[17] It can also be divided by the age group affected such as children and adults.

Primary or functional constipation is defined by ongoing symptoms for greater than six months not due to an underlying cause such as medication side effects or an underlying medical condition.[2][18] It is not associated with abdominal pain, thus distinguishing it from irritable bowel syndrome.[2] It is the most common kind of constipation, and is often multifactorial.[17][19] In adults, such primary causes include: dietary choices such as insufficient dietary fiber or fluid intake, or behavioral causes such as decreased physical activity. In the elderly, common causes have been attributed to insufficient dietary fiber intake, inadequate fluid intake, decreased physical activity, side effects of medications, hypothyroidism, and obstruction by colorectal cancer.[20] Evidence to support these factors however is poor.[20]

Secondary causes include side effects of medications such as opiates, endocrine and metabolic disorders such as hypothyroidism, and obstruction such as from colorectal cancer[19] or ovarian cancer.[21] Celiac disease and non-celiac gluten sensitivity may also present with constipation.[5][22][6] Cystocele can develop as a result of chronic constipation.[23]

Diet

Constipation can be caused or exacerbated by a low-fiber diet, low liquid intake, or dieting.[16][24] Dietary fiber helps to decrease colonic transport time, increases stool bulk but simultaneously softens stool. Therefore, diets low in fiber can lead to primary constipation.[19]

Medications

Many medications have constipation as a side effect. Some include (but are not limited to) opioids, diuretics, antidepressants, antihistamines, antispasmodics, anticonvulsants, tricyclic antidepressants, antiarrythmics, beta-adrenoceptor antagonists, anti-diarrheals, 5-HT3 receptor antagonists such as ondansetron, and aluminum antacids.[16][25] Certain calcium channel blockers such as nifedipine and verapamil can cause severe constipation due to dysfunction of motility in the rectosigmoid colon.[26] Supplements such as calcium and iron supplements can also have constipation as a notable side effect.

Medical conditions

Metabolic and endocrine problems which may lead to constipation include: pheochromocytoma, hypercalcemia, hypothyroidism, hyperparathyroidism, porphyria, chronic kidney disease, pan-hypopituitarism, diabetes mellitus, and cystic fibrosis.[16][17] Constipation is also common in individuals with muscular and myotonic dystrophy.[16]

Systemic diseases that may present with constipation include celiac disease and systemic sclerosis.[5][22][27]

Constipation has a number of structural (mechanical, morphological, anatomical) causes, namely through creating space-occupying lesions within the colon that stop the passage of stool, such as colorectal cancer, strictures, rectocoles, anal sphincter damage or malformation and post-surgical changes. Extra-intestinal masses such as other malignancies can also lead to constipation from external compression.[28]

Constipation also has neurological causes, including anismus, descending perineum syndrome, and Hirschsprung's disease.[7] In infants, Hirschsprung's disease is the most common medical disorder associated with constipation. Anismus occurs in a small minority of persons with chronic constipation or obstructed defecation.[29]

Spinal cord lesions and neurological disorders such as Parkinson's disease and pelvic floor dysfunction[17] can also lead to constipation.

Chagas disease may cause constipation through the destruction of the myenteric plexus.[30][31]

Psychological

Voluntary withholding of the stool is a common cause of constipation.[16] The choice to withhold can be due to factors such as fear of pain, fear of public restrooms, or laziness.[16] When a child holds in the stool a combination of encouragement, fluids, fiber, and laxatives may be useful to overcome the problem.[32] Early intervention with withholding is important as this can lead to anal fissures.[33]

Congenital

A number of diseases present at birth can result in constipation in children. They are as a group uncommon with Hirschsprung's disease (HD) being the most common.[34] There are also congenital structural anomalies that can lead to constipation, including anterior displacement of the anus, imperforate anus, strictures, and small left colon syndrome.[35]

Pathophysiology

Diagnostic approach

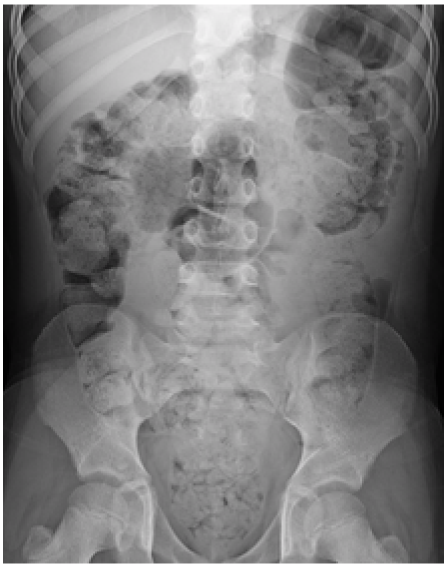

Significant constipation in the plain X-ray of an 8-year-old

The diagnosis is typically made based on a person's description of the symptoms. Bowel movements that are difficult to pass, very firm, or made up of small hard pellets (like those excreted by rabbits) qualify as constipation, even if they occur every day. Constipation is traditionally defined as three or fewer bowel movements per week.[14] Other symptoms related to constipation can include bloating, distension, abdominal pain, headaches, a feeling of fatigue and nervous exhaustion, or a sense of incomplete emptying.[36] Although constipation may be a diagnosis, it is typically viewed as a symptom that requires evaluation to discern a cause.

Description

Distinguish between acute (days to weeks) or chronic (months to years) onset of constipation because this information changes the differential diagnosis. This in the context of accompanied symptoms helps physicians discover the cause of constipation. People often describe their constipation as bowel movements that are difficult to pass, firm stool with lumpy or hard consistency, and excessive straining during bowel movements. Bloating, abdominal distension, and abdominal pain often accompany constipation.[37] Chronic constipation (symptoms present at least three days per month for more than three months) associated with abdominal discomfort is often diagnosed as irritable bowel syndrome (IBS) when no obvious cause is found.[38]

Poor dietary habits, previous abdominal surgeries, and certain medical conditions can contribute to constipation. Diseases associated with constipation include hypothyroidism, certain types of cancer, and irritable bowel syndrome. Low fiber intake, inadequate amounts of fluids, poor ambulation or immobility, or medications can contribute to constipation.[16][24] Once the presence of constipation is identified based on a culmination of the symptoms described above, then the cause of constipation should be figured out.

Separating non-life-threatening from serious causes may be partly based on symptoms. For example, colon cancer may be suspected if a person has a family history of colon cancer, fever, weight loss, and rectal bleeding.[14] Other alarming signs and symptoms include family or personal history of inflammatory bowel disease, age of onset over 50, change in stool caliber, nausea, vomiting, and neurological symptoms like weakness, numbness and difficulty urinating.[37]

Examination

A physical examination should involve at least an abdominal exam and rectal exam. Abdominal exam may reveal an abdominal mass if there is significant stool burden and may reveal abdominal discomfort. Rectal examination gives an impression of the anal sphincter tone and whether the lower rectum contains any feces or not. Rectal examination also gives information on the consistency of the stool, the presence of hemorrhoids, blood and whether any perineal irregularities are present including skin tags, fissures, anal warts.[24][16][14] Physical examination is done manually by a physician and is used to guide which diagnostic tests to order.

Diagnostic tests

Functional constipation is common and does not warrant diagnostic testing. Imaging and laboratory tests are typically recommended for those with alarm signs or symptoms.[14]

The laboratory tests performed depends on the suspected underlying cause of the constipation. Tests may include CBC (complete blood count), thyroid function tests, serum calcium, serum potassium, etc.[16][14]

Abdominal X-rays are generally only performed if bowel obstruction is suspected, may reveal extensive impacted fecal matter in the colon, and may confirm or rule out other causes of similar symptoms.[24][16]

Colonoscopy may be performed if an abnormality in the colon like a tumor is suspected.[14] Other tests rarely ordered include anorectal manometry, anal sphincter electromyography, and defecography.[16]

Colonic propagating pressure wave sequences (PSs) are responsible for discrete movements of the bowel contents and are vital for normal defecation. Deficiencies in PS frequency, amplitude, and extent of propagation are all implicated in severe defecatory dysfunction (SDD). Mechanisms that can normalize these aberrant motor patterns may help rectify the problem. Recently the novel therapy of sacral nerve stimulation (SNS) has been utilized for the treatment of severe constipation.[39]

Criteria

The Rome III Criteria for functional constipation must include two or more of the following and present for the past three months, with symptoms starting for at least 6 months prior to diagnosis.[14]

- Straining during defecation for at least 25% of bowel movements
- Lumpy or hard stools in at least 25% of defecations
- Sensation of incomplete evacuation for at least 25% of defecations
- Sensation of anorectal obstruction/blockage for at least 25% of defecations
- Manual maneuvers to facilitate at least 25% of defecations
- Fewer than 3 defecations per week
- Loose stools are rarely present without the use of laxatives
- There are insufficient criteria for irritable bowel syndrome

Prevention

Constipation is usually easier to prevent than to treat. Following the relief of constipation, maintenance with adequate exercise, fluid intake, and high-fiber diet is recommended.[16]

Treatment

A limited number of cases require urgent medical intervention or will result in severe consequences.[3]

The treatment of constipation should focus on the underlying cause if known. The National Institute of Health and Care Excellence (NICE) break constipation in adults into two categories - chronic constipation of unknown cause and constipation due to opiates.[40]

In chronic constipation of unknown cause, the main treatment involves the increased intake of water and fiber (either dietary or as supplements).[17] The routine use of laxatives or enemas is discouraged, as having bowel movements may come to be dependent upon their use.[41]

Fiber supplements

Soluble fiber supplements such as psyllium are generally considered first-line treatment for chronic constipation, compared to insoluble fibers such as wheat bran. Side effects of fiber supplements include bloating, flatulence, diarrhea, and possible malabsorption of iron, calcium, and some medications. However, patients with opiate-induced constipation will likely not benefit from fiber supplements.[33]

Laxatives

If laxatives are used, milk of magnesia or polyethylene glycol are recommended as first-line agents due to their low cost and safety.[3] Stimulants should only be used if this is not effective.[17] In cases of chronic constipation, polyethylene glycol appears superior to lactulose.[42] Prokinetics may be used to improve gastrointestinal motility. A number of new agents have shown positive outcomes in chronic constipation; these include prucalopride[43] and lubiprostone.[44] Cisapride is widely available in third world countries, but has been withdrawn in most of the west. It has not been shown to have a benefit on constipation, while potentially causing cardiac arrhythmias and deaths.[45]

Enemas

Enemas can be used to provide a form of mechanical stimulation. A large volume or high enema[46] can be given to cleanse as much of the colon as possible of feces,[47][48] and the solution administered commonly contains castile soap which irritates the colon's lining resulting in increased urgency to defecate.[49] However, a low enema is generally useful only for stool in the rectum, not in the intestinal tract.[50]

Physical intervention

Constipation that resists the above measures may require physical intervention such as manual disimpaction (the physical removal of impacted stool using the hands; see fecal impaction).

Regular exercise

Regular exercise can help improve chronic constipation.[51]

Surgical intervention

In refractory cases, procedures can be performed to help relieve constipation. Sacral nerve stimulation has been demonstrated to be effective in a minority of cases. Colectomy with ileorectal anastomosis is another intervention performed only in patients known to have a slow colonic transit time and in whom a defecation disorder has either been treated or is not present.[3] Because this is a major operation, side effects can include considerable abdominal pain, small bowel obstruction, and post-surgical infections. Furthermore, it has a very variable rate of success and is very case dependent.[33]

Prognosis

Complications that can arise from constipation include hemorrhoids, anal fissures, rectal prolapse, and fecal impaction.[16][24][52][53] Straining to pass stool may lead to hemorrhoids. In later stages of constipation, the abdomen may become distended, hard and diffusely tender. Severe cases ("fecal impaction" or *malignant constipation*) may exhibit symptoms of bowel obstruction (nausea, vomiting, tender abdomen) and encopresis, where soft stool from the small intestine bypasses the mass of impacted fecal matter in the colon.

Epidemiology

Constipation is the most common chronic gastrointestinal disorder in adults. Depending on the definition employed, it occurs in 2% to 20% of the population.[17][54] It is more common in women, the elderly and children.[54] Specifically constipation with no known cause affects females more often affected than males.[55] The reasons it occurs more frequently in the elderly is felt to be due to an increasing number of health problems as humans age and decreased physical activity.[18]

- 12% of the population worldwide reports having constipation.[56]
- Chronic constipation accounts for 3% of all visits annually to pediatric outpatient clinics.[16]
- Constipation–related health care costs total $6.9 billion in the US annually.[17]
- More than four million Americans have frequent constipation, accounting for 2.5 million physician visits a year.[53]
- Around $725 million is spent on laxative products each year in America.[53]

History

Since ancient times different societies have published medical opinions about how health care providers should respond to constipation in patients.[57] In various times and places, doctors have made claims that constipation has all sorts of medical or social causes.[57] Doctors in history have treated constipation in reasonable and unreasonable ways, including use of a spatula mundani.[57]

After the advent of the germ theory of disease then the idea of "auto-intoxication" entered popular Western thought in a fresh way.[57] Enema as a scientific medical treatment and colon cleansing as alternative medical treatment became more common in medical practice.[57]

Since the 1700s in the West there has been some popular thought that people with constipation have some moral failing with gluttony or laziness.[58]

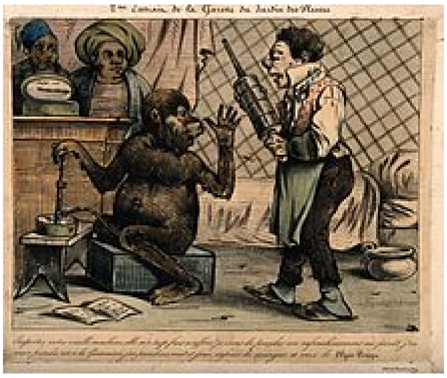
19th century satirical cartoon of a monkey rejecting an old style clyster for a new design, filled with marshmallow and opium

Special populations

Children

Approximately 3% of children have constipation, with girls and boys being equally affected.[35] With constipation accounting for approximately 5% of general pediatrician visits and 25% of pediatric gastroenterologist visits, the symptom carries a significant financial impact upon the healthcare system.[8] While it is difficult to assess an exact age at which constipation most commonly arises, children frequently experience constipation in conjunction with life-changes. Examples include: toilet training, starting or transferring to a new school, and changes in diet.[8] Especially in infants, changes in formula or transitioning from breast milk to formula can cause constipation. The majority of constipation cases are not tied to a medical disease, and treatment can be focused on simply relieving the symptoms.[35]

Postpartum women

The six-week period after pregnancy is called the postpartum stage.[59] During this time, women are at increased risk of being constipated. Multiple studies estimate the prevalence of constipation to be around 25% during the first 3 months.[60] Constipation can cause discomfort for women, as they are still recovering from the delivery process especially if they have had a perineal tear or underwent an episiotomy.[61] Risk factors that increase the risk of constipation in this population include:[61]

- Damage to the levator ani muscles (pelvic floor muscles) during childbirth
- Forceps–assisted delivery
- Lengthy second stage of labor
- Delivering a large child
- Hemorrhoids

Hemorrhoids are common in pregnancy and also may get exacerbated when constipated. Anything that can cause pain with stooling (hemorrhoids, perineal tear, episiotomy) can lead to constipation because patients may withhold from having a bowel movement so as to avoid pain.[61]

The pelvic floor muscles play an important role in helping pass a bowel movement. Injury to those muscles by some of the above risk factors (examples- delivering a large child, lengthy second stage of labor, forceps delivery) can result in constipation.[61] Enemas may be administered during labor

and these can also alter bowel movements in the days after giving birth.[59] However, there is insufficient evidence to make conclusions about the effectiveness and safety of laxatives in this group of people.[61]

See also

- Obstructed defecation
- Rectal tenesmus

References

1. "Costiveness – Definition and More from the Free Merriam-Webster Dictionary" (http://www.merriam-webster.com/dictionary/costiveness). Archived (https://web.archive.org/web/20100411110846/http://www.merriam-webster.com/dictionary/costiveness) from the original on 11 April 2010.
2. Chatoor D, Emmnauel A (2009). "Constipation and evacuation disorders". *Best Pract Res Clin Gastroenterol*. Baillière Tindall. **23** (4): 517–30. doi:10.1016/j.bpg.2009.05.001 (https://doi.org/10.1016%2Fj.bpg.2009.05.001). PMID 19647687 (https://pubmed.ncbi.nlm.nih.gov/19647687).
3. Bharucha, A. E.; Dorn, S. D.; Lembo, A.; Pressman, A. (January 2013). "American Gastroenterological Association medical position statement on constipation" (https://pubmed.ncbi.nlm.nih.gov/19647687/). *Best Practice & Research: Clinical Gastroenterology* (Review). Baillière Tindall. **144** (1): 211–217. doi:10.1053/j.gastro.2012.10.029 (https://doi.org/10.1053%2Fj.gastro.2012.10.029). PMID 23261064 (https://pubmed.ncbi.nlm.nih.gov/23261064). Archived (https://web.archive.org/web/20220220164705/https://pubmed.ncbi.nlm.nih.gov/19647687/) from the original on 20 February 2022.
4. "Constipation" (https://web.archive.org/web/20170315085221/https://www.niddk.nih.gov/health-information/digestive-diseases/constipation/all-content). *National Institute of Diabetes and Digestive and Kidney Diseases*. February 2015. Archived from the original (https://www.niddk.nih.gov/health-information/digestive-diseases/constipation/all-content) on 15 March 2017. Retrieved 14 March 2017.
5. "Symptoms & Causes of Celiac Disease | NIDDK" (https://www.niddk.nih.gov/health-information/digestive-diseases/celiac-disease/symptoms-causes). *National Institute of Diabetes and Digestive and Kidney Diseases*. June 2016. Archived (https://web.archive.org/web/20170424175500/https://www.niddk.nih.gov/health-information/digestive-diseases/celiac-disease/symptoms-causes) from the original on 24 April 2017. Retrieved 24 April 2017.
6. Makharia A, Catassi C, Makharia GK (2015). "The Overlap between Irritable Bowel Syndrome and Non-Celiac Gluten Sensitivity: A Clinical Dilemma" (https://www.ncbi.nlm.nih.gov/pmc/articles/PMC4690093). *Nutrients* (Review). **7** (12): 10417–26. doi:10.3390/nu7125541 (https://doi.org/10.3390%2Fnu7125541). PMC 4690093 (https://www.ncbi.nlm.nih.gov/pmc/articles/PMC4690093). PMID 26690475 (https://pubmed.ncbi.nlm.nih.gov/26690475).
7. Andromanakos N, Skandalakis P, Troupis T, Filippou D (2006). "Constipation of anorectal outlet obstruction: Pathophysiology, evaluation and management" (https://doi.org/10.1111%2Fj.1440-1746.2006.04333.x). *Journal of Gastroenterology and Hepatology*. **21** (4): 638–646. doi:10.1111/j.1440-1746.2006.04333.x (https://doi.org/10.1111%2Fj.1440-1746.2006.04333.x). PMID 16677147 (https://pubmed.ncbi.nlm.nih.gov/16677147). S2CID 30296908 (https://api.semanticscholar.org/CorpusID:30296908).

8. Colombo, Jennifer M.; Wassom, Matthew C.; Rosen, John M. (1 September 2015). "Constipation and Encopresis in Childhood". *Pediatrics in Review*. **36** (9): 392–401, quiz 402. doi:10.1542/pir.36-9-392 (https://doi.org/10.1542%2Fpir.36-9-392). ISSN 1526-3347 (https://www.worldcat.org/issn/1526-3347). PMID 26330473 (https://pubmed.ncbi.nlm.nih.gov/26330473). S2CID 35482415 (https://api.semanticscholar.org/CorpusID:35482415).
9. Bharucha, AE; Pemberton, JH; Locke GR, 3rd (January 2013). "American Gastroenterological Association technical review on constipation" (https://www.ncbi.nlm.nih.gov/pmc/articles/PMC3531555). *Gastroenterology*. **144** (1): 218–38. doi:10.1053/j.gastro.2012.10.028 (https://doi.org/10.1053%2Fj.gastro.2012.10.028). PMC 3531555 (https://www.ncbi.nlm.nih.gov/pmc/articles/PMC3531555). PMID 23261065 (https://pubmed.ncbi.nlm.nih.gov/23261065).
10. Canadian Agency for Drugs and Technologies in Health (26 June 2014). "Dioctyl Sulfosuccinate or Docusate (Calcium or Sodium) for the Prevention or Management of Constipation: A Review of the Clinical Effectiveness". PMID 25520993 (https://pubmed.ncbi.nlm.nih.gov/25520993).
11. Brenner, DM; Shah, M (June 2016). "Chronic Constipation". *Gastroenterology Clinics of North America*. **45** (2): 205–16. doi:10.1016/j.gtc.2016.02.013 (https://doi.org/10.1016%2Fj.gtc.2016.02.013). PMID 27261894 (https://pubmed.ncbi.nlm.nih.gov/27261894).
12. Li, Yuyuan; Long, Shangqin; Liu, Qiaochu; Ma, Hong; Li, Jianxin; Xiaoking, Wei; Yuan, Jieli; Li, Ming; Hou, Minmin (8 August 2020). "Gut microbiota is involved in the alleviation of loperamide-induced constipation by honey supplementation in mice" (https://www.ncbi.nlm.nih.gov/pmc/articles/PMC7455974). *Food Science & Nutrition*. NIH. **8** (8): 4388–4398. doi:10.1002/fsn3.1736 (https://doi.org/10.1002%2Ffsn3.1736). PMC 7455974 (https://www.ncbi.nlm.nih.gov/pmc/articles/PMC7455974). PMID 32884719 (https://pubmed.ncbi.nlm.nih.gov/32884719). "The results of this study suggested that honey can improve the symptoms of constipation by elevating fecal water content and intestinal transit rate in loperamide-induced constipation model."
13. Avunduk, Canan (2008). *Manual of gastroenterology : diagnosis and therapy* (https://books.google.com/books?id=PekixjgbRlcC&pg=PA240) (4th ed.). Philadelphia: Wolters Kluwer Health/Lippincott Williams & Wilkins. p. 240. ISBN 9780781769747. Archived (https://web.archive.org/web/20160911004050/https://books.google.com/books?id=PekixjgbRlcC&pg=PA240) from the original on 11 September 2016.
14. Jamshed, Namirah; Lee, Zone-En; Olden, Kevin W. (1 August 2011). "Diagnostic approach to chronic constipation in adults". *American Family Physician*. **84** (3): 299–306. ISSN 1532-0650 (https://www.worldcat.org/issn/1532-0650). PMID 21842777 (https://pubmed.ncbi.nlm.nih.gov/21842777).
15. "Constipation" (http://www.emedicine.com/med/topic2833.htm) Archived (https://web.archive.org/web/20070329015509/http://www.emedicine.com/med/topic2833.htm) 29 March 2007 at the Wayback Machine. eMedicine.
16. Walia R, Mahajan L, Steffen R (October 2009). "Recent advances in chronic constipation". *Curr Opin Pediatr*. **21** (5): 661–6. doi:10.1097/MOP.0b013e32832ff241 (https://doi.org/10.1097%2FMOP.0b013e32832ff241). PMID 19606041 (https://pubmed.ncbi.nlm.nih.gov/19606041). S2CID 11606786 (https://api.semanticscholar.org/CorpusID:11606786).
17. Locke GR, Pemberton JH, Phillips SF (December 2000). "American Gastroenterological Association Medical Position Statement: guidelines on constipation". *Gastroenterology*. **119** (6): 1761–6. doi:10.1053/gast.2000.20390 (https://doi.org/10.1053%2Fgast.2000.20390). PMID 11113098 (https://pubmed.ncbi.nlm.nih.gov/11113098).
18. Hsieh C (December 2005). "Treatment of constipation in older adults" (http://www.aafp.org/afp/2005/1201/p2277.html). *Am Fam Physician*. **72** (11): 2277–84. PMID 16342852 (https://pubmed.ncbi.nlm.nih.gov/16342852). Archived (https://web.archive.org/web/20120410083631/http://www.aafp.org/afp/2005/1201/p2277.html) from the original on 10 April 2012.

19. Basilisco, Guido; Coletta, Marina (2013). "Chronic constipation: A critical review" (https://doi.org/10.1016%2Fj.dld.2013.03.016). *Digestive and Liver Disease*. **45** (11): 886–893. doi:10.1016/j.dld.2013.03.016 (https://doi.org/10.1016%2Fj.dld.2013.03.016). PMID 23639342 (https://pubmed.ncbi.nlm.nih.gov/23639342).
20. Leung FW (February 2007). "Etiologic factors of chronic constipation: review of the scientific evidence". *Dig. Dis. Sci.* **52** (2): 313–6. doi:10.1007/s10620-006-9298-7 (https://doi.org/10.1007%2Fs10620-006-9298-7). PMID 17219073 (https://pubmed.ncbi.nlm.nih.gov/17219073). S2CID 608978 (https://api.semanticscholar.org/CorpusID:608978).
21. "Ovarian Cancer, Inside Knowledge, Get the Facts about Gynecological Cancer" (https://www.cdc.gov/cancer/ovarian/pdf/ovarian_facts.pdf) (PDF). Centers for Disease Control and Prevention. September 2016. Archived (https://web.archive.org/web/20170616220028/https://www.cdc.gov/cancer/ovarian/pdf/ovarian_facts.pdf) (PDF) from the original on 16 June 2017. Retrieved 17 June 2017. This article incorporates public domain material from websites or documents of the Centers for Disease Control and Prevention.
22. "Celiac disease" (http://www.worldgastroenterology.org/guidelines/global-guidelines/celiac-disease/celiac-disease-english). World Gastroenterology Organisation Global Guidelines. July 2016. Archived (https://web.archive.org/web/20170317123604/http://www.worldgastroenterology.org/guidelines/global-guidelines/celiac-disease/celiac-disease-english) from the original on 17 March 2017. Retrieved 23 April 2017.
23. "Cystocele (Prolapsed Bladder) | NIDDK" (https://www.niddk.nih.gov/health-information/urologic-diseases/bladder-control-problems-women/cystocele-prolapsed-bladder). *National Institute of Diabetes and Digestive and Kidney Diseases*. Retrieved 2 December 2017.
24. McCallum IJ, Ong S, Mercer-Jones M (2009). "Chronic constipation in adults". *BMJ*. **338**: b831. doi:10.1136/bmj.b831 (https://doi.org/10.1136%2Fbmj.b831). PMID 19304766 (https://pubmed.ncbi.nlm.nih.gov/19304766). S2CID 8291767 (https://api.semanticscholar.org/CorpusID:8291767).
25. Selby, Warwick; Corte, Crispin (August 2010). "Managing constipation in adults" (https://doi.org/10.18773%2Faustprescr.2010.058). *Australian Prescriber*. **33** (4): 116–9. doi:10.18773/austprescr.2010.058 (https://doi.org/10.18773%2Faustprescr.2010.058).
26. Gallegos-Orozco JF, Foxx-Orenstein AE, Sterler SM, Stoa JM (January 2012). "Chronic constipation in the elderly". *The American Journal of Gastroenterology* (Review). **107** (1): 18–25. doi:10.1038/ajg.2011.349 (https://doi.org/10.1038%2Fajg.2011.349). PMID 21989145 (https://pubmed.ncbi.nlm.nih.gov/21989145). S2CID 205099253 (https://api.semanticscholar.org/CorpusID:205099253).
27. Gyger G, Baron M (2015). "Systemic Sclerosis: Gastrointestinal Disease and Its Management". *Rheum Dis Clin North Am* (Review). **41** (3): 459–73. doi:10.1016/j.rdc.2015.04.007 (https://doi.org/10.1016%2Fj.rdc.2015.04.007). PMID 26210129 (https://pubmed.ncbi.nlm.nih.gov/26210129).
28. Rao, Satish S. C.; Rattanakovit, Kulthep; Patcharatrakul, Tanisa (2016). "Diagnosis and management of chronic constipation in adults". *Nature Reviews Gastroenterology & Hepatology*. **13** (5): 295–305. doi:10.1038/nrgastro.2016.53 (https://doi.org/10.1038%2Fnrgastro.2016.53). PMID 27033126 (https://pubmed.ncbi.nlm.nih.gov/27033126). S2CID 19608417 (https://api.semanticscholar.org/CorpusID:19608417).
29. Schouten WR, Briel JW, Auwerda JJ, van Dam JH, Gosselink MJ, Ginai AZ, Hop WC (1997). "Anismus: fact or fiction?". *Diseases of the Colon and Rectum*. **40** (9): 1033–1041. doi:10.1007/BF02050925 (https://doi.org/10.1007%2FBF02050925). PMID 9293931 (https://pubmed.ncbi.nlm.nih.gov/9293931). S2CID 23587867 (https://api.semanticscholar.org/CorpusID:23587867).
30. Pérez-Molina, José A.; Molina, Israel (6 January 2018). "Chagas disease". *The Lancet*. **391** (10115): 82–94. doi:10.1016/S0140-6736(17)31612-4 (https://doi.org/10.1016%2FS0140-6736%2817%2931612-4). PMID 28673423 (https://pubmed.ncbi.nlm.nih.gov/28673423). S2CID 4514617 (https://api.semanticscholar.org/CorpusID:4514617).

31. Nguyen, Tina; Waseem, Muhammad (2022). "Chagas Disease" (https://www.ncbi.nlm.nih.gov/books/NBK459272/). *StatPearls*. StatPearls Publishing. PMID 29083573 (https://pubmed.ncbi.nlm.nih.gov/29083573).
32. Cohn A (2010). "Stool withholding" (http://www.anthonycohnpaediatrics.co.uk/jpn.pdf) (PDF). *Journal of Pediatric Neurology*. **8** (1): 29–30. doi:10.3233/JPN-2010-0350 (https://doi.org/10.3233%2FJPN-2010-0350). Archived (https://web.archive.org/web/20110907082744/http://www.anthonycohnpaediatrics.co.uk/jpn.pdf) (PDF) from the original on 7 September 2011. Retrieved 7 September 2011.
33. Bharucha, Adil E.; Pemberton, John H.; Locke, G. Richard (2013). "American Gastroenterological Association Technical Review on Constipation" (https://www.ncbi.nlm.nih.gov/pmc/articles/PMC3531555). *Gastroenterology*. **144** (1): 218–238. doi:10.1053/j.gastro.2012.10.028 (https://doi.org/10.1053%2Fj.gastro.2012.10.028). PMC 3531555 (https://www.ncbi.nlm.nih.gov/pmc/articles/PMC3531555). PMID 23261065 (https://pubmed.ncbi.nlm.nih.gov/23261065).
34. Wexner, Steven (2006). *Constipation: etiology, evaluation and management*. New York: Springer.
35. Tabbers, M.M.; DiLorenzo, C.; Berger, M.Y.; Faure, C.; Langendam, M.W.; Nurko, S.; Staiano, A.; Vandenplas, Y.; Benninga, M.A. (2014). "Evaluation and Treatment of Functional Constipation in Infants and Children". *Journal of Pediatric Gastroenterology and Nutrition*. **58** (2): 265–281. doi:10.1097/mpg.0000000000000266 (https://doi.org/10.1097%2Fmpg.0000000000000266). PMID 24345831 (https://pubmed.ncbi.nlm.nih.gov/24345831). S2CID 13834963 (https://api.semanticscholar.org/CorpusID:13834963).
36. "Constipation" (http://www.medicinenet.com/constipation/article.htm) Archived (https://web.archive.org/web/20071130041024/http://www.medicinenet.com/constipation/article.htm) 30 November 2007 at the Wayback Machine MedicineNet
37. Tierney LM, Henderson MC, Smetana GW (2012). *The patient history : an evidence-based approach to differential diagnosis* (2nd ed.). New York: McGraw-Hill Medical. p. Chapter 32. ISBN 9780071624947.
38. Longstreth GF, Thompson WG, Chey WD, Houghton LA, Mearin F, Spiller RC (2006). "Functional bowel disorders". *Gastroenterology*. **130** (5): 1480–91. doi:10.1053/j.gastro.2005.11.061 (https://doi.org/10.1053%2Fj.gastro.2005.11.061). PMID 16678561 (https://pubmed.ncbi.nlm.nih.gov/16678561).
39. Dinning PG (September 2007). "Colonic manometry and sacral nerve stimulation in patients with severe constipation" (https://web.archive.org/web/20080212124726/http://www.pelviperineology.com/pelvis/severe_constipation_colonic_manometry_sacral_nerve_stimulation.html). *Pelviperineology*. **26** (3): 114–116. Archived from the original (http://www.pelviperineology.com/pelvis/severe_constipation_colonic_manometry_sacral_nerve_stimulation.html) on 12 February 2008.
40. "Constipation overview" (http://pathways.nice.org.uk/pathways/constipation). National Institute for Health and Care Excellence. Archived (https://web.archive.org/web/20150908041215/http://pathways.nice.org.uk/pathways/constipation) from the original on 8 September 2015. Retrieved 10 October 2015.
41. "Constipation" (https://www.lecturio.com/concepts/constipation/). *The Lecturio Medical Concept Library*. Retrieved 10 July 2021.
42. Lee-Robichaud H, Thomas K, Morgan J, Nelson RL (7 July 2010). "Lactulose versus Polyethylene Glycol for Chronic Constipation". *Cochrane Database of Systematic Reviews* (7): CD007570. doi:10.1002/14651858.CD007570.pub2 (https://doi.org/10.1002%2F14651858.CD007570.pub2). PMID 20614462 (https://pubmed.ncbi.nlm.nih.gov/20614462).
43. Camilleri M, Deiteren A (February 2010). "Prucalopride for constipation". *Expert Opin Pharmacother*. **11** (3): 451–61. doi:10.1517/14656560903567057 (https://doi.org/10.1517%2F14656560903567057). PMID 20102308 (https://pubmed.ncbi.nlm.nih.gov/20102308). S2CID 207478370 (https://api.semanticscholar.org/CorpusID:207478370).

44. Barish CF, Drossman D, Johanson JF, Ueno R (April 2010). "Efficacy and safety of lubiprostone in patients with chronic constipation". *Dig. Dis. Sci.* **55** (4): 1090–7. doi:10.1007/s10620-009-1068-x (https://doi.org/10.1007%2Fs10620-009-1068-x). PMID 20012484 (https://pubmed.ncbi.nlm.nih.gov/20012484). S2CID 23450010 (https://api.semanticscholar.org/CorpusID:23450010).
45. Aboumarzouk, Omar M; Agarwal, Trisha; Antakia, Ramez; Shariff, Umar; Nelson, Richard L (19 January 2011). "Cisapride for Intestinal Constipation". *Cochrane Database of Systematic Reviews* (1): CD007780. doi:10.1002/14651858.cd007780.pub2 (https://doi.org/10.1002%2F14651858.cd007780.pub2). PMID 21249695 (https://pubmed.ncbi.nlm.nih.gov/21249695).
46. "high enema" (https://www.merriam-webster.com/medical/high%20enema). *Medical Dictionary*. Merriam-Webster. Retrieved 17 February 2018.
47. "Administering an Enema" (https://web.archive.org/web/20180223111015/http://intranet.tdmu.edu.ua/data/kafedra/internal/meds/prac_skills_alg/Care%20of%20patients/Administering%20an%20Enema.htm). *Care of patients*. Ternopil State Medical University. 14 July 2015. Archived from the original (http://intranet.tdmu.edu.ua/data/kafedra/internal/meds/prac_skills_alg/Care%20of%20patients/Administering%20an%20Enema.htm) on 23 February 2018. Retrieved 17 February 2018.
48. Rhodora Cruz. "Types of Enemas" (http://www.peoi.org/Courses/Coursesen/nursepractice/ch/ch12e1.html). *Fundamentals of Nursing Practice*. Professional Education, Testing and Certification Organization International. Retrieved 17 February 2018.
49. MarileeSchmelzer, Lawrence R.Schiller, Richard Meyer, Susan M.Rugari, PattiCase (November 2004). "Safety and effectiveness of large-volume enema solutions". *Applied Nursing Research*. **17** (4): 265–274. doi:10.1016/j.apnr.2004.09.010 (https://doi.org/10.1016%2Fj.apnr.2004.09.010). PMID 15573335 (https://pubmed.ncbi.nlm.nih.gov/15573335).
50. "low enema" (https://www.merriam-webster.com/medical/low%20enema). *Medical Dictionary*. Merriam-Webster. Retrieved 17 February 2018.
51. Canberra Hospital – Gastroenterology Unit. "constipation" (http://www.betterhealth.vic.gov.au/bhcv2/bhcarticles.nsf/pages/Constipation). Archived (https://web.archive.org/web/20130717074139/http://www.betterhealth.vic.gov.au/bhcv2/bhcarticles.nsf/pages/Constipation) from the original on 17 July 2013.
52. Bharucha AE (2007). "Constipation". *Best Practice & Research Clinical Gastroenterology*. **21** (4): 709–31. doi:10.1016/j.bpg.2007.07.001 (https://doi.org/10.1016%2Fj.bpg.2007.07.001). PMID 17643910 (https://pubmed.ncbi.nlm.nih.gov/17643910).
53. National Digestive Diseases Information Clearinghouse. (2007) NIH Publication No. 07–2754. http://digestive.niddk.nih.gov/ddiseases/pubs/constipation/#treatment Archived (https://web.archive.org/web/20100818070426/http://digestive.niddk.nih.gov/ddiseases/pubs/constipation/) 18 August 2010 at the Wayback Machine, Retrieved 7-18-2010.
54. Sonnenberg A, Koch TR (1989). "Epidemiology of constipation in the United States". *Dis Colon Rectum*. **32** (1): 1–8. doi:10.1007/BF02554713 (https://doi.org/10.1007%2FBF02554713). PMID 2910654 (https://pubmed.ncbi.nlm.nih.gov/2910654). S2CID 3161661 (https://api.semanticscholar.org/CorpusID:3161661).
55. Chang L, Toner BB, Fukudo S, Guthrie E, Locke GR, Norton NJ, Sperber AD (2006). "Gender, age, society, culture, and the patient's perspective in the functional gastrointestinal disorders". *Gastroenterology*. **130** (5): 1435–46. doi:10.1053/j.gastro.2005.09.071 (https://doi.org/10.1053%2Fj.gastro.2005.09.071). PMID 16678557 (https://pubmed.ncbi.nlm.nih.gov/16678557). S2CID 8876455 (https://api.semanticscholar.org/CorpusID:8876455).

56. Wald, A.; Scarpignato, C.; Mueller-Lissner, S.; Kamm, M. A.; Hinkel, U.; Helfrich, I.; Schuijt, C.; Mandel, K. G. (1 October 2008). "A multinational survey of prevalence and patterns of laxative use among adults with self-defined constipation" (https://doi.org/10.1111%2Fj.1365-2036.2008.03806.x). *Alimentary Pharmacology & Therapeutics*. **28** (7): 917–930. doi:10.1111/j.1365-2036.2008.03806.x (https://doi.org/10.1111%2Fj.1365-2036.2008.03806.x). ISSN 1365-2036 (https://www.worldcat.org/issn/1365-2036). PMID 18644012 (https://pubmed.ncbi.nlm.nih.gov/18644012). S2CID 33659161 (https://api.semanticscholar.org/CorpusID:33659161).
57. Whorton, James C. (2000). *Inner hygiene : constipation and the pursuit of health in modern society* (https://archive.org/details/innerhygiene00jame_0). New York: Oxford University Press. ISBN 978-0195135817.
58. Hornibrook, F. A. (1929). *The culture of the abdomen;: The cure of obesity and constipation*. Heinemann.
59. Turawa, Eunice B; Musekiwa, Alfred; Rohwer, Anke C (23 September 2014). "Interventions for treating postpartum constipation". *Cochrane Database of Systematic Reviews* (9): CD010273. doi:10.1002/14651858.cd010273.pub2 (https://doi.org/10.1002%2F14651858.cd010273.pub2). PMID 25246307 (https://pubmed.ncbi.nlm.nih.gov/25246307).
60. Drossman DA, Corazziari E, Talley NJ, Grant Thompson W, Whitehead WE, editors. Rome II: the Functional Gastrointestinal Disorders. Diagnosis, Pathophysiology and Treatment: a Multinational Consensus. 2nd Edition. McLean: Degnon Associates, 2000
61. Turawa, Eunice B.; Musekiwa, Alfred; Rohwer, Anke C. (5 August 2020). "Interventions for preventing postpartum constipation" (https://www.ncbi.nlm.nih.gov/pmc/articles/PMC8094226). *The Cochrane Database of Systematic Reviews*. **2020** (8): CD011625. doi:10.1002/14651858.CD011625.pub3 (https://doi.org/10.1002%2F14651858.CD011625.pub3). hdl:10019.1/104303 (https://hdl.handle.net/10019.1%2F104303). ISSN 1469-493X (https://www.worldcat.org/issn/1469-493X). PMC 8094226 (https://www.ncbi.nlm.nih.gov/pmc/articles/PMC8094226). PMID 32761813 (https://pubmed.ncbi.nlm.nih.gov/32761813).

External links

- 09-129b. (http://www.merck.com/mmhe/sec09/ch129/ch129b.html) at *Merck Manual of Diagnosis and Therapy* Home Edition
- Constipation - Introduction (http://www.nhs.uk/Conditions/Constipation/Pages/Introduction.aspx) (UK NHS site)
- MedlinePlus Overview *constipation (https://medlineplus.gov/constipation.html)*
- Constipation Guideline (https://web.archive.org/web/20080723213827/http://www.worldgastroenterology.org/05_constipation.pdf.html) – the World Gastroenterology Organisation (WGO)

Retrieved from "https://en.wikipedia.org/w/index.php?title=Constipation&oldid=1096997764"

This page was last edited on 8 July 2022, at 01:25 (UTC).

Text is available under the Creative Commons Attribution-ShareAlike License 3.0; additional terms may apply. By using this site, you agree to the Terms of Use and Privacy Policy. Wikipedia® is a registered trademark of the Wikimedia Foundation, Inc., a non-profit organization.
