# Supplementary material for: Assessment of medical information on irritable bowel syndrome information in Wikipedia and Baidu Encyclopedia: comparative study
Source: PeerJ. 2024 May 24;12:e17264. doi: 10.7717/peerj.17264 (PMC11129691; doi:10.7717/peerj.17264)
Supplement: Data S1 [file peerj-12-17264-s001.zip › σÄƒσoïμò░μì«/Wikipedia/Dysbiosis - Wikipedia.pdf]

WIKIPEDIA

Dysbiosis

Dysbiosis	
Other names	dysbacteriosis
Treatment	Fecal Microbiota Transplantation, antibiotics, Probiotics

Dysbiosis (also called **dysbacteriosis**) is characterized by a disruption to the microbiome resulting in an imbalance in the microbiota, changes in their functional composition and metabolic activities, or a shift in their local distribution.[1][2] For example, a part of the human microbiota such as the skin flora, gut flora, or vaginal flora, can become deranged, with normally dominating species underrepresented and normally outcompeted or contained species increasing to fill the void. Dysbiosis is most commonly reported as a condition in the gastrointestinal tract.[2]

Typical microbial colonies found on or in the body are benign or beneficial. These appropriately sized microbial colonies carry out a series of helpful and necessary functions, such as aiding in digestion.[3] They also help protect the body from infiltration by pathogenic microbes. These beneficial microbial colonies compete with each other for space and resources.[4] However, when this balance is disturbed, these colonies exhibit a decreased ability to check each other's growth, which can then lead to overgrowth of one or more of the disturbed colonies which may further damage some of the other smaller beneficial ones in a vicious cycle. As more beneficial colonies are damaged, making the imbalance more pronounced, more overgrowth issues occur because the damaged colonies are less able to check the growth of the overgrowing ones. If this goes unchecked long enough, a pervasive and chronic imbalance between colonies will set in, which ultimately minimizes the beneficial nature of these colonies as a whole.[5]

Contents

- Potential Causes of Dysbiosis
  - Gut/Intestinal dysbiosis
  - Oral dysbiosis
  - Skin dysbiosis
  - Vaginal dysbiosis
  - Role of antibiotics in promoting dysbiosis
- Effects
- Associated illnesses
  - IBD
  - Obesity
  - Diabetes Mellitus
  - Cancer
  - *Clostridioides difficile*
  - Periodontitis
  - Acne vulgaris
- Treatments

Antibiotics
Fecal microbiota transplant (FMT)
Probiotics
See also
Notes and references
External links

Potential Causes of Dysbiosis

Any disruption of the body's microbiota is able to lead to dysbiosis. Dysbiosis in the gut happens when the bacteria in the gastrointestinal tract become unbalanced.[6] There are many causes for dysbiosis in the gut. Some reasons include, but are not limited to:

- Dietary changes[7][8]
- An unbalanced diet (eating large amounts of food containing simple carbohydrates and proteins, artificial colors, preservatives, nitrates and pesticides, food additives, etc.)[9]
- Antibiotics that affect your gut flora[10][11]
- Psychological and Physical Stress (weakens immune system)[12]
- Chemical consumption (Example: lingering pesticides on unwashed fruit)[7]
- Drinking two or more alcoholic beverages per day[7]
- Poor dental hygiene (allows bacteria to grow out of balance in your mouth)[7]
- Unprotected sex (exposure to harmful bacteria)[7]
- Use of radiation, chemotherapy, antiviral drugs, radioactive isotopes, and hormone therapy [13][14][15]
- Presence of intestinal helminths (human parasites)[16]
- Uncontrolled rectal cleansing with enemas[17]
- Inflammatory process in the intestine [18]
- Chronic and acute infections (HIV, Hepatitis C & B, herpes, etc.)[19][20]

Gut/Intestinal dysbiosis

Bacteria in the human gut's intestines are the most diverse in the human body and play a vital role in human health. In the gastrointestinal tract,[21] dysbiosis manifests particularly during small intestinal bacterial overgrowth (SIBO),[22] commonly caused by a decrease in the passage of food and waste through the gastrointestinal tract following surgery or other pre-existing conditions.[23] SIBO is characterized by symptoms of abdominal pain, diarrhea, discomfort after eating, and malnutrition.[23] Similarly, dysbiosis manifests during small intestinal fungal overgrowth (SIFO) caused by excessive population levels of fungi in a bowel.[24] SIFO can be characterized by GI symptoms (vomiting, diarrhea) in those previously immunocompromised.[24][25] The consumer's dietary habits can be one of the most influential factors on the gut's microbiota.[26] Diets high in carbohydrates and refined sugars are common links to dysbiosis in the gut, whereas those rich in

fruits, vegetables, and fish oils are considered more favorable to the gut due to their anti-inflammatory properties.[27] Many diseases, such as IBD, Type 2 Diabetes, Crohn's, and even allergies, are suggested to be due, in part, to an alteration in the microbiome of the gut.[27][26]

Oral dysbiosis

The mouth is frequently exposed to novel microbes from the environment, and this can lead to microbial disturbances in the mouth as well as in the stomach and intestines.[28] Hygiene and nutritional variation are imperative in preventing oral diseases such as gingivitis, tooth decay, and cavities, which are linked to altered microbial communities in the oral cavity.[29] Oral pathogens can affect multiple microbiota compartments of the body and alter systemic processes, such as immunological alterations or digestion issues. Smoking, drinking, oral intercourse, and advanced age are all associated with oral dysbiosis.[29]

Skin dysbiosis

There are a number of types of microorganisms that reside in and on the human skin, collectively known as the skin flora. Normal healthy microbial communities may have some positive effects.[30] Altered microbial composition and diversity (dysbiosis), may play a role in some non-infectious skin conditions such as acne,[31] atopic dermatitis,[32] psoriasis,[33] and rosacea.[34] In more extreme cases, such as cellulitis, a pathogenic bacteria can infect the skin, the most common being Streptococci species and Staphylococcus aureus.[35]

Vaginal dysbiosis

The vagina contains a microbiome (vaginal flora) that can become disturbed and result in conditions such as bacterial vaginosis. Alterations in vaginal flora can also affect vaginal health in reproduction, as well as one's risk of acquiring and the subsequent severity of sexually transmitted infections.[36]

Role of antibiotics in promoting dysbiosis

Dysbiosis can occur during many stages of life and can be triggered by many different sources. Antibiotics, for example, are often a significant contributor to disruptions in microbiomes. This occurs because not all microbes will be affected by the antibiotic in the same way, and so it can change the balance of different types of microbes as well as changing the total number of microbes. Antibiotic usage during young childhood development can lead to adverse gut issues (dysbiosis) in adulthood.[37] The gut microbiome is altered from antibiotics and is linked to future gut disease, i.e., IBD, ulcerative colitis, obesity, etc. The intestinal immune system is directly influenced by the gut microbiome and can be hard to recover if damaged through antibiotics.[37] The use of minocycline in acne vulgaris has been associated with skin and gut dysbiosis.[38]

Effects

Gut dysbiosis has been linked to the pathogenesis of both intestinal and extra-intestinal disorders.[26] Dysbiosis may affect intestinal disorders include IBD, IBS, and coeliac disease, as well as extra-intestinal conditions including allergies, asthma, metabolic syndrome, cardiovascular disease, and obesity.[26]

Gut dysbiosis can also be a factor in neurodegenerative and cerebrovascular diseases due to the link between age-related dysbiosis and inflammation. Inflammation is a common factor for a wide variety of age-related pathologies, including neurological diseases.[39] By correcting the dysbiosis in elderly patients, it may be possible to prevent the development of neurodegenerative diseases.[40] Dysbiosis may contribute to the cause or development of neurological conditions, including autism, pain, depression, anxiety, and stroke.[41] Dysbiosis contributing to neurological conditions is due to interactions with the gut-brain axis allowing the gut microbiome to influence neural development, cognition, and behavior.[39] There has also been evidence that the gut microbiota composition can be altered due to changes in behavior, and changing the microbiome can also cause depressive-like behaviors.[39]

Microbial colonies also excrete many different types of waste byproducts.[26] Using different waste removal mechanisms, under normal circumstances the body effectively manages these byproducts with little or no trouble. Unfortunately, oversized and inappropriately large colonies, due to their increased numbers, excrete increased amounts of these byproducts. As the amount of microbial byproducts increases, the higher waste byproducts levels can overburden the body's waste removal mechanisms.[42]

A human's microbiome can change because of inflammatory processes, such as cell-mediated inflammation and host-mediated inflammation, or a 'driver' bacteria causing/aggravating inflammation. This change allows the microbial community to become more susceptible to pathogens. Once the pathogens are established successfully, they contribute to dysbiosis and produce genotoxins and other potential cancer-causing microbial metabolites.[43] The evolution of pathogens is another possible effect of dysbiosis, contributing to a potential increase in cancer risk.[43]

Gut dysbiosis can affect the cardiovascular system "via signaling molecules and bioactive metabolites.[40] This could cause diseases through neuro-entero-endocrine hormones that can lead to heart failure and other conditions such as chronic kidney disease, hypertension, obesity, and diabetes.[40]

Associated illnesses

Cross-regulation occurs between the host and the gut microbiota in healthy people, resulting in a homeostatic equilibrium of bacteria that keeps the gastrointestinal tract healthy and free of potentially pathogenic bacteria.[42] There are three significant categories of dysbiosis: loss of beneficial organisms, excessive growth of potentially harmful microorganisms, and loss of overall microbial diversity.[42] Disruptions in the microbiome can allow outside factors or even pathogenic members of the microbiome to take hold in the gut environment. Dysbiosis has been reported to be associated with illnesses, such as multiple chemical sensitivity, periodontal disease,[44] inflammatory bowel disease,[45][46] chronic fatigue syndrome,[47] obesity,[48][49] cancer,[50][51] bacterial vaginosis,[52] and colitis.[53]

IBD

There is no single or well understood microbial cause of IBD, but three major pathogens have been associated with IBD: Mycobacterium avium paratuberculosis (MAP), adherent-invasive escherichia coli (AIEC), and clostridium difficile.[42] There is no evidence that these pathogens are the culprit

of IBD. Rather than the “one-microbe-one disease” hypothesis, it is thought that IBD is caused by an imbalance of commensal microflora associated with more complex interactions between the host and the entire intestinal microbiota.[42]

Obesity

Obesity is a metabolic condition in which the body retains an unhealthy amount of fat.[42] The prevalence of obesity in the United States is increasing, with about 93.3 million adults and 14.4 million children recorded in 2015-2016, according to the Center for Disease Control and Prevention. Similar to IBD, a specific microbiota appears to be linked to the development of obesity. There is a notable reduction in microbial diversity in obese individuals. Research in humans and animals shows an association of obesity with altered ratios between Bacteroidetes and Firmicutes; as Bacteriodetes decreases, Firmicutes increases.[42] This ratio has been linked to body weight and fat accumulation, indicating that obese people have a higher disproportionate ratio of these bacteria.[42]

Diabetes Mellitus

Diabetes mellitus (DM) is a carbohydrate metabolism disorder characterized by insufficient insulin output or utilization, which is needed for the body to turn sugars and starches into energy. The prevalence of DM in the United States is about 29.1 million, with about 1.7 million new diagnoses annually.[42] The two forms of diabetes are Type 1 and Type 2. Type 1 DM is also known as Insulin-Dependent Diabetes Mellitus (IDDM). Type 1 diabetes is an autoimmune condition that affects the beta cells in the pancreas, causing insulin production to be impaired.[42] It is most often diagnosed in children and young adults. Type 2 diabetes mellitus, also known as Non-Insulin-Dependent Diabetes Mellitus (NIDDM), is a type of diabetes that affects adults and is characterized by insulin resistance, which occurs when tissue sensitivity insulin is reduced, causing the body to ignore the insulin released. Research has shown dysbiosis of the intestinal microbiota may contribute to both forms of diabetes. Dysbiosis related to type 1 DM is characterized by a decline in mucin-degrading bacteria, such as Bifidobacteria, Lactobacillus, and Prevotella, and an increase in Bacteroidetes and Clostridium.[42]

Cancer

Sustained periods of dysbiosis lead to extended amounts of stress and inflammation in the gut microbiome, which can in turn promote the production of carcinogenic metabolites.[43] Intestinal dysbiosis has been associated with colorectal cancer (CRC). According to the American Cancer Society, colorectal cancer is the third most common cancer and the second leading cause of cancer death in the United States.[42] In CRC patients, a general dysbiosis pattern has been discovered, including a decrease in butyrate-producing bacteria and an increase in the proportion of several potentially pathogenic bacteria.[42]

Clostridioides difficile

C. difficile is an opportunistic bacteria that commonly infects patients following a disruption in the microbiome, such as treatment with antibiotics.[54][55] Infection can lead to several different symptoms including watery diarrhea, fever, loss of appetite, nausea, and abdominal pain.[56] Severe or chronic infections of *C. difficile* can lead to inflammation of the colon, a condition known as colitis.[57]

Periodontitis

Periodontitis is an oral infection that can damage the bones supporting teeth and lead to tooth loss.[58] One of the major risk factors for periodontitis is the disruption of the oral microbiome such that there is an accumulation of pathogenic bacteria.[44] Studies show that the oral microbiota changes as periodontitis progress, shifting from gram-positive aerobes to gram-negative anaerobes. Oral dysbiosis is likely to evolve, shifting the symbiotic host-microbe relationship to a pathogenic one. During this time, the host's oral health deteriorates, eventually leading to clinical disease.[44]

Acne vulgaris

The use of minocycline in acne vulgaris has been associated with skin and gut dysbiosis.[38]

Treatments

Antibiotics

Because of the complex interactions in the microbiome, not much data exists on the efficacy of using antibiotics to treat dysbiosis. However, the broad-spectrum antibiotic rifaximin has been shown to have a favorable response in several of the ailments associated with dysbiosis, including irritable bowel syndrome.[59]

While most antibiotics alter the gut microbiota for the duration of the treatment, some cause long-lasting changes. However, repeated exposure to antibiotics can also cause the opposite of the intended effect and destabilize the gut microbiome, resulting in promoting “outgrowth of antibiotic-resistant pathogenic bacteria” (see antibiotic misuse) thus aggravating gut dysbiosis.[60]

Fecal microbiota transplant (FMT)

Fecal Microbiota Transplantation (FMT) is an experimental treatment that has resolved 80-90 percent of dysbiosis-related infections caused by recurrent *C. difficile* infections that do not respond to antibiotics in randomized, controlled clinical trials.[61] A patient's colon is transplanted during FMT with a fecal preparation from a carefully screened, healthy stool donor. FMT is thought to work by repopulating the patient's microbiome with various microorganisms that compete with *C. difficile* for space.[62]

FMTs use the same line of reasoning as probiotics; to recreate a healthy balance of microbiota in the microbiome by inserting beneficial microbes into the environment. FMT accomplishes this by taking a donation of fecal matter from a healthy individual, diluted, strained and introduced to a diseased patient.[63] FMTs are currently used to treat patients with Clostridium difficile infections, who have proved resistant to other therapies.;[64] however, this is considered an investigational

therapy at present with risks that have not been fully defined.[65] FMT is also being investigated for use in psychiatric disorders.[66] Because the process is not sterile and contaminations can pass from donor to patient, there is a push to isolate key microbiota and culture them independently.[67]

Probiotics

The World Health Organization defines probiotics as "live microorganisms, which when administered in adequate amounts, confer a health benefit on the host".[68] The benefit of using probiotics to treat dysbiosis related diseases lies in its ability to treat the underlying cause of said diseases. Some benefits include their ability to suppress inflammation in the microbiome[69][70] and disrupt colonization by pathogens.[71]

Excessive use of antibiotics, IBD, obesity, diabetes, cardiovascular disease, and many more ailments are related to interruptions in the microbiome(dysbiosis), especially in the human gut. Probiotics can promote healthier microbial function by introducing or reintroducing helpful bacteria to strengthen the weaknesses presented in a dysbiotic microbiome.[72] It is essential to recognize that such circumstances are beneficial bacteria that occur more frequently than harmful ones. Probiotics can be utilized in aiding existing conditions and preventing such diseases by instituting anti-inflammatory properties and improving immune cell function.[72]

The human gut contains a wide diversity of bacteria and can easily be disrupted through diet, medicinal usage, diseases, and many others. Probiotics have proven influential in returning the intestinal microbiota to homeostatic balance and improve intestinal health.[73]

Probiotics contain anti-inflammatory properties that assist in the prevention and treatment of intestinal diseases due to microbial dysbiosis. More research is needed to understand better the many benefits probiotics can offer for multiple forms of dysbiosis.[73] Lactobacillus is the most researched single strain of probiotic bacteria.[74] It is sold to consumers for gut health either as a single strain or part of a multi-strain formulation. To date, the most clinically researched multi-strain probiotic with over 70 human clinical trials since the 1990s for various forms of dysbiosis is the De Simone Formulation. The formulation was first sold and researched under the brand name VSL#3 until 2016 when the makers of VSL#3 changed their formulation.[75] Since 2016, the original formulation known as the De Simone Forumulation is sold under various regional brand names including Visbiome in the United States and Canada plus Vivomixx in Europe. Studies since 2016 reference the probiotic either by its formulation name (De Simone Formulation) or regional brand name.[76][77]

See also

- Gut flora
- Human microbiome
- List of bacterial vaginosis microbiota
- Microbiome

Notes and references

1. Tamboli CP, Neut C, Desreumaux P, Colombel JF (January 2004). "Dysbiosis in inflammatory bowel disease" (https://www.ncbi.nlm.nih.gov/pmc/articles/PMC1773911). *Gut*. **53** (1): 1–4. doi:10.1136/gut.53.1.1 (https://doi.org/10.1136%2Fgut.53.1.1). PMC 1773911 (https://www.ncbi.nlm.nih.gov/pmc/articles/PMC1773911). PMID 14684564 (https://pubmed.ncbi.nlm.nih.gov/14684564).

2. Moos WH, Faller DV, Harpp DN, Kanara I, Pernokas J, Powers WR, Steliou K (2016). "Microbiota and Neurological Disorders: A Gut Feeling" (https://www.ncbi.nlm.nih.gov/pmc/articles/PMC4892191). *BioResearch Open Access*. **5** (1): 137–45. doi:10.1089/biores.2016.0010 (https://doi.org/10.1089%2Fbiores.2016.0010). PMC 4892191 (https://www.ncbi.nlm.nih.gov/pmc/articles/PMC4892191). PMID 27274912 (https://pubmed.ncbi.nlm.nih.gov/27274912). "As reviewed in this report, synthetic biology shows potential in developing microorganisms for correcting pathogenic dysbiosis (gut microbiota–host maladaptation), although this has yet to be proven."
3. Kau AL, Ahern PP, Griffin NW, Goodman AL, Gordon JI (June 2011). "Human nutrition, the gut microbiome and the immune system" (https://www.ncbi.nlm.nih.gov/pmc/articles/PMC3298082). *Nature*. **474** (7351): 327–36. doi:10.1038/nature10213 (https://doi.org/10.1038%2Fnature10213). PMC 3298082 (https://www.ncbi.nlm.nih.gov/pmc/articles/PMC3298082). PMID 21677749 (https://pubmed.ncbi.nlm.nih.gov/21677749).
4. Xuan C, Shamonki JM, Chung A, Dinome ML, Chung M, Sieling PA, Lee DJ (2014-01-08). "Microbial dysbiosis is associated with human breast cancer" (https://www.ncbi.nlm.nih.gov/pmc/articles/PMC3885448). *PLOS ONE*. **9** (1): e83744. Bibcode:2014PLoSO...983744X (https://ui.adsabs.harvard.edu/abs/2014PLoSO...983744X). doi:10.1371/journal.pone.0083744 (https://doi.org/10.1371%2Fjournal.pone.0083744). PMC 3885448 (https://www.ncbi.nlm.nih.gov/pmc/articles/PMC3885448). PMID 24421902 (https://pubmed.ncbi.nlm.nih.gov/24421902).
5. DeGruttola AK, Low D, Mizoguchi A, Mizoguchi E (May 2016). "Current Understanding of Dysbiosis in Disease in Human and Animal Models" (https://www.ncbi.nlm.nih.gov/pmc/articles/PMC4838534). *Inflammatory Bowel Diseases*. **22** (5): 1137–1150. doi:10.1097/MIB.0000000000000750 (https://doi.org/10.1097%2FMIB.0000000000000750). PMC 4838534 (https://www.ncbi.nlm.nih.gov/pmc/articles/PMC4838534). PMID 27070911 (https://pubmed.ncbi.nlm.nih.gov/27070911).
6. WebMD Editorial Contributors. "What Is Dysbiosis?" (https://www.webmd.com/digestive-disorders/what-is-dysbiosis). *WebMD*. Retrieved 2022-12-10.
7. Jewell T (2017-09-19). Sethi S (ed.). "What Causes Dysbiosis and How Is It Treated?" (https://www.healthline.com/health/digestive-health/dysbiosis). *Healthline*. Retrieved 2022-12-10.
8. Buttó LF, Haller D (August 2016). "Dysbiosis in intestinal inflammation: Cause or consequence". *International Journal of Medical Microbiology*. **306** (5): 302–309. doi:10.1016/j.ijmm.2016.02.010 (https://doi.org/10.1016%2Fj.ijmm.2016.02.010). PMID 27012594 (https://pubmed.ncbi.nlm.nih.gov/27012594).
9. Yaneff J (2018-05-23). "Dysbiosis Diet: How Diet Impacts Dysbiosis and Gut Flora Imbalance" (https://www.doctorshealthpress.com/dysbiosis-diet/). *Doctors Health Press – Daily Free Health Articles and Natural Health Advice*. Retrieved 2022-12-10.
10. Hawrelak JA, Myers SP (June 2004). "The causes of intestinal dysbiosis: a review" (https://pubmed.ncbi.nlm.nih.gov/15253677/). *Alternative Medicine Review*. **9** (2): 180–197. PMID 15253677 (https://pubmed.ncbi.nlm.nih.gov/15253677).
11. Buttó LF, Haller D (August 2016). "Dysbiosis in intestinal inflammation: Cause or consequence". *International Journal of Medical Microbiology*. **306** (5): 302–309. doi:10.1016/j.ijmm.2016.02.010 (https://doi.org/10.1016%2Fj.ijmm.2016.02.010). PMID 27012594 (https://pubmed.ncbi.nlm.nih.gov/27012594).
12. Hawrelak JA, Myers SP (June 2004). "The causes of intestinal dysbiosis: a review" (https://pubmed.ncbi.nlm.nih.gov/15253677/). *Alternative Medicine Review*. **9** (2): 180–197. PMID 15253677 (https://pubmed.ncbi.nlm.nih.gov/15253677).

13. Wang Z, Wang Q, Wang X, Zhu L, Chen J, Zhang B, et al. (May 2019). "Gut microbial dysbiosis is associated with development and progression of radiation enteritis during pelvic radiotherapy" (https://www.ncbi.nlm.nih.gov/pmc/articles/PMC6484301). *Journal of Cellular and Molecular Medicine*. **23** (5): 3747–3756. doi:10.1111/jcmm.14289 (https://doi.org/10.1111%2Fjcmm.14289). PMC 6484301 (https://www.ncbi.nlm.nih.gov/pmc/articles/PMC6484301). PMID 30908851 (https://pubmed.ncbi.nlm.nih.gov/30908851).
14. Luo N, Zhu W, Li X, Fu M, Peng X, Yang F, et al. (April 2022). "Impact of Gut Microbiota on Radiation-Associated Cognitive Dysfunction and Neuroinflammation in Mice". *Radiation Research*. **197** (4): 350–364. doi:10.1667/RADE-21-00006.1 (https://doi.org/10.1667%2FRADE-21-00006.1). PMID 34982167 (https://pubmed.ncbi.nlm.nih.gov/34982167). S2CID 245670201 (https://api.semanticscholar.org/CorpusID:245670201).
15. Liu J, Liu C, Yue J (January 2021). "Radiotherapy and the gut microbiome: facts and fiction" (https://www.ncbi.nlm.nih.gov/pmc/articles/PMC7805150). *Radiation Oncology*. **16** (1): 9. doi:10.1186/s13014-020-01735-9 (https://doi.org/10.1186%2Fs13014-020-01735-9). PMC 7805150 (https://www.ncbi.nlm.nih.gov/pmc/articles/PMC7805150). PMID 33436010 (https://pubmed.ncbi.nlm.nih.gov/33436010).
16. Yaneff J (2018-05-22). "How to Deal with Dysbiosis Microbial Imbalance in the Body" (https://www.doctorshealthpress.com/dysbiosis-microbial-imbalance/). *Doctors Health Press - Daily Free Health Articles and Natural Health Advice*. Retrieved 2022-12-10.
17. "Enemas" (https://www.doctorsbeyondmedicine.com/listing/enemas). *www.doctorsbeyondmedicine.com*. Retrieved 2022-12-10.
18. Zeng MY, Inohara N, Nuñez G (January 2017). "Mechanisms of inflammation-driven bacterial dysbiosis in the gut" (https://www.ncbi.nlm.nih.gov/pmc/articles/PMC5788567). *Mucosal Immunology*. **10** (1): 18–26. doi:10.1038/mi.2016.75 (https://doi.org/10.1038%2Fmi.2016.75). PMC 5788567 (https://www.ncbi.nlm.nih.gov/pmc/articles/PMC5788567). PMID 27554295 (https://pubmed.ncbi.nlm.nih.gov/27554295).
19. Pham TA, Lawley TD (February 2014). "Emerging insights on intestinal dysbiosis during bacterial infections" (https://www.ncbi.nlm.nih.gov/pmc/articles/PMC3969284). *Current Opinion in Microbiology*. **17** (100): 67–74. doi:10.1016/j.mib.2013.12.002 (https://doi.org/10.1016%2Fj.mib.2013.12.002). PMC 3969284 (https://www.ncbi.nlm.nih.gov/pmc/articles/PMC3969284). PMID 24581695 (https://pubmed.ncbi.nlm.nih.gov/24581695).
20. Buttó LF, Haller D (August 2016). "Dysbiosis in intestinal inflammation: Cause or consequence". *International Journal of Medical Microbiology*. **306** (5): 302–309. doi:10.1016/j.ijmm.2016.02.010 (https://doi.org/10.1016%2Fj.ijmm.2016.02.010). PMID 27012594 (https://pubmed.ncbi.nlm.nih.gov/27012594).
21. "Gastrointestinal tract" (https://en.wikipedia.org/w/index.php?title=Gastrointestinal_tract&oldid=1123080520), *Wikipedia*, 2022-11-21, retrieved 2022-11-28
22. "Small intestinal bacterial overgrowth" (https://en.wikipedia.org/w/index.php?title=Small_intestinal_bacterial_overgrowth&oldid=1091451125), *Wikipedia*, 2022-06-04, retrieved 2022-11-28
23. "Small intestinal bacterial overgrowth (SIBO) - Symptoms and causes" (https://www.mayoclinic.org/diseases-conditions/small-intestinal-bacterial-overgrowth/symptoms-causes/syc-20370168). *Mayo Clinic*. Retrieved 2022-11-28.
24. Singh R, Mullin GE (June 2017). "A Wasting Syndrome and Malnutrition Caused by Small Intestine Fungal Overgrowth: Case Report and Review of the Literature" (https://www.ncbi.nlm.nih.gov/pmc/articles/PMC6419785). *Integrative Medicine*. **16** (3): 48–51. PMC 6419785 (https://www.ncbi.nlm.nih.gov/pmc/articles/PMC6419785). PMID 30881247 (https://pubmed.ncbi.nlm.nih.gov/30881247).
25. Erdogan A, Rao SS (April 2015). "Small intestinal fungal overgrowth". *Current Gastroenterology Reports*. **17** (4): 16. doi:10.1007/s11894-015-0436-2 (https://doi.org/10.1007%2Fs11894-015-0436-2). PMID 25786900 (https://pubmed.ncbi.nlm.nih.gov/25786900). S2CID 3098136 (https://api.semanticscholar.org/CorpusID:3098136).

26. Carding S, Verbeke K, Vipond DT, Corfe BM, Owen LJ (February 2015). "Dysbiosis of the gut microbiota in disease" (https://www.ncbi.nlm.nih.gov/pmc/articles/PMC4315779). *Microbial Ecology in Health and Disease*. **26**: 26191. doi:10.3402/mehd.v26.26191 (https://doi.org/10.3402%2Fmehd.v26.26191). PMC 4315779 (https://www.ncbi.nlm.nih.gov/pmc/articles/PMC4315779). PMID 25651997 (https://pubmed.ncbi.nlm.nih.gov/25651997).
27. Tomasello G, Mazzola M, Leone A, Sinagra E, Zummo G, Farina F, et al. (December 2016). "Nutrition, oxidative stress and intestinal dysbiosis: Influence of diet on gut microbiota in inflammatory bowel diseases" (https://doi.org/10.5507%2Fbp.2016.052). *Biomedical Papers of the Medical Faculty of the University Palacky, Olomouc, Czechoslovakia*. **160** (4): 461–466. doi:10.5507/bp.2016.052 (https://doi.org/10.5507%2Fbp.2016.052). PMID 27812084 (https://pubmed.ncbi.nlm.nih.gov/27812084).
28. Koliarakis I, Messaritakis I, Nikolouzakis TK, Hamilos G, Souglakos J, Tsiaoussis J (August 2019). "Oral Bacteria and Intestinal Dysbiosis in Colorectal Cancer" (https://www.ncbi.nlm.nih.gov/pmc/articles/PMC6747549). *International Journal of Molecular Sciences*. **20** (17): 4146. doi:10.3390/ijms20174146 (https://doi.org/10.3390%2Fijms20174146). PMC 6747549 (https://www.ncbi.nlm.nih.gov/pmc/articles/PMC6747549). PMID 31450675 (https://pubmed.ncbi.nlm.nih.gov/31450675).
29. Chimenos-Küstner E, Giovannoni ML, Schemel-Suárez M (October 2017). "Dysbiosis as a determinant factor of systemic and oral pathology: importance of microbiome". *Medicina Clinica*. **149** (7): 305–309. doi:10.1016/j.medcli.2017.05.036 (https://doi.org/10.1016%2Fj.medcli.2017.05.036). hdl:2445/116548 (https://hdl.handle.net/2445%2F116548). PMID 28669517 (https://pubmed.ncbi.nlm.nih.gov/28669517).
30. Grice EA, Segre JA (April 2011). "The skin microbiome" (https://www.ncbi.nlm.nih.gov/pmc/articles/PMC3535073). *Nature Reviews. Microbiology*. **9** (4): 244–253. doi:10.1038/nrmicro2537 (https://doi.org/10.1038%2Fnrmicro2537). PMC 3535073 (https://www.ncbi.nlm.nih.gov/pmc/articles/PMC3535073). PMID 21407241 (https://pubmed.ncbi.nlm.nih.gov/21407241).
31. Holland KT, Cunliffe WJ, Roberts CD (June 1977). "Acne vulgaris: an investigation into the number of anaerobic diphtheroids and members of the Micrococcaceae in normal and acne skin". *The British Journal of Dermatology*. **96** (6): 623–626. doi:10.1111/j.1365-2133.1977.tb05206.x (https://doi.org/10.1111%2Fj.1365-2133.1977.tb05206.x). PMID 141301 (https://pubmed.ncbi.nlm.nih.gov/141301). S2CID 37507292 (https://api.semanticscholar.org/CorpusID:37507292).
32. Baker BS (April 2006). "The role of microorganisms in atopic dermatitis" (https://www.ncbi.nlm.nih.gov/pmc/articles/PMC1809642). *Clinical and Experimental Immunology*. **144** (1): 1–9. doi:10.1111/j.1365-2249.2005.02980.x (https://doi.org/10.1111%2Fj.1365-2249.2005.02980.x). PMC 1809642 (https://www.ncbi.nlm.nih.gov/pmc/articles/PMC1809642). PMID 16542358 (https://pubmed.ncbi.nlm.nih.gov/16542358).
33. Paulino LC, Tseng CH, Strober BE, Blaser MJ (August 2006). "Molecular analysis of fungal microbiota in samples from healthy human skin and psoriatic lesions" (https://www.ncbi.nlm.nih.gov/pmc/articles/PMC1594634). *Journal of Clinical Microbiology*. **44** (8): 2933–2941. doi:10.1128/JCM.00785-06 (https://doi.org/10.1128%2FJCM.00785-06). PMC 1594634 (https://www.ncbi.nlm.nih.gov/pmc/articles/PMC1594634). PMID 16891514 (https://pubmed.ncbi.nlm.nih.gov/16891514).
34. Grice EA (June 2014). "The skin microbiome: potential for novel diagnostic and therapeutic approaches to cutaneous disease" (https://www.ncbi.nlm.nih.gov/pmc/articles/PMC4425451). *Seminars in Cutaneous Medicine and Surgery*. **33** (2): 98–103. doi:10.12788/j.sder.0087 (https://doi.org/10.12788%2Fj.sder.0087). PMC 4425451 (https://www.ncbi.nlm.nih.gov/pmc/articles/PMC4425451). PMID 25085669 (https://pubmed.ncbi.nlm.nih.gov/25085669).
35. Vary JC, O'Connor KM (May 2014). "Common dermatologic conditions". *The Medical Clinics of North America*. **98** (3): 445–485. doi:10.1016/j.mcna.2014.01.005 (https://doi.org/10.1016%2Fj.mcna.2014.01.005). PMID 24758956 (https://pubmed.ncbi.nlm.nih.gov/24758956).

36. Lewis FM, Bernstein KT, Aral SO (April 2017). "Vaginal Microbiome and Its Relationship to Behavior, Sexual Health, and Sexually Transmitted Diseases" (https://www.ncbi.nlm.nih.gov/pmc/articles/PMC6743080). *Obstetrics and Gynecology*. **129** (4): 643–654. doi:10.1097/AOG.0000000000001932 (https://doi.org/10.1097%2FAOG.0000000000001932). PMC 6743080 (https://www.ncbi.nlm.nih.gov/pmc/articles/PMC6743080). PMID 28277350 (https://pubmed.ncbi.nlm.nih.gov/28277350).
37. Vangay P, Ward T, Gerber JS, Knights D (May 2015). "Antibiotics, pediatric dysbiosis, and disease" (https://www.ncbi.nlm.nih.gov/pmc/articles/PMC5555213). *Cell Host & Microbe*. **17** (5): 553–564. doi:10.1016/j.chom.2015.04.006 (https://doi.org/10.1016%2Fj.chom.2015.04.006). PMC 5555213 (https://www.ncbi.nlm.nih.gov/pmc/articles/PMC5555213). PMID 25974298 (https://pubmed.ncbi.nlm.nih.gov/25974298).
38. Thompson KG, Rainer BM, Antonescu C, Florea L, Mongodin EF, Kang S, Chien AL (February 2020). "Minocycline and Its Impact on Microbial Dysbiosis in the Skin and Gastrointestinal Tract of Acne Patients" (https://www.ncbi.nlm.nih.gov/pmc/articles/PMC7992645). *Annals of Dermatology*. **32** (1): 21–30. doi:10.5021/ad.2020.32.1.21 (https://doi.org/10.5021%2Fad.2020.32.1.21). PMC 7992645 (https://www.ncbi.nlm.nih.gov/pmc/articles/PMC7992645). PMID 33911705 (https://pubmed.ncbi.nlm.nih.gov/33911705).
39. Rogers GB, Keating DJ, Young RL, Wong ML, Licinio J, Wesselingh S (June 2016). "From gut dysbiosis to altered brain function and mental illness: mechanisms and pathways" (https://www.ncbi.nlm.nih.gov/pmc/articles/PMC4879184). *Molecular Psychiatry*. **21** (6): 738–48. doi:10.1038/mp.2016.50 (https://doi.org/10.1038%2Fmp.2016.50). PMC 4879184 (https://www.ncbi.nlm.nih.gov/pmc/articles/PMC4879184). PMID 27090305 (https://pubmed.ncbi.nlm.nih.gov/27090305).
40. Koszewicz M, Jaroch J, Brzecka A, Ejma M, Budrewicz S, Mikhaleva LM, et al. (February 2021). "Dysbiosis is one of the risk factor for stroke and cognitive impairment and potential target for treatment". *Pharmacological Research*. **164**: 105277. doi:10.1016/j.phrs.2020.105277 (https://doi.org/10.1016%2Fj.phrs.2020.105277). PMID 33166735 (https://pubmed.ncbi.nlm.nih.gov/33166735). S2CID 226296498 (https://api.semanticscholar.org/CorpusID:226296498).
41. Kigerl KA, Hall JC, Wang L, Mo X, Yu Z, Popovich PG (November 2016). "Gut dysbiosis impairs recovery after spinal cord injury" (https://www.ncbi.nlm.nih.gov/pmc/articles/PMC5110012). *The Journal of Experimental Medicine*. **213** (12): 2603–2620. doi:10.1084/jem.20151345 (https://doi.org/10.1084%2Fjem.20151345). PMC 5110012 (https://www.ncbi.nlm.nih.gov/pmc/articles/PMC5110012). PMID 27810921 (https://pubmed.ncbi.nlm.nih.gov/27810921).
42. DeGruttola AK, Low D, Mizoguchi A, Mizoguchi E (May 2016). "Current Understanding of Dysbiosis in Disease in Human and Animal Models" (https://www.ncbi.nlm.nih.gov/pmc/articles/PMC4838534). *Inflammatory Bowel Diseases*. **22** (5): 1137–50. doi:10.1097/MIB.0000000000000750 (https://doi.org/10.1097%2FMIB.0000000000000750). PMC 4838534 (https://www.ncbi.nlm.nih.gov/pmc/articles/PMC4838534). PMID 27070911 (https://pubmed.ncbi.nlm.nih.gov/27070911).
43. Sheflin AM, Whitney AK, Weir TL (October 2014). "Cancer-promoting effects of microbial dysbiosis" (https://www.ncbi.nlm.nih.gov/pmc/articles/PMC4180221). *Current Oncology Reports*. **16** (10): 406. doi:10.1007/s11912-014-0406-0 (https://doi.org/10.1007%2Fs11912-014-0406-0). PMC 4180221 (https://www.ncbi.nlm.nih.gov/pmc/articles/PMC4180221). PMID 25123079 (https://pubmed.ncbi.nlm.nih.gov/25123079).
44. Nath SG, Raveendran R (July 2013). "Microbial dysbiosis in periodontitis" (https://www.ncbi.nlm.nih.gov/pmc/articles/PMC3800425). *Journal of Indian Society of Periodontology*. **17** (4): 543–5. doi:10.4103/0972-124X.118334 (https://doi.org/10.4103%2F0972-124X.118334). PMC 3800425 (https://www.ncbi.nlm.nih.gov/pmc/articles/PMC3800425). PMID 24174742 (https://pubmed.ncbi.nlm.nih.gov/24174742).

45. Marteau P (2009). "Bacterial flora in inflammatory bowel disease". *Digestive Diseases*. **27** (Suppl 1): 99–103. doi:10.1159/000268128 (https://doi.org/10.1159%2F000268128). PMID 20203504 (https://pubmed.ncbi.nlm.nih.gov/20203504). S2CID 40843845 (https://api.semanticscholar.org/CorpusID:40843845).
46. Lepage P, Leclerc MC, Joossens M, Mondot S, Blottière HM, Raes J, et al. (January 2013). "A metagenomic insight into our gut's microbiome". *Gut*. **62** (1): 146–58. doi:10.1136/gutjnl-2011-301805 (https://doi.org/10.1136%2Fgutjnl-2011-301805). PMID 22525886 (https://pubmed.ncbi.nlm.nih.gov/22525886). S2CID 206956549 (https://api.semanticscholar.org/CorpusID:206956549).
47. Lakhan SE, Kirchgessner A (October 2010). "Gut inflammation in chronic fatigue syndrome" (https://www.ncbi.nlm.nih.gov/pmc/articles/PMC2964729). *Nutrition & Metabolism*. **7**: 79. doi:10.1186/1743-7075-7-79 (https://doi.org/10.1186%2F1743-7075-7-79). PMC 2964729 (https://www.ncbi.nlm.nih.gov/pmc/articles/PMC2964729). PMID 20939923 (https://pubmed.ncbi.nlm.nih.gov/20939923).
48. Turnbaugh PJ, Ley RE, Mahowald MA, Magrini V, Mardis ER, Gordon JI (December 2006). "An obesity-associated gut microbiome with increased capacity for energy harvest". *Nature*. **444** (7122): 1027–31. Bibcode:2006Natur.444.1027T (https://ui.adsabs.harvard.edu/abs/2006Natur.444.1027T). doi:10.1038/nature05414 (https://doi.org/10.1038%2Fnature05414). PMID 17183312 (https://pubmed.ncbi.nlm.nih.gov/17183312). S2CID 4400297 (https://api.semanticscholar.org/CorpusID:4400297).
49. Turnbaugh PJ, Hamady M, Yatsunenko T, Cantarel BL, Duncan A, Ley RE, et al. (January 2009). "A core gut microbiome in obese and lean twins" (https://www.ncbi.nlm.nih.gov/pmc/articles/PMC2677729). *Nature*. **457** (7228): 480–4. Bibcode:2009Natur.457..480T (https://ui.adsabs.harvard.edu/abs/2009Natur.457..480T). doi:10.1038/nature07540 (https://doi.org/10.1038%2Fnature07540). PMC 2677729 (https://www.ncbi.nlm.nih.gov/pmc/articles/PMC2677729). PMID 19043404 (https://pubmed.ncbi.nlm.nih.gov/19043404).
50. Castellarin M, Warren RL, Freeman JD, Dreolini L, Krzywinski M, Strauss J, et al. (February 2012). "Fusobacterium nucleatum infection is prevalent in human colorectal carcinoma" (https://www.ncbi.nlm.nih.gov/pmc/articles/PMC3266037). *Genome Research*. **22** (2): 299–306. doi:10.1101/gr.126516.111 (https://doi.org/10.1101%2Fgr.126516.111). PMC 3266037 (https://www.ncbi.nlm.nih.gov/pmc/articles/PMC3266037). PMID 22009989 (https://pubmed.ncbi.nlm.nih.gov/22009989).
51. Kostic AD, Gevers D, Pedamallu CS, Michaud M, Duke F, Earl AM, et al. (February 2012). "Genomic analysis identifies association of Fusobacterium with colorectal carcinoma" (https://www.ncbi.nlm.nih.gov/pmc/articles/PMC3266036). *Genome Research*. **22** (2): 292–8. doi:10.1101/gr.126573.111 (https://doi.org/10.1101%2Fgr.126573.111). PMC 3266036 (https://www.ncbi.nlm.nih.gov/pmc/articles/PMC3266036). PMID 22009990 (https://pubmed.ncbi.nlm.nih.gov/22009990).
52. Africa CW, Nel J, Stemmet M (July 2014). "Anaerobes and bacterial vaginosis in pregnancy: virulence factors contributing to vaginal colonisation" (https://www.ncbi.nlm.nih.gov/pmc/articles/PMC4113856). *International Journal of Environmental Research and Public Health*. **11** (7): 6979–7000. doi:10.3390/ijerph110706979 (https://doi.org/10.3390%2Fijerph110706979). PMC 4113856 (https://www.ncbi.nlm.nih.gov/pmc/articles/PMC4113856). PMID 25014248 (https://pubmed.ncbi.nlm.nih.gov/25014248).
53. Mazmanian SK (April 2008). "Capsular polysaccharides of symbiotic bacteria modulate immune responses during experimental colitis". *Journal of Pediatric Gastroenterology and Nutrition*. **46** (Suppl 1): E11–2. doi:10.1097/01.mpg.0000313824.70971.a7 (https://doi.org/10.1097%2F01.mpg.0000313824.70971.a7). PMID 18354314 (https://pubmed.ncbi.nlm.nih.gov/18354314).
54. Knoop FC, Owens M, Crocker IC (July 1993). "Clostridium difficile: clinical disease and diagnosis" (https://www.ncbi.nlm.nih.gov/pmc/articles/PMC358285). *Clinical Microbiology Reviews*. **6** (3): 251–65. doi:10.1128/CMR.6.3.251 (https://doi.org/10.1128%2FCMR.6.3.251). PMC 358285 (https://www.ncbi.nlm.nih.gov/pmc/articles/PMC358285). PMID 8358706 (https://pubmed.ncbi.nlm.nih.gov/8358706).

55. Surawicz CM, Brandt LJ, Binion DG, Ananthakrishnan AN, Curry SR, Gilligan PH, et al. (April 2013). "Guidelines for diagnosis, treatment, and prevention of Clostridium difficile infections". *The American Journal of Gastroenterology*. **108** (4): 478–98, quiz 499. doi:10.1038/ajg.2013.4 (https://doi.org/10.1038%2Fajg.2013.4). PMID 23439232 (https://pubmed.ncbi.nlm.nih.gov/23439232). S2CID 54629762 (https://api.semanticscholar.org/CorpusID:54629762).
56. "Clostridium difficile Infection Information for Patients" (https://www.cdc.gov/hai/organisms/cdiff/cdiff-patient.html). Centers for Disease Control. Retrieved 2018-06-27.
57. Surawicz CM, McFarland LV, Elmer G, Chinn J (October 1989). "Treatment of recurrent Clostridium difficile colitis with vancomycin and Saccharomyces boulardii". *The American Journal of Gastroenterology*. **84** (10): 1285–7. PMID 2679049 (https://pubmed.ncbi.nlm.nih.gov/2679049).
58. "Periodontitis - Symptoms and causes" (https://www.mayoclinic.org/diseases-conditions/periodontitis/symptoms-causes/syc-20354473). *Mayo Clinic*. Retrieved 2018-06-27.
59. Sharara AI, Aoun E, Abdul-Baki H, Mounzer R, Sidani S, Elhajj I (February 2006). "A randomized double-blind placebo-controlled trial of rifaximin in patients with abdominal bloating and flatulence". *The American Journal of Gastroenterology*. **101** (2): 326–33. doi:10.1111/j.1572-0241.2006.00458.x (https://doi.org/10.1111%2Fj.1572-0241.2006.00458.x). PMID 16454838 (https://pubmed.ncbi.nlm.nih.gov/16454838). S2CID 23817453 (https://api.semanticscholar.org/CorpusID:23817453).
60. Weiss GA, Hennet T (August 2017). "Mechanisms and consequences of intestinal dysbiosis" (https://www.zora.uzh.ch/id/eprint/144519/1/144519.pdf) (PDF). *Cellular and Molecular Life Sciences*. **74** (16): 2959–2977. doi:10.1007/s00018-017-2509-x (https://doi.org/10.1007%2Fs00018-017-2509-x). PMID 28352996 (https://pubmed.ncbi.nlm.nih.gov/28352996). S2CID 22334485 (https://api.semanticscholar.org/CorpusID:22334485).
61. Gianotti RJ, Moss AC (November 2016). "The Use and Efficacy of Fecal Microbiota Transplantation for Refractory Clostridium difficile in Patients with Inflammatory Bowel Disease" (https://doi.org/10.1097%2Fmib.0000000000000950). *Inflammatory Bowel Diseases*. **22** (11): 2704–2710. doi:10.1097/mib.0000000000000950 (https://doi.org/10.1097%2Fmib.0000000000000950). PMID 27755271 (https://pubmed.ncbi.nlm.nih.gov/27755271).
62. Giau VV, Lee H, An SS, Hulme J (June 2019). "Recent advances in the treatment of *C. difficile* using biotherapeutic agents" (https://www.ncbi.nlm.nih.gov/pmc/articles/PMC6579870). *Infection and Drug Resistance*. **12**: 1597–1615. doi:10.2147/IDR.S207572 (https://doi.org/10.2147%2FIDR.S207572). PMC 6579870 (https://www.ncbi.nlm.nih.gov/pmc/articles/PMC6579870). PMID 31354309 (https://pubmed.ncbi.nlm.nih.gov/31354309).
63. "What is FMT? — The Fecal Transplant Foundation" (http://thefecaltransplantfoundation.org/what-is-fecal-transplant/). *thefecaltransplantfoundation.org*. Retrieved 2018-06-27.
64. Smith MB, Kelly C, Alm EJ (February 2014). "Policy: How to regulate faecal transplants" (https://doi.org/10.1038%2F506290a). *Nature*. **506** (7488): 290–291. doi:10.1038/506290a (https://doi.org/10.1038%2F506290a). PMID 24558658 (https://pubmed.ncbi.nlm.nih.gov/24558658).
65. "Fecal Microbiota Transplantation" (https://www.idsociety.org/public-health/emerging-clinical-issues/emerging-clinical-issues/fecal-microbiota-transplantation/). *Emerging Infections Network*. 2022.
66. Chinna Meyyappan A, Forth E, Wallace CJ, Milev R (June 2020). "Effect of fecal microbiota transplant on symptoms of psychiatric disorders: a systematic review" (https://www.ncbi.nlm.nih.gov/pmc/articles/PMC7294648). *BMC Psychiatry*. **20** (1): 299. doi:10.1186/s12888-020-02654-5 (https://doi.org/10.1186%2Fs12888-020-02654-5). PMC 7294648 (https://www.ncbi.nlm.nih.gov/pmc/articles/PMC7294648). PMID 32539741 (https://pubmed.ncbi.nlm.nih.gov/32539741).

67. Dupont HL (October 2013). "Diagnosis and management of Clostridium difficile infection". *Clinical Gastroenterology and Hepatology*. **11** (10): 1216–23, quiz e73. doi:10.1016/j.cgh.2013.03.016 (https://doi.org/10.1016%2Fj.cgh.2013.03.016). PMID 23542332 (https://pubmed.ncbi.nlm.nih.gov/23542332).
68. Food and Agriculture Organization of the United Nations; World Health Organization. Health and Nutritional Properties of Probiotics in Food Including Powder Milk with Live Lactic Acid Bacteria.Córdoba, Argentina: Food and Agriculture Organization of the United Nations, World Health Organization; 2001.
69. Drakes M, Blanchard T, Czinn S (June 2004). "Bacterial probiotic modulation of dendritic cells" (https://www.ncbi.nlm.nih.gov/pmc/articles/PMC415669). *Infection and Immunity*. **72** (6): 3299–309. doi:10.1128/IAI.72.6.3299-3309.2004 (https://doi.org/10.1128%2FIAI.72.6.3299-3309.2004). PMC 415669 (https://www.ncbi.nlm.nih.gov/pmc/articles/PMC415669). PMID 15155633 (https://pubmed.ncbi.nlm.nih.gov/15155633).
70. Kim SO, Sheikh HI, Ha SD, Martins A, Reid G (December 2006). "G-CSF-mediated inhibition of JNK is a key mechanism for Lactobacillus rhamnosus-induced suppression of TNF production in macrophages" (https://doi.org/10.1111%2Fj.1462-5822.2006.00763.x). *Cellular Microbiology*. **8** (12): 1958–71. doi:10.1111/j.1462-5822.2006.00763.x (https://doi.org/10.1111%2Fj.1462-5822.2006.00763.x). PMID 16889627 (https://pubmed.ncbi.nlm.nih.gov/16889627).
71. Kendall MM, Sperandio V (January 2007). "Quorum sensing by enteric pathogens". *Current Opinion in Gastroenterology*. **23** (1): 10–5. doi:10.1097/MOG.0b013e3280118289 (https://doi.org/10.1097%2FMOG.0b013e3280118289). PMID 17133078 (https://pubmed.ncbi.nlm.nih.gov/17133078). S2CID 45681413 (https://api.semanticscholar.org/CorpusID:45681413).
72. Wischmeyer PE, McDonald D, Knight R (August 2016). "Role of the microbiome, probiotics, and 'dysbiosis therapy' in critical illness" (https://www.ncbi.nlm.nih.gov/pmc/articles/PMC5065053). *Current Opinion in Critical Care*. **22** (4): 347–53. doi:10.1097/MCC.0000000000000321 (https://doi.org/10.1097%2FMCC.0000000000000321). PMC 5065053 (https://www.ncbi.nlm.nih.gov/pmc/articles/PMC5065053). PMID 27327243 (https://pubmed.ncbi.nlm.nih.gov/27327243).
73. Kim SK, Guevarra RB, Kim YT, Kwon J, Kim H, Cho JH, et al. (September 2019). "Role of Probiotics in Human Gut Microbiome-Associated Diseases" (https://doi.org/10.4014%2Fjmb.1906.06064). *Journal of Microbiology and Biotechnology*. **29** (9): 1335–1340. doi:10.4014/jmb.1906.06064 (https://doi.org/10.4014%2Fjmb.1906.06064). PMID 31434172 (https://pubmed.ncbi.nlm.nih.gov/31434172).
74. Dempsey E, Corr SC (2022). "Lactobacillus spp. for Gastrointestinal Health: Current and Future Perspectives" (https://www.ncbi.nlm.nih.gov/pmc/articles/PMC9019120). *Frontiers in Immunology*. **13**: 840245. doi:10.3389/fimmu.2022.840245 (https://doi.org/10.3389%2Ffimmu.2022.840245). PMC 9019120 (https://www.ncbi.nlm.nih.gov/pmc/articles/PMC9019120). PMID 35464397 (https://pubmed.ncbi.nlm.nih.gov/35464397).
75. De Simone C (16 January 2018). "P884 No shared mechanisms among "old" and "new" VSL#3: Implications for claims and guidelines" (https://academic.oup.com/ecco-jcc/article/12/supplement_1/S564/4808250). *Journal of Crohn's and Colitis*. **12** (supplement_1): S564–S565. doi:10.1093/ecco-jcc/jjx180.1011 (https://doi.org/10.1093%2Fecco-jcc%2Fjjx180.1011).
76. "ExeGi Wins Court Ruling Against VSL#3®*, Permanent Injunction Blocks False Advertising to Doctors, Consumers" (https://www.biospace.com/article/releases/exegi-wins-court-ruling-against-vsl-3-permanent-injunction-blocks-false-advertising-to-doctors-consumers/). *BioSpace*. 25 June 2019.
77. Gray N (17 April 2018). "Trademarks, branding and product names: An 'unregulated' part of the probiotic market?" (https://www.nutraingredients.com/Article/2018/04/17/Trademarks-branding-and-product-names-An-unregulated-part-of-the-probiotic-market). *NutraIngredients.com*.

External links

- Intestinal dysbiosis (http://www.ei-resource.org/articles/candida-and-gut-dysbiosis-articles/intestinal-dysbiosis-and-the-causes-of-disease/)

Retrieved from "https://en.wikipedia.org/w/index.php?title=Dysbiosis&oldid=1127568778"

This page was last edited on 15 December 2022, at 13:05 (UTC).

Text is available under the Creative Commons Attribution-ShareAlike License 3.0; additional terms may apply. By using this site, you agree to the Terms of Use and Privacy Policy. Wikipedia® is a registered trademark of the Wikimedia Foundation, Inc., a non-profit organization.
