# Supplementary material for: Assessment of medical information on irritable bowel syndrome information in Wikipedia and Baidu Encyclopedia: comparative study
Source: PeerJ. 2024 May 24;12:e17264. doi: 10.7717/peerj.17264 (PMC11129691; doi:10.7717/peerj.17264)
Supplement: Data S1 [file peerj-12-17264-s001.zip › σÄƒσoïμò░μì«/Wikipedia/Functional gastrointestinal disorder - Wikipedia.pdf]

WIKIPEDIA

Functional gastrointestinal disorder

Functional gastrointestinal disorders (**FGID**), also known as **disorders of gut–brain interaction**, include a number of separate idiopathic disorders which affect different parts of the gastrointestinal tract and involve visceral hypersensitivity and motility disturbances.[1]

Functional gastrointestinal disorder	
**Other names**	Disorders of gut–brain interaction
**Specialty**	Gastroenterology

Contents

Definition of functional gastrointestinal disorders/disorders of gut–brain interaction
Classification
Causes
- Early life factors
- Psychosocial factors
- Physiology
- Brain–gut axis

Epidemiology
Research
See also
References
External links

Definition of functional gastrointestinal disorders/disorders of gut-brain interaction

Using the Delphi method, the Rome Foundation and its board of directors, chairs and co-chairs of the ROME IV committees developed the current definition for disorders of gut-brain interaction.[2]

A group of disorders classified by GI symptoms related to any combination of:[2]

- Motility disturbance
- Visceral hypersensitivity
- Altered mucosal and immune function
- Altered gut microbiota

- Altered central nervous system (CNS) processing

Classification

Terms such as *functional colonic disease* (or *functional bowel disorder*) refer in medicine to a group of bowel disorders which are characterised by chronic abdominal complaints without a structural or biochemical cause that could explain symptoms. Other *functional* disorders relate to other aspects of the process of digestion.[1]

The consensus review process of meetings and publications organised by the Rome Foundation, known as the Rome process, has helped to define the functional gastrointestinal disorders.[3] Successively, the Rome I, Rome II, Rome III and Rome IV proposed consensual classification system and terminology, as recommended by the Rome Coordinating Committee. These now include classifications appropriate for adults, children and neonates/toddlers.[1]

The current ROME IV classification, published in 2016, is as follows:[1]

A. Esophageal disorders

- A1. Functional chest pain
- A2. Functional heartburn
- A3. Reflux hypersensitivity
- A4. Globus
- A5. Functional dysphagia

B. Gastroduodenal disorders

- B1. Functional dyspepsia
  - B1a. Postprandial distress syndrome (PDS)
  - B1b. Epigastric pain syndrome (EPS)
- B2. Belching disorders
  - B2a. Excessive supragastric belching
  - B2b. Excessive gastric belching
- B3. Nausea and vomiting disorders
  - B3a. Chronic nausea vomiting syndrome (CNVS)
  - B3b. Cyclic vomiting syndrome (CVS)
  - B3c. Cannabinoid hyperemesis syndrome (CHS)
- B4. Rumination syndrome

C. Bowel disorders

- C1. Irritable bowel syndrome (IBS)
  - IBS with predominant constipation (IBS-C)
  - IBS with predominant diarrhea (IBS-D)
  - IBS with mixed bowel habits (IBS-M)

  - IBS unclassified (IBS-U)
- C2. Functional constipation
- C3. Functional diarrhea
- C4. Functional abdominal bloating/distension
- C5. Unspecified functional bowel disorder
- C6. Opioid-induced constipation

D. Centrally mediated disorders of gastrointestinal pain

- D1. Centrally mediated abdominal pain syndrome (CAPS)
- D2. Narcotic bowel syndrome (NBS)/ Opioid-induced GI hyperalgesia

E. Gallbladder and sphincter of Oddi disorders

- E1. Biliary pain
  - E1a. Functional gallbladder disorder
  - E1b. Functional biliary sphincter of Oddi disorder
- E2. Functional pancreatic sphincter of Oddi disorder

F. Anorectal disorders

- F1. Fecal incontinence
- F2. Functional anorectal pain
  - F2a. Levator ani syndrome
  - F2b. Unspecified functional anorectal pain
  - F2c. Proctalgia fugax
- F3. Functional defecation disorders
  - F3a. Inadequate defecatory propulsion
  - F3b. Dyssynergic defecation

G. Childhood functional GI disorders: Neonate/Toddler

- G1. Infant regurgitation
- G2. Rumination syndrome
- G3. Cyclic vomiting syndrome (CVS)
- G4. Infant colic
- G5. Functional diarrhea
- G6. Infant dyschezia
- G7. Functional constipation

H. Childhood functional GI disorders: Child/Adolescent

- H1. Functional nausea and vomiting disorders

    - H1a. Cyclic vomiting syndrome (CVS)
    - H1b. Functional nausea and functional vomiting
        - H1b1. Functional nausea
        - H1b2. Functional vomiting
    - H1c. Rumination syndrome
    - H1d. Aerophagia
- H2. Functional abdominal pain disorders
    - H2a. Functional dyspepsia
        - H2a1. Postprandial distress syndrome
        - H2a2. Epigastric pain syndrome
    - H2b. Irritable bowel syndrome (IBS)
    - H2c. Abdominal migraine
    - H2d. Functional abdominal pain - NOS
- H3. Functional defecation disorders
    - H3a. Functional constipation
    - H3b. Nonretentive fecal incontinence

Causes

FGIDs share in common any of several physiological features including increased motor reactivity, enhanced visceral hypersensitivity, altered mucosal immune and inflammatory function (associated with bacterial dysbiosis), and altered central nervous system and enteric nervous system (CNS-ENS) regulation.

The pathophysiology of FGID has been best conceptualized using biopsychosocial model help to explain the relationships between an individual factors in their early life that in turn can influence their psychosocial factor and physiological functioning. This model also shows the complex interactions between these factors through the brain-gut axis.[4][5][6][7][8] These factors affect how FGID manifest in terms of symptoms but also affect the clinical outcome. These factors are interconnected and the influences on these factors are bidirectional and mutually interactive.

Early life factors

Early life factors include genetic factors, psychophysiological and sociocultural factors, and environmental exposures.

- **Genetics** — Several polymorphisms and candidate genes may predispose individuals to develop FGID. These include alpha-2 adrenergic and 5-HT receptors; serotonin and norepinephrine transporters (SERT, NET); inflammatory markers interleukin-(IL)10, tumor necrosis factor-(TNF) alpha, and TNF super family member 15 (TNF-SF15); intracellular cell signaling (G proteins); and ion channels (SCN5A).[9] However, the expression of a FGID requires the influence of additional environmental exposures such as infection, illness modeling and other factors.

- **Psychophysiological factors** may affect the expression of these genes, thus leading to symptoms production associated with FGID.[10]
- **Sociocultural factors and family interactions** have been shown to shape later reporting of symptoms, the development of FGIDs, and health care seeking. The expression of pain varies across cultures as well including denial of symptoms to dramatic expression.[11]
- **Environmental exposures** — Prior studies have shown the effect of environmental exposures in relation to the development of FGIDs. Environmental exposures such as childhood salmonella infection can be a risk factor for IBS in adulthood.[12]

Psychosocial factors

Psychosocial factors influence the functioning of the GI tract through the brain-gut axis (motility, sensitivity, barrier function). They also affect experience and behavior, treatment selection and the clinical outcome. Psychological stress or one's emotional response to stress exacerbates gastrointestinal symptoms and may contribute to FGID development.[13][14]

Physiology

The physiology of FGID is characterized by abnormal motility, visceral hypersensitivity as well as dysregulation of the immune system and barrier function of the GI tract as well as inflammatory changes.

- **Abnormal motility**
  Studies have shown altered muscle contractility and tone, bowel compliance, and transit may contribute to many of the gastrointestinal symptoms of FGID which may include diarrhea, constipation, and vomiting.[15]
- **Visceral hypersensitivity**
  In FGID there is poor association of pain with GI motility in many functional GI disorders. These patient often have a lower pain threshold with balloon distension of the bowel (visceral hyperalgesia), or they have increased sensitivity even to normal intestinal function; Visceral hypersensitivity may be amplified in patients with FGIDs.[16][17]
- **Immune dysregulation, inflammation, and barrier dysfunction**
  Studies on postinfectious IBS have shown that factors such as mucosal membrane permeability, the intestinal flora, and altered mucosal immune function. Ultimately leading to visceral hypersensitivity. Factors contributing to this occurrence include genetics, psychological stress, and altered receptor sensitivity at the gut mucosa and myenteric plexus, which are enabled by mucosal immune dysfunction.[18][19]
- **Microbiome**
  There has been increased attention to the role of bacteria and the microbiome in overall health and disease. There is evidence for a group of microorganisms which play a role in the brain–gut axis.[20] Studies have revealed that the bacterial composition of the gastrointestinal tract in IBS patient differs from healthy individuals (e.g., increased Firmicutes and reduced Bacteroidetes and Bifidobacteria)[21] However, further research is needed to determine the role of the microbiome in FGIDs.
- **Food and diet**
  The types of food consumed and diet consumed plays a role in the manifestation of

FGID[22] and also their relationship to intestinal microbiota.[23] Studies have shown that specific changes in diet (e.g., low FODMAP—fermentable oligo-, di-, and monosaccharides and polyols, or gluten restriction in some patients) may help and reduce the symptom burden in FGID. However, no one diet has been shown to be recommended for all people.

Brain–gut axis

The brain-gut axis is the mechanism in which the psychosocial factors influence the GI tract and vice versa. There is communication between emotional and cognitive centers of the brain to the GI tract and vice versa.[24] Emotions have been shown to stimulate colon motor function and result in decreased colonic transit time, increased contractile activity, the induction of defecation, and symptoms of diarrhea.[4]

Epidemiology

Functional gastrointestinal disorders are very common. Globally, irritable bowel syndrome and functional dyspepsia alone may affect 16–26% of the population.[1][25]

Research

There is considerable research into the causes, diagnosis and treatments for FGIDs. Diet, microbiome, genetics, neuromuscular function and immunological response all interact.[1] A role for mast cell activation has been proposed as one of the factors.[26][27]

See also

- Allergy
- Food intolerance
- Functional indigestion
- Histamine intolerance

References

1. Drossman DA (2016). "Functional Gastrointestinal Disorders: History, Pathophysiology, Clinical Features and Rome IV" (https://www.ncbi.nlm.nih.gov/entrez/eutils/elink.fcgi?dbfrom=pubmed&tool=sumsearch.org/cite&retmode=ref&cmd=prlinks&id=27144617). *Gastroenterology*. **150**: 1262–1279. doi:10.1053/j.gastro.2016.02.032 (https://doi.org/10.1053%2Fj.gastro.2016.02.032). PMID 27144617 (https://pubmed.ncbi.nlm.nih.gov/27144617).
2. Drossman DA. The Rome IV Committees, editor. Functional Gastrointestinal Disorders and the Rome IV process. In: Drossman DA, Chang L, Chey WD, Kellow J, Tack J, Whitehead WE, editors. Rome IV functional gastrointestinal disorders: disorders of gut-brain interaction. I. Raleigh, NC: The Rome Foundation; 2016. pp. 1–32.
3. "Rome Foundation // Scoring Rome III Questionnaire using SAS" (http://www.romecriteria.org/rome_iii_sas/).
4. Drossman DA. The Rome IV Committees, editor. Functional Gastrointestinal Disorders and the Rome IV process. In: Drossman DA, Chang L, Chey WD, Kellow J, Tack J, Whitehead WE, editors. Rome IV functional gastrointestinal disorders: disorders of gut-brain interaction.I. Raleigh, NC: The Rome Foundation; 2016. pp 1–32.

5. Drossman DA. Biopsychosocial issues in gastroenterology. In: Feldman M, Friedman LS, and Brandt LJ ads. Sleisenger and Fordtran's gastrointestinal and liver disease. 10th ed. Philadelphia: Saunders Elsevier, 2015:349—362.
6. Mayer EA, Savage T, Shulman RJ. Brain–gut microbiome interactions and functional bowel disorders. Gastroenterology 2014;146:1500—1512.
7. Jones MP, Dilley JB, Crowell MD et al. Brain–gut connections in functional GI disorders: anatomic and physiological relationships. Neurogastroenterol Motel 2006; 18:91—103
8. Drossman DA. Presidential Address Address: Gastrointestinal Illness and the Biopsychosocial Model. Psychosomatic Medicine 1998;60:258—267
9. Saito YA, TAlley NJ. Genetics of irritable bowel syndrome. Am J Gastroenterol 2008;103:2100—2104.
10. Tran L, Chaloner A, Sawalha AH, et al. Importance of epigenetic mechanisms in visceral pain induced by chronic water avoidance stress. Psychoneuroendocrinology 2013;38:898—906
11. Zborowski M. Cultural components in responses to pain. J Social Issues 1952;8:16—30.
12. Cremon C, Stanghellini V, Pallotti F, et al. Salmonella gastroenteritis during childhood is a risk factor for irritable bowel syndrome in adulthood. Gastroenterology 2014:147:69—77
13. Drossman DA. The Rome IV Committees, editor. Functional Gastrointestinal Disorders and the Rome IV process. In: Drossman DA, Chang L, Chey WD, Kellow J, Tack J, Whitehead WE, editors. Rome IV functional gastrointestinal disorders: disorders of gut–brain interaction. I. Raleigh, NC: The Rome Foundation; 2016. pp 1—32.
14. Collins SM, Chang C, Mearin F. Postinfectious chronic gut dysfunction: from bench to bedside. Am J Gastroenterol Suppl 2012;1:2—8
15. Camilleri M. Peripheral mechanisms in irritable bowel syndrome. N Engl J Med 2012;367:1626—1635
16. Delgado–Aros S, Camilleri M. Visceral hypersensitivity. J Clin Gastronenterol 2005;39:S194–S203
17. Mayer EA, Gebhart GF. Basic and clinical aspects of visceral hyperalgesia. Gastroenterology 1994;107:271—293
18. Zhou Q, Zhang B, Verne GE. Intestinal membrane permeability and hypersensitivity in the irritable bowel syndrome. Pain 2009;146:41—46
19. Camilleri M, Madsen K, Spiller R, et al. Intestinal barrier function in health and gastrointestinal disease. Neurogastroenterol Motil 2012; 24:503—512
20. Mayer EA, Savage T, Shulman RJ. Brain–gut micro biome interactions and functional bowel disorders. Gastroenterology 2014;146:1500—1512.
21. Simrén M, Barbara G, Flint HJ, et al. Intestinal microbiota in functional bowel disorders: a Rome Foundation working team report. Gut 2013;1:159—176
22. Chey WD. The role of food in the functional gastrointestinal disorders: introduction to a manuscript series. Am J Gastroenterol 2013;108:694—697.
23. Rajilic–Stojanavic M, Jonkers DM, Salonen A, et al. Intestinal microbiota and diet in IBS: causes, consequences, or epiphenomena? Am J Gastroenterol 2015; 110:278—287.
24. Gaman A, Kuo B. Neuromodulatory processes of the brain–gut axis. Neuromodulation 2008; 11:249—259
25. Sperber AD, Drossman DA, Quigley EM (2012). "The global perspective on irritable bowel syndrome: a Rome Foundation–World Gastroenterology Organisation symposium". *Am. J. Gastroenterol.* **107** (11): 1602—9. doi:10.1038/ajg.2012.106 (https://doi.org/10.1038%2Fajg.2012.106). PMID 23160283 (https://pubmed.ncbi.nlm.nih.gov/23160283). S2CID 34208367 (https://api.semanticscholar.org/CorpusID:34208367).

26. Wouters MM, Vicario M, Santos J (2015). "The role of mast cells in functional GI disorders" (https://doi.org/10.1136%2Fgutjnl-2015-309151). *Gut*. **65** (1): 155–168. doi:10.1136/gutjnl-2015-309151 (https://doi.org/10.1136%2Fgutjnl-2015-309151). PMID 26194403 (https://pubmed.ncbi.nlm.nih.gov/26194403). "It is well established that mast cell activation can generate epithelial and neuro-muscular dysfunction and promote visceral hypersensitivity and altered motility patterns in FGIDs, postoperative ileus, food allergy and inflammatory bowel disease."
27. Bashashati, M; Moossavi, S; Cremon, C; Barbaro, MR; Moraveji, S; Talmon, G; Rezaei, N; Hughes, PA; Bian, ZX; Choi, CH; Lee, OY; Coëffier, M; Chang, L; Ohman, L; Schmulson, MJ; McCallum, RW; Simren, M; Sharkey, KA; Barbara, G (January 2018). "Colonic immune cells in irritable bowel syndrome: A systematic review and meta-analysis". *Neurogastroenterology & Motility*. **30** (1): e13192. doi:10.1111/nmo.13192 (https://doi.org/10.1111%2Fnmo.13192). PMID 28851005 (https://pubmed.ncbi.nlm.nih.gov/28851005). S2CID 33807711 (https://api.semanticscholar.org/CorpusID:33807711). "Mast cells and CD3+ T cells are increased in colonic biopsies of patients with IBS vs non-inflamed controls"

External links

Retrieved from "https://en.wikipedia.org/w/index.php?title=Functional_gastrointestinal_disorder&oldid=1128679123"

This page was last edited on 21 December 2022, at 11:08 (UTC).

Text is available under the Creative Commons Attribution-ShareAlike License 3.0; additional terms may apply. By using this site, you agree to the Terms of Use and Privacy Policy. Wikipedia® is a registered trademark of the Wikimedia Foundation, Inc., a non-profit organization.
