# Supplementary material for: Assessment of medical information on irritable bowel syndrome information in Wikipedia and Baidu Encyclopedia: comparative study
Source: PeerJ. 2024 May 24;12:e17264. doi: 10.7717/peerj.17264 (PMC11129691; doi:10.7717/peerj.17264)
Supplement: Data S1 [file peerj-12-17264-s001.zip › σÄƒσoïμò░μì«/Wikipedia/Gut Dysbiosis Promotes Preeclampsia by Regulating Macrophages and Trophoblasts(τoæτáöΘÇÜ-ablesci.com).pdf]

Circulation Research

ORIGINAL RESEARCH

Gut Dysbiosis Promotes Preeclampsia by Regulating Macrophages and Trophoblasts

Jiajia Jin,* Liaomei Gao,* Xiuli Zou,* Yun Zhang, Zhijian Zheng, Xinjie Zhang, Jiaxuan Li, Zhenyu Tian, Xiaowei Wang, Junfei Gu, Cheng Zhang, Tiejun Wu, Zhe Wang, Qunye Zhang*

BACKGROUND: Preeclampsia is one of the leading causes of maternal and perinatal morbidity and is characterized by hypertension, inflammation, and placental dysfunction. Gut microbiota plays key roles in inflammation and hypertension. However, its roles and mechanisms in preeclampsia have not been fully elucidated.

METHODS: 16S rRNA gene sequencing and targeted metabolomics were conducted on stool samples from 92 preeclamptic patients and 86 normal late-pregnant women. Then, fecal microbiota transplantation and in vitro and in vivo functional experiments were performed to explore the roles and mechanisms of gut microbiota in preeclampsia development.

RESULTS: We revealed the gut microbiota dysbiosis in preeclamptic patients, including significant reductions in short-chain fatty acid-producing bacteria and short-chain fatty acids. The gut microbiota of preeclamptic patients significantly exacerbated pathologies and symptoms of preeclamptic rats, whereas the gut microbiota of healthy pregnant women had significant protective effects. *Akkermansia muciniphila,* propionate, or butyrate significantly alleviated the symptoms of preeclamptic rats. Mechanistically, they significantly promoted autophagy and M2 polarization of macrophages in placental bed, thereby suppressing inflammation. Propionate also significantly promoted trophoblast invasion, thereby improved spiral arterial remodeling. Additionally, we identified a marker set consisting of *Akkermansia*, *Oscillibacter,* and short-chain fatty acids that could accurately diagnose preeclampsia.

CONCLUSIONS: Our study revealed that gut microbiota dysbiosis is an important etiology of preeclampsia. Gut microbiota and their active metabolites have great potential for the treatment and diagnosis of preeclampsia. Our findings enrich the gut–placenta axis theory and contribute to the development of microecological products for preeclampsia.

GRAPHIC ABSTRACT: A graphic abstract is available for this article.

Circulation Research FIRST PROOF ONLY

Key Words: dysbiosis ■ gut microbiota ■ hypertension ■ placenta ■ preeclampsia

Downloaded from http://ahajournals.org by on August 11, 2022

As a pregnancy-specific disease, preeclampsia is one of the leading causes of maternal and perinatal morbidity.[1] New-onset hypertension is the main characteristics of preeclampsia. Women with preeclampsia have a significantly increased risk of future cardiovascular disease and acute events.[2] Moreover, preeclampsia can affect the neurodevelopment of infants after birth.[3] However, there is still no effective treatment for preeclampsia other than terminating the pregnancy.

It is thought that inadequate spiral artery remodeling in early pregnancy causes preeclampsia. Many inflammatory and vasoactive factors play key roles in preeclampsia development.[4] However, innate immune abnormalities in the uteroplacental region caused by macrophages also are suggested to be the main etiology of preeclampsia. Nevertheless, the placenta, which is the source and target of multiple pathological stimuli, is central to the pathogenesis of preeclampsia, but the

Correspondence to: Qunye Zhang, Wenhua Xi Rd 107#, Jinan 250012, China, Email wz.zhangqy@sdu.edu.cn or Zhe Wang, Jing 5 Wei 7 Rd 324#, Jinan 250021, China, Email wangzhe.zqy@email.sdu.edu.cn or Tiejun Wu, Dongchang Xi Rd 67#, Liaocheng 252003, China, Email tiejunwu@hotmail.com or Cheng Zhang, Wenhua Xi Road 107#, Jinan, 250012, China, Email zhangc@sdu.edu.cn

*J. Jin, L. Gao, and X. Zou contributed equally.

Supplemental Material is available at https://www.ahajournals.org/doi/suppl/10.1161/CIRCRESAHA.122.320771.

For Sources of Funding and Disclosures, see page XXX.

© 2022 American Heart Association, Inc.

Circulation Research is available at www.ahajournals.org/journal/res

ORIGINAL RESEARCH

Novelty and Significance

What Is Known?

- Preeclampsia is characterized by hypertension, inflammation, and placental dysfunction.
- Gut microbiota and its metabolites play key roles in inflammation and hypertension.
- Intestinal flora of preeclamptic patients and animals showed significant dysbiosis.

What New Information Does This Article Contribute?

- Gut microbiota dysbiosis is present in preeclamptic patients and dysbiosis of intestinal flora may contribute to the development of preeclampsia.
- *Akkermansia muciniphila* regulates macrophage polarization and spiral artery remodeling in the placenta through metabolite propionate/butyrate and alleviates the findings in preeclamptic rats.
- A biomarker panel consisting of intestinal bacteria and short-chain fatty acids may have the potential to demonstrate preeclampsia.

Preeclampsia is one of the leading causes of maternal and perinatal morbidity, in which inflammation and placental dysfunction play crucial roles. Currently, effective methods of early screening and treatment for preeclampsia are still lacking. Numerous studies have demonstrated that gut microbiota and their metabolites are key regulators in host homeostasis and diseases. Intestinal flora of preeclamptic patients and animals showed significant dysbiosis. However, the causal relationship between gut dysbiosis and preeclampsia and its underlying mechanisms have not been elucidated to date. In the present study, we revealed the gut dysbiosis in preeclamptic patients, characterized by significant reductions in short-chain fatty acid-producing bacteria and short-chain fatty acids. The gut microbiota of preeclamptic patients significantly exacerbated pathologies and symptoms of preeclamptic rats, whereas the gut microbiota of healthy pregnant women had significant protective effects. Mechanistically, *Akkermansia muciniphila,* propionate, or butyrate significantly alleviated symptoms of preeclamptic rats by promoting autophagy and M2 polarization of macrophages and spiral arterial remodeling in placental bed. Additionally, we also identified a marker set consisting of *Akkermansia*, *Oscillibacter*, and short-chain fatty acids with potential to adjunctively diagnose preeclampsia. Our findings enrich the gut–placenta axis theory and contribute to developing microecological products for adjunctive diagnosis and treatment of preeclampsia.

Nonstandard Abbreviations and Acronyms

**Eng**	endoglin
**Flt-1**	fms-like tyrosine kinase receptor-1
**GPCR**	G protein-coupled receptor
**IQR**	interquartile range
**MIF**	macrophage migration inhibitory factor
**SCFA**	short-chain fatty acid
**TLR4**	toll-like receptor 4

mechanisms of spiral artery dysplasia are incompletely characterized to date.

The human gut microbiota and their metabolites are key regulators of host immune system including macrophage.[5] Lipopolysaccharide and short chain fatty acids (SCFAs) produced by intestinal bacteria can regulate macrophage polarization and inflammation by inhibiting histone deacetylase.[6] The gut microbiota and its metabolites have also been reported to be closely related to the placental function. For example, SCFAs contribute to placental integrity and promote placental vascularization.[7] Intestinal-derived endotoxin induces inflammatory responses in the maternal placenta.[8]

Accumulating evidence suggests that the gut microbiota dysbiosis plays critical roles in many diseases, including hypertension, the typical symptom of preeclampsia. The gut microbiota of hypertensive patients and animals is notably abnormal.[9] Improvement of the gut dysbiosis can reduce blood pressure (BP).[10] Intestinal *Lactobacillus murinus*, which is depleted in high-salt-induced hypertensive mice, was shown to inhibit the differentiation of T lymphocytes to Th17 cells by producing indole compounds, thereby reducing inflammation and BP.[11] Moreover, *Bacteroides fragile* has been reported to reduce BP by antagonizing the production of intestinal-derived corticosterone[12].

Considering these relationships between gut microbiota and hypertension, inflammation, and placental function, speculation that gut microbiota plays important roles in preeclampsia seems obvious. Some studies showed that high intake of probiotics containing *lactobacilli* was associated with a reduced risk of preeclampsia.[13] The gut microbiota of preeclamptic patients (PEs) was markedly abnormal.[14–17] Recently, 2 studies showed that gut microbiota dysbiosis and intestinal-derived SCFAs were involved in preeclampsia.[18,19] Another study suggested that abnormal gut microbiota disrupted the gut barrier and led to colonization of intestinal bacteria in the uterine cavity, thus causing preeclampsia.[20] However, the

Downloaded from http://ahajournals.org by on August 11, 2022

presence or absence of bacteria in the placenta is still hotly debated.[21]

In the present study, we demonstrated the gut dysbiosis in PEs, which is an important cause of preeclampsia. *Akkermansia muciniphila* and propionate/butyrate, whose abundances notably decreased in PEs, significantly alleviated the symptoms of preeclamptic rats by regulating macrophage polarization and spiral artery remodeling in the placenta, indicating their great potential for auxiliary treatment and diagnosis of preeclampsia.

METHODS

An expanded Methods section is available in the Supplemental Material. The datasets can be downloaded from GSA database (CRA006784).

RESULTS

Significant Gut Dysbiosis in Preeclamptic Patients

Herein, the feces of 92 PEs and 86 normal late-pregnant women (LPs) were analyzed using 16S rRNA gene sequencing (Table S1). The average sequencing depth was saturated and not significantly different between the 2 groups (Figure S1A and S1B). However, the α and β diversity and *Firmicutes/Bacteroidetes* (F/B) ratio of the gut microbiota in PEs were significantly changed, suggesting gut dysbiosis (Figure 1A and 1B; Figure S1C and S1D). At phylum and genus levels, the abundances of many bacteria were markedly changed in PEs and plenty of them were significantly correlated with key clinical indicators of preeclampsia. For example, the abundance of *Akkermansia*, which was obviously reduced in PEs, was significantly negatively correlated with BP and UP (Figure 1C through 1E; Figure S1E). These results implied that these intestinal bacteria might be involved in preeclampsia.

Additionally, we found that the correlations among harmful or beneficial bacterial genera in the intestine were mainly positive, while the correlations between beneficial and harmful bacterial genera were primarily negative (Figure S1F and S1G). In the gut microbiota of PEs, the positive correlations among harmful bacteria and the negative correlations between beneficial and harmful bacteria were markedly increased (Figure S1G). This suggested that the co-symbiosis of gut microbiota in PEs was disordered, which might be related to the gut dysbiosis in PEs.

Significant Reductions in Intestinal SCFA-Producing Bacteria and SCFAs Correlates With Symptoms of PEs

SCFA-producing bacteria and SCFAs are known to play key roles in host homeostasis. We found that the abundances of many intestinal SCFA-producing bacteria were significantly reduced in PEs (Figure 2A). Moreover, the levels of 3 key enzymes for SCFA production in intestinal bacteria were also significantly decreased in PEs, indicating the decreased ability of intestinal bacteria to produce SCFAs (Figure 2B). Especially, the fecal, serum, and placental levels of propionic acid and butyric acid were significantly reduced in PEs, and their levels in feces, serum, and placenta significantly positively correlated with each other (Figure 2C and 2D). These results suggested that propionic and butyric acid produced by intestinal bacteria may reach the placenta via the circulation. Moreover, the placental levels of propionic and butyric acid, but not other SCFAs were significantly negatively correlated with BP and UP (Figure 2E; Figure S2A). Some SCFA-producing bacteria, including *A. muciniphila,* whose abundance was significantly reduced in the feces of PEs, were significantly positively correlated with the levels of propionate and butyrate in the placenta (Figure 2F). In particular, the significant reduction of *A. muciniphila* in the feces of PEs was confirmed by qPCR (Figure 2G).

American Heart Association.

Intestinal Bacteria and SCFAs Have Potential for Preeclampsia Diagnosis

To assess the value of gut microbiota and SCFAs in preeclampsia diagnosis, a linear discriminant effect size analysis was performed. Fifteen intestinal bacteria with linear discriminant analysis scores >3 (<−3) were identified at the genus level (Figure S2B). Using the 16S rRNA gene sequencing data of 139 samples as the training dataset, a prediction model consisting of intestinal bacteria for preeclampsia diagnosis was established using the random forest algorithm (Figure 3A). Then, its accuracy was verified in another sample set of 20 PEs and 19 LPs. The results demonstrated that PEs and LPs could be distinguished only based on the abundances of *Akkermansia* and *Oscillibacter* with 89.7% accuracy (Figure 3B). The diagnostic efficacy of the marker set comprising intestinal bacteria and/or serum propionic acid and butyric acid was comparable to those of the main clinical indicators (SBP, DBP, and UP). Moreover, the diagnostic efficacy of this marker set was obviously increased by integrating BP and UP, which increased the accuracy to 98.98% (Figure 3C). These results implied that intestinal bacteria and circulating SCFAs have potential as diagnostic markers for preeclampsia.

Intestinal Barrier Damage and Inflammation Significantly Enhanced in PEs

SCFA-producing bacteria and SCFAs are key regulators of host inflammation and intestinal barrier. Using flow cytometry, we found that compared with LPs, the percentages of Treg and Th17 cells in the peripheral

Circulation Research FIRST PROOF ONLY

Downloaded from http://ahajournals.org by on August 11, 2022

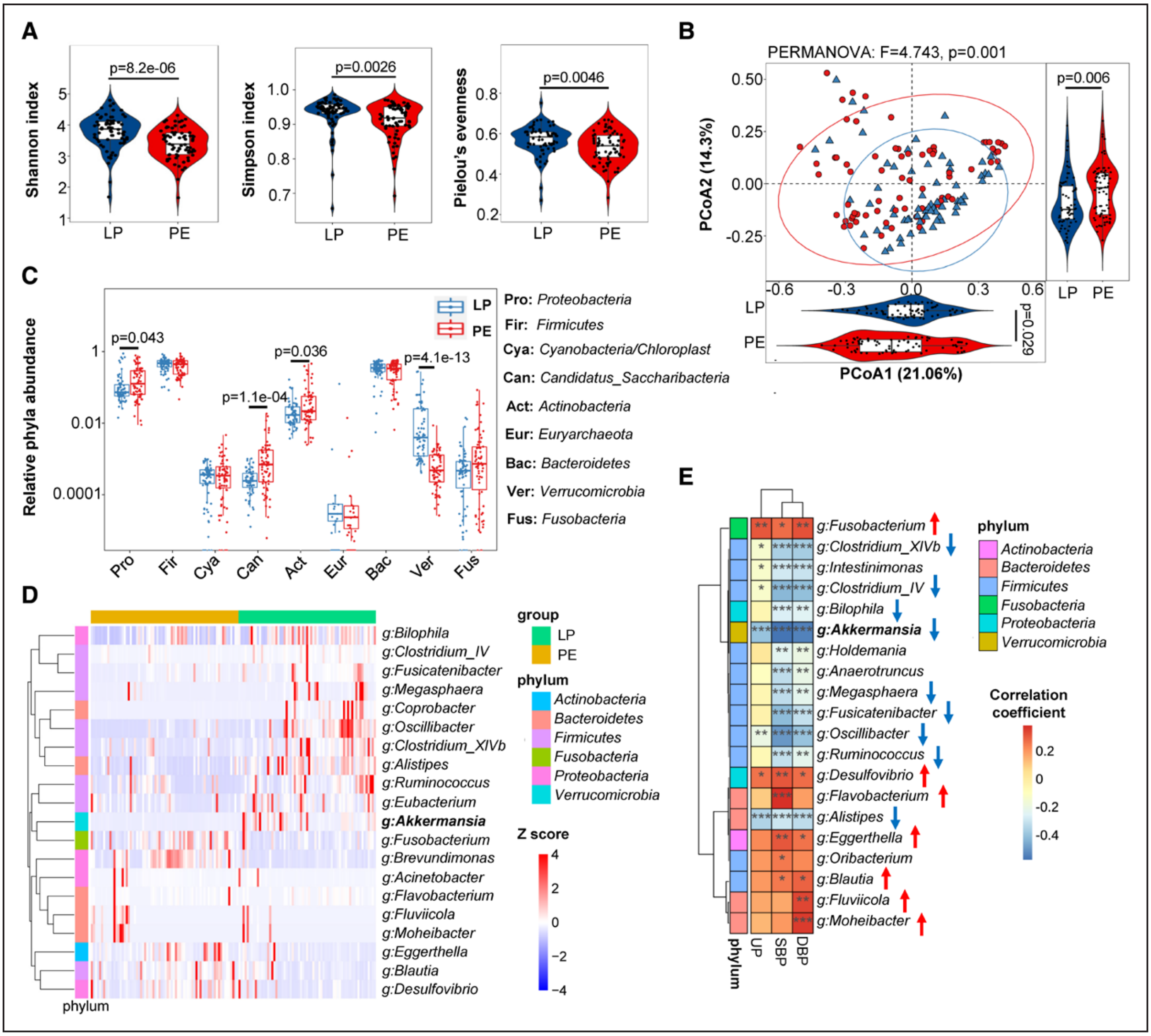

Figure 1. Gut dysbiosis in preeclamptic patients.
A, Comparison of α diversity of the gut microbiota in healthy late-pregnant women (LPs) and preeclamptic patients (PEs). **B**, Plot of principal coordinates analysis (PCoA) based on the Bray–Curtis distance matrix of the 16S rRNA gene sequencing data. **C**, Comparison of the relative abundances of gut bacterial phyla in the PEs and LPs groups. **D**, Heatmap of the relative abundances of the top 20 significantly differentially abundant gut bacteria in the PEs group compared with the LPs group at genus level. **E**, Correlation heatmap between clinical indicators of preeclampsia and the abundant bacterial genera. For **C** and **D**, relative abundance: read number of a certain phylum/genus divided by the total read number of all bacteria in this sample. Data are presented as median and IQR in **A**–**C**. Wilcoxon rank–sum test was used for statistical analysis in **A** and **B**. Wilcoxon rank–sum test (**C**, **D**) and Spearman's correlation test (**E**) were used for statistical analysis with FDR adjustment (FDR<0.05). *Adjusted P<0.05; **Adjusted P<0.01; ***Adjusted P<0.001. LPs: n=67; PEs, n=72.

blood CD4+ cells of PEs were significantly lower and markedly higher, respectively (Figure 4A; Figure S2C), which resulted in a significantly decreased peripheral Treg/Th17 ratio in PEs (Figure 4B). CCR9 is an important intestinal homing marker. Our results showed that the proportion of CCR9+ Treg cells in peripheral CD4+ T cells or Treg cells of PEs was significantly decreased, while the proportion of CCR9+ Th17 cells in peripheral CD4+ T cells or Th17 cells was significantly increased (Figure 4C and 4D). This implied that the abnormalities of peripheral Treg and Th17 cells in PEs were closely related to the intestine. Moreover, the serum IL-10 level was markedly decreased, while the serum levels of IL-17 and IL-1β were significantly increased in PEs (Figure 4E). Additionally, the serum level of lipopolysaccharide, an essential indicator of intestinal barrier damage, was considerably increased in PEs. However, the fecal level of 2-Arachidonoylglycerol, a protector of the intestinal barrier, was significantly decreased in PEs (Figure 4F). These results indicated intestinal barrier damage in PEs. Furthermore, there were strong correlations between the above indicators (Figure 4G). *Akkermansia*

Downloaded from http://ahajournals.org by on August 11, 2022

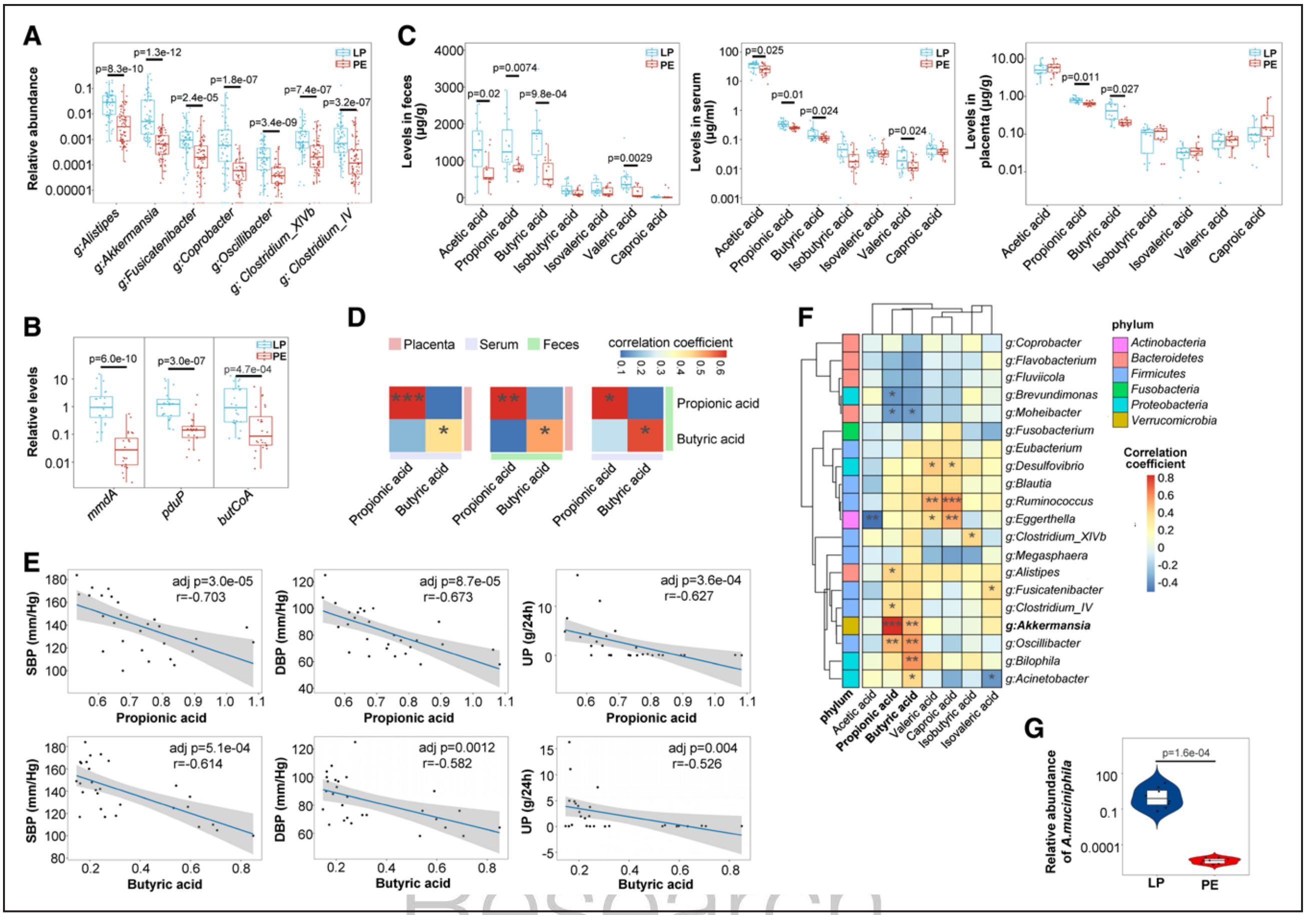

Figure 2. The levels of short-chain fatty acid (SCFA)-producing bacteria and SCFAs and the correlations among them and clinical indicators in preeclamptic patients.

A, Comparison of the relative abundances of representative SCFA-producing bacteria (read number of a certain bacterial genus divided by the total read number of all bacteria in this sample) in healthy late-pregnant women (LPs, n=67) and preeclamptic patients (PEs, n=72). **B**, Comparison of the relative levels of the key enzyme genes for producing propionate and butyrate compared to the level of universal 16S rRNA gene in the intestinal bacteria of LPs (n=24) and PEs (n=24). *mmdA*: methylmalonyl-CoA decarboxylase; *pduP*: propionaldehyde dehydrogenase; *butCoA*: butyryl-CoA transferase. **C**, Comparison of the levels of SCFAs in feces (n=15), serum (n=20), and placenta (n=14) of PEs and LPs. **D**, Correlation heatmap of propionic and butyric acid levels in feces, serum, and placenta. **E**, The scatter plots showing the relationships between the placental levels of propionic and butyric acid and key clinical indicators of preeclampsia. r: Spearman correlation coefficient; adj p: Holm adjusted *P*. **F**, Correlation heatmap between the placental levels of SCFAs and 20 significantly differentially abundant bacteria in preeclampsia. **G**, Comparison sof the relative abundance of *Akkermansia muciniphila* compared with the abundance of universal 16S rRNA gene in feces (n=8) of the LPs and PEs. Data are presented as median and IQR in **A**–**C** and **G**. For statistical analysis, Wilcoxon rank–sum test with FDR adjustment (<0.05) was used in **A** and **C**. Wilcoxon rank–sum test were used in **B** and **G**. Spearman correlation test with FDR adjustment (<0.05) was used in **D** and **F**. *Adjusted *P*<0.05; **adjusted *P*<0.01; ***adjusted *P*<0.001.

Research FIRST PROOF ONLY

abundance was also significantly positively correlated with the levels of fecal 2-Arachidonoylglycerol and serum IL-10 but negatively correlated with the serum levels of lipopolysaccharide and IL-17 (Figure 4H).

Some studies have suggested that intestinal barrier damage causes intestinal bacteria to enter the circulation and colonize extraintestinal tissues, including the placenta. However, many studies oppose this idea. Therefore, we detected intestinal bacteria that were reported to be present in the placenta in most studies by qPCR. The results showed that the abundances of these bacteria were very low in the placentas of both LPs and PEs and did not differ significantly compared to those in the negative controls without template DNA (blank) (Figure S2D). This result indicated that the reported bacteria in the placenta are likely to originate from contamination during the experimental procedure.

Gut Dysbiosis Contributes the Development of Preeclampsia

To evaluate the causal relationship between gut dysbiosis and preeclampsia, fecal microbiota transplantation was performed in the classical preeclamptic rat model (Figure S3). The results showed that quadruple broad-spectrum antibiotics effectively eliminated the gut microbiota of the rats. The gut microbiota from the donors successfully colonized recipient rats (Figure S4A through S4C). The BP of healthy pregnant rats remained relatively constant and normal throughout pregnancy (Figure 5A). The

Downloaded from http://ahajournals.org by on August 11, 2022

ORIGINAL RESEARCH

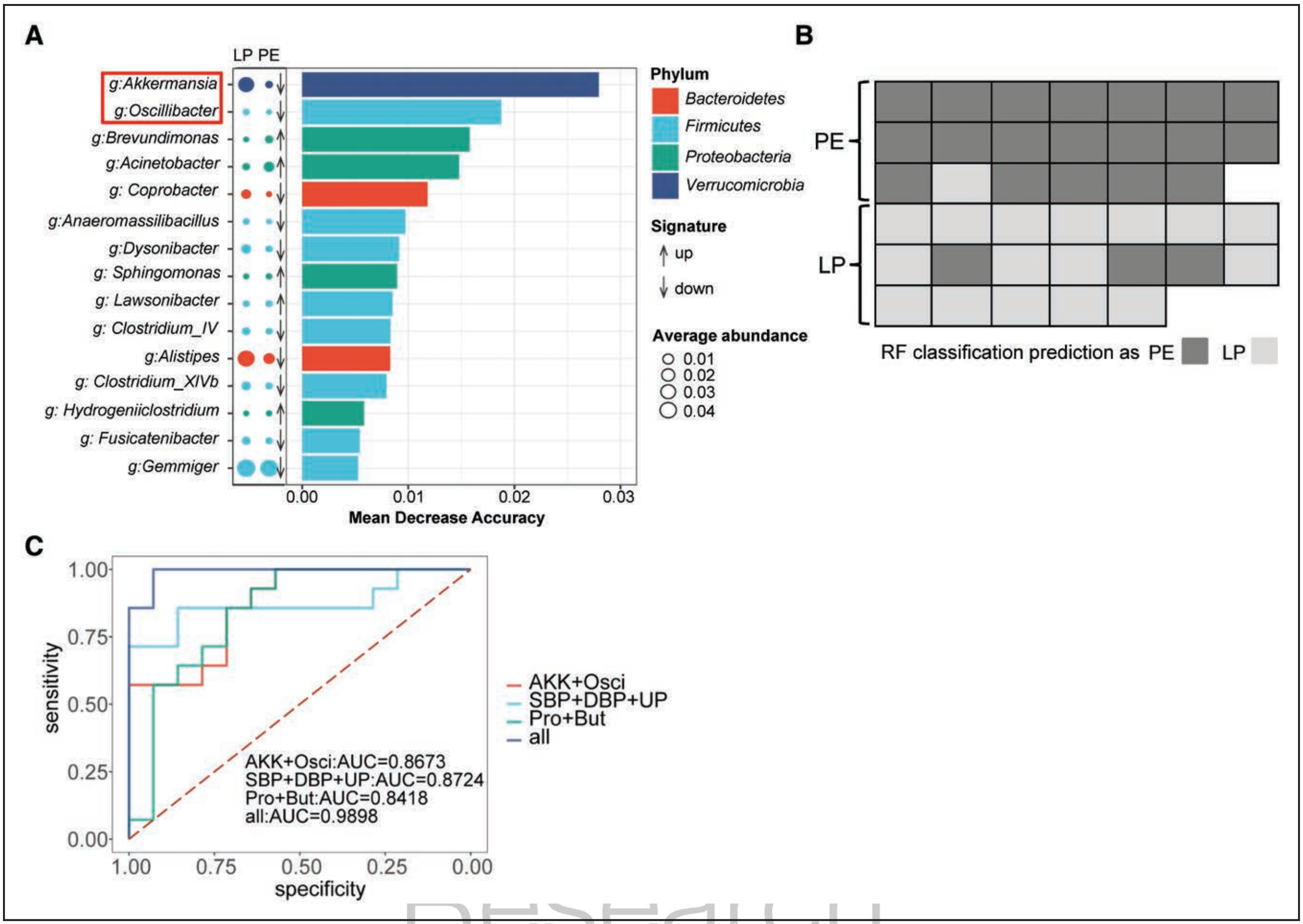

Figure 3. The potential of intestinal bacteria for preeclampsia diagnosis.
A, The characteristic gut bacteria identified by random forest using the 16S rRNA gene sequencing data of 139 samples of healthy late-pregnant women (LPs) and preeclamptic patients (PEs). A rectangle indicates 2 gut bacterial genera that accurately distinguish PEs and LPs with 89.7% accuracy. g: genus. **B**, Graphic representation of the accuracy verification of the classifier (*Akkermansia* and *Oscillibacter*) in another test dataset of PEs (n=20) and LPs (n=19). **C**, Receiver operating characteristic (ROC) curves for the efficacy of clinical indicators, intestinal bacteria, and serum SCFAs alone or in combination for preeclampsia diagnosis. AKK: *A. muciniphila*; Osci: *Oscillibacter*; Pro: propionate; But: butyrate.

Research FIRST PROOF ONLY

Downloaded from http://ahajournals.org by on August 11, 2022

fecal microbiota transplantation results demonstrated that the gut microbiota of PEs significantly elevated BP levels (Figure 5A; Figure S4D and S4E) but had no significant effects on inflammatory factor levels, intestinal barrier function (except for ZO-1 protein) or spiral artery remodeling in normal pregnant rats (Figure 5B through 5G). However, in preeclamptic rats, the gut microbiota of PEs significantly elevated BP and serum IL-17 levels but significantly decreased serum IL-10 level, indicating an enhanced inflammatory response (Figure 5A and 5B; Figure S4D and S4E). Moreover, the gut microbiota of PEs significantly elevated serum lipopolysaccharide levels but decreased fecal 2-Arachidonoylglycerol levels, goblet cell numbers per crypt, and tight junction protein expression in the intestine of preeclamptic rats. These results indicated significant disruption of intestinal barrier (Figure 5C through 5E). However, the gut microbiota of LPs significantly alleviated the aforementioned pathological abnormalities including spiral artery dysfunction in preeclamptic rats (Figure 5A through 5G). Additionally, the gut microbiota of PEs significantly reduced fecal *A. muciniphila* and the key enzymes for propionate and butyrate production (*pduP* and *butCoA*). Conversely, the opposite was observed for the gut microbiota of LPs (Figure S4F and S4G). There are significant correlations among the levels of fecal *A. muciniphila*, *butCoA*, *pduP*, intestinal barrier-related indicators, and BP (Figure S4H and S4I). These results demonstrated that gut dysbiosis could aggravate intestinal barrier damage and contribute to preeclampsia development.

A. muciniphila and Propionate/Butyrate Alleviate the Symptoms of Preeclamptic Rats by Improving Spiral Artery Remodeling and Inflammation in the Placenta

Targeted metabolome analysis showed a significant increase in acetic and propionic acid levels in the culture supernatants of *A. muciniphila*, suggesting that *A. muciniphila* could directly produce acetic and propionic acid (Figure S5A). These products can be used as substrates for butyric acid production by other intestinal

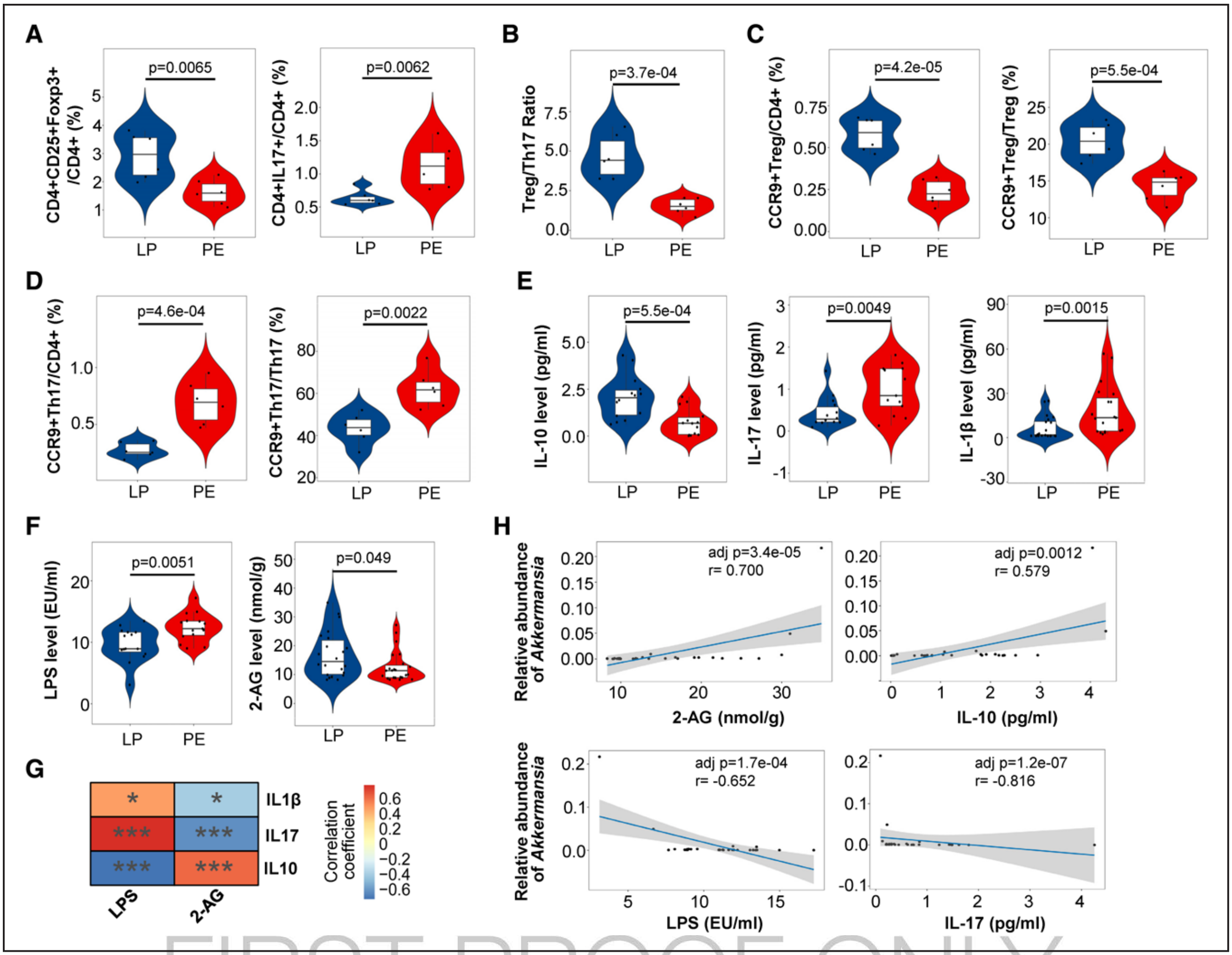

FIRST PROOF ONLY

Figure 4. Increased inflammation and impaired intestinal barrier function and their correlations in preeclamptic patients.
A, Comparison of the percentages of Treg (CD4+CD25+Foxp3+) and Th17 (CD4+IL17+) cells among the peripheral CD4+ T cells of healthy late-pregnant women (LPs) and preeclamptic patients (PEs). **B**, Comparison of the peripheral Treg/Th17 cell ratios of the LPs and PEs groups. **C**, Comparison of the percentages of CCR9+ Treg cells in CD4+ T cells or in Treg cells in the peripheral blood of the LPs and PEs groups. **D**, Comparison of the percentages of CCR9+ Th17 cells in CD4+ T cells or in Th17 cells in the peripheral blood of LPs and PEs groups. For **A**–**D**, n=6. **E**, Comparison of serum levels of IL-10 (n=14), IL-17 (n=14), and IL-1β (LPs: 20, PEs: 19) in LPs and PEs groups. **F**, Comparison of the serum levels of LPS (n=15) and fecal levels of 2-AG (LPs: 22, PEs: 23) in PEs and LPs groups. **G**, Correlation heatmap between inflammatory factors and intestinal barrier-related factors. **H**, The scatter plots showing the relationships between the relative levels of *Akkermansia* (read number of *Akkermansia* genus divided by the total read number of all bacteria in this sample) and 2-AG as well as the serum levels of IL-10, LPS, and IL-17. r: Spearman correlation coefficient; adj p: Holm adjusted *P*. Data are presented as median and IQR in **A**–**F**. T test (**A**–**D**, LPS in **F**) and Wilcoxon rank-sum test (**E**, 2-AG in **F**) were used for statistical analysis. Spearman's correlation test was used for statistical analysis with FDR adjustment in G. *Adjusted $P<0.05$; ***Adjusted $P<0.001$.

bacteria. Therefore, to reveal the roles and mechanisms of *A. muciniphila* in preeclampsia, we treated preeclamptic rats with *A. muciniphila*, sodium propionate, or sodium butyrate by oral administration (Figure S6A). The results showed a significant increase in the abundance of fecal *A. muciniphila* in *A. muciniphila*-treated preeclamptic rats (AKKToPErat), suggesting its successful colonization (Figure S5B). *A. muciniphila*, propionate, and butyrate significantly increased propionate and butyrate levels in the feces, serum, and/or placenta (Figure S5C and S5D), and they also significantly decreased BP and increased the weight and size of the placenta and embryo in preeclamptic rats (Figure 6A through 6C). Moreover, *A. muciniphila* and propionate significantly upregulated Cytokeratin 7 expression and downregulated α-SMA expression in the spiral arteries of preeclamptic rats, indicating significant improvement in spiral artery remodeling (Figure 6D and 6E). We found that *A. muciniphila* and butyrate significantly increased the expression of claudin-1, occludin, and ZO-1 and goblet cell numbers in the intestinal epithelium of preeclamptic rat, while propionate only significantly upregulated the expression of claudin-1 and occludin. These results indicated that intestinal barrier damage in preeclamptic rats was significantly improved after treatment (Figure 6F and 6G). We also found that *A.*

Downloaded from http://ahajournals.org by on August 11, 2022

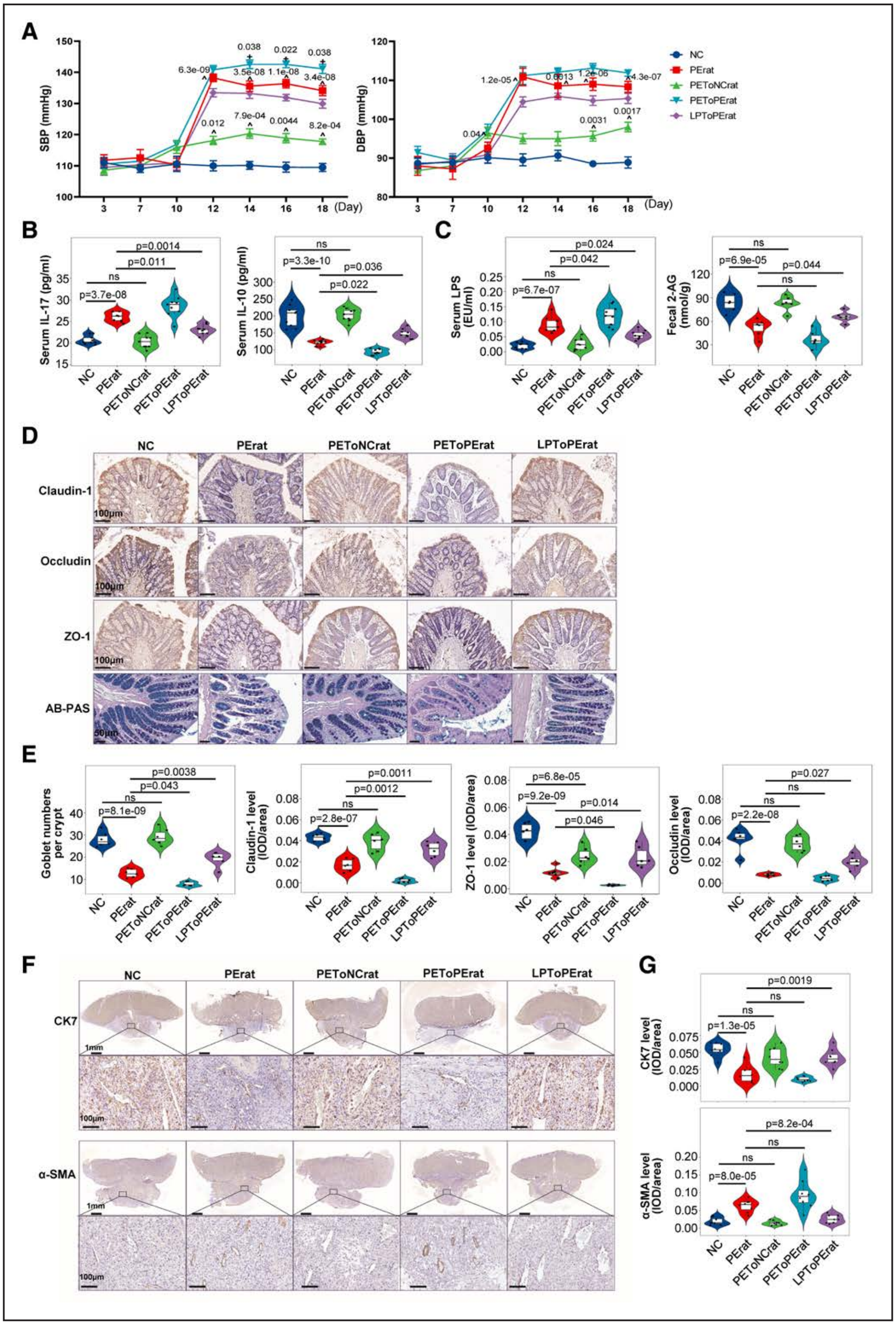

Figure 5. Fecal microbiota transplantation (FMT) in the preeclamptic rats.

A, Dynamic changes and comparison of systolic blood pressure (SBP) and diastolic blood pressure (DBP) of the pregnant rats in different groups during pregnancy. NC: normal pregnant rats; PErat: preeclamptic rats induced by L-NAME; PEToNCrat (PEToPErat): normal pregnant rats (preeclamptic rats) transplanted with fecal microbiota of preeclamptic patients; LPToPErat: preeclamptic rats transplanted with fecal microbiota of healthy late-pregnancy women. n=9. **B** and **C**, Comparison of serum levels of IL-17 (n=9) and IL-10 (n=9) (**B**) as well as serum levels of LPS (n=9) and fecal levels of 2-AG (n=6) (**C**) in pregnant rats of different groups. **D** and **E**, Immunohistochemistry images of colon tissues of the pregnant rats in different groups stained with claudin-1, occludin, ZO-1, and Alcian blue/periodic acid–Schiff (AB-PAS) (**D**) and quantitative comparison of goblet cell numbers per crypt and expression levels of claudin-1, ZO-1, and occludin (**E**). n=6. **F** and **G**, Immunohistochemistry images of CK7 and α-SMA protein in the placenta of pregnant rats with different treatments (**F**) and quantitative comparison of their levels (**G**). n=8. Data are presented as mean±SEM (**A**) and median and IQR (**B**, **C**, **E**, **G**). Two-way ANOVA with Tukey's post hoc test was used for statistical analysis in **A**. One-way ANOVA with Tukey post hoc test was used for statistical analysis in **B**, **C**, **E**, and **G**. ns: not significant. For **A**, ^adjusted *P* vs NC. +: adjusted *P* vs PErat.

Downloaded from http://ahajournals.org by on August 11, 2022

ORIGINAL RESEARCH

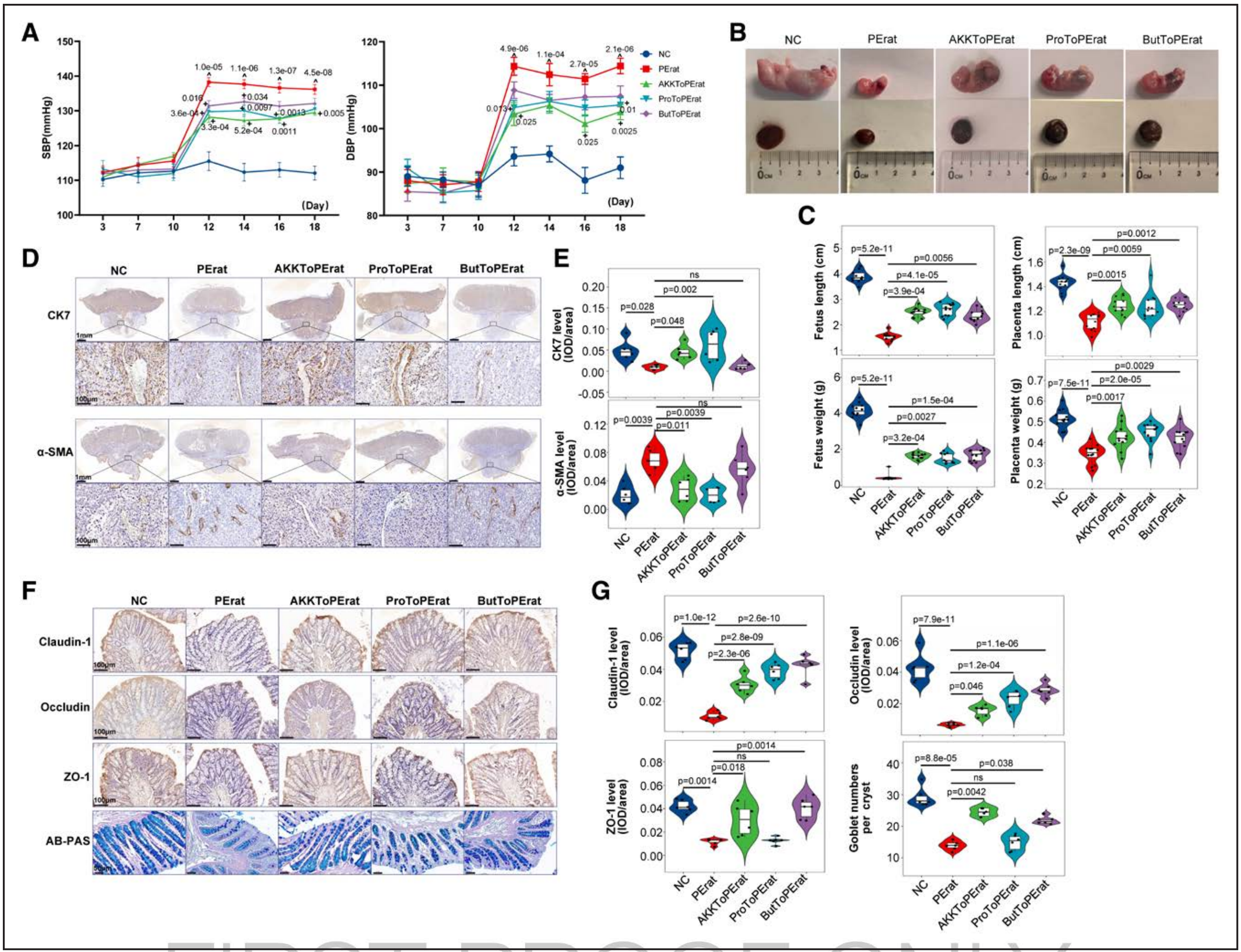

FIRST PROOF ONLY

Figure 6. Akkermansia *muciniphila*, propionate, or butyrate significantly alleviate pathological abnormalities in preeclamptic rats.

A, Dynamic changes and comparison of systolic blood pressure (SBP) and diastolic blood pressure (DBP) of the pregnant rats in different groups during pregnancy. NC: normal pregnant rats; PErat: preeclamptic rats induced by L-NAME; AKKToPErat: preeclamptic rats transplanted with *A. muciniphila*; ProToPErat (ButToPErat): sodium propionate (sodium butyrate)-treated preeclamptic rats. n=12. **B** and **C**, Representative images of embryo and placenta morphology of pregnant rats in each group (**B**) and comparison of their length and weight (**C**). n=12. **D** and **E**, Immunohistochemistry images of CK7 and α-SMA protein in the placenta of pregnant rats in each group (**D**) and comparison of their expression levels (**E**). n=6. **F** and **G**, Immunohistochemistry images of colon tissues of pregnant rats in each group stained with claudin-1, occludin, ZO-1, and AB-PAS (**F**), and comparison of their expression levels and goblet cells numbers per crypt (**G**). n=6. Data are presented as mean±SEM (**A**) or median and IQR (**C**, **E**, **G**). Two-way ANOVA with Tukey post hoc test was used for statistical analysis in **A**. One-way ANOVA with Tukey post hoc test was used for statistical analysis in **C** (placenta weight), **E** (CK7), and **G** (claudin-1, occludin). Kruskal-Wallis test with FDR's post hoc test was used for statistical analysis in **C** (fetus length, fetus weight, and placenta length), **E** (α-SMA), and **G** (ZO-1 and goblet). ns: not significant. For A, ^: adjusted *P* vs NC +: adjusted *P* vs PErat.

Downloaded from http://ahajournals.org by on August 11, 2022

muciniphila, propionate, and butyrate significantly downregulated the mRNA and protein expression of placental inflammatory factors (*Tlr4*, *Mif*, *Tnf*, and *Mcp1/Ccl2*) in preeclamptic rats (Figure S5E through S5G). Additionally, these 3 treatments significantly reduced the mRNA and soluble protein levels of antiangiogenic factor Eng (endoglin) in placenta of preeclamptic rats, and butyrate also had the same effect on Flt-1 (fms-like tyrosine kinase receptor-1; Figure S6B and S6C). Modulation of these angiogenesis-related factors may be an important mechanism by which *A. muciniphila*, propionate, and/or butyrate ameliorate poor spiral artery remodeling in preeclamptic rats.

A. muciniphila and Propionate/Butyrate Reduce Inflammation by Promoting Autophagy and M2 Polarization of Macrophages in the Placental Bed

Placental inflammation plays a central role in preeclampsia, and macrophages are the key regulators of inflammation. We found that in the placental bed of preeclamptic rats, *A. muciniphila*, propionate, or butyrate significantly increased M2 macrophage proportion and decreased M1 macrophage proportion and P62+ macrophage level, thereby promoting M2 polarization and autophagy of macrophages (Figure 7A and 7B; Figure S7A and S7B).

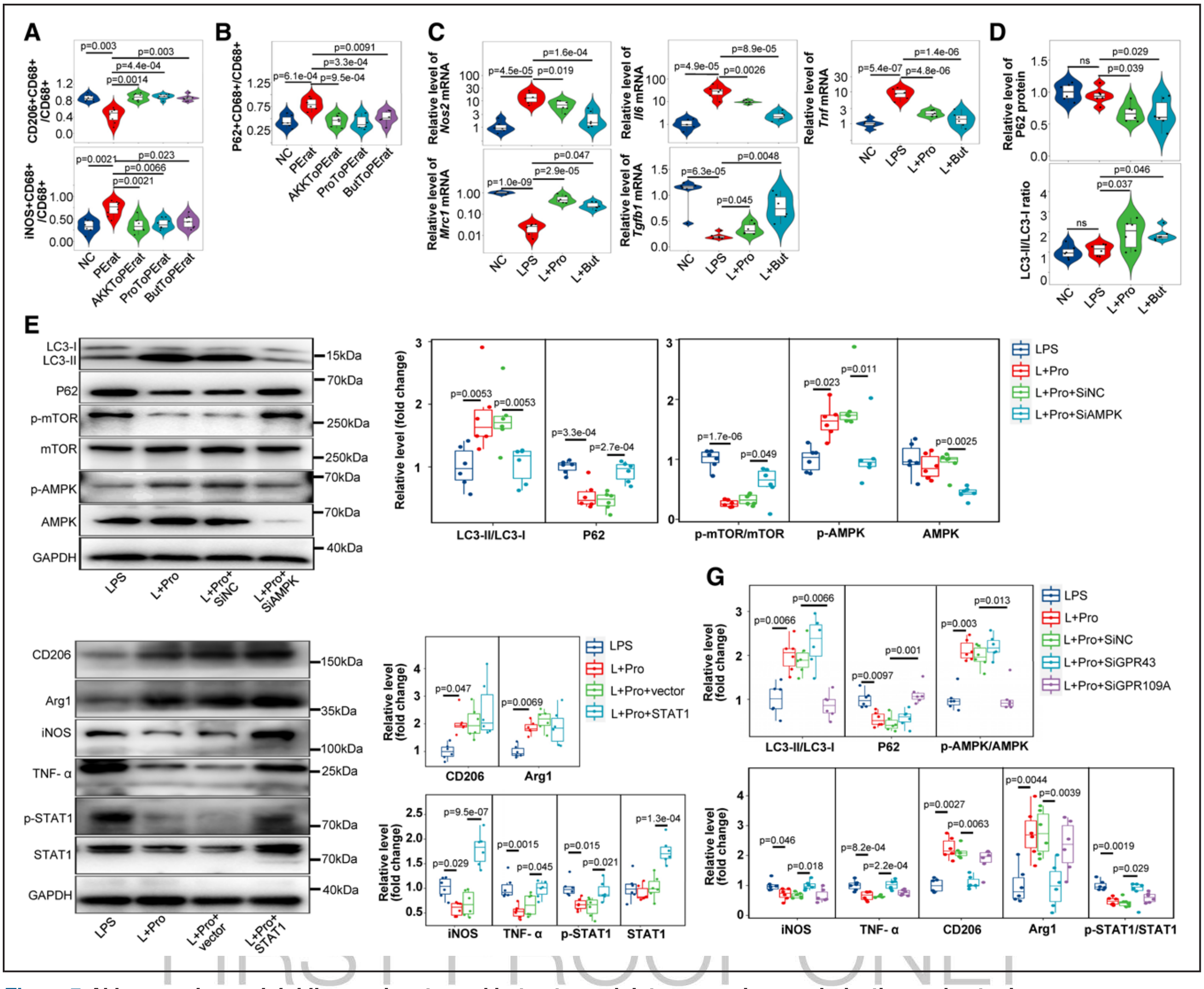

Figure 7. Akkermansia *muciniphila*, propionate, and butyrate modulate macrophage polarization and autophagy.
A and **B**, Comparison of the relative levels of CD206+M2 and iNOS+M1 macrophages (**A**, n=8) and P62+ macrophages (**B**, n=6) compared with macrophage (CD68+) levels in the placental bed of pregnant rats in each group. NC: normal pregnant rats; PErat: preeclamptic rats induced by L-NAME; AKKToPErat: preeclamptic rats transplanted with *A. muciniphila*; ProToPErat (ButToPErat) sodium propionate (sodium butyrate)-treated preeclamptic rats. **C**, Comparison of the relative expression levels of *Nos2*, *Il6*, *Tnf*, *Mrc1*, and *Tgfb1* mRNA compared with *Gapdh* mRNA in each group of RAW264.7 cells. n=6. **D**, Comparison of the relative expression levels of P62 protein compared with GAPDH protein and LC3-II/LC3-I ratio in each group of RAW264.7 cells. n=6. **E** and **F**, Representative Western blot images of LC3, P62, phosphorylated (p)-mTOR, mTOR, phosphorylated (p)-AMPK, and AMPK (E) or CD206, Arg1, iNOS, TNF-α, phosphorylated (p)-STAT1, and STAT1 (F) in RAW264.7 cells with different treatments (left panel) and comparison of their relative levels compared to GAPDH protein (right panel). n=6. **(G)** Comparison of ratios of LC3-II/LC3-I, p-AMPK/AMPK, or p-STAT1/STAT1 and the relative expression levels of P62, iNOS, TNF-α, CD206, and Arg1 proteins compared with GAPDH protein in RAW264.7 cells with different treatments. n=6. Data are presented as median and IQR. One-way ANOVA with Tukey's post hoc test was used for statistical analysis in B, C (except for Tgfb1), D, E (P62 and p-mTOR/mTOR), F, and G (iNOS, TNF-α, Arg1, and p-STAT1/STAT1). Kruskal-Wallis test with FDR's post hoc test was used for statistical analysis in A, C (Tgfb1), E (LC3-II/LC3-I, p-AMPK, and AMPK), and G (LC3-II/LC3-I, P62, p-AMPK/AMPK, and CD206). ns: not significant. NC: negative control; LPS: LPS treatment (100 ng/ml); L+Pro: LPS and sodium propionate (600 μmol/L) treatment; L+But: LPS and sodium butyrate (600 μmol/L) treatment. L+Pro+SiNC (L+Pro+SiAMPK/SiGPR43/SiGPR109A): LPS, sodium propionate, and negative control of siRNA (SiRNA of AMPK, GPR43, or GPR109A) treatment. L+Pro+vector (STAT1): LPS, sodium propionate, and STAT1 empty (expression) vector treatment.

Considering that *A. muciniphila* can produce propionic and butyric acid either directly or indirectly and lipopolysaccharide level significantly increased in the placenta of PEs (Figure S7C), we treated RAW264.7 cells with propionate, butyrate, and/or lipopolysaccharide. The results demonstrated that propionate and butyrate significantly reduced the effects of lipopolysaccharide to promote M1 polarization and inhibit M2 polarization (Figure 7C; Figure S8A and S8B). Moreover, they also noticeably decreased P62 protein level and elevated LC3-II/LC3-I ratio, suggesting their promoting effects on macrophage autophagy (Figure 7D; Figure S8C). Additionally, the culture supernatant of *A. muciniphila* but not acetic acid had effects like those of propionate as described above without significant differences, implying that *A. muciniphila* might promote autophagy and M2 polarization of

Downloaded from http://ahajournals.org by on August 11, 2022

macrophages through its metabolite propionate (Figure S8D and S8E).

mTOR can inhibit autophagy and be negatively regulated by AMPK. We found that in lipopolysaccharide-treated RAW264.7 cells, propionate significantly decreased p-mTOR/mTOR ratios and increased p-AMPK levels. Meanwhile, propionate significantly increased LC3-II/LC3-I ratios while downregulating P62 expression, suggesting enhanced autophagy. Moreover, AMPK siRNA significantly diminished the above effects of propionate (Figure 7E). Additionally, STAT1 can promote M1 macrophage polarization. Our results showed that in lipopolysaccharide-treated RAW264.7 cells, propionate significantly reduced p-STAT1 levels, accompanied by a significant downregulation of iNOS and TNF-α protein. However, STAT1 overexpression significantly diminished the above effects of propionate (Figure 7F). We also found that GPR43 and GPR109A but not GPR41 were expressed in RAW264.7 cells (Figure S8F). In lipopolysaccharide-treated RAW264.7 cells, GPR109A siRNA significantly reduced the effects of propionate in enhancing autophagy and increasing p-AMPK/AMPK ratios. Additionally, GPR43 siRNA significantly attenuated the effects of propionate in inhibiting M1 polarization, promoting M2 polarization and decreasing p-STAT1/STAT1 ratios. Considering that GPR43, but not GPR109A, is the receptor for propionate, the above results implied that propionate might regulate macrophage autophagy through a receptor-independent mechanism involving AMPK and GPR109A. Meanwhile, propionate promoted macrophage M2 polarization through the GPR43-STAT1 pathway (Figure 7G; Figure S8G and S8H).

Our results also showed that in lipopolysaccharide-treated RAW264.7 cells, the autophagy inhibitor chloroquine significantly reduced the effects of propionate on macrophage polarization, indicating that autophagy suppression promotes M1 polarization and inhibits M2 polarization of macrophages. However, the autophagy inducer rapamycin had no significant impact on the effects of propionate on macrophage polarization (Figure S8I and S8J). These results implied that autophagy contributed to M2 polarization of macrophages induced by propionate, but it may function independently of mTOR.

Propionate Reduces Inflammation and Promotes Trophoblast Invasion by Activating AKT Pathway

Our results show that propionate is the major metabolite of *A. muciniphila* and plays important roles in preeclampsia. Considering that placental trophoblasts are critical for spiral artery remodeling, we investigated the effect of propionate on HTR-8/SVneo cells. We found that lipopolysaccharide significantly upregulated the mRNA and protein expression of proinflammatory (TNF-α, MIF [macrophage migration inhibitory factor], and TLR4 [toll-like receptor 4]) and antiangiogenic (*ENG*/sENG) factors. However, propionate dramatically attenuated the above-mentioned effects of lipopolysaccharide (Figure S9A through S9C). Furthermore, propionate counteracted the inhibitory effect of lipopolysaccharide on HTR-8/SVneo cell migration and invasion in a concentration-dependent manner (Figure 8A and 8B). Propionate also significantly upregulated MMP2 and MMP9 expression, which were inhibited by lipopolysaccharide and played key roles in trophoblast invasion (Figure 8C). AKT pathway activation is important to trophoblast invasion. We found that propionate significantly increased the p-AKT/AKT ratio and downregulated the expression of the AKT inhibitory molecule phosphatase and tensin homolog, thereby mitigating the effects of lipopolysaccharide in trophoblasts (Figure 8D; Figure S9D). Moreover, GPR41 siRNA diminished the above effects of propionate except for the downregulation of phosphatase and tensin homolog expression in lipopolysaccharide-treated trophoblasts (Figure 8E through 8G). Additionally, GPR43 and GPR109A siRNA had no significant effect on trophoblast invasion (Figure S9E). These results suggested that propionate activated the AKT signaling through GPR41 and promoted trophoblast invasion.

DISCUSSION

In this study, we revealed the gut dysbiosis in PEs and demonstrated that it is an important etiology of preeclampsia. We found that *A. muciniphila* and propionate/butyrate significantly ameliorated pathologies and symptoms in preeclamptic rats by promoting autophagy and M2 polarization of macrophages in the placenta bed. Moreover, propionate also significantly promoted trophoblast invasion (Figure S10). Intestinal bacteria and their metabolites have important therapeutic and diagnostic potential for preeclampsia.

A. muciniphila, a highly abundant intestinal bacterial species, can produce acetic and propionic acid and provide substrates for butyric acid production by other SCFA-producing bacteria.[22] *A. muciniphila* promotes host immune homeostasis and intestinal barrier integrity.[23] The abundance of intestinal *A. muciniphila* is significantly decreased in many metabolic and inflammatory diseases.[24] Numerous studies have shown that *A. muciniphila* is promising in the treatment of multiple diseases.[23,24] Our study also demonstrated for the first time that *A. muciniphila* could effectively alleviate the symptoms and pathological changes in preeclamptic rats, implying that it has good potential for preeclampsia treatment.

Many studies have demonstrated that SCFAs exert antihypertensive and cardioprotective effects in hypertensive mice through multiple mechanisms, including reducing inflammation and improving intestinal barrier function,[25–27] which is partly dependent on GPCRs (G protein-coupled receptors).[28] The intestinal SCFA-producing bacteria and

Circulation Research FIRST PROOF ONLY

Downloaded from http://ahajournals.org by on August 11, 2022

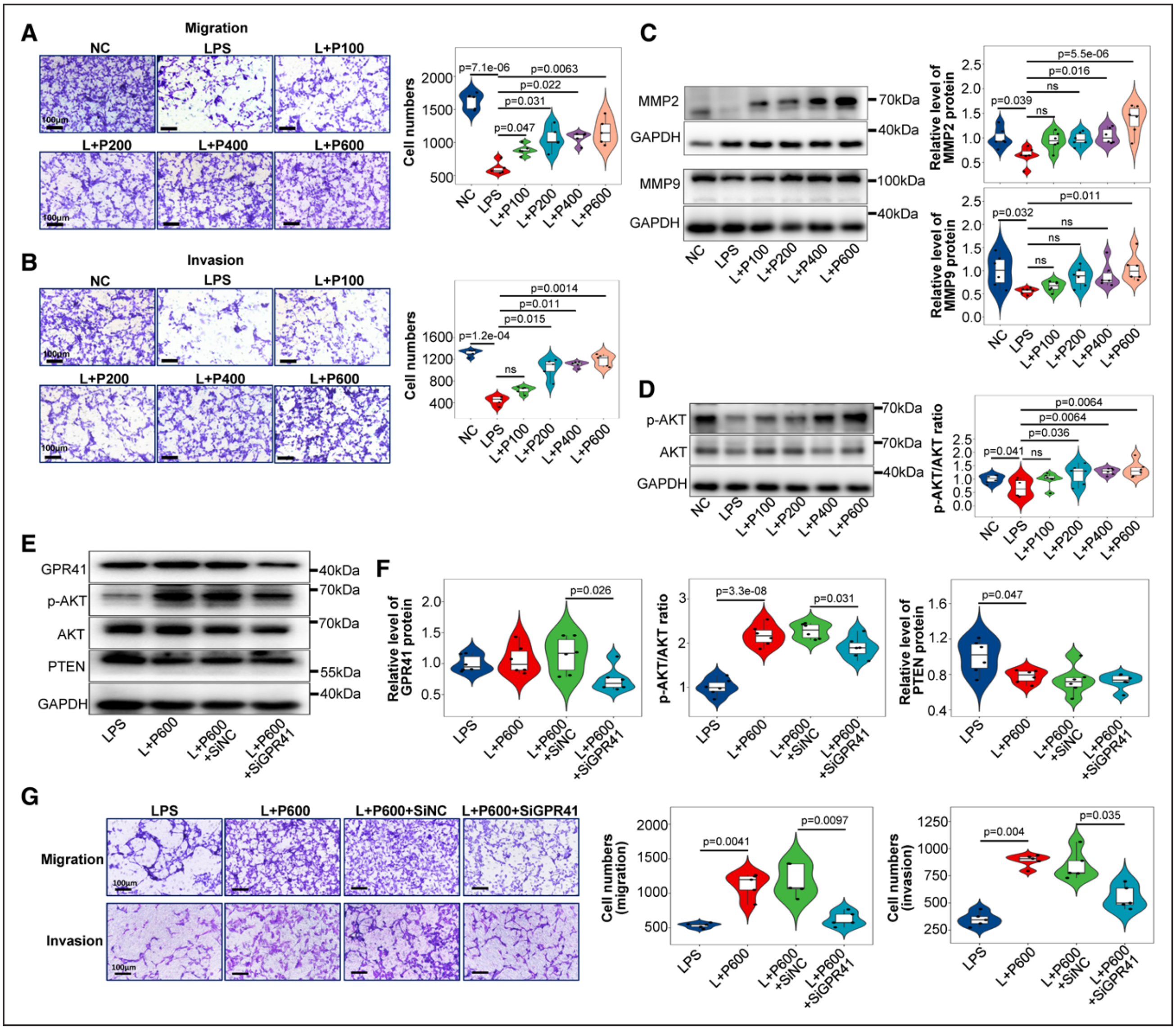

Figure 8. Effects of propionate on migration and invasion of trophoblasts.

A and **B**, Representative images of migration (**A**) and invasion (**B**) of HTR-8/SVneo cells with different treatments (left) and comparison of their levels (right. n=5. **C**, Representative Western blot images of MMP2 and MMP9 in each group of HTR-8/SVneo cells (left) and comparison of their relative levels compared with GAPDH protein (right). n=6. **D**, Representative Western blot images of phosphorylated (p)-AKT and AKT in each group of HTR-8/SVneo cells (left) and comparison of p-AKT/AKT ratio (right). n=6. **E** and **F**, Representative Western blot images of GPR41, p-AKT, AKT, and PTEN (**E**) in each group of HTR-8/SVneo cells and comparison of the ratio of p-AKT/AKT and the relative levels of GPR41 and PTEN compared with GAPDH protein (**F**). n=6. **G**, Representative images of migration and invasion of HTR-8/SVneo cells with different treatments (left) and comparisons of their levels (right). n=5. Data are presented as median and IQR in **A**–**D**, **F**, and **G**. One-way ANOVA with Tukey's post hoc test was used for statistical analysis in **C** and **F**. Kruskal-Wallis test with FDR's post hoc test was used for statistical analysis in **A**, **B**, **D**, and **G**. ns: not significant. NC: blank control; LPS: LPS treatment (100 ng/ml); L+P100: LPS and sodium propionate (100 μmol/L) treatment; L+P200: LPS and sodium propionate (200 μmol/L) treatment; L+P400: LPS and sodium propionate (400 μmol/L) treatment; L+P600: LPS and sodium propionate (600 μmol/L) treatment; L+P600+SiNC (L+P600+SiGPR41): LPS, sodium propionate (600 μmol/L), and negative control of siRNA (GPR41 SiRNA) treatment.

SCFAs are also reported to be associated with placental function and fetal development.[7,29] A recent study found that butyrate regulated inflammatory and angiogenic factors in the placenta by modulating gut microbiota, thereby reducing the BP of preeclamptic rats.[19] However, butyrate was also reported not to modulate gut microbiota.[30] Our study suggests that both butyrate and propionate exerted anti-inflammatory effects by promoting M2 polarization and/or autophagy of macrophage in the placenta bed. Additionally, propionate significantly inhibits the expression of antiangiogenic and inflammatory factors in trophoblasts and promotes trophoblast invasion. Moreover, our results demonstrated that GPR41 siRNA did not have a significant effect on phosphatase and tensin homolog expression inhibited by propionate, suggesting that propionate may regulate phosphatase and tensin

Downloaded from http://ahajournals.org by on August 11, 2022

homolog expression through other receptor or nonreceptor mechanisms. Obviously, the roles and mechanisms of SCFAs in placental development and function are diverse and require further in-depth studies.

Studies have indicated that SCFAs modulate various cytokines expression in macrophages through GPCRs and have anti-inflammatory effects.[31] Butyrate can promote M2 polarization while inhibiting lipopolysaccharide-induced M1 polarization.[32,33] Our study showed that in addition to promoting M2 polarization, SCFAs enhance macrophage autophagy, thereby inhibiting macrophage inflammation. Nevertheless, due to the complexity of macrophage types and functions, more comprehensive studies on the regulation of macrophages in the placenta by SCFAs and their mechanisms are still needed.

Liu et al reported significant abnormalities in the gut microbiota of PEs. The low serum levels of acetate and butyrate were associated with preeclampsia development.[14,16,34,35] The probiotics and butyrate have also been found to reduce BP in preeclamptic rats.[18,36] A recent study reported that the gut microbiota of PEs significantly increased BP in nonpregnant mice and gut barrier damage allowed intestinal bacteria to colonize the placenta, which led to preeclampsia.[20] However, the animal model used in that study had already developed hypertension before pregnancy, so it did not meet the definition of preeclampsia according to the NICE guidelines. Thus, that animal experiments demonstrated the influence of gut microbiota on the BP of pregnant mice with hypertension but not preeclampsia.

Additionally, there is still intense debate whether gut bacteria could transfer to placenta through the circulation. Some studies suggest that human placenta has a distinct microbiota. Bacteria associated with gastrointestinal and respiratory infections, or periodontitis have been reported to be present in the placenta of PEs.[37] However, many studies demonstrated that the reported bacteria in placenta should originate from the contaminants of the experimental or delivery process.[21,38] Marcus et al[21] analyzed placental tissues from 537 pregnant women using various methods and found that bacterial sequences in placental DNA were very low and mainly originated from contamination of reagents and equipment. Microorganisms different from those in negative controls were also not detected in the placenta of other 160 pregnant women.[39] Most importantly, the sterile animals obtained by cesarean section strongly support the view that the placenta is sterile. Our findings also suggest that the multiple bacteria previously reported to be present in the placenta are more likely to be derived from the contamination of experimental reagents.

To date, there is still a lack of effective treatment for preeclampsia other than pregnancy termination. A high intake of probiotics was reported to be significantly associated with a low risk of preeclampsia.[13] We found that oral administration of *A. muciniphila*, propionate, or butyrate significantly decreased BP and improved placental function and embryonic development in preeclamptic rats. Since *A. muciniphila* and propionate/butyrate normally exist in human intestine, their safety is assured, allowing them great potential for preeclampsia treatments. Additionally, the early screening and diagnosis of preeclampsia is difficult due to the lack of specific indicators.[40] In the present study, we identified a marker set consisting of SCFAs and intestinal bacteria for preeclampsia diagnosis, which deserves more in-depth study.

In conclusion, our study reveals gut dysbiosis of preeclamptic patients that contribute to preeclampsia development. We demonstrated the great potential of *A. muciniphila* and propionate/butyrate for preeclampsia treatments and the underlying mechanism. Additionally, we identified a marker set consisting of intestinal bacteria and SCFAs with potential for preeclampsia diagnosis. Our findings enrich the gut–placenta axis theory and contribute to developing microecological products for the diagnosis and treatment of preeclampsia. Nevertheless, it should be noted that the diagnostic and therapeutic potential of *Akkermansia* and SCFAs need to be validated in larger patient cohorts. Moreover, except for bacteria, the roles of intestinal fungi and viruses in preeclampsia are also worth investigating using shotgun metagenomic sequencing.

Circulation Research FIRST PROOF ONLY

ARTICLE INFORMATION

Received January 12, 2022; revision received July 24, 2022; accepted July 27, 2022.

Affiliations

Key Laboratory of Cardiovascular Remodeling and Function Research, Chinese Ministry of Education, Chinese National Health Commission and Chinese Academy of Medical Sciences (J.J., Y.Z., Z.Z., Z.T., X.W., C.Z., Q.Z.). State and Shandong Province Joint Key Laboratory of Translational Cardiovascular Medicine (J.J., Y.Z., Z.Z., Z.T., X.W., C.Z., Q.Z.). Department of Cardiology, Qilu Hospital of Shandong University, Jinan, China (J.J., Y.Z., Z.Z., Z.T., X.W., C.Z., Q.Z.). Division of Geriatrics, Department of Endocrinology, Shandong Provincial Hospital, Shandong University, Jinan, China (L.G., J.L., J.G., Z.W.). Cardiovascular Disease Research Center of Shandong First Medical University, Central Hospital Affiliated to Shandong First Medical University, Jinan, China (Q.Z.). Intensive Care Unit, Liaocheng People's Hospital, China (X.Z., T.W.). Maternal and child health care hospital of Shandong province, Shandong University, Jinan, China (L.G.). Department of Biology, University College London, United Kingdom (X.Z.).

Acknowledgments

The authors would like to convey heartfelt gratitude to the participants for their contribution.

Sources of Funding

This work was financially supported by the National Key R&D Program of China (2021YFA1301102), National Natural Science Foundation of China (82070820 and 82170495) and Major Science and Technology Innovation Project of Shandong Province (2018CXGC1218).

Disclosures

None.

Supplemental Material

Supplemental Methods
References[41–52]
Tables S1–S3
Figure S1–S10

Downloaded from http://ahajournals.org by on August 11, 2022

ORIGINAL RESEARCH

REFERENCES

1. Belay AS, Wudad T. Prevalence and associated factors of pre-eclampsia among pregnant women attending anti-natal care at Mettu Karl referal hospital, Ethiopia: cross-sectional study. *Clin Hypertens*. 2019;25:14. doi: 10.1186/s40885-019-0120-1
2. Brown MC, Best KE, Pearce MS, Waugh J, Robson SC, Bell R. Cardiovascular disease risk in women with pre-eclampsia: systematic review and meta-analysis. *Eur J Epidemiol*. 2013;28:1–19. doi: 10.1007/s10654-013-9762-6
3. Warshafsky C, Pudwell J, Walker M, Wen SW, Smith GN; Preeclampsia New Emerging Team. Prospective assessment of neurodevelopment in children following a pregnancy complicated by severe pre-eclampsia. *BMJ Open*. 2016;6:e010884. doi: 10.1136/bmjopen-2015-010884
4. Ali SM, Khalil RA. Genetic, immune and vasoactive factors in the vascular dysfunction associated with hypertension in pregnancy. *Expert Opin Ther Targets*. 2015;19:1495–1515. doi: 10.1517/14728222.2015.1067684
5. Sun M, Wu W, Liu Z, Cong Y. Microbiota metabolite short chain fatty acids, GPCR, and inflammatory bowel diseases. *J Gastroenterol*. 2017;52:1–8. doi: 10.1007/s00535-016-1242-9
6. Chang PV, Hao L, Offermanns S, Medzhitov R. The microbial metabolite butyrate regulates intestinal macrophage function via histone deacetylase inhibition. *Proc Natl Acad Sci U S A*. 2014;111:2247–2252. doi: 10.1073/pnas.1322269111
7. Wallace JG, Bellissimo CJ, Yeo E, Fei Xia Y, Petrik JJ, Surette MG, Bowdish DME, Sloboda DM. Obesity during pregnancy results in maternal intestinal inflammation, placental hypoxia, and alters fetal glucose metabolism at mid-gestation. *Sci Rep*. 2019;9:17621. doi: 10.1038/s41598-019-54098-x
8. Dowling O, Chatterjee PK, Gupta M, Tam Tam HB, Xue X, Lewis D, Rochelson B, Metz CN. Magnesium sulfate reduces bacterial LPS-induced inflammation at the maternal-fetal interface. *Placenta*. 2012;33:392–398. doi: 10.1016/j.placenta.2012.01.013
9. Kang Y, Cai Y. Gut microbiota and hypertension: from pathogenesis to new therapeutic strategies. *Clin Res Hepatol Gastroenterol*. 2018;42:110–117. doi: 10.1016/j.clinre.2017.09.006
10. Maifeld A, Bartolomaeus H, Löber U, Avery EG, Steckhan N, Markó L, Wilck N, Hamad I, Šušnjar U, Mähler A, et al. Fasting alters the gut microbiome reducing blood pressure and body weight in metabolic syndrome patients. *Nat Commun*. 2021;12:1970. doi: 10.1038/s41467-021-22097-0
11. Wilck N, Matus MG, Kearney SM, Olesen SW, Forslund K, Bartolomaeus H, Haase S, Mähler A, Balogh A, Markó L, et al. Salt-responsive gut commensal modulates TH17 axis and disease. *Nature*. 2017;551:585–589. doi: 10.1038/nature24628
12. Yan X, Jin J, Su X, Yin X, Gao J, Wang X, Zhang S, Bu P, Wang M, Zhang Y, et al. Intestinal flora modulates blood pressure by regulating the synthesis of intestinal-derived corticosterone in high salt-induced hypertension. *Circ Res*. 2020;126:839–853. doi: 10.1161/CIRCRESAHA.119.316394
13. Brantsaeter AL, Myhre R, Haugen M, Myking S, Sengpiel V, Magnus P, Jacobsson B, Meltzer HM. Intake of probiotic food and risk of preeclampsia in primiparous women: the norwegian mother and child cohort study. *Am J Epidemiol*. 2011;174:807–815. doi: 10.1093/aje/kwr168
14. Liu J, Yang H, Yin Z, Jiang X, Zhong H, Qiu D, Zhu F, Li R. Remodeling of the gut microbiota and structural shifts in Preeclampsia patients in South China. *Eur J Clin Microbiol Infect Dis*. 2017;36:713–719. doi: 10.1007/s10096-016-2853-z
15. Lv LJ, Li SH, Li SC, Zhong ZC, Duan HL, Tian C, Li H, He W, Chen MC, He TW, et al. Early-onset preeclampsia is associated with gut microbial alterations in antepartum and postpartum women. *Front Cell Infect Microbiol*. 2019;9:224. doi: 10.3389/fcimb.2019.00224
16. Wang J, Gu X, Yang J, Wei Y, Zhao Y. Gut microbiota dysbiosis and increased plasma LPS and TMAO levels in patients with preeclampsia. *Front Cell Infect Microbiol*. 2019;9:409. doi: 10.3389/fcimb.2019.00409
17. Gomez-Arango LF, Barrett HL, McIntyre HD, Callaway LK, Morrison M, Dekker Nitert M; SPRING Trial Group. Increased systolic and diastolic blood pressure is associated with altered gut microbiota composition and butyrate production in early pregnancy. *Hypertension*. 2016;68:974–981. doi: 10.1161/HYPERTENSIONAHA.116.07910
18. Chang Y, Chen Y, Zhou Q, Wang C, Chen L, Di W, Zhang Y. Short-chain fatty acids accompanying changes in the gut microbiome contribute to the development of hypertension in patients with preeclampsia. *Clin Sci (Lond)*. 2020;134:289–302. doi: 10.1042/CS20191253
19. Yong W, Zhao Y, Jiang X, Li P. Sodium butyrate alleviates pre-eclampsia in pregnant rats by improving the gut microbiota and short-chain fatty acid metabolites production. *J Appl Microbiol*. 2022;132:1370–1383. doi: 10.1111/jam.15279
20. Chen X, Li P, Liu M, Zheng H, He Y, Chen MX, Tang W, Yue X, Huang Y, Zhuang L, et al. Gut dysbiosis induces the development of pre-eclampsia through bacterial translocation. *Gut*. 2020;69:513–522. doi: 10.1136/gutjnl-2019-319101
21. de Goffau MC, Lager S, Sovio U, Gaccioli F, Cook E, Peacock SJ, Parkhill J, Charnock-Jones DS, Smith GCS. Human placenta has no microbiome but can contain potential pathogens. *Nature*. 2019;572:329–334. doi: 10.1038/s41586-019-1451-5
22. Zhai Q, Feng S, Arjan N, Chen W. A next generation probiotic, Akkermansia muciniphila. *Crit Rev Food Sci Nutr*. 2019;59:3227–3236. doi: 10.1080/10408398.2018.1517725
23. Everard A, Belzer C, Geurts L, Ouwerkerk JP, Druart C, Bindels LB, Guiot Y, Derrien M, Muccioli GG, Delzenne NM, et al. Cross-talk between Akkermansia muciniphila and intestinal epithelium controls diet-induced obesity. *Proc Natl Acad Sci U S A*. 2013;110:9066–9071. doi: 10.1073/pnas.1219451110
24. Zhang T, Ji X, Lu G, Zhang F. The potential of Akkermansia muciniphila in inflammatory bowel disease. *Appl Microbiol Biotechnol*. 2021;105:5785–5794. doi: 10.1007/s00253-021-11453-1
25. Marques FZ, Nelson E, Chu PY, Horlock D, Fiedler A, Ziemann M, Tan JK, Kuruppu S, Rajapakse NW, El-Osta A, et al. High-fiber diet and acetate supplementation change the gut microbiota and prevent the development of hypertension and heart failure in hypertensive mice. *Circulation*. 2017;135:964–977. doi: 10.1161/CIRCULATIONAHA.116.024545
26. Bartolomaeus H, Balogh A, Yakoub M, Homann S, Markó L, Höges S, Tsvetkov D, Krannich A, Wundersitz S, Avery EG, et al. Short-Chain fatty acid propionate protects from hypertensive cardiovascular damage. *Circulation*. 2019;139:1407–1421. doi: 10.1161/CIRCULATIONAHA.118.036652
27. Kim S, Goel R, Kumar A, Qi Y, Lobaton G, Hosaka K, Mohammed M, Handberg EM, Richards EM, Pepine CJ, et al. Imbalance of gut microbiome and intestinal epithelial barrier dysfunction in patients with high blood pressure. *Clin Sci (Lond)*. 2018;132:701–718. doi: 10.1042/CS20180087
28. Kaye DM, Shihata WA, Jama HA, Tsyganov K, Ziemann M, Kiriazis H, Horlock D, Vijay A, Giam B, Vinh A, et al. Deficiency of prebiotic fiber and insufficient signaling through gut metabolite-sensing receptors leads to cardiovascular disease. *Circulation*. 2020;141:1393–1403. doi: 10.1161/CIRCULATIONAHA.119.043081
29. Voltolini C, Battersby S, Etherington SL, Petraglia F, Norman JE, Jabbour HN. A novel antiinflammatory role for the short-chain fatty acids in human labor. *Endocrinology*. 2012;153:395–403. doi: 10.1210/en.2011-1457
30. Biagi G, Piva A, Moschini M, Vezzali E, Roth FX. Performance, intestinal microflora, and wall morphology of weanling pigs fed sodium butyrate. *J Anim Sci*. 2007;85:1184–1191. doi: 10.2527/jas.2006-378
31. Parada VD, De la Fuente MK, Landskron G, Gonzalez MJ, Quera R, Dijkstra G, Harmsen H, Faber KN, Hermoso MA. Short Chain Fatty Acids (SCFAs)-mediated gut epithelial and immune regulation and its relevance for inflammatory bowel diseases. *Front Immunol*. 2019;10:277. doi: 10.3389/fimmu.2019.00277
32. Ji J, Shu D, Zheng M, Wang J, Luo C, Wang Y, Guo F, Zou X, Lv X, Li Y, et al. Microbial metabolite butyrate facilitates M2 macrophage polarization and function. *Sci Rep*. 2016;6:24838. doi: 10.1038/srep24838
33. Jiang X, Huang X, Tong Y, Gao H. Butyrate improves cardiac function and sympathetic neural remodeling following myocardial infarction in rats. *Can J Physiol Pharmacol*. 2020;98:391–399. doi: 10.1139/cjpp-2019-0531
34. Hu M, Eviston D, Hsu P, Mariño E, Chidgey A, Santner-Nanan B, Wong K, Richards JL, Yap YA, Collier F, et al; BIS Investigator Group. Decreased maternal serum acetate and impaired fetal thymic and regulatory T cell development in preeclampsia. *Nat Commun*. 2019;10:3031. doi: 10.1038/s41467-019-10703-1
35. Altemani F, Barrett HL, Gomez-Arango L, Josh P, David McIntyre H, Callaway LK, Morrison M, Tyson GW, Dekker Nitert M. Pregnant women who develop preeclampsia have lower abundance of the butyrate-producer Coprococcus in their gut microbiota. *Pregnancy Hypertens*. 2021;23:211–219. doi: 10.1016/j.preghy.2021.01.002
36. Sun BM, Meng L, Liu H, Bao D. Changes in intestinal flora in preeclampsia rats and effects of probiotics on their inflammation and blood pressure. *Eur Rev Med Pharmacol Sci*. 2020;24:10155–10161. doi: 10.26355/eurrev_202010_23235
37. Moreno I, Codoñer FM, Vilella F, Valbuena D, Martinez-Blanch JF, Jimenez-Almazán J, Alonso R, Alamá P, Remohí J, Pellicer A, et al. Evidence that the endometrial microbiota has an effect on implantation success or failure. *Am J Obstet Gynecol*. 2016;215:684–703. doi: 10.1016/j.ajog.2016.09.075
38. Theis KR, Romero R, Winters AD, Greenberg JM, Gomez-Lopez N, Alhousseini A, Bieda J, Maymon E, Pacora P, Fettweis JM, et al. Does

Circulation Research FIRST PROOF ONLY

Downloaded from http://ahajournals.org by on August 11, 2022

the human placenta delivered at term have a microbiota? Results of cultivation, quantitative real-time PCR, 16S rRNA gene sequencing, and metagenomics. *Am J Obstet Gynecol*. 2019;220:267.e1–267.e39. doi: 10.1016/j.ajog.2018.10.018

39. Leiby JS, McCormick K, Sherrill-Mix S, Clarke EL, Kessler LR, Taylor LJ, Hofstaedter CE, Roche AM, Mattei LM, Bittinger K, et al. Lack of detection of a human placenta microbiome in samples from preterm and term deliveries. *Microbiome*. 2018;6:196. doi: 10.1186/s40168-018-0575-4
40. Kenny LC, Black MA, Poston L, Taylor R, Myers JE, Baker PN, McCowan LM, Simpson NA, Dekker GA, Roberts CT, et al. Early pregnancy prediction of preeclampsia in nulliparous women, combining clinical risk and biomarkers: the Screening for Pregnancy Endpoints (SCOPE) international cohort study. *Hypertension*. 2014;64:644–652. doi: 10.1161/HYPERTENSIONAHA.114.03578
41. Zhao WH, Huang ZP, Zhang X, He L, Willett W, Wang JL, Hasegawa K, Chen JS. Reproducibility and validity of a Chinese food frequency questionnaire. *Biomed Environ Sci*. 2010;23:1–38.
42. Phipps EA, Thadhani R, Benzing T, Karumanchi SA. Pre-eclampsia: pathogenesis, novel diagnostics and therapies. *Nat Rev Nephrol*. 2019;15:275–289. doi: 10.1038/s41581-019-0119-6
43. Marques FZ, Jama HA, Tsyganov K, Gill PA, Rhys-Jones D, Muralitharan RR, Muir J, Holmes A, Mackay CR. Guidelines for transparency on gut microbiome studies in essential and experimental hypertension. *Hypertension*. 2019;74:1279–1293. doi: 10.1161/HYPERTENSIONAHA.119.13079
44. Mirzayi C, Renson A, Zohra F, Elsafoury S, Geistlinger L, Kasselman LJ, Eckenrode K, van de Wijgert J, Loughman A, Marques FZ, et al; Genomic Standards Consortium; Massive Analysis and Quality Control Society. Reporting guidelines for human microbiome research: the STORMS checklist. *Nat Med*. 2021;27:1885–1892. doi: 10.1038/s41591-021-01552-x
45. Sheth RU, Li M, Jiang W, Sims PA, Leong KW, Wang HH. Spatial metagenomic characterization of microbial biogeography in the gut. *Nat Biotechnol*. 2019;37:877–883. doi: 10.1038/s41587-019-0183-2
46. Segata N, Izard J, Waldron L, Gevers D, Miropolsky L, Garrett WS, Huttenhower C. Metagenomic biomarker discovery and explanation. *Genome Biol*. 2011;12:R60. doi: 10.1186/gb-2011-12-6-r60
47. Roederer M. Compensation in flow cytometry. *Curr Protoc Cytom*. 2002;Chapter 1:Unit 1.14. doi: 10.1002/0471142956.cy0114s22
48. Gheorghe CE, Ritz NL, Martin JA, Wardill HR, Cryan JF, Clarke G. Investigating causality with fecal microbiota transplantation in rodents: applications, recommendations and pitfalls. *Gut Microbes*. 2021;13:1941711. doi: 10.1080/19490976.2021.1941711
49. Costello SP, Hughes PA, Waters O, Bryant RV, Vincent AD, Blatchford P, Katsikeros R, Makanyanga J, Campaniello MA, Mavrangelos C, et al. Effect of fecal microbiota transplantation on 8-week remission in patients with ulcerative colitis: a randomized clinical trial. *JAMA*. 2019;321:156–164. doi: 10.1001/jama.2018.20046
50. Secombe KR, Al-Qadami GH, Subramaniam CB, Bowen JM, Scott J, Van Sebille YZA, Snelson M, Cowan C, Clarke G, Gheorghe CE, et al. Guidelines for reporting on animal fecal transplantation (GRAFT) studies: recommendations from a systematic review of murine transplantation protocols. *Gut Microbes*. 2021;13:1979878. doi: 10.1080/19490976.2021.1979878
51. Kumasawa K. Animal models in preeclampsia. *Preeclampsia*. 2018;141–155.
52. Kräker K, O'Driscoll JM, Schütte T, Herse F, Patey O, Golic M, Geisberger S, Verlohren S, Birukov A, Heuser A, et al. Statins reverse postpartum cardiovascular dysfunction in a rat model of preeclampsia. *Hypertension*. 2020;75:202–210. doi: 10.1161/HYPERTENSIONAHA.119.13219

American Heart Association.

Circulation Research

FIRST PROOF ONLY

Downloaded from http://ahajournals.org by on August 11, 2022
