# Supplementary material for: Assessment of medical information on irritable bowel syndrome information in Wikipedia and Baidu Encyclopedia: comparative study
Source: PeerJ. 2024 May 24;12:e17264. doi: 10.7717/peerj.17264 (PMC11129691; doi:10.7717/peerj.17264)
Supplement: Data S1 [file peerj-12-17264-s001.zip › σÄƒσoïμò░μì«/Wikipedia/Indigestion - Wikipedia.pdf]

WIKIPEDIA

Indigestion

Indigestion	
Other names	Dyspepsia
Specialty	Gastroenterology
Symptoms	Upper abdominal pain[1]
Frequency	Common[1]

Indigestion, also known as **dyspepsia** or **upset stomach**, is a condition of impaired digestion.[2] Symptoms may include upper abdominal fullness, heartburn, nausea, belching, or upper abdominal pain.[3] People may also experience feeling full earlier than expected when eating.[4] Indigestion is relatively common, affecting 20% of people at some point during their life, and is frequently caused by gastroesophageal reflux disease (GERD) or gastritis.[1][5]

Indigestion is subcategorized as "organic" or "functional", but making the diagnosis can prove challenging for physicians.[6] Organic indigestion is the result of an underlying disease, such as gastritis, peptic ulcer disease (an ulcer of the stomach or duodenum), or cancer.[6] Functional indigestion (previously called nonulcer dyspepsia)[7] is indigestion without evidence of underlying disease.[8] Functional indigestion is estimated to affect about 15% of the general population in western countries and accounts for a majority of dyspepsia cases.[7][9]

In elderly patients (60 years of age or older) or with worrisome symptoms such as trouble swallowing, weight loss, or blood loss, an endoscopy (a procedure whereby a camera attached to a flexible tube is inserted down the throat and into the stomach) is recommended to further assess and find a potential cause.[1] In patients younger than 60 years of age, testing for the bacteria *H. pylori* and if positive, treatment of the infection is recommended.[1] More details about how indigestion is diagnosed and treated can be found below.

Contents

- Signs and symptoms
  - Symptoms
  - Signs
  - Alarm symptoms
- Cause
  - Organic Dyspepsia
    - Esophagitis
    - Gastritis
      - Peptic Ulcer Disease
        - *Helicobacter pylori* (*H.pylori)* infection
    - Pancreatobiliary Disease
    - Duodenal micro-inflammation
  - Functional Dyspepsia
    - Epigastric Pain Syndrome (EPS)
    - Post-Prandial Distress Syndrome (PDS)
  - Food, herb, or drug intolerance

Systemic Diseases
Post-infectious Causes of Dyspepsia
Pathophysiology
Diagnosis
Treatment
Diet
Acid suppression
Prokinetics
Alternative medicine
Epidemiology
See also
References
External links

Signs and symptoms

Symptoms

Patients experiencing indigestion likely report one, a combination of, or all of the following symptoms:[6][10]

- upper abdominal pain or discomfort
- bloating
- early satiety
- postprandial fullness
- nausea with or without vomiting
- anorexia
- regurgitation
- belching

Signs

There may be abdominal tenderness, but this finding is nonspecific and is not required to make a diagnosis.[10] However, there are physical exam signs that may point to a different diagnosis and underlying cause for a patient's reported discomfort. A positive Carnett sign (focal tenderness that increases with abdominal wall contraction and palpation) suggests an etiology involving the abdominal wall musculature. Cutaneous dermatomal distribution of pain may suggest a thoracic polyradiculopathy. Tenderness to palpation over the right upper quadrant, or Murphy's sign, may suggest cholecystitis or gallbladder inflammation.[11]

Alarm symptoms

Also known as *Alarm features*, *alert features*, *red flags*, or *warning signs* in gastrointestinal (GI) literature.

Alarm features are thought to be associated with serious gastroenterologic disease and include:[12]

- chronic gastrointestinal bleeding
- progressive unintentional weight loss
- progressive difficulty swallowing (dysphagia)
- persistent vomiting
- Iron deficiency anemia
- Vitamin B_{12} deficiency (Pernicious anemia)
- epigastric mass

Cause

Indigestion is a diagnosis related to a combination of symptoms that can be attributed to "organic" or "functional" causes.[13] Organic dyspepsia should have pathological findings upon endoscopy, like an ulcer in the stomach lining in peptic ulcer disease.[13] Functional dyspepsia is unlikely to be detected on endoscopy but can be broken down into two subtypes, epigastric pain syndrome (EPS) and post-prandial distress syndrome (PDS).[14] In addition, indigestion could be caused by medications, food, or other disease processes.

Psychosomatic and cognitive factors are important in the evaluation of people with chronic dyspepsia. Studies have shown a high occurrence of mental disorders, notably anxiety and depression, amongst patients with dyspepsia; however, there is little evidence to prove causation.[15]

Organic Dyspepsia

Esophagitis

Esophagitis is an inflammation of the esophagus, most commonly caused by gastroesophageal reflux disease (GERD).[6] It is defined by the sensation of "heartburn" or a burning sensation in the chest as a result of inappropriate relaxation of the lower esophageal sphincter at the site where the esophagus connects to the stomach. It is often treated with proton pump inhibitors. If left untreated, the chronic damage to the esophageal tissues poses a risk of developing cancer.[6] A meta-analysis showed risk factors for developing GERD included age equal to or greater than 50, smoking, the use of non-steroid anti-inflammatory medications, and obesity.[16]

Gastritis

Common causes of gastritis include peptic ulcer disease, infection, or medications.

Peptic Ulcer Disease

Gastric and/or duodenal ulcers are the defining feature of peptic ulcer disease (PUD). PUD is most commonly caused by an infection with H. pylori or NSAID use.[17]

Helicobacter pylori (*H.pylori)* infection

The role of *H. pylori* in functional dyspepsia is controversial, and treatment for *H. pylori* may not lead to complete improvement of a patient's dyspepsia.[6] However, a recent systemic review and meta-analysis of 29 studies published in 2022 suggests that successful treatment of *H. pylori* modestly improves indigestion symptoms.[18]

Pancreatobiliary Disease

These include cholelithiasis, chronic pancreatitis, and pancreatic cancer.

Duodenal micro-inflammation

Duodenal micro-inflammation caused by an altered duodenal gut microbiota, reactions to foods (mainly gluten proteins) or infections may induce dyspepsia symptoms in a subset of people.[19]

Functional Dyspepsia

Functional dyspepsia is a common cause of chronic heartburn. More than 70% of people have no obvious organic cause for their symptoms after evaluation.[13] Symptoms may arise from a complex interaction of increased visceral afferent sensitivity, gastric delayed emptying (gastroparesis) or impaired accommodation to food. Diagnostic criteria for functional dyspepsia categorize it into two subtypes by symptom: epigastric pain syndrome and post-prandial distress syndrome.[14] Anxiety is also associated with functional dyspepsia. In some people, it appears before the onset of gut symptoms; in other cases, anxiety develops after onset of the disorder, which suggests that a gut-driven brain disorder may be a possible cause.[14] Although benign, these symptoms may be chronic and difficult to treat.[20]

Epigastric Pain Syndrome (EPS)

Defined by stomach pain and/or burning that interferes with daily life, without any evidence of organic disease.[21]

Post-Prandial Distress Syndrome (PDS)

Defined by post-prandial fullness or early satiation that interferes with daily life, without any evidence of organic disease.[21]

Food, herb, or drug intolerance

Acute, self-limited dyspepsia may be caused by overeating, eating too quickly, eating high-fat foods, eating during stressful situations, or drinking too much alcohol or coffee. Many medications cause dyspepsia, including aspirin, nonsteroidal anti-inflammatory drugs (NSAIDs), antibiotics (metronidazole, macrolides), bronchodilators (theophylline), diabetes drugs (acarbose, metformin, Alpha-glucosidase inhibitor, amylin analogs, GLP-1 receptor antagonists), antihypertensive medications (angiotensin converting enzyme [ACE] inhibitors, Angiotensin II receptor antagonist),

cholesterol-lowering agents (niacin, fibrates), neuropsychiatric medications (cholinesterase inhibitors [donepezil, rivastigmine]), SSRIs (fluoxetine, sertraline), serotonin-norepinephrine-reuptake inhibitors (venlafaxine, duloxetine), Parkinson drugs (Dopamine agonist, monoamine oxidase [MAO]-B inhibitors), weight-loss medications (orlistat), corticosteroids, estrogens, digoxin, iron, and opioids.[22][23] Common herbs have also been show to cause indigestion, like white willow berry, garlic, ginkgo, chaste tree berry, saw palmetto, and feverfew.[22] Studies have shown that wheat and dietary fats can contribute to indigestion and suggest foods high in short-chain carbohydrates (FODMAP) may be associated with dyspepsia.[24] This suggests reducing or consuming a gluten-free, low-fat, and/or FODMAP diet may improve symptoms.[24][25] Additionally, some people may experience dyspepsia when eating certain spices or spicy food as well as foods like peppers, chocolate, citrus, and fish.[6]

Systemic Diseases

There are a number of systemic diseases that may involve dyspepsia, including coronary disease, congestive heart failure, diabetes mellitus, hyperparathyroidism, thyroid disease, and chronic kidney disease.

Post–infectious Causes of Dyspepsia

Gastroenteritis increases the risk of developing chronic dyspepsia. Post-infectious dyspepsia is the term given when dyspepsia occurs after an acute gastroenteritis infection. It is believed that the underlying causes of post-infectious IBS and post-infectious dyspepsia may be similar and represent different aspects of the same pathophysiology.[26]

Pathophysiology

The pathophysiology for indigestion is not well understood; however, there are many theories. For example, there are studies that suggest a gut-brain interaction, as patients who received an antibiotic saw a reduction in their indigestion symptoms.[27] Other theories propose issues with gut motility, a hypersensitivity of gut viscera, and imbalance of the microbiome.[9] A genetic predisposition is plausible, but there is limited evidence to support this theory.[28]

Diagnosis

A diagnosis for indigestion is based on symptoms, with a possible need for more diagnostic tests. In younger patients (less than 60 years of age) without red flags (e.g., weight loss), it is recommended to test for H. pylori noninvasively, followed by treatment with antibiotics in those who test positively. A negative test warrants discussing additional treatments, like proton pump inhibitors, with your doctor.[1] An upper GI endoscopy may also be recommended.[29] In older patients (60 or older), an endoscopy is often the next step in finding out the cause of newly onset indigestion regardless of the presence of

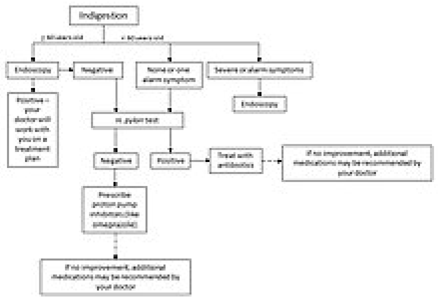

Simplified diagram of how indigestion is diagnosed and treatment(s) determined

alarm symptoms.[1] However, for all patients regardless of age, an official diagnosis requires symptoms to have started at least 6 months ago with a frequency of at least once a week over the last 3 months.[10]

Treatment

Functional and organic dyspepsia have similar treatments. Traditional therapies used for this diagnosis include lifestyle modification (e.g., diet), antacids, proton-pump inhibitors (PPIs), H_2-receptor antagonists (H2-RAs), prokinetic agents, and antiflatulents. PPIs and H2-RAs are often first-line therapies for treating dyspepsia, having shown to be better than placebo medications.[30] Anti-depressants, notably tricyclic antidepressants, have also been shown to be effective treatments for patients who do not respond to traditional therapies.[30]

Diet

A lifestyle change that may help with indigestion is a change in diet, such as a stable and consistent eating schedule and slowing the pace of eating.[31] Additionally, there are studies that support a reduction in the consumption of fats may also alleviate dyspepsia.[31] While some studies suggest a correlation between dyspepsia and celiac disease, not everyone with indigestion needs to refrain from gluten in their diet. However, a gluten-free diet can relieve the symptoms in some patients without celiac disease.[19][31] Lastly, a FODMAPs diet or diet low/free from certain complex sugars and sugar alcohols has also been shown to be potentially beneficial in patients with indigestion.[31]

Acid suppression

Proton pump inhibitors (PPIs) were found to be better than placebo in a literature review, especially when looking at long-term symptom reduction.[32][33] H2 receptor antagonists (H2-RAs) have similar effect on symptoms reduction when compared to PPIs.[32] However, there is little evidence to support prokinetic agents are an appropriate treatment for dyspepsia.[34]

Currently, PPIs are FDA indicated for erosive esophagitis, gastroesophageal reflux disease (GERD), Zollinger-Ellison syndrome, eradication of H. pylori, duodenal and gastric ulcers, and NSAID-induced ulcer healing and prevention, but not functional dyspepsia.[35]

Prokinetics

Prokinetics (medications focused on increasing gut motility), such as metoclopramide or erythromycin, has a history of use as a secondary treatment for dyspepsia.[6] While multiple studies show that it is more effective than placebo, there are multiple concerns about the side effects surrounding the long-term use of these medications.[6]

Alternative medicine

A 2021 meta-analysis concluded that herbal remedies, like menthacarin (a combination of peppermint and caraway oils), ginger, artichoke, licorice, and jollab (a combination of rose water, saffron, and candy sugar), may be as beneficial as conventional therapies when treating dyspepsia

symptoms.[36] However, it is important to note that herbal products are not regulated by the FDA and therefore it is difficult to assess the quality and safety of the ingredients found in alternative medications.[37]

Epidemiology

Indigestion is a common problem and frequent reason for primary care physicians to refer patients to GI specialists.[38] Worldwide, dyspepsia affects about a third of the population.[39] It can affect a person's quality of life even if the symptoms within themselves are usually not life-threatening. Additionally, the financial burden on the patient and healthcare system is costly - patients with dyspepsia were more likely to have lower work productivity and higher healthcare costs compared to those without indigestion.[40] Risk factors include NSAID-use, H. pylori infection, and smoking.[41]

See also

- Functional bowel disorder

References

1. Eusebi, Leonardo H; Black, Christopher J; Howden, Colin W; Ford, Alexander C (11 December 2019). "Effectiveness of management strategies for uninvestigated dyspepsia: systematic review and network meta-analysis" (https://www.ncbi.nlm.nih.gov/pmc/articles/PMC7190054). *BMJ*. **367**: l6483. doi:10.1136/bmj.l6483 (https://doi.org/10.1136%2Fbmj.l6483). PMC 7190054 (https://www.ncbi.nlm.nih.gov/pmc/articles/PMC7190054). PMID 31826881 (https://pubmed.ncbi.nlm.nih.gov/31826881).
2. "dyspepsia (https://web.archive.org/web/20090628224336/http://www.mercksource.com/pp/us/cns/cns_hl_dorlands_split.jsp?pg=/ppdocs/us/common/dorlands/dorland/three/000033170.htm)" at *Dorland's Medical Dictionary*
3. Duvnjak, Marko (2011). *Dyspepsia in clinical practice* (https://books.google.com/books?id=UGpATfS5-T8C&pg=PA2) (1. Aufl. ed.). New York: Springer. p. 2. ISBN 9781441917300.
4. Talley NJ, Vakil N (October 2005). "Guidelines for the management of dyspepsia". *Am. J. Gastroenterol*. **100** (10): 2324–37. doi:10.1111/j.1572-0241.2005.00225.x (https://doi.org/10.1111%2Fj.1572-0241.2005.00225.x). PMID 16181387 (https://pubmed.ncbi.nlm.nih.gov/16181387). S2CID 16499689 (https://api.semanticscholar.org/CorpusID:16499689).
5. Zajac, P; Holbrook, A; Super, ME; Vogt, M (March–April 2013). "An overview: Current clinical guidelines for the evaluation, diagnosis, treatment, and management of dyspepsia". *Osteopathic Family Physician*. **5** (2): 79–85. doi:10.1016/j.osfp.2012.10.005 (https://doi.org/10.1016%2Fj.osfp.2012.10.005).
6. Greenberger, Norton; Blumberg, R.S.; Burakoff, Robert (2016). *Current diagnosis & treatment. Gastroenterology, hepatology, and endoscopy* (https://www.worldcat.org/oclc/925478002) (3 ed.). New York. ISBN 978-1-259-25097-2. OCLC 925478002 (https://www.worldcat.org/oclc/925478002).
7. Saad RJ, Chey WD (August 2006). "Review article: current and emerging therapies for functional dyspepsia" (https://deepblue.lib.umich.edu/bitstream/2027.42/74835/1/j.1365-2036.2006.03005.x.pdf) (PDF). *Aliment. Pharmacol. Ther*. **24** (3): 475–92. doi:10.1111/j.1365-2036.2006.03005.x (https://doi.org/10.1111%2Fj.1365-2036.2006.03005.x). hdl:2027.42/74835 (https://hdl.handle.net/2027.42%2F74835). PMID 16886913 (https://pubmed.ncbi.nlm.nih.gov/16886913).

8. van Kerkhoven LA, van Rossum LG, van Oijen MG, Tan AC, Laheij RJ, Jansen JB (September 2006). "Upper gastrointestinal endoscopy does not reassure people with functional dyspepsia" (https://web.archive.org/web/20110727213028/http://www.researchcore.org/publications/docs/endo_vankerkhoven_reassurence.pdf) (PDF). *Endoscopy*. **38** (9): 879–85. doi:10.1055/s-2006-944661 (https://doi.org/10.1055%2Fs-2006-944661). PMID 16981103 (https://pubmed.ncbi.nlm.nih.gov/16981103). Archived from the original (http://www.researchcore.org/publications/docs/endo_vankerkhoven_reassurence.pdf) (PDF) on 2011-07-27.
9. Ford, Alexander C.; Mahadeva, Sanjiv; Carbone, M. Florencia; Lacy, Brian E.; Talley, Nicholas J. (2020-11-21). "Functional dyspepsia" (https://www.thelancet.com/journals/lancet/article/PIIS0140-6736(20)30469-4/abstract). *The Lancet*. **396** (10263): 1689–1702. doi:10.1016/S0140-6736(20)30469-4 (https://doi.org/10.1016%2FS0140-6736%2820%2930469-4). ISSN 0140-6736 (https://www.worldcat.org/issn/0140-6736). PMID 33049222 (https://pubmed.ncbi.nlm.nih.gov/33049222). S2CID 222254300 (https://api.semanticscholar.org/CorpusID:222254300).
10. "Rome IV Criteria" (https://theromefoundation.org/rome-iv/rome-iv-criteria/). *Rome Foundation*. Retrieved 2022-01-19.
11. Flier, SN; S, Rose (2006). "Is functional dyspepsia of particular concern in women? A review of gender differences in epidemiology, pathophysiologic mechanism, clinical presentation and management". *Am J Gastroenterol*. **101** (12 Suppl): S644–53. doi:10.1111/j.1572-0241.2006.01015.x (https://doi.org/10.1111%2Fj.1572-0241.2006.01015.x). PMID 17177870 (https://pubmed.ncbi.nlm.nih.gov/17177870). S2CID 27922893 (https://api.semanticscholar.org/CorpusID:27922893).
12. Vakil, Nimish (2006). "Limited Value of Alarm Features in the Diagnosis of Upper Gastrointestinal Malignancy: Systematic Review and Meta-analysis" (https://doi.org/10.1053/j.gastro.2006.04.029). *Gastroenterology*. **131** (2): 390–401. doi:10.1053/j.gastro.2006.04.029 (https://doi.org/10.1053%2Fj.gastro.2006.04.029). PMID 16890592 (https://pubmed.ncbi.nlm.nih.gov/16890592).
13. Barberio, Brigida; Mahadeva, Sanjiv; Black, Christopher J.; Savarino, Edoardo V.; Ford, Alexander C. (2020-07-28). "Systematic review with meta-analysis: global prevalence of uninvestigated dyspepsia according to the Rome criteria" (https://dx.doi.org/10.1111/apt.16006). *Alimentary Pharmacology & Therapeutics*. **52** (5): 762–773. doi:10.1111/apt.16006 (https://doi.org/10.1111%2Fapt.16006). ISSN 0269-2813 (https://www.worldcat.org/issn/0269-2813). PMID 32852839 (https://pubmed.ncbi.nlm.nih.gov/32852839). S2CID 221344221 (https://api.semanticscholar.org/CorpusID:221344221).
14. Sayuk, Gregory S.; Gyawali, C. Prakash (2020-09-01). "Functional Dyspepsia: Diagnostic and Therapeutic Approaches" (https://doi.org/10.1007/s40265-020-01362-4). *Drugs*. **80** (13): 1319–1336. doi:10.1007/s40265-020-01362-4 (https://doi.org/10.1007%2Fs40265-020-01362-4). ISSN 1179-1950 (https://www.worldcat.org/issn/1179-1950). PMID 32691294 (https://pubmed.ncbi.nlm.nih.gov/32691294). S2CID 220656815 (https://api.semanticscholar.org/CorpusID:220656815).
15. Holtmann, Gerald; Shah, Ayesha; Morrison, Mark (2017). "Pathophysiology of Functional Gastrointestinal Disorders: A Holistic Overview" (https://www.karger.com/Article/FullText/485409). *Digestive Diseases*. **35** (S1): 5–13. doi:10.1159/000485409 (https://doi.org/10.1159%2F000485409). ISSN 0257-2753 (https://www.worldcat.org/issn/0257-2753). PMID 29421808 (https://pubmed.ncbi.nlm.nih.gov/29421808). S2CID 3556796 (https://api.semanticscholar.org/CorpusID:3556796).
16. Eusebi, Leonardo H.; Ratnakumaran, Raguprakash; Yuan, Yuhong; Solaymani-Dodaran, Masoud; Bazzoli, Franco; Ford, Alexander C. (March 2018). "Global prevalence of, and risk factors for, gastro-oesophageal reflux symptoms: a meta-analysis" (https://pubmed.ncbi.nlm.nih.gov/28232473). *Gut*. **67** (3): 430–440. doi:10.1136/gutjnl-2016-313589 (https://doi.org/10.1136%2Fgutjnl-2016-313589). ISSN 1468-3288 (https://www.worldcat.org/issn/1468-3288). PMID 28232473 (https://pubmed.ncbi.nlm.nih.gov/28232473). S2CID 3496003 (https://api.semanticscholar.org/CorpusID:3496003).

17. Fashner, Julia; Gitu, Alfred C. (2015-02-15). "Diagnosis and Treatment of Peptic Ulcer Disease and H. pylori Infection" (https://pubmed.ncbi.nlm.nih.gov/25955624). *American Family Physician*. **91** (4): 236–242. ISSN 1532-0650 (https://www.worldcat.org/issn/1532-0650). PMID 25955624 (https://pubmed.ncbi.nlm.nih.gov/25955624).
18. Ford, Alexander C.; Tsipotis, Evangelos; Yuan, Yuhong; Leontiadis, Grigorios I.; Moayyedi, Paul (2022-01-12). "Efficacy of Helicobacter pylori eradication therapy for functional dyspepsia: updated systematic review and meta-analysis" (https://pubmed.ncbi.nlm.nih.gov/35022266). *Gut*. **71** (10): gutjnl–2021–326583. doi:10.1136/gutjnl-2021-326583 (https://doi.org/10.1136%2Fgutjnl-2021-326583). ISSN 1468-3288 (https://www.worldcat.org/issn/1468-3288). PMID 35022266 (https://pubmed.ncbi.nlm.nih.gov/35022266). S2CID 245922275 (https://api.semanticscholar.org/CorpusID:245922275).
19. Jung HK, Talley NJ (2018). "Role of the Duodenum in the Pathogenesis of Functional Dyspepsia: A Paradigm Shift" (https://www.ncbi.nlm.nih.gov/pmc/articles/PMC6034675). *J Neurogastroenterol Motil* (Review). **24** (3): 345–354. doi:10.5056/jnm18060 (https://doi.org/10.5056%2Fjnm18060). PMC 6034675 (https://www.ncbi.nlm.nih.gov/pmc/articles/PMC6034675). PMID 29791992 (https://pubmed.ncbi.nlm.nih.gov/29791992).
20. Talley NJ, Ford AC (Nov 5, 2015). "Functional Dyspepsia" (http://eprints.whiterose.ac.uk/97326/10/nejmra1501505.pdf) (PDF). *N Engl J Med* (Review). **373** (19): 1853–63. doi:10.1056/NEJMra1501505 (https://doi.org/10.1056%2FNEJMra1501505). PMID 26535514 (https://pubmed.ncbi.nlm.nih.gov/26535514).
21. Stanghellini, Vincenzo; Chan, Francis K. L.; Hasler, William L.; Malagelada, Juan R.; Suzuki, Hidekazu; Tack, Jan; Talley, Nicholas J. (May 2016). "Gastroduodenal Disorders" (https://pubmed.ncbi.nlm.nih.gov/27147122). *Gastroenterology*. **150** (6): 1380–1392. doi:10.1053/j.gastro.2016.02.011 (https://doi.org/10.1053%2Fj.gastro.2016.02.011). ISSN 1528-0012 (https://www.worldcat.org/issn/1528-0012). PMID 27147122 (https://pubmed.ncbi.nlm.nih.gov/27147122).
22. Mounsey, Anne; Barzin, Amir; Rietz, Ashley (2020-01-15). "Functional Dyspepsia: Evaluation and Management" (https://pubmed.ncbi.nlm.nih.gov/31939638). *American Family Physician*. **101** (2): 84–88. ISSN 1532-0650 (https://www.worldcat.org/issn/1532-0650). PMID 31939638 (https://pubmed.ncbi.nlm.nih.gov/31939638).
23. Ford AC, Moayyedi P (2013). "Dysepsia" (https://archive.is/20141221173257/http://pylori.org/indigestion-symptoms-signs-treatment/). *BMJ*. **347**: f5059. doi:10.1136/bmj.f5059 (https://doi.org/10.1136%2Fbmj.f5059). PMID 23990632 (https://pubmed.ncbi.nlm.nih.gov/23990632). S2CID 220190440 (https://api.semanticscholar.org/CorpusID:220190440). Archived from the original (http://pylori.org/indigestion-symptoms-signs-treatment/) on 2014-12-21. Retrieved 2014-12-21.
24. Duncanson, K. R.; Talley, N. J.; Walker, M. M.; Burrows, T. L. (June 2018). "Food and functional dyspepsia: a systematic review" (https://pubmed.ncbi.nlm.nih.gov/28913843). *Journal of Human Nutrition and Dietetics*. **31** (3): 390–407. doi:10.1111/jhn.12506 (https://doi.org/10.1111%2Fjhn.12506). ISSN 1365-277X (https://www.worldcat.org/issn/1365-277X). PMID 28913843 (https://pubmed.ncbi.nlm.nih.gov/28913843). S2CID 22800900 (https://api.semanticscholar.org/CorpusID:22800900).
25. Duncanson KR, Talley NJ, Walker MM, Burrows TL (2017). "Food and functional dyspepsia: a systematic review". *J Hum Nutr Diet* (Systematic Review). **31** (3): 390–407. doi:10.1111/jhn.12506 (https://doi.org/10.1111%2Fjhn.12506). PMID 28913843 (https://pubmed.ncbi.nlm.nih.gov/28913843). S2CID 22800900 (https://api.semanticscholar.org/CorpusID:22800900).
26. Futagami S, Itoh T, Sakamoto C (2015). "Systematic review with meta-analysis: post-infectious functional dyspepsia" (https://doi.org/10.1111%2Fapt.13006). *Aliment. Pharmacol. Ther.* **41** (2): 177–88. doi:10.1111/apt.13006 (https://doi.org/10.1111%2Fapt.13006). PMID 25348873 (https://pubmed.ncbi.nlm.nih.gov/25348873).

27. Tan, V. P. Y.; Liu, K. S. H.; Lam, F. Y. F.; Hung, I. F. N.; Yuen, M. F.; Leung, W. K. (2017-01-23). "Randomised clinical trial: rifaximin versus placebo for the treatment of functional dyspepsia" (https://dx.doi.org/10.1111/apt.13945). *Alimentary Pharmacology & Therapeutics*. **45** (6): 767–776. doi:10.1111/apt.13945 (https://doi.org/10.1111%2Fapt.13945). ISSN 0269-2813 (https://www.worldcat.org/issn/0269-2813). PMID 28112426 (https://pubmed.ncbi.nlm.nih.gov/28112426). S2CID 207052951 (https://api.semanticscholar.org/CorpusID:207052951).
28. Enck, Paul; Azpiroz, Fernando; Boeckxstaens, Guy; Elsenbruch, Sigrid; Feinle-Bisset, Christine; Holtmann, Gerald; Lackner, Jeffrey M.; Ronkainen, Jukka; Schemann, Michael; Stengel, Andreas; Tack, Jan (2017-11-03). "Functional dyspepsia" (https://pubmed.ncbi.nlm.nih.gov/29099093). *Nature Reviews. Disease Primers*. **3**: 17081. doi:10.1038/nrdp.2017.81 (https://doi.org/10.1038%2Fnrdp.2017.81). ISSN 2056-676X (https://www.worldcat.org/issn/2056-676X). PMID 29099093 (https://pubmed.ncbi.nlm.nih.gov/29099093). S2CID 4929427 (https://api.semanticscholar.org/CorpusID:4929427).
29. Milivojevic, Vladimir; Rankovic, Ivan; Krstic, Miodrag N.; Milosavljevic, Tomica (2021-06-14). "Dyspepsia-challenge in primary care gastroenterology" (https://doi.org/10.1159%2F000517668). *Digestive Diseases*. S. Karger AG. **40** (3): 270–275. doi:10.1159/000517668 (https://doi.org/10.1159%2F000517668). ISSN 0257-2753 (https://www.worldcat.org/issn/0257-2753). PMID 34126614 (https://pubmed.ncbi.nlm.nih.gov/34126614).
30. Ford, Alexander C.; Moayyedi, Paul; Black, Christopher J.; Yuan, Yuhong; Veettil, Sajesh K.; Mahadeva, Sanjiv; Kengkla, Kirati; Chaiyakunapruk, Nathorn; Lee, Yeong Yeh (2020-09-16). "Systematic review and network meta-analysis: efficacy of drugs for functional dyspepsia" (https://doi.org/10.1111/apt.16072). *Alimentary Pharmacology & Therapeutics*. **53** (1): 8–21. doi:10.1111/apt.16072 (https://doi.org/10.1111%2Fapt.16072). ISSN 0269-2813 (https://www.worldcat.org/issn/0269-2813). PMID 32936964 (https://pubmed.ncbi.nlm.nih.gov/32936964). S2CID 221768794 (https://api.semanticscholar.org/CorpusID:221768794).
31. Duboc, Henri; Latrache, Sofya; Nebunu, Nicoleta; Coffin, Benoit (2020). "The Role of Diet in Functional Dyspepsia Management" (https://www.ncbi.nlm.nih.gov/pmc/articles/PMC7012988). *Frontiers in Psychiatry*. **11**: 23. doi:10.3389/fpsyt.2020.00023 (https://doi.org/10.3389%2Ffpsyt.2020.00023). ISSN 1664-0640 (https://www.worldcat.org/issn/1664-0640). PMC 7012988 (https://www.ncbi.nlm.nih.gov/pmc/articles/PMC7012988). PMID 32116840 (https://pubmed.ncbi.nlm.nih.gov/32116840).
32. Pinto-Sanchez, Maria Ines; Yuan, Yuhong; Hassan, Ahmed; Bercik, Premysl; Moayyedi, Paul (2017-11-21). "Proton pump inhibitors for functional dyspepsia" (https://www.ncbi.nlm.nih.gov/pmc/articles/PMC6485982). *The Cochrane Database of Systematic Reviews*. **11** (3): CD011194. doi:10.1002/14651858.CD011194.pub3 (https://doi.org/10.1002%2F14651858.CD011194.pub3). ISSN 1469-493X (https://www.worldcat.org/issn/1469-493X). PMC 6485982 (https://www.ncbi.nlm.nih.gov/pmc/articles/PMC6485982). PMID 29161458 (https://pubmed.ncbi.nlm.nih.gov/29161458).
33. Huang, Xinyi; Oshima, Tadayuki; Tomita, Toshihiko; Fukui, Hirokazu; Miwa, Hiroto (November 2021). "Meta-Analysis: Placebo Response and Its Determinants in Functional Dyspepsia" (https://journals.lww.com/10.14309/ajg.0000000000001397). *American Journal of Gastroenterology*. **116** (11): 2184–2196. doi:10.14309/ajg.0000000000001397 (https://doi.org/10.14309%2Fajg.0000000000001397). ISSN 0002-9270 (https://www.worldcat.org/issn/0002-9270). PMID 34404084 (https://pubmed.ncbi.nlm.nih.gov/34404084). S2CID 237199057 (https://api.semanticscholar.org/CorpusID:237199057).
34. Pittayanon, Rapat; Yuan, Yuhong; Bollegala, Natasha P; Khanna, Reena; Leontiadis, Grigorios I; Moayyedi, Paul (2018-10-18). Cochrane Upper GI and Pancreatic Diseases Group (ed.). "Prokinetics for functional dyspepsia" (https://www.ncbi.nlm.nih.gov/pmc/articles/PMC6516965). *Cochrane Database of Systematic Reviews*. **2018** (10): CD009431. doi:10.1002/14651858.CD009431.pub3 (https://doi.org/10.1002%2F14651858.CD009431.pub3). PMC 6516965 (https://www.ncbi.nlm.nih.gov/pmc/articles/PMC6516965). PMID 30335201 (https://pubmed.ncbi.nlm.nih.gov/30335201).

35. "Proton Pump Inhibitors: Use in Adults" (https://www.cms.gov/Medicare-Medicaid-Coordination/Fraud-Prevention/Medicaid-Integrity-Education/Pharmacy-Education-Materials/Downloads/ppi-adult-factsheet11-14.pdf) (PDF). *Centers for Medicare & Medicaid Services*. 2015. Retrieved 2022-01-27.
36. Heiran, Alireza; Bagheri Lankarani, Kamran; Bradley, Ryan; Simab, Alireza; Pasalar, Mehdi (2021-12-01). "Efficacy of herbal treatments for functional dyspepsia: A systematic review and meta-analysis of randomized clinical trials" (https://pubmed.ncbi.nlm.nih.gov/34851546). *Phytotherapy Research*. **36** (2): 686–704. doi:10.1002/ptr.7333 (https://doi.org/10.1002%2Fptr.7333). ISSN 1099-1573 (https://www.worldcat.org/issn/1099-1573). PMID 34851546 (https://pubmed.ncbi.nlm.nih.gov/34851546). S2CID 244774488 (https://api.semanticscholar.org/CorpusID:244774488).
37. Kanfer, Isadore; Patnala, Srinivas (2021-01-01), Henkel, Ralf; Agarwal, Ashok (eds.), "Chapter 7 - Regulations for the use of herbal remedies" (https://www.sciencedirect.com/science/article/pii/B9780128155653000072), *Herbal Medicine in Andrology*, Academic Press, pp. 189–206, doi:10.1016/b978-0-12-815565-3.00007-2 (https://doi.org/10.1016%2Fb978-0-12-815565-3.00007-2), ISBN 978-0-12-815565-3, S2CID 234186151 (https://api.semanticscholar.org/CorpusID:234186151), retrieved 2022-01-27
38. Rodrigues, David M; Motomura, Douglas I; Tripp, Dean A; Beyak, Michael J (2021-06-16). "Are psychological interventions effective in treating functional dyspepsia? A systematic review and meta-analysis" (https://dx.doi.org/10.1111/jgh.15566). *Journal of Gastroenterology and Hepatology*. **36** (8): 2047–2057. doi:10.1111/jgh.15566 (https://doi.org/10.1111%2Fjgh.15566). ISSN 0815-9319 (https://www.worldcat.org/issn/0815-9319). PMID 34105186 (https://pubmed.ncbi.nlm.nih.gov/34105186). S2CID 235379735 (https://api.semanticscholar.org/CorpusID:235379735).
39. Esterita, Tasia; Dewi, Sheilla; Suryatenggara, Felicia Grizelda; Glenardi, Glenardi (2021-06-18). "Association of Functional Dyspepsia with Depression and Anxiety: A Systematic Review" (https://pubmed.ncbi.nlm.nih.gov/33951117). *Journal of Gastrointestinal and Liver Diseases: JGLD*. **30** (2): 259–266. doi:10.15403/jgld-3325 (https://doi.org/10.15403%2Fjgld-3325). ISSN 1842-1121 (https://www.worldcat.org/issn/1842-1121). PMID 33951117 (https://pubmed.ncbi.nlm.nih.gov/33951117). S2CID 233868221 (https://api.semanticscholar.org/CorpusID:233868221).
40. Esterita, Tasia; Dewi, Sheilla; Suryatenggara, Felicia Grizelda; Glenardi, Glenardi (2021-06-18). "Association of Functional Dyspepsia with Depression and Anxiety: A Systematic Review" (https://www.jgld.ro/jgld/index.php/jgld/article/view/3325). *Journal of Gastrointestinal and Liver Diseases*. **30** (2): 259–266. doi:10.15403/jgld-3325 (https://doi.org/10.15403%2Fjgld-3325). ISSN 1842-1121 (https://www.worldcat.org/issn/1842-1121). PMID 33951117 (https://pubmed.ncbi.nlm.nih.gov/33951117). S2CID 233868221 (https://api.semanticscholar.org/CorpusID:233868221).
41. Tsukanov, V. V.; Vasyutin, A. V.; Tonkikh, Ju. L. (2020-10-22). "Modern aspects of the pathogenesis and treatment of dyspepsia" (https://dx.doi.org/10.21518/2079-701x-2020-15-40-46). *Meditsinskiy Sovet = Medical Council* (15): 40–46. doi:10.21518/2079-701x-2020-15-40-46 (https://doi.org/10.21518%2F2079-701x-2020-15-40-46). ISSN 2658-5790 (https://www.worldcat.org/issn/2658-5790). S2CID 226340276 (https://api.semanticscholar.org/CorpusID:226340276).

External links

Retrieved from "https://en.wikipedia.org/w/index.php?title=Indigestion&oldid=1121453386#Functional"

This page was last edited on 12 November 2022, at 10:43 (UTC).

Text is available under the Creative Commons Attribution-ShareAlike License 3.0; additional terms may apply. By using this site, you agree to the Terms of Use and Privacy Policy. Wikipedia® is a registered trademark of the Wikimedia Foundation, Inc., a non-profit organization.
