# Supplementary material for: Assessment of medical information on irritable bowel syndrome information in Wikipedia and Baidu Encyclopedia: comparative study
Source: PeerJ. 2024 May 24;12:e17264. doi: 10.7717/peerj.17264 (PMC11129691; doi:10.7717/peerj.17264)
Supplement: Data S1 [file peerj-12-17264-s001.zip › σÄƒσoïμò░μì«/Wikipedia/Small intestinal bacterial overgrowth - Wikipedia.pdf]

Wikipedia

Small intestinal bacterial overgrowth

Small intestinal bacterial overgrowth (**SIBO**), also termed **bacterial overgrowth**, or **small bowel bacterial overgrowth syndrome** (**SBBOS**), is a disorder of excessive bacterial growth in the small intestine. Unlike the colon (or large bowel), which is rich with bacteria, the small bowel usually has fewer than 100,000 organisms per millilitre.[1] Patients with bacterial overgrowth typically develop symptoms which may include nausea, bloating, vomiting, diarrhea, malnutrition, weight loss and malabsorption,[2] which is caused by a number of mechanisms.

Small intestinal bacterial overgrowth	
**Other names**	bacterial overgrowth, small bowel bacterial overgrowth syndrome (SBBOS)

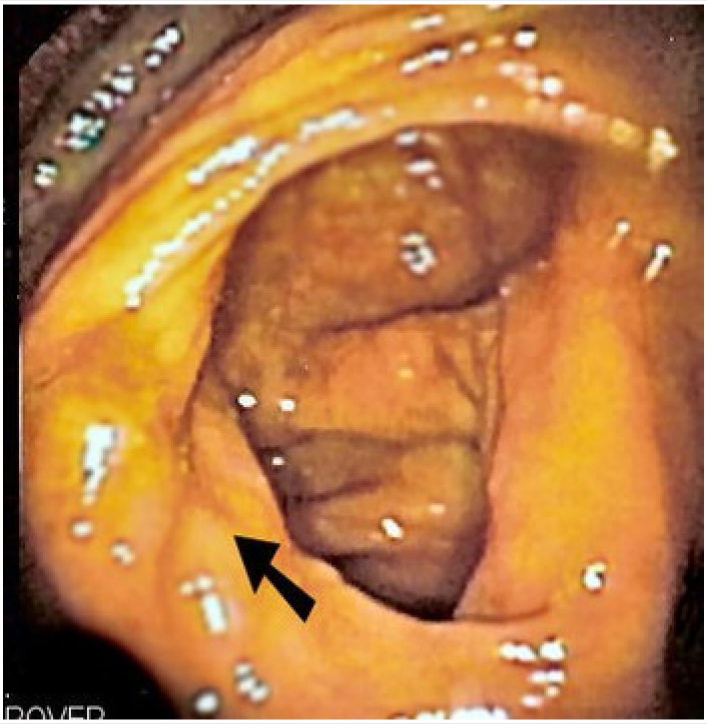

The ileocecal valve prevents reflux of bacteria from the colon into the small bowel. Resection of the valve can lead to bacterial overgrowth.

**Specialty**	Gastroenterology

The diagnosis of bacterial overgrowth is made by a number of techniques, with the gold standard[3] being an aspirate from the jejunum that grows in excess of 10^5 bacteria per millilitre. Risk factors for the development of bacterial overgrowth include dysmotility; anatomical disturbances in the bowel, including fistulae, diverticula and blind loops created after surgery, and resection of the ileo-cecal valve; gastroenteritis-induced alterations to the small intestine; and the use of certain medications, including proton pump inhibitors.

Small bowel bacterial overgrowth syndrome is treated with an elemental diet or antibiotics, which may be given in a cyclic fashion to prevent tolerance to the antibiotics, sometimes followed by prokinetic drugs to prevent recurrence if dysmotility is a suspected cause.

Contents

Definition
- Methane-dominant SIBO

Signs and symptoms
- Related conditions
  - Irritable bowel syndrome

Etiology and risk factors

Diagnosis

Treatment

References

External links

Definition

SIBO may be defined as an increased number of bacteria measured via exhaled hydrogen and/or methane gas following the ingestion of glucose, or via analysis of small bowel aspirate fluid.[3] Nevertheless, as of 2020, the definition of SIBO as a clinical entity lacks precision and consistency; it is a term generally applied to a clinical disorder where symptoms, clinical signs, and/or laboratory abnormalities are attributed to changes in the numbers of bacteria or in the composition of the bacterial population in the small intestine.[4] The main obstacle to accurately define SIBO is limited understanding of the normal intestinal microbial population. Future advances in sampling technology and techniques for counting bacterial populations and their metabolites should provide much-needed clarity.[4]

Methane-dominant SIBO

The archaeon *Methanobrevibacter smithii*, has been associated with symptoms of SIBO which result in a positive methane breath test.[5] In addition to the archaeon, a few bacteria can also produce methane, such as members of the *Clostridium* and *Bacteroides* genus. Production of methane therefore, may not be bacterial, nor limited to the small intestine, and it has been proposed that the condition should be classified as a separate 'intestinal methanogen overgrowth' (IMO).[5]

Signs and symptoms

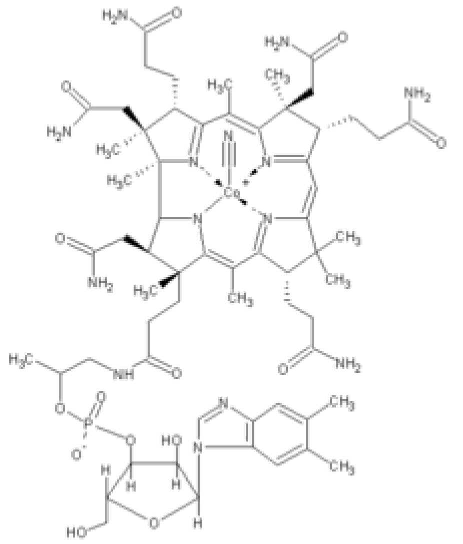

Deficiency of vitamin B_{12} can occur in bacterial overgrowth

Symptoms traditionally linked to SIBO include bloating, diarrhea, and abdominal pain/discomfort. Steatorrhea may be seen in more severe cases.[4]

Bacterial overgrowth can cause a variety of symptoms, many of which are also found in other conditions, making the diagnosis challenging at times.[4] Many of the symptoms are due to malabsorption of nutrients due to the effects of bacteria which either metabolize nutrients or cause inflammation of the small bowel, impairing absorption. The symptoms of bacterial overgrowth include nausea, flatus,[6] constipation,[7] bloating, abdominal distension, abdominal pain or discomfort, diarrhea,[8] fatigue, and weakness. SIBO also causes an increased permeability of the small intestine.[9] Some patients may lose weight. Children with bacterial overgrowth may develop malnutrition and have difficulty attaining proper growth. Steatorrhea, a sticky type of diarrhea where fats are not properly absorbed and spill into the stool, may also occur.[6]

Patients with bacterial overgrowth that is longstanding can develop complications of their illness as a result of malabsorption of nutrients.[10] Laboratory test results may include elevated folate, and, less commonly, vitamin B_{12} deficiency or other nutritional deficiencies.[4] Anemia may occur from a variety of mechanisms, as many of the nutrients involved in production of red blood cells are absorbed in the affected small bowel. Iron is absorbed in the more proximal parts of the small

bowel, the duodenum and jejunum, and patients with malabsorption of iron can develop a microcytic anemia, with small red blood cells. Vitamin B_{12} is absorbed in the last part of the small bowel, the ileum, and patients who malabsorb vitamin B_{12} can develop a megaloblastic anemia with large red blood cells.[6]

Related conditions

In recent years, several proposed links between SIBO and other disorders have been made. However, the usual methodology of these studies involves the use of breath testing as an indirect investigation for SIBO. Breath testing has been criticized by some authors for being an imperfect test for SIBO, with multiple known false positives.[11]

Irritable bowel syndrome

Some studies reported up to 80% of patients with irritable bowel syndrome (IBS) have SIBO (using the hydrogen breath test). IBS-D is associated with elevated hydrogen numbers on breath tests while IBS-C is associated with elevated methane numbers on breath tests. Subsequent studies demonstrated statistically significant reduction in IBS symptoms following therapy for SIBO.[12][13][14]

Various mechanisms are involved in the development of diarrhea and IBS-D in bacterial overgrowth. First, the excessive bacterial concentrations can cause direct inflammation of the small bowel cells, leading to an *inflammatory* diarrhea. The malabsorption of lipids, proteins and carbohydrates may cause poorly digestible products to enter into the colon. This can cause diarrhea by the osmotic drive of these molecules, but can also stimulate the secretory mechanisms of colonic cells, leading to a *secretory diarrhea*.[6]

There is a lack of consensus however, regarding the suggested link between IBS and SIBO. Other authors concluded that the abnormal breath results so common in IBS patients do not suggest SIBO, and state that "abnormal fermentation timing and dynamics of the breath test findings support a role for abnormal intestinal bacterial distribution in IBS."[15] There is general consensus that breath tests are abnormal in IBS; however, the disagreement lies in whether this is representative of SIBO.[16]

Etiology and risk factors

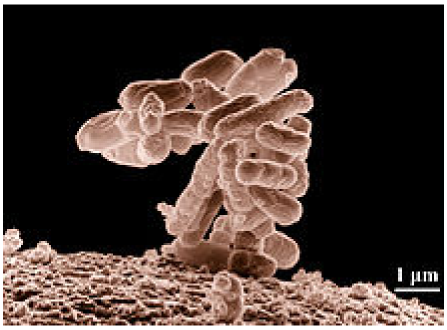

E. coli, shown in this electron micrograph, is commonly isolated in patients with bacterial overgrowth

Certain people are more predisposed to the development of bacterial overgrowth because of certain risk factors. These factors can be grouped into four categories: (1) disordered motility or movement of the small bowel or anatomical changes that lead to stasis, (2) disorders in the immune system, (3) interference with the production of proteolytic enzymes, gastric acid, or bile, and (4) conditions that cause more bacteria from the colon to enter the small bowel.[4] In some people, methanogens may reside in the oral cavity, as evidenced by reductions in breath methane levels following mouthwash with chlorhexidine.[17] This may affect results from hydrogen-methane breath testing.

Absence or impairment of the migrating motor complex (MMC), a cyclical motility pattern in the small intestine, and phase III of the MMC in particular, is associated with the development of SIBO.[18] Problems with motility may either be diffuse, or

localized to particular areas.

MMC impairment may be a result of post-infectious irritable bowel syndrome, drug use, or intestinal pseudo-obstruction among other causes.[19] There is an overlap in findings between tropical sprue, post-infectious irritable bowel syndrome and small intestinal bacterial overgrowth in the pathophysiology of the three conditions and also SIBO can similarly sometimes be triggered by an acute gastrointestinal infection.[20][21][22] As of 2020, there is still controversy about the role of SIBO in the pathogenesis of common functional symptoms such as those considered to be components of irritable bowel syndrome.[4] Diseases like scleroderma[23] cause diffuse slowing of the bowel, leading to increased bacterial concentrations. More commonly, the small bowel may have anatomical problems, such as out-pouchings known as diverticula that can cause bacteria to accumulate.[24] After surgery involving the stomach and duodenum (most commonly with Billroth II antrectomy), a *blind loop* may be formed, leading to stasis of flow of intestinal contents. This can cause overgrowth, and is termed *blind loop syndrome*.

Systemic or metabolic disorders may lead to conditions allowing bacterial overgrowth as well. For example, diabetes can cause intestinal neuropathy, pancreatitis leading to pancreatic insufficiency can impair digestive enzyme production, and bile may be affected as part of cirrhosis of the liver.[25] Proton pump inhibitors, a class of medication that are used to reduce stomach acid, is associated with an increased risk of developing SIBO.[26]

Finally, abnormal connections between the bacteria-rich colon and the small bowel can increase the bacterial load in the small bowel. Patients with Crohn's disease or other diseases of the ileum may require surgery that removes the ileocecal valve connecting the small and large bowel; this leads to an increased reflux of bacteria into the small bowel. After bariatric surgery for obesity, connections between the stomach and the ileum can be formed, which may increase bacterial load in the small bowel.[27]

Diagnosis

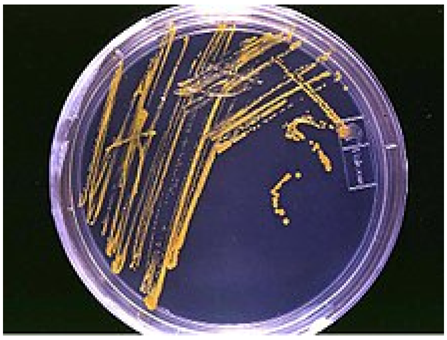

Aspiration of bacteria from the jejunum is the gold standard for diagnosis. A bacterial load of greater than 10^5 bacteria per millilitre is diagnostic for bacterial overgrowth

The diagnosis of bacterial overgrowth can be made by physicians in various ways. Malabsorption can be detected by a test called the D-xylose absorption test. Xylose is a sugar that does not require enzymes to be digested. The D-xylose test involves having a patient drink a certain quantity of D-xylose, and measuring levels in the urine and blood; if there is no evidence of D-xylose in the urine and blood, it suggests that the small bowel is not absorbing properly (as opposed to problems with enzymes required for digestion).[28]

The gold standard for detection of bacterial overgrowth is the aspiration of more than 10^5 bacteria per millilitre from the small bowel. The normal small bowel has less than 10^4 bacteria per millilitre. Some experts however, consider aspiration of more than 10^3 positive if the flora is predominately colonic type bacteria as these types of bacteria are considered pathological in excessive numbers in the small intestine. The reliability of aspiration in the diagnosis of SIBO has been questioned as SIBO can be patchy and the reproducibility can be as low as 38 percent. Breath tests have their own reliability problems with a high rate of false positive. Some doctors factor in a patients' response to treatment as part of the diagnosis.[4]

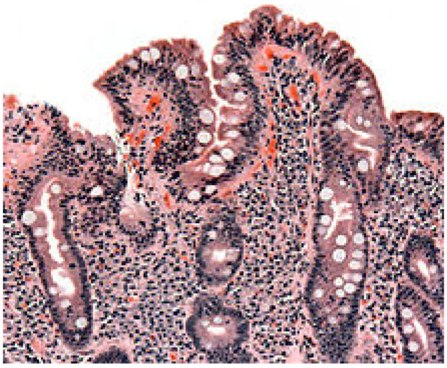

Biopsies of the small bowel in bacterial overgrowth can mimic celiac disease, with partial villous atrophy.

Breath tests have been developed to test for bacterial overgrowth. These tests are either based on bacterial metabolism of carbohydrates to hydrogen, methane, or hydrogen sulfide, or based on the detection of by-products of digestion of carbohydrates that are not usually metabolized. The hydrogen breath test involves having the patient fast for a minimum of 12 hours then having them drink a substrate usually glucose or lactulose, then measuring expired hydrogen and methane concentrations typically over a period of several hours. It compares well to jejunal aspirates in making the diagnosis of bacterial overgrowth. Carbon-13 (^{13}C) and carbon-14 (^{14}C) based tests have also been developed based on the bacterial metabolism of D-xylose. Increased bacterial concentrations are also involved in the deconjugation of bile acids. The glycocholic acid breath test involves the administration of the bile acid ^{14}C glycocholic acid, and the detection of $^{14}CO_2$, which would be elevated in bacterial overgrowth.

However, some physicians suggest that if the suspicion of bacterial overgrowth is high enough, the best diagnostic test is a trial of treatment. If the symptoms improve, an empiric diagnosis of bacterial overgrowth can be made.[29]

There is insufficient evidence to support the use of inflammatory markers, such as fecal calprotectin, to detect SIBO.[4]

Treatment

Treatment strategies should focus on identifying and correcting the root causes, where possible, resolving nutritional deficiencies, and administering antibiotics. This is especially important for patients with indigestion and malabsorption.[4] Although whether antibiotics should be a first line treatment is a matter of debate. Some experts recommend probiotics as first line therapy with antibiotics being reserved as a second line treatment for more severe cases of SIBO. Prokinetic drugs are other options but research in humans is limited.[30][31] A variety of antibiotics, including tetracycline, amoxicillin-clavulanate, metronidazole, neomycin, cephalexin and trimethoprim-sulfamethoxazole have been used; however, the best evidence is for the use of rifaximin, a poorly-absorbed antibiotic.[32] Although irritable bowel syndrome has been shown to respond to the treatment of poorly-absorbed antibiotics, there is limited evidence on the effectiveness of such treatment in cases of SIBO, and as of 2020, randomized controlled trials are still needed to further confirm the eradicating effect of such treatment in SIBO.[4] A course of one week of antibiotics is usually sufficient to treat the condition. However, if the condition recurs, antibiotics can be given in a cyclical fashion in order to prevent tolerance. For example, antibiotics may be given for a week, followed by three weeks off antibiotics, followed by another week of treatment. Alternatively, the choice of antibiotic used can be cycled.[29] There is still limited data to guide the clinician in developing antibiotic strategies for SIBO. Therapy remains, for the most part, empiric. However, concerns exist about the potential risks of long-term broad-spectrum antibiotic therapy.[4]

Probiotics are bacterial preparations that alter the bacterial flora in the bowel to cause a beneficial effect. Animal research has demonstrated that probiotics have barrier enhancing, antibacterial, immune modulating and anti-inflammatory effects which may have a positive effect in the management of SIBO in humans.[4] *Lactobacillus casei* has been found to be effective in improving breath hydrogen scores after 6 weeks of treatment presumably by suppressing levels of a small intestinal bacterial overgrowth of fermenting bacteria. The multi-strain preparation VSL#3 was found to be effective in suppressing SIBO.[33] *Lactobacillus plantarum*, *Lactobacillus acidophilus*,

and *Lactobacillus casei* have all demonstrated effectiveness in the treatment and management of SIBO. Conversely, *Lactobacillus fermentum* and *Saccharomyces boulardii* have been found to be ineffective.[4] A combination of *Lactobacillus plantarum* and *Lactobacillus rhamnosus* has been found to be effective in suppressing bacterial overgrowth of abnormal gas producing organisms in the small intestine.[34]

Probiotics are superior to antibiotics in the treatment of SIBO.[9] A combination of probiotic strains has been found to produce better results than therapy with the antibiotic drug metronidazole.[35]

An elemental diet has been shown to be highly effective for eliminating SIBO with a two-week diet demonstrating 80% efficacy and a three-week diet demonstrating 85% efficacy.[36][37] An elemental diet works via providing nutrition for the individual while depriving the bacteria of a food source.[38] Additional treatment options include the use of prokinetic drugs such as 5-HT4 receptor agonists or motilin agonists to extend the SIBO free period after treatment with an elemental diet or antibiotics.[39] A diet void of certain foods that feed the bacteria can help alleviate the symptoms.[40] For example, if the symptoms are caused by bacterial overgrowth feeding on indigestible carbohydrate rich foods, following a FODMAP restriction diet may help.[40]

References

1. Finegold SM (June 1969). "Intestinal Bacteria — The Role They Play in Normal Physiology, Pathologic Physiology, and Infection" (https://www.ncbi.nlm.nih.gov/pmc/articles/PMC1503548). *Western Journal of Medicine*. **110** (6): 455 —4 59. PMC 1503548 (https://www.ncbi.nlm.nih.gov/pmc/articles/PMC1503548). PMID 5789139 (https://pubmed.ncbi.nlm.nih.gov/5789139).
2. Bures J, Cyrany J, Kohoutova D, Förstl M, Rejchrt S, Kvetina J, et al. (June 2010). "Small intestinal bacterial overgrowth syndrome" (https://www.ncbi.nlm.nih.gov/pmc/articles/PMC2890937). *World Journal of Gastroenterology*. **16** (24): 2978–90. doi:10.3748/wjg.v16.i24.2978 (https://doi.org/10.3748%2Fwjg.v16.i24.2978). PMC 2890937 (https://www.ncbi.nlm.nih.gov/pmc/articles/PMC2890937). PMID 20572300 (https://pubmed.ncbi.nlm.nih.gov/20572300).
3. Ghoshal UC, Ghoshal U, Das K, Misra A (January–February 2006). "Utility of hydrogen breath tests in diagnosis of small intestinal bacterial overgrowth in malabsorption syndrome and its relationship with oro-cecal transit time" (https://pubmed.ncbi.nlm.nih.gov/16567886/). *Indian Journal of Gastroenterology*. **25** (1): 6–10. PMID 16567886 (https://pubmed.ncbi.nlm.nih.gov/16567886).
4. Quigley E, Murray J, Pimentel M (October 2020). "AGA Clinical Practice Update on Small Intestinal Bacterial Overgrowth: Expert Review" (https://doi.org/10.1053%2Fj.gastro.2020.06.090). *Gastroenterology*. **159** (4): 1526–1532. doi:10.1053/j.gastro.2020.06.090 (https://doi.org/10.1053%2Fj.gastro.2020.06.090). PMID 32679220 (https://pubmed.ncbi.nlm.nih.gov/32679220).
5. Takakura, Will; Pimentel, Mark (2020-07-10). "Small Intestinal Bacterial Overgrowth and Irritable Bowel Syndrome – An Update" (https://www.ncbi.nlm.nih.gov/pmc/articles/PMC7366247). *Frontiers in Psychiatry*. **11**: 664. doi:10.3389/fpsyt.2020.00664 (https://doi.org/10.3389%2Ffpsyt.2020.00664). ISSN 1664-0640 (https://www.worldcat.org/issn/1664-0640). PMC 7366247 (https://www.ncbi.nlm.nih.gov/pmc/articles/PMC7366247). PMID 32754068 (https://pubmed.ncbi.nlm.nih.gov/32754068).
6. Kirsch M (March 1990). "Bacterial overgrowth". *The American Journal of Gastroenterology*. **85** (3): 231–7. PMID 2178395 (https://pubmed.ncbi.nlm.nih.gov/2178395).

7. Lin HC (August 2004). "Small intestinal bacterial overgrowth: a framework for understanding irritable bowel syndrome" (https://doi.org/10.1001%2Fjama.292.7.852). *JAMA*. **292** (7): 852–8. doi:10.1001/jama.292.7.852 (https://doi.org/10.1001%2Fjama.292.7.852). PMID 15316000 (https://pubmed.ncbi.nlm.nih.gov/15316000).
8. Sachdev AH, Pimentel M (September 2013). "Gastrointestinal bacterial overgrowth: pathogenesis and clinical significance" (https://www.ncbi.nlm.nih.gov/pmc/articles/PMC3752184). *Therapeutic Advances in Chronic Disease*. **4** (5): 223–31. doi:10.1177/2040622313496126 (https://doi.org/10.1177%2F2040622313496126). PMC 3752184 (https://www.ncbi.nlm.nih.gov/pmc/articles/PMC3752184). PMID 23997926 (https://pubmed.ncbi.nlm.nih.gov/23997926).
9. Lykova EA, Bondarenko VM, Parfenov AI, Matsulevich TV (2005). "[Bacterial overgrowth syndrome in the small intestine: pathogenesis, clinical significance and therapy tactics]". *Eksperimental'naia i Klinicheskaia Gastroenterologiia = Experimental & Clinical Gastroenterology* (in Russian) (6): 51–7, 113. PMID 17378388 (https://pubmed.ncbi.nlm.nih.gov/17378388).
10. Ensari A (21 Aug 2014). "The Malabsorption Syndrome and Its Causes and Consequences" (https://www.ncbi.nlm.nih.gov/pmc/articles/PMC7149679). *Pathobiology of Human Disease*: 1266–1287. doi:10.1016/B978-0-12-386456-7.03804-1 (https://doi.org/10.1016%2FB978-0-12-386456-7.03804-1). ISBN 9780123864574. PMC 7149679 (https://www.ncbi.nlm.nih.gov/pmc/articles/PMC7149679).
11. Simrén M, Stotzer PO (March 2006). "Use and abuse of hydrogen breath tests" (https://www.ncbi.nlm.nih.gov/pmc/articles/PMC1856094). *Gut*. **55** (3): 297–303. doi:10.1136/gut.2005.075127 (https://doi.org/10.1136%2Fgut.2005.075127). PMC 1856094 (https://www.ncbi.nlm.nih.gov/pmc/articles/PMC1856094). PMID 16474100 (https://pubmed.ncbi.nlm.nih.gov/16474100).
12. Pimentel M (2006). *A new IBS solution : bacteria, the missing link in treating irritable bowel syndrome*. Sherman Oaks, CA: Health Point Press. ISBN 978-0977435609.
13. Reddymasu SC, Sostarich S, McCallum RW (February 2010). "Small intestinal bacterial overgrowth in irritable bowel syndrome: are there any predictors?" (https://www.ncbi.nlm.nih.gov/pmc/articles/PMC2838757). *BMC Gastroenterology*. **10**: 23. doi:10.1186/1471-230X-10-23 (https://doi.org/10.1186%2F1471-230X-10-23). PMC 2838757 (https://www.ncbi.nlm.nih.gov/pmc/articles/PMC2838757). PMID 20175924 (https://pubmed.ncbi.nlm.nih.gov/20175924).
14. Lin HC (August 2004). "Small intestinal bacterial overgrowth: a framework for understanding irritable bowel syndrome" (https://doi.org/10.1001%2Fjama.292.7.852). *JAMA*. **292** (7): 852–8. doi:10.1001/jama.292.7.852 (https://doi.org/10.1001%2Fjama.292.7.852). PMID 15316000 (https://pubmed.ncbi.nlm.nih.gov/15316000).
15. Shah ED, Basseri RJ, Chong K, Pimentel M (September 2010). "Abnormal breath testing in IBS: a meta-analysis". *Digestive Diseases and Sciences*. **55** (9): 2441–9. doi:10.1007/s10620-010-1276-4 (https://doi.org/10.1007%2Fs10620-010-1276-4). PMID 20467896 (https://pubmed.ncbi.nlm.nih.gov/20467896). S2CID 38690372 (https://api.semanticscholar.org/CorpusID:38690372).
16. Ford AC, Spiegel BM, Talley NJ, Moayyedi P (December 2009). "Small intestinal bacterial overgrowth in irritable bowel syndrome: systematic review and meta-analysis". *Clinical Gastroenterology and Hepatology*. **7** (12): 1279–86. doi:10.1016/j.cgh.2009.06.031 (https://doi.org/10.1016%2Fj.cgh.2009.06.031). PMID 19602448 (https://pubmed.ncbi.nlm.nih.gov/19602448).
17. Erdrich S, Tan Edwin CK, Hawrelak JA, Myers SP, Harnett JE (2021). "Hydrogen–methane breath testing results influenced by oral hygiene" (https://doi.org/10.1038/s41598-020-79554-x). *Scientific Reports*. **11** (26): 26. doi:10.1038/s41598-020-79554-x (https://doi.org/10.1038%2Fs41598-020-79554-x). PMC 7794545 (https://www.ncbi.nlm.nih.gov/pmc/articles/PMC7794545). PMID 33420116 (https://pubmed.ncbi.nlm.nih.gov/33420116).

18. Deloose E, Janssen P, Depoortere I, Tack J (March 2012). "The migrating motor complex: control mechanisms and its role in health and disease". *Nature Reviews. Gastroenterology & Hepatology*. **9** (5): 271–85. doi:10.1038/nrgastro.2012.57 (https://doi.org/10.1038%2Fnrgastro.2012.57). PMID 22450306 (https://pubmed.ncbi.nlm.nih.gov/22450306). S2CID 22753804 (https://api.semanticscholar.org/CorpusID:22753804).
19. Pimentel M (31 July 2018). Lamont JT, Grover S (eds.). "Small intestinal bacterial overgrowth: Etiology and pathogenesis" (https://www.uptodate.com/contents/small-intestinal-bacterial-overgrowth-etiology-and-pathogenesis). *UpToDate*. Retrieved 2019-12-11.
20. Ghoshal UC, Park H, Gwee KA (February 2010). "Bugs and irritable bowel syndrome: The good, the bad and the ugly" (https://doi.org/10.1111%2Fj.1440-1746.2009.06133.x). *Journal of Gastroenterology and Hepatology*. **25** (2): 244–51. doi:10.1111/j.1440-1746.2009.06133.x (https://doi.org/10.1111%2Fj.1440-1746.2009.06133.x). PMID 20074148 (https://pubmed.ncbi.nlm.nih.gov/20074148). S2CID 22023086 (https://api.semanticscholar.org/CorpusID:22023086).
21. Othman M, Agüero R, Lin HC (January 2008). "Alterations in intestinal microbial flora and human disease" (http://www.medscape.com/viewarticle/568375_1). *Current Opinion in Gastroenterology*. **24** (1): 11–6. doi:10.1097/MOG.0b013e3282f2b0d7 (https://doi.org/10.1097%2FMOG.0b013e3282f2b0d7). PMID 18043226 (https://pubmed.ncbi.nlm.nih.gov/18043226). S2CID 20329070 (https://api.semanticscholar.org/CorpusID:20329070).
22. Song KH, Jung HK, Kim HJ, Koo HS, Kwon YH, Shin HD, et al. (April 2018). "Clinical Practice Guidelines for Irritable Bowel Syndrome in Korea, 2017 Revised Edition" (https://www.ncbi.nlm.nih.gov/pmc/articles/PMC5885719). *Journal of Neurogastroenterology and Motility*. **24** (2): 197–215. doi:10.5056/jnm17145 (https://doi.org/10.5056%2Fjnm17145). PMC 5885719 (https://www.ncbi.nlm.nih.gov/pmc/articles/PMC5885719). PMID 29605976 (https://pubmed.ncbi.nlm.nih.gov/29605976).
23. Rose S, Young MA, Reynolds JC (September 1998). "Gastrointestinal manifestations of scleroderma". *Gastroenterology Clinics of North America*. **27** (3): 563–94. doi:10.1016/S0889-8553(05)70021-2 (https://doi.org/10.1016%2FS0889-8553%2805%2970021-2). PMID 9891698 (https://pubmed.ncbi.nlm.nih.gov/9891698).
24. Kongara KR, Soffer EE (January 2000). "Intestinal motility in small bowel diverticulosis: a case report and review of the literature". *Journal of Clinical Gastroenterology*. **30** (1): 84–6. doi:10.1097/00004836-200001000-00017 (https://doi.org/10.1097%2F00004836-200001000-00017). PMID 10636218 (https://pubmed.ncbi.nlm.nih.gov/10636218).
25. Dukowicz AC, Lacy BE, Levine GM (February 2007). "Small intestinal bacterial overgrowth: a comprehensive review" (https://www.ncbi.nlm.nih.gov/pmc/articles/PMC3099351). *Gastroenterology & Hepatology*. **3** (2): 112–22. PMC 3099351 (https://www.ncbi.nlm.nih.gov/pmc/articles/PMC3099351). PMID 21960820 (https://pubmed.ncbi.nlm.nih.gov/21960820).
26. Lo WK, Chan WW (May 2013). "Proton pump inhibitor use and the risk of small intestinal bacterial overgrowth: a meta-analysis". *Clinical Gastroenterology and Hepatology*. **11** (5): 483–90. doi:10.1016/j.cgh.2012.12.011 (https://doi.org/10.1016%2Fj.cgh.2012.12.011). PMID 23270866 (https://pubmed.ncbi.nlm.nih.gov/23270866).
27. Abell TL, Minocha A (April 2006). "Gastrointestinal complications of bariatric surgery: diagnosis and therapy". *The American Journal of the Medical Sciences*. **331** (4): 214–8. doi:10.1097/00000441-200604000-00008 (https://doi.org/10.1097%2F00000441-200604000-00008). PMID 16617237 (https://pubmed.ncbi.nlm.nih.gov/16617237). S2CID 25920218 (https://api.semanticscholar.org/CorpusID:25920218).
28. Craig RM, Atkinson AJ (July 1988). "D-xylose testing: a review". *Gastroenterology*. **95** (1): 223–31. doi:10.1016/0016-5085(88)90318-6 (https://doi.org/10.1016%2F0016-5085%2888%2990318-6). PMID 3286361 (https://pubmed.ncbi.nlm.nih.gov/3286361).

29. Singh VV, Toskes PP (February 2004). "Small Bowel Bacterial Overgrowth: Presentation, Diagnosis, and Treatment". *Current Treatment Options in Gastroenterology*. **7** (1): 19–28. doi:10.1007/s11938-004-0022-4 (https://doi.org/10.1007%2Fs11938-004-0022-4). PMID 14723835 (https://pubmed.ncbi.nlm.nih.gov/14723835). S2CID 10045342 (https://api.semanticscholar.org/CorpusID:10045342).
30. Goulet O, Joly F (September 2010). "[Intestinal microbiota in short bowel syndrome]". *Gastroenterologie Clinique et Biologique*. 34 Suppl 1: S37–43. doi:10.1016/S0399-8320(10)70019-1 (https://doi.org/10.1016%2FS0399-8320%2810%2970019-1). PMID 20889003 (https://pubmed.ncbi.nlm.nih.gov/20889003).
31. Bondarenko VM, Lykova EA, Matsulevich TV (2006). "[Microecological aspects of small intestinal bacterial overgrowth syndrome]". *Zhurnal Mikrobiologii, Epidemiologii, I Immunobiologii* (in Russian) (6): 57–63. PMID 17163142 (https://pubmed.ncbi.nlm.nih.gov/17163142).
32. Bures J, Cyrany J, Kohoutova D, Förstl M, Rejchrt S, Kvetina J, et al. (June 2010). "Small intestinal bacterial overgrowth syndrome" (https://www.ncbi.nlm.nih.gov/pmc/articles/PMC2890937). *World Journal of Gastroenterology*. **16** (24): 2978–90. doi:10.3748/wjg.v16.i24.2978 (https://doi.org/10.3748%2Fwjg.v16.i24.2978). PMC 2890937 (https://www.ncbi.nlm.nih.gov/pmc/articles/PMC2890937). PMID 20572300 (https://pubmed.ncbi.nlm.nih.gov/20572300).
33. Meier R, Burri E, Steuerwald M (September 2003). "The role of nutrition in diarrhoea syndromes". *Current Opinion in Clinical Nutrition and Metabolic Care*. **6** (5): 563–7. doi:10.1097/00075197-200309000-00010 (https://doi.org/10.1097%2F00075197-200309000-00010). PMID 12913674 (https://pubmed.ncbi.nlm.nih.gov/12913674). S2CID 24535656 (https://api.semanticscholar.org/CorpusID:24535656).
34. Rolfe RD (February 2000). "The role of probiotic cultures in the control of gastrointestinal health" (https://doi.org/10.1093%2Fjn%2F130.2.396S). *The Journal of Nutrition*. **130** (2S Suppl): 396S–402S. doi:10.1093/jn/130.2.396S (https://doi.org/10.1093%2Fjn%2F130.2.396S). PMID 10721914 (https://pubmed.ncbi.nlm.nih.gov/10721914).
35. Bested AC, Logan AC, Selhub EM (March 2013). "Intestinal microbiota, probiotics and mental health: from Metchnikoff to modern advances: part III – convergence toward clinical trials" (https://www.ncbi.nlm.nih.gov/pmc/articles/PMC3605358). *Gut Pathogens*. **5** (1): 4. doi:10.1186/1757-4749-5-4 (https://doi.org/10.1186%2F1757-4749-5-4). PMC 3605358 (https://www.ncbi.nlm.nih.gov/pmc/articles/PMC3605358). PMID 23497650 (https://pubmed.ncbi.nlm.nih.gov/23497650).
36. Rezaie A, Pimentel M, Rao SS (February 2016). "How to Test and Treat Small Intestinal Bacterial Overgrowth: an Evidence-Based Approach". *Current Gastroenterology Reports*. **18** (2): 8. doi:10.1007/s11894-015-0482-9 (https://doi.org/10.1007%2Fs11894-015-0482-9). PMID 26780631 (https://pubmed.ncbi.nlm.nih.gov/26780631). S2CID 207329305 (https://api.semanticscholar.org/CorpusID:207329305).
37. Krajicek EJ, Hansel SL (December 2016). "Small Intestinal Bacterial Overgrowth: A Primary Care Review" (https://doi.org/10.1016%2Fj.mayocp.2016.07.025). *Mayo Clinic Proceedings*. **91** (12): 1828–1833. doi:10.1016/j.mayocp.2016.07.025 (https://doi.org/10.1016%2Fj.mayocp.2016.07.025). PMID 27916156 (https://pubmed.ncbi.nlm.nih.gov/27916156).
38. Buchman A (2006). *Clinical nutrition in gastrointestinal diseas* (https://books.google.com/books?id=lo4lurHUYWcC&pg=PA277). Thorofare, NJ: SLACK. p. 277. ISBN 978-1-55642-697-1.
39. Talley NJ, Kane S, Wallace MB (2010). *Practical Gastroenterology and Hepatology: Small and Large Intestine and Pancreas* (https://books.google.com/books?id=NPfdOD93UPEC). Chichester, West Sussex: Wiley-Blackwell. ISBN 978-1-4051-8274-4.
40. Gibson PR, Barrett JS (2010). "The concept of small intestinal bacterial overgrowth in relation to functional gastrointestinal disorders". *Nutrition*. **26** (11–12): 1038–43. doi:10.1016/j.nut.2010.01.005 (https://doi.org/10.1016%2Fj.nut.2010.01.005). PMID 20418060 (https://pubmed.ncbi.nlm.nih.gov/20418060).

External links

Retrieved from "https://en.wikipedia.org/w/index.php?title=Small_intestinal_bacterial_overgrowth&oldid=1091451125"

This page was last edited on 4 June 2022, at 12:06 (UTC).

Text is available under the Creative Commons Attribution-ShareAlike License 3.0; additional terms may apply. By using this site, you agree to the Terms of Use and Privacy Policy. Wikipedia® is a registered trademark of the Wikimedia Foundation, Inc., a non-profit organization.
