# Supplementary material for: Assessment of medical information on irritable bowel syndrome information in Wikipedia and Baidu Encyclopedia: comparative study
Source: PeerJ. 2024 May 24;12:e17264. doi: 10.7717/peerj.17264 (PMC11129691; doi:10.7717/peerj.17264)
Supplement: Data S1 [file peerj-12-17264-s001.zip › σÄƒσoïμò░μì«/Wikipedia/Flatulence - Wikipedia.pdf]

WIKIPEDIA

# Flatulence

**Flatulence**, in humans, is the expulsion of gas from the intestines via the anus, commonly referred to as **farting**. "Flatus" is the medical word for gas generated in the stomach or bowels.[1] A proportion of intestinal gas may be swallowed environmental air, and hence flatus is not entirely generated in the stomach or bowels. The scientific study of this area of medicine is termed **flatology**.[2]

| Flatulence | |
|---|---|
| **Other names** | Farting, breaking wind, passing gas, cutting the cheese, cutting one loose, ripping one, guffing, chuffing, trumping, pumping, blowing off, letting off, foisting |

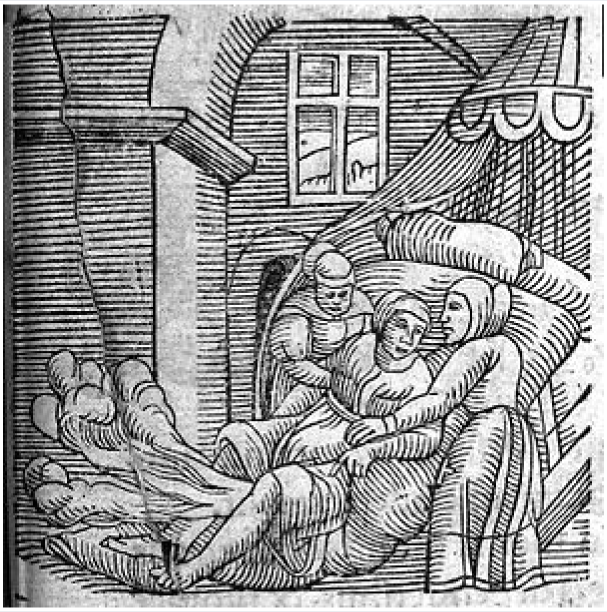

Illustration of man suffering from "wind"

| | |
|---|---|
| **Specialty** | Gastroenterology |

Flatus is brought to the rectum and pressurized by muscles in the intestines. It is normal to pass flatus ("to fart"), though volume and frequency vary greatly among individuals. It is also normal for intestinal gas to have a feculent or unpleasant odor, which may be intense. The noise commonly associated with flatulence ("blowing a raspberry") is produced by the anus and buttocks, which act together in a manner similar to that of an embouchure. Both the sound and odor are sources of embarrassment, annoyance or amusement (flatulence humor).

There are several general symptoms related to intestinal gas: pain, bloating and abdominal distension, excessive flatus volume, excessive flatus odor, and gas incontinence. Furthermore, eructation (colloquially known as "burping") is sometimes included under the topic of flatulence.[3] When excessive or malodorous, flatus can be a sign of a health disorder, such as irritable bowel syndrome, celiac disease or lactose intolerance.[4]

## Contents


Terminology

Signs and symptoms

- Bloating and pain
- Excessive volume
- Odor
- Incontinence of flatus

Cause

Mechanism

- Production, composition, and odor
- Volume and intestinal gas dynamics

Management
- Pain and bloating
- Volume
- Odor
- Incontinence

Society and culture
- Environmental impact
- Entertainment
- Religion

Personal experiences

See also

References
- Citations
- General and cited references

External links

# Terminology

0:02

Sound of a human flatus

Non-medical definitions of the term include "the uncomfortable condition of having gas in the stomach and bowels", or "a state of excessive gas in the alimentary canal". These definitions highlight that many people consider "bloating", abdominal distension or increased volume of intestinal gas, to be synonymous with the term flatulence (although this is technically inaccurate).

Colloquially, flatulence may be referred to as "farting", "pumping", "trumping",[5] "blowing off", "pooting", "passing gas", "breaking wind", "backfiring", or simply (in American English) "gas" or (British English) "wind". Derived terms include vaginal flatulence, otherwise known as a *queef*.

# Signs and symptoms

Generally speaking, there are four different types of complaints that relate to intestinal gas, which may present individually or in combination.

## Bloating and pain

Patients may complain of bloating as abdominal distension, discomfort and pain from "trapped wind". In the past, functional bowel disorders such as irritable bowel syndrome that produced symptoms of bloating were attributed to increased production of intestinal gas.

However, three significant pieces of evidence refute this theory. First, in normal subjects, even very high rates of gas infusion into the small intestine (30 mL/min) is tolerated without complaints of pain or bloating and harmlessly passed as flatus per rectum.[6] Secondly, studies aiming to quantify the total volume of gas produced by patients with irritable bowel syndrome (some including gas emitted from the mouth by eructation) have consistently failed to demonstrate increased volumes compared to healthy subjects. The proportion of hydrogen produced may be increased in some

patients with irritable bowel syndrome, but this does not affect the total volume.[7] Thirdly, the volume of flatus produced by patients with irritable bowel syndrome who have pain and abdominal distension would be tolerated in normal subjects without any complaints of pain.

Patients who complain of bloating frequently can be shown to have objective increases in abdominal girth, often increased throughout the day and then resolving during sleep. The increase in girth combined with the fact that the total volume of flatus is not increased led to studies aiming to image the distribution of intestinal gas in patients with bloating. They found that gas was not distributed normally in these patients: there was segmental gas pooling and focal distension.[6] In conclusion, abdominal distension, pain and bloating symptoms are the result of abnormal intestinal gas dynamics rather than increased flatus production.

## Excessive volume

The normal range of volumes of flatus in normal individuals varies hugely (476–1,491 mL/24 h).[2] All intestinal gas is either swallowed environmental air, present intrinsically in foods and beverages, or the result of gut fermentation.

Swallowing small amounts of air occurs while eating and drinking. This is emitted from the mouth by eructation (burping) and is normal. Excessive swallowing of environmental air is called aerophagia, and has been shown in a few case reports to be responsible for increased flatus volume. This is, however, considered a rare cause of increased flatus volume. Gases contained in food and beverages are likewise emitted largely through eructation, e.g., carbonated beverages.

Endogenously produced intestinal gases make up 74 percent of flatus in normal subjects. The volume of gas produced is partially dependent upon the composition of the intestinal microbiota, which is normally very resistant to change, but is also very different in different individuals. Some patients are predisposed to increased endogenous gas production by virtue of their gut microbiota composition.[6] The greatest concentration of gut bacteria is in the colon, while the small intestine is normally nearly sterile. Fermentation occurs when unabsorbed food residues arrive in the colon.

Therefore, even more than the composition of the microbiota, diet is the primary factor that dictates the volume of flatus produced.[6] Diets that aim to reduce the amount of undigested fermentable food residues arriving in the colon have been shown to significantly reduce the volume of flatus produced. Again, increased volume of intestinal gas will not cause bloating and pain in normal subjects. Abnormal intestinal gas dynamics will create pain, distension, and bloating, regardless of whether there is high or low total flatus volume.

## Odor

Although flatus possesses an odor, this may be abnormally increased in some patients and cause social distress to the patient. Increased odor of flatus presents a distinct clinical issue from other complaints related to intestinal gas.[8] Some patients may exhibit over-sensitivity to bad flatus odor, and in extreme forms, olfactory reference syndrome may be diagnosed. Recent informal research found a correlation between flatus odor and both loudness and humidity content.[9]

## Incontinence of flatus

"Gas incontinence" could be defined as loss of voluntary control over the passage of flatus. It is a recognised subtype of faecal incontinence, and is usually related to minor disruptions of the continence mechanisms. Some consider gas incontinence to be the first, sometimes only, symptom of faecal incontinence.[10]

# Cause

Intestinal gas is composed of varying quantities of exogenous sources and endogenous sources.[11] The exogenous gases are swallowed (aerophagia) when eating or drinking or increased swallowing during times of excessive salivation (as might occur when nauseated or as the result of gastroesophageal reflux disease). The endogenous gases are produced either as a by-product of digesting certain types of food, or of incomplete digestion, as is the case during steatorrhea. Anything that causes food to be incompletely digested by the stomach or small intestine may cause flatulence when the material arrives in the large intestine, due to fermentation by yeast or prokaryotes normally or abnormally present in the gastrointestinal tract.

Flatulence-producing foods are typically high in certain polysaccharides, especially oligosaccharides such as inulin. Those foods include beans, lentils, dairy products, onions, garlic, spring onions, leeks, turnips, swedes, radishes, sweet potatoes, potatoes, cashews, Jerusalem artichokes, oats, wheat, and yeast in breads. Cauliflower, broccoli, cabbage, Brussels sprouts and other cruciferous vegetables that belong to the genus *Brassica* are commonly reputed to not only increase flatulence, but to increase the pungency of the flatus.[12][13]

In beans, endogenous gases seem to arise from complex oligosaccharides (carbohydrates) that are particularly resistant to digestion by mammals, but are readily digestible by microorganisms (methane-producing archaea; *Methanobrevibacter smithii*) that inhabit the digestive tract. These oligosaccharides pass through the small intestine largely unchanged, and when they reach the large intestine, bacteria ferment them, producing copious amounts of flatus.[14]

When excessive or malodorous, flatus can be a sign of a health disorder, such as irritable bowel syndrome, celiac disease, non-celiac gluten sensitivity or lactose intolerance. It can also be caused by certain medicines, such as ibuprofen, laxatives, antifungal medicines or statins.[4][15] Some infections, such as giardiasis, are also associated with flatulence.[16]

Interest in the causes of flatulence was spurred by high-altitude flight and human spaceflight; the low atmospheric pressure, confined conditions, and stresses peculiar to those endeavours were cause for concern.[14] In the field of mountaineering, the phenomenon of high altitude flatus expulsion was first recorded over two hundred years ago.

# Mechanism

## Production, composition, and odor

Flatus (intestinal gas) is mostly produced as a byproduct of bacterial fermentation in the gastrointestinal (GI) tract, especially the colon.[17] There are reports of aerophagia (excessive air swallowing) causing excessive intestinal gas, but this is considered rare.[18]

Over 99% of the volume of flatus is composed of odorless gases.[2] These include oxygen, nitrogen, carbon dioxide, hydrogen and methane. Nitrogen is not produced in the gut, but a component of environmental air. Patients who have excessive intestinal gas that is mostly composed of nitrogen

have aerophagia.[19] Hydrogen, carbon dioxide and methane are all produced in the gut and contribute 74% of the volume of flatus in normal subjects.[20] Methane and hydrogen are flammable, and so flatus can be ignited if it contains adequate amounts of these components.[21]

Not all humans produce flatus that contains methane. For example, in one study of the faeces of nine adults, only five of the samples contained archaea capable of producing methane.[22] The prevalence of methane over hydrogen in human flatus may correlate with obesity, constipation and irritable bowel syndrome, as archaea that oxidise hydrogen into methane promote the metabolism's ability to absorb fatty acids from food.[23]

The remaining trace (<1% volume) compounds contribute to the odor of flatus. Historically, compounds such as indole, skatole, ammonia and short chain fatty acids were thought to cause the odor of flatus. More recent evidence proves that the major contribution to the odor of flatus comes from a combination of volatile sulfur compounds.[2][24] Hydrogen sulfide, methyl mercaptan (also known as methanethiol), dimethyl sulfide, dimethyl disulfide and dimethyl trisulfide are present in flatus. The benzopyrrole volatiles indole and skatole have an odor of mothballs, and therefore probably do not contribute greatly to the characteristic odor of flatus.

In one study, hydrogen sulfide concentration was shown to correlate convincingly with perceived bad odor of flatus, followed by methyl mercaptan and dimethyl sulfide.[19] This is supported by the fact that hydrogen sulfide may be the most abundant volatile sulfur compound present. These results were generated from subjects who were eating a diet high in pinto beans to stimulate flatus production.

Others report that methyl mercaptan was the greatest contributor to the odor of flatus in patients not under any specific dietary alterations.[2] It has now been demonstrated that methyl mercaptan, dimethyl sulfide, and hydrogen sulfide (described as decomposing vegetables, unpleasantly sweet/wild radish and rotten eggs respectively) are all present in human flatus in concentrations above their smell perception thresholds.[2]

It is recognized that increased dietary sulfur-containing amino acids significantly increases the odor of flatus. It is therefore likely that the odor of flatus is created by a combination of volatile sulfur compounds, with minimal contribution from non-sulfur volatiles.[19] This odor can also be caused by the presence of large numbers of microflora bacteria or the presence of faeces in the rectum. Diets high in protein, especially sulfur-containing amino acids, have been demonstrated to significantly increase the odor of flatus.

## Volume and intestinal gas dynamics

Normal flatus volume is 476 to 1491 mL per 24 hours.[2][17] This variability between individuals is greatly dependent upon diet. Similarly, the number of flatus episodes per day is variable; the normal range is given as 8–20 per day.[19] The volume of flatus associated with each flatulence event again varies (5–375 mL).[2][17][20] The volume of the first flatulence upon waking in the morning is significantly larger than those during the day.[2] This may be due to buildup of intestinal gas in the colon during sleep, the peak in peristaltic activity in the first few hours after waking or the strong prokinetic effect of rectal distension on the rate of transit of intestinal gas.[6] It is now known that gas is moved along the gut independently of solids and liquids, and this transit is more efficient in the erect position compared to when supine.[6] It is thought that large volumes of intestinal gas present low resistance, and can be propelled by subtle changes in gut tone, capacitance and proximal contraction and distal relaxation. This process is thought not to affect solid and liquid intra-lumenal contents.[6]

Researchers investigating the role of sensory nerve endings in the anal canal did not find them to be essential for retaining fluids in the anus, and instead speculate that their role may be to distinguish between flatus and faeces, thereby helping detect a need to defecate or to signal the end of defecation.[25]

The sound varies depending on the tightness of the sphincter muscle and velocity of the gas being propelled, as well as other factors, such as water and body fat. The auditory pitch (sound) of the flatulence outburst can also be affected by the anal embouchure. Among humans, flatulence occasionally happens accidentally, such as incidentally to coughing or sneezing or during orgasm; on other occasions, flatulence can be voluntarily elicited by tensing the rectum or "bearing down" on stomach or bowel muscles and subsequently relaxing the anal sphincter, resulting in the expulsion of flatus.

# Management

Since problems involving intestinal gas present as different (but sometimes combined) complaints, the management is cause-related.

## Pain and bloating

While not affecting the production of the gases themselves, surfactants (agents that lower surface tension) can reduce the disagreeable sensations associated with flatulence, by aiding the dissolution of the gases into liquid and solid faecal matter.[26] Preparations containing simethicone reportedly operate by promoting the coalescence of smaller bubbles into larger ones more easily passed from the body, either by burping or flatulence. Such preparations do not decrease the total amount of gas generated in or passed from the colon, but make the bubbles larger and thereby allowing them to be passed more easily.[26]

Other drugs including prokinetics, lubiprostone, antibiotics and probiotics are also used to treat bloating in patients with functional bowel disorders such as irritable bowel syndrome, and there is some evidence that these measures may reduce symptoms.[27]

A flexible tube, inserted into the rectum, can be used to collect intestinal gas in a flatus bag. This method is occasionally needed in a hospital setting, when the patient is unable to pass gas normally.[28]

## Volume

One method of reducing the volume of flatus produced is dietary modification, reducing the amount of fermentable carbohydrates. This is the theory behind diets such as the low-FODMAP diet (a diet low in fermentable oligosaccharides, disaccharides, monosaccharides, alcohols, and polyols).[29]

Most starches, including potatoes, corn, noodles, and wheat, produce gas as they are broken down in the large intestine.[11] Intestinal gas can be reduced by fermenting the beans, and making them less gas-inducing, or by cooking them in the liquor from a previous batch. For example, the fermented bean product miso is less likely to produce as much intestinal gas. Some legumes also stand up to prolonged cooking, which can help break down the oligosaccharides into simple sugars. Fermentative lactic acid bacteria such as *Lactobacillus casei* and *Lactobacillus plantarum* reduce flatulence in the human intestinal tract.[30]

Probiotics (live yogurt, kefir, etc.) are reputed to reduce flatulence when used to restore balance to the normal intestinal flora.[31] Live (bioactive) yogurt contains, among other lactic bacteria, *Lactobacillus acidophilus*, which may be useful in reducing flatulence. *L. acidophilus* may make the intestinal environment more acidic, supporting a natural balance of the fermentative processes. *L. acidophilus* is available in supplements. Prebiotics, which generally are non-digestible oligosaccharides, such as fructooligosaccharide, generally increase flatulence in a similar way as described for lactose intolerance.

Digestive enzyme supplements may significantly reduce the amount of flatulence caused by some components of foods not being digested by the body and thereby promoting the action of microbes in the small and large intestines. It has been suggested that alpha-galactosidase enzymes, which can digest certain complex sugars, are effective in reducing the volume and frequency of flatus.[32] The enzymes alpha-galactosidase, lactase, amylase, lipase, protease, cellulase, glucoamylase, invertase, malt diastase, pectinase, and bromelain are available, either individually or in combination blends, in commercial products.

The antibiotic rifaximin, often used to treat diarrhea caused by the microorganism *E. coli*, may reduce both the production of intestinal gas and the frequency of flatus events.[33]

## Odor

### Bismuth

The odor created by flatulence is commonly treated with bismuth subgallate, available over-the-counter in the US as Devrom. Bismuth subgallate is commonly used by individuals who have had ostomy surgery, bariatric surgery, faecal incontinence and irritable bowel syndrome.[34][35] Bismuth subsalicylate is a compound that binds hydrogen sulfide, and one study reported a dose of 524 mg four times a day for 3–7 days bismuth subsalicylate yielded a >95% reduction in faecal hydrogen sulfide release in both humans and rats.[36] Another bismuth compound, bismuth subnitrate was also shown to bind to hydrogen sulfide.[37] Another study showed that bismuth acted synergistically with various antibiotics to inhibit sulfate-reducing gut bacteria and sulfide production.[38] Some authors proposed a theory that hydrogen sulfide was involved in the development of ulcerative colitis and that bismuth might be helpful in the management of this condition.[39] However, bismuth administration in rats did not prevent them from developing ulcerative colitis despite reduced hydrogen sulfide production.[39] Also, evidence suggests that colonic hydrogen sulfide is largely present in bound forms, probably sulfides of iron and other metals.[2] Rarely, serious bismuth toxicity may occur with higher doses.[40]

### Activated charcoal

Despite being an ancient treatment for various digestive complaints, activated charcoal did not produce reduction in both the total flatus volume nor the release of sulfur-containing gasses, and there was no reduction in abdominal symptoms (after 0.52 g activated charcoal four times a day for one week).[41] The authors suggested that saturation of charcoal binding sites during its passage through the gut was the reason for this. A further study concluded that activated charcoal (4 g) does not influence gas formation in vitro or in vivo.[42] Other authors reported that activated charcoal was effective. A study in 8 dogs concluded activated charcoal (unknown oral dose) reduced hydrogen sulfide levels by 71%. In combination with yucca schidigera, and zinc acetate, this was increased to an 86% reduction in hydrogen sulfide, although flatus volume and number was unchanged.[43] An early study reported activated charcoal (unknown oral dose) prevented a large increase in the number of flatus events and increased breath hydrogen concentrations that normally occur following a gas-producing meal.[44]

### Garments and external devices

In 1998, Chester "Buck" Weimer of Pueblo, Colorado, received a patent for the first undergarment that contained a replaceable charcoal filter. The undergarments are air-tight and provide a pocketed escape hole in which a charcoal filter can be inserted.[45] In 2001 Weimer received the Ig Nobel Prize for Biology for his invention.[46]

A similar product was released in 2002, but rather than an entire undergarment, consumers are able to purchase an insert similar to a pantiliner that contains activated charcoal.[47] The inventors, Myra and Brian Conant of Mililani, Hawaii, still claim on their website to have discovered the undergarment product in 2002 (four years after Chester Weimer filed for a patent for his product), but state that their tests "concluded" that they should release an insert instead.[48]

## Incontinence

Flatus incontinence where there is involuntary passage of gas, is a type of faecal incontinence, and is managed similarly.

# Society and culture

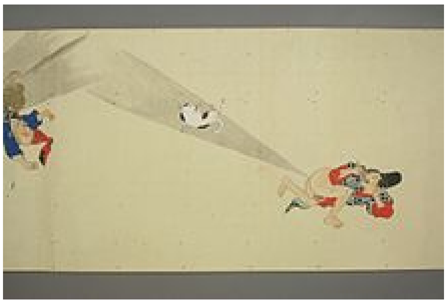

*He-gassen* (detail), an art scroll depicting a battle of flatulence, from Japan during the Edo period

In many cultures, flatulence in public is regarded as embarrassing, but, depending on context, may also be considered humorous. People will often strain to hold in the passing of gas when in polite company, or position themselves to silence or conceal the passing of gas. In other cultures, it may be no more embarrassing than coughing.

While the act of passing flatus in some cultures is generally considered to be an unfortunate occurrence in public settings, flatulence may, in casual circumstances and especially among children, be used as either a humorous supplement to a joke ("pull my finger"), or as a comic activity in and of itself. The social acceptability of flatulence-based humour in entertainment and the mass media varies over the course of time and between cultures. A sufficient number of entertainers have performed using their flatus to lead to the coining of the term flatulist. The whoopee cushion is a joking device invented in the early 20th century for simulating a fart. In 2008, a farting application for the iPhone earned nearly $10,000 in one day.[49]

A farting game named *Touch Wood* was documented by John Gregory Bourke in the 1890s.[50] It existed under the name of *Safety* in the 20th century in the U.S., and has been found being played in 2011.[50]

In January 2011, the Malawi Minister of Justice, George Chaponda, said that Air Fouling Legislation would make public "farting" illegal in his country. When reporting the story, the media satirised Chaponda's statement with punning headlines. Later, the minister withdrew his statement.[51]

## Environmental impact

Flatulence is often blamed as a significant source of greenhouse gases, owing to the erroneous belief that the methane released by livestock is in the flatus.[53] While livestock account for around 20% of global methane emissions,[54] 90–95% of that is released by exhaling or burping.[55] In

cows, gas and burps are produced by methane-generating microbes called methanogens, that live inside the cow's digestive system. Proposals for reducing methane production in cows include the feeding of supplements such as oregano and seaweed, and the genetic engineering of gut biome microbes to produce less methane.[52]

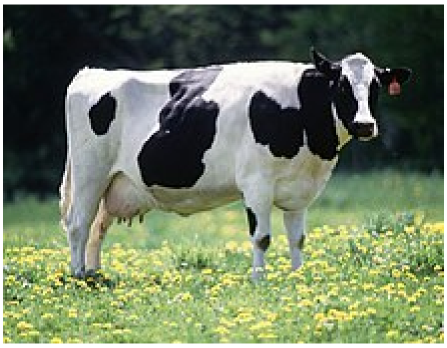

The flatulence of cows is only a small portion (around one-twentieth) of cows' methane release. Cows also *burp* methane, due to the physiology of their digestive systems.[52]

Since New Zealand produces large amounts of agricultural products, it is in the unique position of having high methane emissions from livestock compared to other greenhouse gas sources. The New Zealand government is a signatory to the Kyoto Protocol and therefore attempts are being made to reduce greenhouse emissions. To achieve this, an agricultural emissions research levy was proposed, which promptly became known as a "fart tax" or "flatulence tax". It encountered opposition from farmers, farming lobby groups and opposition politicians.

### Entertainment

Historical comment on the ability to fart at will is observed as early as Saint Augustine's *The City of God* (5th century A.D.). Augustine mentions men who "have such command of their bowels, that they can break wind continuously at will, so as to produce the effect of singing".[56] Intentional passing of gas and its use as entertainment for others appear to have been somewhat well known in pre-modern Europe, according to mentions of it in medieval and later literature, including Rabelais.

Le Pétomane ("the Fartomaniac") was a famous French performer in the 19th century who, as well as many professional farters before him, did flatulence impressions and held shows. The performer Mr. Methane carries on le Pétomane's tradition today. Also, a 2002 fiction film *Thunderpants* revolves around a boy named Patrick Smash who has an ongoing flatulence problem from the time of his birth.[57]

Since the 1970s, farting has increasingly been featured in film, especially comedies such as *Blazing Saddles* and *Scooby-Doo*.[58]

### Religion

In Islam, flatulence, if audible or odorous, invalidates *wudu* (ritual purity).[59]

## Personal experiences

People find other peoples' flatus unpleasant, but are unfazed by, and may even enjoy, the scent of their own.[60] While there has been little research carried out upon the subject, some speculative guesses have been made as to why this might be so. For example, one explanation for this phenomenon is that people are very familiar with the scent of their own flatus, and that survival in nature may depend on the detection of and reaction to foreign scents.[61]

## See also

- Antiflatulent
- Armpit fart

- Borborygmus
- Eproctophilia
- Fart lighting
- Flatulence humor
- *The Gas We Pass*
- Tympany

# References

## Citations


1. "Medical Dictionary: Flatus" (http://www.merriam-webster.com/medlineplus/flatus). *Merriam-Webster*. Retrieved August 24, 2015.
2. Tangerman, Albert (October 1, 2009). "Measurement and biological significance of the volatile sulfur compounds hydrogen sulfide, methanethiol and dimethyl sulfide in various biological matrices". *Journal of Chromatography B*. **877** (28): 3366–77. doi:10.1016/j.jchromb.2009.05.026 (https://doi.org/10.1016%2Fj.jchromb.2009.05.026). PMID 19505855 (https://pubmed.ncbi.nlm.nih.gov/19505855).
3. Parveen Kumar; Michael L. Clark, eds. (2005). *Kumar & Clark Clinical Medicine* (6th ed.). Edinburgh: Saunders. p. 266. ISBN 0702027634.
4. "Flatulence" (https://www.nhs.uk/conditions/flatulence/). NHS. December 12, 2017.
5. "trump" (https://web.archive.org/web/20170623075303/https://en.oxforddictionaries.com/definition/trump). *Oxford English Dictionary*. Archived from the original (https://en.oxforddictionaries.com/definition/trump) on June 23, 2017.
6. Azpiroz, F (July 1, 2005). "Intestinal gas dynamics: mechanisms and clinical relevance" (https://www.ncbi.nlm.nih.gov/pmc/articles/PMC1774596). *Gut*. **54** (7): 893–95. doi:10.1136/gut.2004.048868 (https://doi.org/10.1136%2Fgut.2004.048868). PMC 1774596 (https://www.ncbi.nlm.nih.gov/pmc/articles/PMC1774596). PMID 15951528 (https://pubmed.ncbi.nlm.nih.gov/15951528).
7. King, TS; Elia, M; Hunter, JO (October 10, 1998). "Abnormal colonic fermentation in irritable bowel syndrome". *The Lancet*. **352** (9135): 1187–9. doi:10.1016/S0140-6736(98)02146-1 (https://doi.org/10.1016%2FS0140-6736%2898%2902146-1). PMID 9777836 (https://pubmed.ncbi.nlm.nih.gov/9777836). S2CID 1750363 (https://api.semanticscholar.org/CorpusID:1750363).
8. Bailey, J; Carter, NJ; Neher, JO (June 15, 2009). "FPIN's Clinical Inquiries: Effective management of flatulence". *American Family Physician*. **79** (12): 1098–100. PMID 19530642 (https://pubmed.ncbi.nlm.nih.gov/19530642).
9. Brain, Monkey (October 8, 2020). "Bimodal Farts" (https://flatology.com/bimodal-farts/). *The Invisible College of Experimental Flatology*.
10. Bruce G. Wolff; James W. Fleshman; David E. Beck; John H. Pemberton; Steven D. Wexner, eds. (2007), *The ASCRS textbook of colon and rectal surgery*, New York: Springer Publishing, ISBN 978-0-387-24846-2
11. "Gas in the Digestive Tract" (http://www.niddk.nih.gov/health-information/health-topics/digestive-diseases/gas/Pages/facts.aspx). National Institute of Diabetes and Digestive and Kidney Diseases. Retrieved August 24, 2015.
12. "Flatulence: Causes, remedies, and complications" (https://www.medicalnewstoday.com/articles/7622.php). *Medical News Today*. Retrieved April 17, 2018.
13. "Paleo Foods: Brassicas (and not Just the Ones you Know) | Paleo Leap" (https://paleoleap.com/eat-brassicas-just-ones-know/). *Paleo Leap | Paleo diet Recipes & Tips*. September 14, 2014. Retrieved April 17, 2018.
14. McGee, Harold (1984). *On Food and Cooking*. Scribner. pp. 257–58. ISBN 0-684-84328-5.

15. Czaja-Bulsa G (April 2015). "Non coeliac gluten sensitivity — A new disease with gluten intolerance" (https://doi.org/10.1016%2Fj.clnu.2014.08.012). *Clin Nutr* (Review). **34** (2): 189–94. doi:10.1016/j.clnu.2014.08.012 (https://doi.org/10.1016%2Fj.clnu.2014.08.012). PMID 25245857 (https://pubmed.ncbi.nlm.nih.gov/25245857).
16. Flanagan PA (1992). "Giardia — diagnosis, clinical course and epidemiology: A review" (https://www.ncbi.nlm.nih.gov/pmc/articles/PMC2272232). *Epidemiology and Infection*. **109** (1): 1–22. JSTOR 3863897 (https://www.jstor.org/stable/3863897). PMC 2272232 (https://www.ncbi.nlm.nih.gov/pmc/articles/PMC2272232). PMID 1499664 (https://pubmed.ncbi.nlm.nih.gov/1499664).
17. Tomlin, J; Lowis, C; Read, NW (June 1991). "Investigation of normal flatus production in healthy volunteers" (https://www.ncbi.nlm.nih.gov/pmc/articles/PMC1378885). *Gut*. **32** (6): 665–669. doi:10.1136/gut.32.6.665 (https://doi.org/10.1136%2Fgut.32.6.665). PMC 1378885 (https://www.ncbi.nlm.nih.gov/pmc/articles/PMC1378885). PMID 1648028 (https://pubmed.ncbi.nlm.nih.gov/1648028).
18. Hemmink, GJ; Weusten, BL; Bredenoord, AJ; Timmer, R; Smout, AJ (October 2009). "Aerophagia: excessive air swallowing demonstrated by esophageal impedance monitoring". *Clinical Gastroenterology and Hepatology*. **7** (10): 1127–29. doi:10.1016/j.cgh.2009.06.029 (https://doi.org/10.1016%2Fj.cgh.2009.06.029). PMID 19602452 (https://pubmed.ncbi.nlm.nih.gov/19602452).
19. Levitt, MD; Furne, J; Aeolus, MR; Suarez, FL (November 1998). "Evaluation of an extremely flatulent patient: case report and proposed diagnostic and therapeutic approach". *The American Journal of Gastroenterology*. **93** (11): 2276–81. doi:10.1111/j.1572-0241.1998.00635.x (https://doi.org/10.1111%2Fj.1572-0241.1998.00635.x). PMID 9820415 (https://pubmed.ncbi.nlm.nih.gov/9820415). S2CID 20991299 (https://api.semanticscholar.org/CorpusID:20991299).
20. Suarez, F; Furne, J; Springfield, J; Levitt, M (May 1997). "Insights into human colonic physiology obtained from the study of flatus composition". *American Journal of Physiology*. **272** (5 Pt 1): G1028–33. doi:10.1152/ajpgi.1997.272.5.G1028 (https://doi.org/10.1152%2Fajpgi.1997.272.5.G1028). PMID 9176210 (https://pubmed.ncbi.nlm.nih.gov/9176210).
21. Mercer, Bobby (April 18, 2009). *How Do You Light a Fart?: And 150 Other Essential Things Every Guy Should Know about Science* (https://books.google.com/books?id=gV01_pPEO_AC&pg=PT88). Adams Media. p. 71. ISBN 9781440519871. Retrieved October 2, 2014.
22. Miller TL; Wolin MJ; de Macario EC; Macario AJ (1982). "Isolation of *Methanobrevibacter smithii* from human faeces" (https://www.ncbi.nlm.nih.gov/pmc/articles/PMC241804). *Applied and Environmental Microbiology*. **43** (1): 227–32. Bibcode:1982ApEnM..43..227M (https://ui.adsabs.harvard.edu/abs/1982ApEnM..43..227M). doi:10.1128/AEM.43.1.227-232.1982 (https://doi.org/10.1128%2FAEM.43.1.227-232.1982). PMC 241804 (https://www.ncbi.nlm.nih.gov/pmc/articles/PMC241804). PMID 6798932 (https://pubmed.ncbi.nlm.nih.gov/6798932).
23. Pimentel, Mark; Robert P Gunsalus; Satish SC Rao; Husen Zhang (2012). "Methanogens in Human Health and Disease" (http://www.nature.com/ajgsup/journal/v1/n1/full/ajgsup20126a.html). *The American Journal of Gastroenterology Supplements*. **1** (1): 28–33. doi:10.1038/ajgsup.2012.6 (https://doi.org/10.1038%2Fajgsup.2012.6).
24. Suarez, FL; Springfield, J; Levitt, MD (July 1998). "Identification of gases responsible for the odour of human flatus and evaluation of a device purported to reduce this odour" (https://www.ncbi.nlm.nih.gov/pmc/articles/PMC1727181). *Gut*. **43** (1): 100–04. doi:10.1136/gut.43.1.100 (https://doi.org/10.1136%2Fgut.43.1.100). PMC 1727181 (https://www.ncbi.nlm.nih.gov/pmc/articles/PMC1727181). PMID 9771412 (https://pubmed.ncbi.nlm.nih.gov/9771412).

25. Read, M. G.; Read, N. W. (1982). "Role of anorectal sensation in preserving continence" (https://www.ncbi.nlm.nih.gov/pmc/articles/PMC1419736). *Gut*. **23** (4): 345–47. doi:10.1136/gut.23.4.345 (https://doi.org/10.1136%2Fgut.23.4.345). PMC 1419736 (https://www.ncbi.nlm.nih.gov/pmc/articles/PMC1419736). PMID 7076012 (https://pubmed.ncbi.nlm.nih.gov/7076012).
26. Brecević L, Bosan-Kilibarda I, Strajnar F (1994). "Mechanism of antifoaming action of simethicone". *Journal of Applied Toxicology*. **14** (3): 207–11. doi:10.1002/jat.2550140311 (https://doi.org/10.1002%2Fjat.2550140311). PMID 8083482 (https://pubmed.ncbi.nlm.nih.gov/8083482). S2CID 7453218 (https://api.semanticscholar.org/CorpusID:7453218).
27. Schmulson, M; Chang, L (May 2011). "Review article: the treatment of functional abdominal bloating and distension" (https://doi.org/10.1111%2Fj.1365-2036.2011.04637.x). *Alimentary Pharmacology & Therapeutics*. **33** (10): 1071–86. doi:10.1111/j.1365-2036.2011.04637.x (https://doi.org/10.1111%2Fj.1365-2036.2011.04637.x). PMID 21488913 (https://pubmed.ncbi.nlm.nih.gov/21488913). S2CID 20718176 (https://api.semanticscholar.org/CorpusID:20718176).
28. "Flatus tube" (http://www.gastrotraining.com/product-information/colorectal/flatus-tube). *Gastro Training*. Retrieved April 3, 2016.
29. Staudacher, HM; Whelan, K; Irving, PM; Lomer, MC (October 2011). "Comparison of symptom response following advice for a diet low in fermentable carbohydrates (FODMAPs) versus standard dietary advice in patients with irritable bowel syndrome". *Journal of Human Nutrition and Dietetics*. **24** (5): 487–95. doi:10.1111/j.1365-277X.2011.01162.x (https://doi.org/10.1111%2Fj.1365-277X.2011.01162.x). PMID 21615553 (https://pubmed.ncbi.nlm.nih.gov/21615553).
30. "Study shows secret to gas-free beans" (https://web.archive.org/web/20060502110835/http://reuters.myway.com/article/20060426/2006-04-26T170153Z_01_N25328545_RTRIDST_0_ODD-BEANS-DC-DC.html). April 26, 2006. Archived from the original (http://reuters.myway.com/article/20060426/2006-04-26T170153Z_01_N25328545_RTRIDST_0_ODD-BEANS-DC-DC.html) on May 2, 2006. Retrieved September 10, 2007.
31. Rubin, Jordan S.; Joseph Brasco (2003). *Restoring Your Digestive Health* (https://archive.org/details/restoringyourdig00rubi). Kensington Books. ISBN 978-0758202826.
32. Ganiats TG; Norcross WA; Halverson AL; Burford PA; Palinkas LA (1994). "Does Beano prevent gas? A double-blind crossover study of oral alpha-galactosidase to treat dietary oligosaccharide intolerance". *The Journal of Family Practice*. **39** (5): 441–45. PMID 7964541 (https://pubmed.ncbi.nlm.nih.gov/7964541).
33. Di Stefano M; Strocchi A; Malservisi S; Veneto G; Ferrieri A; Corazza GR (2000). "Non-absorbable antibiotics for managing intestinal gas production and gas-related symptoms" (https://doi.org/10.1046%2Fj.1365-2036.2000.00808.x). *Alimentary Pharmacology & Therapeutics*. **14** (8): 1001–08. doi:10.1046/j.1365-2036.2000.00808.x (https://doi.org/10.1046%2Fj.1365-2036.2000.00808.x). PMID 10930893 (https://pubmed.ncbi.nlm.nih.gov/10930893). S2CID 19613386 (https://api.semanticscholar.org/CorpusID:19613386).
34. Turnbull G (2005). "The Ostomy Files:The Issue of Oral Medications and a Fecal Ostomy" (https://www.o-wm.com/content/the-issue-oral-medications-and-a-fecal-ostomy). *Ostomy Wound Management*. **51**: 14–16.
35. "Colostomy Guide" (http://www.cancer.org/docroot/CRI/content/CRI_2_6x_Colostomy.asp). January 4, 2006. Retrieved September 10, 2007.
36. Suarez, F.L.; Furne, J.K.; Springfield, J.R.; Levitt, M.D. (1998). "Bismuth subsalicylate markedly decreases hydrogen sulfide release in the human colon". *Gastroenterology*. **114** (5): 923–29. doi:10.1016/S0016-5085(98)81700-9 (https://doi.org/10.1016%2FS0016-5085%2898%2981700-9). PMID 9558280 (https://pubmed.ncbi.nlm.nih.gov/9558280).

37. Levitt, MD; Springfield, J; Furne, J; Koenig, T; Suarez, FL (April 2002). "Physiology of sulfide in the rat colon: use of bismuth to assess colonic sulfide production". *Journal of Applied Physiology*. **92** (4): 1655–60. doi:10.1152/japplphysiol.00907.2001 (https://doi.org/10.1152%2Fjapplphysiol.00907.2001). PMID 11896034 (https://pubmed.ncbi.nlm.nih.gov/11896034). S2CID 37217656 (https://api.semanticscholar.org/CorpusID:37217656).
38. Ohge, H; Furne, JK; Springfield, J; Sueda, T; Madoff, RD; Levitt, MD (November 7, 2003). "The effect of antibiotics and bismuth on fecal hydrogen sulfide and sulfate-reducing bacteria in the rat" (https://doi.org/10.1016%2Fs0378-1097%2803%2900748-1). *FEMS Microbiology Letters*. **228** (1): 137–42. doi:10.1016/s0378-1097(03)00748-1 (https://doi.org/10.1016%2Fs0378-1097%2803%2900748-1). PMID 14612249 (https://pubmed.ncbi.nlm.nih.gov/14612249).
39. Furne, JK; Suarez, FL; Ewing, SL; Springfield, J; Levitt, MD (July 2000). "Binding of hydrogen sulfide by bismuth does not prevent dextran sulfate-induced colitis in rats". *Digestive Diseases and Sciences*. **45** (7): 1439–43. doi:10.1023/A:1005580709390 (https://doi.org/10.1023%2FA%3A1005580709390). PMID 10961726 (https://pubmed.ncbi.nlm.nih.gov/10961726). S2CID 740767 (https://api.semanticscholar.org/CorpusID:740767).
40. Gordon, MF; Abrams, RI; Rubin, DB; Barr, WB; Correa, DD (March 1995). "Bismuth subsalicylate toxicity as a cause of prolonged encephalopathy with myoclonus". *Movement Disorders*. **10** (2): 220–22. doi:10.1002/mds.870100215 (https://doi.org/10.1002%2Fmds.870100215). PMID 7753066 (https://pubmed.ncbi.nlm.nih.gov/7753066). S2CID 11723220 (https://api.semanticscholar.org/CorpusID:11723220).
41. Suarez, FL; Furne, J; Springfield, J; Levitt, MD (January 1999). "Failure of activated charcoal to reduce the release of gases produced by the colonic flora". *The American Journal of Gastroenterology*. **94** (1): 208–12. doi:10.1111/j.1572-0241.1999.00798.x (https://doi.org/10.1111%2Fj.1572-0241.1999.00798.x). PMID 9934757 (https://pubmed.ncbi.nlm.nih.gov/9934757). S2CID 20729051 (https://api.semanticscholar.org/CorpusID:20729051).
42. Potter, T; Ellis, C; Levitt, M (March 1985). "Activated charcoal: in vivo and in vitro studies of effect on gas formation". *Gastroenterology*. **88** (3): 620–24. doi:10.1016/0016-5085(85)90129-5 (https://doi.org/10.1016%2F0016-5085%2885%2990129-5). PMID 3917957 (https://pubmed.ncbi.nlm.nih.gov/3917957).
43. Giffard, CJ; Collins, SB; Stoodley, NC; Butterwick, RF; Batt, RM (March 15, 2001). "Administration of charcoal, Yucca schidigera, and zinc acetate to reduce malodorous flatulence in dogs". *Journal of the American Veterinary Medical Association*. **218** (6): 892–96. doi:10.2460/javma.2001.218.892 (https://doi.org/10.2460%2Fjavma.2001.218.892). PMID 11294313 (https://pubmed.ncbi.nlm.nih.gov/11294313).
44. Hall RG, Jr; Thompson, H; Strother, A (March 1981). "Effects of orally administered activated charcoal on intestinal gas". *The American Journal of Gastroenterology*. **75** (3): 192–96. PMID 7015846 (https://pubmed.ncbi.nlm.nih.gov/7015846).
45. Weimer, Chester (January 14, 1997). "Protective underwear with malodorous flatus filter" (http://patft.uspto.gov/netacgi/nph-Parser?TERM1=5593398&u=/netahtml/srchnum.htm&Sect1=PTO1&Sect2=HITOFF&p=1&r=0&l=50&f=S&d=PALL). Retrieved July 27, 2007.
46. "The 2001 Ig Nobel Prize Winners" (https://web.archive.org/web/20110225074430/http://www.improb.com/ig/ig-pastwinners.html#ig2001). Archived from the original (http://www.improb.com/ig/ig-pastwinners.html#ig2001) on February 25, 2011. Retrieved June 22, 2010.
47. Conant, Brian J.; Myra M. Conant (November 6, 2001). "Flatulence deodorizer" (http://patft.uspto.gov/netacgi/nph-Parser?TERM1=6313371&u=/netahtml/srchnum.htm&Sect1=PTO1&Sect2=HITOFF&p=1&r=0&l=50&f=S&d=PALL). Retrieved September 10, 2007.
48. "About the Inventor" (http://www.flat-d.com/american-inventor.html). Flat-D Innovations Inc. Retrieved September 10, 2007.
49. Chen, Brian X. (December 24, 2008). "iPhone Fart App Rakes in $10,000 a Day" (https://www.wired.com/gadgetlab/2008/12/iphone-fart-app/). *Wired News*.

50. Fishlock, Diana (June 8, 2011). "Penn State professor's essay on farting takes the prize" (http://www.pennlive.com/midstate/index.ssf/2011/06/penn_state_professors_essay_on.html). *The Patriot-News*. Harrisburg, Pennsylvania. Retrieved December 28, 2011. "Trevor Blank [...] found Pennsylvania boys playing "Safety," a farting game, the same as Blank had as a kid in Maryland, and the same game John Bourke documented in the 1890s, when it was called "Touch Wood." Basically, a boy who farts must say "Safety" or touch wood before his friends say another key phrase. If not, they're allowed to punch him. (It usually is boys who make farting a game or a weapon.)"
51. Joe Chibewa (February 4, 2011). "Chaponda: Oops I goofed, you can fart!" (https://web.archive.org/web/20110714032623/http://maravipost.com/index.php?option=com_content&view=article&id=4663%3Achaponda-oops-i-goofed-you-can-fart&catid=74%3Ageneral&Itemid=160#ixzz1D8VQamqG). *Marevi Post*. Archived from the original (http://maravipost.com/index.php?option=com_content&view=article&id=4663:chaponda-oops-i-goofed-you-can-fart&catid=74:general&Itemid=160#ixzz1D8VQamqG) on July 14, 2011. Retrieved March 2, 2011.
52. Kean, Sam (2018). "Tummy Trouble" (https://www.sciencehistory.org/distillations/magazine/tummy-trouble). *Distillations*. Science History Institute. **4** (1): 5. Retrieved June 26, 2018.
53. ABC Southern Queensland: "Could skippy stop cows farting and end global warming?" (http://www.abc.net.au/southqld/stories/s1560903.htm?backyard) February 3, 2006. Example of error. Although the article doesn't specify whether the methane is released by flatulence or eructation, it appears the headline-writer assumes it's through flatulence. Archived (https://web.archive.org/web/20071014025622/http://www.abc.net.au/southqld/stories/s1560903.htm?backyard) October 14, 2007, at the Wayback Machine
54. Nowak, Rachel (September 24, 2004). "Burp vaccine cuts greenhouse gas" (https://www.newscientist.com/article.ns?id=dn6431). *New Scientist*.
55. "Bovine belching called udderly serious gas problem — Global warming concerns spur effort to cut methane." (http://www.mycattle.com/health/dsp_health_article.cfm?storyid=10045) Archived (https://web.archive.org/web/20040813063303/http://www.mycattle.com/health/dsp_health_article.cfm?storyid=10045) August 13, 2004, at the Wayback Machine By Gary Polakovic. *Los Angeles Times*, July 13, 2003.
56. *The City of God Against the Pagans*. Philip Levine, editor and translator. Cambridge, Massachusetts: Harvard University Press. 1966., XIV.24.
57. Hewitt, Peter (May 24, 2002), *Thunderpants* (https://www.imdb.com/title/tt0283054/) (Comedy, Family, Sci-Fi), CP Medien AG, Mission Pictures, Pathé Pictures International, retrieved September 8, 2022
58. *Scooby-Doo (5/10) Movie CLIP - Burping and Farting (2002) HD* (https://www.youtube.com/watch?v=FgYr00Scelc), retrieved September 8, 2022
59. Wudu and Passing Wind (http://www.sunniforum.com/forum/showthread.php?77168-Wudu-and-Passing-Wind). sunniforum.com (2011-09-03) Archived (https://web.archive.org/web/20150515073210/http://www.sunniforum.com/forum/showthread.php?77168-Wudu-and-Passing-Wind) May 15, 2015, at the Wayback Machine
60. Engelhaupt, Erika (September 30, 2015). "People Sometimes Like Stinky Things—Here's Why" (https://www.nationalgeographic.com/science/article/why-do-people-sometimes-like-stinky-things). *Nationalgeographic.com*. National Geographic. Retrieved September 3, 2021.
61. Millard, Elizabeth (November 10, 2017). "Why You Like the Smell Of Your Own Farts" (https://www.menshealth.com/health/a19542002/why-you-like-the-smell-of-your-own-farts/). *www.menshealth.com*. Men's Health. Retrieved September 3, 2021.


## General and cited references

- Allen, V. (2007). *On Farting: Language and Laughter in the Middle Ages*. Palgrave MacMillan. ISBN 978-0-312-23493-5.
- Bolin, T. D. & Stanton, R. (1997). *Wind Breaks*. Allen & Unwin. ISBN 978-1-86448-321-5.
- Dawson, Jim (1999). *Who Cut the Cheese?: A Cultural History of the Fart*. Ten Speed Press. ISBN 1-58008-011-1.
- Dawson, Jim (2006). *Blame it on the Dog: A Modern History of the Fart*. Ten Speed Press. ISBN 1-58008-751-5.
- Franklin, Benjamin (2003). Japikse, Carl (ed.). *Fart Proudly* ((Reprint) ed.). Frog Ltd/Blue Snake. ISBN 1-58394-079-0.
- Persels, J., & Ganim, R. (2004). *Fecal Matters in Early Modern Literature and Art: Studies in Scatology*. (Chap. 1: "The Honorable Art of Farting in Continental Renaissance"). ISBN 0-7546-4116-3.
- von Schmausen, D. (2002). *Official Rules, New World Odor International Freestyle Farting Championship*. LULU. ISBN 1435709195.


# External links

- The Merck Manual of Diagnosis and Therapy, Gas (http://www.merck.com/mmpe/sec02/ch008/ch008d.html)
- Dictionary of Fart Slang (http://www.sillyjokes.co.uk/fart-dictionary)
- Invisible College of Experimental Flatology (https://flatology.com/)

Retrieved from "https://en.wikipedia.org/w/index.php?title=Flatulence&oldid=1125863477"

**This page was last edited on 6 December 2022, at 07:59 (UTC).**

Text is available under the Creative Commons Attribution-ShareAlike License 3.0; additional terms may apply. By using this site, you agree to the Terms of Use and Privacy Policy. Wikipedia® is a registered trademark of the Wikimedia Foundation, Inc., a non-profit organization.
