# Supplementary material for: Assessment of medical information on irritable bowel syndrome information in Wikipedia and Baidu Encyclopedia: comparative study
Source: PeerJ. 2024 May 24;12:e17264. doi: 10.7717/peerj.17264 (PMC11129691; doi:10.7717/peerj.17264)
Supplement: Data S1 [file peerj-12-17264-s001.zip › σÄƒσoïμò░μì«/Wikipedia/Proctalgia fugax - Wikipedia.pdf]

WIKIPEDIA

Proctalgia fugax

Proctalgia fugax, a variant of **levator ani syndrome**, is a severe, episodic pain in the regions of the rectum and anus.[1] It can be caused by cramping of the levator ani muscle, particularly in the pubococcygeal part.[2]

Proctalgia fugax	
**Specialty**	General surgery

Contents

Signs and symptoms

Prevention

Treatment

References

External links

Signs and symptoms

It most often occurs in the middle of the night[3] and lasts from seconds to minutes;[4] pain and aching lasting twenty minutes or longer would likely be diagnosed instead as levator ani syndrome. In a study published in 2007 involving 1809 patients, the attacks occurred in the daytime (33 percent) as well as at night (33 percent) and the average number of attacks was 13. Onset can be in childhood; however, in multiple studies the average age of onset was 45. Many studies showed that women are affected more commonly than men,[5] but this can be at least partly explained by men's reluctance to seek medical advice concerning rectal pain.[6] Data on the number of people affected vary, but prevalence may be as high as 8–18%.[4][7] It is thought that only 17–20% of patients consult a physician, so obtaining accurate data on occurrence presents a challenge.[4]

During an episode, the patient feels spasm-like, sometimes excruciating, pain in the rectum or anus, often misinterpreted as a need to defecate. To be diagnosed as proctalgia fugax, the pain must arise *de novo* (meaning the absence of clear cause). As such, pain associated with constipation (either chronic, or acute), penetrative anal intercourse, trauma (such as tears or fissures of the rectal sphincter or anal canal), side-effects of some medications (particularly opiates), or rectal foreign body insertion preclude this diagnosis. The pain episode subsides by itself as the spasm disappears on its own, but may reoccur.[4]

Because of the high incidence of internal anal sphincter thickening with the disorder, it is thought to be a disorder of that muscle or that it is a neuralgia of pudendal nerves. It is not known to be linked to any disease process.

Prevention

High-voltage pulsed galvanic stimulation (HGVS) has been shown to be of prophylactic benefit, to reduce the incidence of attacks. The patient is usually placed in the left lateral decubitus position and a sterile probe is inserted into the anus. The negative electrode is used and the stimulator is set with a pulse frequency of 80 to 120 cycles per second. The voltage (intensity) is started at 0,

progressively raised to a threshold of patient discomfort, and then is decreased to a level that the patient finds comfortable. As the patient's tolerance increases, the voltage can be gradually increased to 250 to 350 Volts. Each treatment session usually lasts between 15 and 60 minutes. Several studies have reported short-term success rates that ranged from 65 to 91%.[8][9][10][11]

Low dose diazepam of around 2 mg or less, or similar muscle-relaxant, taken orally at bedtime has been suggested as preventative, but its benefits are limited.[12]

Treatment

There is no known cure. The most common approach for mild cases is simply reassurance and topical treatment with calcium-channel blocker (diltiazem, nifedipine) ointment, salbutamol inhalation and topical nitroglycerine. For persistent cases, local anesthetic blocks, clonidine or botulinum toxin injections can be considered.[13][14] Supportive treatments directed at aggravating factors include high-fiber diet, withdrawal of drugs which have gut effects (e.g., drugs that provoke or worsen constipation including narcotics and oral calcium channel blockers; drugs that provoke or worsen diarrhea including quinidine, theophylline, and antibiotics), warm baths, rectal massage, perineal strengthening exercises, anticholinergic agents, non-narcotic analgesics, sedatives or muscle relaxants such as diazepam. In patients who have frequent, severe, prolonged attacks, inhaled salbutamol has been shown in some studies to reduce their duration.[15]

Traditional remedies have ranged from warm baths (if the pain lasts long enough), warm to hot enemas,[16] and relaxation techniques.

References

1. Alberts, Daniel (2012). *Dorland's illustrated medical dictionary* (32nd ed.). Philadelphia, PA: Saunders/Elsevier. p. 1521. ISBN 978-1-4160-6257-8.
2. Olden, Kevin W. (1996). *Handbook of functional gastrointestinal disorders*. New York: M. Dekker. p. 369. ISBN 0-8247-9409-5.
3. Takano M (2005). "Proctalgia fugax: caused by pudendal neuropathy?". *Dis. Colon Rectum*. **48** (1): 114–20. doi:10.1007/s10350-004-0736-3 (https://doi.org/10.1007%2Fs10350-004-0736-3). PMID 15690667 (https://pubmed.ncbi.nlm.nih.gov/15690667). S2CID 792567 (https://api.semanticscholar.org/CorpusID:792567).
4. Whitehead WE, Wald A, Diamant NE, Enck P, Pemberton JH, Rao SS (September 1999). "Functional disorders of the anus and rectum" (https://www.ncbi.nlm.nih.gov/pmc/articles/PMC1766682). *Gut*. **45** (Suppl 2): II55–9. doi:10.1136/gut.45.2008.ii55 (https://doi.org/10.1136%2Fgut.45.2008.ii55). PMC 1766682 (https://www.ncbi.nlm.nih.gov/pmc/articles/PMC1766682). PMID 10457046 (https://pubmed.ncbi.nlm.nih.gov/10457046).
5. de Parades V, Etienney I, Bauer P, Taouk M, Atienza P (2007). "Proctalgia fugax: demographic and clinical characteristics. What every doctor should know from a prospective study of 54 patients". *Dis. Colon Rectum*. **50** (6): 893–8. doi:10.1007/s10350-006-0754-4 (https://doi.org/10.1007%2Fs10350-006-0754-4). PMID 17164968 (https://pubmed.ncbi.nlm.nih.gov/17164968). S2CID 11697730 (https://api.semanticscholar.org/CorpusID:11697730).
6. Brannon, Linda; Feist, Jess (2009-03-19). *Health Psychology: An Introduction to Behavior and Health* (https://books.google.com/books?id=NfCDR_YI7f0C). Cengage Learning. p. 54. ISBN 978-0495601326.

7. Jeyarajah, Santhini; Purkayastha, Sanjay (2013-03-19). "Proctalgia fugax" (https://www.ncbi.nlm.nih.gov/pmc/articles/PMC3602260). *Canadian Medical Association Journal*. **185** (5): 417. doi:10.1503/cmaj.101613 (https://doi.org/10.1503%2Fcmaj.101613). ISSN 1488-2329 (https://www.worldcat.org/issn/1488-2329). PMC 3602260 (https://www.ncbi.nlm.nih.gov/pmc/articles/PMC3602260). PMID 23184844 (https://pubmed.ncbi.nlm.nih.gov/23184844).
8. Sohn N, Weinstein MA, Robbins RD. The levator syndrome and its treatment with high-voltage electrogalvanic stimulation. Am J Surg. 1982;144(5):580-582.
9. Oliver GC, Rubin RJ, Salvati EP, Eisenstat TE. Electrogalvanic stimulation in the treatment of levator syndrome. Dis Colon Rectum. 1985;28(9):662-663.
10. Nicosia JF, Abcarian H. Levator syndrome: A treatment that works. Dis Colon Rectum. 1985;28(6):406-408.
11. Morris L, Newton RA. Use of high voltage pulsed galvanic stimulation for patients with levator ani syndrome. Phys Ther. 1987;67(10):1522-1525
12. Pfenninger JL, Zainea GG (2001). "Common anorectal conditions: Part I. Symptoms and complaints" (http://www.aafp.org/afp/20010615/2391.html). *Am Fam Physician*. **63** (12): 2391—8. PMID 11430454 (https://pubmed.ncbi.nlm.nih.gov/11430454).
13. Jeyarajah S, Chow A, Ziprin P, Tilney H, Purkayastha S (September 2010). "Proctalgia fugax, an evidence-based management pathway". *Int J Colorectal Dis*. **25** (9): 1037—46. doi:10.1007/s00384-010-0984-8 (https://doi.org/10.1007%2Fs00384-010-0984-8). PMID 20556402 (https://pubmed.ncbi.nlm.nih.gov/20556402). S2CID 8635926 (https://api.semanticscholar.org/CorpusID:8635926).
14. Wollina U, Konrad H, Petersen S (2005). "Botulinum toxin in dermatology – beyond wrinkles and sweat". *Journal of Cosmetic Dermatology*. **4** (4): 223—7. doi:10.1111/j.1473-2165.2005.00195.x (https://doi.org/10.1111%2Fj.1473-2165.2005.00195.x). PMID 17168867 (https://pubmed.ncbi.nlm.nih.gov/17168867). S2CID 11333698 (https://api.semanticscholar.org/CorpusID:11333698).
15. Eckardt VF, Dodt O, Kanzler G, Bernhard G (1996). "Treatment of proctalgia fugax with salbutamol inhalation". *Am. J. Gastroenterol*. **91** (4): 686—9. PMID 8677929 (https://pubmed.ncbi.nlm.nih.gov/8677929).
16. Olsen B (2007). "Proctalgia fugax – a nightmare drowned in enema". *Colorectal Disease*. **10** (5): 522—3. doi:10.1111/j.1463-1318.2007.01399.x (https://doi.org/10.1111%2Fj.1463-1318.2007.01399.x). PMID 17949444 (https://pubmed.ncbi.nlm.nih.gov/17949444). S2CID 45958437 (https://api.semanticscholar.org/CorpusID:45958437).

External links

- PatientPlus (http://www.patient.co.uk/showdoc/40024939/)

Retrieved from "https://en.wikipedia.org/w/index.php?title=Proctalgia_fugax&oldid=1097892061"

This page was last edited on 13 July 2022, at 05:31 (UTC).

Text is available under the Creative Commons Attribution-ShareAlike License 3.0; additional terms may apply. By using this site, you agree to the Terms of Use and Privacy Policy. Wikipedia® is a registered trademark of the Wikimedia Foundation, Inc., a non-profit organization.
