# Supplementary material for: Assessment of medical information on irritable bowel syndrome information in Wikipedia and Baidu Encyclopedia: comparative study
Source: PeerJ. 2024 May 24;12:e17264. doi: 10.7717/peerj.17264 (PMC11129691; doi:10.7717/peerj.17264)
Supplement: Data S1 [file peerj-12-17264-s001.zip › σÄƒσoïμò░μì«/IBSΦ»äσêåτ╗ôμ₧£/IBSτû╛τùàσêåτ▒╗.docx]

**The pathophysiology of functional gastrointestinal disorders：**

- **bidirectional dysregulation of gut–brain interaction (via the gut–brain axis)**
- **microbial dysbiosis within the gut**
- **altered mucosal immune function**
- **visceral hypersensitivity**
- **abnormal gastrointestinal motility**

Panel 1: Rome IV adult functional gastrointestinal

disorders (disorders of the gut–brain interaction)14

A: Oesophageal disorders

A1: Functional chest pain

A2: Functional heartburn

A3: Reflux hypersensitivity

A4: Globus

A5: Functional dysphagia

B: Gastroduodenal disorders

B1: Functional dyspepsia

B1a: Postprandial distress syndrome

B1b: Epigastric pain syndrome

B2: Belching disorders

B2a: Excessive supragastric belching

B2b: Excessive gastric belching

B3: Nausea and vomiting disorders

B3a: Chronic nausea vomiting syndrome

B3b: Cyclic vomiting syndrome

B3c: Cannabinoid hyperemesis syndrome

B4: Rumination syndrome

C: Bowel disorders

C1: IBS

C1a: IBS with predominant constipation

C1b: IBS with predominant diarrhoea

C1c: IBS with mixed bowel habits

C1d: IBS unclassified

C2: Functional constipation

C3: Functional diarrhoea

C4: Functional abdominal bloating or distension

C5: Unspecified functional bowel disorder

C6: Opioid-induced constipation

D: Centrally mediated disorders of gastrointestinal pain

D1: Centrally mediated abdominal pain syndrome

D2: Narcotic bowel syndrome or opioid-induced

gastrointestinal hyperalgesia

E: Gallbladder and sphincter of Oddi disorders

E1: Biliary pain

E1a: Functional gallbladder disorder

E1b: Functional biliary sphincter of Oddi disorder

E2: Functional pancreatic sphincter of Oddi disorder

F: Anorectal disorders

F1: Faecal incontinence

F2: Functional anorectal pain

F2a: Levator ani syndrome

F2b: Unspecified functional anorectal pain

F2c: Proctalgia fugax

F3: Functional defaecation disorders

F3a: Inadequate defaecation propulsion

F3b: Dyssynergic defaecation

第 1 组：罗马 IV 成人功能性胃肠道疾病（肠脑相互作用疾病）14

**A：食道疾病**

A1：功能性胸痛

A2：功能性胃灼热

A3：回流过敏

A4：球状体

A5：功能性吞咽困难

**B：胃十二指肠疾病**

B1：功能性消化不良

B1a：餐后不适综合症

B1b：上腹痛综合征

B2：嗳气障碍

B2a：胃上过度嗳气

B2b：过度胃嗳气

B3：恶心和呕吐障碍

B3a：慢性恶心呕吐综合征

B3b：周期性呕吐综合征

B3c：大麻素剧吐综合征

B4：反刍综合症

**C：肠道疾病**

C1：肠易激综合征

C1a：以便秘为主的 IBS

C1b：以腹泻为主的 IBS

C1c：具有混合排便习惯的 IBS

C1d：IBS 未分类

C2：功能性便秘

C3：功能性腹泻

C4：功能性腹胀或腹胀

C5：未明确的功能性肠病

C6：阿片类药物引起的便秘

**D：胃肠道疼痛的中枢介导疾病**

D1：中枢介导的腹痛综合征

D2：麻醉性肠综合征或阿片类药物诱发胃肠道痛觉过敏

**E：Oddi 病症的胆囊和括约肌**

E1：胆道疼痛

E1a：功能性胆囊疾病

E1b：Oddi 病症的功能性胆道括约肌

E2：Oddi 障碍的功能性胰腺括约肌

**F：肛肠疾病**

F1：大便失禁

F2：功能性肛门直肠痛

F2a：肛提肌综合征

F2b：未明确的功能性肛门直肠痛

F2c：一过性直肠痛

F3：功能性排便障碍

F3a：排便推进不足

F3b：失调性排便

第 1 组：罗马 IV 成人功能性胃肠道疾病（肠脑相互作用疾病）14

**B：胃十二指肠疾病**

B1：功能性消化不良

B1a：餐后不适综合症

B1b：上腹痛综合征

B2：嗳气障碍

B2a：胃上过度嗳气

B2b：过度胃嗳气

**C：肠道疾病**

C1：肠易激综合征

C1a：以便秘为主的 IBS

C1b：以腹泻为主的 IBS

C1c：具有混合排便习惯的 IBS

C1d：IBS 未分类

C2：功能性便秘

C3：功能性腹泻

C4：功能性腹胀或腹胀

C6：阿片类药物引起的便秘

**D：胃肠道疼痛的中枢介导疾病**

D1：中枢介导的腹痛综合征

D2：麻醉性肠综合征或阿片类药物诱发胃肠道痛觉过敏

**E：Oddi 病症的胆囊和括约肌**

E1：胆道疼痛

E1a：功能性胆囊疾病

E1b：Oddi 病症的功能性胆道括约肌

E2：Oddi 障碍的功能性胰腺括约肌

**F：肛肠疾病**

F2：功能性肛门直肠痛

F2a：肛提肌综合征

F2b：未明确的功能性肛门直肠痛

F2c：一过性直肠痛

F3：功能性排便障碍

F3a：排便推进不足

F3b：失调性排便
