# Supplementary material for: Assessment of medical information on irritable bowel syndrome information in Wikipedia and Baidu Encyclopedia: comparative study
Source: PeerJ. 2024 May 24;12:e17264. doi: 10.7717/peerj.17264 (PMC11129691; doi:10.7717/peerj.17264)

网页 新闻 贴吧 知道 网盘 图片 视频 地图 文库 百科 百度首页 登录

Baidu百科 大肠激躁症 进入词条 全站搜索 帮助

近期有不法分子冒充百度百科官方人员，以删除词条为由威胁并敲诈相关企业。在此严正声明：百度百科是免费编辑平台，绝不存在收费代编服务，请勿上当受骗！ 详情>>

首页 秒懂百科 特色百科 用户 知识专题 权威合作 下载百科APP 个人中心

收藏 34 1

大肠激躁症

播报 编辑 上传视频

大肠激躁症，Constipation -Irritable Bowel Syndrome,即C-IBS。大肠激躁症简言之是由于肠道之功能异常所表现出来的症候群。其症状可包含腹痛、排便习惯改变、胀气、腹胀、解便不完全的感觉，或粪便中出现黏液。几乎每位成人都曾经历过其中一或数种状况。然而，当这些症状一起出现，持续存在，且具有相当严重之生活困扰时，它们就倾向于被认定为一种功能障碍疾病。

词条统计
浏览次数：41855次
编辑次数：4次历史版本
最近更新：yghm （2012-06-01）
突出贡献榜
crocosmia

1 哈佛大学申请 12 网络工程师
2 电商平台怎么 13 自己创建个
3 37游戏平台 14 网络安全培
4 虚拟货币平台 15 无人机反制
5 csgo电脑配置 16 价格便宜的
6 亚马逊图书 17 sci论文投稿
7 图书批发网 18 vr消防演练
8 购买域名 19 csgo网站开
9 游戏盒子 20 国际期货
10 出版社自费出 21 怎么创建小
11 战队logo设计 22 供应链管理

目录

1 简介
2 病因及致病机转
(1）肠道蠕动功能障碍
(2）饮食因素
(3）神经异常
(4）感觉异常
(5）肠道与大脑之讯息交流异常
3 诊断

简介

播报 编辑

肠道激躁症不会致命，但很少被治愈，且终其一生均可出现，或持续进展。通常患者之解便习惯无一定之准则 - 有时便秘，有时腹泻，有时短期内同时发生这两种状况。大肠激躁症与器质病变之异，在于其症状无法由具体的病灶来解释，甚至也无法明确地以病理生理学阐明。虽然患者之肠道有明显的功能异常，目前之科技尚无法精确衡量，也缺乏适当的诊断工具及相关的检查。因此，临床上多只能由患者描述的症状，来诊断大肠激躁症之存在。

病因及致病机转

播报 编辑

以下为可能为诱发大肠激躁症之原因，但目前尚无定论：

（1）肠道蠕动功能障碍

此学说主张部分患者之肠道蠕动缓慢，部分较快，另有部分患者为快慢交替出现，型态多变。目前这派学说仍有待以肠道蠕动之研究结果证实。

（2）饮食因素

若以特定药物在正常自愿者造成便秘时，他们亦会产生大肠激躁症之症状。而纤维素之减少摄取，也可有类似效果。然而，并无研究证实大肠激躁症患者摄取之纤维素量与正常人有异。因此，纤维素之缺乏，似非明确的致病原因。虽然许多患者认为大肠激躁症之发作与特定食物有关，但这不易以客观方法证实。事实上，摄食本身即可刺激肠道蠕动。总结来说，摄食，特别是一次吃下大量食物，可在大肠激躁症之患者，以一种非特异性之方式刺激症状产生，但目前尚未证实与何种特定食物有关。

（3）神经异常

另一种学说，是症状源于肠道神经控制系统之异常，而非肠道本身。目前许多研究着重于肠间神经丛，也被称为 " 肠道之脑 "。针对肠间神经丛及其与中枢神经系统关联之研究，已开启了一个新的领域：胃肠神经学。学者认为，了解肠间神经丛之生理特性及化学组成，有助于解开大肠激躁症之谜团。肠间神经丛相当复杂，也含有中枢神经系统内所含，大部分的神经传导物质。举例来说，睡眠期肠道的电气活动频率与大脑相同，且患者多有不正常的快速动眼期睡眠。然而，目前尚未证实肠间神经丛为大肠激躁症之主要病因所在。

（4）感觉异常

1980年代以降，感觉异常之理论亦被提出，并引起广泛的注意。许多研究发现，当直肠或乙状结肠中置入气球，开始打气时，大肠激躁症患者会比正常人在较小的打气量或压力下即感到不适或疼痛。类似的状况，也出现在小肠，甚至食道。然而，大肠激躁症患者皮肤感觉疼痛之阈值与正常人无异，甚至更高，因此有 "肠道过度敏感" 之理论。这可解释为何大肠激躁症患者对正常之刺激过度敏感，而产生不正常之反射及肠道运动。但导致肠道过度敏感的原因，又是什么？

以下数种状况，被认为可能造成肠道过度敏感：

- 肠道黏膜受损（如发炎、感染或暴露于有毒化学物质中）

- 肠道肌肉过度伸张

秒懂百科 金灿荣 详解2022年俄乌冲突

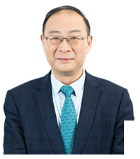

- 脊髓神经之交界处有渗漏现象

- 某种诱发讯号由较高层次之神经中枢经脊髓神经下传

（5）肠道与大脑之讯息交流异常

此理论除了融入前一理论外，亦兼顾心理及生理因素，亦能严重影响症候之形成。

诊断

播报 编辑

临床医师面临之大肠激躁症之诊断挑战，是如何分析患者之症状，并认清大肠激躁症之次型。Manning Criteria 指出了六种于大肠激躁症比结构性腹部疾病更常出现之症状：

I. 解便后腹痛减轻

II. 开始腹痛时，大便变软

III. 开始腹痛时，解便次数较频繁

IV. 腹胀

V. 解出黏液

VI. 感觉大便解不干净

1999年，一群对大肠激躁症有高度研究兴趣之胃肠专家，以Manning Criteria及Rome Criteria为依据，制定了Rome II Criteria，此即成为目前最广为接受及应用之诊断标准：

※ 在过去12个月中，至少有12周（不须连续）发生腹痛或不适，且至少包含以下两种特征：

I. 腹部不适于解便后缓解

II. 解便频率改变

III. 粪便形态改变

※ 在有症状之时日或场合中，至少有四分之一时间发生以下一种或一种以上之症状，而且可用来区分不同分型之大肠激躁症。这些症状并非诊断所必需，但出现愈多，医师对大肠激躁症之诊断就更具说服力：

I. 异常之排便频率（一天多于三次或一周少于三次）

II. 异常之粪便型态（硬块或稀软便/水便）

III. 异常之排便型态（用力、急迫或感到大便解不干净）

IV. 排出黏液

V. 胀气或感到腹胀

附注：患者必须无造成这些症状之结构性或代谢疾病症状出现之十二周不需有连续性

值得注意的是，若患者出现以下现象，则为器质性疾病之警讯，须一步安排适当之检查：

I. 血便或由肛门排血

II. 体重减轻

III. 持续腹泻

IV. 最近才出现，且持续未缓解之腹胀

V. 贫血

VI. 发烧

举例来说，肠炎，特别是糜烂性肠炎，可符合 Manning Criteria 中之五项标准。然而，其血便形态、发烧、体重减轻、贫血，可作为鉴别。

搜索发现

激躁性大肠要吃药吗 大肠激躁症如何治疗 大肠激躁症能喝酸奶吗 大肠激躁症吃什么药 大肠激躁症可[illegible]
激躁性大肠症放屁多 激躁性大肠症怎么治愈 大肠激躁症会背痛 部队文职是什么工作 免费兼职

秒懂百科
金灿荣
详解2022年俄乌冲突
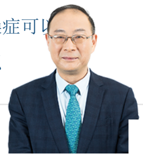

新手上路
成长任务 编辑入门
编辑规则 本人编辑 NEW

我有疑问
内容质疑 在线客服
官方贴吧 意见反馈

投诉建议
举报不良信息 未通过词条申诉
投诉侵权信息 封禁查询与解封

©2022 Baidu 使用百度前必读 | 百科协议 | 隐私政策 | 百度百科合作平台 | 京ICP证030173号
京公网安备11000002000001号

秒懂百科
金灿荣
详解2022年俄乌冲突

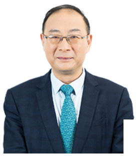

Supplement: Data S1 [file peerj-12-17264-s001.zip › σÄƒσoïμò░μì«/Baidu/Baidu-Chinese/6-σñoΦéáμ┐ÇΦ║üτùç_τÖ╛σ║aτÖ╛τoæ.pdf]
