# Supplementary material for: Assessment of medical information on irritable bowel syndrome information in Wikipedia and Baidu Encyclopedia: comparative study
Source: PeerJ. 2024 May 24;12:e17264. doi: 10.7717/peerj.17264 (PMC11129691; doi:10.7717/peerj.17264)

近期有不法分子冒充百度百科官方人员，以删除词条为由威胁并敲诈相关企业。在此严正声明：百度百科是免费编辑平台，绝不存在收费代编服务，请勿上当受骗！ 详情>>

肠易激综合征是一个多义词，请在下列义项上选择浏览（共3个义项） | 添加义项 +

- 肠易激综合征
- **2005年中国医药科技出版社出版的图书**
- 2015年中国中医药出版社出版的图书

收藏 | 0 | 0

肠易激综合征

播报 编辑 讨论 1 上传视频

2005年中国医药科技出版社出版的图书

《肠易激综合征》是中国医药科技出版社出版的图书，作者是李延青，杨云生，陈建 [1]

**书　名**	肠易激综合征 中国医药临床医学专著系列	**出版社**	中国医药科技出版社
**作　者**	李延青、杨云生、陈建	**定　价**	69.38 元
		**ISBN**	9787506731010

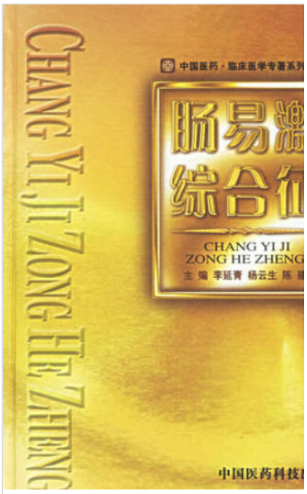

肠易激综合征的概述图（1）

正版 肠易激综合征 李延青,杨云生,陈建 主...
¥43.00
服务由京东提供 购买

正品！肠易激综合征
¥51.00
服务由京东提供 购买

词条图册

更多图册 >

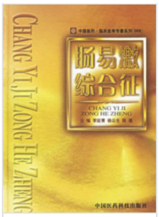
概述图册 (1)

词条统计

浏览次数：316次
编辑次数：2次历史版本
最近更新：一如既往11024 （2

1 亚马逊图书
2 csgo网站开箱
3 图书管理系统
4 出版社自费出
5 自己创建个网
6 战队logo设计
7 游戏盒子
8 购买域名
9 37游戏平台
10 sci论文投稿
11 csgo电脑配置
12 国际期货
13 图书批发网
14 哈佛大学申
15 vr消防演练
16 虚拟货币平
17 无人机反制
18 电商平台怎
19 网络安全培
20 网络工程师
21 怎么创建小
22 价格便宜的

参考资料

1 肠易激综合征 . 京东[引用日期2021-06-22]

猜你喜欢

幽门螺旋杆菌，吃益生菌有用吗?高活性益生菌
幽门螺旋杆菌哪些症状，感染幽门螺旋杆菌怎么办，幽门螺杆菌是胃部发炎引起的怎么治幽门螺旋杆菌，恶心干呕，易导致口臭，...
item.jd.com

秒懂百科
金灿荣
详解2022年俄乌冲突

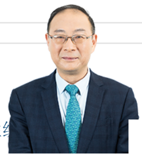

搜索发现

治疗肠易激的中成药 肝郁脾虚最好中成药 中医治疗肠易激综合征 拉肚子拉水止泻小妙招 肠应激性

肠激惹综合症吃什么药 肠易激综合征的症状特点 肠易激综合征病因 肠易激综合症怎么调理 肠易激综合症自愈方法

新手上路
成长任务 编辑入门
编辑规则 本人编辑 NEW

我有疑问
内容质疑 在线客服
官方贴吧 意见反馈

投诉建议
举报不良信息 未通过词条申诉
投诉侵权信息 封禁查询与解封

©2022 Baidu 使用百度前必读 | 百科协议 | 隐私政策 | 百度百科合作平台 | 京ICP证030173号
京公网安备11000002000001号

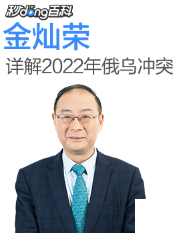

Supplement: Data S1 [file peerj-12-17264-s001.zip › σÄƒσoïμò░μì«/Baidu/Baidu-Chinese/3-Φéáμÿôμ┐Çτ╗╝σÉêσ╛ü∩╝ê2005σ╣┤Σ╕¡σ¢╜σî╗Φì»τoæμèÇσç║τëêτñ╛σç║τëêτÜäσ¢╛Σ╣a∩╝ë_τÖ╛σ║aτÖ╛τoæ.pdf]
