# Supplementary material for: Assessment of medical information on irritable bowel syndrome information in Wikipedia and Baidu Encyclopedia: comparative study
Source: PeerJ. 2024 May 24;12:e17264. doi: 10.7717/peerj.17264 (PMC11129691; doi:10.7717/peerj.17264)

网页 新闻 贴吧 知道 网盘 图片 视频 地图 文库 百科 | 百度首页 登录

Baidu百科 功能性腹胀 进入词条 全站搜索 帮助

近期有不法分子冒充百度百科官方人员，以删除词条为由威胁并敲诈相关企业。在此严正声明：百度百科是免费编辑平台，绝不存在收费代编服务，请勿上当受骗！ 详情>>

首页 秒懂百科 特色百科 用户 知识专题 权威合作 下载百科APP | 个人中心

收藏 0 1

功能性腹胀

播报 编辑 上传视频

本词条由“科普中国”科学百科词条编写与应用工作项目 认证 。

功能性腹胀（functionl bloating）是一种反复出现腹部膨胀的主观感觉，不同于进食后出现的饱胀不适感，伴有或不伴有可测量的腹围增加，它不属于肠易激综合征等其他功能性肠病或功能性消化不良等功能性胃十二指肠病的一部分。10%～30%的普通人群可受到腹胀的影响，女性常见，与年龄无关。一般认为，功能性腹胀与肥胖、吞气症、贪饮多食、膈肌下降、脊柱前突、腹部肌力减弱、特别是精神状态有关，它是一个间歇的慢性过程。

**就诊科室**	消化内科	**常见病因**	不明，可能与生理、心理因素有关
**多发群体**	女性	**常见症状**	腹胀，白天逐渐加重，尤其进食后 展开
**常见发病**	腹部	**作者**	

科普中国

致力于权威的科学传播

本词条认证专家为

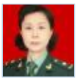

韩英 | 主任医师

北京军区总医院 消化内科

审核

目录

1 病因

2 临床表现

3 诊断

4 鉴别诊断

5 治疗

权威合作编辑

“科普中国”科学百科词条编写与应用工作项目

“科普中国”是为我国科普信息化建设塑造的全...

什么是权威编辑

词条统计

浏览次数：20067次

编辑次数：2次历史版本

最近更新：卫计委科普项目 （2017-11-2

突出贡献榜

卫计委科普项目

基本信息

**就诊科室**	消化内科	**常见病因**	不明，可能与生理、心理因素有关
**多发群体**	女性	**常见症状**	腹胀，白天逐渐加重，尤其进食后，晚上减轻；伴上腹部疼痛、早饱和食物胃内滞留
**常见发病部位**	腹部	**作　者**	

病因

播报 编辑

目前，功能性腹胀的病理生理机制尚未完全阐明，主要与生理因素、精神心理因素有关。①生理因素包括肠道气体堆积、感觉动力功能异常、食物不耐受、液体潴留、腹壁肌肉力量薄弱等；②精神心理因素包括抑郁、失眠、应对障碍、惊恐障碍、恐惧症等。

临床表现

播报 编辑

典型的症状是腹胀，白天腹胀症状逐渐加重，尤其是在进食后，晚上减轻。同时伴有上腹部疼痛、早饱和食物在胃内滞留的聚集现象。

秒懂百科
金灿荣
详解2022年俄乌冲突

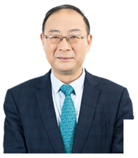

诊断

播报 编辑

功能性腹胀的罗马Ⅲ诊断标准如下：①3个月内每月至少有3天反复出现腹胀感或肉眼可见的腹部膨胀；②没有足够的证据诊断功能性消化不良、肠易激综合征或其他功能性胃肠疾病。诊断前症状出现至少6个月，近3个月满足以上标准。

鉴别诊断

播报 编辑

1.吞气症

患者常有精神紧张、情绪不稳定或抑郁的表现，消化道主要症状是嗳气或呃逆，患者自感嗳气后舒服，实际上是在嗳气的同时吞咽了大量的空气，使上腹部有膨胀或饱胀感。X线钡餐或胃镜检查均无明显的器质性病变。

2.慢性萎缩性胃炎

多见于中年以上患者，主要症状为上腹隐痛、腹胀、食欲减退及消瘦、贫血等症状。胃镜及黏膜活检组织病理检查可确立诊断。

3.胃下垂

好发于瘦长、无力体型者，腹壁松弛的老年人与经产妇或慢性消耗性疾病者。腹胀一般于清晨起床时较轻，站立过久至下午、晚上时症状加重，还可伴有食欲减退、恶心、嗳气、四肢无力等症状。X线钡餐检查可见胃的位置明显下移，胃轮廓下移至两侧髂脊连线以下，胃呈无力型，有利于胃下垂的诊断。

治疗

播报 编辑

本病目前尚无有效的防治措施。

1.一般治疗

对患者进行健康宣传教育，提醒患者注意规律运动、减轻体重等。

2.饮食调整

避免摄入产气的食物，如高糖食物、豆类或牛奶等。若食用日常食品、新鲜水果或果汁后症状加重，提示乳糖或果糖不耐受，需要进一步检查或进行饮食排除试验。

3.药物治疗

（1）益生菌：①调整微生态失调，防治腹泻；②缓解乳糖不耐受症状，促进机体营养吸收；③代谢产物可产生生物拮抗，抵抗细菌病毒的感染，增强人体免疫力，改善肠道的屏障功能，缓解过敏作用；④预防和治疗某些疾病，如肠道综合征、呼吸道感染、过敏、口臭、胃溃疡等。

（2）胃肠促动力剂：胃肠促动力剂对部分患者可能有一定效果。

（3）其他药物：如胰酶制剂、活性炭（药用炭）、表面活性物质等可能有效。

学术论文

内容来自 Baidu学术

. 年

许卫华，王微，李妮娇，吕冉等. 加味三香汤治疗脾虚痰湿型功能性腹胀的临床观察. 《中华中医药杂志》，2014

张红英，王进海，李永等. 功能性腹胀发病机制的研究. 《CNKI;WanFang》，2013

王峰，李小芳，周晓军. 大柴胡汤加减治疗功能性腹胀的效果. 《实用临床医药杂志》，2015

贾志新，冯五金等. 冯五金老中医温阳通调法治疗功能性腹胀经验体会. 《世界中西医结合杂志》，2015

查看全部 >

搜索发现

肝病会引起腹胀吗 肚子老是胀气怎么办 英语该如何学习 篆体字图片识别 怎么炒饭
二手包回收 青岛搬家 米粉图片 酒柜样式

新手上路
成长任务 编辑入门
编辑规则 本人编辑 NEW

我有疑问
内容质疑 在线客服
官方贴吧 意见反馈

投诉建议
举报不良信息 未通过词条申诉
投诉侵权信息 封禁查询与解封

©2022 Baidu 使用百度前必读 | 百科协议 | 隐私政策 | 百度百科合作平台 | 京ICP证030173号
京公网安备11000002000001号

秒懂百科
金灿荣
详解2022年俄乌冲突

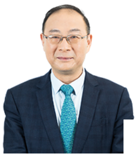

Supplement: Data S1 [file peerj-12-17264-s001.zip › σÄƒσoïμò░μì«/Baidu/Baidu-Chinese/11-σèƒΦâ╜μÇoΦà╣ΦâÇ_τÖ╛σ║aτÖ╛τoæ.pdf]
