# Supplementary material for: Assessment of medical information on irritable bowel syndrome information in Wikipedia and Baidu Encyclopedia: comparative study
Source: PeerJ. 2024 May 24;12:e17264. doi: 10.7717/peerj.17264 (PMC11129691; doi:10.7717/peerj.17264)

近期有不法分子冒充百度百科官方人员，以删除词条为由威胁并敲诈相关企业。在此严正声明：百度百科是免费编辑平台，绝不存在收费代编服务，请勿上当受骗！ 详情>>

易激结肠

播报 编辑 讨论 上传视频

医学疾病

本词条缺少**概述图**，补充相关内容使词条更完整，还能快速升级，赶紧来编辑吧！

易激结肠（irritable bowel syndrome，IBS）为一种与胃肠功能改变有关，以慢性或复发性腹痛、腹泻、排便习惯和大便性状异常为主要症状而又缺乏胃肠道结构或生化异常的综合征。

中文名	易激结肠	外文名	irritable bowel syndrome
		ICD号	K63.8

词条统计

浏览次数：5609次

编辑次数：8次历史版本

最近更新：杨家将1206 （2022-06-05）

突出贡献榜

hnta933

1 csgo电脑配置
2 电商平台怎么
3 网络安全培训
4 无人机反制
5 自己创建个网
6 csgo网站开箱
7 亚马逊图书
8 图书批发网
9 出版社自费出
10 国际期货
11 游戏盒子
12 购买域名
13 sci论文投稿
14 37游戏平台
15 价格便宜的
16 vr消防演练
17 哈佛大学申
18 网络工程师
19 战队logo设
20 虚拟货币平
21 怎么创建小
22 供应链管理

目录

1 基本信息
2 疾病名称
3 英文名称
4 别名
5 分类
6 ICD号
7 流行病学
8 病因
- 精神、神经因素
- 肠道刺激因素
9 发病机制
- 肠运动异常
- 感觉异常
- 分泌异常
10 易激结肠的临床表现
- 症状
- 体征
11 易激结肠的并发症
12 实验室检查
13 辅助检查
- X线钡灌肠检查
- 乙状结肠镜或纤维结肠镜检查
- 肠道动力检查
14 诊断
15 鉴别诊断
- 慢性细菌感染
- 慢性阿米巴痢疾
- 血吸虫感染
- 吸收不良综合征
- 肠肿瘤
- 溃疡性结肠炎
- 克罗恩病
- 乳糖酶缺乏
- 胃肠道内分泌肿瘤
- 甲状腺疾病
16 易激结肠的治疗
- 治疗原则
- 生活和饮食调节
- 精神治疗
- 药物治疗
17 预后
18 易激结肠的预防
19 相关药品
20 相关检查

基本信息

播报 编辑

常与胃肠道其他功能性疾病如胃食管反流性疾病（GERD）和功能性消化不良（FD）同时存在。需要经过检查排除可引起这些症状的器质性疾病.本病属于胃肠功能紊乱性疾病，包括腹痛、腹胀、排便习惯和大便性状异常、粘液便，持续存在或间歇发作，而又缺乏形态学和生化异常改变可资解释的症候群。其特征是肠道功能的易激性。

疾病名称

播报 编辑

易激结肠

英文名称

播报 编辑

irritable bowel syndrome

别名

播报 编辑

肠易激综合症；肠应激综合征；过敏性肠综合征；过敏性大肠综合征；过敏性结肠炎；结肠痉挛；黏液性结肠炎；肠易激综合征；应激性结肠综合征

分类

播报 编辑

消化科 > 肠道疾病 > 大肠疾病

ICD号

播报 编辑

K63.8

秒懂百科 金灿荣 详解2022年俄乌冲突

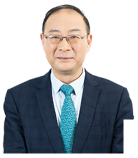

流行病学

播报 编辑

IBS是最常见的消化系统疾病之一。据西方统计，IBS约占成年人群的14%～22%，女性为男性的1.38倍，其中只有50%的IBS患者就医。另一些资料显示欧美人群的患病率约为7.1%～13.6%。我国1994年南、北方9省市成人人群调查（非随机）发现每年有6次以上腹痛、便后缓解的发生率22.1%。据北京协和医院报道，1996年北京市城乡18～70岁的普通人群调查结果，具有IBS症状符合Manning标准的人群患病率为7.01%，以18～30岁年轻人较多见，其中就医者仅占20%。

病因

播报 编辑

IBS病因尚不明确。目前认为与以下因素有关。

精神、神经因素

IBS患者精神心理异常的出现率明显高于普通人。有研究表明，精神紧张可以改变肠道的mmc，精神刺激对IBS病人比正常人更易引起肠动力紊乱。现代神经生理学认为IBS患者的肠道对于张力和多种刺激的敏感性增加。但这究竟是由于肠壁神经丛及其感受器或传入神经通路上的异常，还是中枢神经系统对肠道的调节异常目前还不明确。另外有研究发现应激可引起大鼠功能性结肠动力紊乱，同时发现应激后一些胃肠道激素的释放增加，说明神经内分泌的调节参与应激所引起的肠功能紊乱反应过程。以上精神、神经因素与IBS的关系，支持目前认为IBS是属于身心疾病类胃肠病的观点。

肠道刺激因素

肠道内某些因素可能改变肠功能，加重原有的易激结肠。这些刺激因素包括外部的食物、药物、微生物等，也可能包括消化过程中所产生的某些内部物质。有实验发现腔内抗原激发致敏的鼠肠道可明显诱导鼠肠道收缩活动和产生腹泻。有分析认为，当某些刺激物多次作用于肠道时，可能改变肠道的感觉运动功能以及对于刺激的敏感性，从而使肠管产生"易激性"。有报道IBS病人回肠对灌注胆汁酸的分泌作用非常敏感，但可能未被诊断为胆汁酸吸收不良。短链或中链脂肪酸在吸收容量受限或在小肠内快速运转等的病人中可能到达右半结肠，引起右半结肠出现快速通过的高压力波，这些波极有效地推进结肠内容物并可能引起疼痛和腹泻。这些肠道刺激物在IBS中是诱因还是病因目前尚未定论。

发病机制

播报 编辑

肠运动异常

IBS的主要发病机制是肠运动功能异常。有研究发现IBS患者空肠段丛集状收缩波（discreted clustered constrictions，DCCs）及回肠推进性收缩波（prolonged propagated constrictions，PPCs）增多，且与痉挛性疼痛一致。腹泻型IBS患者白天的移行性运动复合波（migrating motor complex，MMc）出现次数增多，周期缩短；在Ⅱ期和进餐后有较多的空肠收缩；结肠显示大量的快速收缩和推进性收缩；近段结肠快速通过且与大便的重量呈正相关；胆碱能刺激后降-乙状结肠多项动力指标增加。相反，便秘型IBS患者近段结肠通过时间延长，排空明显减慢；高幅推进性收缩减少；降、乙状结肠在基础状态下的收缩频率和收缩时间减少，对胆碱能刺激的反应性降低，与此同时近端结肠收缩时间的百分比却显著增加，表现为不协调性。肛管内压力升高，肛门括约肌对直肠扩张的反应性松弛迟钝。排便时外括约肌异常收缩，与IBS患者排便困难有关。

IBS动力异常不仅限于肠道，食管、胃、胆道均存在动力紊乱，以致有"胃肠道哮喘（asthma of gut）"之称。目前有关IBS动力的研究结果尚不完全一致，有些甚至得出相反的结果。说明IBS的动力紊乱是很复杂的，它不单是某一肠段动力发生某种异常，而存在着相互间的协调问题。

感觉异常

IBS患者的腹痛阈值较正常人低，因此对标准的结肠扩张产生过度的感觉。这种感觉异常与丛集运动异常的协同作用是IBS患者出现痉挛性疼痛的主要因素。精神压力、焦虑加重患者结肠扩张时的疼痛感觉。相反，松弛状态下对肠扩张知觉减低。超常的直肠肛门感觉引起排便不尽感，甚至排便前的腹痛感。而直肠肛门的过度感觉又与直肠过度的反射运动相伴随。即排便不尽感刺激引起运动应答增强，从而导致排便频度增加，但不伴排便重量增加。

分泌异常

IBS患者小肠黏膜对刺激性物质的分泌反应增强。结肠黏膜分泌黏液增多。

易激结肠的临床表现

播报 编辑

症状

（1）腹痛、腹部不适：常沿肠管有不适感或腹痛，可发展为绞痛，持续数分钟至数小时，在排气排便后缓解。有些食物如粗纤维蔬菜、粗质水果、浓烈调味品、酒、冷饮等，可诱发腹痛。但腹痛不进行性加重。睡眠时不发作。

（2）腹泻或不成形便：常于餐后，尤其是早餐后多次排便。亦可发生于其余时间，但不发生在夜间。偶尔大便最多可达10次以上。但每次大便量少，总量很少超过正常范围。有时大便仅1～2次，但不成形。腹泻或不成形便有时与正常便或便秘相交替。

（3）便秘：每周排便1～2次，偶尔10余天1次。早期多间断性，后期可持续性而需服用泻药。

（4）排便过程异常：患者常出现排便困难，排便不尽感或便急等症状。

（5）黏液便：大便常带有少量黏液。但偶有大量黏液或黏液管型排出。

秒懂百科
金灿荣
详解2022年俄乌冲突
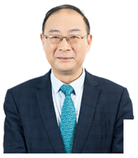

（6）腹胀：白天明显、夜间睡眠后减轻，一般腹围不增大。

体征

盲肠和乙状结肠常可触及，盲肠多呈充气肠管样感觉；乙状结肠常呈索条样痉挛肠管或触及粪块。所触肠管可有轻度压痛，但压痛不固定，持续压迫时疼痛消失。部分病人肛门指诊有痛感，且有括约肌张力增高的感觉。

易激结肠的并发症

播报 编辑

症状出现或加重常与精神因素或一些应激状态有关。部分病人伴有上胃肠道及肠道外多种功能紊乱的症状。还可伴有心理精神异常表现，如抑郁、多疑、紧张、焦虑、敌意等。

实验室检查

播报 编辑

粪便呈水样便，软便或硬块，可有黏液。无其他异常。

辅助检查

播报 编辑

X线钡灌肠检查

常无异常发现。少数病例因肠管痉挛出现“线征”。其他非特异性的表现可有结肠袋加深或增多等。

乙状结肠镜或纤维结肠镜检查

肉眼观察黏膜无异常，活检也无异常。但在插镜时可引起痉挛、疼痛，或在充气时引起疼痛。如疑有脾区综合征，可在检查时慢慢注入100～200ml气体，然后迅速将镜拔出，嘱病人坐起，在5～10min后即可出现左上腹痛，向左肩放射，这可作为脾区综合征的客观指征。

有的医生在直肠中放入气囊，打气后病人出现疼痛。过敏结肠病人出现腹痛时，气囊的压力比正常人明显低。

肠道动力检查

与食管和胃不尽相同且不完善。

（1）肠道通过时间检查：

①氢呼吸试验法：其原理为不能在小肠吸收的糖类如乳果糖在结肠内经细菌酵解释放氢气，经肺呼出。因而口服乳果糖后，相隔一定时间（10～15min）收集呼气氢，利用气敏色谱仪测定呼气氢浓度。根据呼出气体氢气浓度的变化，测算口-盲通过时间。当呼吸氢浓度高出基础值的50%或高出4～l0ppm水平时，即为峰值，从口服乳果糖至到达峰值的时间为口-盲通过时间。有些因素影响检查结果，如空腹时服乳果糖，对于不同的病人，当时所处的消化间期时相不同，造成口-盲通过时间结果不均一性，因此应与试餐同时服乳果糖，因进餐后消化间期立刻被停止，而代之以消化期的活动。这样受试者条件一致。若与液体试餐同给则代表液体的通过时间，若与固体同给则表示固体的通过时间。试餐的成分需与普通膳食的成分相似的规定膳食；运动量影响动力，需规定同样的运动量；药物会影响呼吸试验，要求检查前48h停服抗胆碱药、钙离子通道阻滞药，硝酸甘油、镇静药及精神药物，1个月内未用抗生素；另外还应考虑胃排空功能和消化道细菌的影响。

②放射性核素扫描法：

A.小肠通过时间测定：通常用99mTC标记试餐，试餐后在γ相机下进行计数（前后体位的核素扫描，以纠正误差）。若用两种放射性核素标记，其中一种用于胃排空，一种用于测定口-盲通过时间，由此推算出小肠通过时间。

B.结肠通过时间的测定：以放射性核素标记液态物灌入盲肠或口服定位在盲肠崩解的放射性核素胶囊。从而测算结肠的充盈和各段的通过时间，其缺点是非生理性标志物。相比之下，口服胶囊法更接近生理情况。

③不透X线标志物法：口服一种或一种以上（需间隔一定时间）不透X线标志物后定期摄片，根据腹平片上标志物的分布，利用结肠含气，同时利用腹平片上的骨性标志，以及连续摄片的标志物移动方向，对平片上的标志物做出位置的判断，测算全胃肠通过时间、口-盲通过时间、全结肠及各段结肠通过时间。

（2）压力测定：

①小肠压力测定：将压力集合管或带有微型压力传感器的导管经胃插入十二指肠直至空肠上段（X线下需导丝导引，可通过内镜插入），可测得小肠消化间期和消化期的动力活动（收缩次数、收缩幅度和动力指数）。影响因素较多，导管内气泡影响测压准确性；肠道收缩时肠腔不消失将影响记录的准确性；多种药物影响准确性，插管和测定过程中，技术和配合也是重要的影响因素。标化仪器十分重要，否则引起测定误差。

②结肠压力测定：经结肠镜活检孔送入导丝，再将压力导管套入导丝，也在X线导引下，将其送至结肠，记录空腹及餐后或给药后动力活动情况。影响因素如下：空腹与进餐活动不一致；药物影响明显；方法不同结果差异很大；插入技术要求轻巧迅速，否则影响结果。

诊断

播报 编辑

金灿荣
详解2022年俄乌冲突

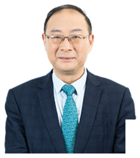

诊断有肠道功能性疾病的症状，在排除各种可能的器质性病变后，可诊断为肠功能性疾病。易激结肠症状诊断标准不统一，并不断修改。目前国际普遍采用的为1992年罗马标准：

1.症状持续存在或反复发生超过3个月。

2.必须具备以下症状

（1）腹痛或腹部不适，并具有下述特征：排便后缓解；和（或）伴有大便性状改变。

（2）排便不正常至少发生于25%时间，至少具有以下2种：大便频率改变（>3次/d或者<3次/周）；大便性状改变（硬：团块（或）稀：水便）；排便过程改变（排便费力或便急或排便不尽感）；排黏液，伴随有肠胀气或腹胀感。

根据其主要表现形式，易激结肠可分为不同类型，一般分为腹泻型（diarrhea-predominant，IBS-D）和便秘型（constipation-predominant，IBS-C）二大类，还有各种混合型。

鉴别诊断

播报 编辑

慢性细菌感染

多次粪便常规及培养有阳性发现，以及充分有效地抗生素系统性治疗，症状改善明显，可明确诊断。

慢性阿米巴痢疾

多次大便找阿米巴及甲硝唑试验治疗可明确诊断。

血吸虫感染

血吸虫疫区病人可作乙状镜检查，取直肠黏膜找血吸虫卵，或用粪便孵化法和其他方法加以鉴别。

吸收不良综合征

有腹泻，但大便中常有脂肪和未消化食物。

肠肿瘤

小肠的良性小肿瘤可发生腹泻和间歇性发作的部分肠梗阻。结肠肿瘤也可以出现类似肠道功能性疾病的症状。特别是对老年人应注意。可进行X线钡剂造影检查或结肠镜检查以明确诊断。

溃疡性结肠炎

有发热、脓血便等异常表现。经X线钡剂造影或结肠镜检查可以鉴别。

克罗恩病

常有发热、贫血、虚弱等全身症状。X线钡剂造影或结肠镜检查即可鉴别。

乳糖酶缺乏

乳糖耐量试验可以鉴别。乳糖酶缺乏有先天和后天之分。临床表现为吃乳制品后有严重的腹泻，大便含有大量泡沫和乳糖、乳酸。食物中去掉牛奶或奶制品，症状即可改善。酸牛奶经乳酸菌将乳糖分解，可供这类病人食用。

胃肠道内分泌肿瘤

促胃液素瘤可出现严重的腹泻和顽固的溃疡病，血清促胃液素水平极高，一般治疗无效。血管活性肠肽瘤（Vipoma）也引起严重腹泻；血清VIP水平增高。

甲状腺疾病

甲状腺功能亢进可出现腹泻。甲状旁腺功能亢进可出现便秘。可作甲状腺、甲状旁腺功能检查以进行鉴别。

易激结肠的治疗

播报 编辑

治疗原则

IBS病因复杂，症状较多且易反复，不能单纯依靠特定的药物治疗，需按不同个体采用综合性的全身性治疗。

生活和饮食调节

避免诱发因素，饮食选用易消化、少脂肪，禁食刺激性、敏感性食品。对便秘、腹胀者，可适当多吃些富含纤维素，但不易产气的饮食，避免过食及零食。以腹泻为主的患者，应少吃含粗纤维的食品。

精神治疗

精神状态与肠道症状密切相关。医务工作者要以同情和负责的态度向病人解释疾病的性质和注意事项，应解除病人许多疑虑的心态，使其消除恐惧，提高战胜疾病的信心。必要时应用镇静、抗抑郁治疗。可用去郁敏50mg， 3次/d，或盐酸氟西丁（百忧解，Prozae）20mg/d，或用氟哌噻吨/二甲胺丙/二甲胺丙烯（黛安神），每天上午2片，口服，以缓解其精神异常，使腹痛等不适得以缓解。亦可选用阿米替林25mg，2次/d、多塞平25mg，2～3次/d，睡眠差者服地西泮（安定）等。

金灿荣
详解2022年俄乌冲突
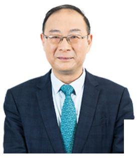

药物治疗

虽可减轻症状，但不能预防复发，故应合理用药，并避免滥用药。

①抗胆碱能药：如山莨菪碱（654-2）、贝那替秦（胃复康）等，因不良反应较多渐被其他药物替代。目前推荐应用美贝维林（麦皮凡林）10mg；双环维林10mg；吡芬嗅铵（prifiniumbromide） 30mg；口服3次/d。亦有应用西托溴铵（cimetropium bromide）50mg餐前服，取得良好效果。

②盐酸醋丁洛尔（盐酸醋丁酰心胺）：具有单一抗运动，无麻醉的抗胆碱能作用，用量为2mg，3～4次/d。亦可用可乐定（氯压定）0.3～0.4mg，3次/d，口服，能促进小肠对液体物质吸收，增强结肠对电解质的吸收，以减慢小肠的转运时间，有较好的止泻效果。

③钙通道阻滞药：硝苯地平（心痛定）10～20mg或维拉帕米（异搏定）40mg，3次/d口服，可抑制胃结肠反射，缓解腹痛，减少便次。

④阿片类止泻剂：洛哌丁胺（易蒙停）作用于肠壁的阿片受体，阻滞乙酰胆碱和前列腺素释放，抑制肠蠕动，增加水、电解质吸收。每次口服2mg，2～3次/d，每天用量不得超过10mg。便成形后可渐减用量至停药。有患者出现口干、腹胀，甚至呈假性肠梗阻等副作用。亦有服复方苯乙哌啶，本药系哌替丁衍生物，除有止泻效果外，尚有兴奋中枢神经作用，大剂量有止痛和欣快感，长期使用有依赖性。

⑤微生态调节剂：是通过微生物学技术，将人体内正常菌群分离出来，经纯培养后，进行工业化生产，制成益生菌制品，再按原途径回归人体，调整微生态失常，达到防治疾病增强免疫功能作用。微生态调节剂除有益生菌制品（Probiotics）外，尚有益生菌生长促进物质称益生元（Prebiotcs）。益生菌制剂服后进入肠道，迅速定植于肠道黏膜，并迅速繁殖形成生物学屏障，分解葡萄糖产生乳酸，使肠道pH降低，抑制致病菌的繁殖生长，纠正肠道菌群失调，恢复和维持肠内微生物生态系统稳定，改变肠道运动功能。常益生菌制剂有：

A. 口服双歧杆菌三联活菌制三联活菌制剂（培菲康）：为双歧杆菌、嗜酸乳杆菌、粪链球菌等组成，对抗生素、化疗药物具有抵抗性的乳酸菌制剂，使患者服用抗生素、化疗药后，肠内菌群保持平衡，消除由菌群失调引起的一些症状。每次服2～4粒，2～3次/d。

B. 嗜酸乳杆菌（乐托尔）：主含嗜酸乳杆菌及其代谢产物，具有抑制肠道致病菌的生长，阻止细菌、病毒与肠绒毛黏附作用。每次1粒，口服3～4次。

C.聚克通：主要含嗜酸乳杆菌、乳酸乳杆菌、乳链球菌3种乳酸菌，对多种抗生素有抵抗性。每次2粒，3次/d。

D.佳士康（gastrferm）主要为活性粪肠球菌，能耐受多种抗生素。每次1～2粒，2～3次/d。

E. 酪酸梭状芽孢肝菌（米雅BBM）：为宫入菌（酪酸菌）是芽孢厌氧梭状杆菌，在体内不受胃酸、胆汁等影响，是一体内正常菌群，可阻止有害菌定植，纠正肠内菌群紊乱，每服2片（含宫入菌末40mg），3次/d。此外，尚有双歧杆菌（丽珠肠乐）、地衣芽孢杆菌（整肠生）、双歧杆菌（回春生）、乳酸菌素、乳酶生等，均属此类药物。

①饮食调整：进食有软化和扩大粪便容积的食物，如粗纤维多的食物，适量多饮水，定时排便。

②西沙必利（普瑞博思）：通过兴奋节前神经元的5-羟色胺4（5-HT4）受体，并作用于肠肌丛神经节细胞使乙酰胆碱释放，增加胃肠推动力，促进排便。每次5～10mg， 3次/d餐前半小时服。

③乳果糖：口服15ml/d，可增加便次，使粪便变软，缓解排便困难。但有过敏者应慎用。

④口服甘露醇2～4g，3次/d；或服葡甘聚糖1g，3次/d。

注意情绪与腹痛的关系，必要时暗示疗法或局部热敷、理疗、按摩或封闭。近年报道钙通道阻滞药有良好作用，对胃肠平滑肌有松弛作用，尤其对食管及结肠作用为佳，腹痛者可试用硝苯地平（心痛定），及解痉镇痛药等。选择性胃肠道钙通道抑制剂能解除胃肠道平滑肌痉挛，抑制餐后结肠运动反应。对IBS患者腹痛治疗效果较好，对腹泻和便秘亦有一定疗效。常用选择性钙通道拮抗药有匹维溴胺（得舒特）5mg/次，3次/d；双环维林（双环胺） 10～20mg/次，3～4次/d；西托溴铵（cimetropium bromide）50mg/次，3次/d，口服。

促性腺激素醋酸亮丙瑞亮丙瑞亮丙瑞林（1euprolide acetate）是一种促性腺激素释放激素促进剂。一些作者发现行经期妇女较易出现IBS症状，故可试用此药。6个月试用发现此药对IBS患者的腹痛、恶心、呕吐等症状有明显改善作用。

奥曲肽（Octreotide）为长效人工合成的八肽生长抑素，对其他大多数激素如缩胆囊素及内啡肽都有抑制作用，故可试用于治疗IBS，但其价格昂贵。

5-羟色胺在体外对肠运动功能有多种作用，可加强收缩或促使松弛，但在人体内尚未开展研究。

①十六角蒙脱石（思密达）：为天然矿物质，系双八面体蒙脱石，该药为层纹状结构，有很强覆盖力，对病毒、细菌及毒素有极强的固定、清除力，防止肠上皮细胞损伤，并可吸收肠内气体，降低肠道的敏感性。本药不被细胞吸收，不进入血循环，故无毒副作用，可与抗生素、益生菌制剂等并用。十六角蒙脱石（思密达）每袋3g，倒入50ml温水中搅匀口服，每次服1～2袋；亦用1～3袋混于50～100ml温水，保留灌肠，1～3次/d。

②促性腺激素醋酸亮丙瑞亮丙瑞亮丙瑞林[leuprolide acetate（1eupron）]：是一促性腺激素释放激素促进剂。有人用以治疗IBS使腹痛、恶心、呕吐等症均得以明显改善。

③钠铬铜（naloxone和natrexem）：是一鸦片对抗剂，可使肠蠕动亢进，对便秘的IBS病人应用较好。

秒懂百科
金灿荣
详解2022年俄乌冲突
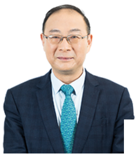

④应用调节自主神经药：如谷维素每次服20～60mg，3次/d；亦可应用泛酸钙、桂利嗪（肉桂嗪）等。

辨证论治或根据征候选用麻仁滋脾汤、七味白术散等加减，亦可试用健脾益肠丸。有报道针灸治疗也有一定疗效。

预后

播报 编辑

无生命威胁，可长期保持健康生活

易激结肠的预防

播报 编辑

减少对消化道的不良刺激，避免食物过敏反应和少摄入能在消化道内产气的食品。应避免过分辛辣、甘、酸、粗糙等刺激性食物。多食易消化、富营养的食品。便秘患者应多摄入富含纤维素的食品和水果。对有过敏史者，就避免摄入可能引起过敏的食物。对疑有乳糖不耐受者，应避免摄入大量牛奶及牛奶制品。宜细嚼慢咽、戒烟、少饮碳酸饮料。

如患者有明显的精神、神经因素，则应从解除病人多疑虑的心态，消除恐惧的心理着手，预防本病的发生或加重。

相关药品

播报 编辑

乳果糖、硝酸甘油、甘油、氟哌噻吨、氟哌噻吨/二甲胺丙烯、阿米替林、多塞平、地西泮、山莨菪碱、贝那替秦、美贝维林、双环维林、西托溴铵、醋丁洛尔、可乐定、硝苯地平、维拉帕米、洛哌丁胺、葡萄糖、嗜酸乳杆菌、乳酸菌、聚克通、佳士康、酪酸梭状芽孢肝菌、米雅、氧、地衣芽孢杆菌、乳酸菌素、乳酶生、西沙必利、甘露醇、醋酸、亮丙瑞林、奥曲肽、生长抑素、蒙脱石、谷维素、泛酸钙、桂利嗪

相关检查

播报 编辑

5-羟色胺

搜索发现

哪里有钢格板厂家 机器人教育加盟哪家好 孕妇护肤品哪种好 礼堂椅生产批发厂家 机房用空调
折叠自行车 跑车租车

新手上路
成长任务 编辑入门
编辑规则 本人编辑 NEW

我有疑问
内容质疑 在线客服
官方贴吧 意见反馈

投诉建议
举报不良信息 未通过词条申诉
投诉侵权信息 封禁查询与解封

©2022 Baidu 使用百度前必读 | 百科协议 | 隐私政策 | 百度百科合作平台 | 京ICP证030173号
京公网安备11000002000001号

秒懂百科
金灿荣
详解2022年俄乌冲突

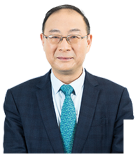

Supplement: Data S1 [file peerj-12-17264-s001.zip › σÄƒσoïμò░μì«/Baidu/Baidu-Chinese/4-μÿôμ┐Çτ╗ôΦéá_τÖ╛σ║aτÖ╛τoæ.pdf]
