# Supplementary material for: Assessment of medical information on irritable bowel syndrome information in Wikipedia and Baidu Encyclopedia: comparative study
Source: PeerJ. 2024 May 24;12:e17264. doi: 10.7717/peerj.17264 (PMC11129691; doi:10.7717/peerj.17264)

近期有不法分子冒充百度百科官方人员，以删除词条为由威胁并敲诈相关企业。在此严正声明：百度百科是免费编辑平台，绝不存在收费代编服务，请勿上当受骗！ 详情>>

收藏 16 17

慢性功能性便秘

播报 编辑 讨论 上传视频

常见病

慢性功能性便秘是一种常见病、多发病。主要是指排便次数减少、粪便量减少、粪便干结、排便费力等。必须结合粪便的性状、本人平时排便习惯和排便有无困难作出有无便秘的判断。如超过6个月即为慢性便秘。

多发群体	老人，女性	西医学名	慢性功能性便秘
常见病因	饮食因素，精神压力	所属科室	内科 - 消化内科

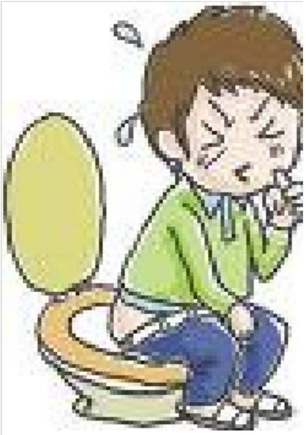

慢性功能性便秘的概述图（1张）

相关视频

查看全部 >

8085播放 | 01:21

如何治疗便秘？

1.4万播放 | 01:24

有一种便秘叫“慢性功能性便秘”，你要知道！

词条统计

浏览次数：244558次

编辑次数：18次历史版本

最近更新：涵宇Q （2021-12-0…

突出贡献榜

bianmi8

目录

1 诊断标准
2 病因病机
3 形成原因
4 食疗方法
5 治疗便秘方法
6 缓解便秘妙招
7 辨证施治要点
8 慢性便秘治疗
9 不同病症区别

1 自己怎样建网
2 焊缝无损检测
3 价格便宜的香
4 电脑主机多少
5 蓄热式焚烧炉
6 高温隔热材料
7 哈佛大学申请
8 俄语口语学习
9 新概念英语网
10 战队logo设计
11 10万级无尘车
12 dna
13 去
14 螺
15 vr消防演练
16 无人机反制
17 自己创建个
18 csgo电脑配
19 自己建个网
20 机器人展会
21 什么叫云计
22 动物焚烧炉

诊断标准

播报 编辑

诊断标准：在过去的一年里至少3个月连续或间断出现以下2个或2个以上症状：(1)少于1/4的时间内有排便费力；(2)少于1/4的时间内有有粪便干结；(3)少于1/4的时间内有排便不尽感；(4)少于1/4的时间内排便时有肛门阻塞感或肛门直肠梗阻；(5)少于1/4的时间内有排便需用手法协助；(6)少于1/4的时间内有每周排便少于3次。不存在稀便，也不符合肠易激综合征的诊断标准；同时需除外肠道或全身器质性病因以及药物因素所致的便秘。

病因病机

播报 编辑

《伤寒论》将便秘分为阳结、阴结、脾约。唐容川《血证论》云：“肺移热于大肠则便结，肺津不润则便结，肺气不降则便结”。便秘的发生与大肠、脾、胃、肺、肝、肾等脏腑关系密切。《内经》云：“大肠者，传导之官，变化出焉。”又提出“肺与大肠相表里”。脾胃为运化水谷之海，脾主运化，胃主和降，胃与肠相连，水谷入口，经脾的运化输布，胃的腐熟收纳，最后将糟粕转输于大肠。这其中也依赖于肝主疏泄的功能，若肝郁气滞，则腑气不通，气滞不行，则大肠失运。肾司二便，肾气不足，则大肠传导无力，大便难于排出。一般认为，素体热盛，或恣食肥甘厚味，易致胃肠积热，耗伤津液，便燥难排；忧思恼怒，或久坐少动，易导致气机郁滞，通降失常，糟粕内停；外感寒邪，过食寒凉，导致阴寒内盛，凝滞胃肠，糟粕传导不能；饮食劳倦，年老体虚，大病产后，均可因体质的不同，而出现气虚阳衰、阴亏血少等情况，导致大肠传导无力，或肠道失濡，而致大便秘结。

西医学将功能性便秘分为慢传输型、出口梗阻型、混合型三种。中医认为，慢传输型便秘多为虚证便秘，如年老体弱、久病产后、素体虚弱；或饮食劳倦、过食生冷、感受寒湿，伤及脾胃，均可导致肺、脾、肾诸脏虚弱。脾虚则运化无力，水谷精微不得转输，糟粕无力运行于大肠。肺虚则宣降失常，肺与大肠相表里，大肠因而气机不利，运行受阻。华佗《中藏经》云：“大肠者，肺之腑也，为传导之司，监仓之官。肺病久不已，则可下传大肠”，《医经精义·脏腑之言》曰：“大肠之所以能传导者，以其为肺之腑。肺气下达，故能传导”。肾主水，司二便，肾虚则肠道失濡，开合不利，糟粕受阻。金元《兰室密藏·大便结燥》曰：“夫肾主五液，津液润则大便如常。……又有年老体虚，津液不足而结燥者。”

出口梗阻性便秘多为实证便秘。如忧思恼怒，肝郁气滞，过食生冷肥甘厚腻、辛辣刺激食物，或嗜烟酒，感外邪，根据体质不同邪从热化或邪从寒化，导致或肠胃积热，或阴寒积滞，或气机郁滞，从而腑气不通，胃气不降，大肠失于传导。如《内经》云：“太阴之厥，则腹胀而后不利”，提到便秘与脾胃受寒有关；《内经》云：“热气留于小肠，肠中痛，瘅热焦渴，则坚干不得

秒懂百科
金灿荣
详解2022年俄乌冲突
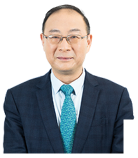

出，故痛而闭不通矣”，说明肠胃积热便秘的病理基础。肝主疏泄，有助于大肠传导，肝气郁结，则大肠气机不利，腑气不通。如《金匮要略·便秘》云：“气秘者，气内滞，而物不行也。”

混合型便秘多为虚实夹杂型，或为虚中挟实性便秘，其本为虚，其标为实，本虚为脾、肺、肾虚弱，标实则为大肠气机不利，腑气不通。

便秘的病因是复杂的，在疾病的发展过程中，各种证候可相兼出现，或互相转化。辨证应以虚实为纲，阴阳气血为目。其病位在大肠，病机与脾胃肺肝肾有关。西医疾病分型与中医辨证分型有其内在规律可循，但因为疾病本身的多变性和复杂性，故不能拘泥于此。治疗时应审证求因，审因论治。

形成原因

播报 编辑

1、饮食因素：一些人饮食过少，食品过精过细，食物中的纤维素和水份不足，对肠道不能形成一定量的刺激，肠蠕动缓慢，不能及时将食物残渣推向直肠，在肠内停留时间延长，水份过多吸收而使粪便干燥。

2、药物影响：长期滥用泻药，使肠壁神经感受细胞的应激性降低，即使肠内有足量粪便，也不能产生正常蠕动及排便反射，因而导致顽固性排便困难。

3、大肠病变：如过敏性结肠炎、大肠憩室炎、先天性巨结肠等疾病可引起大肠痉挛、运动失常，使粪便通过不畅而发生排便困难。

4、拖延大便时间：一些人把大便当作无关紧要，可早可迟的事，忽视定时排便的习惯，使直肠壁上的神经细胞对粪便进入直肠后产生的压力感受反应变迟钝，使粪便在直肠内停留时间延长而不引起排便感觉，形成习惯性排便困难。

5、排便动力不足：排便时不仅需要肛门括约肌的舒张，提肛肌向上向外牵拉，而且还需要膈肌下降、腹肌收缩、屏气用力来推动粪便排出。年老体弱、久病卧床、产后等，可因膈肌、腹肌、肛门括约肌收缩力减弱，腹压降低而使排便动力不足，使粪便排不干净，粪块残留，发生大便困难。

6、精神因素：精神上受到强烈刺激、惊恐、情绪紧张、忧愁焦虑或注意力高度集中某一工作等会使便意消失，形成排便困难。另外还有神经系统障碍、内分泌紊乱、维生素缺乏等亦可引起排便困难 [1] 。

食疗方法

播报 编辑

便秘患者多吃什么好

1、每日至少喝8杯水，尤其在食用高纤维食品时，更应注意保证饮水；

2、多吃新鲜蔬菜，增加饮食中纤维的摄取量；

3、每天加食糠皮、麦麸等，以扩充粪便体积，促进肠蠕动，减少便秘的发生；

4、每天早上起来空腹喝温水冲的蜂蜜水、蜂蜜对肠道有润滑作用；

5、增加B 族维生素食品的供给，尽量选用天然、未经加工的食品，如粗粮、豆类、酵母等，以增强肠道的紧张力；

6、多吃芹菜、核桃仁、熟香蕉、核桃、柚子、苹果、葡萄柚、糙米、胡萝卜、红薯等。

治疗便秘方法

播报 编辑

疗方1：芝麻（黑芝麻最佳）焙熟研细末2～3羹匙，兑温开水（开水待温后）200～300毫升加10天成熟洋槐蜂蜜3～5羹匙调成糊状口服，早、晚各1次。用此疗方治疗习惯性便秘50例，见效最快者2天，最长者10余天，2～10天见效40例，10天以上见效10例，50例均获良效。

疗方2：蜂蜜60克，每日早、晚各服30克，以凉开水冲饮。适用于老年、孕妇便秘及习惯性便秘。

疗方3：蜂蜜60克，蜂王浆6克，将其调匀，每日早、晚分2次用温开水送服，适用于习惯性便秘。

疗方4：蜂花粉150克研碎，加入蜂蜜250克调成膏，每日早、晚空腹服1汤匙，适用于习惯性便秘。

疗方5：10天成熟洋槐花蜂蜜、白萝卜适量，先将白萝卜洗净切成片，蘸蜂蜜生食，每日数次。最适用于青少年便秘者。

疗方6：蜂蜜、香蕉适量，将香蕉剥皮以其肉蘸蜂蜜生食，每日数次。最适用于老年人及习惯性便秘者。

缓解便秘妙招

播报 编辑

跳绳可缓解便秘

秒懂百科
金灿荣
详解2022年俄乌冲突
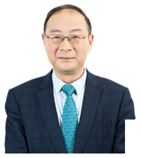

女性发生便秘的几率要比男性高。首先，因为女性吃东西少、细、精，因此肠道蠕动更慢。其次，女性运动量较小，而坚持规律的锻炼可以使肠道更好地蠕动。

防治便秘最好的方法就是通过跳绳振动内脏。弹跳能刺激骨骼、肌肉，促进血液循环，此外还能加强淋巴系统的免疫功能，这对缓解便秘十分重要。

便秘的人走路时，可以尽量加大腰和胯部的转动，像模特一样走猫步，这能起到对腹腔按摩的作用，能够加强内脏特别是肠胃的蠕动，促进营养的吸收和废弃物的排出，对肠胃功能失常、消化不良引起的便秘，有特别明显的疗效。

卫生间装饰成淡黄色有助排便

日本新研究发现，卫生间里装饰一些黄色，有助于排便。黄色是暖色调，有放松心情、缓解紧张情绪的作用。人们在排便时，身体部分肌肉处于紧张收缩状态，这时保持愉快的心情，能放松肌肉，有助排便。卫生间里的颜色选暖色调，比如淡黄色、淡绿色、淡蓝色、淡橙色等，可以帮你安神，而大紫大红的颜色则容易刺激神经，不利于精神的放松。在空间相对狭窄的浴室里，黄颜色还能起到开阔视野的效果，使得整个卫生间更加柔和、宽敞和明亮。

喝醋防便秘

醋的酸性成分与胃里的消化液相似，故而食醋可以起到刺激肠胃、促进肠道蠕动的作用。而随之增强的排便感，可避免大便在体内长时间存留、干结，对预防便秘有不错的效果。人在饱腹时，肠道被填满，醋不易刺激到肠胃，故食醋通便，最好选在早晚空腹时。

食用时，每次不要超过1汤匙，但也不能少于半汤匙，1天最多不能超过3次。

食用后，紧接着要饮一杯温开水。因为原汁醋的浓度太大，如果食用过多，可能会对胃黏膜造成一定损伤，所以一定要掌握好醋的食用量。

也可在一汤匙陈醋中加入等量的蜂蜜，用温开水或凉开水均匀搅拌后喝下。

葡萄干治疗便秘

葡萄干治便秘的说法，在古代医书中并没有记载，至今相关报道也极少，而且对其有两种相反观点：有报道称，葡萄干含有纤维和酒石酸，能让排泄物快速通过直肠，每天吃一小把可防治便秘；也有人认为，葡萄干多食“易生内热”，反而会加重便秘。

但这两种观点，现均没有可靠的实验或临床研究加以证明。实验表明，从葡萄籽中提炼的葡萄籽油，对动物可以起到润肠通便功效。

但市售葡萄干多为无籽的，治好便秘的几率极小，即使偶尔有效，也只是个例，并不说明此方法对其他人也有效，不宜效法。

起床后喝杯白开水

晨起一杯白开水可以补充身体代谢失去的水分，又可以促进排泄，防治便秘。还能稀释血液，促进血液循环，对减肥女性尤为重要。也可以用淡盐水代替白开水。

洗澡巧揉肚子治便秘

洗澡时可以用手掌在腹部按顺时针方向按摩，同时腹部一鼓一收地大口呼吸，并淋浴腹部，可治疗慢性便秘并防治痔疮。而神经性便秘，则要沿着肠部用40℃热水冲3分钟左右，再用25℃的温水冲10秒钟，反复5次，可让大肠的蠕动增加。

核桃蜂蜜茶治疗便秘

核桃蜂蜜茶是民间常用的便秘偏方，有很好的治疗便秘的疗效。具体的方法是，用核桃5克，蜂蜜1克，用温开水冲泡后代茶喝，即可达到润肠通便的效果。

辨证施治要点

播报 编辑

1.重视脾阴，液增舟行

朱丹溪《局方发挥》云：“脾土之阴受伤，传输之官失职”。《素问·玉机真脏论篇》云：“脾不足，令人九窍不通”。《类经》训诂曰：“不足病在中，故令九窍不通，以脾气弱者四脏皆弱而气不行也。”《金匮要略》认为，脾阴不足，不能为胃行其津液，肠道失润，即形成脾约之证，导致便秘。《脾胃论》曰：“胃者卫之源，脾乃营之本”、“四季脾旺不受邪”，营为阴，脾阴充足，则脾转输功能正常，精微得以化生，糟粕得以传导。长期习惯性便秘患者，尤其是老年患者，多为脾气或脾阴不足，或气阴两虚。治疗时应顾护脾气脾阴，润肠通便。健脾气多用四君子汤，养脾阴多用沙参麦门冬汤。

2.通降和胃，积滞自除

“胃者，五脏六腑之海也，水谷皆入于胃，五脏六腑皆禀气于胃”，“胃者人之常气，人无胃气曰逆，逆者死。”李杲也说：“内伤脾胃，百病由生。”胃以通降为和，胃气和则浊气下降，糟粕得以排出大肠。通降可用枳实、厚朴、莱菔子，和胃可用生姜、大枣、甘草。

百度百科
金灿荣
详解2022年俄乌冲突
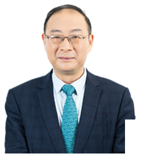

3.审证求因，用药有别

在治疗功能性便秘时，应因证择方，因人施药。脾虚型便秘，大剂量用生白术30～60g以健脾补气通便；血虚肠燥者大剂量用生白芍、火麻仁以补血润肠通便；肾阴虚者重用何首乌、生地以补肾滋润通便；肾阳虚者重用肉苁蓉以温补肾阳通便；脾气虚重用生黄芪30～50g；痰热壅肺、肺失宣降者加用杏仁、黄芩、栝楼仁以宣肺清热通便；大肠实热者用大黄、虎杖以清热泻腑通便；久病多瘀者则用桃仁、酒大黄以活血化瘀通便；肝郁气滞者重用郁金、枳实以疏肝理气通便。

慢性便秘治疗

播报 编辑

首先应加强科学的生活管理，保持良好的精神及心理状态，注意高速饮食结构，多食富含纤维的食物，足量饮水，养成按时排便习惯，参加适当的体育锻炼等，还可进行腹式呼吸锻炼或按摩。

1.药物治疗：（1）通过上述方法达不到疗效时可考虑药物治疗，对于STC患者，首选是促动力剂，西沙必利作为一种全胃肠道促动力剂，对某些STC患者有效。一种新型特异性促肠动力药普卡必利晚近已问世，该药系苯并呋喃族化合物，特异性作用于5-HT4受体，可望成为一种理想的治疗CFC的药物。Soffer等[10]报道，misoprostol可用于治疗顽固性便秘。某些中药可能具有促肠动力作用，也可选择性应用。（2）常用泻剂：①容量性泻药：硫酸镁、硫酸钠、甲基纤维素、琼脂等；②刺激性泻剂：番泻叶、蓖麻油、双酯酚汀等；③粪便软化剂：液体石蜡、乳果糖等；④直肠内给药：甘油栓、开塞露等。应避免长期滥用泻剂而导致泻剂性肠病。

2.生物反馈排便行为治疗：生物反馈治疗法是一种纠正不协调排便行为的训练法，主要用于治疗肛门括约肌失协调和盆底肌、肛门外括约肌排便时矛盾性收缩导致的FOOC，有人报告其疗效可达96%，该法与药物治疗相比具有无药物副作用、成本低、非创伤性等优点，国内已开展此项疗法。

3.手术治疗：对于严重慢性便秘经上述治疗无效，严重影响患者生活质量甚至营养障碍者，可采取手术治疗。主要是对STC患者，可采取结肠次全切除和回直肠吻合术，对FOOC患者可行肛管直肠括约肌切除术。这种手术虽然可恢复肠管的节律性蠕动，缩短全胃肠通过时间，但必须严格掌握适应证，同时也必须重视术后并发症如腹泻、大便失禁、甚至复发等问题。

不同病症区别

播报 编辑

功能性便秘(functionalconstipation)和盆底功能障碍(pelvicfloordyssynergia)：前者指无器质性疾病证据的慢性便秘，部分功能性便秘患者有盆底障碍的表现，后者需伴有盆底障碍的表现，即符合罗马II的功能性便秘的诊断标准，还需具备盆底障碍的客观依据。

(1)有肛门直肠测压，肌电图或X线检查的证据，表明在反复作排便动作时，盆底肌群不合适的收缩或不能放松。

(2)力排时直肠能出现足够的推进性收缩。

(3)并有粪便排出不畅的证据。

词条图册

更多图册 >

参考资料

1 引起大便困难的原因 . 新浪健康[引用日期2014-09-14]

学术论文

内容来自 Bai学术

于金源，孙长岗，尹国富．从肝论治慢性功能性便秘．《WanFang》，2004

金洵，丁义江，王玲玲等．针刺治疗慢性功能性便秘疗效观察．《CNKI;WanFang》，2010

王丽娟，王玲玲．麦粒灸结合针刺治疗慢性功能性便秘随机对照研究．《CNKI;WanFang》，2011

施永敏，周永香，马文芳等．老年慢性功能性便秘的干预护理．《实用临床医药杂志》，2005

郭荣，丁义江，张建淮．慢性功能性便秘诊断和中医证型的量化研究．《CNKI》，2011

查看全部 >

秒懂百科
金灿荣
详解2022年俄乌冲突

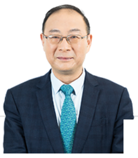

搜索发现

功能性便秘怎么治疗 便秘的解决方法 慢性便秘的症状 闭合性粉刺怎么排出来 乳果糖治疗便秘
为什么老是便秘 预防便秘的方法有哪些 突然便秘怎么办 便秘怎么办快速解决 便秘有什么危害

新手上路
成长任务 编辑入门
编辑规则 本人编辑 NEW

我有疑问
内容质疑 在线客服
官方贴吧 意见反馈

投诉建议
举报不良信息 未通过词条申诉
投诉侵权信息 封禁查询与解封

©2022 Baidu 使用百度前必读 | 百科协议 | 隐私政策 | 百度百科合作平台 | 京ICP证030173号
京公网安备11000002000001号

金灿荣
详解2022年俄乌冲突
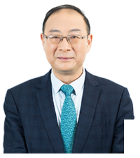

Supplement: Data S1 [file peerj-12-17264-s001.zip › σÄƒσoïμò░μì«/Baidu/Baidu-Chinese/9-μàóμÇoσèƒΦâ╜μÇoΣ╛┐τoÿ_τÖ╛σ║aτÖ╛τoæ.pdf]
