# Supplementary material for: Assessment of medical information on irritable bowel syndrome information in Wikipedia and Baidu Encyclopedia: comparative study
Source: PeerJ. 2024 May 24;12:e17264. doi: 10.7717/peerj.17264 (PMC11129691; doi:10.7717/peerj.17264)
Supplement: Data S1 [file peerj-12-17264-s001.zip › σÄƒσoïμò░μì«/Baidu/Baidu-Chinese/8-σèƒΦâ╜μÇoΣ╛┐τoÿ_τÖ╛σ║aτÖ╛τoæ.pdf]

网页 新闻 贴吧 知道 网盘 图片 视频 地图 文库 百科 | 百度首页 登录

Baidu百科 功能性便秘 进入词条 全站搜索 帮助

首页 秒懂百科 特色百科 用户 知识专题 权威合作 下载百科APP | 个

收藏 | 89 | 39

# 功能性便秘

播报 锁定 上传视频

本词条由好大夫在线 提供内容并参与编辑 。

郭晓峰 （主任医师） 山西省人民医院消化科

便秘是一个常见的临床症状，表现为粪便干结，排便困难、粪便重量和次数减少。随着社会的老龄化、现代生活节奏和饮食习惯的改变、疾病谱的变化等对疾病的影响，便秘已成为影响现代人生活质量的重要因素之一，而且与大肠癌发病关系密切。便秘，可由许多原因引起如神经源性，全身疾病等，称继发性便秘。如便秘不存在引起便秘的器质性病变称功能性便秘，以往曾认为是单纯性便秘、习惯性便秘或特发性便秘等。便秘患者滥用泻剂导致的泻剂性肠病和结肠黑变病已引起大家的关注，因为结肠黑变病与结肠癌有关，因此功能性便秘的治疗越来越受到重视。

| | | | |
|---|---|---|---|
| **中文名** | 便秘 | **所属科室** | 内科-消化内科 |
| **表　现** | 粪便干结，排便困难 | **发病原因** | 暂不明确 |

## 相关视频

查看全部 >

18万播放 | 05:11 发生便秘怎么办?

7385播放 | 04:31 让专家来为你解释一下，功能性便秘"罗马标准"｜央视网

7002播放 | 01:23 什么叫功能性便秘？有以下几种表现，可不要忽视了

5653播放 | 01:04 肠道百科-功能性便秘的诊断标准是什么?

目录

1 疾病介绍
2 疾病分类
3 发病原因
4 发病机制
5 临床表现
6 诊断鉴别
- 辅助检查
- 鉴别诊断
7 疾病治疗
- 饮食治疗
- 养成定时排便习惯
- 药物治疗
- 外科治疗
- 生物反馈
- 高电位治疗

## 疾病介绍

播报

功能性便秘是指缺乏器质性病因，没有结构异常或代谢障碍，又除外肠易激综合征的慢性便秘。功能性便秘患者可以有粪便坚硬、排便困难、便不尽感和便次减少等表现。表1列出了功能性便秘的罗马III诊断标准，诊断之前症状出现至少已有6个月，且近3个月症状符合以上诊断标准。

**表1 罗马III功能性<a href="#" data-lemmaid="332148"> 便秘</a>的诊断标准**

| |
|---|
| 1.必须符合以下2项或2项以上： |
| （1）至少25%的排便感到费力 |
| （2）至少25%的排便为干球状便或硬便 |
| （3）至少25%的排便有肛门直肠阻塞感或梗阻感 |
| （4）至少25%的排便需要手法帮助（如用手指助便、盆底支持） |
| （5）便次<3次/周 |
| 2.在不使用泻药时很少出现稀便 |
| 3.没有足够的证据诊断IBS |

便秘型肠易激综合症（IBS-C）的诊断要点患者有腹痛和/或腹胀症状，并与排便关系密切发作时排便频率及粪便性状改变有关。

权威合作编辑

好大夫在线
好大夫在线创立于2006年，中国领先的医疗信息...

什么是权威编辑

词条统计

浏览次数：226250次
编辑次数：28次历史版本
最近更新：壹统华夏 （2017-07-12）

突出贡献榜

haodf_hz
映雪红梅

1 自己创建个网
2 csgo电脑配置
3 哈佛大学申请
4 战队logo设计
5 vr消防演练
6 去德国留学
7 无人机反制
8 dna亲子鉴定多
9 10万级无尘车
10 新概念英语网
11 高温隔热材料
12 价格便宜的
13 自己怎样建
14 螺杆式家用
15 电脑主机多
16 俄语口语学
17 动物焚烧炉
18 自己[illegible]
19 MC服
20 GTX
21 机器
22 什么

秒懂百科
金灿荣
详解2022年俄乌冲突

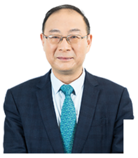

1999年提出的便秘概念系指大便量少，太硬排出太困难，并合并一些特殊的症状如长时间用力排便，直肠胀感，排便不尽感，甚至需手法帮助排便，1周排便少于2次或长期无便意。与国际标准相比更量化，更便于理解。

## 疾病分类

播报

为了便于临床治疗功能性便秘方案和药物的选择，目前大多采用根据结肠动力学特点而进行的分型，分为慢传输型便秘、功能性出口梗阻型便秘和混合型便秘。这种分型的依据是以结肠或肛门直肠动力障碍特点为基础的。

**（1）慢传输型便秘：**是最常见的类型，系指由于结肠动力障碍，使内容物滞留于结肠或结肠通过缓慢的便秘，结肠测压显示结肠动力降低，导致结肠内容推进速度慢，排空迟缓。同时可能伴有其它自主神经功能异常所致的胃肠功能紊乱如胃排空迟缓或小肠运动障碍。患者主诉多为排便次数少、粪便质地坚硬、无便意。用闪烁照像术或不透X线标记物法检查提示结肠通过时间延缓可确立诊断。因此有人称之为结肠无力，它是功能性便秘最常见的类型。治疗上首选促肠动力剂。

**（2）出口梗阻型便秘：**具有正常的结肠传输功能，由于肛、直肠的功能异常（非器质性病变）如排便反射缺如，盆底肌痉挛综合征或排便时肛门括约肌不协调所致。包括横纹肌功能不良，直肠平滑肌动力异常，直肠感觉功能损害，肛门括约肌失协调症以及盆底痉挛综合征等。患者主诉是排便困难，肛门直肠阻塞感，排便时需要用手协助。多发生于儿童、妇女和老年人。治疗上可选择生物反馈治疗。

**（3）混合型便秘**：具有结肠慢传输特点，也存在肛、直肠功能异常，或二者均不典型，治疗上因人而异。该型可能是由于慢传输型便秘发展而来，也有人认为长期的出口梗阻影响了结肠排空继发结肠无力。

## 发病原因

播报

功能性便秘的病因学并不十分明确，可能是多因素的影响。研究表明功能性便秘老年人发病率高，与进食量、老年性胃肠道功能下降如肠管分泌消化液减少、肠管张力蠕动减弱以及参与排便肌肉张力低下有关。某些主诉功能性便秘的病人，可能有明显的食物因素，如低渣饮食。食物中增加30g/d植物纤维可明显增加肠蠕动，称为纤维素样效应。精神心理因素也占主要地位，功能性便秘患者忧郁，焦虑明显增多，功能性便秘患者存在自主神经功能异常。功能性便秘患者中，可能伴有全胃肠的功能障碍，如胆囊和胃排空及小肠运转缓慢等。

**(一)**由于不良的饮食习惯，使得食物中所含机械或化学的刺激不足(如蔬菜中的纤维素)或因摄台量过少，尤其是缺少遗留大量残渣的食物。使肠道所受刺激不足，反射性蠕动减弱造成便秘。

**(二)**在结肠的总蠕动后，粪块进入直肠，从而引起排便反射。但当便意经常被忽视，排便场合和排便姿势不适当，以及经常服用强泻剂或洗肠等，均可造成宜肠反射敏感性减弱。以致虽有粪块进入，而不足以引起有效的神经冲动，故排便反射无由产生，结果造成便秘。

**(三)**精神抑郁或过分激动，使条件反射发生障碍，高级小枢对副交感神经抑制加强，使分布在肠壁的胸腰支交感神经作用加强，因而产生便秘。相当一部分功能性便秘患者发病前曾有心理障碍。

**(四)**不良的生活习惯，睡眠不足。持续高度精神紧张状态等，亦可造成结肠的蠕动失常或痉挛性收缩，因而造成便秘。①食物过少或过精，缺少纤维残渣对结肠运动的刺激。②妊娠：妊娠后期平滑肌动力减低，可能是由黄体酮的作用所致。③生活规律的改变。④某些药物：如鸦片、吗啡、可待因、抗胆碱能和神经节阻滞药、镇静药、抗郁药、某些制酸剂(碳酸钙、氢氧化铝)等。此外，经常应用酒肠和服用泻药，可使肠道的敏感度减弱，以致引起或加重便秘。

## 发病机制

播报

便秘可以看作是不同病理生理过程的最终症状表现。排便过程需外周神经兴奋，将冲动传到初级排便中枢和大脑皮层，引起结肠、直肠和肛门括约肌及盆底肌肉的协调运动而完成。任何一个环节发生障碍都可导致便秘。

1 结肠 结肠运动形式中蠕动最为重要。是由一些稳定向前的收缩波组成。还有一种进行很快且推进很远的蠕动，即集团性蠕动。集团蠕动常见于餐后，由于十二指肠-结肠反射所引起。肠道内容物的移动由餐后结肠各部分压力梯度决定，集团蠕动是维持肠道正常功能所必须的。

2 直肠肛管 正常排便时，当粪便进入直肠便产生便意，肛门内括约肌松弛，对包绕其外的肛门外括约肌环形成扩张作用，直肠收缩使直肠腔内压力超过肛管压力同时，排便反射发生，肛门内括约肌松弛使粪便排出。肛管内压超过直肠内压而引起排便困难是出口梗阻型便秘的常见动力障碍。盆底痉挛综合征患者排粪造影显示肛管直肠角缩小，用力排便时不增大，盆底直肠前突深度和直肠排空时间相关。耻骨直肠肌痉挛综合征肌电图表现为矛盾性耻骨直肠肌收缩。另一个重要的病理生理是盆腔底功能失调，其特点是结肠通过正常或轻微减慢，但粪便残渣在直肠中储留延长，其主要缺陷是不能从直肠排出其内容物。这种功能性缺陷还有许多其他名称(出口梗阻、大便困难、松弛不能、盆腔底协同失调)。对导致不能将粪便从直肠排出的这一推论性病理生理的理解尚不深。最简单的可能分类为：(1)肌肉高张力(松弛不能)；盆腔底不完全松弛以及试图排便时盆腔底和肛门外括约肌的矛盾收缩。(2)肌肉低张力，有时伴有巨直肠和盆腔底过度降低。这些综合征是多因素的，有些尚不甚了解。

3、肠壁肌层及肌间神经丛的病理改变　许多研究资料显示，便秘病人的结肠壁有肌纤维变性、肌肉萎缩、肠壁肌间神经丛变性、变形、数量减少等病理改变。

4、肠壁内神经递质的变化

## 临床表现

播报

秒懂百科
金灿荣
详解2022年俄乌冲突
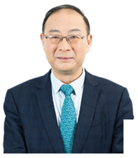

由于粪块在乙状结肠与直肠内过度停滞，病人有时左下腹有胀压感，常有里急后重欲便不畅等症状。痔疮常作为便秘的继发症出现。在习惯用泻药或洗肠的病人，由于胃肠运动功能的紊乱可出现上腹饱胀不适、嗳气、反胃、恶心、腹痛、腹鸣、排气多等主诉。长期便秘在部分病人可出现轻度“毒血症”症状，如食欲不振、口苦、精神萎靡、头晕乏力、全身酸痛等。至于造成轻度贫血及营养不良者较为少见。少数病例有臀部、大腿后侧隐痛与憋胀感觉，是由于粪块压迫第三，四及五脊神经根前支所致。对于头痛，疲倦，失眠等神经衰弱症状，与其说是功能性便秘的后果，不如说是它的原因。

粪便性状常成为患者的特有主诉。直肠便秘者排出的粪便多为粗大块状，而结肠便秘则多为小粒，类似羊粪状。硬便的机械性刺激引起直肠粘膜分泌粘液，常覆在硬粪的表面及缝隙间，有时呈粘液膜状排出。便秘患者有时于排便过程中，突然腹痛发作。开始排出硬便，继之有恶臭稀便排出称为“假性腹泻”。

多数病人体征不明显。在痉挛性便秘时往往可们及痉挛收缩的肠管。直肠便秘时在左下胶常可触到粪块，肛门指诊时触到坚实粪块，排便后指诊发现因壶腹扩张四处空旷，而不易触到肠壁。

在罗马标准定义中，排便频率仅是6种基本特征(包括摒力、大便坚硬和排便不尽感)之一。患者定义包含的症状是(重要性次序)：摒力、大便过度坚硬、大便急而无效、大便次数少以及排便不尽感。症状评定应包括针对特异症状的病史。必须列出完整的处方和非处方药物。便秘副作用在常用药物中很广泛。大多数便秘患者为缓解其症状，常自己应用非处方药物。人群研究显示，轻泻药的使用和滥用分别为7%和4%。

## 诊断鉴别

播报

### 辅助检查

**(一)** 胃肠x线检查 根据钡剂在胃肠道内运行的情况来了解其运动功能状态。在张力减慢性便秘者，可看到钡剂到达结肠后运行明显减慢，在左侧结肠内长期停滞，特别显出扩张的直肠壶腹。在痉挛性便秘者，可见结肠内钡剂被分成小块，并可见到由于逆蠕动的结果， 使到达降结肠或乙状结肠的钡剂，有时又可逆行到横结肠。胃肠x线检查的更大意义在于排除肿瘤、结核、巨结肠症、梗阻等器质性病变造成的便秘，这对确立功能性便秘的诊断是非常重要的。

**(二)** 直肠镜，乙状结肠镜及纤维结肠镜检查 可直接诊视肠粘膜状态，必要时采取活组织检查。在功能性便秘病人，由于硬粪的滞留和刺激，结肠粘膜特别是直肠粘膜常有不同程度的炎性改变。表现为充血、水肿、血管走向模糊不清等。在挛缩性便秘者，除炎性改变外，有时肠镜下可见到肠管的痉挛性收缩。表现为肠壁向腔内聚拢，肠腔收缩变窄，推进肠镜困难，同时病人感到腹痛。稍停片刻挛缩即可缓解，肠腔开放，腹痛消失。

**(三)** 排粪造影 是一种形态与动态相结合评价肛门直肠区功能的方法。采用X线造影技术，测静坐、提肛、强忍、用力排便各时相的肛门直肠角、肛上距、乙耻距。用于诊断解剖畸形（直肠脱垂、直肠突出等）和肠道远端局部功能障碍（功能性出口梗阻、直肠乏力等），在便秘中有重要价值，并可为选择治疗方法提供依据。

**(四)** 肛门直肠测压 对功能性便秘的病因诊断及治疗也是很有帮助的。肛管、直肠测压在诊断慢性便秘中有十分重要作用，借助于此项检查可区分终末性和其它类型的便秘。常用的参数有肛管内括约肌压力及长度、肛管最大缩榨压、直肠敏感性及直肠肛门反射等。

**（五）** 肛管直肠感觉检查 用电流刺激法测肛门感觉。将通电探针与肛门粘膜接触，分别测肛门括约肌上、中、下三处，逐渐增加电流量，直到患者出现烧灼感或麻刺感，记录阈值，计算平均阈值。正常值为2．0—7．3mA。用气囊扩张法测直肠敏感性。Kamm等报道采用电流刺激法更为精确，避免了气囊压力、直肠内径和顺应性差异。两种方法测得结果有显著相关性。但后者易接受且可重复性好。

**（六）** 肛门括约肌肌电图 将针状电极或柱状电极插入肛门外括约肌皮下束记录肌电活动。便秘患者最常见的EMG改变为耻骨直肠肌矛盾收缩。用EMG可区分盆底随意肌群肌肉和神经功能异常，77%患者排便时盆底肌肉不能松弛，对出口梗阻型便秘的诊断有重要意义。

### 鉴别诊断

功能性便秘的诊断须依靠病史，分析便秘的原因，配合指诊可作出便秘的诊断。必要时可进行胃肠道X线钡剂或/和结肠镜检查，以排除器质性疾病，确定功能性便秘的诊断。

首先必须弄清患者所称便秘的确实含意，有许多人误认为只有每天排便1次才算正常，也有人因内痔脱垂，引起肛门异物感而误认为排便不全。在询问大便是否干硬时应明确粪便的物理性状，因为有些患者在回答“大便干燥”时，实际上只是略干的成形便而已。也有些慢性便秘患者，经常服用缓泻剂排便，如不详细询问，可误以为便次正常。故只有自然排便(非服用泻剂排便)少于每周3次，或大便干硬，或大便不干硬而排出困难，并伴有不适，才能认为是便秘。

起病时间对诊断有一定意义，幼年起病提示病因与先天因素有关，而近期发病则多为肠道器质性病变或饮食环境因素所致。伴有排便疼痛者提示肛管附近有病变，而排便无痛却伴有血和粘液者则多为结、直肠腔内病变。

不良饮食习惯如进食量少、饮水少、偏食、不喜食蔬菜及不良排便习惯如经常忽视便意等常可直接提示初步的诊断，如有的商店营业员、纺织厂的女工，因上班有意少饮水甚至不饮水而造成慢性便秘。逐步升级地滥用泻药是造成顽固性便秘难以纠正的另一大原因，必须详细询问用药种类、使用方法、起止时间及用药效果。因其他疾病而长期服用某种可致便秘的药物是常易遗漏的病因。

腹部及会阴手术史应予记录并问明与便秘发生的关系。一些较为特异的表现如排便时间延长，反复过度用力，直肠胀满，排便不全，手助排便(即用手指伸入肛门或阴道以协助排便)常提示盆底出口病变。粪便的物理性状有时也能帮助判断病变部位，长期排板栗状干硬便提示便秘可能是结肠性的，而软便排出困难、粪块变细者则提示便秘的原因可能在直肠、盆底。

秒懂百科
金灿荣
详解2022年俄乌冲突
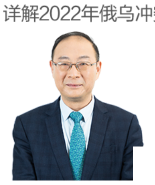

由于便秘不是一种独立的疾病，而是多种病因引起的一组症状，故对便秘的诊断应重在病因诊断，而不是症状诊断，诸如“慢性便秘”、“习惯性便秘”等。仅作出症状诊断是不完整甚至危险的，并有误诊、漏诊重大病变的可能。接诊者应按常规对病人进行全面、系统的检查，尤在导致便秘的原发病的特征性表现尚不明显，而首先表现为便秘症状时，这一点特别重要。作者曾见数例被诊断为“慢性便秘”的病人，未作常规检查，在等待特殊检查的过程中发生便血、肠梗阻，经常规检查很容易地诊断为直肠癌、结肠癌。过去亦曾有手术疗效不好的便秘患者，最后被确诊为糖尿病、系统性硬化症等。

因此，便秘一词，不应成为独立的诊断，在其项下，应列出可能的病因。对一时难以明确原发病的患者，必须先排除已知的重大器质性病变。只有在全面系统检查后仍未能发现已知的器质性病变时，才考虑进行有关功能检查，如肠道转运、肛肠动力学、排粪造影、盆底肌电图等。

结肠转运功能检查：系利用不透X线标志物，口服后定时拍摄腹部平片，追踪标志物在结肠运行中的情况，为判断结肠内容物运行速度及受阻部位的一种方法。

肛肠动力学检查：利用压力测定装置，检查内外括约肌、盆底、直肠功能状态及它们之间的协调情况，对判断便秘与上述结构的功能失常是否相关有重要意义。

盆底肌电图检查：应用电生理技术，检查盆底肌、耻骨直肠肌、外括约肌等横纹肌的功能状态，及其支配神经的功能状态。由于该项技术对检查者的要求较高，检查结果亦较难判断，所以目前仅用于观察模拟排便时盆底横纹肌有无反常放电的情况。使用针电极者，因系创伤性检查，易诱发保护性反射而造成假阳性，尤其在同时使用多根针电极时，经验不足者常判断失误，应引起注意。

排粪造影检查：将钡剂注入直肠、结肠(有时还可口服钡剂以观察小肠)后，患者坐在易透X线的便器上，在患者排便的过程中，多次摄片或录像，以观察肛管、直肠的影像学改变。

检查者应亲自阅片，结合临床资料与其他检查结果综合判断，不能仅凭影像资料诊断。组织学检查：疑为先天性巨结肠时，应进行活检。过去常在齿线上方2～3cm取材，但有人认为取材以在齿线以上1～1 5cm为好，因过高部位的取材可能遗漏“超短段巨结肠”。

一般对于有便秘症状的病人来说，若他以便秘为主诉，则在诊断上应解决三个层次的问题。第一层次是症状诊断。即病人主诉是否符合便秘的定义，亦即自然便次减少或排出困难、伴有不适症状，只有符合定义才能认定其有便秘症状。第二层次是功能诊断。即通过肠道转运功能检查，将其分为肠道正常转运型(全肠道通过时间≤3天)，或肠道慢转运型(全肠道通过时间>3天)。正常转运型主要表现为出口型便秘，通过肛肠动力学、盆底电生理、排粪造影检查，可发现内括约肌、外括约肌(亦包括耻骨直肠肌、提肛肌)、直肠、内生殖器官、泌尿器官的异常。第三层次是病因诊断。即按照病因分类表逐步排除，确定最主要的病因。

便秘的特殊检查及参考值：①结肠运输试验：受试者自检查前3天起禁服泻剂及其他影响肠功能的药物。检查日服含有20粒标记物胶囊二粒，每隔24小时摄腹部平片1张。正常者在72小时内应排出80%标记物。②排粪造影：正常：肛直角力排较静息时增大，应≥90度，提肛时最小。肛上距力排≥静息，但肛上距必须≤30mm(经产妇<35mm)。乙耻距、小耻距均为负值。骶直间距≤10mm、或20mm左右且均匀者。钡剂排出顺畅，且未发现异常。③肛肠压力测定：左侧卧位，测压前不做肛门指诊。首先将球囊或探头置于肛管内，测量肛管静息压和最大缩窄压，然后将球囊送入直肠壶腹测直肠静息压，导管接拖动装置测括约肌功能长度。换双囊导管，大囊置于直肠壶腹部，小囊或探头置于肛管部，向大囊内快速充气50～100ml。正常为肛管压力下降且时程大于30秒，为肛管直肠抑制反射阳性。④直肠感觉功能及顺应性测定：最大耐受容量减直肠感觉阈值为容积变化(v)，最大耐受容量时压力减直肠感觉阈值压力为压力变化(P)，V/P即为直肠顺应性。⑤球囊逼出试验：将球囊置于直肠壶腹部，注入温水50ml，嘱受试者取习惯排便姿势，尽快将球囊排出。正常在5分钟内排出。⑥盆底肌电图检查：将针电极分别穿刺至耻骨直肠肌、外括约肌深部或浅部，记录受试者静息、轻度收缩、用力收缩及排便动作时的肌电活动。分析波形、波幅、频率的变化。

对中年以上的病人，排便习惯一向规律，逐渐发生顽固性便秘时，则必须给以及时和彻底地检查，以便除外结肠癌。年幼开始就有顽固性便秘时，应想到过长结肠和先天性巨结肠症的可能。

便秘作为症状之一，可见于各种疾病所造成的排便动力的不足。如长期慢性消耗性疾病造成的恶病质、衰弱、营养不良、妊娠、腹水、巨大卵巢囊肿的压迫、慢性肺气肿、历肌麻痹等常可引起腹肌、厢肌、提肛肌以及平滑肌的无力，都有可能引起便秘。脊髓及马尾部损伤常造成排便反射障碍。肛裂、痔、肛周的炎症等引起肛门括约肌的痉挛以及肛门短暂性狭窄等，均可引起便秘。至于铅、砷、汞、磷等中毒，碳酸钙、氢氧化铝、阿托品、鸦片等药物的使用，各种原因造成的肠腔狭窄等情况，虽然都可发生便秘，但它常掩盖不了原发病的主要表现，因此与功能性便秘作鉴别常无困难。

## 疾病治疗

播报

功能性便秘的治疗宜采取综合措施和整体治疗，以改善或恢复正常的排便，达到缓解各种症状的目的。在整体治疗和起效时间已不符合现代治疗的要求，同时还应考虑治疗药物能否长期使用，安全性如何以及能否预期患者对药物具有良好的耐受性。

根本的治疗在于去除病因。对于功能性便秘者，应建立合理的饮食和生活习惯。纠正不良习惯、调整饮食内容，增加富含纤维素的蔬菜和水果，适当摄取粗糙而多渣的杂粮，如标准粉、薯类、玉米、大麦米等。油脂类的食物、凉开水、蜂蜜均有助于便秘的预防和治疗。

合理安排工作和生活，做到劳逸结合。适当的文体活动，特别是腹肌的锻炼有利于胃肠功能的改善。对于长期脑力劳动，久坐办公室少活动者更为有益。

养成良好的排便运动习惯。建立每日按时排便的习惯，使直肠的排便运动产生条件反射。对那些有神经衰弱的患者，可适当服用安慰剂调节植物神经中枢的功能。对有肛裂、肛周感染、子宫附件炎的患者，应及时给予治疗，消除其以反射方式影响排便，造成便秘。

金灿荣
详解2022年俄乌冲突
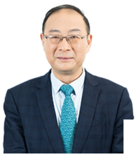

应针对便秘的病因和发病机理进行治疗，对不存在器质性病变的便秘患者，保守治疗的原则是：①增加摄取膳食纤维；②养成定时排便习惯；③避免用使泻药；④治疗个别化。

## 饮食治疗

食疗膳食纤维能改变粪便性质和排便习性，纤维本身不被吸收，能使粪便膨胀，刺激结肠动力。这对于膳食纤维摄取少的便秘患者，可能更有效。肠梗阻或巨结肠以及神经性便秘患者，则不能用增加膳食纤维来达到通便的目的，应减少肠内容物，并定期排便。

饮食宜选用含粗纤维丰富的蔬菜和水果及富含B族维生素的食物，如粗粮、豆类等。可选用芝麻、蜂蜜、松子、杏仁、山萸、核桃仁、竹笋、土豆、萝卜、香蕉、银耳、花生、玉米、菠菜、蕹菜、芹菜、麦麸、荞麦、葵花子、植物油、无花果、荸荠等食物及桑椹子、决明子、生首乌、当归、火麻仁、郁李仁、肉苁蓉等药食兼用之品。忌食酒、烟、浓茶、咖啡、大蒜、辣椒等刺激性食物。在食物调治方面可选择以下几例食疗方：

**1** 芝麻桃仁白糖粉黑芝麻500g，核桃仁250g，绵白糖100g。先将黑芝麻、核桃仁去除杂质，晒干，炒熟，研成细末，调入绵白糖，拌匀，装入瓶罐内，备用。2次/d，15d/次，或早晚各嚼食15g。本食疗方适用于各型功能性便秘。

**2** 柏子仁炖猪心柏子仁20g，猪心1个(约500g)。先将猪心放入清水中浸泡片刻，洗净，切成薄片。将柏子仁洗净盛入碗中。砂锅中加清水适量，置火上，加猪心片，大火煮沸，烹入料酒，加葱花、姜片及柏子仁，改用小火煨炖1h，待猪心熟烂，停火，加精盐、味精、五香粉各少许，拌和均匀即成。佐餐当菜。本食疗方适用于血虚便秘。

**3** 三仁粥柏子仁20g，松子仁15g，郁李仁20g，粳米100g。先将郁李仁打碎，入锅，加水煎煮20min，去渣取汁。将柏子仁、松子仁敲碎，除去外衣，与淘净的粳米同入砂锅，加水适量，先用大火煮沸，缓缓加入郁李仁煎汁，改用小火煨煮成稠粥，即成。早晚2次分服。本食疗方适合各型功能性便秘。

**4** 黄芪火麻仁蜂蜜饮蜜炙黄芪20g，火麻仁10g，蜂蜜15g。先将生火麻仁打碎，与蜜炙黄芪同入锅中，加水煎煮30min，去渣，取浓汁，趁温热加入蜂蜜，调匀即成。每日早晨空腹顿服。本食疗方对气虚型便秘尤为适宜。

**5** 番泻叶决明子茶番泻叶3g，决明子30g。将番泻叶，决明子同放入有盖杯中，用沸水冲泡，加盖，闷15min即可饮用。当茶，频频饮用，一般可冲泡2次。本食疗方对热积型便秘尤为适宜。[1]

## 养成定时排便习惯

定时排便能防止粪便堆积，这对于粪便嵌塞的患者尤其重要。注意在训练前，宜先清肠，可用生理盐水清洁清肠，每日2次，共3日。清肠后摄腹部平片，确定肠内已无粪便嵌塞。近年来、也有报道口服电解质平衡液，可达到清肠目的。清肠后可给轻泻剂，便便次至少达到1次/日。并鼓励患者早餐后解便，如仍不排便，还可鼓励晚餐后再次解便。使患者恢复正常排便习惯。一旦餐后排便有规律地发生，且维持2～3个月以上，可渐停用泻药。如在过程中有2～3日不解便，仍要清肠，以免再次发生粪便嵌塞。这种通过清肠，服用轻泻剂并训练排便习惯的方法，常用于治疗功能性便秘、其成功率可达到70～80%，但有不少复发。对于直肠括约肌功能紊乱的便秘患者，可应用生物反馈（Biofeedback）来纠正排便时盆底肌和肛门外扩约肌的不合适的收缩，在儿童和成人的功能性便秘中已获成功的例子．但对精神抑郁的便秘患者，疗效较差。

## 药物治疗

通过上述方法达不到疗效时可考虑药物治疗，对于STC患者，首选是促动力剂，西沙必利作为一种全胃肠道促动力剂，对某些STC患者有效。一种新型特异性促肠动力药普卡必利晚近已问世，该药系苯并呋喃族化合物，特异性作用于5-HT4受体，可望成为一种理想的治疗功能性便秘的药物。常用泻剂有：①容量性泻药：硫酸镁、硫酸钠、甲基纤维素、琼脂等；②刺激性泻剂：番泻叶、蓖麻油、双酯酚汀等；③粪便软化剂：液体石蜡、乳果糖等；④直肠内给药：甘油栓、开塞露等。应避免长期滥用泻剂而导致泻剂性肠病。

**1.** 容积性泻药（纤维素）能加速结肠或全肠道转运，吸附水分，使大便松软易派出，缓解便秘及排便紧迫感；果胶、车前草、燕麦麸等可容性纤维有助于保持粪便水分；而植物纤维素、木质素等不可溶纤维素可增加大便量。

纤维素制剂的优点在于其经济、安全、适用于各级医疗机构；但摄入纤维素制剂较多时会发生胃肠胀气，对于结肠乏力的患者应该慎用。

补充纤维素后并不能立即显效，应用7-10天后根据具体情况适当加减用量。

**2.** 盐类泻剂（硫酸镁）口服硫酸镁在肠道内不易吸收，留在肠腔内形成高渗状态，导泻作用强且迅速，一般口服2-6小时后即可排出水样或半流体粪便。可引起严重不良反应，临床上应慎用。目前通常用于全结肠镜或钡剂灌肠等检查前的肠道准备工作。

**3.** 刺激性腹泻（蕃泻叶、鼠李、酚酞、篦麻油等）长期使用刺激性泻剂可损害患者的肠神经系统，而且很可能是不可逆的。

酚酞：口服后在肠内形成可溶性钠盐，刺激结肠粘膜促进蠕动；并阻止肠液被肠壁吸收而其导泻作用。一般用药后4-8小时可排出半流动性软便，导泻与肠腔内液体酸度有关。对阑尾炎、肠出血、心肾功能不全，高血压、肠梗阻及婴幼儿、孕妇禁用。临床应用每次1-4片，临睡前服用。对全结肠镜检查前、X线检查或术前作肠道准备者，应提前8小时服用。

比沙可啶：口服后经肠内细菌分解的产物及药物本身对壁均有较强的刺激作用，能增加肠蠕动，促进解便；同时可抑制结肠内NA+、CA2+及水分的吸收，从而使肠腔内容积增大，引起反射性排便。临床上对急，慢性便秘有效率较高。还可用于分娩前、手术前，腹部X线检查或内镜检查前的肠道排空，手术后，产后恢复正常的排便习惯。服用后可引起腹痛，偶可发生剧烈的腹部痉挛。急腹症、痉挛性便秘、重症硬结便、肛门破裂或痔疮溃疡患者禁用，孕妇慎用。

秒懂百科
金灿荣
详解2022年俄乌冲突
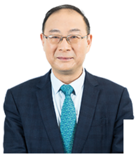

**4**、渗透性泻剂（聚乙二醇4000）、乳果糖等

乳果糖：是人工合成双糖，在胃及小肠内不被分解和吸收，到达结肠后，通过渗透作用使水和电解质保留于肠腔内；并被肠道正常菌群分解为乳酸和乙酸等，并进一步提高腔内渗透压，产生导泻作用；阻断氨的吸收；其酸性代谢产物能刺激肠黏膜，增加肠蠕动，促进排便。由于乳果糖在体内分解产生气体，故部分患者会有腹胀、排气增多等胃肠胀气表现。用量过大会产生恶心、腹胀、腹泻和低钾血症、高钠血症等。禁用于胃肠道阻塞、糖尿病或低糖饮食者。慢性便秘患者治疗剂量为每天1-2次，每次5-10g，及俩美好以每日保持2-3次软便为宜。临床用于慢性功能性便秘，包括老人、儿童、婴儿和孕妇各个年龄组的患者，安全性高。对于肝性脑病患者，应用乳果糖后，不仅具有保持大便通畅的作用，还可减少氨的吸收，有利于肝性脑病的恢复。

**5**.促动力药（西沙必利）是临床上广泛应用的胃肠道促动力药，属于苯二氮卓类药物，其促动力效应直接作用于上段结肠。它曾用于便秘的治疗，但疗效并不肯定。对于结肠乏力即STC患者，选用促动剂改善肠神经和特异选择性作用于结肠平滑肌的促动力药，如5—HT4受体激动剂，西沙必利，普卡必利(procaloprid)，以及5—HT4部分激动剂，特异作用结肠的替加色罗(tegaserod)等，后者多用于CIBS。此外，米索前列醇(misoprost01)，阿片类拮抗剂纳络酮(naloxone)也可改善某些患者的便秘症状，但对功能和梗阻型便秘的排便功能，尚未能证实其确切疗效。

6.润滑性泻剂（开塞露 液状石蜡）

开塞露（含硫酸镁、甘油、丙二醇）：能润滑并刺激肠壁，软化大便，使其易于排出，成人20ml/次，主要适用于硬结便患者，尤其是老年症患者。

液状石蜡：在肠道内不被吸收或消化，润滑肠壁，使粪便易于排出。对年老体弱、长期卧床的便秘患者使用是应注意其有引起脂质性吸入性肺炎的可能，长期服用可致脂溶性维生素缺乏。成人15-30ml/次，用药后6-8小时产生效果，一般于睡前服用。

**7**.微生态制剂:含有双歧杆菌、乳酸杆菌、肠球菌等肠道正常菌群。是一种良好的微生态调节制剂，直接补充正常生理性菌群，改善肠道微生态环境。但应避免与抗生素合用。

调节肠道微生态的制剂还有米雅BM、丽珠肠乐等。可作为便秘的辅助治疗。

**8**.中药泻剂 就中医而言，便秘分为实秘、虚秘。热秘以清热润肠为主，可服麻仁丸；气秘应理气导滞，以苏子降气汤加味。虚秘又分气虚，以益气润肠为主，用补中益气汤加减；血虚则宜养血润燥，四物汤可用；寒凝则应温通开秘，以温脾汤加味。

临床上常用的中药制剂应注意，制剂中大都含有大黄、芦荟等刺激性泻剂成分的药物，故不主张长时间的应用。

简言之，在慢性便秘治疗中，选用不恰当的泻剂或泻剂应用剂量不合理等，均可能引起患者脱水、电解质平衡紊乱等到不良反应。对有高血压、心脏病、糖尿病、肾功能不全合并便秘的患者，应选用安全的通便药物，如聚乙二醇4000。

## 外科治疗

当应用轻泻药、纤维和促动力药进行的积极的、延长疗程的结肠惰性治疗失败时，其治疗应是全结肠切除伴回-直肠吻合术。应告诉患者，该手术是设计用来治疗便秘症状(排便困难或频率稀少)。其他症状(腹痛和腹胀)可能不会缓解。结肠切除到骶骨岬水平，在末端回肠和直肠上端之间进行吻合。进入骶前区时需仔细保留交感神经。

回-直肠吻合较回肠-乙状结肠吻合更为成功。如果任何部位留下乙状结肠，便秘可能复发，相反，吻合口低于距肛门边缘7～10厘米水平可能导致无法接受的高排便频率，有时甚至大便失禁。回-直肠吻合术后仍持续便秘的患者可能有盆腔底功能异常。

**1、排空异常的外科治疗**

切断耻骨直肠肌的后纤维被认为可能对排便时该肌肉呈矛盾收缩的患者有益。然而并非如此，不论是切断耻骨直肠肌的后部或侧面都令人失望。将耻骨直肠肌肉纤维在中线任何一边切断，7名患者中仅1人症状改善，而将侧面肌肉切断在15名患者中仅3人症状改善。

**2、会阴下降综合征**

会阴下降综合征患者也会发生便秘，这种患者排便时无止境地摒力但直肠不能完全排空。可以观察到会阴明显鼓出坐骨结节平面，这种会阴异常下降可能继发于分娩，或是排便时长时间摒力造成骶神经损伤。不完全排空导致更摒力，对神经的牵拉更强，以及肛门外括约肌和耻骨直肠肌的进行性去神经支配。这种情况会造成大便失禁，因而增加患者的痛苦。手术不能纠正该问题。最佳的治疗方法是生物反馈，尽管成功率只有50%。

**3、造口术**

患者有时因便秘而要求作造口。造口是个好的选择，因其能回复。再次，仔细选择患者极为重要。结肠造口容许作结肠冲洗的可能性，但一些作者报道因造口近端的持续结肠惰性或更全面的动力紊乱，导致效果不满意。

最近描述的一种称为"自制结肠导管"的手术可能是对某些患者的解决方法。通过在中点横断乙状结肠，将之用作为自制结肠导管。该手术成功地降低患者的排便时间，增加排便次数。该手术是可逆的，但复杂。

因此，在许多诉有便秘的患者中只有一小部分将从手术中得益，可能是占经高度选择的转诊病人的5%。

## 生物反馈

生物反馈治疗的实质是利用声音和影像的反馈，刺激训练病人正确地控制肛门外括约肌的舒缩，达到正常排便。生物反馈治疗法是一种纠正不协调排便行为的训练法，主要用于治疗肛门括约肌失协调和盆底肌、肛门外括约肌排便时矛盾性收缩导致的FOOC，有人报告其疗效可达96%，该法与药物治疗相比具有无药物副作用、成本低、非创伤性等优点，目前国内已开展此项疗法。生物反馈疗法对功能性便秘有确定的疗效，无副作用，治疗费用低。Faliakou等报道对100例功能性便秘病人65%为结肠慢

秒懂百科
金灿荣
详解2022年俄乌冲突
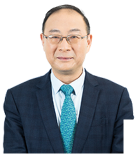

传输，59%为反常性盆底肌痉挛）历时4年的研究结果显示，生物反馈疗法对慢传输型、出口梗阻型、混合型便秘病人均有效。Glia等对26例功能性便秘病人10例为结肠慢传输，16 例为反常性盆底肌痉挛进行生物反馈治疗，6个月的随访结果表明，生物反馈疗法对出口梗阻型便秘病人有较好疗效。

**1、生物反馈疗法的具体步骤**

生物反馈疗法强调动员病人大脑的调控功能，强调医生与病人之间良好的沟通，这一思想贯穿生物反馈疗法的各个步骤。

首先，在治疗前，要向病人详细讲解人体结肠、直肠、肛门和盆底肌的正常解剖和生理功能，讲解正常排便的机制；还要向病人讲解清楚生物反馈治疗的机理和目的以及生物反馈仪器的使用。将治疗仪与病人连接好后，安排病人坐或躺在治疗仪和治疗师的右侧，面对治疗仪和治疗师。向病人讲解清楚仪器上所显示的曲线的意义，并指出病人在静息、屏气和用力排便时的异常所在。耐心告诉病人如何调控括约肌的舒缩，鼓励其尝试，病人的每一次尝试都会在仪器上显示，一旦有正确的活动，仪器便会以悦耳的声音和动感的图象刺激病人，治疗师亦会给予鼓励。最后，病人在无治疗师帮助的情况下，面对仪器自行练习，直至连续三次正常排便出现为止。

**2、生物反馈疗法的时间安排**

行生物反馈治疗者绝大多数为门诊病人，一般安排病人每周治疗2次，持续5周以上。

**3、生物反馈疗法的几种形式**

**（1）**肌电图介导的生物反馈方式（EMG - basedbiofeedbackmethod）是目前最为常用的生物反馈方式。有两种系统较为常用：带有温度和呼吸传感器的大型治疗系统（SRS Orion

PC - 12）和便携式家用小型治疗系统（U - ControlT“EMGHome Trainer），为CTD - SYSNETICS公司生产。

**（2）**压力测定介导的生物反馈方式（manometrybasedbiofeedback method）其机理为使用肛门括约肌探头进行括约肌压力测定，通过压力变化行生物反馈治疗。

**（3）**其他生物反馈方式 Fleshman等发明了一种可以上下摆动，同时也可以发出声音信号的光棒来训练病人。首先，插人直肠带电极的塞子，记录静息、屏息及用力排塞时的肌肉活动，然后指导病人控制肌肉的活动。

超短波、短波、水疗、矿泉水浴、按摩等理疗方法作为辅助治疗可有帮助。

## 高电位治疗

临床上无理想治疗方法，目前广泛采用的常规导泻剂虽然有效，但均有不同程度的副作用，如干扰肠道正常活动和吸收，降低肠壁感受细胞的应激性等，还可造成病人对药物依赖性，长期使用可造成便秘的恶性循环。而高电位治疗器治疗功能性便秘避免了以往治法的弊端，完全突破了以泻治秘的常规疗法，取得满意效果，高于常规导泻方法。在总便次数、软便次数的增加及无便日、硬便次数、排便时间减少的五项指标上，无论是治疗期，还是停疗期均较常规导泻法有显著性差异(P＜0．0１)。从高电位的角度探讨其治疗机理，包括以下四方面：(1 )刺激作用(振动效果)：高电位的正负相位变化，即是刺激，它对活跃细胞，调节神经机能等都有影响；(2 )电离作用：施加高电位因电离的作用，膜的通透性增加，提高了失神经肌纤维膜对钾的通透性；(3 )植物神经的调节作用：电位负荷可以减轻副交感神经的紧张和调节植物神经的功能。(4)水束分解作用：在人体内起着运输营养、氧、排泄废物的水，负荷高电位后活动加剧。上述四方面的作用最终达到调节肠道的功能，加强肠管节率性推进，促进肠蠕动而排便，且以软便为主。另外，高电位治疗器治疗时，仅个别患者有发热感、疲乏感，在降低电压，缩短治疗时间后消失。 [2-3]

## 参考资料


1 中华中医药学会。 便秘诊疗指南。 中国中医药现代远程教育，2011年09卷 第17期

2 周晓娜，刘培茹，张莉。 生物反馈治疗老年慢性功能性便秘的肛门直肠动力学改变。 中国老年学杂志，2008年28卷 第03期

3 李延青，于岩波 .功能性便秘的诊断与治疗 .中国实用内科杂志 2011年31卷02期



搜索发现

老人便秘怎么回事 便秘的主要原因 便秘会有什么症状 经常便秘怎么办 长期便秘的解决方法

为什么老是便秘 功能行便秘 功能性便秘如何治 预防便秘的方法有哪些 便秘并发症

新手上路 成长任务 编辑入门 编辑规则 本人编辑 NEW

我有疑问 内容质疑 在线客服 官方贴吧 意见反馈

投诉建议 举报不良信息 未通过词条申诉 投诉侵权信息 封禁查询与解封

金灿荣
详解2022年俄乌冲突


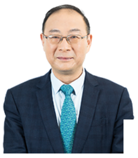


©2022 Baidu 使用百度前必读 | 百科协议 | 隐私政策 | 百度百科合作平台 | 京ICP证030173号

京公网安备11000002000001号
