# Supplementary material for: Assessment of medical information on irritable bowel syndrome information in Wikipedia and Baidu Encyclopedia: comparative study
Source: PeerJ. 2024 May 24;12:e17264. doi: 10.7717/peerj.17264 (PMC11129691; doi:10.7717/peerj.17264)
Supplement: Data S1 [file peerj-12-17264-s001.zip › σÄƒσoïμò░μì«/Baidu/Baidu-Chinese/5-μ┐ÇΦ║üμÇoσñoΦéáτùçσÇÖτ╛ñ_τÖ╛σ║aτÖ╛τoæ.docx]

| 2022/12/14 10:41 | 激躁性大肠症候群_百度百科  [**网页**](https://www.baidu.com/) [**新闻**](http://news.baidu.com/) [**贴吧**](https://tieba.baidu.com/) [**知道**](https://zhidao.baidu.com/) [**网盘**](https://pan.baidu.com/?from=1027327l) [**图片**](http://image.baidu.com/) | [**视频**](http://v.baidu.com/) | [**地图**](http://map.baidu.com/) | [**文库**](https://wenku.baidu.com/) | **百科** | 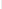 [百度首页](http://www.baidu.com/) [登录](javascript:;) |
| --- | --- | --- | --- | --- | --- | --- |

| [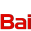岔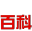](https://baike.baidu.com/) | \| 激躁性大肠症候群 \| 进入词条 \| \| --- \| --- \| | \| 全站搜索 \| \| --- \| | [帮助](https://baike.baidu.com/help) |
| --- | --- | --- | --- | --- | --- | --- |
| 近期有不法分子冒充百度百科官方人员，以删除词条为由威胁并敲诈相关企业。在此严正声明：百度百科是免费编辑平台，绝不存在收费代编服务，请勿上当受骗！ [详情>>](https://baike.baidu.com/common/declaration) | | | |
| [首页](https://baike.baidu.com/) 秒懂百科 特色百科 用户 知识专题 权威合作 [口下载百科APP](https://baike.baidu.com/wapui/subpage/baikeappdownload?sfrom=pc_lemmapage_navigation) 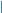 [2 个](https://baike.baidu.com/usercenter) | | | |

|  | [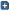](javascript:;)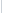 . 收藏 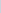[山 158](javascript:void(0);)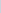 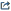  **性** **类**  **质** **别**  激躁性大肠症候群 [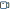上传视频](javascript:;)   \| 小播报 \| \| --- \|  \| 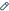编辑 \| \| --- \|  \| O讨论 \| \| --- \|   肠胃道疾病  [激燥性大肠症](https://baike.baidu.com/item/%E6%BF%80%E7%87%A5%E6%80%A7%E5%A4%A7%E8%82%A0%E7%97%87/1506814?fromModule=lemma_inlink)(Irritable Bowel Syndrome) 是临床上最常见到的肠胃道疾病，虽然该病不是致命的疾病，但病人常有很大的痛 苦，并且医师在治疗时有爱莫能助和灰心丧气的感觉。  激燥性大肠症常见于青年或中年人，女/男 比例1： 2。主要表现是[慢性便秘](https://baike.baidu.com/item/%E6%85%A2%E6%80%A7%E4%BE%BF%E7%A7%98/10430220?fromModule=lemma_inlink)，间歇性腹泻或两者均有。  激躁性大肠症候群  医学  疾病  **中文名**  **外文名**  Irritable Bowel Syndrome | 13 | 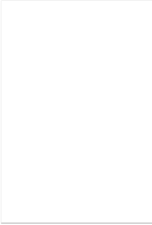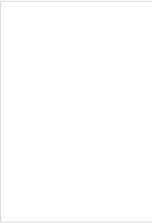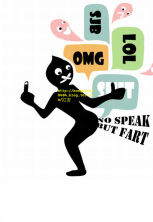 [激躁性大肠症候群的概述 张)](https://baike.baidu.com/pic/%E6%BF%80%E8%BA%81%E6%80%A7%E5%A4%A7%E8%82%A0%E7%97%87%E5%80%99%E7%BE%A4/4549268/1/0b55b319ebc4b74570b842efc5fc1e178a82151e?fr=lemma&fromModule=lemma_top-image&ct=single) |
| --- | --- | --- | --- | --- | --- | --- |
|  | \| 目录 \| 3 [病理生理](#_bookmark2)  1 [疾病类型](#_bookmark1) 2 [临床表现](#_bookmark4)  5 [治疗方式](#_bookmark5) 6 [预防保养](#_bookmark6)  4 [诊断要点](#_bookmark3) \| \| --- \| --- \| |  |  |
|  |  |  | [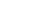](https://cpro.baidu.com/cpro/ui/uijs.php?en=mywWUA71T1YsFh7sT7qGujYsFhPC5H0huAbqrauGTdq9TZ0qnauJp1YvrjI-mhcLuhwhujb4nyRsFh_qFRcdFRRLFRn4FRPjFRnvFRFDFRPjFRD3FRfYFRmdFRn1FRcYFRfLFRmvFhkdpvbqnHchUyPsUHY3njTLrauk5HnLnjbYnHfsgvPsTBuzmWYsFMF15HDhTvN_UANzgv-b5HDhTv-b5yRsrH7brywhPvuBmWI-PW6hTLwGujY3FMfqIZKWUA-WpvNbndqCmzuYujYkrHbLPWn1FMwV5Hcvrj6sn1R3niuYUgnqnHmLnjb3PHmsPBuYIHddnHb4P1m1nzud5y9YIZK1FHPKFHFAFHFAmh7GpvR-nbNBmy-bIiRzwyPEUiuv5HchpHY4PhR1uAcLm6&besl=6&c=news&cf=1&cvrq=3247861&eid_list=201577_202016_204226_204854_205526_207574_209357&expid=201577_202016_202257_202564_204854_205526_205809_207574_209394&fr=20&fv=0&haacp=3601&img_typ=0&itm=0&lu_idc=gzhxy&lukid=12&lus=e091d9df7fbb7e68&lust=63993786&luwtr=7777852140969087504&mscf=0&n=10&nttp=1&p=baidu&pbs=220093&sce=7&sr=72&ssp2=1&tpl=baiduCustITagLinkUnitRankCol&tsf=dtp:1&tu_type=0&u=%2Fitem%2F%25E6%25BF%2580%25E8%25BA%2581%25E6%2580%25A7%25E5%25A4%25A7%25E8%2582%25A0%25E7%2597%2587%25E5%2580%2599%25E7%25BE%25A4%2F4549268%3FfromModule%3Dsearch%2Dresult%5Flemma&uicf=lurecv&urlid=0&eot=1)[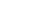](https://cpro.baidu.com/cpro/ui/uijs.php?en=mywWUA71T1YsFh7sT7qGujYsFhPC5H0huAbqrauGTdq9TZ0qnauJp1YvrjI-mhcLuhwhujb4nyRsFh_qFRfsFRR4FRnYFRRzFRFaFRmdFRckFRfzFRnvFRFDFRPjFRD3FhkdpvbqnHfhUyPsUHYLrjmdPauk5HnLnjbYnHfsgvPsTBuzmWYsFMF15HDhTvN_UANzgv-b5HDhTv-b5yRsrH7brywhPvuBmWI-PW6hTLwGujY3FMfqIZKWUA-WpvNbndqCmzuYujYkrHbLPWn1FMwV5Hcvrj6sn1R3niuYUgnqnHmLnjb3PHmsPBuYIHddnHb4P1m1nzud5y9YIZK1FHPKFHFAFHFAmh7GpvR-nbNBmy-bIiRzwyPEUiuv5HchpHYvmvnvryuhmf&besl=6&c=news&cf=1&cvrq=3011782&eid_list=201577_202016_204226_204854_205526_207574_209357&expid=201577_202016_202257_202564_204854_205526_205809_207574_209394&fr=20&fv=0&haacp=194&img_typ=0&itm=0&lu_idc=gzhxy&lukid=14&lus=e091d9df7fbb7e68&lust=63993786&luwtr=2314272910775226794&mscf=0&n=10&nttp=1&p=baidu&pbs=220093&sce=7&sr=72&ssp2=1&tpl=baiduCustITagLinkUnitRankCol&tsf=dtp:1&tu_type=0&u=%2Fitem%2F%25E6%25BF%2580%25E8%25BA%2581%25E6%2580%25A7%25E5%25A4%25A7%25E8%2582%25A0%25E7%2597%2587%25E5%2580%2599%25E7%25BE%25A4%2F4549268%3FfromModule%3Dsearch%2Dresult%5Flemma&uicf=lurecv&urlid=0&eot=1)[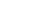](https://cpro.baidu.com/cpro/ui/uijs.php?en=mywWUA71T1YsFh7sT7qGujYsFhPC5H0huAbqrauGTdq9TZ0qnauJp1YvrjI-mhcLuhwhujb4nyRsFh_qFRPDFRFjFRPKFRR4FRndFRuKFRcLFRDzFRPDFRm3FhkdpvbqnHRhUyPsUHYLrHfzPiuk5HnLnjbYnHfsgvPsTBuzmWYsFMF15HDhTvN_UANzgv-b5HDhTv-b5yRsrH7brywhPvuBmWI-PW6hTLwGujY3FMfqIZKWUA-WpvNbndqCmzuYujYkrHbLPWn1FMwV5Hcvrj6sn1R3niuYUgnqnHmLnjb3PHmsPBuYIHddnHb4P1m1nzud5y9YIZK1FHPKFHFAFHFAmh7GpvR-nbNBmy-bIiRzwyPEUiuv5HchpHYLnWT1nhmdnf&besl=6&c=news&cf=1&cvrq=4468518&eid_list=201577_202016_204226_204854_205526_207574_209357&expid=201577_202016_202257_202564_204854_205526_205809_207574_209394&fr=20&fv=0&haacp=317&img_typ=0&itm=0&lu_idc=gzhxy&lukid=15&lus=e091d9df7fbb7e68&lust=63993786&luwtr=639862747281857404&mscf=0&n=10&nttp=1&p=baidu&pbs=220093&sce=7&sr=72&ssp2=1&tpl=baiduCustITagLinkUnitRankCol&tsf=dtp:1&tu_type=0&u=%2Fitem%2F%25E6%25BF%2580%25E8%25BA%2581%25E6%2580%25A7%25E5%25A4%25A7%25E8%2582%25A0%25E7%2597%2587%25E5%2580%2599%25E7%25BE%25A4%2F4549268%3FfromModule%3Dsearch%2Dresult%5Flemma&uicf=lurecv&urlid=0&eot=1)[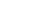](https://cpro.baidu.com/cpro/ui/uijs.php?en=mywWUA71T1YsFh7sT7qGujYsFhPC5H0huAbqrauGTdq9TZ0qnauJp1YvrjI-mhcLuhwhujb4nyRsFh_qFRPDFRm3FRnzFRRLFRc4FRDYFRc1FRPjFRPKFRDvFhkdpvbqnHmhUyPsUHYkn1Dkrj0hTHY1P104PjDYn7qWTZchThcqnauzT1YkFMP-UAk-T-qGujYkFMPGujd-njbkuj-buWIhmhcLuHm3FMPYpyfqrauY5gwsmvkGmvV-ujPxpAnhIAfqnHb4P1m1nzuYUHYzPW63njndrjDhIAd15HDvP104rjRvnjmhIZRqIHD4rHTvn1nhIHdCIZwsTzR1fiRzwBRzwhF9pyV-FHF7mh7GuZR-nbNWUvYhIWYzFhbqnvRLmhf3PH0&besl=6&c=news&cf=1&cvrq=3661677&eid_list=201577_202016_204226_204854_205526_207574_209357&expid=201577_202016_202257_202564_204854_205526_205809_207574_209394&fr=20&fv=0&haacp=1439&img_typ=0&itm=0&lu_idc=gzhxy&lukid=16&lus=e091d9df7fbb7e68&lust=63993786&luwtr=750531414731215287&mscf=0&n=10&nttp=1&p=baidu&pbs=220093&sce=7&sr=72&ssp2=1&tpl=baiduCustITagLinkUnitRankCol&tsf=dtp:1&tu_type=0&u=%2Fitem%2F%25E6%25BF%2580%25E8%25BA%2581%25E6%2580%25A7%25E5%25A4%25A7%25E8%2582%25A0%25E7%2597%2587%25E5%2580%2599%25E7%25BE%25A4%2F4549268%3FfromModule%3Dsearch%2Dresult%5Flemma&uicf=lurecv&urlid=0&eot=1)[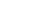](https://cpro.baidu.com/cpro/ui/uijs.php?en=mywWUA71T1YsFh7sT7qGujYsFhPC5H0huAbqrauGTdq9TZ0qnauJp1YvrjI-mhcLuhwhujb4nyRsFh_qFRc1FRmvFRcsFRRvFRn4FRRLFRfLFRfYFRcLFRfkFRc1FRmvFRPKFRR4FhkdpvbqnHThUyPsUHYknH0dn1mhTHY1P104PjDYn7qWTZchThcqnauzT1YkFMP-UAk-T-qGujYkFMPGujd-njbkuj-buWIhmhcLuHm3FMPYpyfqrauY5gwsmvkGmvV-ujPxpAnhIAfqnHb4P1m1nzuYUHYzPW63njndrjDhIAd15HDvP104rjRvnjmhIZRqIHD4rHTvn1nhIHdCIZwsTzR1fiRzwBRzwhF9pyV-FHF7mh7GuZR-nbNWUvYhIWYzFhbqnyPhrjP9Pym&besl=6&c=news&cf=1&cvrq=7189029&eid_list=201577_202016_204226_204854_205526_207574_209357&expid=201577_202016_202257_202564_204854_205526_205809_207574_209394&fr=20&fv=0&haacp=618&img_typ=0&itm=0&lu_idc=gzhxy&lukid=17&lus=e091d9df7fbb7e68&lust=63993786&luwtr=7476068094432150857&mscf=0&n=10&nttp=1&p=baidu&pbs=220093&sce=7&sr=72&ssp2=1&tpl=baiduCustITagLinkUnitRankCol&tsf=dtp:1&tu_type=0&u=%2Fitem%2F%25E6%25BF%2580%25E8%25BA%2581%25E6%2580%25A7%25E5%25A4%25A7%25E8%2582%25A0%25E7%2597%2587%25E5%2580%2599%25E7%25BE%25A4%2F4549268%3FfromModule%3Dsearch%2Dresult%5Flemma&uicf=lurecv&urlid=0&eot=1)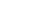   \| 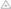**词条统计**  ~~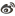~~  浏览次数： 247543次  [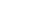](https://baike.baidu.com/historylist/%E6%BF%80%E8%BA%81%E6%80%A7%E5%A4%A7%E8%82%A0%E7%97%87%E5%80%99%E7%BE%A4/4549268)编辑次数： 17次历史版本  [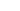](https://baike.baidu.com/usercenter/userpage?uk=TY3CXj_hJSEcBfJBVP43Rg&from=lemma)最近更新： w_ou ( 2021-01-26)  **突出贡献榜**  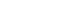qqq582311436  [女](javascript:void(0);) \| \| \| \| --- \| --- \| --- \| \| **1** \| [购买域名](https://cpro.baidu.com/cpro/ui/uijs.php?en=mywWUA71T1YsFh7sT7qGujYsFhPC5H0huAbqrauGTdq9TZ0qnauJp1YvrjI-mhcLuhwhujb4nyRsFh_qFRc4FRFKFRnzFRmzFRf1FRmzFRn1FRuaFhkdpvbqniuVmLKV5HbYnW0LFMDqn1TsrHfkPjKxmLKzFMFB5H0hTMnqniu1uyk_ugFxpyfqniu1pyfquH04nyf4uAmLuhFBPvRvrau1IA-b5H6hIjdYTAP_pyPouyf1gv9WFMwb5HD4rHTvn1nhIAYqnWm3rj01PH6kFMwVT1YkPWTsrH6dPW0vFMwd5gRkrHbLPWn1FMRqpZwYTZn-nYD-nbm-nbuBmy-ouiRzwyF9pywdFHF7mvqVFMmqnBuG5yn3m1FBnAPh&besl=6&c=news&cf=1&cvrq=1941531&eid_list=201577_202016_204226_204854_205526_207574_209357&expid=201577_202016_202257_202564_204854_205526_205809_207574_209394&fr=20&fv=0&haacp=1286&img_typ=0&itm=0&lu_idc=gzhxy&lukid=1&lus=e091d9df7fbb7e68&lust=63993786&luwtr=17492749210616066771&mscf=0&n=10&nttp=1&p=baidu&pbs=220093&sce=7&sr=72&ssp2=1&tpl=baiduCustITagLinkUnitRankCol&tsf=dtp:1&tu_type=0&u=%2Fitem%2F%25E6%25BF%2580%25E8%25BA%2581%25E6%2580%25A7%25E5%25A4%25A7%25E8%2582%25A0%25E7%2597%2587%25E5%2580%2599%25E7%25BE%25A4%2F4549268%3FfromModule%3Dsearch%2Dresult%5Flemma&uicf=lurecv&urlid=0&eot=1) \| **12** 电商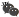台怎 \| \| **2** \| [37游戏平台](https://cpro.baidu.com/cpro/ui/uijs.php?en=mywWUA71T1YsFh7sT7qGujYsFhPC5H0huAbqrauGTdq9TZ0qnauJp1YvrjI-mhcLuhwhujb4nyRsFh_qn1T-wjn-fYR-fYm-fWT-f1m-fbf-fYn-fH6hUZNopHYzFhdWTAYqrHc1nj0hTHY1P104PjDYn7qWTZchThcqnauzT1YkFMP-UAk-T-qGujYkFMPGujd-njbkuj-buWIhmhcLuHm3FMPYpyfqrauY5gwsmvkGmvV-ujPxpAnhIAfqnHb4P1m1nzuYUHYzPW63njndrjDhIAd15HDvP104rjRvnjmhIZRqIHD4rHTvn1nhIHdCIZwsTzR1fiRzwBRzwhF9pyV-FHF7mh7GuZR-nbNWUvYhIWYzFhbqmHfzuWTzrjn&besl=6&c=news&cf=1&cvrq=3623810&eid_list=201577_202016_204226_204854_205526_207574_209357&expid=201577_202016_202257_202564_204854_205526_205809_207574_209394&fr=20&fv=0&haacp=188&img_typ=0&itm=0&lu_idc=gzhxy&lukid=2&lus=e091d9df7fbb7e68&lust=63993786&luwtr=676781759077005289&mscf=0&n=10&nttp=1&p=baidu&pbs=220093&sce=7&sr=72&ssp2=1&tpl=baiduCustITagLinkUnitRankCol&tsf=dtp:1&tu_type=0&u=%2Fitem%2F%25E6%25BF%2580%25E8%25BA%2581%25E6%2580%25A7%25E5%25A4%25A7%25E8%2582%25A0%25E7%2597%2587%25E5%2580%2599%25E7%25BE%25A4%2F4549268%3FfromModule%3Dsearch%2Dresult%5Flemma&uicf=lurecv&urlid=0&eot=1) \| [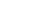](https://cpro.baidu.com/cpro/ui/uijs.php?en=mywWUA71T1YsFh7sT7qGujYsFhPC5H0huAbqrauGTdq9TZ0qnauJp1YvrjI-mhcLuhwhujb4nyRsFh_qTvPGFRnzFRwaFRP7FRnYFRPDFRcvFRc3FRRdFhkdpvbqnHnhUyPsUHYknW6zrjDhTHY1P104PjDYn7qWTZchThcqnauzT1YkFMP-UAk-T-qGujYkFMPGujd-njbkuj-buWIhmhcLuHm3FMPYpyfqrauY5gwsmvkGmvV-ujPxpAnhIAfqnHb4P1m1nzuYUHYzPW63njndrjDhIAd15HDvP104rjRvnjmhIZRqIHD4rHTvn1nhIHdCIZwsTzR1fiRzwBRzwhF9pyV-FHF7mh7GuZR-nbNWUvYhIWYzFhbqPADvmHmdnvD&besl=6&c=news&cf=1&cvrq=3196467&eid_list=201577_202016_204226_204854_205526_207574_209357&expid=201577_202016_202257_202564_204854_205526_205809_207574_209394&fr=20&fv=0&haacp=1032&img_typ=0&itm=0&lu_idc=gzhxy&lukid=13&lus=e091d9df7fbb7e68&lust=63993786&luwtr=2480674890665447709&mscf=0&n=10&nttp=1&p=baidu&pbs=220093&sce=7&sr=72&ssp2=1&tpl=baiduCustITagLinkUnitRankCol&tsf=dtp:1&tu_type=0&u=%2Fitem%2F%25E6%25BF%2580%25E8%25BA%2581%25E6%2580%25A7%25E5%25A4%25A7%25E8%2582%25A0%25E7%2597%2587%25E5%2580%2599%25E7%25BE%25A4%2F4549268%3FfromModule%3Dsearch%2Dresult%5Flemma&uicf=lurecv&urlid=0&eot=1)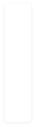**13** sci论文投稿 \| \| **3**  **4**  **5** \| [国际期货](https://cpro.baidu.com/cpro/ui/uijs.php?en=mywWUA71T1YsFh7sT7qGujYsFhPC5H0huAbqrauGTdq9TZ0qnauJp1YvrjI-mhcLuhwhujb4nyRsFh_qFRc4FRuKFRFjFRPKFRnvFRwKFRFaFRmdFhkdpvbqnzuVmLKV5HDsrjb1Pzuk5HnLnjbYnHfsgvPsTBuzmWYsFMF15HDhTvN_UANzgv-b5HDhTv-b5yRsrH7brywhPvuBmWI-PW6hTLwGujY3FMfqIZKWUA-WpvNbndqCmzuYujYkrHbLPWn1FMwV5Hcvrj6sn1R3niuYUgnqnHmLnjb3PHmsPBuYIHddnHb4P1m1nzud5y9YIZK1FHPKFHFAFHFAmh7GpvR-nbNBmy-bIiRzwyPEUiuv5HchpHYkn1bkmWmdrf&besl=6&c=news&cf=1&cvrq=1621391&eid_list=201577_202016_204226_204854_205526_207574_209357&expid=201577_202016_202257_202564_204854_205526_205809_207574_209394&fr=20&fv=0&haacp=2073&img_typ=0&itm=0&lu_idc=gzhxy&lukid=3&lus=e091d9df7fbb7e68&lust=63993786&luwtr=14608826967881669318&mscf=0&n=10&nttp=1&p=baidu&pbs=220093&sce=7&sr=72&ssp2=1&tpl=baiduCustITagLinkUnitRankCol&tsf=dtp:1&tu_type=0&u=%2Fitem%2F%25E6%25BF%2580%25E8%25BA%2581%25E6%2580%25A7%25E5%25A4%25A7%25E8%2582%25A0%25E7%2597%2587%25E5%2580%2599%25E7%25BE%25A4%2F4549268%3FfromModule%3Dsearch%2Dresult%5Flemma&uicf=lurecv&urlid=0&eot=1) [csgo电脑配置](https://cpro.baidu.com/cpro/ui/uijs.php?en=mywWUA71T1YsFh7sT7qGujYsFhPC5H0huAbqrauGTdq9TZ0qnauJp1YvrjI-mhcLuhwhujb4nyRsFh_qmLPMUzNaPiN7PzNjPaNDPaNjPiN7PaNDPBNjnzu_IyVG5HfhUyPsUHYLrjcsniuk5HnLnjbYnHfsgvPsTBuzmWYsFMF15HDhTvN_UANzgv-b5HDhTv-b5yRsrH7brywhPvuBmWI-PW6hTLwGujY3FMfqIZKWUA-WpvNbndqCmzuYujYkrHbLPWn1FMwV5Hcvrj6sn1R3niuYUgnqnHmLnjb3PHmsPBuYIHddnHb4P1m1nzud5y9YIZK1FHPKFHFAFHFAmh7GpvR-nbNBmy-bIiRzwyPEUiuv5HchpHdBnWfsPW6kns&besl=6&c=news&cf=1&cvrq=3472465&eid_list=201577_202016_204226_204854_205526_207574_209357&expid=201577_202016_202257_202564_204854_205526_205809_207574_209394&fr=20&fv=0&haacp=611&img_typ=0&itm=0&lu_idc=gzhxy&lukid=4&lus=e091d9df7fbb7e68&lust=63993786&luwtr=2357911270216075011&mscf=0&n=10&nttp=1&p=baidu&pbs=220093&sce=7&sr=72&ssp2=1&tpl=baiduCustITagLinkUnitRankCol&tsf=dtp:1&tu_type=0&u=%2Fitem%2F%25E6%25BF%2580%25E8%25BA%2581%25E6%2580%25A7%25E5%25A4%25A7%25E8%2582%25A0%25E7%2597%2587%25E5%2580%2599%25E7%25BE%25A4%2F4549268%3FfromModule%3Dsearch%2Dresult%5Flemma&uicf=lurecv&urlid=0&eot=1) [战队logo设计](https://cpro.baidu.com/cpro/ui/uijs.php?en=mywWUA71T1YsFh7sT7qGujYsFhPC5H0huAbqrauGTdq9TZ0qnauJp1YvrjI-mhcLuhwhujb4nyRsFh_qFRfdFRFDFRcvFRf1UAqMUzNjriN7raNafzNjPBu_IyVG5HRhUyPsUHY4PHc1Pauk5HnLnjbYnHfsgvPsTBuzmWYsFMF15HDhTvN_UANzgv-b5HDhTv-b5yRsrH7brywhPvuBmWI-PW6hTLwGujY3FMfqIZKWUA-WpvNbndqCmzuYujYkrHbLPWn1FMwV5Hcvrj6sn1R3niuYUgnqnHmLnjb3PHmsPBuYIHddnHb4P1m1nzud5y9YIZK1FHPKFHFAFHFAmh7GpvR-nbNBmy-bIiRzwyPEUiuv5HchpHY3mWR3ujPBn6&besl=6&c=news&cf=1&cvrq=1756705&eid_list=201577_202016_204226_204854_205526_207574_209357&expid=201577_202016_202257_202564_204854_205526_205809_207574_209394&fr=20&fv=0&haacp=707&img_typ=0&itm=0&lu_idc=gzhxy&lukid=5&lus=e091d9df7fbb7e68&lust=63993786&luwtr=1863292750894598650&mscf=0&n=10&nttp=1&p=baidu&pbs=220093&sce=7&sr=72&ssp2=1&tpl=baiduCustITagLinkUnitRankCol&tsf=dtp:1&tu_type=0&u=%2Fitem%2F%25E6%25BF%2580%25E8%25BA%2581%25E6%2580%25A7%25E5%25A4%25A7%25E8%2582%25A0%25E7%2597%2587%25E5%2580%2599%25E7%25BE%25A4%2F4549268%3FfromModule%3Dsearch%2Dresult%5Flemma&uicf=lurecv&urlid=0&eot=1) \| 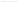[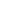](javascript:void(0);)  **16** 网络工口程师  **14** 虚拟[货疊币](javascript:void(0);)平  **15** 图书批发网 \| \| **6** \| [自己创建个网](https://cpro.baidu.com/cpro/ui/uijs.php?en=mywWUA71T1YsFh7sT7qGujYsFhPC5H0huAbqrauGTdq9TZ0qnauJp1YvrjI-mhcLuhwhujb4nyRsFh_qFRfLFRfYFRFjFRFKFRcYFRcYFRFDFRD3FRc3FRmvFRPDFRm3FRfdFRF7FhkdpvbqPBuVmLKV5HDzn1cYnzuk5HnLnjbYnHfsgvPsTBuzmWYsFMF15HDhTvN_UANzgv-b5HDhTv-b5yRsrH7brywhPvuBmWI-PW6hTLwGujY3FMfqIZKWUA-WpvNbndqCmzuYujYkrHbLPWn1FMwV5Hcvrj6sn1R3niuYUgnqnHmLnjb3PHmsPBuYIHddnHb4P1m1nzud5y9YIZK1FHPKFHFAFHFAmh7GpvR-nbNBmy-bIiRzwyPEUiuv5HchpHd9Pj6snjTkms&besl=6&c=news&cf=1&cvrq=2235876&eid_list=201577_202016_204226_204854_205526_207574_209357&expid=201577_202016_202257_202564_204854_205526_205809_207574_209394&fr=20&fv=0&haacp=1001&img_typ=0&itm=0&lu_idc=gzhxy&lukid=6&lus=e091d9df7fbb7e68&lust=63993786&luwtr=6556233252601032990&mscf=0&n=10&nttp=1&p=baidu&pbs=220093&sce=7&sr=72&ssp2=1&tpl=baiduCustITagLinkUnitRankCol&tsf=dtp:1&tu_type=0&u=%2Fitem%2F%25E6%25BF%2580%25E8%25BA%2581%25E6%2580%25A7%25E5%25A4%25A7%25E8%2582%25A0%25E7%2597%2587%25E5%2580%2599%25E7%25BE%25A4%2F4549268%3FfromModule%3Dsearch%2Dresult%5Flemma&uicf=lurecv&urlid=0&eot=1) \| **17** 出版社自费 \| \| **7** \| [游戏盒子](https://cpro.baidu.com/cpro/ui/uijs.php?en=mywWUA71T1YsFh7sT7qGujYsFhPC5H0huAbqrauGTdq9TZ0qnauJp1YvrjI-mhcLuhwhujb4nyRsFh_qFRf1FRP7FRPAFRcLFRFKFRfsFRfLFRf1FhkdpvbqPzuVmLKV5HTzrH0dFMDqn1TsrHfkPjKxmLKzFMFB5H0hTMnqniu1uyk_ugFxpyfqniu1pyfquH04nyf4uAmLuhFBPvRvrau1IA-b5H6hIjdYTAP_pyPouyf1gv9WFMwb5HD4rHTvn1nhIAYqnWm3rj01PH6kFMwVT1YkPWTsrH6dPW0vFMwd5gRkrHbLPWn1FMRqpZwYTZn-nYD-nbm-nbuBmy-ouiRzwyF9pywdFHF7mvqVFMmqnBuG5H6dnyRvnHRL&besl=6&c=news&cf=1&cvrq=3228426&eid_list=201577_202016_204226_204854_205526_207574_209357&expid=201577_202016_202257_202564_204854_205526_205809_207574_209394&fr=20&fv=0&haacp=271&img_typ=0&itm=0&lu_idc=gzhxy&lukid=7&lus=e091d9df7fbb7e68&lust=63993786&luwtr=13245032438307475642&mscf=0&n=10&nttp=1&p=baidu&pbs=220093&sce=7&sr=72&ssp2=1&tpl=baiduCustITagLinkUnitRankCol&tsf=dtp:1&tu_type=0&u=%2Fitem%2F%25E6%25BF%2580%25E8%25BA%2581%25E6%2580%25A7%25E5%25A4%25A7%25E8%2582%25A0%25E7%2597%2587%25E5%2580%2599%25E7%25BE%25A4%2F4549268%3FfromModule%3Dsearch%2Dresult%5Flemma&uicf=lurecv&urlid=0&eot=1) \| [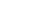](https://cpro.baidu.com/cpro/ui/uijs.php?en=mywWUA71T1YsFh7sT7qGujYsFhPC5H0huAbqrauGTdq9TZ0qnauJp1YvrjI-mhcLuhwhujb4nyRsFh_qIMc-fYm-wbc-fWT-f10-wjD-wDf-f1D-fWThUZNopHYkrauVmLKV5HD1P161Pauk5HnLnjbYnHfsgvPsTBuzmWYsFMF15HDhTvN_UANzgv-b5HDhTv-b5yRsrH7brywhPvuBmWI-PW6hTLwGujY3FMfqIZKWUA-WpvNbndqCmzuYujYkrHbLPWn1FMwV5Hcvrj6sn1R3niuYUgnqnHmLnjb3PHmsPBuYIHddnHb4P1m1nzud5y9YIZK1FHPKFHFAFHFAmh7GpvR-nbNBmy-bIiRzwyPEUiuv5HchpHYzrHwBPADdP6&besl=6&c=news&cf=1&cvrq=4705749&eid_list=201577_202016_204226_204854_205526_207574_209357&expid=201577_202016_202257_202564_204854_205526_205809_207574_209394&fr=20&fv=0&haacp=1024&img_typ=0&itm=0&lu_idc=gzhxy&lukid=18&lus=e091d9df7fbb7e68&lust=63993786&luwtr=754962655298315806&mscf=0&n=10&nttp=1&p=baidu&pbs=220093&sce=7&sr=72&ssp2=1&tpl=baiduCustITagLinkUnitRankCol&tsf=dtp:1&tu_type=0&u=%2Fitem%2F%25E6%25BF%2580%25E8%25BA%2581%25E6%2580%25A7%25E5%25A4%25A7%25E8%2582%25A0%25E7%2597%2587%25E5%2580%2599%25E7%25BE%25A4%2F4549268%3FfromModule%3Dsearch%2Dresult%5Flemma&uicf=lurecv&urlid=0&eot=1)**18** vr消防演练 \| \| **8** \| [哈佛大学申请](https://cpro.baidu.com/cpro/ui/uijs.php?en=mywWUA71T1YsFh7sT7qGujYsFhPC5H0huAbqrauGTdq9TZ0qnauJp1YvrjI-mhcLuhwhujb4nyRsFh_qFRc4FRu7FRcLFRmsFRcYFRm1FRfkFRDLFRn4FRNKFRnLFRNaFRPjFRmdFRFjFRu7FhkdpvbqrauVmLKV5HbknjDvFMDqn1TsrHfkPjKxmLKzFMFB5H0hTMnqniu1uyk_ugFxpyfqniu1pyfquH04nyf4uAmLuhFBPvRvrau1IA-b5H6hIjdYTAP_pyPouyf1gv9WFMwb5HD4rHTvn1nhIAYqnWm3rj01PH6kFMwVT1YkPWTsrH6dPW0vFMwd5gRkrHbLPWn1FMRqpZwYTZn-nYD-nbm-nbuBmy-ouiRzwyF9pywdFHF7mvqVFMmqnBuG5HDsPhn3nym1&besl=6&c=news&cf=1&cvrq=2024363&eid_list=201577_202016_204226_204854_205526_207574_209357&expid=201577_202016_202257_202564_204854_205526_205809_207574_209394&fr=20&fv=0&haacp=870&img_typ=0&itm=0&lu_idc=gzhxy&lukid=8&lus=e091d9df7fbb7e68&lust=63993786&luwtr=7841139350953723636&mscf=0&n=10&nttp=1&p=baidu&pbs=220093&sce=7&sr=72&ssp2=1&tpl=baiduCustITagLinkUnitRankCol&tsf=dtp:1&tu_type=0&u=%2Fitem%2F%25E6%25BF%2580%25E8%25BA%2581%25E6%2580%25A7%25E5%25A4%25A7%25E8%2582%25A0%25E7%2597%2587%25E5%2580%2599%25E7%25BE%25A4%2F4549268%3FfromModule%3Dsearch%2Dresult%5Flemma&uicf=lurecv&urlid=0&eot=1) \| [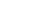](https://cpro.baidu.com/cpro/ui/uijs.php?en=mywWUA71T1YsFh7sT7qGujYsFhPC5H0huAbqrauGTdq9TZ0qnauJp1YvrjI-mhcLuhwhujb4nyRsFh_qFRFjFRwaFRc3FRmkFRckFRR1FRfzFRPaFRcdFRnYFRPAFRR1FRc3FRwaFRcLFRu7FRP7FRmkFRnvFRmLFhkdpvbqnHbhUyPsUHYLrHc3nauk5HnLnjbYnHfsgvPsTBuzmWYsFMF15HDhTvN_UANzgv-b5HDhTv-b5yRsrH7brywhPvuBmWI-PW6hTLwGujY3FMfqIZKWUA-WpvNbndqCmzuYujYkrHbLPWn1FMwV5Hcvrj6sn1R3niuYUgnqnHmLnjb3PHmsPBuYIHddnHb4P1m1nzud5y9YIZK1FHPKFHFAFHFAmh7GpvR-nbNBmy-bIiRzwyPEUiuv5HchpHYYuADvuWfzns&besl=6&c=news&cf=1&cvrq=2363941&eid_list=201577_202016_204226_204854_205526_207574_209357&expid=201577_202016_202257_202564_204854_205526_205809_207574_209394&fr=20&fv=0&haacp=1750&img_typ=0&itm=0&lu_idc=gzhxy&lukid=19&lus=e091d9df7fbb7e68&lust=63993786&luwtr=18274635138362951998&mscf=0&n=10&nttp=1&p=baidu&pbs=220093&sce=7&sr=72&ssp2=1&tpl=baiduCustITagLinkUnitRankCol&tsf=dtp:1&tu_type=0&u=%2Fitem%2F%25E6%25BF%2580%25E8%25BA%2581%25E6%2580%25A7%25E5%25A4%25A7%25E8%2582%25A0%25E7%2597%2587%25E5%2580%2599%25E7%25BE%25A4%2F4549268%3FfromModule%3Dsearch%2Dresult%5Flemma&uicf=lurecv&urlid=0&eot=1)**19** 价格便宜的 \| \| **9** \| [无人机反制](https://cpro.baidu.com/cpro/ui/uijs.php?en=mywWUA71T1YsFh7sT7qGujYsFhPC5H0huAbqrauGTdq9TZ0qnauJp1YvrjI-mhcLuhwhujb4nyRsFh_qFRP7FRw7FRn3FRPaFRFaFRuKFRcLFRcYFRfvFRnvFhkdpvbqriuVmLKV5H6sP1bvFMDqn1TsrHfkPjKxmLKzFMFB5H0hTMnqniu1uyk_ugFxpyfqniu1pyfquH04nyf4uAmLuhFBPvRvrau1IA-b5H6hIjdYTAP_pyPouyf1gv9WFMwb5HD4rHTvn1nhIAYqnWm3rj01PH6kFMwVT1YkPWTsrH6dPW0vFMwd5gRkrHbLPWn1FMRqpZwYTZn-nYD-nbm-nbuBmy-ouiRzwyF9pywdFHF7mvqVFMmqnBuG5yczm1bvPyR3&besl=6&c=news&cf=1&cvrq=1415934&eid_list=201577_202016_204226_204854_205526_207574_209357&expid=201577_202016_202257_202564_204854_205526_205809_207574_209394&fr=20&fv=0&haacp=904&img_typ=0&itm=0&lu_idc=gzhxy&lukid=9&lus=e091d9df7fbb7e68&lust=63993786&luwtr=685006414410405008&mscf=0&n=10&nttp=1&p=baidu&pbs=220093&sce=7&sr=72&ssp2=1&tpl=baiduCustITagLinkUnitRankCol&tsf=dtp:1&tu_type=0&u=%2Fitem%2F%25E6%25BF%2580%25E8%25BA%2581%25E6%2580%25A7%25E5%25A4%25A7%25E8%2582%25A0%25E7%2597%2587%25E5%2580%2599%25E7%25BE%25A4%2F4549268%3FfromModule%3Dsearch%2Dresult%5Flemma&uicf=lurecv&urlid=0&eot=1) \| [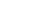](https://cpro.baidu.com/cpro/ui/uijs.php?en=mywWUA71T1YsFh7sT7qGujYsFhPC5H0huAbqrauGTdq9TZ0qnauJp1YvrjI-mhcLuhwhujb4nyRsFh_qmLPMUzNjwaNAraNDPiNawiNawBNKfiNjwBN7Pau_IyVG5HcsFhdWTAYqnHcknjbkFMDqn1TsrHfkPjKxmLKzFMFB5H0hTMnqniu1uyk_ugFxpyfqniu1pyfquH04nyf4uAmLuhFBPvRvrau1IA-b5H6hIjdYTAP_pyPouyf1gv9WFMwb5HD4rHTvn1nhIAYqnWm3rj01PH6kFMwVT1YkPWTsrH6dPW0vFMwd5gRkrHbLPWn1FMRqpZwYTZn-nYD-nbm-nbuBmy-ouiRzwyF9pywdFHF7mvqVFMmqnBuG5ym4nWD1njIb&besl=6&c=news&cf=1&cvrq=4489244&eid_list=201577_202016_204226_204854_205526_207574_209357&expid=201577_202016_202257_202564_204854_205526_205809_207574_209394&fr=20&fv=0&haacp=624&img_typ=0&itm=0&lu_idc=gzhxy&lukid=20&lus=e091d9df7fbb7e68&lust=63993786&luwtr=2267704484931445128&mscf=0&n=10&nttp=1&p=baidu&pbs=220093&sce=7&sr=72&ssp2=1&tpl=baiduCustITagLinkUnitRankCol&tsf=dtp:1&tu_type=0&u=%2Fitem%2F%25E6%25BF%2580%25E8%25BA%2581%25E6%2580%25A7%25E5%25A4%25A7%25E8%2582%25A0%25E7%2597%2587%25E5%2580%2599%25E7%25BE%25A4%2F4549268%3FfromModule%3Dsearch%2Dresult%5Flemma&uicf=lurecv&urlid=0&eot=1)**20** csgo网站开 \| \| **10** \| [网络安全培训](https://cpro.baidu.com/cpro/ui/uijs.php?en=mywWUA71T1YsFh7sT7qGujYsFhPC5H0huAbqrauGTdq9TZ0qnauJp1YvrjI-mhcLuhwhujb4nyRsFh_qFRPDFRm3FRnzFRRLFRcsFRczFRn3FR7aFRndFRRsFRfkFRcdFhkdpvbqnH0hUyPsUHYknW0vP1DhTHY1P104PjDYn7qWTZchThcqnauzT1YkFMP-UAk-T-qGujYkFMPGujd-njbkuj-buWIhmhcLuHm3FMPYpyfqrauY5gwsmvkGmvV-ujPxpAnhIAfqnHb4P1m1nzuYUHYzPW63njndrjDhIAd15HDvP104rjRvnjmhIZRqIHD4rHTvn1nhIHdCIZwsTzR1fiRzwBRzwhF9pyV-FHF7mh7GuZR-nbNWUvYhIWYzFhbqPAckP179PHc&besl=6&c=news&cf=1&cvrq=3292150&eid_list=201577_202016_204226_204854_205526_207574_209357&expid=201577_202016_202257_202564_204854_205526_205809_207574_209394&fr=20&fv=0&haacp=1162&img_typ=0&itm=0&lu_idc=gzhxy&lukid=10&lus=e091d9df7fbb7e68&lust=63993786&luwtr=2249689388344567100&mscf=0&n=10&nttp=1&p=baidu&pbs=220093&sce=7&sr=72&ssp2=1&tpl=baiduCustITagLinkUnitRankCol&tsf=dtp:1&tu_type=0&u=%2Fitem%2F%25E6%25BF%2580%25E8%25BA%2581%25E6%2580%25A7%25E5%25A4%25A7%25E8%2582%25A0%25E7%2597%2587%25E5%2580%2599%25E7%25BE%25A4%2F4549268%3FfromModule%3Dsearch%2Dresult%5Flemma&uicf=lurecv&urlid=0&eot=1) \| [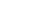](https://cpro.baidu.com/cpro/ui/uijs.php?en=mywWUA71T1YsFh7sT7qGujYsFhPC5H0huAbqrauGTdq9TZ0qnauJp1YvrjI-mhcLuhwhujb4nyRsFh_qFRfYFRmdFRn1FRcYFRcYFRcYFRFDFRD3FRfsFRDkFRc1FRPjFRfsFRmzFhkdpvbqnWDhUyPsUHY3n1mkniuk5HnLnjbYnHfsgvPsTBuzmWYsFMF15HDhTvN_UANzgv-b5HDhTv-b5yRsrH7brywhPvuBmWI-PW6hTLwGujY3FMfqIZKWUA-WpvNbndqCmzuYujYkrHbLPWn1FMwV5Hcvrj6sn1R3niuYUgnqnHmLnjb3PHmsPBuYIHddnHb4P1m1nzud5y9YIZK1FHPKFHFAFHFAmh7GpvR-nbNBmy-bIiRzwyPEUiuv5HchpHYzrHKBPWKhm6&besl=6&c=news&cf=1&cvrq=2189719&eid_list=201577_202016_204226_204854_205526_207574_209357&expid=201577_202016_202257_202564_204854_205526_205809_207574_209394&fr=20&fv=0&haacp=734&img_typ=0&itm=0&lu_idc=gzhxy&lukid=21&lus=e091d9df7fbb7e68&lust=63993786&luwtr=7550494890368818072&mscf=0&n=10&nttp=1&p=baidu&pbs=220093&sce=7&sr=72&ssp2=1&tpl=baiduCustITagLinkUnitRankCol&tsf=dtp:1&tu_type=0&u=%2Fitem%2F%25E6%25BF%2580%25E8%25BA%2581%25E6%2580%25A7%25E5%25A4%25A7%25E8%2582%25A0%25E7%2597%2587%25E5%2580%2599%25E7%25BE%25A4%2F4549268%3FfromModule%3Dsearch%2Dresult%5Flemma&uicf=lurecv&urlid=0&eot=1)**21** 怎么创建小 \| \| **11** \| [亚马逊图书](https://cpro.baidu.com/cpro/ui/uijs.php?en=mywWUA71T1YsFh7sT7qGujYsFhPC5H0huAbqrauGTdq9TZ0qnauJp1YvrjI-mhcLuhwhujb4nyRsFh_qFRfkFRnLFRnzFRNDFRfkFRcLFRPDFRFjFRPKFRR4FhkdpvbqnHDhUyPsUHY3nj0dPiuk5HnLnjbYnHfsgvPsTBuzmWYsFMF15HDhTvN_UANzgv-b5HDhTv-b5yRsrH7brywhPvuBmWI-PW6hTLwGujY3FMfqIZKWUA-WpvNbndqCmzuYujYkrHbLPWn1FMwV5Hcvrj6sn1R3niuYUgnqnHmLnjb3PHmsPBuYIHddnHb4P1m1nzud5y9YIZK1FHPKFHFAFHFAmh7GpvR-nbNBmy-bIiRzwyPEUiuv5HchpHYvuHFBm1T3n0&besl=6&c=news&cf=1&cvrq=3335536&eid_list=201577_202016_204226_204854_205526_207574_209357&expid=201577_202016_202257_202564_204854_205526_205809_207574_209394&fr=20&fv=0&haacp=219&img_typ=0&itm=0&lu_idc=gzhxy&lukid=11&lus=e091d9df7fbb7e68&lust=63993786&luwtr=718744168689952326&mscf=0&n=10&nttp=1&p=baidu&pbs=220093&sce=7&sr=72&ssp2=1&tpl=baiduCustITagLinkUnitRankCol&tsf=dtp:1&tu_type=0&u=%2Fitem%2F%25E6%25BF%2580%25E8%25BA%2581%25E6%2580%25A7%25E5%25A4%25A7%25E8%2582%25A0%25E7%2597%2587%25E5%2580%2599%25E7%25BE%25A4%2F4549268%3FfromModule%3Dsearch%2Dresult%5Flemma&uicf=lurecv&urlid=0&eot=1) \| [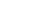](https://cpro.baidu.com/cpro/ui/uijs.php?en=mywWUA71T1YsFh7sT7qGujYsFhPC5H0huAbqrauGTdq9TZ0qnauJp1YvrjI-mhcLuhwhujb4nyRsFh_qFRc4FRD4FRf1FRDvFRnkFRcYFRc4FRwjFRnsFRNDFRnvFRFDFRPjFRD3FhkdpvbqnWchUyPsUHY3PH0Lnauk5HnLnjbYnHfsgvPsTBuzmWYsFMF15HDhTvN_UANzgv-b5HDhTv-b5yRsrH7brywhPvuBmWI-PW6hTLwGujY3FMfqIZKWUA-WpvNbndqCmzuYujYkrHbLPWn1FMwV5Hcvrj6sn1R3niuYUgnqnHmLnjb3PHmsPBuYIHddnHb4P1m1nzud5y9YIZK1FHPKFHFAFHFAmh7GpvR-nbNBmy-bIiRzwyPEUiuv5HchpHd-PAP9Ph7WPf&besl=6&c=news&cf=1&cvrq=2271496&eid_list=201577_202016_204226_204854_205526_207574_209357&expid=201577_202016_202257_202564_204854_205526_205809_207574_209394&fr=20&fv=0&haacp=952&img_typ=0&itm=0&lu_idc=gzhxy&lukid=22&lus=e091d9df7fbb7e68&lust=63993786&luwtr=6130823900782380766&mscf=0&n=10&nttp=1&p=baidu&pbs=220093&sce=7&sr=72&ssp2=1&tpl=baiduCustITagLinkUnitRankCol&tsf=dtp:1&tu_type=0&u=%2Fitem%2F%25E6%25BF%2580%25E8%25BA%2581%25E6%2580%25A7%25E5%25A4%25A7%25E8%2582%25A0%25E7%2597%2587%25E5%2580%2599%25E7%25BE%25A4%2F4549268%3FfromModule%3Dsearch%2Dresult%5Flemma&uicf=lurecv&urlid=0&eot=1)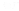**22** 供应链管理 \| |
|  | 疾病类型  [小 播报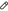编辑](javascript:;)  [激燥性大肠症](https://baike.baidu.com/item/%E6%BF%80%E7%87%A5%E6%80%A7%E5%A4%A7%E8%82%A0%E7%97%87/1506814?fromModule=lemma_inlink)有三种：  (一) 慢性无痛间歇性腹泻 ：很多人都有胃肠毛病，如腹泻、便秘、胀气等却又查不出原因所在。  (二) 慢性间歇性腹痛与便秘： [痉挛性结肠炎](https://baike.baidu.com/item/%E7%97%89%E6%8C%9B%E6%80%A7%E7%BB%93%E8%82%A0%E7%82%8E?fromModule=lemma_inlink)的病人主诉有慢性腹痛和便秘。  (三) 腹泻与便秘交替出现 ：还有一些病人可出现上述两种表现，便秘与腹泻交替出现。  临床表现  [小 播报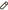编辑](javascript:;) |  |  |
|  | 典型病人可数年或数月内间断出现水样腹泻，常在清晨或早餐后腹泻加重，在排泄3-4次带大量黏液的稀便后，病人其余时 间均感觉正常。整天腹泻，或总是夜间腹泻者很少见。腹泻可持续几周或数月，随后自动消失一段时间。有些病人无腹泻而排泄 “铅笔样”糊状大便。 病人另一种典型的表现是慢性腹痛，伴便秘，或者便秘与腹泻交替出现。这些病人主诉间断性下腹部绞痛， 排气或排便后可缓解。激燥性大肠症还常有胃灼热、明显腹胀、 [背痛](https://baike.baidu.com/item/%E8%83%8C%E7%97%9B?fromModule=lemma_inlink)、软弱、疲乏、心悸等。 |  |  |
|  | 病理生理  [小 播报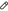编辑](javascript:;) |  |  |
|  | 激燥性大肠症的基本病理生理异常是肠运动改变。 [结肠痉挛](https://baike.baidu.com/item/%E7%BB%93%E8%82%A0%E7%97%89%E6%8C%9B/16178467?fromModule=lemma_inlink)的病人(腹痛和便秘)静止时结肠动力增加。  与此相反，主要表现为腹泻的病人静止时结肠动力减弱。某些[激燥性大肠症](https://baike.baidu.com/item/%E6%BF%80%E7%87%A5%E6%80%A7%E5%A4%A7%E8%82%A0%E7%97%87/1506814?fromModule=lemma_inlink)病人可有显著精神异常的证据。常见的有忧郁、 歇斯底里、强迫症。  精神刺激也可使症状加重。然而应该注意是正常人在急性压力反应时，结肠内压力可升高。  这提示精神刺激可能是激燥性大肠症的非特异性诱发因素，同在其他疾病中一样，和许多病因一起致病。 |  |  |
|  |  |  | 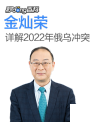 |
|  | 诊断要点  [小 播报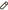编辑](javascript:;) |  |  |
|  | [1.有上述胃肠功能失常的症状及伴有全身性官能症状。 2.患者的症状常随情绪而变化。 3.全消化道摄影检查或胃镜、 直肠镜 检查正常，排除器质性病变。 三、病因病机： 1.感受外邪：六淫之中以寒、湿、暑、热为常见，又以感受湿邪致泻者尤多。](https://baike.baidu.com/item/%E7%9B%B4%E8%82%A0%E9%95%9C%E6%A3%80%E6%9F%A5/16306279?fromModule=lemma_inlink)  2.饮食所伤：多为饮食过量，宿食内停；或不节肥甘，呆胃滞脾；或生冷不洁，有伤脾胃。  3.情志失调：肝郁乘脾 4.体虚久病：脾虚及肾 |  |  |
|  | 治疗方式  [小 播报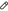编辑](javascript:;) |  |  |

<https://baike.baidu.com/item/>激躁性大肠症候群/4549268?fromModule=search-result_lemma 1/2


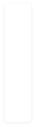

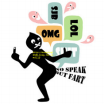
2022/12/14 10:41

[
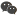

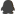
口](javascript:void(0);)

寒湿为[泄泻](https://baike.baidu.com/item/%E6%B3%84%E6%B3%BB?fromModule=lemma_inlink)的主要病理因素， [脾虚湿盛](https://baike.baidu.com/item/%E8%84%BE%E8%99%9A%E6%B9%BF%E7%9B%9B/9969264?fromModule=lemma_inlink)是其发病关键。固治疗当以运脾祛湿为原则。辩证施治

1.[寒湿困脾](https://baike.baidu.com/item/%E5%AF%92%E6%B9%BF%E5%9B%B0%E8%84%BE?fromModule=lemma_inlink)：散寒化湿， [健脾](https://baike.baidu.com/item/%E5%81%A5%E8%84%BE?fromModule=lemma_inlink)止泻

2.[湿热下注](https://baike.baidu.com/item/%E6%B9%BF%E7%83%AD%E4%B8%8B%E6%B3%A8/2874619?fromModule=lemma_inlink)： [清热利湿](https://baike.baidu.com/item/%E6%B8%85%E7%83%AD%E5%88%A9%E6%B9%BF?fromModule=lemma_inlink)，厚肠止泻

3.食滞肠胃： [消食导滞](https://baike.baidu.com/item/%E6%B6%88%E9%A3%9F%E5%AF%BC%E6%BB%9E?fromModule=lemma_inlink)、调和脾胃

4.肝气乘脾：抑肝扶脾、调和脾胃

5.脾虚湿盛：健脾益气、运中止泻

6.[脾肾阳虚](https://baike.baidu.com/item/%E8%84%BE%E8%82%BE%E9%98%B3%E8%99%9A/7341157?fromModule=lemma_inlink)：温补脾肾、固肠止泻

预防保养

1.注意饮食卫生，起居有常

2.勿过食生冷、或肥甘厚腻、或饮酒无度

3.注意情志因素

4.注意保暖，切勿受湿受凉

[词条图册 更多图册 >](https://baike.baidu.com/pic/%E6%BF%80%E8%BA%81%E6%80%A7%E5%A4%A7%E8%82%A0%E7%97%87%E5%80%99%E7%BE%A4/4549268?fr=lemma)

[概述图册(1)](https://baike.baidu.com/pic/%E6%BF%80%E8%BA%81%E6%80%A7%E5%A4%A7%E8%82%A0%E7%97%87%E5%80%99%E7%BE%A4/4549268/1/0b55b319ebc4b74570b842efc5fc1e178a82151e?fr=lemma)

[女
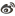
](javascript:void(0);)

[小 播报
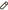
编辑](javascript:;)

激躁性大肠症候群_百度百科


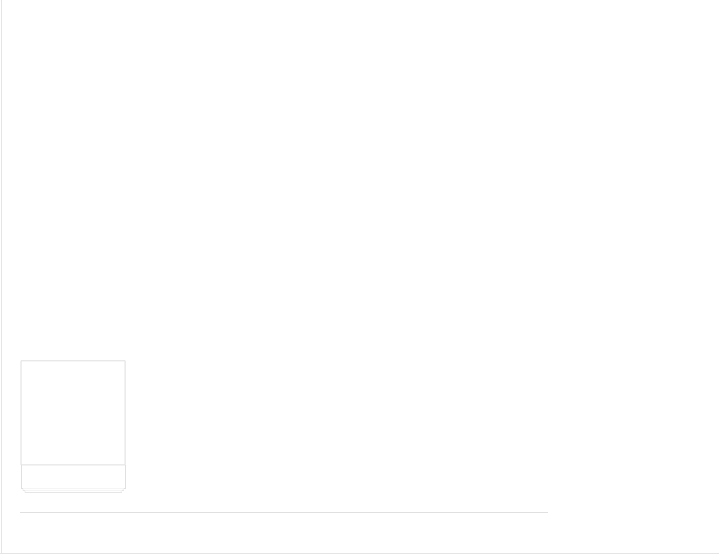


| [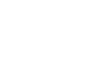](http://www.baidu.com/baidu.php?url=Ks00000EAMrnlPLIyCNSmyXz9UGTU52obwY0vDioEMchaZhpNgYYofrKouLvcTqto0hdwElFKg6Iv-oyLybb3CA9pGUy9OdhJ5mFJfWFJbzMFvrZabGsfmVVjXuet92gcBQVas355TwhaU0ZSy2M2gpeb7htKt3pj6losOyuKSs19s0ze-hysXcjXvATQ-OBUJ5nT0dRNCBD-JM7CdFnktr_Jh_3.7Y_NR2Ar5Od66E89WtJPgKwzl526eZKfHZf68Hfuxwe3h2SMowJ32rgwuuuY4PlhGv-5QWdQjPakYeVdB6.U1Yk0ZDqE2HRlVjRV5r1GVpB8EQc86KY5IHJYOHgdQQB0A-V5HDzPWc0Iybq0ZKGujYzn0KWpyfqP1c0mhbqn10k0AuY5H00TA6qn0KET1Ys0AFL5H00UMfqn0K1XWY0ThIYmyTqn0K8IM0qna3snj0snj0sn0K-ThTqn0KYTh7buHYs0AFbpyfqnW77fbc4nYu7wWFDnRwArj97fYfYrD7DrRwKPDnvnbD0uAPWujY0mgPxpywW5gK1QyIlpZ940AqW5HD0u1dLTv41IZc0TMfqn1bY0Z7spyfqn0Kkmv-b5H00mycqn7ts0ZKs5H00Ugws5H00uAwETjYk0ZFJ5H00IZN15HnsPHf4PW6vP1TzPWcknH6krjb0mynqnfKsUWYs0ZK9I7qhUA7M5H00ugPY5H00ugwGujYVnfK9TLKWm1Ys0ZNspy4Wm1Ys0AuWIgfqn0K9uAu_myTqnfKLuMFEUHY0mMfqnfKzug7Y5HDvP104rjRvPHbdrHR0Tv-b5H0smhc1PHD4rHc4mHw-PWD0ULfqnfKETMKY5HcWnanknanzc1b1PWTdnWbsnan1rH0sc1n4nj08nan1c1cWnanV0AVG5H00UgfqnW0vn6KVm1YzPWfdn16sn1mkn0KVmdqhThqV5H00uA78IyF-gLK_my4GuZnqn0K9uZ745UAGdroyCogD_XL5d6K9uZ7Y5H00pgPWUjYs0Z7VIjYs0A7bgLPEIgFWuHYznzPYpgw_uNqkIyNzXiPxgdqxUMnWIA-YUARWUhksgvkY0APzm1Yvn1TsP6&us=newvui&ai=0_429389852_1_0&word=&ck=0.0.0.0.0.0.0.0&shh=baike.baidu.com)猜你喜欢    [躁郁自测--抑郁自评量表SDS，标准版](http://www.baidu.com/baidu.php?url=Ks00000EAMrnlPLIyCNSmyXz9UGTU52obwY0vDioEMchaZhpNgYYofrKouLvcTqto0hdwElFKg6Iv-oyLybb3CA9pGUy9OdhJ5mFJfWFJbzMFvrZabGsfmVVjXuet92gcBQVas355TwhaU0ZSy2M2gpeb7htKt3pj6losOyuKSs19s0ze-hysXcjXvATQ-OBUJ5nT0dRNCBD-JM7CdFnktr_Jh_3.7Y_NR2Ar5Od66E89WtJPgKwzl526eZKfHZf68Hfuxwe3h2SMowJ32rgwuuuY4PlhGv-5QWdQjPakYeVdB6.U1Yk0ZDqE2HRlVjRV5r1GVpB8EQc86KY5IHJYOHgdQQB0A-V5HDzPWc0Iybq0ZKGujYzn0KWpyfqP1c0mhbqn10k0AuY5H00TA6qn0KET1Ys0AFL5H00UMfqn0K1XWY0ThIYmyTqn0K8IM0qna3snj0snj0sn0K-ThTqn0KYTh7buHYs0AFbpyfqnW77fbc4nYu7wWFDnRwArj97fYfYrD7DrRwKPDnvnbD0uAPWujY0mgPxpywW5gK1QyIlpZ940AqW5HD0u1dLTv41IZc0TMfqn1bY0Z7spyfqn0Kkmv-b5H00mycqn7ts0ZKs5H00Ugws5H00uAwETjYk0ZFJ5H00IZN15HnsPHf4PW6vP1TzPWcknH6krjb0mynqnfKsUWYs0ZK9I7qhUA7M5H00ugPY5H00ugwGujYVnfK9TLKWm1Ys0ZNspy4Wm1Ys0AuWIgfqn0K9uAu_myTqnfKLuMFEUHY0mMfqnfKzug7Y5HDvP104rjRvPHbdrHR0Tv-b5H0smhc1PHD4rHc4mHw-PWD0ULfqnfKETMKY5HcWnanknanzc1b1PWTdnWbsnan1rH0sc1n4nj08nan1c1cWnanV0AVG5H00UgfqnW0vn6KVm1YzPWfdn16sn1mkn0KVmdqhThqV5H00uA78IyF-gLK_my4GuZnqn0K9uZ745UAGdroyCogD_XL5d6K9uZ7Y5H00pgPWUjYs0Z7VIjYs0A7bgLPEIgFWuHYznzPYpgw_uNqkIyNzXiPxgdqxUMnWIA-YUARWUhksgvkY0APzm1Yvn1TsP6&us=newvui&ai=0_429389852_1_0&word=&ck=0.0.0.0.0.0.0.0&shh=baike.baidu.com)  [躁郁自测，抑郁测量表，网上的测评试题 评分标准，综合心理，](http://www.baidu.com/baidu.php?url=Ks00000EAMrnlPLIyCNSmyXz9UGTU52obwY0vDioEMchaZhpNgYYofrKouLvcTqto0hdwElFKg6Iv-oyLybb3CA9pGUy9OdhJ5mFJfWFJbzMFvrZabGsfmVVjXuet92gcBQVas355TwhaU0ZSy2M2gpeb7htKt3pj6losOyuKSs19s0ze-hysXcjXvATQ-OBUJ5nT0dRNCBD-JM7CdFnktr_Jh_3.7Y_NR2Ar5Od66E89WtJPgKwzl526eZKfHZf68Hfuxwe3h2SMowJ32rgwuuuY4PlhGv-5QWdQjPakYeVdB6.U1Yk0ZDqE2HRlVjRV5r1GVpB8EQc86KY5IHJYOHgdQQB0A-V5HDzPWc0Iybq0ZKGujYzn0KWpyfqP1c0mhbqn10k0AuY5H00TA6qn0KET1Ys0AFL5H00UMfqn0K1XWY0ThIYmyTqn0K8IM0qna3snj0snj0sn0K-ThTqn0KYTh7buHYs0AFbpyfqnW77fbc4nYu7wWFDnRwArj97fYfYrD7DrRwKPDnvnbD0uAPWujY0mgPxpywW5gK1QyIlpZ940AqW5HD0u1dLTv41IZc0TMfqn1bY0Z7spyfqn0Kkmv-b5H00mycqn7ts0ZKs5H00Ugws5H00uAwETjYk0ZFJ5H00IZN15HnsPHf4PW6vP1TzPWcknH6krjb0mynqnfKsUWYs0ZK9I7qhUA7M5H00ugPY5H00ugwGujYVnfK9TLKWm1Ys0ZNspy4Wm1Ys0AuWIgfqn0K9uAu_myTqnfKLuMFEUHY0mMfqnfKzug7Y5HDvP104rjRvPHbdrHR0Tv-b5H0smhc1PHD4rHc4mHw-PWD0ULfqnfKETMKY5HcWnanknanzc1b1PWTdnWbsnan1rH0sc1n4nj08nan1c1cWnanV0AVG5H00UgfqnW0vn6KVm1YzPWfdn16sn1mkn0KVmdqhThqV5H00uA78IyF-gLK_my4GuZnqn0K9uZ745UAGdroyCogD_XL5d6K9uZ7Y5H00pgPWUjYs0Z7VIjYs0A7bgLPEIgFWuHYznzPYpgw_uNqkIyNzXiPxgdqxUMnWIA-YUARWUhksgvkY0APzm1Yvn1TsP6&us=newvui&ai=0_429389852_1_0&word=&ck=0.0.0.0.0.0.0.0&shh=baike.baidu.com)  [健康测试，心理测试，心理抑郁，全方位解读，图表分析，针对 …](http://www.baidu.com/baidu.php?url=Ks00000EAMrnlPLIyCNSmyXz9UGTU52obwY0vDioEMchaZhpNgYYofrKouLvcTqto0hdwElFKg6Iv-oyLybb3CA9pGUy9OdhJ5mFJfWFJbzMFvrZabGsfmVVjXuet92gcBQVas355TwhaU0ZSy2M2gpeb7htKt3pj6losOyuKSs19s0ze-hysXcjXvATQ-OBUJ5nT0dRNCBD-JM7CdFnktr_Jh_3.7Y_NR2Ar5Od66E89WtJPgKwzl526eZKfHZf68Hfuxwe3h2SMowJ32rgwuuuY4PlhGv-5QWdQjPakYeVdB6.U1Yk0ZDqE2HRlVjRV5r1GVpB8EQc86KY5IHJYOHgdQQB0A-V5HDzPWc0Iybq0ZKGujYzn0KWpyfqP1c0mhbqn10k0AuY5H00TA6qn0KET1Ys0AFL5H00UMfqn0K1XWY0ThIYmyTqn0K8IM0qna3snj0snj0sn0K-ThTqn0KYTh7buHYs0AFbpyfqnW77fbc4nYu7wWFDnRwArj97fYfYrD7DrRwKPDnvnbD0uAPWujY0mgPxpywW5gK1QyIlpZ940AqW5HD0u1dLTv41IZc0TMfqn1bY0Z7spyfqn0Kkmv-b5H00mycqn7ts0ZKs5H00Ugws5H00uAwETjYk0ZFJ5H00IZN15HnsPHf4PW6vP1TzPWcknH6krjb0mynqnfKsUWYs0ZK9I7qhUA7M5H00ugPY5H00ugwGujYVnfK9TLKWm1Ys0ZNspy4Wm1Ys0AuWIgfqn0K9uAu_myTqnfKLuMFEUHY0mMfqnfKzug7Y5HDvP104rjRvPHbdrHR0Tv-b5H0smhc1PHD4rHc4mHw-PWD0ULfqnfKETMKY5HcWnanknanzc1b1PWTdnWbsnan1rH0sc1n4nj08nan1c1cWnanV0AVG5H00UgfqnW0vn6KVm1YzPWfdn16sn1mkn0KVmdqhThqV5H00uA78IyF-gLK_my4GuZnqn0K9uZ745UAGdroyCogD_XL5d6K9uZ7Y5H00pgPWUjYs0Z7VIjYs0A7bgLPEIgFWuHYznzPYpgw_uNqkIyNzXiPxgdqxUMnWIA-YUARWUhksgvkY0APzm1Yvn1TsP6&us=newvui&ai=0_429389852_1_0&word=&ck=0.0.0.0.0.0.0.0&shh=baike.baidu.com)  [bdcs.lhwljy.top](http://www.baidu.com/baidu.php?url=Ks00000EAMrnlPLIyCNSmyXz9UGTU52obwY0vDioEMchaZhpNgYYofrKouLvcTqto0hdwElFKg6Iv-oyLybb3CA9pGUy9OdhJ5mFJfWFJbzMFvrZabGsfmVVjXuet92gcBQVas355TwhaU0ZSy2M2gpeb7htKt3pj6losOyuKSs19s0ze-hysXcjXvATQ-OBUJ5nT0dRNCBD-JM7CdFnktr_Jh_3.7Y_NR2Ar5Od66E89WtJPgKwzl526eZKfHZf68Hfuxwe3h2SMowJ32rgwuuuY4PlhGv-5QWdQjPakYeVdB6.U1Yk0ZDqE2HRlVjRV5r1GVpB8EQc86KY5IHJYOHgdQQB0A-V5HDzPWc0Iybq0ZKGujYzn0KWpyfqP1c0mhbqn10k0AuY5H00TA6qn0KET1Ys0AFL5H00UMfqn0K1XWY0ThIYmyTqn0K8IM0qna3snj0snj0sn0K-ThTqn0KYTh7buHYs0AFbpyfqnW77fbc4nYu7wWFDnRwArj97fYfYrD7DrRwKPDnvnbD0uAPWujY0mgPxpywW5gK1QyIlpZ940AqW5HD0u1dLTv41IZc0TMfqn1bY0Z7spyfqn0Kkmv-b5H00mycqn7ts0ZKs5H00Ugws5H00uAwETjYk0ZFJ5H00IZN15HnsPHf4PW6vP1TzPWcknH6krjb0mynqnfKsUWYs0ZK9I7qhUA7M5H00ugPY5H00ugwGujYVnfK9TLKWm1Ys0ZNspy4Wm1Ys0AuWIgfqn0K9uAu_myTqnfKLuMFEUHY0mMfqnfKzug7Y5HDvP104rjRvPHbdrHR0Tv-b5H0smhc1PHD4rHc4mHw-PWD0ULfqnfKETMKY5HcWnanknanzc1b1PWTdnWbsnan1rH0sc1n4nj08nan1c1cWnanV0AVG5H00UgfqnW0vn6KVm1YzPWfdn16sn1mkn0KVmdqhThqV5H00uA78IyF-gLK_my4GuZnqn0K9uZ745UAGdroyCogD_XL5d6K9uZ7Y5H00pgPWUjYs0Z7VIjYs0A7bgLPEIgFWuHYznzPYpgw_uNqkIyNzXiPxgdqxUMnWIA-YUARWUhksgvkY0APzm1Yvn1TsP6&us=newvui&ai=0_429389852_1_0&word=&ck=0.0.0.0.0.0.0.0&shh=baike.baidu.com) |
| --- |

| 岔 搜索发现   [激躁性大肠症候群](https://www.baidu.com/s?word=%E6%BF%80%E8%BA%81%E6%80%A7%E5%A4%A7%E8%82%A0%E7%97%87%E5%80%99%E7%BE%A4&tn=SE_baikepcxf02_fcetbk02&pos=baike_pc_turbo_1767&ori_sid=00bb350b8f61098b)   [大肠激躁症能喝酸奶吗](https://www.baidu.com/s?word=%E5%A4%A7%E8%82%A0%E6%BF%80%E8%BA%81%E7%97%87%E8%83%BD%E5%96%9D%E9%85%B8%E5%A5%B6%E5%90%97&tn=SE_baikepcxf02_fcetbk02&pos=baike_pc_turbo_1767&ori_sid=00bb350b8f61098b) |  [肠炎的症状](https://www.baidu.com/s?word=%E8%82%A0%E7%82%8E%E7%9A%84%E7%97%87%E7%8A%B6&tn=SE_baikepcxf02_fcetbk02&pos=baike_pc_turbo_1767&ori_sid=00bb350b8f61098b)   [大肠激躁症可以自愈](https://www.baidu.com/s?word=%E5%A4%A7%E8%82%A0%E6%BF%80%E8%BA%81%E7%97%87%E5%8F%AF%E4%BB%A5%E8%87%AA%E6%84%88&tn=SE_baikepcxf02_fcetbk02&pos=baike_pc_turbo_1767&ori_sid=00bb350b8f61098b) |  [激躁性大肠要吃药吗](https://www.baidu.com/s?word=%E6%BF%80%E8%BA%81%E6%80%A7%E5%A4%A7%E8%82%A0%E8%A6%81%E5%90%83%E8%8D%AF%E5%90%97&tn=SE_baikepcxf02_fcetbk02&pos=baike_pc_turbo_1767&ori_sid=00bb350b8f61098b)  [狂躁症](https://www.baidu.com/s?word=%E7%8B%82%E8%BA%81%E7%97%87&tn=SE_baikepcxf02_fcetbk02&pos=baike_pc_turbo_1767&ori_sid=00bb350b8f61098b) |  [肠梗阻症状及治疗](https://www.baidu.com/s?word=%E8%82%A0%E6%A2%97%E9%98%BB%E7%97%87%E7%8A%B6%E5%8F%8A%E6%B2%BB%E7%96%97&tn=SE_baikepcxf02_fcetbk02&pos=baike_pc_turbo_1767&ori_sid=00bb350b8f61098b)   [暴躁症](https://www.baidu.com/s?word=%E6%9A%B4%E8%BA%81%E7%97%87&tn=SE_baikepcxf02_fcetbk02&pos=baike_pc_turbo_1767&ori_sid=00bb350b8f61098b) |  [运动治好了我的肠易激](https://www.baidu.com/s?word=%E8%BF%90%E5%8A%A8%E6%B2%BB%E5%A5%BD%E4%BA%86%E6%88%91%E7%9A%84%E8%82%A0%E6%98%93%E6%BF%80&tn=SE_baikepcxf02_fcetbk02&pos=baike_pc_turbo_1767&ori_sid=00bb350b8f61098b)  [狂躁症吃什么药](https://www.baidu.com/s?word=%E7%8B%82%E8%BA%81%E7%97%87%E5%90%83%E4%BB%80%E4%B9%88%E8%8D%AF&tn=SE_baikepcxf02_fcetbk02&pos=baike_pc_turbo_1767&ori_sid=00bb350b8f61098b) |
| --- | --- | --- | --- | --- |

| Q | 新手上路 [成长任务](https://baike.baidu.com/usercenter/tasks#guide)  [编辑规则](https://baike.baidu.com/help#main06) | [编辑入门](https://baike.baidu.com/help#main01)  [本人编辑](https://baike.baidu.com/item/%E7%99%BE%E5%BA%A6%E7%99%BE%E7%A7%91%EF%BC%9A%E6%9C%AC%E4%BA%BA%E8%AF%8D%E6%9D%A1%E7%BC%96%E8%BE%91%E6%9C%8D%E5%8A%A1/22442459?bk_fr=pcFooter) 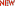 | 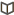 | 我有疑问 [内容质疑](javascript:void(0);) [官方贴吧](http://tieba.baidu.com/f?ie=utf-8&fr=bks0000&kw=%E7%99%BE%E5%BA%A6%E7%99%BE%E7%A7%91) | 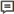 投诉建议  [举报不良信息](http://help.baidu.com/newadd?word=%E6%BF%80%E8%BA%81%E6%80%A7%E5%A4%A7%E8%82%A0%E7%97%87%E5%80%99%E7%BE%A4&&submit_link=https%3A%2F%2Fbaike.baidu.com%2Fitem%2F%25E6%25BF%2580%25E8%25BA%2581%25E6%2580%25A7%25E5%25A4%25A7%25E8%2582%25A0%25E7%2597%2587%25E5%2580%2599%25E7%25BE%25A4%2F4549268%3FfromModule%3Dsearch-result_lemma&prod_id=10&category=1) [投诉侵权信息](http://help.baidu.com/newadd?word=%E6%BF%80%E8%BA%81%E6%80%A7%E5%A4%A7%E8%82%A0%E7%97%87%E5%80%99%E7%BE%A4&&submit_link=https%3A%2F%2Fbaike.baidu.com%2Fitem%2F%25E6%25BF%2580%25E8%25BA%2581%25E6%2580%25A7%25E5%25A4%25A7%25E8%2582%25A0%25E7%2597%2587%25E5%2580%2599%25E7%25BE%25A4%2F4549268%3FfromModule%3Dsearch-result_lemma&prod_id=10&category=6)  [在线客服](http://zhiqiu.baidu.com/baike/passport/html/baikechat.html) [意见反馈](javascript:void(0);) |
| --- | --- | --- | --- | --- | --- |

©2022 Baidu [使用百度前必读](http://www.baidu.com/duty/) | [百科协议](http://help.baidu.com/question?prod_en=baike&class=89&id=1637) | [隐私政策](http://help.baidu.com/question?prod_id=10&class=690&id=1001779) | [百度百科合作平台](https://baike.baidu.com/operation/cooperation) | 京ICP证030173号
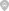
 [京公网安备11000002000001号](http://www.beian.gov.cn/portal/registerSystemInfo?recordcode=11000002000001)

<https://baike.baidu.com/item/>激躁性大肠症候群/4549268?fromModule=search-result_lemma

[
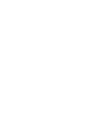
](http://baike.baidu.com/l/WWoXYu7P)[未通过词条申诉](http://help.baidu.com/newadd?word=%E6%BF%80%E8%BA%81%E6%80%A7%E5%A4%A7%E8%82%A0%E7%97%87%E5%80%99%E7%BE%A4&&submit_link=https%3A%2F%2Fbaike.baidu.com%2Fitem%2F%25E6%25BF%2580%25E8%25BA%2581%25E6%2580%25A7%25E5%25A4%25A7%25E8%2582%25A0%25E7%2597%2587%25E5%2580%2599%25E7%25BE%25A4%2F4549268%3FfromModule%3Dsearch-result_lemma&prod_id=10&category=2)

[封禁查询与解封](http://help.baidu.com/newadd?word=%E6%BF%80%E8%BA%81%E6%80%A7%E5%A4%A7%E8%82%A0%E7%97%87%E5%80%99%E7%BE%A4&&submit_link=https%3A%2F%2Fbaike.baidu.com%2Fitem%2F%25E6%25BF%2580%25E8%25BA%2581%25E6%2580%25A7%25E5%25A4%25A7%25E8%2582%25A0%25E7%2597%2587%25E5%2580%2599%25E7%25BE%25A4%2F4549268%3FfromModule%3Dsearch-result_lemma&prod_id=10&category=5)


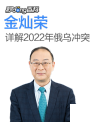


2/2
