# Supplementary material for: Assessment of medical information on irritable bowel syndrome information in Wikipedia and Baidu Encyclopedia: comparative study
Source: PeerJ. 2024 May 24;12:e17264. doi: 10.7717/peerj.17264 (PMC11129691; doi:10.7717/peerj.17264)
Supplement: Data S1 [file peerj-12-17264-s001.zip › σÄƒσoïμò░μì«/Baidu/Baidu-Chinese/3-Φéáμÿôμ┐Çτ╗╝σÉêσ╛ü∩╝ê2005σ╣┤Σ╕¡σ¢╜σî╗Φì»τoæμèÇσç║τëêτñ╛σç║τëêτÜäσ¢╛Σ╣a∩╝ë_τÖ╛σ║aτÖ╛τoæ.docx]

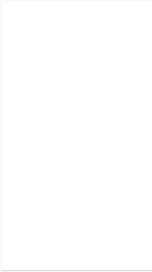

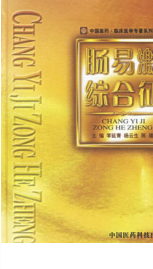

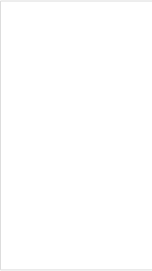

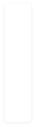

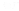
[
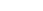
](https://cpro.baidu.com/cpro/ui/uijs.php?en=mywWUA71T1YsFh7sT7qGujYsFhPC5H0huAbqrauGTdq9TZ0qnauJp1d9mHc3mWnsPW0Lnj0LrAnhp1Y-fWb-wbD-fbn-fYD-f1m-wDD-fbc-wWRhUZNopHYknBuVmLKV5HDsrjb1Pzuk5HnLnjbYnHfsgvPsTBuzmWYsFMF15HDhTvN_UANzgv-b5HDhTv-b5H9WnjTLnjmsn103mhDznADhTLwGujY3FMfqIZKWUA-WpvNbndqCmzuYujYkrHbLPWn1FMwV5Hcvrj6sn1R3niuYUgnqnHmLnjb3PH01niuYIHddnHb4P1m1nzud5y9YIZK1FHPKFHFAFHFAmh7GpvR-nbNBmy-bIiRzwyPEUiuv5HchpHYdPAPWmW-Bnf&besl=6&c=news&cf=1&cvrq=1621391&eid_list=202557_208118_208414_209357&expid=202257_202557_202564_203236_206080_206503_208118_209394&fr=20&fv=0&haacp=2073&img_typ=0&itm=0&lu_idc=gzhxy&lukid=12&lus=8c077060308ba20a&lust=63993547&luwtr=14608826967881669318&mscf=0&n=10&nttp=1&p=baidu&pbs=220093&sce=7&sr=72&ssp2=1&tpl=baiduCustITagLinkUnitRankCol&tsf=dtp:1&tu_type=0&u=%2Fitem%2F%25E8%2582%25A0%25E6%2598%2593%25E6%25BF%2580%25E7%25BB%25BC%25E5%2590%2588%25E5%25BE%2581%2F57441595%3FfromModule%3Dlemma%2Dqiyi%5Fsense%2Dlemma&uicf=lurecv&urlid=0&eot=1)[
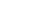
](https://cpro.baidu.com/cpro/ui/uijs.php?en=mywWUA71T1YsFh7sT7qGujYsFhPC5H0huAbqrauGTdq9TZ0qnauJp1d9mHc3mWnsPW0Lnj0LrAnhp1Y-wjD-f1T-f1c-wRf-wjD-fWT-fYf-fbn-fYD-wHbhUZNopHYkFhdWTAYqrj0sPHRhTHY1P104PjDYn7qWTZchThcqnauzT1YkFMP-UAk-T-qGujYkFMPGujY3m10LP10vnjnsrAF9nWK9FMPYpyfqrauY5gwsmvkGmvV-ujPxpAnhIAfqnHb4P1m1nzuYUHYzPW63njndrjDhIAd15HDvP104rjRsn1DhIZRqIHD4rHTvn1nhIHdCIZwsTzR1fiRzwBRzwhF9pyV-FHF7mh7GuZR-nbNWUvYhIWYzFhbqP1DsP1c3m1b&besl=6&c=news&cf=1&cvrq=3335536&eid_list=202557_208118_208414_209357&expid=202257_202557_202564_203236_206080_206503_208118_209394&fr=20&fv=0&haacp=219&img_typ=0&itm=0&lu_idc=gzhxy&lukid=1&lus=8c077060308ba20a&lust=63993547&luwtr=718744168689952326&mscf=0&n=10&nttp=1&p=baidu&pbs=220093&sce=7&sr=72&ssp2=1&tpl=baiduCustITagLinkUnitRankCol&tsf=dtp:1&tu_type=0&u=%2Fitem%2F%25E8%2582%25A0%25E6%2598%2593%25E6%25BF%2580%25E7%25BB%25BC%25E5%2590%2588%25E5%25BE%2581%2F57441595%3FfromModule%3Dlemma%2Dqiyi%5Fsense%2Dlemma&uicf=lurecv&urlid=0&eot=1)[
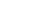
](https://cpro.baidu.com/cpro/ui/uijs.php?en=mywWUA71T1YsFh7sT7qGujYsFhPC5H0huAbqrauGTdq9TZ0qnauJp1d9mHc3mWnsPW0Lnj0LrAnhp1Y-fYf-fbn-fYD-wHb-f1R-wbD-fWT-fHc-fYf-wW6hUZNopHYknzuVmLKV5HT4PjcdFMDqn1TsrHfkPjKxmLKzFMFB5H0hTMnqniu1uyk_ugFxpyfqniu1pyfqrAnsP1TsPW01nj9BmHcsmiu1IA-b5H6hIjdYTAP_pyPouyf1gv9WFMwb5HD4rHTvn1nhIAYqnWm3rj01PH6kFMwVT1YkPWTsrH6dnjnkFMwd5gRkrHbLPWn1FMRqpZwYTZn-nYD-nbm-nbuBmy-ouiRzwyF9pywdFHF7mvqVFMmqnBuG5yDzujI-PyN9&besl=6&c=news&cf=1&cvrq=4468518&eid_list=202557_208118_208414_209357&expid=202257_202557_202564_203236_206080_206503_208118_209394&fr=20&fv=0&haacp=317&img_typ=0&itm=0&lu_idc=gzhxy&lukid=13&lus=8c077060308ba20a&lust=63993547&luwtr=639862747281857404&mscf=0&n=10&nttp=1&p=baidu&pbs=220093&sce=7&sr=72&ssp2=1&tpl=baiduCustITagLinkUnitRankCol&tsf=dtp:1&tu_type=0&u=%2Fitem%2F%25E8%2582%25A0%25E6%2598%2593%25E6%25BF%2580%25E7%25BB%25BC%25E5%2590%2588%25E5%25BE%2581%2F57441595%3FfromModule%3Dlemma%2Dqiyi%5Fsense%2Dlemma&uicf=lurecv&urlid=0&eot=1)[
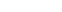
](https://cpro.baidu.com/cpro/ui/uijs.php?en=mywWUA71T1YsFh7sT7qGujYsFhPC5H0huAbqrauGTdq9TZ0qnauJp1d9mHc3mWnsPW0Lnj0LrAnhp1dWTvIEFRPDFRm3FRfdFRF7FRFAFR7KFRPAFRRYFhkdpvbqnBuVmLKV5HDznH04niuk5HnLnjbYnHfsgvPsTBuzmWYsFMF15HDhTvN_UANzgv-b5HDhTv-b5H9WnjTLnjmsn103mhDznADhTLwGujY3FMfqIZKWUA-WpvNbndqCmzuYujYkrHbLPWn1FMwV5Hcvrj6sn1R3niuYUgnqnHmLnjb3PH01niuYIHddnHb4P1m1nzud5y9YIZK1FHPKFHFAFHFAmh7GpvR-nbNBmy-bIiRzwyPEUiuv5HchpHd-myRznj0Lnf&besl=6&c=news&cf=1&cvrq=4489244&eid_list=202557_208118_208414_209357&expid=202257_202557_202564_203236_206080_206503_208118_209394&fr=20&fv=0&haacp=624&img_typ=0&itm=0&lu_idc=gzhxy&lukid=2&lus=8c077060308ba20a&lust=63993547&luwtr=2267704484931445128&mscf=0&n=10&nttp=1&p=baidu&pbs=220093&sce=7&sr=72&ssp2=1&tpl=baiduCustITagLinkUnitRankCol&tsf=dtp:1&tu_type=0&u=%2Fitem%2F%25E8%2582%25A0%25E6%2598%2593%25E6%25BF%2580%25E7%25BB%25BC%25E5%2590%2588%25E5%25BE%2581%2F57441595%3FfromModule%3Dlemma%2Dqiyi%5Fsense%2Dlemma&uicf=lurecv&urlid=0&eot=1)[
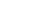
](https://cpro.baidu.com/cpro/ui/uijs.php?en=mywWUA71T1YsFh7sT7qGujYsFhPC5H0huAbqrauGTdq9TZ0qnauJp1d9mHc3mWnsPW0Lnj0LrAnhp1Y-fWb-wbR-fWT-wW0-fWf-wWn-wjD-fHT-f1b-wRD-f1T-wRc-fYn-wWR-fbn-wbRhUZNopHYkPauVmLKV5HbknjDvFMDqn1TsrHfkPjKxmLKzFMFB5H0hTMnqniu1uyk_ugFxpyfqniu1pyfqrAnsP1TsPW01nj9BmHcsmiu1IA-b5H6hIjdYTAP_pyPouyf1gv9WFMwb5HD4rHTvn1nhIAYqnWm3rj01PH6kFMwVT1YkPWTsrH6dnjnkFMwd5gRkrHbLPWn1FMRqpZwYTZn-nYD-nbm-nbuBmy-ouiRzwyF9pywdFHF7mvqVFMmqnBuG5Hu9mHTzrHmv&besl=6&c=news&cf=1&cvrq=2024363&eid_list=202557_208118_208414_209357&expid=202257_202557_202564_203236_206080_206503_208118_209394&fr=20&fv=0&haacp=870&img_typ=0&itm=0&lu_idc=gzhxy&lukid=14&lus=8c077060308ba20a&lust=63993547&luwtr=7841139350953723636&mscf=0&n=10&nttp=1&p=baidu&pbs=220093&sce=7&sr=72&ssp2=1&tpl=baiduCustITagLinkUnitRankCol&tsf=dtp:1&tu_type=0&u=%2Fitem%2F%25E8%2582%25A0%25E6%2598%2593%25E6%25BF%2580%25E7%25BB%25BC%25E5%2590%2588%25E5%25BE%2581%2F57441595%3FfromModule%3Dlemma%2Dqiyi%5Fsense%2Dlemma&uicf=lurecv&urlid=0&eot=1)[
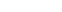
](https://cpro.baidu.com/cpro/ui/uijs.php?en=mywWUA71T1YsFh7sT7qGujYsFhPC5H0huAbqrauGTdq9TZ0qnauJp1d9mHc3mWnsPW0Lnj0LrAnhp1Y-fYf-fbn-fYD-wHb-fWb-wDn-f10-wRf-fYm-fWR-fYf-fWnhUZNopHY1FhdWTAYqnH0YnWn4FMDqn1TsrHfkPjKxmLKzFMFB5H0hTMnqniu1uyk_ugFxpyfqniu1pyfqrAnsP1TsPW01nj9BmHcsmiu1IA-b5H6hIjdYTAP_pyPouyf1gv9WFMwb5HD4rHTvn1nhIAYqnWm3rj01PH6kFMwVT1YkPWTsrH6dnjnkFMwd5gRkrHbLPWn1FMRqpZwYTZn-nYD-nbm-nbuBmy-ouiRzwyF9pywdFHF7mvqVFMmqnBuG5H0znvc4nyNh&besl=6&c=news&cf=1&cvrq=3635363&eid_list=202557_208118_208414_209357&expid=202257_202557_202564_203236_206080_206503_208118_209394&fr=20&fv=0&haacp=640&img_typ=0&itm=0&lu_idc=gzhxy&lukid=3&lus=8c077060308ba20a&lust=63993547&luwtr=2375850642981596205&mscf=0&n=10&nttp=1&p=baidu&pbs=220093&sce=7&sr=72&ssp2=1&tpl=baiduCustITagLinkUnitRankCol&tsf=dtp:1&tu_type=0&u=%2Fitem%2F%25E8%2582%25A0%25E6%2598%2593%25E6%25BF%2580%25E7%25BB%25BC%25E5%2590%2588%25E5%25BE%2581%2F57441595%3FfromModule%3Dlemma%2Dqiyi%5Fsense%2Dlemma&uicf=lurecv&urlid=0&eot=1)[
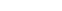
](https://cpro.baidu.com/cpro/ui/uijs.php?en=mywWUA71T1YsFh7sT7qGujYsFhPC5H0huAbqrauGTdq9TZ0qnauJp1d9mHc3mWnsPW0Lnj0LrAnhp1Y-fWn-wWm-fW0-wHm-f1b-wHT-wjT-wjf-fWT-wjD-fWn-wWm-fYD-wHbhUZNopHYYFhdWTAYqnHDsPHnvFMDqn1TsrHfkPjKxmLKzFMFB5H0hTMnqniu1uyk_ugFxpyfqniu1pyfqrAnsP1TsPW01nj9BmHcsmiu1IA-b5H6hIjdYTAP_pyPouyf1gv9WFMwb5HD4rHTvn1nhIAYqnWm3rj01PH6kFMwVT1YkPWTsrH6dnjnkFMwd5gRkrHbLPWn1FMRqpZwYTZn-nYD-nbm-nbuBmy-ouiRzwyF9pywdFHF7mvqVFMmqnBuG5yckuWTduj6Y&besl=6&c=news&cf=1&cvrq=7189029&eid_list=202557_208118_208414_209357&expid=202257_202557_202564_203236_206080_206503_208118_209394&fr=20&fv=0&haacp=618&img_typ=0&itm=0&lu_idc=gzhxy&lukid=4&lus=8c077060308ba20a&lust=63993547&luwtr=7476068094432150857&mscf=0&n=10&nttp=1&p=baidu&pbs=220093&sce=7&sr=72&ssp2=1&tpl=baiduCustITagLinkUnitRankCol&tsf=dtp:1&tu_type=0&u=%2Fitem%2F%25E8%2582%25A0%25E6%2598%2593%25E6%25BF%2580%25E7%25BB%25BC%25E5%2590%2588%25E5%25BE%2581%2F57441595%3FfromModule%3Dlemma%2Dqiyi%5Fsense%2Dlemma&uicf=lurecv&urlid=0&eot=1)[
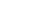
](https://cpro.baidu.com/cpro/ui/uijs.php?en=mywWUA71T1YsFh7sT7qGujYsFhPC5H0huAbqrauGTdq9TZ0qnauJp1d9mHc3mWnsPW0Lnj0LrAnhp1dvTBNjwBNAfBNaPzNjnaNDniNDwaNjniNaPzu_IyVG5HDdFhdWTAYqnHnLrjnYFMDqn1TsrHfkPjKxmLKzFMFB5H0hTMnqniu1uyk_ugFxpyfqniu1pyfqrAnsP1TsPW01nj9BmHcsmiu1IA-b5H6hIjdYTAP_pyPouyf1gv9WFMwb5HD4rHTvn1nhIAYqnWm3rj01PH6kFMwVT1YkPWTsrH6dnjnkFMwd5gRkrHbLPWn1FMRqpZwYTZn-nYD-nbm-nbuBmy-ouiRzwyF9pywdFHF7mvqVFMmqnBuG5yP9nvDzuWPW&besl=6&c=news&cf=1&cvrq=4705749&eid_list=202557_208118_208414_209357&expid=202257_202557_202564_203236_206080_206503_208118_209394&fr=20&fv=0&haacp=1024&img_typ=0&itm=0&lu_idc=gzhxy&lukid=15&lus=8c077060308ba20a&lust=63993547&luwtr=754962655298315806&mscf=0&n=10&nttp=1&p=baidu&pbs=220093&sce=7&sr=72&ssp2=1&tpl=baiduCustITagLinkUnitRankCol&tsf=dtp:1&tu_type=0&u=%2Fitem%2F%25E8%2582%25A0%25E6%2598%2593%25E6%25BF%2580%25E7%25BB%25BC%25E5%2590%2588%25E5%25BE%2581%2F57441595%3FfromModule%3Dlemma%2Dqiyi%5Fsense%2Dlemma&uicf=lurecv&urlid=0&eot=1)[
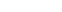
](https://cpro.baidu.com/cpro/ui/uijs.php?en=mywWUA71T1YsFh7sT7qGujYsFhPC5H0huAbqrauGTdq9TZ0qnauJp1d9mHc3mWnsPW0Lnj0LrAnhp1Y-wjT-wjf-fbn-fbD-fWf-fWf-fbf-fH6-fW6-wWm-fYf-wW6-wjR-fbRhUZNopHYdFhdWTAYqnHc1nWf1FMDqn1TsrHfkPjKxmLKzFMFB5H0hTMnqniu1uyk_ugFxpyfqniu1pyfqrAnsP1TsPW01nj9BmHcsmiu1IA-b5H6hIjdYTAP_pyPouyf1gv9WFMwb5HD4rHTvn1nhIAYqnWm3rj01PH6kFMwVT1YkPWTsrH6dnjnkFMwd5gRkrHbLPWn1FMRqpZwYTZn-nYD-nbm-nbuBmy-ouiRzwyF9pywdFHF7mvqVFMmqnBuG5yfLuj0Yrjms&besl=6&c=news&cf=1&cvrq=2235876&eid_list=202557_208118_208414_209357&expid=202257_202557_202564_203236_206080_206503_208118_209394&fr=20&fv=0&haacp=1001&img_typ=0&itm=0&lu_idc=gzhxy&lukid=5&lus=8c077060308ba20a&lust=63993547&luwtr=6556233252601032990&mscf=0&n=10&nttp=1&p=baidu&pbs=220093&sce=7&sr=72&ssp2=1&tpl=baiduCustITagLinkUnitRankCol&tsf=dtp:1&tu_type=0&u=%2Fitem%2F%25E8%2582%25A0%25E6%2598%2593%25E6%25BF%2580%25E7%25BB%25BC%25E5%2590%2588%25E5%25BE%2581%2F57441595%3FfromModule%3Dlemma%2Dqiyi%5Fsense%2Dlemma&uicf=lurecv&urlid=0&eot=1)[
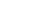
](https://cpro.baidu.com/cpro/ui/uijs.php?en=mywWUA71T1YsFh7sT7qGujYsFhPC5H0huAbqrauGTdq9TZ0qnauJp1d9mHc3mWnsPW0Lnj0LrAnhp1Y-wj0-wHb-f1f-wHc-fbc-wWR-fWD-wjc-f1m-fbf-fYn-fH6hUZNopHYkPBuVmLKV5HT3PWRYFMDqn1TsrHfkPjKxmLKzFMFB5H0hTMnqniu1uyk_ugFxpyfqniu1pyfqrAnsP1TsPW01nj9BmHcsmiu1IA-b5H6hIjdYTAP_pyPouyf1gv9WFMwb5HD4rHTvn1nhIAYqnWm3rj01PH6kFMwVT1YkPWTsrH6dnjnkFMwd5gRkrHbLPWn1FMRqpZwYTZn-nYD-nbm-nbuBmy-ouiRzwyF9pywdFHF7mvqVFMmqnBuG5HndPWn1rjRz&besl=6&c=news&cf=1&cvrq=3011782&eid_list=202557_208118_208414_209357&expid=202257_202557_202564_203236_206080_206503_208118_209394&fr=20&fv=0&haacp=194&img_typ=0&itm=0&lu_idc=gzhxy&lukid=16&lus=8c077060308ba20a&lust=63993547&luwtr=2314272910775226794&mscf=0&n=10&nttp=1&p=baidu&pbs=220093&sce=7&sr=72&ssp2=1&tpl=baiduCustITagLinkUnitRankCol&tsf=dtp:1&tu_type=0&u=%2Fitem%2F%25E8%2582%25A0%25E6%2598%2593%25E6%25BF%2580%25E7%25BB%25BC%25E5%2590%2588%25E5%25BE%2581%2F57441595%3FfromModule%3Dlemma%2Dqiyi%5Fsense%2Dlemma&uicf=lurecv&urlid=0&eot=1)[
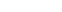
](https://cpro.baidu.com/cpro/ui/uijs.php?en=mywWUA71T1YsFh7sT7qGujYsFhPC5H0huAbqrauGTdq9TZ0qnauJp1d9mHc3mWnsPW0Lnj0LrAnhp1Y-wjR-fbf-fWm-wjP_UvIEFRn4FRR3FRFjFRnvFhkdpvbqPBuVmLKV5HbdnWnYFMDqn1TsrHfkPjKxmLKzFMFB5H0hTMnqniu1uyk_ugFxpyfqniu1pyfqrAnsP1TsPW01nj9BmHcsmiu1IA-b5H6hIjdYTAP_pyPouyf1gv9WFMwb5HD4rHTvn1nhIAYqnWm3rj01PH6kFMwVT1YkPWTsrH6dnjnkFMwd5gRkrHbLPWn1FMRqpZwYTZn-nYD-nbm-nbuBmy-ouiRzwyF9pywdFHF7mvqVFMmqnBuG5HnvnHDYnhn3&besl=6&c=news&cf=1&cvrq=1756705&eid_list=202557_208118_208414_209357&expid=202257_202557_202564_203236_206080_206503_208118_209394&fr=20&fv=0&haacp=707&img_typ=0&itm=0&lu_idc=gzhxy&lukid=6&lus=8c077060308ba20a&lust=63993547&luwtr=1863292750894598650&mscf=0&n=10&nttp=1&p=baidu&pbs=220093&sce=7&sr=72&ssp2=1&tpl=baiduCustITagLinkUnitRankCol&tsf=dtp:1&tu_type=0&u=%2Fitem%2F%25E8%2582%25A0%25E6%2598%2593%25E6%25BF%2580%25E7%25BB%25BC%25E5%2590%2588%25E5%25BE%2581%2F57441595%3FfromModule%3Dlemma%2Dqiyi%5Fsense%2Dlemma&uicf=lurecv&urlid=0&eot=1)[
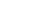
](https://cpro.baidu.com/cpro/ui/uijs.php?en=mywWUA71T1YsFh7sT7qGujYsFhPC5H0huAbqrauGTdq9TZ0qnauJp1d9mHc3mWnsPW0Lnj0LrAnhp1Y-fYR-wDR-f16-fYc-fbc-wbD-fWT-fWf-wjm-f1mhUZNopHYkPzuVmLKV5H6sP1bvFMDqn1TsrHfkPjKxmLKzFMFB5H0hTMnqniu1uyk_ugFxpyfqniu1pyfqrAnsP1TsPW01nj9BmHcsmiu1IA-b5H6hIjdYTAP_pyPouyf1gv9WFMwb5HD4rHTvn1nhIAYqnWm3rj01PH6kFMwVT1YkPWTsrH6dnjnkFMwd5gRkrHbLPWn1FMRqpZwYTZn-nYD-nbm-nbuBmy-ouiRzwyF9pywdFHF7mvqVFMmqnBuG5yw-ujNhnAP9&besl=6&c=news&cf=1&cvrq=1415934&eid_list=202557_208118_208414_209357&expid=202257_202557_202564_203236_206080_206503_208118_209394&fr=20&fv=0&haacp=904&img_typ=0&itm=0&lu_idc=gzhxy&lukid=17&lus=8c077060308ba20a&lust=63993547&luwtr=685006414410405008&mscf=0&n=10&nttp=1&p=baidu&pbs=220093&sce=7&sr=72&ssp2=1&tpl=baiduCustITagLinkUnitRankCol&tsf=dtp:1&tu_type=0&u=%2Fitem%2F%25E8%2582%25A0%25E6%2598%2593%25E6%25BF%2580%25E7%25BB%25BC%25E5%2590%2588%25E5%25BE%2581%2F57441595%3FfromModule%3Dlemma%2Dqiyi%5Fsense%2Dlemma&uicf=lurecv&urlid=0&eot=1)[
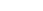
](https://cpro.baidu.com/cpro/ui/uijs.php?en=mywWUA71T1YsFh7sT7qGujYsFhPC5H0huAbqrauGTdq9TZ0qnauJp1d9mHc3mWnsPW0Lnj0LrAnhp1Y-wjn-fYR-fYm-fWT-fbD-wj0-wjT-wjnhUZNopHYLFhdWTAYqP1c4njRhTHY1P104PjDYn7qWTZchThcqnauzT1YkFMP-UAk-T-qGujYkFMPGujY3m10LP10vnjnsrAF9nWK9FMPYpyfqrauY5gwsmvkGmvV-ujPxpAnhIAfqnHb4P1m1nzuYUHYzPW63njndrjDhIAd15HDvP104rjRsn1DhIZRqIHD4rHTvn1nhIHdCIZwsTzR1fiRzwBRzwhF9pyV-FHF7mh7GuZR-nbNWUvYhIWYzFhbqnWuhnhwhPhR&besl=6&c=news&cf=1&cvrq=3228426&eid_list=202557_208118_208414_209357&expid=202257_202557_202564_203236_206080_206503_208118_209394&fr=20&fv=0&haacp=271&img_typ=0&itm=0&lu_idc=gzhxy&lukid=7&lus=8c077060308ba20a&lust=63993547&luwtr=13245032438307475642&mscf=0&n=10&nttp=1&p=baidu&pbs=220093&sce=7&sr=72&ssp2=1&tpl=baiduCustITagLinkUnitRankCol&tsf=dtp:1&tu_type=0&u=%2Fitem%2F%25E8%2582%25A0%25E6%2598%2593%25E6%25BF%2580%25E7%25BB%25BC%25E5%2590%2588%25E5%25BE%2581%2F57441595%3FfromModule%3Dlemma%2Dqiyi%5Fsense%2Dlemma&uicf=lurecv&urlid=0&eot=1)[
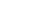
](https://cpro.baidu.com/cpro/ui/uijs.php?en=mywWUA71T1YsFh7sT7qGujYsFhPC5H0huAbqrauGTdq9TZ0qnauJp1d9mHc3mWnsPW0Lnj0LrAnhp1Y-fWR-wHT-f1b-fYn-f1m-fbf-fYn-fH6-wjf-wWR-f1n-fWf-wjT-wWmhUZNopHYkrauVmLKV5H6sP1T3FMDqn1TsrHfkPjKxmLKzFMFB5H0hTMnqniu1uyk_ugFxpyfqniu1pyfqrAnsP1TsPW01nj9BmHcsmiu1IA-b5H6hIjdYTAP_pyPouyf1gv9WFMwb5HD4rHTvn1nhIAYqnWm3rj01PH6kFMwVT1YkPWTsrH6dnjnkFMwd5gRkrHbLPWn1FMRqpZwYTZn-nYD-nbm-nbuBmy-ouiRzwyF9pywdFHF7mvqVFMmqnBuG5HmLPW04rHw9&besl=6&c=news&cf=1&cvrq=3247861&eid_list=202557_208118_208414_209357&expid=202257_202557_202564_203236_206080_206503_208118_209394&fr=20&fv=0&haacp=3601&img_typ=0&itm=0&lu_idc=gzhxy&lukid=18&lus=8c077060308ba20a&lust=63993547&luwtr=7777852140969087504&mscf=0&n=10&nttp=1&p=baidu&pbs=220093&sce=7&sr=72&ssp2=1&tpl=baiduCustITagLinkUnitRankCol&tsf=dtp:1&tu_type=0&u=%2Fitem%2F%25E8%2582%25A0%25E6%2598%2593%25E6%25BF%2580%25E7%25BB%25BC%25E5%2590%2588%25E5%25BE%2581%2F57441595%3FfromModule%3Dlemma%2Dqiyi%5Fsense%2Dlemma&uicf=lurecv&urlid=0&eot=1)[
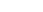
](https://cpro.baidu.com/cpro/ui/uijs.php?en=mywWUA71T1YsFh7sT7qGujYsFhPC5H0huAbqrauGTdq9TZ0qnauJp1d9mHc3mWnsPW0Lnj0LrAnhp1Y-fWb-fbD-f1c-wWc-wjn-wWc-f1n-wbchUZNopHY3FhdWTAYqrHfznjThTHY1P104PjDYn7qWTZchThcqnauzT1YkFMP-UAk-T-qGujYkFMPGujY3m10LP10vnjnsrAF9nWK9FMPYpyfqrauY5gwsmvkGmvV-ujPxpAnhIAfqnHb4P1m1nzuYUHYzPW63njndrjDhIAd15HDvP104rjRsn1DhIZRqIHD4rHTvn1nhIHdCIZwsTzR1fiRzwBRzwhF9pyV-FHF7mh7GuZR-nbNWUvYhIWYzFhbquH0krj7brHD&besl=6&c=news&cf=1&cvrq=1941531&eid_list=202557_208118_208414_209357&expid=202257_202557_202564_203236_206080_206503_208118_209394&fr=20&fv=0&haacp=1286&img_typ=0&itm=0&lu_idc=gzhxy&lukid=8&lus=8c077060308ba20a&lust=63993547&luwtr=17492749210616066771&mscf=0&n=10&nttp=1&p=baidu&pbs=220093&sce=7&sr=72&ssp2=1&tpl=baiduCustITagLinkUnitRankCol&tsf=dtp:1&tu_type=0&u=%2Fitem%2F%25E8%2582%25A0%25E6%2598%2593%25E6%25BF%2580%25E7%25BB%25BC%25E5%2590%2588%25E5%25BE%2581%2F57441595%3FfromModule%3Dlemma%2Dqiyi%5Fsense%2Dlemma&uicf=lurecv&urlid=0&eot=1)[
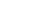
](https://cpro.baidu.com/cpro/ui/uijs.php?en=mywWUA71T1YsFh7sT7qGujYsFhPC5H0huAbqrauGTdq9TZ0qnauJp1d9mHc3mWnsPW0Lnj0LrAnhp1Y-fYf-wW6-f1c-wHT-fW0-fWc-f16-fRc-f1R-wH0-wjD-fWRhUZNopHYkriuVmLKV5HDznjmLniuk5HnLnjbYnHfsgvPsTBuzmWYsFMF15HDhTvN_UANzgv-b5HDhTv-b5H9WnjTLnjmsn103mhDznADhTLwGujY3FMfqIZKWUA-WpvNbndqCmzuYujYkrHbLPWn1FMwV5Hcvrj6sn1R3niuYUgnqnHmLnjb3PH01niuYIHddnHb4P1m1nzud5y9YIZK1FHPKFHFAFHFAmh7GpvR-nbNBmy-bIiRzwyPEUiuv5HchpHYzn1fYmHPbu6&besl=6&c=news&cf=1&cvrq=3292150&eid_list=202557_208118_208414_209357&expid=202257_202557_202564_203236_206080_206503_208118_209394&fr=20&fv=0&haacp=1162&img_typ=0&itm=0&lu_idc=gzhxy&lukid=19&lus=8c077060308ba20a&lust=63993547&luwtr=2249689388344567100&mscf=0&n=10&nttp=1&p=baidu&pbs=220093&sce=7&sr=72&ssp2=1&tpl=baiduCustITagLinkUnitRankCol&tsf=dtp:1&tu_type=0&u=%2Fitem%2F%25E8%2582%25A0%25E6%2598%2593%25E6%25BF%2580%25E7%25BB%25BC%25E5%2590%2588%25E5%25BE%2581%2F57441595%3FfromModule%3Dlemma%2Dqiyi%5Fsense%2Dlemma&uicf=lurecv&urlid=0&eot=1)[
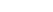
](https://cpro.baidu.com/cpro/ui/uijs.php?en=mywWUA71T1YsFh7sT7qGujYsFhPC5H0huAbqrauGTdq9TZ0qnauJp1d9mHc3mWnsPW0Lnj0LrAnhp1Y1PzNDnzNjwiNjwBNaPzNjPBNawaNjfzNKrau_IyVG5HbhUyPsUHY4nWnsnauk5HnLnjbYnHfsgvPsTBuzmWYsFMF15HDhTvN_UANzgv-b5HDhTv-b5H9WnjTLnjmsn103mhDznADhTLwGujY3FMfqIZKWUA-WpvNbndqCmzuYujYkrHbLPWn1FMwV5Hcvrj6sn1R3niuYUgnqnHmLnjb3PH01niuYIHddnHb4P1m1nzud5y9YIZK1FHPKFHFAFHFAmh7GpvR-nbNBmy-bIiRzwyPEUiuv5HchpHY1rHDvmyFBPf&besl=6&c=news&cf=1&cvrq=3623810&eid_list=202557_208118_208414_209357&expid=202257_202557_202564_203236_206080_206503_208118_209394&fr=20&fv=0&haacp=188&img_typ=0&itm=0&lu_idc=gzhxy&lukid=9&lus=8c077060308ba20a&lust=63993547&luwtr=676781759077005289&mscf=0&n=10&nttp=1&p=baidu&pbs=220093&sce=7&sr=72&ssp2=1&tpl=baiduCustITagLinkUnitRankCol&tsf=dtp:1&tu_type=0&u=%2Fitem%2F%25E8%2582%25A0%25E6%2598%2593%25E6%25BF%2580%25E7%25BB%25BC%25E5%2590%2588%25E5%25BE%2581%2F57441595%3FfromModule%3Dlemma%2Dqiyi%5Fsense%2Dlemma&uicf=lurecv&urlid=0&eot=1)[
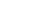
](https://cpro.baidu.com/cpro/ui/uijs.php?en=mywWUA71T1YsFh7sT7qGujYsFhPC5H0huAbqrauGTdq9TZ0qnauJp1d9mHc3mWnsPW0Lnj0LrAnhp1Y-fYf-wW6-f1c-wHT-fWb-fHf-fWn-fYn-fYD-fHmhUZNopHYznauVmLKV5HD1nHD3nauk5HnLnjbYnHfsgvPsTBuzmWYsFMF15HDhTvN_UANzgv-b5HDhTv-b5H9WnjTLnjmsn103mhDznADhTLwGujY3FMfqIZKWUA-WpvNbndqCmzuYujYkrHbLPWn1FMwV5Hcvrj6sn1R3niuYUgnqnHmLnjb3PH01niuYIHddnHb4P1m1nzud5y9YIZK1FHPKFHFAFHFAmh7GpvR-nbNBmy-bIiRzwyPEUiuv5HchpHY3uW03nWcLms&besl=6&c=news&cf=1&cvrq=3661677&eid_list=202557_208118_208414_209357&expid=202257_202557_202564_203236_206080_206503_208118_209394&fr=20&fv=0&haacp=1439&img_typ=0&itm=0&lu_idc=gzhxy&lukid=20&lus=8c077060308ba20a&lust=63993547&luwtr=750531414731215287&mscf=0&n=10&nttp=1&p=baidu&pbs=220093&sce=7&sr=72&ssp2=1&tpl=baiduCustITagLinkUnitRankCol&tsf=dtp:1&tu_type=0&u=%2Fitem%2F%25E8%2582%25A0%25E6%2598%2593%25E6%25BF%2580%25E7%25BB%25BC%25E5%2590%2588%25E5%25BE%2581%2F57441595%3FfromModule%3Dlemma%2Dqiyi%5Fsense%2Dlemma&uicf=lurecv&urlid=0&eot=1)[
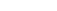
](https://cpro.baidu.com/cpro/ui/uijs.php?en=mywWUA71T1YsFh7sT7qGujYsFhPC5H0huAbqrauGTdq9TZ0qnauJp1d9mHc3mWnsPW0Lnj0LrAnhp1d1mvb-f1c-wDc-fYR-f1f-fYf-fWm-fW6-wHRhUZNopHYknauVmLKV5HDzrjc3niuk5HnLnjbYnHfsgvPsTBuzmWYsFMF15HDhTvN_UANzgv-b5HDhTv-b5H9WnjTLnjmsn103mhDznADhTLwGujY3FMfqIZKWUA-WpvNbndqCmzuYujYkrHbLPWn1FMwV5Hcvrj6sn1R3niuYUgnqnHmLnjb3PH01niuYIHddnHb4P1m1nzud5y9YIZK1FHPKFHFAFHFAmh7GpvR-nbNBmy-bIiRzwyPEUiuv5HchpHdBryRsPWb1n6&besl=6&c=news&cf=1&cvrq=3196467&eid_list=202557_208118_208414_209357&expid=202257_202557_202564_203236_206080_206503_208118_209394&fr=20&fv=0&haacp=1032&img_typ=0&itm=0&lu_idc=gzhxy&lukid=10&lus=8c077060308ba20a&lust=63993547&luwtr=2480674890665447709&mscf=0&n=10&nttp=1&p=baidu&pbs=220093&sce=7&sr=72&ssp2=1&tpl=baiduCustITagLinkUnitRankCol&tsf=dtp:1&tu_type=0&u=%2Fitem%2F%25E8%2582%25A0%25E6%2598%2593%25E6%25BF%2580%25E7%25BB%25BC%25E5%2590%2588%25E5%25BE%2581%2F57441595%3FfromModule%3Dlemma%2Dqiyi%5Fsense%2Dlemma&uicf=lurecv&urlid=0&eot=1)[
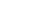
](https://cpro.baidu.com/cpro/ui/uijs.php?en=mywWUA71T1YsFh7sT7qGujYsFhPC5H0huAbqrauGTdq9TZ0qnauJp1d9mHc3mWnsPW0Lnj0LrAnhp1Y-wjf-wWR-f1n-fWf-fWf-fWf-fbf-fH6-wj0-fHD-fWn-fYn-wj0-wWchUZNopHYzniuVmLKV5H61PWDkFMDqn1TsrHfkPjKxmLKzFMFB5H0hTMnqniu1uyk_ugFxpyfqniu1pyfqrAnsP1TsPW01nj9BmHcsmiu1IA-b5H6hIjdYTAP_pyPouyf1gv9WFMwb5HD4rHTvn1nhIAYqnWm3rj01PH6kFMwVT1YkPWTsrH6dnjnkFMwd5gRkrHbLPWn1FMRqpZwYTZn-nYD-nbm-nbuBmy-ouiRzwyF9pywdFHF7mvqVFMmqnBuG5H64uh7WuH0d&besl=6&c=news&cf=1&cvrq=2189719&eid_list=202557_208118_208414_209357&expid=202257_202557_202564_203236_206080_206503_208118_209394&fr=20&fv=0&haacp=734&img_typ=0&itm=0&lu_idc=gzhxy&lukid=21&lus=8c077060308ba20a&lust=63993547&luwtr=7550494890368818072&mscf=0&n=10&nttp=1&p=baidu&pbs=220093&sce=7&sr=72&ssp2=1&tpl=baiduCustITagLinkUnitRankCol&tsf=dtp:1&tu_type=0&u=%2Fitem%2F%25E8%2582%25A0%25E6%2598%2593%25E6%25BF%2580%25E7%25BB%25BC%25E5%2590%2588%25E5%25BE%2581%2F57441595%3FfromModule%3Dlemma%2Dqiyi%5Fsense%2Dlemma&uicf=lurecv&urlid=0&eot=1)[
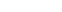
](https://cpro.baidu.com/cpro/ui/uijs.php?en=mywWUA71T1YsFh7sT7qGujYsFhPC5H0huAbqrauGTdq9TZ0qnauJp1d9mHc3mWnsPW0Lnj0LrAnhp1dWTvIEFRcdFRRLFRnYFRfYFRndFRRYFRfvFRn1FhkdpvbqnHDhUyPsUHYLrjcsniuk5HnLnjbYnHfsgvPsTBuzmWYsFMF15HDhTvN_UANzgv-b5HDhTv-b5H9WnjTLnjmsn103mhDznADhTLwGujY3FMfqIZKWUA-WpvNbndqCmzuYujYkrHbLPWn1FMwV5Hcvrj6sn1R3niuYUgnqnHmLnjb3PH01niuYIHddnHb4P1m1nzud5y9YIZK1FHPKFHFAFHFAmh7GpvR-nbNBmy-bIiRzwyPEUiuv5HchpHYkuANhmyckuf&besl=6&c=news&cf=1&cvrq=3472465&eid_list=202557_208118_208414_209357&expid=202257_202557_202564_203236_206080_206503_208118_209394&fr=20&fv=0&haacp=611&img_typ=0&itm=0&lu_idc=gzhxy&lukid=11&lus=8c077060308ba20a&lust=63993547&luwtr=2357911270216075011&mscf=0&n=10&nttp=1&p=baidu&pbs=220093&sce=7&sr=72&ssp2=1&tpl=baiduCustITagLinkUnitRankCol&tsf=dtp:1&tu_type=0&u=%2Fitem%2F%25E8%2582%25A0%25E6%2598%2593%25E6%25BF%2580%25E7%25BB%25BC%25E5%2590%2588%25E5%25BE%2581%2F57441595%3FfromModule%3Dlemma%2Dqiyi%5Fsense%2Dlemma&uicf=lurecv&urlid=0&eot=1)[
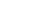
](https://cpro.baidu.com/cpro/ui/uijs.php?en=mywWUA71T1YsFh7sT7qGujYsFhPC5H0huAbqrauGTdq9TZ0qnauJp1d9mHc3mWnsPW0Lnj0LrAnhp1Y-fbn-wDc-fW6-wWD-fWD-wHn-wjc-fYc-fWR-f1f-fYm-wHn-fW6-wDc-fWT-wbR-fYR-wWD-f1m-wWThUZNopHYznBuVmLKV5HT4nW6sFMDqn1TsrHfkPjKxmLKzFMFB5H0hTMnqniu1uyk_ugFxpyfqniu1pyfqrAnsP1TsPW01nj9BmHcsmiu1IA-b5H6hIjdYTAP_pyPouyf1gv9WFMwb5HD4rHTvn1nhIAYqnWm3rj01PH6kFMwVT1YkPWTsrH6dnjnkFMwd5gRkrHbLPWn1FMRqpZwYTZn-nYD-nbm-nbuBmy-ouiRzwyF9pywdFHF7mvqVFMmqnBuG5yP-nhfzmWFb&besl=6&c=news&cf=1&cvrq=2363941&eid_list=202557_208118_208414_209357&expid=202257_202557_202564_203236_206080_206503_208118_209394&fr=20&fv=0&haacp=1750&img_typ=0&itm=0&lu_idc=gzhxy&lukid=22&lus=8c077060308ba20a&lust=63993547&luwtr=18274635138362951998&mscf=0&n=10&nttp=1&p=baidu&pbs=220093&sce=7&sr=72&ssp2=1&tpl=baiduCustITagLinkUnitRankCol&tsf=dtp:1&tu_type=0&u=%2Fitem%2F%25E8%2582%25A0%25E6%2598%2593%25E6%25BF%2580%25E7%25BB%25BC%25E5%2590%2588%25E5%25BE%2581%2F57441595%3FfromModule%3Dlemma%2Dqiyi%5Fsense%2Dlemma&uicf=lurecv&urlid=0&eot=1)
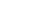

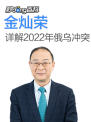
2022/12/14 10:30

[肠易激综合征的概述图(1](https://baike.baidu.com/pic/%E8%82%A0%E6%98%93%E6%BF%80%E7%BB%BC%E5%90%88%E5%BE%81/57441595/1/21a4462309f7905298222d47c9bcc0ca7bcb0a467f30?fr=lemma&fromModule=lemma_top-image&ct=single)

[女](javascript:void(0);)

[
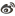

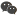
疊口](javascript:void(0);)

| **词条统计**  浏览次数： 316次  编辑次数： 2次[历史版本](https://baike.baidu.com/historylist/%E8%82%A0%E6%98%93%E6%BF%80%E7%BB%BC%E5%90%88%E5%BE%81/57441595)  最近更新： [一如既往11024](https://baike.baidu.com/usercenter/userpage?uk=f3h5jF5KHfPk8ktgAGlalA&from=lemma) ( 2021-08-2 |
| --- |

| **1** 亚马逊图书 **12** 国际期货  **2** csgo网站开箱 **13** 图书批发网  **3** 图书管理系统 **14** 哈佛大学申  **4** 出版社自费出 **15** vr消防演练  **5** 自己创建个网 **16** 虚拟货币平  **6** 战队logo设计 **17** 无人机反制  **7** 游戏盒子 **18** 电商平台怎  **8** 购买域名 **19** 网络安全培  **9** 37游戏平台 **20** 网络工程师  **10** sci论文投稿 **21** 怎么创建小  **11** csgo电脑配置 **22** 价格便宜的 |
| --- |



 [肠应激性综合症](https://www.baidu.com/s?word=%E8%82%A0%E5%BA%94%E6%BF%80%E6%80%A7%E7%BB%BC%E5%90%88%E7%97%87&tn=SE_baikepcxf02_fcetbk02&pos=baike_pc_turbo_1767&ori_sid=00bb347c6d29da4f)[
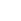
](http://baike.baidu.com/l/WWoXYu7P)

1/2

肠易激综合征(2005年中国医药科技出版社出版的图书)

[**网页**](https://www.baidu.com/) [**新闻**](http://news.baidu.com/) [**贴吧**](https://tieba.baidu.com/) [**知道**](https://zhidao.baidu.com/) [**网盘**](https://pan.baidu.com/?from=1027327l) [**图片**](http://image.baidu.com/) [**视频**](http://v.baidu.com/)

_百度百科

[**地图**](http://map.baidu.com/) [**文库**](https://wenku.baidu.com/)

**百科**


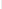
 [百度首页](http://www.baidu.com/) [登录](javascript:;)

| [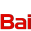岔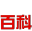](https://baike.baidu.com/) | | 肠易激综合征 | 进入词条 | | --- | --- | | | 全站搜索 | | --- | | [帮助](https://baike.baidu.com/help) | |
| --- | --- | --- | --- | --- | --- | --- | --- |
| 近期有不法分子冒充百度百科官方人员，以删除词条为由威胁并敲诈相关企业。在此严正声明：百度百科是免费编辑平台，绝不存在收费代编服务，请勿上当受骗！ [详情>>](https://baike.baidu.com/common/declaration) | | | | |
| [首页](https://baike.baidu.com/) 秒懂百科 特色百科 用户 知识专题 权威合作 | | [口下载百科APP](https://baike.baidu.com/wapui/subpage/baikeappdownload?sfrom=pc_lemmapage_navigation) | | 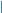 [2 个](https://baike.baidu.com/usercenter) |

| **肠易激综合征**是一个[多义词](https://baike.baidu.com/item/%E7%99%BE%E5%BA%A6%E7%99%BE%E7%A7%91%EF%BC%9A%E5%A4%9A%E4%B9%89%E8%AF%8D)，请在下列[义项](https://baike.baidu.com/item/%E4%B9%89%E9%A1%B9)上选择浏览([共3个义项](https://baike.baidu.com/item/%E8%82%A0%E6%98%93%E6%BF%80%E7%BB%BC%E5%90%88%E5%BE%81?force=1)) | 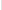[添加义项 +](javascript:;) | |
| --- | --- | --- |
| ▪ [肠易激综合征](https://baike.baidu.com/item/%E8%82%A0%E6%98%93%E6%BF%80%E7%BB%BC%E5%90%88%E5%BE%81/8456761?fromModule=lemma_sense-layer#viewPageContent) | ▪ **2005年中国医药科技出版社出版的图书** | ▪ [2015年中国中医药出版社出版的图书](https://baike.baidu.com/item/%E8%82%A0%E6%98%93%E6%BF%80%E7%BB%BC%E5%90%88%E5%BE%81/60035105?fromModule=lemma_sense-layer#viewPageContent) |

| 肠易激综合征   | [小播报](javascript:;) | | --- |   2005年中国医药科技出版社出版的图书 | | | | [c编辑](javascript:;) | | --- | | | O讨论 | | --- |   [1](https://baike.baidu.com/planet/talk?lemmaId=57441595&fromModule=lemma_right-issue-btn)   | [训上传视频](javascript:;) | | --- | | | | | . 收藏 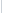[山 0](javascript:void(0);)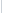 吐 0 |
| --- | --- | --- | --- | --- | --- | --- | --- | --- | --- | --- | --- | --- |
| 《肠易激综合征》是中国医药科技出版社出版的图书，作者是李延青，杨云生，陈建 | | | | | | | [1] | |
| **书** **作** | **名** **者** | 肠易激综合征 中国医药临床医学专著系列 李延青、杨云生、陈建 | | | **出版社**  **定** **价**  **ISBN** | 中国医药科技出版社  69.38 元  9787506731010 | | |
| [服务由京东提供](https://item.jd.com/10065776492390.html) 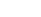 [购买](https://item.jd.com/10065776492390.html)   | [**正版** **肠易激综合征** **李延青,杨云生,陈建** **主** **…**](https://item.jd.com/10065776492390.html)  [**￥43.00**](https://item.jd.com/10065776492390.html) | | --- |  | [**正品！肠易激综合征**](https://item.jd.com/10065145372624.html) 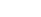 [购买](https://item.jd.com/10065145372624.html)  [**￥51.00**](https://item.jd.com/10065145372624.html)  [服务由京东提供](https://item.jd.com/10065145372624.html) | | --- | | | | | | | | | |
| [词条图册 更多图册 >](https://baike.baidu.com/pic/%E8%82%A0%E6%98%93%E6%BF%80%E7%BB%BC%E5%90%88%E5%BE%81/57441595?fr=lemma) | | | | | | | | |
| | 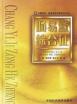 | | --- | | 概述图册(1)  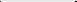 | | | | | | | | | |
| 参考资料 | | | | | | | | |
| 1  [肠易激综合征 会](https://baike.baidu.com/reference/57441595/72e0vNrnxyn1ZxesLkjab9wCM6cXtv8JWJimckMzkNbvxaP7lK3vcZEXoXqy-j8alVJWUPEEomiyVDpqjN_MGw5G0W8) ．京东[引用日期2021-06-22] | | | | | | | | |

| 猜你喜欢 | [幽门螺旋杆菌，吃益生菌有用吗?高活性益生菌](http://www.baidu.com/baidu.php?url=Ks00000EAMrnlPLIyBguLNDNsZEIMS-UyqeAixykX3luIB2WRWdJiLFFfXSgFOnVxOaesUoPzJFe7fqTTWvZGWwBjulXL2tfUSJDDGur85aL2EmvD6pTRsaa8vCo75k3suYKbg2E86kcFPVU_ZyxvtWNTReSh-Ov_Kn_54hRatQcPNen3LJyDaw6WwIGF9L7wpD0Bs9KVX7x_zfBzB6IP8TjzqTb.7D_NR2Ar5Od66xfHGt_Vzc2eQr1k_lX1uEooo3tdPHV2XgZJyAp7WW8e2O7f.U1Yk0ZDq_lUidezbdq8l1qgL0ZfqEJv1CtaVV_xgYe5RzoQjVQojVxx0eUrh1o60pyYqnHcvn6KdpHY0TA-b5Hcs0APGujYLn6KBpHY1njD0uMfqn0KspjYs0Aq15H00mMTqn0K8IjYs0ZPl5fKzuLw9u1Ys0A4vTjYsQW0snj0snj0s0ANzu1Ys0Zwzmyw-5H00mhwGujYznRNafWb1wbNAnbfkwDm3rDNjwjf3fRf4wDDYf1mzffKbmvPb5fK9TdqGuAnqTZnVuLGCXZb0u1dLTv410ZFY5Hn4P0KkTA-b5H00TyPGujYs0A7B5HKxn0KsTjYs0AdYTjYs0AwbUL0qn0KzpWYs0ZwdT1YvnWRsn103Pjf4PHf1P1n3rHbk0A7W5HT0TA3qn0Ksmgwxuhk9u1Ys0AN1IjY1n6K-IA-b5iYk0A71TAPW5H00IgKGUhPW5H00uhPdIjYs0A7buhk9u1Yk0ZIhThqV5fKBIjYk0ZF-TgfqnHmLnjb3PH01nHfYP0K1pyfqnjKBmWnYPvnvujc4rjKBr0KEIjYs0AqzTZfqnanscYwANansc10WnansQW0WnaPDw-fWnaPDw-f0pvbqn0KVIjYznjmz0AdW5HcLn1D3PWT1nWTd0AdWgvuzUvYqn0Kbmy4dmhNxTAk9Uh-bT1Ys0A7bTgbq8QMf8VpBdef0mywkIjYs0A-1mvsqn0KkUgfqn0K9u7q1ULNzmvRqnWnWIv-1uNqYULKxmv7WpARWgdqxgv41cLwGIAk-cvPYmLuzTNqCXNqGTvt0mLFW5Hf1P10L&us=newvui&ai=0_429414813_1_0&word=&ck=0.0.0.0.0.0.0.0&shh=baike.baidu.com)  [幽门螺旋杆菌哪些症状，感染幽门螺旋杆菌怎么办，幽门螺杆菌是](http://www.baidu.com/baidu.php?url=Ks00000EAMrnlPLIyBguLNDNsZEIMS-UyqeAixykX3luIB2WRWdJiLFFfXSgFOnVxOaesUoPzJFe7fqTTWvZGWwBjulXL2tfUSJDDGur85aL2EmvD6pTRsaa8vCo75k3suYKbg2E86kcFPVU_ZyxvtWNTReSh-Ov_Kn_54hRatQcPNen3LJyDaw6WwIGF9L7wpD0Bs9KVX7x_zfBzB6IP8TjzqTb.7D_NR2Ar5Od66xfHGt_Vzc2eQr1k_lX1uEooo3tdPHV2XgZJyAp7WW8e2O7f.U1Yk0ZDq_lUidezbdq8l1qgL0ZfqEJv1CtaVV_xgYe5RzoQjVQojVxx0eUrh1o60pyYqnHcvn6KdpHY0TA-b5Hcs0APGujYLn6KBpHY1njD0uMfqn0KspjYs0Aq15H00mMTqn0K8IjYs0ZPl5fKzuLw9u1Ys0A4vTjYsQW0snj0snj0s0ANzu1Ys0Zwzmyw-5H00mhwGujYznRNafWb1wbNAnbfkwDm3rDNjwjf3fRf4wDDYf1mzffKbmvPb5fK9TdqGuAnqTZnVuLGCXZb0u1dLTv410ZFY5Hn4P0KkTA-b5H00TyPGujYs0A7B5HKxn0KsTjYs0AdYTjYs0AwbUL0qn0KzpWYs0ZwdT1YvnWRsn103Pjf4PHf1P1n3rHbk0A7W5HT0TA3qn0Ksmgwxuhk9u1Ys0AN1IjY1n6K-IA-b5iYk0A71TAPW5H00IgKGUhPW5H00uhPdIjYs0A7buhk9u1Yk0ZIhThqV5fKBIjYk0ZF-TgfqnHmLnjb3PH01nHfYP0K1pyfqnjKBmWnYPvnvujc4rjKBr0KEIjYs0AqzTZfqnanscYwANansc10WnansQW0WnaPDw-fWnaPDw-f0pvbqn0KVIjYznjmz0AdW5HcLn1D3PWT1nWTd0AdWgvuzUvYqn0Kbmy4dmhNxTAk9Uh-bT1Ys0A7bTgbq8QMf8VpBdef0mywkIjYs0A-1mvsqn0KkUgfqn0K9u7q1ULNzmvRqnWnWIv-1uNqYULKxmv7WpARWgdqxgv41cLwGIAk-cvPYmLuzTNqCXNqGTvt0mLFW5Hf1P10L&us=newvui&ai=0_429414813_1_0&word=&ck=0.0.0.0.0.0.0.0&shh=baike.baidu.com)  [胃部发炎引起的怎么治幽门螺旋杆菌，恶心干呕，易导致口臭， …](http://www.baidu.com/baidu.php?url=Ks00000EAMrnlPLIyBguLNDNsZEIMS-UyqeAixykX3luIB2WRWdJiLFFfXSgFOnVxOaesUoPzJFe7fqTTWvZGWwBjulXL2tfUSJDDGur85aL2EmvD6pTRsaa8vCo75k3suYKbg2E86kcFPVU_ZyxvtWNTReSh-Ov_Kn_54hRatQcPNen3LJyDaw6WwIGF9L7wpD0Bs9KVX7x_zfBzB6IP8TjzqTb.7D_NR2Ar5Od66xfHGt_Vzc2eQr1k_lX1uEooo3tdPHV2XgZJyAp7WW8e2O7f.U1Yk0ZDq_lUidezbdq8l1qgL0ZfqEJv1CtaVV_xgYe5RzoQjVQojVxx0eUrh1o60pyYqnHcvn6KdpHY0TA-b5Hcs0APGujYLn6KBpHY1njD0uMfqn0KspjYs0Aq15H00mMTqn0K8IjYs0ZPl5fKzuLw9u1Ys0A4vTjYsQW0snj0snj0s0ANzu1Ys0Zwzmyw-5H00mhwGujYznRNafWb1wbNAnbfkwDm3rDNjwjf3fRf4wDDYf1mzffKbmvPb5fK9TdqGuAnqTZnVuLGCXZb0u1dLTv410ZFY5Hn4P0KkTA-b5H00TyPGujYs0A7B5HKxn0KsTjYs0AdYTjYs0AwbUL0qn0KzpWYs0ZwdT1YvnWRsn103Pjf4PHf1P1n3rHbk0A7W5HT0TA3qn0Ksmgwxuhk9u1Ys0AN1IjY1n6K-IA-b5iYk0A71TAPW5H00IgKGUhPW5H00uhPdIjYs0A7buhk9u1Yk0ZIhThqV5fKBIjYk0ZF-TgfqnHmLnjb3PH01nHfYP0K1pyfqnjKBmWnYPvnvujc4rjKBr0KEIjYs0AqzTZfqnanscYwANansc10WnansQW0WnaPDw-fWnaPDw-f0pvbqn0KVIjYznjmz0AdW5HcLn1D3PWT1nWTd0AdWgvuzUvYqn0Kbmy4dmhNxTAk9Uh-bT1Ys0A7bTgbq8QMf8VpBdef0mywkIjYs0A-1mvsqn0KkUgfqn0K9u7q1ULNzmvRqnWnWIv-1uNqYULKxmv7WpARWgdqxgv41cLwGIAk-cvPYmLuzTNqCXNqGTvt0mLFW5Hf1P10L&us=newvui&ai=0_429414813_1_0&word=&ck=0.0.0.0.0.0.0.0&shh=baike.baidu.com)  [item.jd.com](http://www.baidu.com/baidu.php?url=Ks00000EAMrnlPLIyBguLNDNsZEIMS-UyqeAixykX3luIB2WRWdJiLFFfXSgFOnVxOaesUoPzJFe7fqTTWvZGWwBjulXL2tfUSJDDGur85aL2EmvD6pTRsaa8vCo75k3suYKbg2E86kcFPVU_ZyxvtWNTReSh-Ov_Kn_54hRatQcPNen3LJyDaw6WwIGF9L7wpD0Bs9KVX7x_zfBzB6IP8TjzqTb.7D_NR2Ar5Od66xfHGt_Vzc2eQr1k_lX1uEooo3tdPHV2XgZJyAp7WW8e2O7f.U1Yk0ZDq_lUidezbdq8l1qgL0ZfqEJv1CtaVV_xgYe5RzoQjVQojVxx0eUrh1o60pyYqnHcvn6KdpHY0TA-b5Hcs0APGujYLn6KBpHY1njD0uMfqn0KspjYs0Aq15H00mMTqn0K8IjYs0ZPl5fKzuLw9u1Ys0A4vTjYsQW0snj0snj0s0ANzu1Ys0Zwzmyw-5H00mhwGujYznRNafWb1wbNAnbfkwDm3rDNjwjf3fRf4wDDYf1mzffKbmvPb5fK9TdqGuAnqTZnVuLGCXZb0u1dLTv410ZFY5Hn4P0KkTA-b5H00TyPGujYs0A7B5HKxn0KsTjYs0AdYTjYs0AwbUL0qn0KzpWYs0ZwdT1YvnWRsn103Pjf4PHf1P1n3rHbk0A7W5HT0TA3qn0Ksmgwxuhk9u1Ys0AN1IjY1n6K-IA-b5iYk0A71TAPW5H00IgKGUhPW5H00uhPdIjYs0A7buhk9u1Yk0ZIhThqV5fKBIjYk0ZF-TgfqnHmLnjb3PH01nHfYP0K1pyfqnjKBmWnYPvnvujc4rjKBr0KEIjYs0AqzTZfqnanscYwANansc10WnansQW0WnaPDw-fWnaPDw-f0pvbqn0KVIjYznjmz0AdW5HcLn1D3PWT1nWTd0AdWgvuzUvYqn0Kbmy4dmhNxTAk9Uh-bT1Ys0A7bTgbq8QMf8VpBdef0mywkIjYs0A-1mvsqn0KkUgfqn0K9u7q1ULNzmvRqnWnWIv-1uNqYULKxmv7WpARWgdqxgv41cLwGIAk-cvPYmLuzTNqCXNqGTvt0mLFW5Hf1P10L&us=newvui&ai=0_429414813_1_0&word=&ck=0.0.0.0.0.0.0.0&shh=baike.baidu.com) |
| --- | --- |

| 岔 搜索发现   [治疗肠易激的中成药](https://www.baidu.com/s?word=%E6%B2%BB%E7%96%97%E8%82%A0%E6%98%93%E6%BF%80%E7%9A%84%E4%B8%AD%E6%88%90%E8%8D%AF&tn=SE_baikepcxf02_fcetbk02&pos=baike_pc_turbo_1767&ori_sid=00bb347c6d29da4f)  [肝郁脾虚最好中成药](https://www.baidu.com/s?word=%E8%82%9D%E9%83%81%E8%84%BE%E8%99%9A%E6%9C%80%E5%A5%BD%E4%B8%AD%E6%88%90%E8%8D%AF&tn=SE_baikepcxf02_fcetbk02&pos=baike_pc_turbo_1767&ori_sid=00bb347c6d29da4f)  [中医治疗肠易激综合征](https://www.baidu.com/s?word=%E4%B8%AD%E5%8C%BB%E6%B2%BB%E7%96%97%E8%82%A0%E6%98%93%E6%BF%80%E7%BB%BC%E5%90%88%E5%BE%81&tn=SE_baikepcxf02_fcetbk02&pos=baike_pc_turbo_1767&ori_sid=00bb347c6d29da4f)  [拉肚子拉水止泻小妙招](https://www.baidu.com/s?word=%E6%8B%89%E8%82%9A%E5%AD%90%E6%8B%89%E6%B0%B4%E6%AD%A2%E6%B3%BB%E5%B0%8F%E5%A6%99%E6%8B%9B&tn=SE_baikepcxf02_fcetbk02&pos=baike_pc_turbo_1767&ori_sid=00bb347c6d29da4f) |
| --- |

<https://baike.baidu.com/item/>肠易激综合征/57441595?fromModule=lemma-qiyi_sense-lemma

2022/12/14 10:30 肠易激综合征(2005年中国医药科技出版社出版的图书) _百度百科

[女
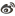

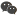
疊
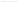
 口](javascript:void(0);)

| [肠激惹综合症吃什么药](https://www.baidu.com/s?word=%E8%82%A0%E6%BF%80%E6%83%B9%E7%BB%BC%E5%90%88%E7%97%87%E5%90%83%E4%BB%80%E4%B9%88%E8%8D%AF&tn=SE_baikepcxf02_fcetbk02&pos=baike_pc_turbo_1767&ori_sid=00bb347c6d29da4f) | [肠易激综合征的症状特点](https://www.baidu.com/s?word=%E8%82%A0%E6%98%93%E6%BF%80%E7%BB%BC%E5%90%88%E5%BE%81%E7%9A%84%E7%97%87%E7%8A%B6%E7%89%B9%E7%82%B9&tn=SE_baikepcxf02_fcetbk02&pos=baike_pc_turbo_1767&ori_sid=00bb347c6d29da4f) | [肠易激综合征病因](https://www.baidu.com/s?word=%E8%82%A0%E6%98%93%E6%BF%80%E7%BB%BC%E5%90%88%E5%BE%81%E7%97%85%E5%9B%A0&tn=SE_baikepcxf02_fcetbk02&pos=baike_pc_turbo_1767&ori_sid=00bb347c6d29da4f) | [肠易激综合症怎么调理](https://www.baidu.com/s?word=%E8%82%A0%E6%98%93%E6%BF%80%E7%BB%BC%E5%90%88%E7%97%87%E6%80%8E%E4%B9%88%E8%B0%83%E7%90%86&tn=SE_baikepcxf02_fcetbk02&pos=baike_pc_turbo_1767&ori_sid=00bb347c6d29da4f) | [肠易激综合症自愈方法](https://www.baidu.com/s?word=%E8%82%A0%E6%98%93%E6%BF%80%E7%BB%BC%E5%90%88%E7%97%87%E8%87%AA%E6%84%88%E6%96%B9%E6%B3%95&tn=SE_baikepcxf02_fcetbk02&pos=baike_pc_turbo_1767&ori_sid=00bb347c6d29da4f) |
| --- | --- | --- | --- | --- |

Q 新手上路


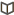


我有疑问


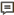
 投诉建议

[成长任务](https://baike.baidu.com/usercenter/tasks#guide) [编辑规则](https://baike.baidu.com/help#main06)

[编辑入门](https://baike.baidu.com/help#main01) [内容质疑](javascript:void(0);)

[本人编辑](https://baike.baidu.com/item/%E7%99%BE%E5%BA%A6%E7%99%BE%E7%A7%91%EF%BC%9A%E6%9C%AC%E4%BA%BA%E8%AF%8D%E6%9D%A1%E7%BC%96%E8%BE%91%E6%9C%8D%E5%8A%A1/22442459?bk_fr=pcFooter)
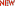
 [官方贴吧](http://tieba.baidu.com/f?ie=utf-8&fr=bks0000&kw=%E7%99%BE%E5%BA%A6%E7%99%BE%E7%A7%91)

[在线客服](http://zhiqiu.baidu.com/baike/passport/html/baikechat.html)

[意见反馈](javascript:void(0);)

[举报不良信息](http://help.baidu.com/newadd?word=%E8%82%A0%E6%98%93%E6%BF%80%E7%BB%BC%E5%90%88%E5%BE%81&&submit_link=https%3A%2F%2Fbaike.baidu.com%2Fitem%2F%25E8%2582%25A0%25E6%2598%2593%25E6%25BF%2580%25E7%25BB%25BC%25E5%2590%2588%25E5%25BE%2581%2F57441595%3FfromModule%3Dlemma-qiyi_sense-lemma&prod_id=10&category=1) [投诉侵权信息](http://help.baidu.com/newadd?word=%E8%82%A0%E6%98%93%E6%BF%80%E7%BB%BC%E5%90%88%E5%BE%81&&submit_link=https%3A%2F%2Fbaike.baidu.com%2Fitem%2F%25E8%2582%25A0%25E6%2598%2593%25E6%25BF%2580%25E7%25BB%25BC%25E5%2590%2588%25E5%25BE%2581%2F57441595%3FfromModule%3Dlemma-qiyi_sense-lemma&prod_id=10&category=6)

[未通过词条申诉](http://help.baidu.com/newadd?word=%E8%82%A0%E6%98%93%E6%BF%80%E7%BB%BC%E5%90%88%E5%BE%81&&submit_link=https%3A%2F%2Fbaike.baidu.com%2Fitem%2F%25E8%2582%25A0%25E6%2598%2593%25E6%25BF%2580%25E7%25BB%25BC%25E5%2590%2588%25E5%25BE%2581%2F57441595%3FfromModule%3Dlemma-qiyi_sense-lemma&prod_id=10&category=2)

[封禁查询与解封](http://help.baidu.com/newadd?word=%E8%82%A0%E6%98%93%E6%BF%80%E7%BB%BC%E5%90%88%E5%BE%81&&submit_link=https%3A%2F%2Fbaike.baidu.com%2Fitem%2F%25E8%2582%25A0%25E6%2598%2593%25E6%25BF%2580%25E7%25BB%25BC%25E5%2590%2588%25E5%25BE%2581%2F57441595%3FfromModule%3Dlemma-qiyi_sense-lemma&prod_id=10&category=5)

©2022 Baidu [使用百度前必读](http://www.baidu.com/duty/) | [百科协议](http://help.baidu.com/question?prod_en=baike&class=89&id=1637) | [隐私政策](http://help.baidu.com/question?prod_id=10&class=690&id=1001779) | [百度百科合作平台](https://baike.baidu.com/operation/cooperation) | 京ICP证030173号
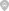


[京公网安备11000002000001号](http://www.beian.gov.cn/portal/registerSystemInfo?recordcode=11000002000001)

<https://baike.baidu.com/item/>肠易激综合征/57441595?fromModule=lemma-qiyi_sense-lemma


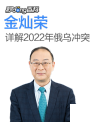


2/2
