# Supplementary material for: Assessment of medical information on irritable bowel syndrome information in Wikipedia and Baidu Encyclopedia: comparative study
Source: PeerJ. 2024 May 24;12:e17264. doi: 10.7717/peerj.17264 (PMC11129691; doi:10.7717/peerj.17264)

网页 新闻 贴吧 知道 网盘 图片 视频 地图 文库 百科 | 百度首页 登录

Baidu百科 功能性腹泻 进入词条 全站搜索 帮助

近期有不法分子冒充百度百科官方人员，以删除词条为由威胁并敲诈相关企业。在此严正声明：百度百科是免费编辑平台，绝不存在收费代编服务，请勿上当受骗！ 详情>>

首页 秒懂百科 特色百科 用户 知识专题 权威合作 下载百科APP | 个

收藏 59 24

功能性腹泻

播报 编辑 上传视频

功能性腹泻（functional diarrhea）是指持续地或反复地出现排稀便或水样便，不伴有腹痛或腹部不适症状的综合征。无细菌、病毒、寄生虫感染的腹泻，一般由胃肠功能失调引起。本病的发生可能与精神因素、肠运动感觉功能异常、自主神经功能紊乱等相关。经各种检查均未能发现引起腹泻的器质性病变。

**外文名**	functional diarrhea	**常见病因**	不明，可能与精神因素、肠运动感
**就诊科室**	消化内科	**常见症状**	不伴有腹痛或腹部不适的少量多次的排便

展开

科普中国
致力于权威的科学传播

本词条认证专家为

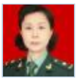

韩英 | 主任医师
北京军区总医院 消化内科
审核

目录

1 病因
2 临床表现
3 检查
4 诊断
5 治疗

词条统计
浏览次数：147939次
编辑次数：8次历史版本
最近更新：卫计委科普项目 （2017-10-03）

突出贡献榜
andnage

基本信息

**外文名**	functional diarrhea	**常见病因**	不明，可能与精神因素、肠运动感觉功能异常、自主神经功能紊乱等有关
**就诊科室**	消化内科	**常见症状**	不伴有腹痛或腹部不适的少量多次的排便

病因

播报 编辑

有腹泻症状但经各种检查均未发现引起腹泻的器质性病变，称为功能性腹泻。其病因复杂，包括原因未明的肠易激综合征和原因已明确的继发性小肠乳糖酶缺乏症等。虽病因还不清楚，但研究证明因精神紧张可引起结肠动力的改变，出现腹泻等症状。

临床表现

播报 编辑

功能性腹泻患者常表现为不伴有腹痛或腹部不适的少量多次的排便，但需要与“假性腹泻”相鉴别，“假性腹泻”表现为排便次数多、伴排便急迫感，但每次都排成形便。

检查

播报 编辑

对疑为腹泻患者应该进行血常规、血沉(ESR)及c反应蛋白(CRP)检查。血清铁蛋白与叶酸的测定可帮助与小肠吸收不良相鉴别。若高度怀疑IBD或小肠吸收不良者，则应进行胃肠道的影像学或胃肠镜检查。对严重水样泻患者需排除内分泌疾病引起的腹泻，如肠血管活性肽（VIP）瘤和5-HIAA等导致的腹泻。

诊断

播报 编辑

排除器质性病变，符合以下条件时可以考虑诊断功能性腹泻：①至少75%的排便为不伴有腹痛的稀粪或水样粪；②诊断前症状出现至少6个月，近3个月符合以上诊断标准。

需要注意的是：腹泻仅是一种非特异性的肠道症状，引起腹泻的病因很多。功能性腹泻须与肠道器质性疾病，如肠道感染性疾病（慢性细菌性痢疾、肠结核、寄生虫感染性腹泻等）、IBD、放射性肠炎、结肠肿瘤、小肠吸收不良及VIP瘤等引起的腹泻相鉴别。消化道内镜、血液生化等相关检查有助于鉴别诊断。

秒懂百科
金灿荣
详解2022年俄乌冲突

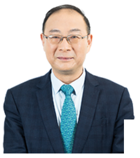

治疗

播报 编辑

对功能性腹泻患者应实施个体化的治疗方案，对由于应激事件引起的腹泻患者，需对其进行详细的病情解释，有助于缓解患者的焦虑情绪。

1.饮食治疗

详细询问患者的饮食习惯。面食、乳制品、柑橘类水果、蛋类、洋葱、咖啡因及酒精等均可能引发功能性腹泻患者症状。因此，限制这些食物将有助于缓解患者的腹泻症状。

2.药物治疗

若改变生活方式无效，可采用药物治疗。常用的抗腹泻药物有洛哌丁胺和苯乙哌啶，此类药物通过与阿片受体结合，阻止胆碱能神经末梢释放乙酰胆碱，而发挥抗腹泻作用。洛哌丁胺还有非阿片样作用达到止泻目的。

学术论文

内容来自 Baidu学术

李琳，李岩．肠道菌群失调与功能性腹泻．《胃肠病学和肝病学杂志》，2014

李红梅，梁浩，唐湖泉，李祥．匹维溴铵对小鼠功能性腹泻及便秘的治疗作用．《世界华人消化杂志》，1998

李琳，李岩．整肠生联合常乐康治疗伴肠道菌群失调功能性腹泻的疗效观察．《CNKI》，2014

许继宗，李玉华，张喆，张波．体感五行音乐疗法联合参苓白术散治疗功能性腹泻疗效观察．《CNKI》，2012

马秀丽，李正军．参苓白术散联合理中汤治疗慢性功能性腹泻35例．《陕西中医》，2011

查看全部 >

猜你喜欢

腹泻贴多少钱一盒零售，潮流新品，好货热卖，更...

腹泻贴多少钱一盒零售，购物上淘宝，优选材质，用的舒心!在线下单，省时省力。你要的好货尽在淘宝网，安心享受网购乐趣!

simba.taobao.com

搜索发现

功能性胃病 | 腹泻的症状 | 肠易激综合征会自愈吗 | 拉肚子拉水止泻小妙招 | 什么是腹泻
治拉肚子的偏方 | 腹泻的治疗方法 | 腹泻出血怎么回事 | 功能性腹泻怎么治 | 腹胀便秘怎么治疗

新手上路
成长任务 编辑入门
编辑规则 本人编辑 NEW

我有疑问
内容质疑 在线客服
官方贴吧 意见反馈

投诉建议
举报不良信息 未通过词条申诉
投诉侵权信息 封禁查询与解封

©2022 Baidu 使用百度前必读 | 百科协议 | 隐私政策 | 百度百科合作平台 | 京ICP证030173号
京公网安备11000002000001号

秒懂百科
金灿荣
详解2022年俄乌冲突

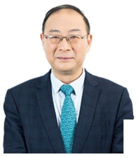

Supplement: Data S1 [file peerj-12-17264-s001.zip › σÄƒσoïμò░μì«/Baidu/Baidu-Chinese/10-σèƒΦâ╜μÇoΦà╣μ│╗_τÖ╛σ║aτÖ╛τoæ.pdf]
