# Supplementary material for: Assessment of medical information on irritable bowel syndrome information in Wikipedia and Baidu Encyclopedia: comparative study
Source: PeerJ. 2024 May 24;12:e17264. doi: 10.7717/peerj.17264 (PMC11129691; doi:10.7717/peerj.17264)
Supplement: Data S1 [file peerj-12-17264-s001.zip › σÄƒσoïμò░μì«/Baidu/Baidu-Chinese/6-σñoΦéáμ┐ÇΦ║üτùç_τÖ╛σ║aτÖ╛τoæ.docx]

| 2022/12/14 10:41  [网页](https://www.baidu.com/) | [新闻](http://news.baidu.com/) | 大肠激躁症_百度百科  [贴吧](https://tieba.baidu.com/) [知道](https://zhidao.baidu.com/) [网盘](https://pan.baidu.com/?from=1027327l) [图片](http://image.baidu.com/) | [视频](http://v.baidu.com/) | [地图](http://map.baidu.com/) | [文库](https://wenku.baidu.com/) | 百科 | 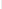 [百度首页](http://www.baidu.com/) [登录](javascript:;) |
| --- | --- | --- | --- | --- | --- | --- | --- |

| [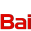岔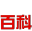](https://baike.baidu.com/) | \| 大肠激躁症 \| 进入词条 \| \| --- \| --- \| | \| 全站搜索 \| \| --- \| | [帮助](https://baike.baidu.com/help) |
| --- | --- | --- | --- | --- | --- | --- |
| 近期有不法分子冒充百度百科官方人员，以删除词条为由威胁并敲诈相关企业。在此严正声明：百度百科是免费编辑平台，绝不存在收费代编服务，请勿上当受骗！ [详情>>](https://baike.baidu.com/common/declaration) | | | |
| [首页](https://baike.baidu.com/) 秒懂百科 特色百科 用户 知识专题 权威合作 [口下载百科APP](https://baike.baidu.com/wapui/subpage/baikeappdownload?sfrom=pc_lemmapage_navigation) 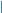 [2 个](https://baike.baidu.com/usercenter) | | | |

| 大肠激躁症 | \| [小播报](javascript:;) \| \| --- \| | \| [c编辑](javascript:;) \| \| --- \| | \| [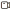上传视频](javascript:;) \| \| --- \| | [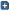](javascript:;)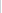 . 收藏 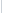[山 34](javascript:void(0);)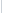 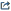 1 | \| 词条统计  浏览次数： 41855次  编辑次数： 4次[历史版本](https://baike.baidu.com/historylist/%E5%A4%A7%E8%82%A0%E6%BF%80%E8%BA%81%E7%97%87/3109976)  最近更新： [yghm](https://baike.baidu.com/usercenter/userpage?uk=CwPuzWXvk8Fzpi5IqCzNNA&from=lemma) ( 2012-06-01)  突出贡献榜  [crocosmia](https://baike.baidu.com/usercenter/userpage?uk=3R4HDrhSyqNd4nVPAZPnWQ&from=lemma) [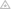](https://baike.baidu.com/item/%E7%A7%91%E9%BE%84%E5%8B%8B%E7%AB%A0/59405227) \| \| --- \| |
| --- | --- | --- | --- | --- | --- | --- | --- | --- | --- |
| 大肠激躁症， Constipation -Irritable Bowel Syndrome,即C-IBS。大肠激躁症简言之是由于肠道之功能异常所表现出来的症候 群。其症状可包含腹痛、排便习惯改变、胀气、腹胀、解便不完全的感觉，或粪便中出现黏液。几乎每位成人都曾经历过其中一 或数种状况。然而，当这些症状一起出现，持续存在，且具有相当严重之生活困扰时，它们就倾向于被认定为一种功能障碍疾 病。 | | | | |  |
| \| 目录 \| 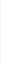▪ [(2)饮食因素](#_bookmark1)  1 [简介](#_bookmark4)  2 [病因及致病机转](#_bookmark5)  ▪ ( 1)肠道蠕动功能障 碍  3 [诊断](#_bookmark6)  ▪ [(3)神经异常](#_bookmark2)  ▪ [(4)感觉异常](#_bookmark3)  ▪ ( 5)肠道与大脑之讯  息交流异常 \| \| --- \| --- \| | | | | |  |
|  |  |  |  |  | [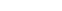](https://cpro.baidu.com/cpro/ui/uijs.php?en=mywWUA71T1YsFh7sT7qGujYsFhPC5H0huAbqrauGTdq9TZ0qnauJp1d-uWIhn1nYnWR3mvD4uW0hp1Y-fWb-wbR-fWT-wW0-fWf-wWn-wjD-fHT-f1b-wRD-f1T-wRc-fYn-wWR-fbn-wbRhUZNopHYkFhdWTAYqrHDsnHmhTHY1P104PjDYn7qWTZchThcqnauzT1YkFMP-UAk-T-qGujYkFMPGujdhnAD4rAnzPHnYuWPhP1K-FMPYpyfqrauY5gwsmvkGmvV-ujPxpAnhIAfqnHb4P1m1nzuYUHYzPW63njndrjDhIAd15HDvP104rjRvnjbhIZRqIHD4rHTvn1nhIHdCIZwsTzR1fiRzwBRzwhF9pyV-FHF7mh7GuZR-nbNWUvYhIWYzFhbqPvfzrym1nhR&besl=6&c=news&cf=1&cvrq=2024363&eid_list=201577_204854_207574_209357&expid=201577_202257_202564_204854_207574_209394&fr=20&fv=0&haacp=870&img_typ=0&itm=0&lu_idc=gzhxy&lukid=1&lus=f0a98c2534f3f70e&lust=63993789&luwtr=7841139350953723636&mscf=0&n=10&nttp=1&p=baidu&pbs=220093&sce=7&sr=72&ssp2=1&tpl=baiduCustITagLinkUnitRankCol&tsf=dtp:1&tu_type=0&u=%2Fitem%2F%25E5%25A4%25A7%25E8%2582%25A0%25E6%25BF%2580%25E8%25BA%2581%25E7%2597%2587%2F3109976%3FfromModule%3Dsearch%2Dresult%5Flemma&uicf=lurecv&urlid=0&eot=1)[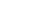](https://cpro.baidu.com/cpro/ui/uijs.php?en=mywWUA71T1YsFh7sT7qGujYsFhPC5H0huAbqrauGTdq9TZ0qnauJp1d-uWIhn1nYnWR3mvD4uW0hp1Y-fYf-wW6-f1c-wHT-fWb-fHf-fWn-fYn-fYD-fHmhUZNopHYknBuVmLKV5HD1nHD3nauk5HnLnjbYnHfsgvPsTBuzmWYsFMF15HDhTvN_UANzgv-b5HDhTv-b5ymsmHb3m1cdn1whnvmLnARhTLwGujY3FMfqIZKWUA-WpvNbndqCmzuYujYkrHbLPWn1FMwV5Hcvrj6sn1R3niuYUgnqnHmLnjb3PHmsriuYIHddnHb4P1m1nzud5y9YIZK1FHPKFHFAFHFAmh7GpvR-nbNBmy-bIiRzwyPEUiuv5HchpHYknjf3mHfvn6&besl=6&c=news&cf=1&cvrq=3661677&eid_list=201577_204854_207574_209357&expid=201577_202257_202564_204854_207574_209394&fr=20&fv=0&haacp=1439&img_typ=0&itm=0&lu_idc=gzhxy&lukid=12&lus=f0a98c2534f3f70e&lust=63993789&luwtr=750531414731215287&mscf=0&n=10&nttp=1&p=baidu&pbs=220093&sce=7&sr=72&ssp2=1&tpl=baiduCustITagLinkUnitRankCol&tsf=dtp:1&tu_type=0&u=%2Fitem%2F%25E5%25A4%25A7%25E8%2582%25A0%25E6%25BF%2580%25E8%25BA%2581%25E7%2597%2587%2F3109976%3FfromModule%3Dsearch%2Dresult%5Flemma&uicf=lurecv&urlid=0&eot=1)[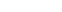](https://cpro.baidu.com/cpro/ui/uijs.php?en=mywWUA71T1YsFh7sT7qGujYsFhPC5H0huAbqrauGTdq9TZ0qnauJp1d-uWIhn1nYnWR3mvD4uW0hp1Y-fWR-wHT-f1b-fYn-f1m-fbf-fYn-fH6-wjf-wWR-f1n-fWf-wjT-wWmhUZNopHYzFhdWTAYqrj0LP16hTHY1P104PjDYn7qWTZchThcqnauzT1YkFMP-UAk-T-qGujYkFMPGujdhnAD4rAnzPHnYuWPhP1K-FMPYpyfqrauY5gwsmvkGmvV-ujPxpAnhIAfqnHb4P1m1nzuYUHYzPW63njndrjDhIAd15HDvP104rjRvnjbhIZRqIHD4rHTvn1nhIHdCIZwsTzR1fiRzwBRzwhF9pyV-FHF7mh7GuZR-nbNWUvYhIWYzFhbqm1PbnjNhrj0&besl=6&c=news&cf=1&cvrq=3247861&eid_list=201577_204854_207574_209357&expid=201577_202257_202564_204854_207574_209394&fr=20&fv=0&haacp=3601&img_typ=0&itm=0&lu_idc=gzhxy&lukid=2&lus=f0a98c2534f3f70e&lust=63993789&luwtr=7777852140969087504&mscf=0&n=10&nttp=1&p=baidu&pbs=220093&sce=7&sr=72&ssp2=1&tpl=baiduCustITagLinkUnitRankCol&tsf=dtp:1&tu_type=0&u=%2Fitem%2F%25E5%25A4%25A7%25E8%2582%25A0%25E6%25BF%2580%25E8%25BA%2581%25E7%2597%2587%2F3109976%3FfromModule%3Dsearch%2Dresult%5Flemma&uicf=lurecv&urlid=0&eot=1)[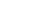](https://cpro.baidu.com/cpro/ui/uijs.php?en=mywWUA71T1YsFh7sT7qGujYsFhPC5H0huAbqrauGTdq9TZ0qnauJp1d-uWIhn1nYnWR3mvD4uW0hp1Y-wjT-wjf-fbn-fbD-fWf-fWf-fbf-fH6-fW6-wWm-fYf-wW6-wjR-fbRhUZNopHYknzuVmLKV5HDzn1cYnzuk5HnLnjbYnHfsgvPsTBuzmWYsFMF15HDhTvN_UANzgv-b5HDhTv-b5ymsmHb3m1cdn1whnvmLnARhTLwGujY3FMfqIZKWUA-WpvNbndqCmzuYujYkrHbLPWn1FMwV5Hcvrj6sn1R3niuYUgnqnHmLnjb3PHmsriuYIHddnHb4P1m1nzud5y9YIZK1FHPKFHFAFHFAmh7GpvR-nbNBmy-bIiRzwyPEUiuv5HchpHYdujfLnjKBP0&besl=6&c=news&cf=1&cvrq=2235876&eid_list=201577_204854_207574_209357&expid=201577_202257_202564_204854_207574_209394&fr=20&fv=0&haacp=1001&img_typ=0&itm=0&lu_idc=gzhxy&lukid=13&lus=f0a98c2534f3f70e&lust=63993789&luwtr=6556233252601032990&mscf=0&n=10&nttp=1&p=baidu&pbs=220093&sce=7&sr=72&ssp2=1&tpl=baiduCustITagLinkUnitRankCol&tsf=dtp:1&tu_type=0&u=%2Fitem%2F%25E5%25A4%25A7%25E8%2582%25A0%25E6%25BF%2580%25E8%25BA%2581%25E7%2597%2587%2F3109976%3FfromModule%3Dsearch%2Dresult%5Flemma&uicf=lurecv&urlid=0&eot=1)[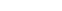](https://cpro.baidu.com/cpro/ui/uijs.php?en=mywWUA71T1YsFh7sT7qGujYsFhPC5H0huAbqrauGTdq9TZ0qnauJp1d-uWIhn1nYnWR3mvD4uW0hp1Y-wj0-wHb-f1f-wHc-fbc-wWR-fWD-wjc-f1m-fbf-fYn-fH6hUZNopHYYFhdWTAYqP16vPHfhTHY1P104PjDYn7qWTZchThcqnauzT1YkFMP-UAk-T-qGujYkFMPGujdhnAD4rAnzPHnYuWPhP1K-FMPYpyfqrauY5gwsmvkGmvV-ujPxpAnhIAfqnHb4P1m1nzuYUHYzPW63njndrjDhIAd15HDvP104rjRvnjbhIZRqIHD4rHTvn1nhIHdCIZwsTzR1fiRzwBRzwhF9pyV-FHF7mh7GuZR-nbNWUvYhIWYzFhbqujwhPhf1uH6&besl=6&c=news&cf=1&cvrq=3011782&eid_list=201577_204854_207574_209357&expid=201577_202257_202564_204854_207574_209394&fr=20&fv=0&haacp=194&img_typ=0&itm=0&lu_idc=gzhxy&lukid=4&lus=f0a98c2534f3f70e&lust=63993789&luwtr=2314272910775226794&mscf=0&n=10&nttp=1&p=baidu&pbs=220093&sce=7&sr=72&ssp2=1&tpl=baiduCustITagLinkUnitRankCol&tsf=dtp:1&tu_type=0&u=%2Fitem%2F%25E5%25A4%25A7%25E8%2582%25A0%25E6%25BF%2580%25E8%25BA%2581%25E7%2597%2587%2F3109976%3FfromModule%3Dsearch%2Dresult%5Flemma&uicf=lurecv&urlid=0&eot=1)[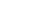](https://cpro.baidu.com/cpro/ui/uijs.php?en=mywWUA71T1YsFh7sT7qGujYsFhPC5H0huAbqrauGTdq9TZ0qnauJp1d-uWIhn1nYnWR3mvD4uW0hp1Y-fYR-wDR-f16-fYc-fbc-wbD-fWT-fWf-wjm-f1mhUZNopHYkPiuVmLKV5H6sP1bvFMDqn1TsrHfkPjKxmLKzFMFB5H0hTMnqniu1uyk_ugFxpyfqniu1pyfquWK9rH9WnWR1PAm1uWTsuiu1IA-b5H6hIjdYTAP_pyPouyf1gv9WFMwb5HD4rHTvn1nhIAYqnWm3rj01PH6kFMwVT1YkPWTsrH6dPW04FMwd5gRkrHbLPWn1FMRqpZwYTZn-nYD-nbm-nbuBmy-ouiRzwyF9pywdFHF7mvqVFMmqnBuG5ymkmWTYPhub&besl=6&c=news&cf=1&cvrq=1415934&eid_list=201577_204854_207574_209357&expid=201577_202257_202564_204854_207574_209394&fr=20&fv=0&haacp=904&img_typ=0&itm=0&lu_idc=gzhxy&lukid=15&lus=f0a98c2534f3f70e&lust=63993789&luwtr=685006414410405008&mscf=0&n=10&nttp=1&p=baidu&pbs=220093&sce=7&sr=72&ssp2=1&tpl=baiduCustITagLinkUnitRankCol&tsf=dtp:1&tu_type=0&u=%2Fitem%2F%25E5%25A4%25A7%25E8%2582%25A0%25E6%25BF%2580%25E8%25BA%2581%25E7%2597%2587%2F3109976%3FfromModule%3Dsearch%2Dresult%5Flemma&uicf=lurecv&urlid=0&eot=1)[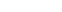](https://cpro.baidu.com/cpro/ui/uijs.php?en=mywWUA71T1YsFh7sT7qGujYsFhPC5H0huAbqrauGTdq9TZ0qnauJp1d-uWIhn1nYnWR3mvD4uW0hp1dWTvIEFRcdFRRLFRnYFRfYFRndFRRYFRfvFRn1FhkdpvbqPiuVmLKV5HT3nW0kFMDqn1TsrHfkPjKxmLKzFMFB5H0hTMnqniu1uyk_ugFxpyfqniu1pyfquWK9rH9WnWR1PAm1uWTsuiu1IA-b5H6hIjdYTAP_pyPouyf1gv9WFMwb5HD4rHTvn1nhIAYqnWm3rj01PH6kFMwVT1YkPWTsrH6dPW04FMwd5gRkrHbLPWn1FMRqpZwYTZn-nYD-nbm-nbuBmy-ouiRzwyF9pywdFHF7mvqVFMmqnBuG5HR4nW03mhmv&besl=6&c=news&cf=1&cvrq=3472465&eid_list=201577_204854_207574_209357&expid=201577_202257_202564_204854_207574_209394&fr=20&fv=0&haacp=611&img_typ=0&itm=0&lu_idc=gzhxy&lukid=5&lus=f0a98c2534f3f70e&lust=63993789&luwtr=2357911270216075011&mscf=0&n=10&nttp=1&p=baidu&pbs=220093&sce=7&sr=72&ssp2=1&tpl=baiduCustITagLinkUnitRankCol&tsf=dtp:1&tu_type=0&u=%2Fitem%2F%25E5%25A4%25A7%25E8%2582%25A0%25E6%25BF%2580%25E8%25BA%2581%25E7%2597%2587%2F3109976%3FfromModule%3Dsearch%2Dresult%5Flemma&uicf=lurecv&urlid=0&eot=1)[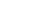](https://cpro.baidu.com/cpro/ui/uijs.php?en=mywWUA71T1YsFh7sT7qGujYsFhPC5H0huAbqrauGTdq9TZ0qnauJp1d-uWIhn1nYnWR3mvD4uW0hp1Y-fbn-wDc-fW6-wWD-fWD-wHn-wjc-fYc-fWR-f1f-fYm-wHn-fW6-wDc-fWT-wbR-fYR-wWD-f1m-wWThUZNopHYkPBuVmLKV5HT4nW6sFMDqn1TsrHfkPjKxmLKzFMFB5H0hTMnqniu1uyk_ugFxpyfqniu1pyfquWK9rH9WnWR1PAm1uWTsuiu1IA-b5H6hIjdYTAP_pyPouyf1gv9WFMwb5HD4rHTvn1nhIAYqnWm3rj01PH6kFMwVT1YkPWTsrH6dPW04FMwd5gRkrHbLPWn1FMRqpZwYTZn-nYD-nbm-nbuBmy-ouiRzwyF9pywdFHF7mvqVFMmqnBuG5ymYPynYuHRv&besl=6&c=news&cf=1&cvrq=2363941&eid_list=201577_204854_207574_209357&expid=201577_202257_202564_204854_207574_209394&fr=20&fv=0&haacp=1750&img_typ=0&itm=0&lu_idc=gzhxy&lukid=16&lus=f0a98c2534f3f70e&lust=63993789&luwtr=18274635138362951998&mscf=0&n=10&nttp=1&p=baidu&pbs=220093&sce=7&sr=72&ssp2=1&tpl=baiduCustITagLinkUnitRankCol&tsf=dtp:1&tu_type=0&u=%2Fitem%2F%25E5%25A4%25A7%25E8%2582%25A0%25E6%25BF%2580%25E8%25BA%2581%25E7%2597%2587%2F3109976%3FfromModule%3Dsearch%2Dresult%5Flemma&uicf=lurecv&urlid=0&eot=1)[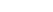](https://cpro.baidu.com/cpro/ui/uijs.php?en=mywWUA71T1YsFh7sT7qGujYsFhPC5H0huAbqrauGTdq9TZ0qnauJp1d-uWIhn1nYnWR3mvD4uW0hp1Y-wjD-f1T-f1c-wRf-wjD-fWT-fYf-fbn-fYD-wHbhUZNopHYvFhdWTAYqrj0sPHRhTHY1P104PjDYn7qWTZchThcqnauzT1YkFMP-UAk-T-qGujYkFMPGujdhnAD4rAnzPHnYuWPhP1K-FMPYpyfqrauY5gwsmvkGmvV-ujPxpAnhIAfqnHb4P1m1nzuYUHYzPW63njndrjDhIAd15HDvP104rjRvnjbhIZRqIHD4rHTvn1nhIHdCIZwsTzR1fiRzwBRzwhF9pyV-FHF7mh7GuZR-nbNWUvYhIWYzFhbqP1bsujDdnWc&besl=6&c=news&cf=1&cvrq=3335536&eid_list=201577_204854_207574_209357&expid=201577_202257_202564_204854_207574_209394&fr=20&fv=0&haacp=219&img_typ=0&itm=0&lu_idc=gzhxy&lukid=6&lus=f0a98c2534f3f70e&lust=63993789&luwtr=718744168689952326&mscf=0&n=10&nttp=1&p=baidu&pbs=220093&sce=7&sr=72&ssp2=1&tpl=baiduCustITagLinkUnitRankCol&tsf=dtp:1&tu_type=0&u=%2Fitem%2F%25E5%25A4%25A7%25E8%2582%25A0%25E6%25BF%2580%25E8%25BA%2581%25E7%2597%2587%2F3109976%3FfromModule%3Dsearch%2Dresult%5Flemma&uicf=lurecv&urlid=0&eot=1)[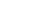](https://cpro.baidu.com/cpro/ui/uijs.php?en=mywWUA71T1YsFh7sT7qGujYsFhPC5H0huAbqrauGTdq9TZ0qnauJp1d-uWIhn1nYnWR3mvD4uW0hp1d1mvb-f1c-wDc-fYR-f1f-fYf-fWm-fW6-wHRhUZNopHYkPzuVmLKV5HDzrjc3niuk5HnLnjbYnHfsgvPsTBuzmWYsFMF15HDhTvN_UANzgv-b5HDhTv-b5ymsmHb3m1cdn1whnvmLnARhTLwGujY3FMfqIZKWUA-WpvNbndqCmzuYujYkrHbLPWn1FMwV5Hcvrj6sn1R3niuYUgnqnHmLnjb3PHmsriuYIHddnHb4P1m1nzud5y9YIZK1FHPKFHFAFHFAmh7GpvR-nbNBmy-bIiRzwyPEUiuv5HchpHYzmymdmWDduf&besl=6&c=news&cf=1&cvrq=3196467&eid_list=201577_204854_207574_209357&expid=201577_202257_202564_204854_207574_209394&fr=20&fv=0&haacp=1032&img_typ=0&itm=0&lu_idc=gzhxy&lukid=17&lus=f0a98c2534f3f70e&lust=63993789&luwtr=2480674890665447709&mscf=0&n=10&nttp=1&p=baidu&pbs=220093&sce=7&sr=72&ssp2=1&tpl=baiduCustITagLinkUnitRankCol&tsf=dtp:1&tu_type=0&u=%2Fitem%2F%25E5%25A4%25A7%25E8%2582%25A0%25E6%25BF%2580%25E8%25BA%2581%25E7%2597%2587%2F3109976%3FfromModule%3Dsearch%2Dresult%5Flemma&uicf=lurecv&urlid=0&eot=1)[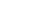](https://cpro.baidu.com/cpro/ui/uijs.php?en=mywWUA71T1YsFh7sT7qGujYsFhPC5H0huAbqrauGTdq9TZ0qnauJp1d-uWIhn1nYnWR3mvD4uW0hp1Y-fYf-fbn-fYD-wHb-f1R-wbD-fWT-fHc-fYf-wW6hUZNopHYLFhdWTAYqP1bYnWRhTHY1P104PjDYn7qWTZchThcqnauzT1YkFMP-UAk-T-qGujYkFMPGujdhnAD4rAnzPHnYuWPhP1K-FMPYpyfqrauY5gwsmvkGmvV-ujPxpAnhIAfqnHb4P1m1nzuYUHYzPW63njndrjDhIAd15HDvP104rjRvnjbhIZRqIHD4rHTvn1nhIHdCIZwsTzR1fiRzwBRzwhF9pyV-FHF7mh7GuZR-nbNWUvYhIWYzFhbquAu9nhnzrHb&besl=6&c=news&cf=1&cvrq=4468518&eid_list=201577_204854_207574_209357&expid=201577_202257_202564_204854_207574_209394&fr=20&fv=0&haacp=317&img_typ=0&itm=0&lu_idc=gzhxy&lukid=7&lus=f0a98c2534f3f70e&lust=63993789&luwtr=639862747281857404&mscf=0&n=10&nttp=1&p=baidu&pbs=220093&sce=7&sr=72&ssp2=1&tpl=baiduCustITagLinkUnitRankCol&tsf=dtp:1&tu_type=0&u=%2Fitem%2F%25E5%25A4%25A7%25E8%2582%25A0%25E6%25BF%2580%25E8%25BA%2581%25E7%2597%2587%2F3109976%3FfromModule%3Dsearch%2Dresult%5Flemma&uicf=lurecv&urlid=0&eot=1)[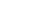](https://cpro.baidu.com/cpro/ui/uijs.php?en=mywWUA71T1YsFh7sT7qGujYsFhPC5H0huAbqrauGTdq9TZ0qnauJp1d-uWIhn1nYnWR3mvD4uW0hp1dvTBNjwBNAfBNaPzNjnaNDniNDwaNjniNaPzu_IyVG5HD3FhdWTAYqnHnLrjnYFMDqn1TsrHfkPjKxmLKzFMFB5H0hTMnqniu1uyk_ugFxpyfqniu1pyfquWK9rH9WnWR1PAm1uWTsuiu1IA-b5H6hIjdYTAP_pyPouyf1gv9WFMwb5HD4rHTvn1nhIAYqnWm3rj01PH6kFMwVT1YkPWTsrH6dPW04FMwd5gRkrHbLPWn1FMRqpZwYTZn-nYD-nbm-nbuBmy-ouiRzwyF9pywdFHF7mvqVFMmqnBuG5yRdPADdn1n3&besl=6&c=news&cf=1&cvrq=4705749&eid_list=201577_204854_207574_209357&expid=201577_202257_202564_204854_207574_209394&fr=20&fv=0&haacp=1024&img_typ=0&itm=0&lu_idc=gzhxy&lukid=18&lus=f0a98c2534f3f70e&lust=63993789&luwtr=754962655298315806&mscf=0&n=10&nttp=1&p=baidu&pbs=220093&sce=7&sr=72&ssp2=1&tpl=baiduCustITagLinkUnitRankCol&tsf=dtp:1&tu_type=0&u=%2Fitem%2F%25E5%25A4%25A7%25E8%2582%25A0%25E6%25BF%2580%25E8%25BA%2581%25E7%2597%2587%2F3109976%3FfromModule%3Dsearch%2Dresult%5Flemma&uicf=lurecv&urlid=0&eot=1)[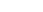](https://cpro.baidu.com/cpro/ui/uijs.php?en=mywWUA71T1YsFh7sT7qGujYsFhPC5H0huAbqrauGTdq9TZ0qnauJp1d-uWIhn1nYnWR3mvD4uW0hp1Y-fWb-fbD-f1c-wWc-wjn-wWc-f1n-wbchUZNopHY3FhdWTAYqrHfznjThTHY1P104PjDYn7qWTZchThcqnauzT1YkFMP-UAk-T-qGujYkFMPGujdhnAD4rAnzPHnYuWPhP1K-FMPYpyfqrauY5gwsmvkGmvV-ujPxpAnhIAfqnHb4P1m1nzuYUHYzPW63njndrjDhIAd15HDvP104rjRvnjbhIZRqIHD4rHTvn1nhIHdCIZwsTzR1fiRzwBRzwhF9pyV-FHF7mh7GuZR-nbNWUvYhIWYzFhbqnHKbmyu9nWb&besl=6&c=news&cf=1&cvrq=1941531&eid_list=201577_204854_207574_209357&expid=201577_202257_202564_204854_207574_209394&fr=20&fv=0&haacp=1286&img_typ=0&itm=0&lu_idc=gzhxy&lukid=8&lus=f0a98c2534f3f70e&lust=63993789&luwtr=17492749210616066771&mscf=0&n=10&nttp=1&p=baidu&pbs=220093&sce=7&sr=72&ssp2=1&tpl=baiduCustITagLinkUnitRankCol&tsf=dtp:1&tu_type=0&u=%2Fitem%2F%25E5%25A4%25A7%25E8%2582%25A0%25E6%25BF%2580%25E8%25BA%2581%25E7%2597%2587%2F3109976%3FfromModule%3Dsearch%2Dresult%5Flemma&uicf=lurecv&urlid=0&eot=1)[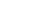](https://cpro.baidu.com/cpro/ui/uijs.php?en=mywWUA71T1YsFh7sT7qGujYsFhPC5H0huAbqrauGTdq9TZ0qnauJp1d-uWIhn1nYnWR3mvD4uW0hp1dWTvIEFRPDFRm3FRfdFRF7FRFAFR7KFRPAFRRYFhkdpvbqnHbhUyPsUHYknWDsrHDhTHY1P104PjDYn7qWTZchThcqnauzT1YkFMP-UAk-T-qGujYkFMPGujdhnAD4rAnzPHnYuWPhP1K-FMPYpyfqrauY5gwsmvkGmvV-ujPxpAnhIAfqnHb4P1m1nzuYUHYzPW63njndrjDhIAd15HDvP104rjRvnjbhIZRqIHD4rHTvn1nhIHdCIZwsTzR1fiRzwBRzwhF9pyV-FHF7mh7GuZR-nbNWUvYhIWYzFhbqnWmdPhmzmWR&besl=6&c=news&cf=1&cvrq=4489244&eid_list=201577_204854_207574_209357&expid=201577_202257_202564_204854_207574_209394&fr=20&fv=0&haacp=624&img_typ=0&itm=0&lu_idc=gzhxy&lukid=19&lus=f0a98c2534f3f70e&lust=63993789&luwtr=2267704484931445128&mscf=0&n=10&nttp=1&p=baidu&pbs=220093&sce=7&sr=72&ssp2=1&tpl=baiduCustITagLinkUnitRankCol&tsf=dtp:1&tu_type=0&u=%2Fitem%2F%25E5%25A4%25A7%25E8%2582%25A0%25E6%25BF%2580%25E8%25BA%2581%25E7%2597%2587%2F3109976%3FfromModule%3Dsearch%2Dresult%5Flemma&uicf=lurecv&urlid=0&eot=1)[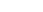](https://cpro.baidu.com/cpro/ui/uijs.php?en=mywWUA71T1YsFh7sT7qGujYsFhPC5H0huAbqrauGTdq9TZ0qnauJp1d-uWIhn1nYnWR3mvD4uW0hp1Y-wjn-fYR-fYm-fWT-fbD-wj0-wjT-wjnhUZNopHY4FhdWTAYqP1c4njRhTHY1P104PjDYn7qWTZchThcqnauzT1YkFMP-UAk-T-qGujYkFMPGujdhnAD4rAnzPHnYuWPhP1K-FMPYpyfqrauY5gwsmvkGmvV-ujPxpAnhIAfqnHb4P1m1nzuYUHYzPW63njndrjDhIAd15HDvP104rjRvnjbhIZRqIHD4rHTvn1nhIHdCIZwsTzR1fiRzwBRzwhF9pyV-FHF7mh7GuZR-nbNWUvYhIWYzFhbqn1Nbuyn4PhD&besl=6&c=news&cf=1&cvrq=3228426&eid_list=201577_204854_207574_209357&expid=201577_202257_202564_204854_207574_209394&fr=20&fv=0&haacp=271&img_typ=0&itm=0&lu_idc=gzhxy&lukid=9&lus=f0a98c2534f3f70e&lust=63993789&luwtr=13245032438307475642&mscf=0&n=10&nttp=1&p=baidu&pbs=220093&sce=7&sr=72&ssp2=1&tpl=baiduCustITagLinkUnitRankCol&tsf=dtp:1&tu_type=0&u=%2Fitem%2F%25E5%25A4%25A7%25E8%2582%25A0%25E6%25BF%2580%25E8%25BA%2581%25E7%2597%2587%2F3109976%3FfromModule%3Dsearch%2Dresult%5Flemma&uicf=lurecv&urlid=0&eot=1)[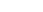](https://cpro.baidu.com/cpro/ui/uijs.php?en=mywWUA71T1YsFh7sT7qGujYsFhPC5H0huAbqrauGTdq9TZ0qnauJp1d-uWIhn1nYnWR3mvD4uW0hp1Y-fWb-wbD-fbn-fYD-f1m-wDD-fbc-wWRhUZNopHYznauVmLKV5HDsrjb1Pzuk5HnLnjbYnHfsgvPsTBuzmWYsFMF15HDhTvN_UANzgv-b5HDhTv-b5ymsmHb3m1cdn1whnvmLnARhTLwGujY3FMfqIZKWUA-WpvNbndqCmzuYujYkrHbLPWn1FMwV5Hcvrj6sn1R3niuYUgnqnHmLnjb3PHmsriuYIHddnHb4P1m1nzud5y9YIZK1FHPKFHFAFHFAmh7GpvR-nbNBmy-bIiRzwyPEUiuv5HchpHdWm1bsuj9-Ps&besl=6&c=news&cf=1&cvrq=1621391&eid_list=201577_204854_207574_209357&expid=201577_202257_202564_204854_207574_209394&fr=20&fv=0&haacp=2073&img_typ=0&itm=0&lu_idc=gzhxy&lukid=20&lus=f0a98c2534f3f70e&lust=63993789&luwtr=14608826967881669318&mscf=0&n=10&nttp=1&p=baidu&pbs=220093&sce=7&sr=72&ssp2=1&tpl=baiduCustITagLinkUnitRankCol&tsf=dtp:1&tu_type=0&u=%2Fitem%2F%25E5%25A4%25A7%25E8%2582%25A0%25E6%25BF%2580%25E8%25BA%2581%25E7%2597%2587%2F3109976%3FfromModule%3Dsearch%2Dresult%5Flemma&uicf=lurecv&urlid=0&eot=1)[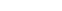](https://cpro.baidu.com/cpro/ui/uijs.php?en=mywWUA71T1YsFh7sT7qGujYsFhPC5H0huAbqrauGTdq9TZ0qnauJp1d-uWIhn1nYnWR3mvD4uW0hp1Y-fWn-wWm-fW0-wHm-f1b-wHT-wjT-wjf-fWT-wjD-fWn-wWm-fYD-wHbhUZNopHYknauVmLKV5HDknjR1PBuk5HnLnjbYnHfsgvPsTBuzmWYsFMF15HDhTvN_UANzgv-b5HDhTv-b5ymsmHb3m1cdn1whnvmLnARhTLwGujY3FMfqIZKWUA-WpvNbndqCmzuYujYkrHbLPWn1FMwV5Hcvrj6sn1R3niuYUgnqnHmLnjb3PHmsriuYIHddnHb4P1m1nzud5y9YIZK1FHPKFHFAFHFAmh7GpvR-nbNBmy-bIiRzwyPEUiuv5HchpHYkPAc1mWwWnf&besl=6&c=news&cf=1&cvrq=7189029&eid_list=201577_204854_207574_209357&expid=201577_202257_202564_204854_207574_209394&fr=20&fv=0&haacp=618&img_typ=0&itm=0&lu_idc=gzhxy&lukid=10&lus=f0a98c2534f3f70e&lust=63993789&luwtr=7476068094432150857&mscf=0&n=10&nttp=1&p=baidu&pbs=220093&sce=7&sr=72&ssp2=1&tpl=baiduCustITagLinkUnitRankCol&tsf=dtp:1&tu_type=0&u=%2Fitem%2F%25E5%25A4%25A7%25E8%2582%25A0%25E6%25BF%2580%25E8%25BA%2581%25E7%2597%2587%2F3109976%3FfromModule%3Dsearch%2Dresult%5Flemma&uicf=lurecv&urlid=0&eot=1)[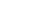](https://cpro.baidu.com/cpro/ui/uijs.php?en=mywWUA71T1YsFh7sT7qGujYsFhPC5H0huAbqrauGTdq9TZ0qnauJp1d-uWIhn1nYnWR3mvD4uW0hp1Y-wjf-wWR-f1n-fWf-fWf-fWf-fbf-fH6-wj0-fHD-fWn-fYn-wj0-wWchUZNopHYzniuVmLKV5H61PWDkFMDqn1TsrHfkPjKxmLKzFMFB5H0hTMnqniu1uyk_ugFxpyfqniu1pyfquWK9rH9WnWR1PAm1uWTsuiu1IA-b5H6hIjdYTAP_pyPouyf1gv9WFMwb5HD4rHTvn1nhIAYqnWm3rj01PH6kFMwVT1YkPWTsrH6dPW04FMwd5gRkrHbLPWn1FMRqpZwYTZn-nYD-nbm-nbuBmy-ouiRzwyF9pywdFHF7mvqVFMmqnBuG5yuWuAw-uycd&besl=6&c=news&cf=1&cvrq=2189719&eid_list=201577_204854_207574_209357&expid=201577_202257_202564_204854_207574_209394&fr=20&fv=0&haacp=734&img_typ=0&itm=0&lu_idc=gzhxy&lukid=21&lus=f0a98c2534f3f70e&lust=63993789&luwtr=7550494890368818072&mscf=0&n=10&nttp=1&p=baidu&pbs=220093&sce=7&sr=72&ssp2=1&tpl=baiduCustITagLinkUnitRankCol&tsf=dtp:1&tu_type=0&u=%2Fitem%2F%25E5%25A4%25A7%25E8%2582%25A0%25E6%25BF%2580%25E8%25BA%2581%25E7%2597%2587%2F3109976%3FfromModule%3Dsearch%2Dresult%5Flemma&uicf=lurecv&urlid=0&eot=1)[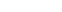](https://cpro.baidu.com/cpro/ui/uijs.php?en=mywWUA71T1YsFh7sT7qGujYsFhPC5H0huAbqrauGTdq9TZ0qnauJp1d-uWIhn1nYnWR3mvD4uW0hp1Y-wjR-fbf-fWm-wjP_UvIEFRn4FRR3FRFjFRnvFhkdpvbqnHDhUyPsUHY4PHc1Pauk5HnLnjbYnHfsgvPsTBuzmWYsFMF15HDhTvN_UANzgv-b5HDhTv-b5ymsmHb3m1cdn1whnvmLnARhTLwGujY3FMfqIZKWUA-WpvNbndqCmzuYujYkrHbLPWn1FMwV5Hcvrj6sn1R3niuYUgnqnHmLnjb3PHmsriuYIHddnHb4P1m1nzud5y9YIZK1FHPKFHFAFHFAmh7GpvR-nbNBmy-bIiRzwyPEUiuv5HchpHd9uyn3uHwBuf&besl=6&c=news&cf=1&cvrq=1756705&eid_list=201577_204854_207574_209357&expid=201577_202257_202564_204854_207574_209394&fr=20&fv=0&haacp=707&img_typ=0&itm=0&lu_idc=gzhxy&lukid=11&lus=f0a98c2534f3f70e&lust=63993789&luwtr=1863292750894598650&mscf=0&n=10&nttp=1&p=baidu&pbs=220093&sce=7&sr=72&ssp2=1&tpl=baiduCustITagLinkUnitRankCol&tsf=dtp:1&tu_type=0&u=%2Fitem%2F%25E5%25A4%25A7%25E8%2582%25A0%25E6%25BF%2580%25E8%25BA%2581%25E7%2597%2587%2F3109976%3FfromModule%3Dsearch%2Dresult%5Flemma&uicf=lurecv&urlid=0&eot=1)[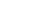](https://cpro.baidu.com/cpro/ui/uijs.php?en=mywWUA71T1YsFh7sT7qGujYsFhPC5H0huAbqrauGTdq9TZ0qnauJp1d-uWIhn1nYnWR3mvD4uW0hp1Y-fWb-fHb-wjn-fHm-f1D-fWf-fWb-wDn-f10-wRf-f1m-fbf-fYn-fH6hUZNopHYznBuVmLKV5H6dnjTsFMDqn1TsrHfkPjKxmLKzFMFB5H0hTMnqniu1uyk_ugFxpyfqniu1pyfquWK9rH9WnWR1PAm1uWTsuiu1IA-b5H6hIjdYTAP_pyPouyf1gv9WFMwb5HD4rHTvn1nhIAYqnWm3rj01PH6kFMwVT1YkPWTsrH6dPW04FMwd5gRkrHbLPWn1FMRqpZwYTZn-nYD-nbm-nbuBmy-ouiRzwyF9pywdFHF7mvqVFMmqnBuG5yPbmyDzrjmz&besl=6&c=news&cf=1&cvrq=2271496&eid_list=201577_204854_207574_209357&expid=201577_202257_202564_204854_207574_209394&fr=20&fv=0&haacp=952&img_typ=0&itm=0&lu_idc=gzhxy&lukid=22&lus=f0a98c2534f3f70e&lust=63993789&luwtr=6130823900782380766&mscf=0&n=10&nttp=1&p=baidu&pbs=220093&sce=7&sr=72&ssp2=1&tpl=baiduCustITagLinkUnitRankCol&tsf=dtp:1&tu_type=0&u=%2Fitem%2F%25E5%25A4%25A7%25E8%2582%25A0%25E6%25BF%2580%25E8%25BA%2581%25E7%2597%2587%2F3109976%3FfromModule%3Dsearch%2Dresult%5Flemma&uicf=lurecv&urlid=0&eot=1)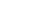   \| 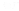**1** 哈佛大学申请 **12** 网络工程师  **2** 电商平台怎么 **13** 自己创建个  [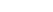](https://cpro.baidu.com/cpro/ui/uijs.php?en=mywWUA71T1YsFh7sT7qGujYsFhPC5H0huAbqrauGTdq9TZ0qnauJp1d-uWIhn1nYnWR3mvD4uW0hp1Y1PzNDnzNjwiNjwBNaPzNjPBNawaNjfzNKrau_IyVG5HnhUyPsUHY4nWnsnauk5HnLnjbYnHfsgvPsTBuzmWYsFMF15HDhTvN_UANzgv-b5HDhTv-b5ymsmHb3m1cdn1whnvmLnARhTLwGujY3FMfqIZKWUA-WpvNbndqCmzuYujYkrHbLPWn1FMwV5Hcvrj6sn1R3niuYUgnqnHmLnjb3PHmsriuYIHddnHb4P1m1nzud5y9YIZK1FHPKFHFAFHFAmh7GpvR-nbNBmy-bIiRzwyPEUiuv5HchpHdbrH6knA7hPf&besl=6&c=news&cf=1&cvrq=3623810&eid_list=201577_204854_207574_209357&expid=201577_202257_202564_204854_207574_209394&fr=20&fv=0&haacp=188&img_typ=0&itm=0&lu_idc=gzhxy&lukid=3&lus=f0a98c2534f3f70e&lust=63993789&luwtr=676781759077005289&mscf=0&n=10&nttp=1&p=baidu&pbs=220093&sce=7&sr=72&ssp2=1&tpl=baiduCustITagLinkUnitRankCol&tsf=dtp:1&tu_type=0&u=%2Fitem%2F%25E5%25A4%25A7%25E8%2582%25A0%25E6%25BF%2580%25E8%25BA%2581%25E7%2597%2587%2F3109976%3FfromModule%3Dsearch%2Dresult%5Flemma&uicf=lurecv&urlid=0&eot=1)[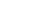](https://cpro.baidu.com/cpro/ui/uijs.php?en=mywWUA71T1YsFh7sT7qGujYsFhPC5H0huAbqrauGTdq9TZ0qnauJp1d-uWIhn1nYnWR3mvD4uW0hp1Y-fYf-wW6-f1c-wHT-fW0-fWc-f16-fRc-f1R-wH0-wjD-fWRhUZNopHYkPauVmLKV5HDznjmLniuk5HnLnjbYnHfsgvPsTBuzmWYsFMF15HDhTvN_UANzgv-b5HDhTv-b5ymsmHb3m1cdn1whnvmLnARhTLwGujY3FMfqIZKWUA-WpvNbndqCmzuYujYkrHbLPWn1FMwV5Hcvrj6sn1R3niuYUgnqnHmLnjb3PHmsriuYIHddnHb4P1m1nzud5y9YIZK1FHPKFHFAFHFAmh7GpvR-nbNBmy-bIiRzwyPEUiuv5HchpHdBnywBPjf4rf&besl=6&c=news&cf=1&cvrq=3292150&eid_list=201577_204854_207574_209357&expid=201577_202257_202564_204854_207574_209394&fr=20&fv=0&haacp=1162&img_typ=0&itm=0&lu_idc=gzhxy&lukid=14&lus=f0a98c2534f3f70e&lust=63993789&luwtr=2249689388344567100&mscf=0&n=10&nttp=1&p=baidu&pbs=220093&sce=7&sr=72&ssp2=1&tpl=baiduCustITagLinkUnitRankCol&tsf=dtp:1&tu_type=0&u=%2Fitem%2F%25E5%25A4%25A7%25E8%2582%25A0%25E6%25BF%2580%25E8%25BA%2581%25E7%2597%2587%2F3109976%3FfromModule%3Dsearch%2Dresult%5Flemma&uicf=lurecv&urlid=0&eot=1)**3** 37游戏平台 **14** 网络安全培  **4** 虚拟货币平台 **15** 无人机反制  **5** csgo电脑配置 **16** 价格便宜的  **6** 亚马逊图书 **17** sci论文投稿  **7** 图书批发网 **18** vr消防演练  **8** 购买域名 **19** csgo网站开  **9** 游戏盒子 **20** 国际期货  **10** 出版社自费出 **21** 怎么创建小  **11** 战队logo设计 **22** 供应链管理 \| \| --- \| |
| [小 播报c编辑](javascript:;)  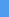 简介 | | | | |  |
| 肠道激躁症不会致命，但很少被治愈，且终其一生均可出现，或持续进展。通常患者之解便习惯无一定之准则－有时便秘， 有时腹泻，有时短期内同时发生这两种状况。大肠激躁症与器质病变之异，在于其症状无法由具体的病灶来解释，甚至也无法明 确地以病理生理学阐明。虽然患者之肠道有明显的功能异常，目前之科技尚无法精确衡量，也缺乏适当的诊断工具及相关的检 查。因此，临床上多只能由患者描述的症状，来诊断大肠激躁症之存在。 | | | | |  |
|  |  |  |  |  | [女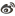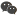疊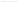 口](javascript:void(0);) |
| 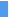 病因及致病机转  [小 播报c编辑](javascript:;) | | | | |  |
| 以下为可能为诱发大肠激躁症之原因，但目前尚无定论：  (1)肠道蠕动功能障碍  此学说主张部分患者之肠道蠕动缓慢，部分较快，另有部分患者为快慢交替出现，型态多变。目前这派学说仍有待以肠道蠕 动之研究结果证实。  (2)饮食因素  若以特定药物在正常自愿者造成便秘时，他们亦会产生大肠激躁症之症状。而纤维素之减少摄取，也可有类似效果。然而， 并无研究证实大肠激躁症患者摄取之纤维素量与正常人有异。因此，纤维素之缺乏，似非明确的致病原因。虽然许多患者认为大 肠激躁症之发作与特定食物有关，但这不易以客观方法证实。事实上，摄食本身即可刺激肠道蠕动。总结来说，摄食，特别是一 次吃下大量食物，可在大肠激躁症之患者，以一种非特异性之方式刺激症状产生，但目前尚未证实与何种特定食物有关。  (3)神经异常  另一种学说，是症状源于肠道神经控制系统之异常，而非肠道本身。目前许多研究着重于肠间神经丛，也被称为 “ 肠道之脑 “。针对肠间神经丛及其与中枢神经系统关联之研究，已开启了一个新的领域：胃肠神经学。学者认为，了解肠间神经丛之生理特 性及化学组成，有助于解开大肠激躁症之谜团。肠间神经丛相当复杂，也含有中枢神经系统内所含，大部分的神经传导物质。举 例来说，睡眠期肠道的电气活动频率与大脑相同，且患者多有不正常的快速动眼期睡眠。然而，目前尚未证实肠间神经丛为大肠 激躁症之主要病因所在。  (4)感觉异常  1980年代以降，感觉异常之理论亦被提出，并引起广泛的注意。许多研究发现，当直肠或乙状结肠中置入气球，开始打气 时，大肠激躁症患者会比正常人在较小的打气量或压力下即感到不适或疼痛。类似的状况，也出现在小肠，甚至食道。然而，大 肠激躁症患者皮肤感觉疼痛之阈值与正常人无异，甚至更高，因此有 ”肠道过度敏感” 之理论。这可解释为何大肠激躁症患者对正 常之刺激过度敏感，而产生不正常之反射及肠道运动。但导致肠道过度敏感的原因，又是什么？  以下数种状况，被认为可能造成肠道过度敏感：  －肠道黏膜受损(如发炎、感染或暴露于有毒化学物质中)  －肠道肌肉过度伸张 | | | | |  |
|  |  |  |  |  | 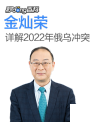 |

<https://baike.baidu.com/item/>大肠激躁症/3109976?fromModule=search-result_lemma 1/3


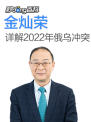
2022/12/14 10:41

[女
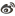

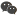
疊口](javascript:void(0);)

大肠激躁症_百度百科

－脊髓神经之交界处有渗漏现象

－某种诱发讯号由较高层次之神经中枢经脊髓神经下传

(5)肠道与大脑之讯息交流异常

此理论除了融入前一理论外，亦兼顾心理及生理因素，亦能严重影响症候之形成。

[小 播报c编辑](javascript:;)


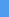
 诊断

临床医师面临之大肠激躁症之诊断挑战，是如何分析患者之症状，并认清大肠激躁症之次型。 Manning Criteria 指出了六种 于大肠激躁症比结构性腹部疾病更常出现之症状：

I. 解便后腹痛减轻

II. 开始腹痛时，大便变软

III. 开始腹痛时，解便次数较频繁

IV. 腹胀

V. 解出黏液

VI. 感觉大便解不干净

1999年，一群对大肠激躁症有高度研究兴趣之胃肠专家，以Manning Criteria及Rome Criteria为依据，制定了Rome II Criteria，此即成为目前最广为接受及应用之诊断标准：

※ 在过去12个月中，至少有12周(不须连续)发生腹痛或不适，且至少包含以下两种特征：

I. 腹部不适于解便后缓解

II. 解便频率改变

III. 粪便形态改变

※ 在有症状之时日或场合中，至少有四分之一时间发生以下一种或一种以上之症状，而且可用来区分不同分型之大肠激躁 症。这些症状并非诊断所必需，但出现愈多，医师对大肠激躁症之诊断就更具说服力：

I. 异常之排便频率(一天多于三次或一周少于三次)

II. 异常之粪便型态(硬块或稀软便/水便)

III. 异常之排便型态(用力、急迫或感到大便解不干净)

IV. 排出黏液

V. 胀气或感到腹胀

附注：患者必须无造成这些症状之结构性或代谢疾病症状出现之十二周不需有连续性

值得注意的是，若患者出现以下现象，则为器质性疾病之警讯，须一步安排适当之检查：

I. 血便或由肛门排血

II. 体重减轻

III. 持续腹泻

IV. 最近才出现，且持续未缓解之腹胀

V. 贫血

VI. 发烧

举例来说，肠炎，特别是糜烂性肠炎，可符合 Manning Criteria 中之五项标准。然而，其血便形态、发烧、体重减轻、贫 血，可作为鉴别。

| 岔 搜索发现   [激躁性大肠要吃药吗](https://www.baidu.com/s?word=%E6%BF%80%E8%BA%81%E6%80%A7%E5%A4%A7%E8%82%A0%E8%A6%81%E5%90%83%E8%8D%AF%E5%90%97&tn=SE_baikepcxf02_fcetbk02&pos=baike_pc_turbo_1767&ori_sid=00bb350c8f9b552a)   [大肠激躁症如何治疗](https://www.baidu.com/s?word=%E5%A4%A7%E8%82%A0%E6%BF%80%E8%BA%81%E7%97%87%E5%A6%82%E4%BD%95%E6%B2%BB%E7%96%97&tn=SE_baikepcxf02_fcetbk02&pos=baike_pc_turbo_1767&ori_sid=00bb350c8f9b552a)  [激躁性大肠症怎么治愈](https://www.baidu.com/s?word=%E6%BF%80%E8%BA%81%E6%80%A7%E5%A4%A7%E8%82%A0%E7%97%87%E6%80%8E%E4%B9%88%E6%B2%BB%E6%84%88&tn=SE_baikepcxf02_fcetbk02&pos=baike_pc_turbo_1767&ori_sid=00bb350c8f9b552a)   [大肠激躁症能喝酸奶吗](https://www.baidu.com/s?word=%E5%A4%A7%E8%82%A0%E6%BF%80%E8%BA%81%E7%97%87%E8%83%BD%E5%96%9D%E9%85%B8%E5%A5%B6%E5%90%97&tn=SE_baikepcxf02_fcetbk02&pos=baike_pc_turbo_1767&ori_sid=00bb350c8f9b552a)  [大肠激躁症会背痛](https://www.baidu.com/s?word=%E5%A4%A7%E8%82%A0%E6%BF%80%E8%BA%81%E7%97%87%E4%BC%9A%E8%83%8C%E7%97%9B&tn=SE_baikepcxf02_fcetbk02&pos=baike_pc_turbo_1767&ori_sid=00bb350c8f9b552a)   [大肠激躁症吃什么药](https://www.baidu.com/s?word=%E5%A4%A7%E8%82%A0%E6%BF%80%E8%BA%81%E7%97%87%E5%90%83%E4%BB%80%E4%B9%88%E8%8D%AF&tn=SE_baikepcxf02_fcetbk02&pos=baike_pc_turbo_1767&ori_sid=00bb350c8f9b552a)  [部队文职是什么工作](https://www.baidu.com/s?word=%E9%83%A8%E9%98%9F%E6%96%87%E8%81%8C%E6%98%AF%E4%BB%80%E4%B9%88%E5%B7%A5%E4%BD%9C&tn=SE_baikepcxf02_fcetbk02&pos=baike_pc_turbo_1767&ori_sid=00bb350c8f9b552a)   [大肠激躁症可以自愈](https://www.baidu.com/s?word=%E5%A4%A7%E8%82%A0%E6%BF%80%E8%BA%81%E7%97%87%E5%8F%AF%E4%BB%A5%E8%87%AA%E6%84%88&tn=SE_baikepcxf02_fcetbk02&pos=baike_pc_turbo_1767&ori_sid=00bb350c8f9b552a)  [免费兼职](https://www.baidu.com/s?word=%E5%85%8D%E8%B4%B9%E5%85%BC%E8%81%8C&tn=SE_baikepcxf02_fcetbk02&pos=baike_pc_turbo_1767&ori_sid=00bb350c8f9b552a)   [激躁性大肠症放屁多](https://www.baidu.com/s?word=%E6%BF%80%E8%BA%81%E6%80%A7%E5%A4%A7%E8%82%A0%E7%97%87%E6%94%BE%E5%B1%81%E5%A4%9A&tn=SE_baikepcxf02_fcetbk02&pos=baike_pc_turbo_1767&ori_sid=00bb350c8f9b552a) |
| --- |

<https://baike.baidu.com/item/>大肠激躁症/3109976?fromModule=search-result_lemma 2/3

[
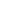
](javascript:;)2022/12/14 10:41 大肠激躁症_百度百科

[女
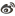

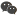
疊
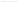
 口](javascript:void(0);)

Q 新手上路


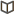


我有疑问


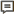
 投诉建议

[成长任务](https://baike.baidu.com/usercenter/tasks#guide) [编辑规则](https://baike.baidu.com/help#main06)

[编辑入门](https://baike.baidu.com/help#main01) [内容质疑](javascript:void(0);)

[本人编辑](https://baike.baidu.com/item/%E7%99%BE%E5%BA%A6%E7%99%BE%E7%A7%91%EF%BC%9A%E6%9C%AC%E4%BA%BA%E8%AF%8D%E6%9D%A1%E7%BC%96%E8%BE%91%E6%9C%8D%E5%8A%A1/22442459?bk_fr=pcFooter)
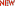
 [官方贴吧](http://tieba.baidu.com/f?ie=utf-8&fr=bks0000&kw=%E7%99%BE%E5%BA%A6%E7%99%BE%E7%A7%91)

[在线客服](http://zhiqiu.baidu.com/baike/passport/html/baikechat.html)

[意见反馈](javascript:void(0);)

[举报不良信息](http://help.baidu.com/newadd?word=%E5%A4%A7%E8%82%A0%E6%BF%80%E8%BA%81%E7%97%87&&submit_link=https%3A%2F%2Fbaike.baidu.com%2Fitem%2F%25E5%25A4%25A7%25E8%2582%25A0%25E6%25BF%2580%25E8%25BA%2581%25E7%2597%2587%2F3109976%3FfromModule%3Dsearch-result_lemma&prod_id=10&category=1) [投诉侵权信息](http://help.baidu.com/newadd?word=%E5%A4%A7%E8%82%A0%E6%BF%80%E8%BA%81%E7%97%87&&submit_link=https%3A%2F%2Fbaike.baidu.com%2Fitem%2F%25E5%25A4%25A7%25E8%2582%25A0%25E6%25BF%2580%25E8%25BA%2581%25E7%2597%2587%2F3109976%3FfromModule%3Dsearch-result_lemma&prod_id=10&category=6)

[未通过词条申诉](http://help.baidu.com/newadd?word=%E5%A4%A7%E8%82%A0%E6%BF%80%E8%BA%81%E7%97%87&&submit_link=https%3A%2F%2Fbaike.baidu.com%2Fitem%2F%25E5%25A4%25A7%25E8%2582%25A0%25E6%25BF%2580%25E8%25BA%2581%25E7%2597%2587%2F3109976%3FfromModule%3Dsearch-result_lemma&prod_id=10&category=2)

[封禁查询与解封](http://help.baidu.com/newadd?word=%E5%A4%A7%E8%82%A0%E6%BF%80%E8%BA%81%E7%97%87&&submit_link=https%3A%2F%2Fbaike.baidu.com%2Fitem%2F%25E5%25A4%25A7%25E8%2582%25A0%25E6%25BF%2580%25E8%25BA%2581%25E7%2597%2587%2F3109976%3FfromModule%3Dsearch-result_lemma&prod_id=10&category=5)

©2022 Baidu [使用百度前必读](http://www.baidu.com/duty/) | [百科协议](http://help.baidu.com/question?prod_en=baike&class=89&id=1637) | [隐私政策](http://help.baidu.com/question?prod_id=10&class=690&id=1001779) | [百度百科合作平台](https://baike.baidu.com/operation/cooperation) | 京ICP证030173号
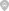


[京公网安备11000002000001号](http://www.beian.gov.cn/portal/registerSystemInfo?recordcode=11000002000001)

<https://baike.baidu.com/item/>大肠激躁症/3109976?fromModule=search-result_lemma


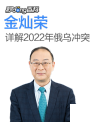


3/3
