# Supplementary material for: Assessment of medical information on irritable bowel syndrome information in Wikipedia and Baidu Encyclopedia: comparative study
Source: PeerJ. 2024 May 24;12:e17264. doi: 10.7717/peerj.17264 (PMC11129691; doi:10.7717/peerj.17264)
Supplement: Data S1 [file peerj-12-17264-s001.zip › σÄƒσoïμò░μì«/Baidu/Baidu-Chinese/9-μàóμÇoσèƒΦâ╜μÇoΣ╛┐τoÿ_τÖ╛σ║aτÖ╛τoæ.docx]

| 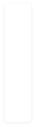[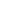](javascript:void(0);)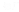[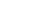](https://cpro.baidu.com/cpro/ui/uijs.php?en=mywWUA71T1YsFh7sT7qGujYsFhPC5H0huAbqrauGTdq9TZ0qnauJp1YzmHcYmWT1mvRvuW01nWPWFh_qFRn3FRDdFRcdFRnzFRc4FRuKFRnkFRmYFRfkFRDLFhkdpvbqnHnhUyPsUHY4nH0kPiuk5HnLnjbYnHfsgvPsTBuzmWYsFMF15HDhTvN_UANzgv-b5HDhTv-b5HPWn1FhnARvnvPBP1cYnhDhTLwGujY3FMfqIZKWUA-WpvNbndqCmzuYujYkrHbLPWn1FMwV5Hcvrj6sn1R3niuYUgnqnHmLnjb3PW04PBuYIHddnHb4P1m1nzud5y9YIZK1FHPKFHFAFHFAmh7GpvR-nbNBmy-bIiRzwyPEUiuv5HchpHYvPyFWn1N9P0&besl=6&c=news&cf=1&cvrq=2626231&eid_list=201577_203434_207574_208118_209357&expid=201577_202257_202564_203434_205809_207574_208118_209394&fr=20&fv=0&haacp=184&img_typ=0&itm=0&lu_idc=gzhxy&lukid=13&lus=3c32f0e63cb7242a&lust=63993970&luwtr=828544633882669046&mscf=0&n=10&nttp=1&p=baidu&pbs=220093&sce=7&sr=72&ssp2=1&tpl=baiduCustITagLinkUnitRankCol&tsf=dtp:1&tu_type=0&u=%2Fitem%2F%25E6%2585%25A2%25E6%2580%25A7%25E5%258A%259F%25E8%2583%25BD%25E6%2580%25A7%25E4%25BE%25BF%25E7%25A7%2598%3FfromModule%3Dlemma%5Fsearch%2Dbox&uicf=lurecv&urlid=0&eot=1)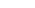2022/12/14 10:48 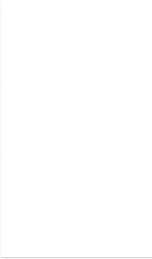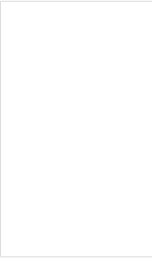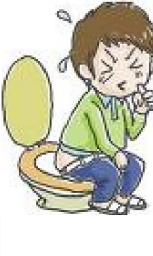 [慢性功能性便秘的概述图 张)](https://baike.baidu.com/pic/%E6%85%A2%E6%80%A7%E5%8A%9F%E8%83%BD%E6%80%A7%E4%BE%BF%E7%A7%98/8952323/1/6c224f4a20a4462333086e729b22720e0df3d7e4?fr=lemma&fromModule=lemma_top-image&ct=single)   \| 词条统计  浏览次数： 244558次  编辑次数： 18次[历史版本](https://baike.baidu.com/historylist/%E6%85%A2%E6%80%A7%E5%8A%9F%E8%83%BD%E6%80%A7%E4%BE%BF%E7%A7%98/8952323)  最近更新： [涵宇Q](https://baike.baidu.com/usercenter/userpage?uk=sAKxvCB7Bi22BNyXo0TMfQ&from=lemma) ( 2021-12-04)  突出贡献榜  [bianmi8](https://baike.baidu.com/usercenter/userpage?uk=fn9sdkW-KBqeK3DkiYmx7w&from=lemma) [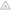](https://baike.baidu.com/item/%E7%A7%91%E9%BE%84%E5%8B%8B%E7%AB%A0/59405227) \| \| --- \|   [女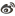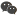](javascript:void(0);)  [疊](javascript:void(0);)  [dna亲子鉴定](https://cpro.baidu.com/cpro/ui/uijs.php?en=mywWUA71T1YsFh7sT7qGujYsFhPC5H0huAbqrauGTdq9TZ0qnauJp1YzmHcYmWT1mvRvuW01nWPWFh_quA49FRnLFRfLFRfLFRf1FRFjFRm3FRcvFRD3FRcvFRRsFRn4FRf4FRnLFR77FhkdpvbqnHchUyPsUHYknHD4njbhTHY1P104PjDYn7qWTZchThcqnauzT1YkFMP-UAk-T-qGujYkFMPGujY1m1nzuWK-PWPWmWTzPjF9FMPYpyfqrauY5gwsmvkGmvV-ujPxpAnhIAfqnHb4P1m1nzuYUHYzPW63njndrjDhIAd15HDvP104rjmsrHmhIZRqIHD4rHTvn1nhIHdCIZwsTzR1fiRzwBRzwhF9pyV-FHF7mh7GuZR-nbNWUvYhIWYzFhbquH9-mHb3PHT&besl=6&c=news&cf=1&cvrq=1893597&eid_list=201577_203434_207574_208118_209357&expid=201577_202257_202564_203434_205809_207574_208118_209394&fr=20&fv=0&haacp=348&img_typ=0&itm=0&lu_idc=gzhxy&lukid=12&lus=3c32f0e63cb7242a&lust=63993970&luwtr=84518820883097642&mscf=0&n=10&nttp=1&p=baidu&pbs=220093&sce=7&sr=72&ssp2=1&tpl=baiduCustITagLinkUnitRankCol&tsf=dtp:1&tu_type=0&u=%2Fitem%2F%25E6%2585%25A2%25E6%2580%25A7%25E5%258A%259F%25E8%2583%25BD%25E6%2580%25A7%25E4%25BE%25BF%25E7%25A7%2598%3FfromModule%3Dlemma%5Fsearch%2Dbox&uicf=lurecv&urlid=0&eot=1)  去德国口留学  [螺杆式家用](https://cpro.baidu.com/cpro/ui/uijs.php?en=mywWUA71T1YsFh7sT7qGujYsFhPC5H0huAbqrauGTdq9TZ0qnauJp1YzmHcYmWT1mvRvuW01nWPWFh_qFRnzFRwDFRc3FRPaFRPKFRFDFRFjFRfzFRf1FRn1FRcdFRRLFRPjFRwDFhkdpvbqnHfhUyPsUHY4PWTvriuk5HnLnjbYnHfsgvPsTBuzmWYsFMF15HDhTvN_UANzgv-b5HDhTv-b5HPWn1FhnARvnvPBP1cYnhDhTLwGujY3FMfqIZKWUA-WpvNbndqCmzuYujYkrHbLPWn1FMwV5Hcvrj6sn1R3niuYUgnqnHmLnjb3PW04PBuYIHddnHb4P1m1nzud5y9YIZK1FHPKFHFAFHFAmh7GpvR-nbNBmy-bIiRzwyPEUiuv5HchpHY4uWIhuW6zrf&besl=6&c=news&cf=1&cvrq=1618597&eid_list=201577_203434_207574_208118_209357&expid=201577_202257_202564_203434_205809_207574_208118_209394&fr=20&fv=0&haacp=547&img_typ=0&itm=0&lu_idc=gzhxy&lukid=14&lus=3c32f0e63cb7242a&lust=63993970&luwtr=7205834330722693679&mscf=0&n=10&nttp=1&p=baidu&pbs=220093&sce=7&sr=72&ssp2=1&tpl=baiduCustITagLinkUnitRankCol&tsf=dtp:1&tu_type=0&u=%2Fitem%2F%25E6%2585%25A2%25E6%2580%25A7%25E5%258A%259F%25E8%2583%25BD%25E6%2580%25A7%25E4%25BE%25BF%25E7%25A7%2598%3FfromModule%3Dlemma%5Fsearch%2Dbox&uicf=lurecv&urlid=0&eot=1) [vr消防演练](https://cpro.baidu.com/cpro/ui/uijs.php?en=mywWUA71T1YsFh7sT7qGujYsFhPC5H0huAbqrauGTdq9TZ0qnauJp1YzmHcYmWT1mvRvuW01nWPWFh_qIMc-fYm-wbc-fWT-f10-wjD-wDf-f1D-fWThUZNopHYkPiuVmLKV5HD1rjczrauk5HnLnjbYnHfsgvPsTBuzmWYsFMF15HDhTvN_UANzgv-b5HDhTv-b5HPWn1FhnARvnvPBP1cYnhDhTLwGujY3FMfqIZKWUA-WpvNbndqCmzuYujYkrHbLPWn1FMwV5Hcvrj6sn1R3niuYUgnqnHmLnjb3PW04PBuYIHddnHb4P1m1nzud5y9YIZK1FHPKFHFAFHFAmh7GpvR-nbNBmy-bIiRzwyPEUiuv5HchpHYknv79PARzP6&besl=6&c=news&cf=1&cvrq=4705752&eid_list=201577_203434_207574_208118_209357&expid=201577_202257_202564_203434_205809_207574_208118_209394&fr=20&fv=0&haacp=1024&img_typ=0&itm=0&lu_idc=gzhxy&lukid=15&lus=3c32f0e63cb7242a&lust=63993970&luwtr=754962655298315806&mscf=0&n=10&nttp=1&p=baidu&pbs=220093&sce=7&sr=72&ssp2=1&tpl=baiduCustITagLinkUnitRankCol&tsf=dtp:1&tu_type=0&u=%2Fitem%2F%25E6%2585%25A2%25E6%2580%25A7%25E5%258A%259F%25E8%2583%25BD%25E6%2580%25A7%25E4%25BE%25BF%25E7%25A7%2598%3FfromModule%3Dlemma%5Fsearch%2Dbox&uicf=lurecv&urlid=0&eot=1) [无人机反制](https://cpro.baidu.com/cpro/ui/uijs.php?en=mywWUA71T1YsFh7sT7qGujYsFhPC5H0huAbqrauGTdq9TZ0qnauJp1YzmHcYmWT1mvRvuW01nWPWFh_qFRP7FRw7FRn3FRPaFRFaFRuKFRcLFRcYFRfvFRnvFhkdpvbqnHmhUyPsUHY3nHnsPzuk5HnLnjbYnHfsgvPsTBuzmWYsFMF15HDhTvN_UANzgv-b5HDhTv-b5HPWn1FhnARvnvPBP1cYnhDhTLwGujY3FMfqIZKWUA-WpvNbndqCmzuYujYkrHbLPWn1FMwV5Hcvrj6sn1R3niuYUgnqnHmLnjb3PW04PBuYIHddnHb4P1m1nzud5y9YIZK1FHPKFHFAFHFAmh7GpvR-nbNBmy-bIiRzwyPEUiuv5HchpHd-uHRsm1czrf&besl=6&c=news&cf=1&cvrq=1415933&eid_list=201577_203434_207574_208118_209357&expid=201577_202257_202564_203434_205809_207574_208118_209394&fr=20&fv=0&haacp=904&img_typ=0&itm=0&lu_idc=gzhxy&lukid=16&lus=3c32f0e63cb7242a&lust=63993970&luwtr=685006414410405008&mscf=0&n=10&nttp=1&p=baidu&pbs=220093&sce=7&sr=72&ssp2=1&tpl=baiduCustITagLinkUnitRankCol&tsf=dtp:1&tu_type=0&u=%2Fitem%2F%25E6%2585%25A2%25E6%2580%25A7%25E5%258A%259F%25E8%2583%25BD%25E6%2580%25A7%25E4%25BE%25BF%25E7%25A7%2598%3FfromModule%3Dlemma%5Fsearch%2Dbox&uicf=lurecv&urlid=0&eot=1) [自己创建个](https://cpro.baidu.com/cpro/ui/uijs.php?en=mywWUA71T1YsFh7sT7qGujYsFhPC5H0huAbqrauGTdq9TZ0qnauJp1YzmHcYmWT1mvRvuW01nWPWFh_qFRfLFRfYFRFjFRFKFRcYFRcYFRFDFRD3FRc3FRmvFRPDFRm3FRfdFRF7FhkdpvbqnHThUyPsUHYknWn1PjThTHY1P104PjDYn7qWTZchThcqnauzT1YkFMP-UAk-T-qGujYkFMPGujY1m1nzuWK-PWPWmWTzPjF9FMPYpyfqrauY5gwsmvkGmvV-ujPxpAnhIAfqnHb4P1m1nzuYUHYzPW63njndrjDhIAd15HDvP104rjmsrHmhIZRqIHD4rHTvn1nhIHdCIZwsTzR1fiRzwBRzwhF9pyV-FHF7mh7GuZR-nbNWUvYhIWYzFhbqPv7hnHIBuW0&besl=6&c=news&cf=1&cvrq=2235877&eid_list=201577_203434_207574_208118_209357&expid=201577_202257_202564_203434_205809_207574_208118_209394&fr=20&fv=0&haacp=1001&img_typ=0&itm=0&lu_idc=gzhxy&lukid=17&lus=3c32f0e63cb7242a&lust=63993970&luwtr=6556233252601032990&mscf=0&n=10&nttp=1&p=baidu&pbs=220093&sce=7&sr=72&ssp2=1&tpl=baiduCustITagLinkUnitRankCol&tsf=dtp:1&tu_type=0&u=%2Fitem%2F%25E6%2585%25A2%25E6%2580%25A7%25E5%258A%259F%25E8%2583%25BD%25E6%2580%25A7%25E4%25BE%25BF%25E7%25A7%2598%3FfromModule%3Dlemma%5Fsearch%2Dbox&uicf=lurecv&urlid=0&eot=1) [csgo电脑配](https://cpro.baidu.com/cpro/ui/uijs.php?en=mywWUA71T1YsFh7sT7qGujYsFhPC5H0huAbqrauGTdq9TZ0qnauJp1YzmHcYmWT1mvRvuW01nWPWFh_qmLPMUzNaPiN7PzNjPaNDPaNjPiN7PaNDPBNjnzu_IyVG5HD3FhdWTAYqP16zP1fhTHY1P104PjDYn7qWTZchThcqnauzT1YkFMP-UAk-T-qGujYkFMPGujY1m1nzuWK-PWPWmWTzPjF9FMPYpyfqrauY5gwsmvkGmvV-ujPxpAnhIAfqnHb4P1m1nzuYUHYzPW63njndrjDhIAd15HDvP104rjmsrHmhIZRqIHD4rHTvn1nhIHdCIZwsTzR1fiRzwBRzwhF9pyV-FHF7mh7GuZR-nbNWUvYhIWYzFhbqn1nvuhDduym&besl=6&c=news&cf=1&cvrq=3472466&eid_list=201577_203434_207574_208118_209357&expid=201577_202257_202564_203434_205809_207574_208118_209394&fr=20&fv=0&haacp=611&img_typ=0&itm=0&lu_idc=gzhxy&lukid=18&lus=3c32f0e63cb7242a&lust=63993970&luwtr=2357911270216075011&mscf=0&n=10&nttp=1&p=baidu&pbs=220093&sce=7&sr=72&ssp2=1&tpl=baiduCustITagLinkUnitRankCol&tsf=dtp:1&tu_type=0&u=%2Fitem%2F%25E6%2585%25A2%25E6%2580%25A7%25E5%258A%259F%25E8%2583%25BD%25E6%2580%25A7%25E4%25BE%25BF%25E7%25A7%2598%3FfromModule%3Dlemma%5Fsearch%2Dbox&uicf=lurecv&urlid=0&eot=1) [自己建个网](https://cpro.baidu.com/cpro/ui/uijs.php?en=mywWUA71T1YsFh7sT7qGujYsFhPC5H0huAbqrauGTdq9TZ0qnauJp1YzmHcYmWT1mvRvuW01nWPWFh_qFRfLFRfYFRFjFRFKFRFDFRD3FRc3FRmvFRPDFRm3FRfdFRF7FhkdpvbqnHbhUyPsUHYdPW0snBuk5HnLnjbYnHfsgvPsTBuzmWYsFMF15HDhTvN_UANzgv-b5HDhTv-b5HPWn1FhnARvnvPBP1cYnhDhTLwGujY3FMfqIZKWUA-WpvNbndqCmzuYujYkrHbLPWn1FMwV5Hcvrj6sn1R3niuYUgnqnHmLnjb3PW04PBuYIHddnHb4P1m1nzud5y9YIZK1FHPKFHFAFHFAmh7GpvR-nbNBmy-bIiRzwyPEUiuv5HchpHYYmW9WmWD1P6&besl=6&c=news&cf=1&cvrq=2719613&eid_list=201577_203434_207574_208118_209357&expid=201577_202257_202564_203434_205809_207574_208118_209394&fr=20&fv=0&haacp=1125&img_typ=0&itm=0&lu_idc=gzhxy&lukid=19&lus=3c32f0e63cb7242a&lust=63993970&luwtr=2471349030649590347&mscf=0&n=10&nttp=1&p=baidu&pbs=220093&sce=7&sr=72&ssp2=1&tpl=baiduCustITagLinkUnitRankCol&tsf=dtp:1&tu_type=0&u=%2Fitem%2F%25E6%2585%25A2%25E6%2580%25A7%25E5%258A%259F%25E8%2583%25BD%25E6%2580%25A7%25E4%25BE%25BF%25E7%25A7%2598%3FfromModule%3Dlemma%5Fsearch%2Dbox&uicf=lurecv&urlid=0&eot=1) [机器人展会](https://cpro.baidu.com/cpro/ui/uijs.php?en=mywWUA71T1YsFh7sT7qGujYsFhPC5H0huAbqrauGTdq9TZ0qnauJp1YzmHcYmWT1mvRvuW01nWPWFh_qFRFaFRuKFRnvFRmLFRn3FRPaFRfdFRc4FRFaFRRkFhkdpvbqnW0hUyPsUHYLPHb1riuk5HnLnjbYnHfsgvPsTBuzmWYsFMF15HDhTvN_UANzgv-b5HDhTv-b5HPWn1FhnARvnvPBP1cYnhDhTLwGujY3FMfqIZKWUA-WpvNbndqCmzuYujYkrHbLPWn1FMwV5Hcvrj6sn1R3niuYUgnqnHmLnjb3PW04PBuYIHddnHb4P1m1nzud5y9YIZK1FHPKFHFAFHFAmh7GpvR-nbNBmy-bIiRzwyPEUiuv5HchpHYLrjbdPjRLP6&besl=6&c=news&cf=1&cvrq=2360884&eid_list=201577_203434_207574_208118_209357&expid=201577_202257_202564_203434_205809_207574_208118_209394&fr=20&fv=0&haacp=1509&img_typ=0&itm=0&lu_idc=gzhxy&lukid=20&lus=3c32f0e63cb7242a&lust=63993970&luwtr=717749295403362800&mscf=0&n=10&nttp=1&p=baidu&pbs=220093&sce=7&sr=72&ssp2=1&tpl=baiduCustITagLinkUnitRankCol&tsf=dtp:1&tu_type=0&u=%2Fitem%2F%25E6%2585%25A2%25E6%2580%25A7%25E5%258A%259F%25E8%2583%25BD%25E6%2580%25A7%25E4%25BE%25BF%25E7%25A7%2598%3FfromModule%3Dlemma%5Fsearch%2Dbox&uicf=lurecv&urlid=0&eot=1) [什么叫云计](https://cpro.baidu.com/cpro/ui/uijs.php?en=mywWUA71T1YsFh7sT7qGujYsFhPC5H0huAbqrauGTdq9TZ0qnauJp1YzmHcYmWT1mvRvuW01nWPWFh_qFRPKFRczFRn1FRcYFRFDFRfsFRfYFRnvFRFjFRnvFRPaFRR1FhkdpvbqnWDhUyPsUHYLP1b3nzuk5HnLnjbYnHfsgvPsTBuzmWYsFMF15HDhTvN_UANzgv-b5HDhTv-b5HPWn1FhnARvnvPBP1cYnhDhTLwGujY3FMfqIZKWUA-WpvNbndqCmzuYujYkrHbLPWn1FMwV5Hcvrj6sn1R3niuYUgnqnHmLnjb3PW04PBuYIHddnHb4P1m1nzud5y9YIZK1FHPKFHFAFHFAmh7GpvR-nbNBmy-bIiRzwyPEUiuv5HchpHYsnhFhnjKBu0&besl=6&c=news&cf=1&cvrq=2481698&eid_list=201577_203434_207574_208118_209357&expid=201577_202257_202564_203434_205809_207574_208118_209394&fr=20&fv=0&haacp=894&img_typ=0&itm=0&lu_idc=gzhxy&lukid=21&lus=3c32f0e63cb7242a&lust=63993970&luwtr=2262954635798908161&mscf=0&n=10&nttp=1&p=baidu&pbs=220093&sce=7&sr=72&ssp2=1&tpl=baiduCustITagLinkUnitRankCol&tsf=dtp:1&tu_type=0&u=%2Fitem%2F%25E6%2585%25A2%25E6%2580%25A7%25E5%258A%259F%25E8%2583%25BD%25E6%2580%25A7%25E4%25BE%25BF%25E7%25A7%2598%3FfromModule%3Dlemma%5Fsearch%2Dbox&uicf=lurecv&urlid=0&eot=1) [动物焚烧炉](https://cpro.baidu.com/cpro/ui/uijs.php?en=mywWUA71T1YsFh7sT7qGujYsFhPC5H0huAbqrauGTdq9TZ0qnauJp1YzmHcYmWT1mvRvuW01nWPWFh_qFRcvFR7AFRP7FRNAFRcLFRf4FRn4FRfdFRnzFR7AFhkdpvbqnWchUyPsUHYdnHnsnauk5HnLnjbYnHfsgvPsTBuzmWYsFMF15HDhTvN_UANzgv-b5HDhTv-b5HPWn1FhnARvnvPBP1cYnhDhTLwGujY3FMfqIZKWUA-WpvNbndqCmzuYujYkrHbLPWn1FMwV5Hcvrj6sn1R3niuYUgnqnHmLnjb3PW04PBuYIHddnHb4P1m1nzud5y9YIZK1FHPKFHFAFHFAmh7GpvR-nbNBmy-bIiRzwyPEUiuv5HchpHY1rAuBuWT3P0&besl=6&c=news&cf=1&cvrq=3128930&eid_list=201577_203434_207574_208118_209357&expid=201577_202257_202564_203434_205809_207574_208118_209394&fr=20&fv=0&haacp=386&img_typ=0&itm=0&lu_idc=gzhxy&lukid=22&lus=3c32f0e63cb7242a&lust=63993970&luwtr=751581624084886335&mscf=0&n=10&nttp=1&p=baidu&pbs=220093&sce=7&sr=72&ssp2=1&tpl=baiduCustITagLinkUnitRankCol&tsf=dtp:1&tu_type=0&u=%2Fitem%2F%25E6%2585%25A2%25E6%2580%25A7%25E5%258A%259F%25E8%2583%25BD%25E6%2580%25A7%25E4%25BE%25BF%25E7%25A7%2598%3FfromModule%3Dlemma%5Fsearch%2Dbox&uicf=lurecv&urlid=0&eot=1)  **1**  **2**  **3**  **4**  **5**  **6**  **7**  **8**  **9**  **10**  **11**  **12**  **13**  **14**  **15**  **16**  **17**  **18**  **19**  **20**  **21**  **22**   \| [自己怎样建网](https://cpro.baidu.com/cpro/ui/uijs.php?en=mywWUA71T1YsFh7sT7qGujYsFhPC5H0huAbqrauGTdq9TZ0qnauJp1YzmHcYmWT1mvRvuW01nWPWFh_qFRfLFRfYFRFjFRFKFRfYFRmdFRfkFRm4FRFDFRD3FRPDFRm3FRfdFRF7FhkdpvbqniuVmLKV5Hm4P1RsFMDqn1TsrHfkPjKxmLKzFMFB5H0hTMnqniu1uyk_ugFxpyfqniu1pyfqnvn1nhmsuHm1mvcLnWfzmiu1IA-b5H6hIjdYTAP_pyPouyf1gv9WFMwb5HD4rHTvn1nhIAYqnWm3rj01PH6kFMwVT1YkPWTsrH6vnjbvFMwd5gRkrHbLPWn1FMRqpZwYTZn-nYD-nbm-nbuBmy-ouiRzwyF9pywdFHF7mvqVFMmqnBuG5H63nHckmHR3&besl=6&c=news&cf=1&cvrq=2061742&eid_list=201577_203434_207574_208118_209357&expid=201577_202257_202564_203434_205809_207574_208118_209394&fr=20&fv=0&haacp=2362&img_typ=0&itm=0&lu_idc=gzhxy&lukid=1&lus=3c32f0e63cb7242a&lust=63993970&luwtr=6786335543231135880&mscf=0&n=10&nttp=1&p=baidu&pbs=220093&sce=7&sr=72&ssp2=1&tpl=baiduCustITagLinkUnitRankCol&tsf=dtp:1&tu_type=0&u=%2Fitem%2F%25E6%2585%25A2%25E6%2580%25A7%25E5%258A%259F%25E8%2583%25BD%25E6%2580%25A7%25E4%25BE%25BF%25E7%25A7%2598%3FfromModule%3Dlemma%5Fsearch%2Dbox&uicf=lurecv&urlid=0&eot=1)  [焊缝无损检测](https://cpro.baidu.com/cpro/ui/uijs.php?en=mywWUA71T1YsFh7sT7qGujYsFhPC5H0huAbqrauGTdq9TZ0qnauJp1YzmHcYmWT1mvRvuW01nWPWFh_qFRFKFRc3FRcLFRNjFRP7FRw7FRPaFRmsFRFjFRNjFRczFRRzFhkdpvbqnBuVmLKV5HDknjnvrauk5HnLnjbYnHfsgvPsTBuzmWYsFMF15HDhTvN_UANzgv-b5HDhTv-b5HPWn1FhnARvnvPBP1cYnhDhTLwGujY3FMfqIZKWUA-WpvNbndqCmzuYujYkrHbLPWn1FMwV5Hcvrj6sn1R3niuYUgnqnHmLnjb3PW04PBuYIHddnHb4P1m1nzud5y9YIZK1FHPKFHFAFHFAmh7GpvR-nbNBmy-bIiRzwyPEUiuv5HchpHYYryR4uycLns&besl=6&c=news&cf=1&cvrq=2411344&eid_list=201577_203434_207574_208118_209357&expid=201577_202257_202564_203434_205809_207574_208118_209394&fr=20&fv=0&haacp=1596&img_typ=0&itm=0&lu_idc=gzhxy&lukid=2&lus=3c32f0e63cb7242a&lust=63993970&luwtr=2196606844417253483&mscf=0&n=10&nttp=1&p=baidu&pbs=220093&sce=7&sr=72&ssp2=1&tpl=baiduCustITagLinkUnitRankCol&tsf=dtp:1&tu_type=0&u=%2Fitem%2F%25E6%2585%25A2%25E6%2580%25A7%25E5%258A%259F%25E8%2583%25BD%25E6%2580%25A7%25E4%25BE%25BF%25E7%25A7%2598%3FfromModule%3Dlemma%5Fsearch%2Dbox&uicf=lurecv&urlid=0&eot=1)  [价格便宜的香](https://cpro.baidu.com/cpro/ui/uijs.php?en=mywWUA71T1YsFh7sT7qGujYsFhPC5H0huAbqrauGTdq9TZ0qnauJp1YzmHcYmWT1mvRvuW01nWPWFh_qFRFjFRwaFRc3FRmkFRckFRR1FRfzFRPaFRcdFRnYFRPAFRR1FRc3FRwaFRcLFRu7FRP7FRmkFRnvFRmLFhkdpvbqnzuVmLKV5HT4PWDsFMDqn1TsrHfkPjKxmLKzFMFB5H0hTMnqniu1uyk_ugFxpyfqniu1pyfqnvn1nhmsuHm1mvcLnWfzmiu1IA-b5H6hIjdYTAP_pyPouyf1gv9WFMwb5HD4rHTvn1nhIAYqnWm3rj01PH6kFMwVT1YkPWTsrH6vnjbvFMwd5gRkrHbLPWn1FMRqpZwYTZn-nYD-nbm-nbuBmy-ouiRzwyF9pywdFHF7mvqVFMmqnBuG5HndPWFbP1Ih&besl=6&c=news&cf=1&cvrq=2363942&eid_list=201577_203434_207574_208118_209357&expid=201577_202257_202564_203434_205809_207574_208118_209394&fr=20&fv=0&haacp=1750&img_typ=0&itm=0&lu_idc=gzhxy&lukid=3&lus=3c32f0e63cb7242a&lust=63993970&luwtr=18274635138362951998&mscf=0&n=10&nttp=1&p=baidu&pbs=220093&sce=7&sr=72&ssp2=1&tpl=baiduCustITagLinkUnitRankCol&tsf=dtp:1&tu_type=0&u=%2Fitem%2F%25E6%2585%25A2%25E6%2580%25A7%25E5%258A%259F%25E8%2583%25BD%25E6%2580%25A7%25E4%25BE%25BF%25E7%25A7%2598%3FfromModule%3Dlemma%5Fsearch%2Dbox&uicf=lurecv&urlid=0&eot=1)  [电脑主机多少](https://cpro.baidu.com/cpro/ui/uijs.php?en=mywWUA71T1YsFh7sT7qGujYsFhPC5H0huAbqrauGTdq9TZ0qnauJp1YzmHcYmWT1mvRvuW01nWPWFh_qFRcdFRRLFRnYFRfYFRfvFRmLFRFaFRuKFRcvFRRsFRn4FRf4FRnLFR77FhkdpvbqPauVmLKV5HmLnjnLFMDqn1TsrHfkPjKxmLKzFMFB5H0hTMnqniu1uyk_ugFxpyfqniu1pyfqnvn1nhmsuHm1mvcLnWfzmiu1IA-b5H6hIjdYTAP_pyPouyf1gv9WFMwb5HD4rHTvn1nhIAYqnWm3rj01PH6kFMwVT1YkPWTsrH6vnjbvFMwd5gRkrHbLPWn1FMRqpZwYTZn-nYD-nbm-nbuBmy-ouiRzwyF9pywdFHF7mvqVFMmqnBuG5H63P1Phnymv&besl=6&c=news&cf=1&cvrq=2978018&eid_list=201577_203434_207574_208118_209357&expid=201577_202257_202564_203434_205809_207574_208118_209394&fr=20&fv=0&haacp=217&img_typ=0&itm=0&lu_idc=gzhxy&lukid=4&lus=3c32f0e63cb7242a&lust=63993970&luwtr=6400949698628764850&mscf=0&n=10&nttp=1&p=baidu&pbs=220093&sce=7&sr=72&ssp2=1&tpl=baiduCustITagLinkUnitRankCol&tsf=dtp:1&tu_type=0&u=%2Fitem%2F%25E6%2585%25A2%25E6%2580%25A7%25E5%258A%259F%25E8%2583%25BD%25E6%2580%25A7%25E4%25BE%25BF%25E7%25A7%2598%3FfromModule%3Dlemma%5Fsearch%2Dbox&uicf=lurecv&urlid=0&eot=1)  [蓄热式焚烧炉](https://cpro.baidu.com/cpro/ui/uijs.php?en=mywWUA71T1YsFh7sT7qGujYsFhPC5H0huAbqrauGTdq9TZ0qnauJp1YzmHcYmWT1mvRvuW01nWPWFh_qFRfsFRN7FRn3FRn3FRPKFRFDFRcLFRf4FRn4FRfdFRnzFR7AFhkdpvbqPiuVmLKV5HRvnHDvFMDqn1TsrHfkPjKxmLKzFMFB5H0hTMnqniu1uyk_ugFxpyfqniu1pyfqnvn1nhmsuHm1mvcLnWfzmiu1IA-b5H6hIjdYTAP_pyPouyf1gv9WFMwb5HD4rHTvn1nhIAYqnWm3rj01PH6kFMwVT1YkPWTsrH6vnjbvFMwd5gRkrHbLPWn1FMRqpZwYTZn-nYD-nbm-nbuBmy-ouiRzwyF9pywdFHF7mvqVFMmqnBuG5H7-rjm4PHR3&besl=6&c=news&cf=1&cvrq=2461324&eid_list=201577_203434_207574_208118_209357&expid=201577_202257_202564_203434_205809_207574_208118_209394&fr=20&fv=0&haacp=1686&img_typ=0&itm=0&lu_idc=gzhxy&lukid=5&lus=3c32f0e63cb7242a&lust=63993970&luwtr=2270768104828311148&mscf=0&n=10&nttp=1&p=baidu&pbs=220093&sce=7&sr=72&ssp2=1&tpl=baiduCustITagLinkUnitRankCol&tsf=dtp:1&tu_type=0&u=%2Fitem%2F%25E6%2585%25A2%25E6%2580%25A7%25E5%258A%259F%25E8%2583%25BD%25E6%2580%25A7%25E4%25BE%25BF%25E7%25A7%2598%3FfromModule%3Dlemma%5Fsearch%2Dbox&uicf=lurecv&urlid=0&eot=1)  [高温隔热材料](https://cpro.baidu.com/cpro/ui/uijs.php?en=mywWUA71T1YsFh7sT7qGujYsFhPC5H0huAbqrauGTdq9TZ0qnauJp1YzmHcYmWT1mvRvuW01nWPWFh_qFRc3FRwAFRP7FRnzFRc3FRmYFRn3FRn3FRczFRnYFRnkFRPAFhkdpvbqPBuVmLKV5HTsnjmLFMDqn1TsrHfkPjKxmLKzFMFB5H0hTMnqniu1uyk_ugFxpyfqniu1pyfqnvn1nhmsuHm1mvcLnWfzmiu1IA-b5H6hIjdYTAP_pyPouyf1gv9WFMwb5HD4rHTvn1nhIAYqnWm3rj01PH6kFMwVT1YkPWTsrH6vnjbvFMwd5gRkrHbLPWn1FMRqpZwYTZn-nYD-nbm-nbuBmy-ouiRzwyF9pywdFHF7mvqVFMmqnBuG5yRdPHTLmvf1&besl=6&c=news&cf=1&cvrq=2582209&eid_list=201577_203434_207574_208118_209357&expid=201577_202257_202564_203434_205809_207574_208118_209394&fr=20&fv=0&haacp=1183&img_typ=0&itm=0&lu_idc=gzhxy&lukid=6&lus=3c32f0e63cb7242a&lust=63993970&luwtr=2200308873988409662&mscf=0&n=10&nttp=1&p=baidu&pbs=220093&sce=7&sr=72&ssp2=1&tpl=baiduCustITagLinkUnitRankCol&tsf=dtp:1&tu_type=0&u=%2Fitem%2F%25E6%2585%25A2%25E6%2580%25A7%25E5%258A%259F%25E8%2583%25BD%25E6%2580%25A7%25E4%25BE%25BF%25E7%25A7%2598%3FfromModule%3Dlemma%5Fsearch%2Dbox&uicf=lurecv&urlid=0&eot=1)  [哈佛大学申请](https://cpro.baidu.com/cpro/ui/uijs.php?en=mywWUA71T1YsFh7sT7qGujYsFhPC5H0huAbqrauGTdq9TZ0qnauJp1YzmHcYmWT1mvRvuW01nWPWFh_qFRc4FRu7FRcLFRmsFRcYFRm1FRfkFRDLFRn4FRNKFRnLFRNaFRPjFRmdFRFjFRu7FhkdpvbqPzuVmLKV5HbknHnLFMDqn1TsrHfkPjKxmLKzFMFB5H0hTMnqniu1uyk_ugFxpyfqniu1pyfqnvn1nhmsuHm1mvcLnWfzmiu1IA-b5H6hIjdYTAP_pyPouyf1gv9WFMwb5HD4rHTvn1nhIAYqnWm3rj01PH6kFMwVT1YkPWTsrH6vnjbvFMwd5gRkrHbLPWn1FMRqpZwYTZn-nYD-nbm-nbuBmy-ouiRzwyF9pywdFHF7mvqVFMmqnBuG5H04nhwbuHwW&besl=6&c=news&cf=1&cvrq=2024364&eid_list=201577_203434_207574_208118_209357&expid=201577_202257_202564_203434_205809_207574_208118_209394&fr=20&fv=0&haacp=870&img_typ=0&itm=0&lu_idc=gzhxy&lukid=7&lus=3c32f0e63cb7242a&lust=63993970&luwtr=7841139350953723636&mscf=0&n=10&nttp=1&p=baidu&pbs=220093&sce=7&sr=72&ssp2=1&tpl=baiduCustITagLinkUnitRankCol&tsf=dtp:1&tu_type=0&u=%2Fitem%2F%25E6%2585%25A2%25E6%2580%25A7%25E5%258A%259F%25E8%2583%25BD%25E6%2580%25A7%25E4%25BE%25BF%25E7%25A7%2598%3FfromModule%3Dlemma%5Fsearch%2Dbox&uicf=lurecv&urlid=0&eot=1)  [俄语口语学习](https://cpro.baidu.com/cpro/ui/uijs.php?en=mywWUA71T1YsFh7sT7qGujYsFhPC5H0huAbqrauGTdq9TZ0qnauJp1YzmHcYmWT1mvRvuW01nWPWFh_qFRcvFRNDFRf1FRNAFRFAFRwKFRf1FRNAFRfkFRDLFRPAFRcsFhkdpvbqrauVmLKV5HRLnWbzFMDqn1TsrHfkPjKxmLKzFMFB5H0hTMnqniu1uyk_ugFxpyfqniu1pyfqnvn1nhmsuHm1mvcLnWfzmiu1IA-b5H6hIjdYTAP_pyPouyf1gv9WFMwb5HD4rHTvn1nhIAYqnWm3rj01PH6kFMwVT1YkPWTsrH6vnjbvFMwd5gRkrHbLPWn1FMRqpZwYTZn-nYD-nbm-nbuBmy-ouiRzwyF9pywdFHF7mvqVFMmqnBuG5yRzmhFhmHRL&besl=6&c=news&cf=1&cvrq=2822133&eid_list=201577_203434_207574_208118_209357&expid=201577_202257_202564_203434_205809_207574_208118_209394&fr=20&fv=0&haacp=727&img_typ=0&itm=0&lu_idc=gzhxy&lukid=8&lus=3c32f0e63cb7242a&lust=63993970&luwtr=2414741795559411788&mscf=0&n=10&nttp=1&p=baidu&pbs=220093&sce=7&sr=72&ssp2=1&tpl=baiduCustITagLinkUnitRankCol&tsf=dtp:1&tu_type=0&u=%2Fitem%2F%25E6%2585%25A2%25E6%2580%25A7%25E5%258A%259F%25E8%2583%25BD%25E6%2580%25A7%25E4%25BE%25BF%25E7%25A7%2598%3FfromModule%3Dlemma%5Fsearch%2Dbox&uicf=lurecv&urlid=0&eot=1)  [新概念英语网](https://cpro.baidu.com/cpro/ui/uijs.php?en=mywWUA71T1YsFh7sT7qGujYsFhPC5H0huAbqrauGTdq9TZ0qnauJp1YzmHcYmWT1mvRvuW01nWPWFh_qFRfsFRnzFRc3FRndFRnYFRN7FRf1FRDzFRf1FRNAFRPDFRm3FRFAFRP7FhkdpvbqriuVmLKV5HDsPj0dnzuk5HnLnjbYnHfsgvPsTBuzmWYsFMF15HDhTvN_UANzgv-b5HDhTv-b5HPWn1FhnARvnvPBP1cYnhDhTLwGujY3FMfqIZKWUA-WpvNbndqCmzuYujYkrHbLPWn1FMwV5Hcvrj6sn1R3niuYUgnqnHmLnjb3PW04PBuYIHddnHb4P1m1nzud5y9YIZK1FHPKFHFAFHFAmh7GpvR-nbNBmy-bIiRzwyPEUiuv5HchpHd9mvwhrjP9u0&besl=6&c=news&cf=1&cvrq=3212251&eid_list=201577_203434_207574_208118_209357&expid=201577_202257_202564_203434_205809_207574_208118_209394&fr=20&fv=0&haacp=1071&img_typ=0&itm=0&lu_idc=gzhxy&lukid=9&lus=3c32f0e63cb7242a&lust=63993970&luwtr=6912353898211953940&mscf=0&n=10&nttp=1&p=baidu&pbs=220093&sce=7&sr=72&ssp2=1&tpl=baiduCustITagLinkUnitRankCol&tsf=dtp:1&tu_type=0&u=%2Fitem%2F%25E6%2585%25A2%25E6%2580%25A7%25E5%258A%259F%25E8%2583%25BD%25E6%2580%25A7%25E4%25BE%25BF%25E7%25A7%2598%3FfromModule%3Dlemma%5Fsearch%2Dbox&uicf=lurecv&urlid=0&eot=1)  [战队logo设计](https://cpro.baidu.com/cpro/ui/uijs.php?en=mywWUA71T1YsFh7sT7qGujYsFhPC5H0huAbqrauGTdq9TZ0qnauJp1YzmHcYmWT1mvRvuW01nWPWFh_qFRfdFRFDFRcvFRf1UAqMUzNjriN7raNafzNjPBu_IyVG5HDsFhdWTAYqrHm3PjnhTHY1P104PjDYn7qWTZchThcqnauzT1YkFMP-UAk-T-qGujYkFMPGujY1m1nzuWK-PWPWmWTzPjF9FMPYpyfqrauY5gwsmvkGmvV-ujPxpAnhIAfqnHb4P1m1nzuYUHYzPW63njndrjDhIAd15HDvP104rjmsrHmhIZRqIHD4rHTvn1nhIHdCIZwsTzR1fiRzwBRzwhF9pyV-FHF7mh7GuZR-nbNWUvYhIWYzFhbqmH0zuW9Bm1b&besl=6&c=news&cf=1&cvrq=1756706&eid_list=201577_203434_207574_208118_209357&expid=201577_202257_202564_203434_205809_207574_208118_209394&fr=20&fv=0&haacp=707&img_typ=0&itm=0&lu_idc=gzhxy&lukid=10&lus=3c32f0e63cb7242a&lust=63993970&luwtr=1863292750894598650&mscf=0&n=10&nttp=1&p=baidu&pbs=220093&sce=7&sr=72&ssp2=1&tpl=baiduCustITagLinkUnitRankCol&tsf=dtp:1&tu_type=0&u=%2Fitem%2F%25E6%2585%25A2%25E6%2580%25A7%25E5%258A%259F%25E8%2583%25BD%25E6%2580%25A7%25E4%25BE%25BF%25E7%25A7%2598%3FfromModule%3Dlemma%5Fsearch%2Dbox&uicf=lurecv&urlid=0&eot=1)  [10万级无尘车](https://cpro.baidu.com/cpro/ui/uijs.php?en=mywWUA71T1YsFh7sT7qGujYsFhPC5H0huAbqrauGTdq9TZ0qnauJp1YzmHcYmWT1mvRvuW01nWPWFh_qnH0-fYf-wWc-fbn-fWm-fYR-wDR-fWn-fbR-fWn-fWR-fbn-wHfhUZNopHYkniuVmLKV5HT3nW6LFMDqn1TsrHfkPjKxmLKzFMFB5H0hTMnqniu1uyk_ugFxpyfqniu1pyfqnvn1nhmsuHm1mvcLnWfzmiu1IA-b5H6hIjdYTAP_pyPouyf1gv9WFMwb5HD4rHTvn1nhIAYqnWm3rj01PH6kFMwVT1YkPWTsrH6vnjbvFMwd5gRkrHbLPWn1FMRqpZwYTZn-nYD-nbm-nbuBmy-ouiRzwyF9pywdFHF7mvqVFMmqnBuG5HIbPjNWPvcd&besl=6&c=news&cf=1&cvrq=3846261&eid_list=201577_203434_207574_208118_209357&expid=201577_202257_202564_203434_205809_207574_208118_209394&fr=20&fv=0&haacp=369&img_typ=0&itm=0&lu_idc=gzhxy&lukid=11&lus=3c32f0e63cb7242a&lust=63993970&luwtr=7318665999692295159&mscf=0&n=10&nttp=1&p=baidu&pbs=220093&sce=7&sr=72&ssp2=1&tpl=baiduCustITagLinkUnitRankCol&tsf=dtp:1&tu_type=0&u=%2Fitem%2F%25E6%2585%25A2%25E6%2580%25A7%25E5%258A%259F%25E8%2583%25BD%25E6%2580%25A7%25E4%25BE%25BF%25E7%25A7%2598%3FfromModule%3Dlemma%5Fsearch%2Dbox&uicf=lurecv&urlid=0&eot=1) \| \| --- \|  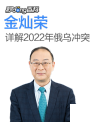 | 慢性功能性便秘_百度百科  [网页](https://www.baidu.com/) [新闻](http://news.baidu.com/) [贴吧](https://tieba.baidu.com/) [知道](https://zhidao.baidu.com/) [网盘](https://pan.baidu.com/?from=1027327l) [图片](http://image.baidu.com/) | [视频](http://v.baidu.com/) | [地图](http://map.baidu.com/) | [文库](https://wenku.baidu.com/) | 百科 | 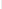 [百度首页](http://www.baidu.com/) [登录](javascript:;) |
| --- | --- | --- | --- | --- | --- | --- | --- | --- |

| [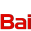岔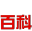](https://baike.baidu.com/) | \| 慢性功能性便秘 \| 进入词条 \| \| --- \| --- \| | \| 全站搜索 \| \| --- \| | [帮助](https://baike.baidu.com/help) |
| --- | --- | --- | --- | --- | --- | --- |
| 近期有不法分子冒充百度百科官方人员，以删除词条为由威胁并敲诈相关企业。在此严正声明：百度百科是免费编辑平台，绝不存在收费代编服务，请勿上当受骗！ [详情>>](https://baike.baidu.com/common/declaration) | | | |
| [首页](https://baike.baidu.com/) 秒懂百科 特色百科 用户 知识专题 权威合作 [口下载百科APP](https://baike.baidu.com/wapui/subpage/baikeappdownload?sfrom=pc_lemmapage_navigation) 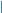 [2 个](https://baike.baidu.com/usercenter) | | | |

| [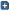](javascript:;)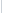 . 收藏 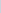[山 16](javascript:void(0);)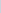 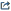 17  慢性功能性便秘 [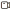上传视频](javascript:;)   \| 小播报 \| \| --- \|  \| !编辑 \| \| --- \|  \| 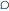讨论 \| \| --- \|   常见病  慢性功能性便秘是一种常见病、多发病。主要是指排便次数减少、粪便量减少、粪便干结、排便费力等。必须结合粪便的性  状、本人平时排便习惯和排便有无困难作出有无便秘的判断。如超过6个月即为[慢性便秘](https://baike.baidu.com/item/%E6%85%A2%E6%80%A7%E4%BE%BF%E7%A7%98/10430220?fromModule=lemma_inlink)。 | | | | | | |
| --- | --- | --- | --- | --- | --- | --- | --- | --- | --- |
| 多发群体  常见病因 | 老人，女性  饮食因素，精神压力 | | 西医学名 所属科室 | | 慢性功能性[便秘](https://baike.baidu.com/item/%E4%BE%BF%E7%A7%98/332148?fromModule=lemma_inlink)  [内科](https://baike.baidu.com/item/%E5%86%85%E7%A7%91/274870?fromModule=lemma_inlink) - 消化内科 | |
| 相关视频 | | 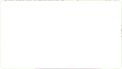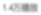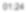 1.4万播放 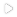 01:24 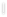 | | | | 查看全部 > |
| 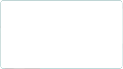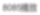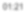 8085播放 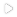 01:21 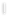 | |  |  |  |  |  |
|  |  | 有一种便秘叫“慢性功能性便  秘”，你要知道！ | | | |  |
| 如何治疗便秘？ | |  |  |  |  |  |
| \| 目录 \| 1 [诊断标准](#_bookmark4) 2 [病因病机](#_bookmark5) 3 [形成原因](#_bookmark6)  7 [辨证施治要点](#_bookmark7) 8 [慢性便秘治疗](#_bookmark8) 9 [不同病症区别](#_bookmark9)   \| 4 [食疗方法](#_bookmark1) \| \| --- \| \| 5 [治疗便秘方法](#_bookmark2) \| \| 6 [缓解便秘妙招](#_bookmark3) \| \| \| --- \| --- \| --- \| --- \| --- \| | | | | | | |
| 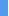 诊断标准 | | | | [小 播报!编辑](javascript:;) | | |
| 诊断标准：在过去的一年里至少3个月连续或间断出现以下2个或2个以上症状： (1)少于1/4的时间内有排便费力； (2)少于1/4  的时间内有有粪便干结； (3)少于1/4的时间内有[排便不尽感](https://baike.baidu.com/item/%E6%8E%92%E4%BE%BF%E4%B8%8D%E5%B0%BD%E6%84%9F/15786777?fromModule=lemma_inlink)； (4)少于1/4的时间内排便时有[肛门](https://baike.baidu.com/item/%E8%82%9B%E9%97%A8?fromModule=lemma_inlink)阻塞感或肛门[直肠](https://baike.baidu.com/item/%E7%9B%B4%E8%82%A0?fromModule=lemma_inlink)梗阻； (5)少于  1/4的时间内有排便需用手法协助； (6)少于1/4的时间内有每周排便少于3次。不存在稀便，也不符合肠易激综合征的诊断标准；  同时需除外肠道或全身器质性病因以及药物因素所致的便秘。 | | | | | | |
| 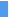 病因病机 | | | | [小 播报!编辑](javascript:;) | | |
| 《伤寒论》将便秘分为阳结、阴结、脾约。唐容川《血证论》云： “肺移热于大肠则便结，肺津不润则便结，肺气不降则便  结”。便秘的发生与大肠、脾、胃、肺、肝、肾等脏腑关系密切。《内经》云： “大肠者，传导之官，变化出焉。 ”又提出“肺与大肠  相表里”。脾胃为运化水谷之海，脾主运化，胃主和降，胃与肠相连，水谷入口，经脾的运化输布，胃的腐熟收纳，最后将糟粕转  输于大肠。这其中也依赖于肝主疏泄的功能，若肝郁气滞，则腑气不通，气滞不行，则大肠失运。肾司二便， [肾气不足](https://baike.baidu.com/item/%E8%82%BE%E6%B0%94%E4%B8%8D%E8%B6%B3/8615215?fromModule=lemma_inlink)，则大肠  传导无力，大便难于排出。一般认为，素体热盛，或恣食肥甘厚味，易致胃肠积热，耗伤津液，便燥难排；忧思恼怒，或久坐少  动，易导致气机郁滞，通降失常，糟粕内停；外感寒邪，过食寒凉，导致阴寒内盛，凝滞胃肠，糟粕传导不能；饮食劳倦，年老  体虚，大病产后，均可因体质的不同，而出现气虚阳衰、阴亏血少等情况，导致大肠传导无力，或肠道失濡，而致大便秘结。  西医学将功能性便秘分为慢传输型、出口梗阻型、混合型三种。中医认为，慢传输型便秘多为虚证便秘，如年老体弱、久病  产后、素体虚弱；或饮食劳倦、过食生冷、感受寒湿，伤及脾胃，均可导致肺、脾、肾诸脏虚弱。脾虚则运化无力，水谷精微不  得转输，糟粕无力运行于大肠。肺虚则宣降失常，肺与大肠相表里，大肠因而气机不利，运行受阻。华佗《中藏经》云： “大肠  者，肺之腑也，为传导之司，监仓之官。肺病久不已，则可下传大肠”，《医经精义 ·脏腑之言》曰： “大肠之所以能传导者，以其  为肺之腑。肺气下达，故能传导”。肾主水，司二便，肾虚则肠道失濡，开合不利，糟粕受阻。金元《兰室密藏 ·大便结燥》曰：  “夫肾主五液，津液润则大便如常。 ……又有年老体虚，津液不足而结燥者。 ”  出口[梗阻性便秘](https://baike.baidu.com/item/%E6%A2%97%E9%98%BB%E6%80%A7%E4%BE%BF%E7%A7%98/10430272?fromModule=lemma_inlink)多为实证便秘。如忧思恼怒， [肝郁气滞](https://baike.baidu.com/item/%E8%82%9D%E9%83%81%E6%B0%94%E6%BB%9E/3529334?fromModule=lemma_inlink)，过食生冷肥甘厚腻、辛辣刺激食物，或嗜烟酒，感外邪，根据体质  不同邪从热化或邪从寒化，导致或肠胃积热，或阴寒积滞，或气机郁滞，从而腑气不通，胃气不降，大肠失于传导。如《内经》  云： “太阴之厥，则腹胀而后不利”，提到便秘与脾胃受寒有关；《内经》云： “热气留于小肠，肠中痛，瘅热焦渴，则坚干不得 | | | | | | |

<https://baike.baidu.com/item/>慢性功能性便秘?fromModule=lemma_search-box

1/5

2022/12/14 10:48

慢性功能性便秘_百度百科

| 出，故痛而闭不通矣”，说明肠胃积热便秘的病理基础。肝主疏泄，有助于大肠传导， [肝气郁结](https://baike.baidu.com/item/%E8%82%9D%E6%B0%94%E9%83%81%E7%BB%93/6350942?fromModule=lemma_inlink)，则大肠气机不利，腑气不通。如  《金匮要略 ·便秘》云： “气秘者，气内滞，而物不行也。 ”  混合型便秘多为虚实夹杂型，或为虚中挟实性便秘，其本为虚，其标为实，本虚为脾、肺、肾虚弱，标实则为大肠气机不  利，腑气不通。  便秘的病因是复杂的，在疾病的发展过程中，各种证候可相兼出现，或互相转化。辨证应以虚实为纲，阴阳气血为目。其病  位在大肠，病机与脾胃肺肝肾有关。西医疾病分型与中医辨证分型有其内在规律可循，但因为疾病本身的多变性和复杂性，故不  能拘泥于此。治疗时应审证求因，审因论治。 | | | |
| --- | --- | --- | --- |
| 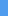 形成原因 | | [小 播报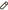编辑](javascript:;) | |
| 1、饮食因素：一些人饮食过少，食品过精过细，食物中的纤维素和水份不足，对肠道不能形成一定量的刺激，肠蠕动缓  慢，不能及时将食物残渣推向直肠，在肠内停留时间延长，水份过多吸收而使粪便干燥。  2、药物影响：长期滥用泻药，使肠壁神经感受细胞的应激性降低，即使肠内有足量粪便，也不能产生正常蠕动及排便反  射，因而导致顽固性[排便困难](https://baike.baidu.com/item/%E6%8E%92%E4%BE%BF%E5%9B%B0%E9%9A%BE/9752777?fromModule=lemma_inlink)。  3、大肠病变：如过敏性结肠炎、大肠憩室炎、先天性巨结肠等疾病可引起大肠痉挛、运动失常，使粪便通过不畅而发生排  便困难。  4、拖延大便时间：一些人把大便当作无关紧要，可早可迟的事，忽视定时排便的习惯，使直肠壁上的神经细胞对粪便进入  直肠后产生的压力感受反应变迟钝，使粪便在直肠内停留时间延长而不引起排便感觉，形成习惯性排便困难。  5、排便动力不足：排便时不仅需要肛门括约肌的舒张，提肛肌向上向外牵拉，而且还需要膈肌下降、腹肌收缩、屏气用力  来推动粪便排出。年老体弱、久病卧床、产后等，可因膈肌、腹肌、肛门括约肌收缩力减弱，腹压降低而使排便动力不足，使粪  便排不干净，粪块残留，发生大便困难。  6、精神因素：精神上受到强烈刺激、惊恐、情绪紧张、忧愁焦虑或注意力高度集中某一工作等会使便意消失，形成排便困  难。另外还有神经系统障碍、 [内分泌紊乱](https://baike.baidu.com/item/%E5%86%85%E5%88%86%E6%B3%8C%E7%B4%8A%E4%B9%B1/2427719?fromModule=lemma_inlink)、维生素缺乏等亦可引起排便困难 [1] 。 | | | |
|  | 食疗方法  [小 播报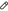编辑](javascript:;)  便秘患者多吃什么好   \|  \| \| --- \|   1、每日至少喝8杯水，尤其在食用高纤维食品时，更应注意保证饮水；  2、多吃新鲜蔬菜，增加饮食中纤维的摄取量；  3、每天加食糠皮、麦麸等，以扩充粪便体积，促进肠蠕动，减少便秘的发生；  4、每天早上起来空腹喝温水冲的蜂蜜水、蜂蜜对肠道有润滑作用；  5、增加B 族维生素食品的供给，尽量选用天然、未经加工的食品，如粗粮、豆类、  酵母等，以增强肠道的紧张力；  6、多吃芹菜、核桃仁、熟香蕉、核桃、柚子、苹果、葡萄柚、糙米、胡萝卜、红薯等。  治疗便秘方法  [小 播报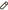编辑](javascript:;)  疗方1： [芝麻](https://baike.baidu.com/item/%E8%8A%9D%E9%BA%BB/64577?fromModule=lemma_inlink) (黑芝麻最佳)焙熟研细末2 ~ 3羹匙，兑温开水(开水待温后) 200 ~   \|  \| \| --- \|   300毫升加10天成熟洋槐蜂蜜3 ~ 5羹匙调成糊状口服，早、晚各1次。用此疗方治疗习惯  性便秘50例，见效最快者2天，最长者10余天， 2 ~ 10天见效40例， 10天以上见效10例，  50例均获良效。  疗方2： [蜂蜜](https://baike.baidu.com/item/%E8%9C%82%E8%9C%9C?fromModule=lemma_inlink)60克，每日早、晚各服30克，以凉开水冲饮。适用于老年、孕妇便秘及  习惯性便秘。  疗方3：蜂蜜60克，蜂王浆6克，将其调匀，每日早、晚分2次用温开水送服，适用于  习惯性便秘。 | | [女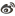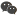疊 口](javascript:void(0);) |
|  |  |  |  |
|  | 疗方4：蜂花粉150克研碎，加入蜂蜜250克调成膏，每日早、晚空腹服1汤匙，适用于习惯性便秘。  疗方5： 10天成熟[洋槐花蜂蜜](https://baike.baidu.com/item/%E6%B4%8B%E6%A7%90%E8%8A%B1%E8%9C%82%E8%9C%9C?fromModule=lemma_inlink)、白萝卜适量，先将白萝卜洗净切成片，蘸蜂蜜生食，每日数次。最适用于青少年便秘者。  疗方6：蜂蜜、香蕉适量，将香蕉剥皮以其肉蘸蜂蜜生食，每日数次。最适用于老年人及[习惯性便秘](https://baike.baidu.com/item/%E4%B9%A0%E6%83%AF%E6%80%A7%E4%BE%BF%E7%A7%98?fromModule=lemma_inlink)者。 | |  |
|  |  |  |  |
|  | 缓解便秘妙招  [小 播报编辑](javascript:;)  跳绳可缓解便秘 | |  |

<https://baike.baidu.com/item/>慢性功能性便秘?fromModule=lemma_search-box

2/5

2022/12/14 10:48

慢性功能性便秘_百度百科

| 女性发生便秘的几率要比男性高。首先，因为女性吃东西少、细、精，因此肠道蠕动 更慢。其次，女性运动量较小，而坚持规律的锻炼可以使肠道更好地蠕动。  防治便秘最好的方法就是通过跳绳振动内脏。弹跳能刺激骨骼、肌肉，促进血液循 环，此外还能加强淋巴系统的免疫功能，这对缓解便秘十分重要。  便秘的人走路时，可以尽量加大腰和胯部的转动，像模特一样走猫步，这能起到对腹 | |  |  | |
| --- | --- | --- | --- | --- |
| 腔按摩的作用，能够加强内脏特别是肠胃的蠕动，促进营养的吸收和废弃物的排出，对肠  胃功能失常、 [消化不良](https://baike.baidu.com/item/%E6%B6%88%E5%8C%96%E4%B8%8D%E8%89%AF?fromModule=lemma_inlink)引起的便秘，有特别明显的疗效。  卫生间装饰成淡黄色有助排便  日本新研究发现，卫生间里装饰一些黄色，有助于排便。黄色是暖色调，有放松心情、缓解紧张情绪的作用。人们在排便 时，身体部分肌肉处于紧张收缩状态，这时保持愉快的心情，能放松肌肉，有助排便。卫生间里的颜色选暖色调，比如淡黄色、 淡绿色、淡蓝色、淡橙色等，可以帮你安神，而大紫大红的颜色则容易刺激神经，不利于精神的放松。在空间相对狭窄的浴室 里，黄颜色还能起到开阔视野的效果，使得整个卫生间更加柔和、宽敞和明亮。  喝醋防便秘  醋的酸性成分与胃里的消化液相似，故而食醋可以起到刺激肠胃、促进肠道蠕动的作用。而随之增强的排便感，可避免大便 在体内长时间存留、干结，对预防便秘有不错的效果。人在饱腹时，肠道被填满，醋不易刺激到肠胃，故食醋通便，最好选在早 晚空腹时。  食用时，每次不要超过1汤匙，但也不能少于半汤匙， 1天最多不能超过3次。  食用后，紧接着要饮一杯温开水。因为原汁醋的浓度太大，如果食用过多，可能会对胃黏膜造成一定损伤，所以一定要掌握 好醋的食用量。  也可在一汤匙陈醋中加入等量的蜂蜜，用温开水或凉开水均匀搅拌后喝下。  葡萄干治疗便秘  葡萄干治便秘的说法，在古代医书中并没有记载，至今相关报道也极少，而且对其有两种相反观点：有报道称，葡萄干含有 纤维和酒石酸，能让排泄物快速通过直肠，每天吃一小把可防治便秘；也有人认为，葡萄干多食“易生内热”，反而会加重便秘。  [但这两种观点，现均没有可靠的实验或临床研究加以证明。实验表明，从葡萄籽中提炼的葡萄籽油，对动物可以起到润肠通 便功效。](https://baike.baidu.com/item/%E6%B6%A6%E8%82%A0%E9%80%9A%E4%BE%BF/10853569?fromModule=lemma_inlink)  但市售葡萄干多为无籽的，治好便秘的几率极小，即使偶尔有效，也只是个例，并不说明此方法对其他人也有效，不宜效 法。  起床后喝杯白开水  晨起一杯白开水可以补充身体代谢失去的水分，又可以促进排泄，防治便秘。还能稀释血液，促进血液循环，对减肥女性尤 为重要。也可以用淡盐水代替白开水。  洗澡巧揉肚子治便秘  洗澡时可以用手掌在腹部按顺时针方向按摩，同时腹部一鼓一收地大口呼吸，并淋浴腹部，可治疗[慢性便秘](https://baike.baidu.com/item/%E6%85%A2%E6%80%A7%E4%BE%BF%E7%A7%98/10430220?fromModule=lemma_inlink)并防治痔疮。而 神经性便秘，则要沿着肠部用40℃热水冲3分钟左右，再用25℃的温水冲10秒钟，反复5次，可让大肠的蠕动增加。  核桃蜂蜜茶治疗便秘  核桃蜂蜜茶是民间常用的便秘偏方，有很好的治疗便秘的疗效。具体的方法是，用核桃5克，蜂蜜1克，用温开水冲泡后代茶 喝，即可达到润肠通便的效果。 | | | | [女疊 口](javascript:void(0);) |
| 辨证施治要点 | | [小 播报编辑](javascript:;) | |  |
| 1.重视脾阴，液增舟行  朱丹溪《局方发挥》云： “脾土之阴受伤，传输之官失职”。《素问 ·玉机真脏论篇》  云： “脾不足，令人九窍不通”。《类经》训诂曰： “不足病在中，故令九窍不通，以脾气弱 者四脏皆弱而气不行也。 ” 《金匮要略》认为，脾阴不足，不能为胃行其津液，肠道失 润，即形成脾约之证，导致便秘。《脾胃论》曰： “胃者卫之源，脾乃营之本”、“四季脾旺 不受邪”，营为阴，脾阴充足，则脾转输功能正常，精微得以化生，糟粕得以传导。长期 [习惯性便秘](https://baike.baidu.com/item/%E4%B9%A0%E6%83%AF%E6%80%A7%E4%BE%BF%E7%A7%98?fromModule=lemma_inlink)患者，尤其是老年患者，多为脾气或脾阴不足，或[气阴两虚](https://baike.baidu.com/item/%E6%B0%94%E9%98%B4%E4%B8%A4%E8%99%9A/9116833?fromModule=lemma_inlink)。治疗时应顾护脾 气脾阴，润肠通便。健脾气多用[四君子汤](https://baike.baidu.com/item/%E5%9B%9B%E5%90%9B%E5%AD%90%E6%B1%A4/4460067?fromModule=lemma_inlink)，养脾阴多用沙参麦门冬汤。  2.通降和胃，积滞自除  “胃者，五脏六腑之海也，水谷皆入于胃，五脏六腑皆禀气于胃”， “胃者人之常气，人 | \|  \| \| --- \| | | |  |
| 无胃气曰逆，逆者死。 ”李杲也说： “内伤脾胃，百病由生。 ”胃以通降为和，胃气和则浊气下降，糟粕得以排出大肠。通降可用枳 实、厚朴、莱菔子，和胃可用生姜、大枣、甘草。 | | | |  |

<https://baike.baidu.com/item/>慢性功能性便秘?fromModule=lemma_search-box

3/5

2022/12/14 10:48

慢性功能性便秘_百度百科

| 3.审证求因，用药有别  [女](javascript:void(0);) [疊](http://baike.baidu.com/l/WWoXYu7P) [口](javascript:void(0);)  在治疗功能性便秘时，应因证择方，因人施药。脾虚型便秘，大剂量用生白术30 ~ 60g以健脾补气通便；血虚肠燥者大剂量  用生白芍、火麻仁以补血润肠通便；肾阴虚者重用何首乌、生地以补肾滋润通便；肾阳虚者重用肉苁蓉以温补肾阳通便；脾气虚  重用生黄芪30 ~ 50g；痰热壅肺、肺失宣降者加用杏仁、黄芩、栝楼仁以宣肺清热通便；大肠实热者用大黄、虎杖以清热泻腑通  便；久病多瘀者则用桃仁、酒大黄以活血化瘀通便；肝郁气滞者重用郁金、枳实以疏肝理气通便。  [小 播报编辑](javascript:;)  慢性便秘治疗  首先应加强科学的生活管理，保持良好的精神及心理状态，注意高速饮食结构，多食富含纤维的食物，足量饮水，养成按时  排便习惯，参加适当的体育锻炼等，还可进行腹式呼吸锻炼或按摩。  1.药物治疗：(1)通过上述方法达不到疗效时可考虑药物治疗，对于STC患者，首选是促动力剂， [西沙必利](https://baike.baidu.com/item/%E8%A5%BF%E6%B2%99%E5%BF%85%E5%88%A9/3204058?fromModule=lemma_inlink)作为一种全胃肠  道促动力剂，对某些STC患者有效。一种新型特异性促肠动力药普卡必利晚近已问世，该药系苯并呋喃族化合物，特异性作用于  5-HT4受体，可望成为一种理想的治疗CFC的药物。 Soffer等[10]报道， misoprostol可用于治疗[顽固性便秘](https://baike.baidu.com/item/%E9%A1%BD%E5%9B%BA%E6%80%A7%E4%BE%BF%E7%A7%98/1302553?fromModule=lemma_inlink)。某些中药可能具有促  肠动力作用，也可选择性应用。(2)常用泻剂：①容量性泻药：硫酸镁、硫酸钠、 [甲基纤维素](https://baike.baidu.com/item/%E7%94%B2%E5%9F%BA%E7%BA%A4%E7%BB%B4%E7%B4%A0/10596970?fromModule=lemma_inlink)、琼脂等；②刺激性泻剂：番泻  叶、蓖麻油、双酯酚汀等；③粪便软化剂：液体石蜡、乳果糖等；④直肠内给药：甘油栓、开塞露等。应避免长期滥用泻剂而导  致泻剂性肠病。  2.生物反馈排便行为治疗：生物反馈治疗法是一种纠正不协调排便行为的训练法，主要用于治疗肛门括约肌失协调和盆底  肌、 [肛门外括约肌](https://baike.baidu.com/item/%E8%82%9B%E9%97%A8%E5%A4%96%E6%8B%AC%E7%BA%A6%E8%82%8C/580939?fromModule=lemma_inlink)排便时矛盾性收缩导致的FOOC，有人报告其疗效可达96%，该法与药物治疗相比具有无药物副作用、成本  低、非创伤性等优点，国内已开展此项疗法。  3.手术治疗：对于严重慢性便秘经上述治疗无效，严重影响患者生活质量甚至营养障碍者，可采取手术治疗。主要是对STC  患者，可采取[结肠](https://baike.baidu.com/item/%E7%BB%93%E8%82%A0?fromModule=lemma_inlink)次全切除和回直肠吻合术，对FOOC患者可行肛管直肠括约肌切除术。这种手术虽然可恢复肠管的节律性蠕  动，缩短全胃肠通过时间，但必须严格掌握适应证，同时也必须重视术后并发症如腹泻、 [大便失禁](https://baike.baidu.com/item/%E5%A4%A7%E4%BE%BF%E5%A4%B1%E7%A6%81?fromModule=lemma_inlink)、甚至复发等问题。  [小 播报编辑](javascript:;)  不同病症区别  功能性便秘(functionalconstipation)和盆底功能障碍(pelvicfloordyssynergia)：前者指无器质性疾病证据的慢性便秘，部分功  能性便秘患者有盆底障碍的表现，后者需伴有盆底障碍的表现，即符合罗马II的功能性便秘的诊断标准，还需具备盆底障碍的客  观依据。  (1)有肛门直肠测压，肌电图或X线检查的证据，表明在反复作排便动作时，盆底肌群不合适的收缩或不能放松。  (2)力排时直肠能出现足够的推进性收缩。  (3)并有粪便排出不畅的证据。  [词条图册 更多图册 >](https://baike.baidu.com/pic/%E6%85%A2%E6%80%A7%E5%8A%9F%E8%83%BD%E6%80%A7%E4%BE%BF%E7%A7%98/8952323?fr=lemma)   \| 参考资料 \| \| \| \| --- \| --- \| --- \| \| 1  [引起大便困难的原因](https://baike.baidu.com/reference/8952323/d534pBFZSmjcEux9giMW6puudTdzEyH3Qc73SKqfBYVAb3IJ8b0oLE7ELq4JPFO5FhM0DGVph4mT48aDuy_6PiAcvcK_QhVZ6-5zfRlYGbFEpK2ToKRYkA)  ．新浪健康[引用日期2014-09-14]  学术论文 \| 内容来自 \|  \|   [于金源，孙长岗，尹国富. 从肝论治慢性功能性便秘．](https://xueshu.baidu.com/usercenter/paper/show?paperid=c159fab5fe04c6d800eee2482893d9d2&tn=SE_baiduxueshu_c1gjeupa&ie=utf-8&site=baike) 《WanFang》， 2004  [金洵，丁义江，王玲玲等. 针刺治疗慢性功能性便秘疗效观察．](https://xueshu.baidu.com/usercenter/paper/show?paperid=08ea5c676b12a77cebaea0b09289a4ff&tn=SE_baiduxueshu_c1gjeupa&ie=utf-8&site=baike) 《CNKI;WanFang》， 2010  [王丽娟，王玲玲. 麦粒灸结合针刺治疗慢性功能性便秘随机对照研究．](https://xueshu.baidu.com/usercenter/paper/show?paperid=a83daa8478be44b3f132313ad0939954&tn=SE_baiduxueshu_c1gjeupa&ie=utf-8&site=baike) 《CNKI;WanFang》， 2011  [施永敏，周永香，马文芳等. 老年慢性功能性便秘的干预护理．](https://xueshu.baidu.com/usercenter/paper/show?paperid=3d1a2ef60c684f1493b52e22a91f78c8&tn=SE_baiduxueshu_c1gjeupa&ie=utf-8&site=baike) 《实用临床医药杂志》， 2005  [郭荣，丁义江，张建淮. 慢性功能性便秘诊断和中医证型的量化研究．](https://xueshu.baidu.com/usercenter/paper/show?paperid=d18bbb7c4b527ec929dd0b6efe923447&tn=SE_baiduxueshu_c1gjeupa&ie=utf-8&site=baike) 《 CNKI》， 2011  [查看全部](https://xueshu.baidu.com/s?wd=intitle%3A%28%E6%85%A2%E6%80%A7%E5%8A%9F%E8%83%BD%E6%80%A7%E4%BE%BF%E7%A7%98%29&tn=SE_baiduxueshu_c1gjeupa&ie=utf-8&sc_from=pingtai6&site=baike) |
| --- | --- | --- | --- | --- | --- | --- |
|  |

<https://baike.baidu.com/item/>慢性功能性便秘?fromModule=lemma_search-box

4/5

2022/12/14 10:48

[女](http://baike.baidu.com/l/WWoXYu7P)  [口](javascript:void(0);)

慢性功能性便秘_百度百科

| 岔 搜索发现  [功能性便秘怎么治疗](https://www.baidu.com/s?word=%E5%8A%9F%E8%83%BD%E6%80%A7%E4%BE%BF%E7%A7%98%E6%80%8E%E4%B9%88%E6%B2%BB%E7%96%97&tn=SE_baikepcxf02_fcetbk02&pos=baike_pc_turbo_1767&ori_sid=00bb3586ac9751ad)  [为什么老是便秘](https://www.baidu.com/s?word=%E4%B8%BA%E4%BB%80%E4%B9%88%E8%80%81%E6%98%AF%E4%BE%BF%E7%A7%98&tn=SE_baikepcxf02_fcetbk02&pos=baike_pc_turbo_1767&ori_sid=00bb3586ac9751ad) | [便秘的解决方法](https://www.baidu.com/s?word=%E4%BE%BF%E7%A7%98%E7%9A%84%E8%A7%A3%E5%86%B3%E6%96%B9%E6%B3%95&tn=SE_baikepcxf02_fcetbk02&pos=baike_pc_turbo_1767&ori_sid=00bb3586ac9751ad)  [预防便秘的方法有哪些](https://www.baidu.com/s?word=%E9%A2%84%E9%98%B2%E4%BE%BF%E7%A7%98%E7%9A%84%E6%96%B9%E6%B3%95%E6%9C%89%E5%93%AA%E4%BA%9B&tn=SE_baikepcxf02_fcetbk02&pos=baike_pc_turbo_1767&ori_sid=00bb3586ac9751ad) | [慢性便秘的症状](https://www.baidu.com/s?word=%E6%85%A2%E6%80%A7%E4%BE%BF%E7%A7%98%E7%9A%84%E7%97%87%E7%8A%B6&tn=SE_baikepcxf02_fcetbk02&pos=baike_pc_turbo_1767&ori_sid=00bb3586ac9751ad)  [突然便秘怎么办](https://www.baidu.com/s?word=%E7%AA%81%E7%84%B6%E4%BE%BF%E7%A7%98%E6%80%8E%E4%B9%88%E5%8A%9E&tn=SE_baikepcxf02_fcetbk02&pos=baike_pc_turbo_1767&ori_sid=00bb3586ac9751ad) | [闭合性粉刺怎么排出来](https://www.baidu.com/s?word=%E9%97%AD%E5%90%88%E6%80%A7%E7%B2%89%E5%88%BA%E6%80%8E%E4%B9%88%E6%8E%92%E5%87%BA%E6%9D%A5&tn=SE_baikepcxf02_fcetbk02&pos=baike_pc_turbo_1767&ori_sid=00bb3586ac9751ad) [便秘怎么办快速解决](https://www.baidu.com/s?word=%E4%BE%BF%E7%A7%98%E6%80%8E%E4%B9%88%E5%8A%9E%E5%BF%AB%E9%80%9F%E8%A7%A3%E5%86%B3&tn=SE_baikepcxf02_fcetbk02&pos=baike_pc_turbo_1767&ori_sid=00bb3586ac9751ad) | [乳果糖治疗便秘](https://www.baidu.com/s?word=%E4%B9%B3%E6%9E%9C%E7%B3%96%E6%B2%BB%E7%96%97%E4%BE%BF%E7%A7%98&tn=SE_baikepcxf02_fcetbk02&pos=baike_pc_turbo_1767&ori_sid=00bb3586ac9751ad)  [便秘有什么危害](https://www.baidu.com/s?word=%E4%BE%BF%E7%A7%98%E6%9C%89%E4%BB%80%E4%B9%88%E5%8D%B1%E5%AE%B3&tn=SE_baikepcxf02_fcetbk02&pos=baike_pc_turbo_1767&ori_sid=00bb3586ac9751ad) |
| --- | --- | --- | --- | --- |

Q 新手上路

我有疑问

投诉建议

[成长任务](https://baike.baidu.com/usercenter/tasks#guide) [编辑规则](https://baike.baidu.com/help#main06)

[编辑入门](https://baike.baidu.com/help#main01) [内容质疑](javascript:void(0);)

[本人编辑](https://baike.baidu.com/item/%E7%99%BE%E5%BA%A6%E7%99%BE%E7%A7%91%EF%BC%9A%E6%9C%AC%E4%BA%BA%E8%AF%8D%E6%9D%A1%E7%BC%96%E8%BE%91%E6%9C%8D%E5%8A%A1/22442459?bk_fr=pcFooter) [官方贴吧](http://tieba.baidu.com/f?ie=utf-8&fr=bks0000&kw=%E7%99%BE%E5%BA%A6%E7%99%BE%E7%A7%91)

[在线客服](http://zhiqiu.baidu.com/baike/passport/html/baikechat.html)

[意见反馈](javascript:void(0);)

[举报不良信息](http://help.baidu.com/newadd?word=%E6%85%A2%E6%80%A7%E5%8A%9F%E8%83%BD%E6%80%A7%E4%BE%BF%E7%A7%98&&submit_link=https%3A%2F%2Fbaike.baidu.com%2Fitem%2F%25E6%2585%25A2%25E6%2580%25A7%25E5%258A%259F%25E8%2583%25BD%25E6%2580%25A7%25E4%25BE%25BF%25E7%25A7%2598%3FfromModule%3Dlemma_search-box&prod_id=10&category=1) [投诉侵权信息](http://help.baidu.com/newadd?word=%E6%85%A2%E6%80%A7%E5%8A%9F%E8%83%BD%E6%80%A7%E4%BE%BF%E7%A7%98&&submit_link=https%3A%2F%2Fbaike.baidu.com%2Fitem%2F%25E6%2585%25A2%25E6%2580%25A7%25E5%258A%259F%25E8%2583%25BD%25E6%2580%25A7%25E4%25BE%25BF%25E7%25A7%2598%3FfromModule%3Dlemma_search-box&prod_id=10&category=6)

[未通过词条申诉](http://help.baidu.com/newadd?word=%E6%85%A2%E6%80%A7%E5%8A%9F%E8%83%BD%E6%80%A7%E4%BE%BF%E7%A7%98&&submit_link=https%3A%2F%2Fbaike.baidu.com%2Fitem%2F%25E6%2585%25A2%25E6%2580%25A7%25E5%258A%259F%25E8%2583%25BD%25E6%2580%25A7%25E4%25BE%25BF%25E7%25A7%2598%3FfromModule%3Dlemma_search-box&prod_id=10&category=2)

[封禁查询与解封](http://help.baidu.com/newadd?word=%E6%85%A2%E6%80%A7%E5%8A%9F%E8%83%BD%E6%80%A7%E4%BE%BF%E7%A7%98&&submit_link=https%3A%2F%2Fbaike.baidu.com%2Fitem%2F%25E6%2585%25A2%25E6%2580%25A7%25E5%258A%259F%25E8%2583%25BD%25E6%2580%25A7%25E4%25BE%25BF%25E7%25A7%2598%3FfromModule%3Dlemma_search-box&prod_id=10&category=5)

©2022 Baidu [使用百度前必读](http://www.baidu.com/duty/) | [百科协议](http://help.baidu.com/question?prod_en=baike&class=89&id=1637) | [隐私政策](http://help.baidu.com/question?prod_id=10&class=690&id=1001779) | [百度百科合作平台](https://baike.baidu.com/operation/cooperation) | 京ICP证030173号

[京公网安备11000002000001号](http://www.beian.gov.cn/portal/registerSystemInfo?recordcode=11000002000001)

<https://baike.baidu.com/item/>慢性功能性便秘?fromModule=lemma_search-box

5/5
