# Supplementary material for: Assessment of medical information on irritable bowel syndrome information in Wikipedia and Baidu Encyclopedia: comparative study
Source: PeerJ. 2024 May 24;12:e17264. doi: 10.7717/peerj.17264 (PMC11129691; doi:10.7717/peerj.17264)
Supplement: Data S1 [file peerj-12-17264-s001.zip › σÄƒσoïμò░μì«/Baidu/Baidu-Chinese/10-σèƒΦâ╜μÇoΦà╣μ│╗_τÖ╛σ║aτÖ╛τoæ.docx]

| 2022/12/14 10:50  [网页](https://www.baidu.com/) | [新闻](http://news.baidu.com/) | 功能性腹泻_百度百科  [贴吧](https://tieba.baidu.com/) [知道](https://zhidao.baidu.com/) [网盘](https://pan.baidu.com/?from=1027327l) [图片](http://image.baidu.com/) | [视频](http://v.baidu.com/) | [地图](http://map.baidu.com/) | [文库](https://wenku.baidu.com/) | 百科 | [百度首页](http://www.baidu.com/) [登录](javascript:;) |
| --- | --- | --- | --- | --- | --- | --- | --- |

| [岔](https://baike.baidu.com/) | \| 功能性腹泻 \| 进入词条 \| \| --- \| --- \| | \| 全站搜索 \| \| --- \| | [帮助](https://baike.baidu.com/help) |
| --- | --- | --- | --- | --- | --- | --- |
| 近期有不法分子冒充百度百科官方人员，以删除词条为由威胁并敲诈相关企业。在此严正声明：百度百科是免费编辑平台，绝不存在收费代编服务，请勿上当受骗！ [详情>>](https://baike.baidu.com/common/declaration) | | | |
| [首页](https://baike.baidu.com/) 秒懂百科 特色百科 用户 知识专题 权威合作 [口下载百科APP](https://baike.baidu.com/wapui/subpage/baikeappdownload?sfrom=pc_lemmapage_navigation) [2 个](https://baike.baidu.com/usercenter) | | | |

| 功能性腹泻 | \| [小播报](javascript:;) \| \| --- \| | \| [c编辑](javascript:;) \| \| --- \| | \| [上传视频](javascript:;) \| \| --- \| | . 收藏 [山 59](javascript:void(0);) 24 |  | |
| --- | --- | --- | --- | --- | --- | --- | --- | --- | --- |
|  |  |  |  |  |  | 科普中国  致力于权威的科学传播 |
| 功能性腹泻(functional diarrhea)是指持续地或反复地出现排稀便或水样便，不伴有腹痛或腹部不适症状的综合征。无细 菌、病毒、寄生虫感染的腹泻，一般由胃肠功能失调引起。本病的发生可能与精神因素、肠运动感觉功能异常、自主神经功能紊 乱等相关。经各种检查均未能发现引起腹泻的器质性病变。  外文名 functional diarrhea 常见病因 不明，可能与精神因素、肠运动感觉功能异展…开 、  就诊科室 消化内科 常见症状 不伴有腹痛或腹部不适的少量多次的排便 | | | | | 本词条认证专家为  韩英 丨主任医师   \|  \| \| --- \|   北京军区总医院 消化内科  审核 | |
|  |  |  |  |  |  | |

| \| 目录 \| 1 [病因](#_bookmark1)  2 [临床表现](#_bookmark2)  3 [检查](#_bookmark3)  4 [诊断](#_bookmark4)  5 [治疗](#_bookmark5) \| \| --- \| --- \| | | | 常见病因 | 不明，可能与精神因素、肠运动感觉功能异常、自主 神经功能紊乱等有关  不伴有腹痛或腹部不适的少量多次的排便 | [女疊 口](javascript:void(0);)   \| 词条统计  浏览次数： 147939次  编辑次数： 8次[历史版本](https://baike.baidu.com/historylist/%E5%8A%9F%E8%83%BD%E6%80%A7%E8%85%B9%E6%B3%BB/1309776)  最近更新： [卫计委科普项目](https://baike.baidu.com/usercenter/userpage?uk=x54mmYyOgfn1hjDv9q0UTQ&from=lemma) ( 2017-10-0  3)  突出贡献榜  [andnage](https://baike.baidu.com/usercenter/userpage?uk=sbIszXoccXLdwjYitVh0pw&from=lemma) \| \| --- \| |
| --- | --- | --- | --- | --- | --- | --- | --- | --- |
|  | 基本信息  外文名  就诊科室 | functional diarrhea  消化内科 |  |  |  |
|  |  |  | 常见症状 |  |  |
| 病因 | | |  |  |  |
|  |  |  |  | [小 播报c编辑](javascript:;) |  |
| 有腹泻症状但经各种检查均未发现引起腹泻的器质性病变，称为功能性腹泻。其病因复杂，包括原因未明的肠易激综合征和    原因已明确的继发性小肠乳糖酶缺乏症等。虽病因还不清楚，但研究证明因精神紧张可引起结肠动力的改变，出现腹泻等症状。  临床表现  [小 播报c编辑](javascript:;)  功能性腹泻患者常表现为不伴有腹痛或腹部不适的少量多次的排便，但需要与“假性腹泻”相鉴别， “假性腹泻”表现为排便次数  多、伴排便急迫感，但每次都排成形便。  检查  [小 播报c编辑](javascript:;)  对疑为腹泻患者应该进行血常规、血沉(ESR)及c反应蛋白(CRP)检查。血清铁蛋白与叶酸的测定可帮助与小肠吸收不良相鉴  别。若高度怀疑IBD或小肠吸收不良者，则应进行胃肠道的影像学或胃肠镜检查。对严重水样泻患者需排除内分泌疾病引起的腹  泻，如肠血管活性肽(VIP)瘤和5-HIAA等导致的腹泻。  诊断  [小 播报c编辑](javascript:;)  排除器质性病变，符合以下条件时可以考虑诊断功能性腹泻：①至少75%的排便为不伴有腹痛的稀粪或水样粪；②诊断前症  状出现至少6个月，近3个月符合以上诊断标准。  需要注意的是：腹泻仅是一种非特异性的肠道症状，引起腹泻的病因很多。功能性腹泻须与肠道器质性疾病，如肠道感染性  疾病(慢性细菌性痢疾、肠结核、寄生虫感染性腹泻等)、 IBD、放射性肠炎、结肠肿瘤、小肠吸收不良及VIP瘤等引起的腹泻相  鉴别。消化道内镜、血液生化等相关检查有助于鉴别诊断。  治疗  [小 播报c编辑](javascript:;) | | | | | |

<https://baike.baidu.com/item/>功能性腹泻?fromModule=lemma_search-box

1/2

2022/12/14 10:50

| 岔 搜索发现  [功能性胃病](https://www.baidu.com/s?word=%E5%8A%9F%E8%83%BD%E6%80%A7%E8%83%83%E7%97%85&tn=SE_baikepcxf02_fcetbk02&pos=baike_pc_turbo_1767&ori_sid=00bb359fb29db277)  [治拉肚子的偏方](https://www.baidu.com/s?word=%E6%B2%BB%E6%8B%89%E8%82%9A%E5%AD%90%E7%9A%84%E5%81%8F%E6%96%B9&tn=SE_baikepcxf02_fcetbk02&pos=baike_pc_turbo_1767&ori_sid=00bb359fb29db277) | [腹泻的症状](https://www.baidu.com/s?word=%E8%85%B9%E6%B3%BB%E7%9A%84%E7%97%87%E7%8A%B6&tn=SE_baikepcxf02_fcetbk02&pos=baike_pc_turbo_1767&ori_sid=00bb359fb29db277)  [腹泻的治疗方法](https://www.baidu.com/s?word=%E8%85%B9%E6%B3%BB%E7%9A%84%E6%B2%BB%E7%96%97%E6%96%B9%E6%B3%95&tn=SE_baikepcxf02_fcetbk02&pos=baike_pc_turbo_1767&ori_sid=00bb359fb29db277) | [肠易激综合征会自愈吗](https://www.baidu.com/s?word=%E8%82%A0%E6%98%93%E6%BF%80%E7%BB%BC%E5%90%88%E5%BE%81%E4%BC%9A%E8%87%AA%E6%84%88%E5%90%97&tn=SE_baikepcxf02_fcetbk02&pos=baike_pc_turbo_1767&ori_sid=00bb359fb29db277) [腹泻出血怎么回事](https://www.baidu.com/s?word=%E8%85%B9%E6%B3%BB%E5%87%BA%E8%A1%80%E6%80%8E%E4%B9%88%E5%9B%9E%E4%BA%8B&tn=SE_baikepcxf02_fcetbk02&pos=baike_pc_turbo_1767&ori_sid=00bb359fb29db277) | [拉肚子拉水止泻小妙招](https://www.baidu.com/s?word=%E6%8B%89%E8%82%9A%E5%AD%90%E6%8B%89%E6%B0%B4%E6%AD%A2%E6%B3%BB%E5%B0%8F%E5%A6%99%E6%8B%9B&tn=SE_baikepcxf02_fcetbk02&pos=baike_pc_turbo_1767&ori_sid=00bb359fb29db277) [功能性腹泻怎么治](https://www.baidu.com/s?word=%E5%8A%9F%E8%83%BD%E6%80%A7%E8%85%B9%E6%B3%BB%E6%80%8E%E4%B9%88%E6%B2%BB&tn=SE_baikepcxf02_fcetbk02&pos=baike_pc_turbo_1767&ori_sid=00bb359fb29db277) | [什么是腹泻](https://www.baidu.com/s?word=%E4%BB%80%E4%B9%88%E6%98%AF%E8%85%B9%E6%B3%BB&tn=SE_baikepcxf02_fcetbk02&pos=baike_pc_turbo_1767&ori_sid=00bb359fb29db277)  [腹胀便秘怎么治疗](https://www.baidu.com/s?word=%E8%85%B9%E8%83%80%E4%BE%BF%E7%A7%98%E6%80%8E%E4%B9%88%E6%B2%BB%E7%96%97&tn=SE_baikepcxf02_fcetbk02&pos=baike_pc_turbo_1767&ori_sid=00bb359fb29db277) |
| --- | --- | --- | --- | --- |
|  | | | | |

[口](javascript:void(0);)

功能性腹泻_百度百科

| 对功能性腹泻患者应实施个体化的治疗方案，对由于应激事件引起的腹泻患者，需对其进行详细的病情解释，有助于缓解患  者的焦虑情绪。  **1.**饮食治疗  详细询问患者的饮食习惯。面食、乳制品、柑橘类水果、蛋类、洋葱、咖啡因及酒精等均可能引发功能性腹泻患者症状。因  此，限制这些食物将有助于缓解患者的腹泻症状。  **2.**药物治疗  若改变生活方式无效，可采用药物治疗。常用的抗腹泻药物有洛哌丁胺和苯乙哌啶，此类药物通过与阿片受体结合，阻止胆  碱能神经末梢释放乙酰胆碱，而发挥抗腹泻作用。洛哌丁胺还有非阿片样作用达到止泻目的。 | |
| --- | --- |
| 学术论文 | 内容来自 |
| [李琳，李岩. 肠道菌群失调与功能性腹泻．](https://xueshu.baidu.com/usercenter/paper/show?paperid=620c4c0ed0fb8d935b7f80c469bb44f7&tn=SE_baiduxueshu_c1gjeupa&ie=utf-8&site=baike) 《胃肠病学和肝病学杂志》， 2014  [李红梅，梁浩，唐湖泉，李祥. 匹维溴铵对小鼠功能性腹泻及便秘的治疗作用．](https://xueshu.baidu.com/usercenter/paper/show?paperid=46d6c5256b6e170df77584bf8cb7973a&tn=SE_baiduxueshu_c1gjeupa&ie=utf-8&site=baike) 《世界华人消化杂志》， 1998  [李琳，李岩. 整肠生联合常乐康治疗伴肠道菌群失调功能性腹泻的疗效观察．](https://xueshu.baidu.com/usercenter/paper/show?paperid=c33ca1f2e7997d97344df9f36f247db2&tn=SE_baiduxueshu_c1gjeupa&ie=utf-8&site=baike) 《 CNKI》， 2014  [许继宗，李玉华，张喆，张波. 体感五行音乐疗法联合参苓白术散治疗功能性腹泻疗效观察．](https://xueshu.baidu.com/usercenter/paper/show?paperid=7fdf45e8c158297022072a08332931db&tn=SE_baiduxueshu_c1gjeupa&ie=utf-8&site=baike) 《 CNKI》， 2012  [马秀丽，李正军. 参苓白术散联合理中汤治疗慢性功能性腹泻35例．](https://xueshu.baidu.com/usercenter/paper/show?paperid=9c80887f160c6afc1bd5970093ae7264&tn=SE_baiduxueshu_c1gjeupa&ie=utf-8&site=baike) 《陕西中医》， 2011  [查看全部](https://xueshu.baidu.com/s?wd=intitle%3A%28%E5%8A%9F%E8%83%BD%E6%80%A7%E8%85%B9%E6%B3%BB%29&tn=SE_baiduxueshu_c1gjeupa&ie=utf-8&sc_from=pingtai6&site=baike) | |

| 猜你喜欢 | [腹泻贴多少钱一盒零售，潮流新品，好货热卖，更](http://www.baidu.com/baidu.php?url=Ks00000EAMrnlPLIyW15gPJnEGUHQh0d6RV8AxERo18JrH4TgzWQNa7o-Mo8nKInb4TnBiU0XRBadvSqfgxzTGduEewgRz917ijYbinXTwsqJjr1d_a_S8ckmupJriveDbdws1fdMMsxBIaFjGRvjnAdARf00iWPMBPrtL2WRHzmreq-KBafsauqN7sGOFEIp2KF9DvYzaQ5qd0iqiDDf2TB8XMb.Db_iHF8xnhA94wEYL_SNK-deQbfHgI3ynDgg6msw5I7AMHdey5Z_otIv8EWj3q-Xek8dqTUAMHz4rMG34nheuztIdMugbzTEZF83e5ZGzIUvZO-OtZeOL3Xho1Ce5ZHOkxWqqgYFlk_IIU7Na9WWstxU9zxgjbS8aB17I4RAgg_8w9zxyPrMjbSVaSPQnYPyZWqubltXQjkSyMHz4rMG34nheuztIdMugbzTEZF83e5ZGzmTMHvGYTjGo_5Z4mThe1vmTheu8s4PLoEs4PLMY3Thed33ThedqTrHI4enrHIlqhZF8qX1jbLXXejbLXL_s4PvOv3The1LmThed_s4PvSEjbLXXOjbLUthZF8qptrHI4qhZF8qIhZF8vymThe1tTrHIEqTrHIEzmThe1L3Thexl3ThedlTrHI4e_rHIEenrHIexPHReiM-kl-9h9mzyUO7f0.U1Yk0ZDq8pUDLPjR8QMf86KY5UB4YQonSUU6zIMZoV2e8VjK3toU0A-V5HDzPWc0Iybq0ZKGujYzn0KWpyfqP1c0mhbqn10k0AuY5H00TA6qn0KET1Ys0AFL5H00UMfqn0K1XWY0ThIYmyTqn0K8IM0qna3snj0snj0sn0K-ThTqn0KYTh7buHYs0AFbpyfqnW77fbc4nYu7wWFDnRwArj97fYfYrD7DrRwKPDnvnbD0uAPWujY0mgPxpywW5gK1QyIlpZ940ATqILP8TsKzIjY1rHf0TgKGujYs0Z7Wpyfqn0K9mWYsg100TZ0qn0KVIZ0qn0KbuAqs5H00ThCqn0KYIgnqnHTdPWm4nH0YPWnvnjTLPjRzPjc0mynqnfKsUWYs0ZK9I7qhUA7M5H00ugPY5H00ugwGujYVnfK9TLKWm1Ys0ZNspy4Wm1Ys0AuWIgfqn0K9uAu_myTqnfKLuMFEUHY0mMfqnfKzug7Y5HDvP104rjmkrHbzPHm0Tv-b5H0smhc1PyDsmWFWPAN9njc0ULfqn0KETMKY5H0WnaPDw-fWnansc10Wna3sc10WwDuRc10WwDuR0AVG5H00UgfqnW0vn6KVm1YzPWTYnW64nH6vn0KVmdqhThqV5H00uA78IyF-gLK_my4GuZnqn0K9uZ745UB4YQonSfK9uZ7Y5H00pgPWUjYs0Z7VIjYs0A7bgLPEIgFWuHYznzPkIyNzXNqkIyNzXNqsmzPxgdqxTAP8TzPkIyNzXiP-TvdVT-qvnfKWThnqPH0knj6&us=newvui&ai=0_429107157_1_1&word=&ck=0.0.0.0.0.0.0.0&shh=baike.baidu.com)…  [腹泻贴多少钱一盒零售，购物上淘宝，优选材质，用的舒心!在线](http://www.baidu.com/baidu.php?url=Ks00000EAMrnlPLIyW15gPJnEGUHQh0d6RV8AxERo18JrH4TgzWQNa7o-Mo8nKInb4TnBiU0XRBadvSqfgxzTGduEewgRz917ijYbinXTwsqJjr1d_a_S8ckmupJriveDbdws1fdMMsxBIaFjGRvjnAdARf00iWPMBPrtL2WRHzmreq-KBafsauqN7sGOFEIp2KF9DvYzaQ5qd0iqiDDf2TB8XMb.Db_iHF8xnhA94wEYL_SNK-deQbfHgI3ynDgg6msw5I7AMHdey5Z_otIv8EWj3q-Xek8dqTUAMHz4rMG34nheuztIdMugbzTEZF83e5ZGzIUvZO-OtZeOL3Xho1Ce5ZHOkxWqqgYFlk_IIU7Na9WWstxU9zxgjbS8aB17I4RAgg_8w9zxyPrMjbSVaSPQnYPyZWqubltXQjkSyMHz4rMG34nheuztIdMugbzTEZF83e5ZGzmTMHvGYTjGo_5Z4mThe1vmTheu8s4PLoEs4PLMY3Thed33ThedqTrHI4enrHIlqhZF8qX1jbLXXejbLXL_s4PvOv3The1LmThed_s4PvSEjbLXXOjbLUthZF8qptrHI4qhZF8qIhZF8vymThe1tTrHIEqTrHIEzmThe1L3Thexl3ThedlTrHI4e_rHIEenrHIexPHReiM-kl-9h9mzyUO7f0.U1Yk0ZDq8pUDLPjR8QMf86KY5UB4YQonSUU6zIMZoV2e8VjK3toU0A-V5HDzPWc0Iybq0ZKGujYzn0KWpyfqP1c0mhbqn10k0AuY5H00TA6qn0KET1Ys0AFL5H00UMfqn0K1XWY0ThIYmyTqn0K8IM0qna3snj0snj0sn0K-ThTqn0KYTh7buHYs0AFbpyfqnW77fbc4nYu7wWFDnRwArj97fYfYrD7DrRwKPDnvnbD0uAPWujY0mgPxpywW5gK1QyIlpZ940ATqILP8TsKzIjY1rHf0TgKGujYs0Z7Wpyfqn0K9mWYsg100TZ0qn0KVIZ0qn0KbuAqs5H00ThCqn0KYIgnqnHTdPWm4nH0YPWnvnjTLPjRzPjc0mynqnfKsUWYs0ZK9I7qhUA7M5H00ugPY5H00ugwGujYVnfK9TLKWm1Ys0ZNspy4Wm1Ys0AuWIgfqn0K9uAu_myTqnfKLuMFEUHY0mMfqnfKzug7Y5HDvP104rjmkrHbzPHm0Tv-b5H0smhc1PyDsmWFWPAN9njc0ULfqn0KETMKY5H0WnaPDw-fWnansc10Wna3sc10WwDuRc10WwDuR0AVG5H00UgfqnW0vn6KVm1YzPWTYnW64nH6vn0KVmdqhThqV5H00uA78IyF-gLK_my4GuZnqn0K9uZ745UB4YQonSfK9uZ7Y5H00pgPWUjYs0Z7VIjYs0A7bgLPEIgFWuHYznzPkIyNzXNqkIyNzXNqsmzPxgdqxTAP8TzPkIyNzXiP-TvdVT-qvnfKWThnqPH0knj6&us=newvui&ai=0_429107157_1_1&word=&ck=0.0.0.0.0.0.0.0&shh=baike.baidu.com)  [下单，省时省力。你要的好货尽在淘宝网，安心享受网购乐趣!](http://www.baidu.com/baidu.php?url=Ks00000EAMrnlPLIyW15gPJnEGUHQh0d6RV8AxERo18JrH4TgzWQNa7o-Mo8nKInb4TnBiU0XRBadvSqfgxzTGduEewgRz917ijYbinXTwsqJjr1d_a_S8ckmupJriveDbdws1fdMMsxBIaFjGRvjnAdARf00iWPMBPrtL2WRHzmreq-KBafsauqN7sGOFEIp2KF9DvYzaQ5qd0iqiDDf2TB8XMb.Db_iHF8xnhA94wEYL_SNK-deQbfHgI3ynDgg6msw5I7AMHdey5Z_otIv8EWj3q-Xek8dqTUAMHz4rMG34nheuztIdMugbzTEZF83e5ZGzIUvZO-OtZeOL3Xho1Ce5ZHOkxWqqgYFlk_IIU7Na9WWstxU9zxgjbS8aB17I4RAgg_8w9zxyPrMjbSVaSPQnYPyZWqubltXQjkSyMHz4rMG34nheuztIdMugbzTEZF83e5ZGzmTMHvGYTjGo_5Z4mThe1vmTheu8s4PLoEs4PLMY3Thed33ThedqTrHI4enrHIlqhZF8qX1jbLXXejbLXL_s4PvOv3The1LmThed_s4PvSEjbLXXOjbLUthZF8qptrHI4qhZF8qIhZF8vymThe1tTrHIEqTrHIEzmThe1L3Thexl3ThedlTrHI4e_rHIEenrHIexPHReiM-kl-9h9mzyUO7f0.U1Yk0ZDq8pUDLPjR8QMf86KY5UB4YQonSUU6zIMZoV2e8VjK3toU0A-V5HDzPWc0Iybq0ZKGujYzn0KWpyfqP1c0mhbqn10k0AuY5H00TA6qn0KET1Ys0AFL5H00UMfqn0K1XWY0ThIYmyTqn0K8IM0qna3snj0snj0sn0K-ThTqn0KYTh7buHYs0AFbpyfqnW77fbc4nYu7wWFDnRwArj97fYfYrD7DrRwKPDnvnbD0uAPWujY0mgPxpywW5gK1QyIlpZ940ATqILP8TsKzIjY1rHf0TgKGujYs0Z7Wpyfqn0K9mWYsg100TZ0qn0KVIZ0qn0KbuAqs5H00ThCqn0KYIgnqnHTdPWm4nH0YPWnvnjTLPjRzPjc0mynqnfKsUWYs0ZK9I7qhUA7M5H00ugPY5H00ugwGujYVnfK9TLKWm1Ys0ZNspy4Wm1Ys0AuWIgfqn0K9uAu_myTqnfKLuMFEUHY0mMfqnfKzug7Y5HDvP104rjmkrHbzPHm0Tv-b5H0smhc1PyDsmWFWPAN9njc0ULfqn0KETMKY5H0WnaPDw-fWnansc10Wna3sc10WwDuRc10WwDuR0AVG5H00UgfqnW0vn6KVm1YzPWTYnW64nH6vn0KVmdqhThqV5H00uA78IyF-gLK_my4GuZnqn0K9uZ745UB4YQonSfK9uZ7Y5H00pgPWUjYs0Z7VIjYs0A7bgLPEIgFWuHYznzPkIyNzXNqkIyNzXNqsmzPxgdqxTAP8TzPkIyNzXiP-TvdVT-qvnfKWThnqPH0knj6&us=newvui&ai=0_429107157_1_1&word=&ck=0.0.0.0.0.0.0.0&shh=baike.baidu.com)  [simba.taobao.com](http://www.baidu.com/baidu.php?url=Ks00000EAMrnlPLIyW15gPJnEGUHQh0d6RV8AxERo18JrH4TgzWQNa7o-Mo8nKInb4TnBiU0XRBadvSqfgxzTGduEewgRz917ijYbinXTwsqJjr1d_a_S8ckmupJriveDbdws1fdMMsxBIaFjGRvjnAdARf00iWPMBPrtL2WRHzmreq-KBafsauqN7sGOFEIp2KF9DvYzaQ5qd0iqiDDf2TB8XMb.Db_iHF8xnhA94wEYL_SNK-deQbfHgI3ynDgg6msw5I7AMHdey5Z_otIv8EWj3q-Xek8dqTUAMHz4rMG34nheuztIdMugbzTEZF83e5ZGzIUvZO-OtZeOL3Xho1Ce5ZHOkxWqqgYFlk_IIU7Na9WWstxU9zxgjbS8aB17I4RAgg_8w9zxyPrMjbSVaSPQnYPyZWqubltXQjkSyMHz4rMG34nheuztIdMugbzTEZF83e5ZGzmTMHvGYTjGo_5Z4mThe1vmTheu8s4PLoEs4PLMY3Thed33ThedqTrHI4enrHIlqhZF8qX1jbLXXejbLXL_s4PvOv3The1LmThed_s4PvSEjbLXXOjbLUthZF8qptrHI4qhZF8qIhZF8vymThe1tTrHIEqTrHIEzmThe1L3Thexl3ThedlTrHI4e_rHIEenrHIexPHReiM-kl-9h9mzyUO7f0.U1Yk0ZDq8pUDLPjR8QMf86KY5UB4YQonSUU6zIMZoV2e8VjK3toU0A-V5HDzPWc0Iybq0ZKGujYzn0KWpyfqP1c0mhbqn10k0AuY5H00TA6qn0KET1Ys0AFL5H00UMfqn0K1XWY0ThIYmyTqn0K8IM0qna3snj0snj0sn0K-ThTqn0KYTh7buHYs0AFbpyfqnW77fbc4nYu7wWFDnRwArj97fYfYrD7DrRwKPDnvnbD0uAPWujY0mgPxpywW5gK1QyIlpZ940ATqILP8TsKzIjY1rHf0TgKGujYs0Z7Wpyfqn0K9mWYsg100TZ0qn0KVIZ0qn0KbuAqs5H00ThCqn0KYIgnqnHTdPWm4nH0YPWnvnjTLPjRzPjc0mynqnfKsUWYs0ZK9I7qhUA7M5H00ugPY5H00ugwGujYVnfK9TLKWm1Ys0ZNspy4Wm1Ys0AuWIgfqn0K9uAu_myTqnfKLuMFEUHY0mMfqnfKzug7Y5HDvP104rjmkrHbzPHm0Tv-b5H0smhc1PyDsmWFWPAN9njc0ULfqn0KETMKY5H0WnaPDw-fWnansc10Wna3sc10WwDuRc10WwDuR0AVG5H00UgfqnW0vn6KVm1YzPWTYnW64nH6vn0KVmdqhThqV5H00uA78IyF-gLK_my4GuZnqn0K9uZ745UB4YQonSfK9uZ7Y5H00pgPWUjYs0Z7VIjYs0A7bgLPEIgFWuHYznzPkIyNzXNqkIyNzXNqsmzPxgdqxTAP8TzPkIyNzXiP-TvdVT-qvnfKWThnqPH0knj6&us=newvui&ai=0_429107157_1_1&word=&ck=0.0.0.0.0.0.0.0&shh=baike.baidu.com) |
| --- | --- |

[女](javascript:void(0);)

Q 新手上路 我有疑问 投诉建议

[成长任务](https://baike.baidu.com/usercenter/tasks#guide) [编辑规则](https://baike.baidu.com/help#main06)

[编辑入门](https://baike.baidu.com/help#main01) [内容质疑](javascript:void(0);)

[本人编辑](https://baike.baidu.com/item/%E7%99%BE%E5%BA%A6%E7%99%BE%E7%A7%91%EF%BC%9A%E6%9C%AC%E4%BA%BA%E8%AF%8D%E6%9D%A1%E7%BC%96%E8%BE%91%E6%9C%8D%E5%8A%A1/22442459?bk_fr=pcFooter) [官方贴吧](http://tieba.baidu.com/f?ie=utf-8&fr=bks0000&kw=%E7%99%BE%E5%BA%A6%E7%99%BE%E7%A7%91)

[在线客服](http://zhiqiu.baidu.com/baike/passport/html/baikechat.html)

[意见反馈](javascript:void(0);)

[举报不良信息](http://help.baidu.com/newadd?word=%E5%8A%9F%E8%83%BD%E6%80%A7%E8%85%B9%E6%B3%BB&&submit_link=https%3A%2F%2Fbaike.baidu.com%2Fitem%2F%25E5%258A%259F%25E8%2583%25BD%25E6%2580%25A7%25E8%2585%25B9%25E6%25B3%25BB%3FfromModule%3Dlemma_search-box&prod_id=10&category=1) [投诉侵权信息](http://help.baidu.com/newadd?word=%E5%8A%9F%E8%83%BD%E6%80%A7%E8%85%B9%E6%B3%BB&&submit_link=https%3A%2F%2Fbaike.baidu.com%2Fitem%2F%25E5%258A%259F%25E8%2583%25BD%25E6%2580%25A7%25E8%2585%25B9%25E6%25B3%25BB%3FfromModule%3Dlemma_search-box&prod_id=10&category=6)

[未通过词条申诉](http://help.baidu.com/newadd?word=%E5%8A%9F%E8%83%BD%E6%80%A7%E8%85%B9%E6%B3%BB&&submit_link=https%3A%2F%2Fbaike.baidu.com%2Fitem%2F%25E5%258A%259F%25E8%2583%25BD%25E6%2580%25A7%25E8%2585%25B9%25E6%25B3%25BB%3FfromModule%3Dlemma_search-box&prod_id=10&category=2)

[封禁查询与解封](http://help.baidu.com/newadd?word=%E5%8A%9F%E8%83%BD%E6%80%A7%E8%85%B9%E6%B3%BB&&submit_link=https%3A%2F%2Fbaike.baidu.com%2Fitem%2F%25E5%258A%259F%25E8%2583%25BD%25E6%2580%25A7%25E8%2585%25B9%25E6%25B3%25BB%3FfromModule%3Dlemma_search-box&prod_id=10&category=5)

©2022 Baidu [使用百度前必读](http://www.baidu.com/duty/) | [百科协议](http://help.baidu.com/question?prod_en=baike&class=89&id=1637) | [隐私政策](http://help.baidu.com/question?prod_id=10&class=690&id=1001779) | [百度百科合作平台](https://baike.baidu.com/operation/cooperation) | 京ICP证030173号

[京公网安备11000002000001号](http://www.beian.gov.cn/portal/registerSystemInfo?recordcode=11000002000001)

<https://baike.baidu.com/item/>功能性腹泻?fromModule=lemma_search-box

2/2
