# Supplementary material for: Assessment of medical information on irritable bowel syndrome information in Wikipedia and Baidu Encyclopedia: comparative study
Source: PeerJ. 2024 May 24;12:e17264. doi: 10.7717/peerj.17264 (PMC11129691; doi:10.7717/peerj.17264)
Supplement: Data S1 [file peerj-12-17264-s001.zip › σÄƒσoïμò░μì«/Baidu/Baidu-Chinese/1-IBS∩╝êΦéáμÿôμ┐Çτ╗╝σÉêσ╛ü∩╝ë_τÖ╛σ║aτÖ╛τoæ.docx]

2022/12/14 10:29

[网页](https://www.baidu.com/) [新闻](http://news.baidu.com/) [贴吧](https://tieba.baidu.com/) [知道](https://zhidao.baidu.com/) [网盘](https://pan.baidu.com/?from=1027327l) [图片](http://image.baidu.com/) [视频](http://v.baidu.com/) [地图](http://map.baidu.com/) [文库](https://wenku.baidu.com/) 百科 [百度首页](http://www.baidu.com/) [登录](javascript:;)

[小播报](javascript:;)

[c编辑](javascript:;)

[O讨论](https://baike.baidu.com/planet/talk?lemmaId=10530572&fromModule=lemma_right-issue-btn)

IBS

| IBS | 进入词条 |
| --- | --- |

[岔](https://baike.baidu.com/)

小 播报

[夕 编辑](javascript:;)

[回 讨论](https://baike.baidu.com/planet/talk?lemmaId=10530572)

[让 收藏](javascript:;)

[凸 赞](javascript:;)

IBS (肠易激综合征) _百度百科

[岔](https://baike.baidu.com/) 进入词条 [帮助](https://baike.baidu.com/help)

近期有不法分子冒充百度百科官方人员，以删除词条为由威胁并敲诈相关企业。在此严正声明：百度百科是免费编辑平台，绝不存在收费代编服务，请勿上当受骗！ [详情>>](https://baike.baidu.com/common/declaration)

[首页](https://baike.baidu.com/)

秒懂百科

特色百科

用户

知识专题

权威合作

[口下载百科APP](https://baike.baidu.com/wapui/subpage/baikeappdownload?sfrom=pc_lemmapage_navigation) [2 个](https://baike.baidu.com/usercenter)

**IBS**是一个[多义词](https://baike.baidu.com/item/%E7%99%BE%E5%BA%A6%E7%99%BE%E7%A7%91%EF%BC%9A%E5%A4%9A%E4%B9%89%E8%AF%8D)，请在下列[义项](https://baike.baidu.com/item/%E4%B9%89%E9%A1%B9)上选择浏览([共6个义项](https://baike.baidu.com/item/IBS?force=1)) [展开 、添加义项 +](javascript:;)

| [训上传视频](javascript:;) |
| --- |

肠易激综合征

什么是肠易激综合征？

02:22

| . 收藏 [山 178](javascript:void(0);) 吐 108  IBS即[肠易激综合征](https://baike.baidu.com/item/%E8%82%A0%E6%98%93%E6%BF%80%E7%BB%BC%E5%90%88%E5%BE%81/8456761?fromModule=lemma_inlink)(Irritable Bowel Syndrome， IBS)是一种常见的功能性肠病，以腹痛或腹部不适为主要症状，排便后可改 善，常伴有排便习惯改变，缺乏解释症状的形态学和生化学异常。  世界各地流行病学研究报道显示IBS是一种世界范围内的多发病。西方国家人群患病率达10%一20%，我国的一项按罗马Ⅱ 标准的流行病学调查显示社区人群IBS患病率为5.7%，其中22%曾因IBS症状而就诊。  肠易激综合症  irritable bowel syndrome,IBS  西医学名  外文名  常见症状  英文简称  腹痛，腹胀，腹泻，便秘  IBS   \| 目录 \| 1 [病因](#_bookmark1)  2 [诊断](#_bookmark2)  3 [肠易激综合征](#_bookmark3)  4 [鉴别诊断](#_bookmark4)  5 [治疗](#_bookmark5) \| \| --- \| --- \|   口  病因  [小 播报c编辑](javascript:;)  一般的病因是机体应激反应与心理因素相互作用的结果，不同的个体都可能涉及遗传、环境、心理、社会和胃肠感染等因 素，导致胃肠动力改变、脑—肠轴相互作用的紊乱、自主神经和激素的变化等，伴有精神障碍(如恐慌、焦虑、创伤后应激紊乱 等)、 [睡眠障碍](https://baike.baidu.com/item/%E7%9D%A1%E7%9C%A0%E9%9A%9C%E7%A2%8D/767228?fromModule=lemma_inlink)和心理应对障碍的患者，应激性生活事件常可导致症状的加重，但对心理因素与IBS之间的确切联系还不十分清 楚。研究发现有1/3的IBS患者有胃肠道感染史，国内外都强调精神心理因素对IBS发病的影响，更加重视神经肽类和相关受体功 能在IBS发病机制中的作用；近些年人们加强了IBS与[炎症性肠病](https://baike.baidu.com/item/%E7%82%8E%E7%97%87%E6%80%A7%E8%82%A0%E7%97%85/3521532?fromModule=lemma_inlink) ( IBD)之间的联系研究，少数学者甚至认为IBS为IBD的前期表 现。  诊断  [小 播报c编辑](javascript:;)  依旧以症状学作为[临床诊断](https://baike.baidu.com/item/%E4%B8%B4%E5%BA%8A%E8%AF%8A%E6%96%AD/9401523?fromModule=lemma_inlink)和研究。  肠易激综合征  [小 播报c编辑](javascript:;)     \| [[女](javascript:void(0);)](https://baike.baidu.com/pic/IBS/10530572/1/91ef76c6a7efce1b766d655ea551f3deb58f65e4?fr=lemma&fromModule=lemma_top-image&ct=single)  IBS的概述图(2张)[疊](javascript:void(0);) \| \| --- \| \|  \| \| 词条统计 \| \| 浏览次数： 871420次  编辑次数： 51次[历史版本](https://baike.baidu.com/historylist/IBS/10530572)  最近更新： [w_ou](https://baike.baidu.com/usercenter/userpage?uk=TY3CXj_hJSEcBfJBVP43Rg&from=lemma) ( 2021-01-26)  突出贡献榜  [shmily32123](https://baike.baidu.com/usercenter/userpage?uk=5Iw5RYbIVJ8rf4-PMPKeGQ&from=lemma) \|  \| **1** aci营养师 **12** 自己创建个  **2** sci文献 **13** 手机空号  **3** 营养师报考资 **14** 大专生怎么  **4** 二级心理咨询 **15** 意大利留学  **5** 电商怎么做 **16** 征文投稿  **6** 快速学日语 **17** 如何治脸瘫  **7** 战队logo设计 **18** 9733游戏平  **8** 哈佛大学申请 **19** 芝加哥留学  **9** 哈佛大学入学 **20** 怎么成为插  **10** 价格便宜的香 **21** 华师在职研  **11** 千锋教育 **22** 哈佛大学宿 \| \| --- \|   1) IBS的一般标准 在最近的3个月内，每个月至少有3天出现反复发作的腹痛或不适症状，并具有下列中的2项或2项以上：  ①排便后症状改善。  ②伴随排便频率的改变。  ③伴随粪便性状的改变。 |
| --- | --- | --- | --- | --- | --- | --- | --- |

<https://baike.baidu.com/item/IBS/10530572?fromModule=lemma_search-box>

1/4

2022/12/14 10:29

IBS (肠易激综合征) _百度百科

| 诊断标准建立于患者至少在诊断前的6个月内出现症状，并在最近的3个月持续存在，在观察期间疼痛(不适)症状的频率至  [岔](https://baike.baidu.com/)  小 播报  [编辑](javascript:;)  [回 讨论](https://baike.baidu.com/planet/talk?lemmaId=10530572)  [收藏](javascript:;)  [赞](javascript:;)  少一周2天。 |
| --- |
| 2) IBS的一般下列症状可支持诊断：  ①异常的排便频率： a.每周≤3次排便或b.每天>3次排便。  ②异常的粪便性状： c.块状便/硬便或d.松散便/稀水便。  ③排便费力。  ④排便急迫感或[排便不尽感](https://baike.baidu.com/item/%E6%8E%92%E4%BE%BF%E4%B8%8D%E5%B0%BD%E6%84%9F/15786777?fromModule=lemma_inlink)。  ⑤排出黏液。  ⑥腹胀。  3) IBS亚型 依据粪便的性状分为以下亚型：  ①IBS便秘型(IBS-C)：硬便或块状便排便比例≥25%，稀便(糊状便)或水样便排便比例<25%。  ②IBS腹泻型(IBS－D)：稀便(糊状便)或水样便排便比例≥25%，硬便或块状便排便比例<25%。  ③混合型IBS(IBS-M)：硬便或块状便排便比例>25%，稀便(糊状便)或水样便排便比例≥25%。  ④不确定型IBS ( IBS-U)：粪便的性状不符合上述IBS-C， D， M之中的任一标准。  在IBS分型时除需注重粪便性状外，还应注意到患者的排便费力、急迫感和排便不尽感等症状，在多数情况下粪便性状(从  稀水样泻到硬结便)能够反映肠管的转运时间。  既往强调诊断的要点是：排便后腹痛减轻，腹痛时伴大便次数[增多](https://baike.baidu.com/item/%E5%A2%9E%E5%A4%9A?fromModule=lemma_inlink)；腹痛发作时大便变稀，明显腹胀。现依旧在使用，但在  鉴别器质性肠病上价值有限，需要适时作恰当的检查。确定IBS的诊断通常需要进行详细的采集病史和体格检查，根据不同患者  的需求，有针对性地[进行](https://baike.baidu.com/item/%E8%BF%9B%E8%A1%8C?fromModule=lemma_inlink)实验室和辅助检查。以便尽可能以[器质性疾病](https://baike.baidu.com/item/%E5%99%A8%E8%B4%A8%E6%80%A7%E7%96%BE%E7%97%85/1575755?fromModule=lemma_inlink)本身解释所有的症状，轻的器质病也可以与IBS同在。 |
| 鉴别诊断  [小 播报编辑](javascript:;)  [女疊 口](javascript:void(0);)    需要与IBS鉴别诊断的疾病主要有[炎症性肠病](https://baike.baidu.com/item/%E7%82%8E%E7%97%87%E6%80%A7%E8%82%A0%E7%97%85/3521532?fromModule=lemma_inlink)、结直肠肿瘤，还要注意IBS· D与[乳糖不耐受](https://baike.baidu.com/item/%E4%B9%B3%E7%B3%96%E4%B8%8D%E8%80%90%E5%8F%97/2789926?fromModule=lemma_inlink)、[小肠细菌过度生长](https://baike.baidu.com/item/%E5%B0%8F%E8%82%A0%E7%BB%86%E8%8F%8C%E8%BF%87%E5%BA%A6%E7%94%9F%E9%95%BF/637336?fromModule=lemma_inlink)、寄生虫感染  等鉴别。  [小 播报编辑](javascript:;)  治疗  在此强调个体化的综合治疗，即应包括精神心理行为干预治疗、饮食调整和药物治疗，患者的治疗方法和对药物的选择应因  人而异对症处理。制定罗马Ⅲ中IBS专家组对治疗的推荐意见有三类：  1、心理治疗  症状严重而顽固，经一般治疗和药物治疗无效者应考虑予心理行为治疗。包括心理治疗、认知治疗、催眠疗法、生物反馈  等。  2、饮食调整  不良的饮食习惯和膳食结构可以加剧IBS的症状。因此，健康、平衡的饮食可有助于减轻患者的[胃肠功能紊乱](https://baike.baidu.com/item/%E8%83%83%E8%82%A0%E5%8A%9F%E8%83%BD%E7%B4%8A%E4%B9%B1/11043122?fromModule=lemma_inlink)症状。 IBS患者  宜避免：  ①过度饮食；  ②大量饮酒；  ③咖啡因；  ④高脂饮食；  ⑤某些具有“产气”作用的蔬菜类等；  ⑥精加工食粮和人工食品(便秘者)，山梨醇及果糖(腹泻者)；  ⑦不耐受的食物(因不同个体而异)。  增加膳食纤维主要用于便秘为主的IBS患者，增加纤维摄入量的方法应个体化。  3、药物治疗  主要针对症状显著的患者，由于IBS患者症状复杂多变且与中枢和肠神经系统间复杂的关系，药物都存在有效性和安全性的  限制，只能一定程度起作用。得舒特为代表胃肠选择性[钙离子拮抗剂](https://baike.baidu.com/item/%E9%92%99%E7%A6%BB%E5%AD%90%E6%8B%AE%E6%8A%97%E5%89%82/1100351?fromModule=lemma_inlink)在各类平滑肌解痉剂中最欢迎，在数十个国家使用；替加色  罗(随罗马Ⅲ标准一同推出)曾风光无限，其有效性和安全性的限制了它的使用；近几年对益生菌治疗很重视，具有调节内脏敏 |

<https://baike.baidu.com/item/IBS/10530572?fromModule=lemma_search-box>

2/4

2022/12/14 10:29

IBS (肠易激综合征) _百度百科

| [岔](https://baike.baidu.com/)  感性的药物(作用于各级神经调节的内脏敏感性)为研制的重点，现有的低剂量三环类和5羟色胺再摄取抑制剂治疗抗抑郁药有 一定的调节内脏敏感性，其长期治疗仍被强调，尤其有较顽固症状者。  小 播报  [编辑](javascript:;)  [回 讨论](https://baike.baidu.com/planet/talk?lemmaId=10530572)  [收藏](javascript:;)  [赞](javascript:;) |
| --- |
| 其它非药物治疗有心理专科治疗、催眠治疗、生物反馈治疗、行为治疗等，这些现虽未被罗马Ⅲ中IBS专家组对治疗的推  [女疊 口](javascript:void(0);)  荐，但与上述有交叉，也显出一定的疗效，需要进一步研究。  [肠易激综合征](https://baike.baidu.com/item/%E8%82%A0%E6%98%93%E6%BF%80%E7%BB%BC%E5%90%88%E5%BE%81/8456761?fromModule=lemma_inlink) ( IBS)是一种常见的[功能性胃肠病](https://baike.baidu.com/item/%E5%8A%9F%E8%83%BD%E6%80%A7%E8%83%83%E8%82%A0%E7%97%85/7388928?fromModule=lemma_inlink)，以腹痛或腹部不适为主要症状，排便后可改善，常伴排便习惯及大便性  状改变，便秘和腹泻可交替出现，但缺乏可解释症状的形态学和生化异常。过去称为“[痉挛性结肠炎](https://baike.baidu.com/item/%E7%97%89%E6%8C%9B%E6%80%A7%E7%BB%93%E8%82%A0%E7%82%8E/2423303?fromModule=lemma_inlink)”“黏液性结肠炎”及“不稳定性  结肠炎”等。该病患者以20-40岁中青年居多，女性多见；具有反复发作的倾向，常迁延难愈。在药物治疗方面，西医暂无理想的  药物。  [肠易激综合征](https://baike.baidu.com/item/%E8%82%A0%E6%98%93%E6%BF%80%E7%BB%BC%E5%90%88%E5%BE%81/8456761?fromModule=lemma_inlink)在中国的发病率在10%-20%左右，患者以中青年为主。确切的致病机制仍不明确，但精神心理因素是导致发病  的重要因素，严重影响患者生活质量。患者应及时就医，可在医生指导下选用解痉剂缓解相应症状；以及止泻剂/导泻剂对应改善  腹泻/便秘的症状；或通过益生菌调整肠道有益菌群，减轻腹部不适症状。调整饮食结构，改善情绪状态，才能实现逐渐康复的目  的。 [1]  [词条图册 更多图册 >](https://baike.baidu.com/pic/IBS/10530572?fr=lemma)   \|  \| \| \| --- \| --- \| \| 概述图册(2) \| \| \|  \|  \|  \| **NRN** \| \| \| \| \| \| \| \| \| \| \| \| --- \| --- \| --- \| --- \| --- \| --- \| --- \| --- \| --- \| --- \| --- \| \|  \| ▪ \| [STV](http://baike.baidu.com/view/444125.htm) \| ▪ \| [HBC](http://baike.baidu.com/searchword/?word=HBC&pic=1&sug=1&enc=utf8) \| ▪ \| [RAB](http://baike.baidu.com/view/1082772.htm) \| ▪ [IBC](http://baike.baidu.com/view/1518618.htm) \| ▪ \| [TBC](http://baike.baidu.com/view/713117.htm) \| ▪ [ABS](http://baike.baidu.com/view/8910.htm) \| \|  \| ▪ \| [YBC](http://baike.baidu.com/view/609803.htm) \| ▪ \| [RFC](http://baike.baidu.com/view/6108.htm) \| ▪ \| [QR](http://baike.baidu.com/view/124747.htm) \| ▪ [LF](http://baike.baidu.com/view/3446937.htm) \| ▪ \| [IBS](http://baike.baidu.com/view/601942.htm) \| ▪ [CRT](http://baike.baidu.com/view/1190.htm) \| \|  \| ▪ \| [YBS](http://baike.baidu.com/searchword/?word=YBS&pic=1&sug=1&enc=utf8) \| ▪ \| [SBC](http://baike.baidu.com/view/259912.htm) \| ▪ \| [BSN](http://baike.baidu.com/view/805622.htm) \| ▪ [SBS](http://baike.baidu.com/view/306571.htm) \| ▪ \| [SF](http://baike.baidu.com/view/22900.htm) \| ▪ [KNB](http://baike.baidu.com/searchword/?word=KNB&pic=1&sug=1&enc=utf8) \| \| 加盟电台 \| ▪  ▪  ▪  ▪ \| [MRO](http://baike.baidu.com/view/981647.htm)  [BSS](http://baike.baidu.com/view/453125.htm)  [RNB](http://baike.baidu.com/view/1031667.htm)  [MBC](http://baike.baidu.com/view/335494.htm) \| ▪  ▪  ▪  ▪ \| [FBC](http://baike.baidu.com/view/1653990.htm)  [RSK](http://baike.baidu.com/searchword/?word=RSK&pic=1&sug=1&enc=utf8)  [RKC](http://baike.baidu.com/searchword/?word=RKC&pic=1&sug=1&enc=utf8)  [ROK](http://baike.baidu.com/searchword/?word=ROK&pic=1&sug=1&enc=utf8) \| ▪  ▪  ▪ \| [MBS](http://baike.baidu.com/view/254906.htm)  [RCC](http://baike.baidu.com/view/1193496.htm)  [KBC](http://baike.baidu.com/view/818218.htm) \| ▪ [ABC](http://baike.baidu.com/view/5544.htm)  ▪ [KRY](http://baike.baidu.com/view/1581361.htm)  ▪ [NBC](http://baike.baidu.com/view/495074.htm) \| ▪  ▪  ▪ \| [OBC](http://baike.baidu.com/view/1443013.htm)  [JRT](http://baike.baidu.com/searchword/?word=JRT&pic=1&sug=1&enc=utf8)  [RKK](http://baike.baidu.com/searchword/?word=RKK&pic=1&sug=1&enc=utf8) \| ▪ [KBS](http://baike.baidu.com/view/84140.htm)  ▪ [RNC](http://baike.baidu.com/view/364500.htm)  ▪ [MRT](http://baike.baidu.com/view/1336474.htm) \| \| 原加盟电台 \| ▪ \| [CRK](http://baike.baidu.com/searchword/?word=CRK&pic=1&sug=1&enc=utf8) \|  \|  \|  \|  \|  \|  \|  \|  \|   参考资料    1 [加强大众科学认知肠易激综合征](https://baike.baidu.com/reference/10530572/266ftLVUuWJg1qDmaGdi8vSYeg9ImFQsvjUX2KTEq6scwAIJTwGmbZIw3mbPh0grgnwgp1kRhALnIBi8uAWGrE0SgspBbNsgWs7rY4nZqQxUx54) ．新华网[引用日期2015-04-22]  学术论文  内容来自    [李红缨，高丽，李宁秀. IBS-QOL专用量表在肠易激综合征患者中的运用．](https://xueshu.baidu.com/usercenter/paper/show?paperid=e8ae296528f4be36f74ad73fa71e4e44&tn=SE_baiduxueshu_c1gjeupa&ie=utf-8&site=baike) 《中国循证医学杂志》， 2004  [王世勇，陈曦，高权国. 艾灸治疗肠道易激综合征(IBS)30例临床观察．](https://xueshu.baidu.com/usercenter/paper/show?paperid=8f3562abecd2d4f54d67fa2d6b880780&tn=SE_baiduxueshu_c1gjeupa&ie=utf-8&site=baike) 《CNKI;WanFang》， 2003  [樊冬梅，刘凤斌，杨晓军等. 便秘型肠易激综合征(IBS)从脾论治的病机探微．](https://xueshu.baidu.com/usercenter/paper/show?paperid=b5eca34bd35010aa17e44ad5b7197e4a&tn=SE_baiduxueshu_c1gjeupa&ie=utf-8&site=baike) 《吉林中医药》， 2006  [李敏雅. 陆维宏辨证治疗肠易激综合征(IBS)经验拾萃．](https://xueshu.baidu.com/usercenter/paper/show?paperid=698afb956aebfc4359183ee1181f329b&tn=SE_baiduxueshu_c1gjeupa&ie=utf-8&site=baike) 《浙江中医药大学学报》， 2009  [康宁宁，潘迪，谭悦，付钰. 益生菌联合情志疗法对腹泻型肠易激综合征(IBS-D)患者的疗效及生存](https://xueshu.baidu.com/usercenter/paper/show?paperid=9dc3d60b304047ee76d8df11851e63ae&tn=SE_baiduxueshu_c1gjeupa&ie=utf-8&site=baike)  《VIP》， 2016  [查看全部](https://xueshu.baidu.com/s?wd=IBS+%E8%82%A0%E6%98%93%E6%BF%80%E7%BB%BC%E5%90%88%E5%BE%81&tn=SE_baiduxueshu_c1gjeupa&ie=utf-8&sc_from=pingtai6&site=baike) |

| 猜你喜欢 | [vvs欧洲供应商凯信贸易](http://www.baidu.com/baidu.php?url=Ks00000EAMrnlPLIyWswfF5Pk8AMjjS_PrGalQaSjuJY8_SUc2m93To1RXXexAu-OjKJmq-LAHr3tR97s3ViQcfrI4H_8SjSTdceMNV9WovmDCPi5JN6Uz5uJXYbCkkKBC-kz98ovfF7jB3Ju2PZhwwA9lS1GiiTBFDvqVIg6migruRXMVYNkTnV5zDqszEZe1gu4pOVXx1RWyaaQBuM8UGYq7Fw.Db_iIoQS9tNSaPtim3Y2pMpRt85R_nYQ7xu83tN0.U1Yk0ZDqiRFH0ZfqpyF10A-V5HDzPWc0Iybq0ZKGujYzn0KWpyfqP1c0mhbqn10k0AuY5H00TA6qn0KET1Ys0AFL5H00UMfqn0K1XWY0ThIYmyTqn0K8IM0qna3snj0snj0sn0K-ThTqn0KYTh7buHYdPH0znjD0mhwGujdaPHn3nDfsfbNanDmYPbuarDnvrHcdfbDYfHbkrj7An6KbmvPb5fK9TdqGuAnquj0VuLGCXZb0u1dLTv410ZFY5Hm4nfKkTA-b5H00TyPGujYs0A7B5HKxn0KsTjYs0AdYTjYs0AwbUL0qn0KzpWYs0ZwdT1YYnHR3rH0kPWc1rH6znHcsn10v0A7W5HD0TA3qn0Ksmgwxuhk9u1Ys0AN1IjYs0ANYpyfqQHD0mgPsmvnqn0KdTA-8mvnqn0KhmLNY5H00mywhUA7M5HD0IvuzUvYq0AFY5H00XZPYIHY1nHDYn163nfKzug7Y5HDvP104rjf4n1TYnHn0Tv-b5H0smhc1PjmYrjFhuj6vuAD0ULfqn0KETMKY5H0WnaPDw-fWnansc10Wna3sc10WwDuRc10WwDuR0AVG5H00UgfqnW0vn6KVm1YznjcLP1mvn1cLP0KVmdqhThqV5H00uA78IyF-gLK_my4GuZnqn0K9uZ745R-aRsK9uZ7Y5H00pgPWUjYs0Z7VIjYs0A7bgLPEIgFWuHYkranW0APzm1YzPjcvP0&us=newvui&xst=m1YKmWdaPHn3nDfsfbNanDmYPbuarDnvrHcdfbDYfHbkrj7An6715HRzPWb1PjcvrHf1rHD4rjnYg1DzPWFxn07L5y-BTs7k5y-BTs7d5HnknHf1rj6k0gfqnHmLnjb3Pjb1Ps7VTHYk0W0aiRFH0yPC5yuWgLKW0Hnkn1D3PHRzPj6&ai=0_429217685_1_0&word=&ck=0.0.0.0.0.0.0.0&shh=baike.baidu.com)  [ibs德国直采，原厂报价单报价，折扣优惠，可提供报关单，欧洲 工业品上游供应商，经营65家代理品牌， 2486家真实成交20000 … www.kaixinbusiness.com](http://www.baidu.com/baidu.php?url=Ks00000EAMrnlPLIyWswfF5Pk8AMjjS_PrGalQaSjuJY8_SUc2m93To1RXXexAu-OjKJmq-LAHr3tR97s3ViQcfrI4H_8SjSTdceMNV9WovmDCPi5JN6Uz5uJXYbCkkKBC-kz98ovfF7jB3Ju2PZhwwA9lS1GiiTBFDvqVIg6migruRXMVYNkTnV5zDqszEZe1gu4pOVXx1RWyaaQBuM8UGYq7Fw.Db_iIoQS9tNSaPtim3Y2pMpRt85R_nYQ7xu83tN0.U1Yk0ZDqiRFH0ZfqpyF10A-V5HDzPWc0Iybq0ZKGujYzn0KWpyfqP1c0mhbqn10k0AuY5H00TA6qn0KET1Ys0AFL5H00UMfqn0K1XWY0ThIYmyTqn0K8IM0qna3snj0snj0sn0K-ThTqn0KYTh7buHYdPH0znjD0mhwGujdaPHn3nDfsfbNanDmYPbuarDnvrHcdfbDYfHbkrj7An6KbmvPb5fK9TdqGuAnquj0VuLGCXZb0u1dLTv410ZFY5Hm4nfKkTA-b5H00TyPGujYs0A7B5HKxn0KsTjYs0AdYTjYs0AwbUL0qn0KzpWYs0ZwdT1YYnHR3rH0kPWc1rH6znHcsn10v0A7W5HD0TA3qn0Ksmgwxuhk9u1Ys0AN1IjYs0ANYpyfqQHD0mgPsmvnqn0KdTA-8mvnqn0KhmLNY5H00mywhUA7M5HD0IvuzUvYq0AFY5H00XZPYIHY1nHDYn163nfKzug7Y5HDvP104rjf4n1TYnHn0Tv-b5H0smhc1PjmYrjFhuj6vuAD0ULfqn0KETMKY5H0WnaPDw-fWnansc10Wna3sc10WwDuRc10WwDuR0AVG5H00UgfqnW0vn6KVm1YznjcLP1mvn1cLP0KVmdqhThqV5H00uA78IyF-gLK_my4GuZnqn0K9uZ745R-aRsK9uZ7Y5H00pgPWUjYs0Z7VIjYs0A7bgLPEIgFWuHYkranW0APzm1YzPjcvP0&us=newvui&xst=m1YKmWdaPHn3nDfsfbNanDmYPbuarDnvrHcdfbDYfHbkrj7An6715HRzPWb1PjcvrHf1rHD4rjnYg1DzPWFxn07L5y-BTs7k5y-BTs7d5HnknHf1rj6k0gfqnHmLnjb3Pjb1Ps7VTHYk0W0aiRFH0yPC5yuWgLKW0Hnkn1D3PHRzPj6&ai=0_429217685_1_0&word=&ck=0.0.0.0.0.0.0.0&shh=baike.baidu.com) |  | [德国ibs的产品，原装进口，欢迎询价 优势供应ibs的产品，报价快，货期稳定，品质保证，质优价 提供报关单和原产地证明，期待您的来电!](http://www.baidu.com/baidu.php?url=Ks00000EAMrnlPLIyVQ77okPpeChwzuhhaFGcwbivRbRi04bVLVLrdEXY6Dc9CpNAqWiPAjkjumaZdwlLDLncUM59Cw3TbAN_ze4QGQxlO0UWP6D6a9nkT8CUSNHJSscUec9LeJZelXx2ZeGUY_hfGTMPsaMStw8hdPKoGwCOUK1sABiF6Yp8RnnLarBGHPDPMpMDqNiDcn97yRhOp5yzdUmHCBR.7R_jv5sfdF1ulampbf2pMpRt85R_nYQAeI34Pl6.U1Yz0ZDqiRFH0ZfqpyF10A-V5HDzPWc0Iybq0ZKGujYzn0KWpyfqP1c0mhbqn10k0AuY5H00TA6qn0KET1Ys0AFL5H00UMfqn0K1XWY0ThIYmyTqn0K8IM0qna3snj0snj0sn0K-ThTqn0KYTh7buHYvPj0znjn0mhwGujdaPHn3nDfsfbNanDmYPbuarDnvrHcdfbDYfHbkrj7An6KbmvPb5fK9TdqGuAnquj0VuLGCXZb0u1dLTv410ZFY5Hm4nfKkTA-b5H00TyPGujYs0A7B5HKxn0KsTjYs0AdYTjYs0AwbUL0qn0KzpWYs0ZwdT1Y4njfdnj0LPWmdrjDdnjD4njnL0A7W5HD0TA3qn0Ksmgwxuhk9u1Ys0AN1IjYs0ANYpyfqQHD0mgPsmvnqn0KdTA-8mvnqn0KhmLNY5H00mywhUA7M5HD0IvuzUvYq0AFY5H00XZPYIHY1njn4Pjfdn0Kzug7Y5HDvP104rjf4n1TYnHn0Tv-b5H0smhc1PjmYrjFhuj6vuAD0ULfqn6KETMKY5HcWnandr1DLr1nsc1Dsnj0WnHmzPjmLPHmsPBnsc108nan1c1Dsnj0WnanV0AVG5H00UgfqnW0vn6KVm1YznWRknjbdPj6dP6KVmdqhThqV5H00uA78IyF-gLK_my4GuZnqn0K9uZ745R-aRsK9uZ7Y5H00pgPWUjYs0Z7VIjYs0A7bgLPEIgFWuHYkranW0APzm1Y1Pj0k&us=newvui&xst=m1YKmWdaPHn3nDfsfbNanDmYPbuarDnvrHcdfbDYfHbkrj7An6715HRzPWb1PjcvrHf1rHD4rjnYg1DzPWFxnf7L5y-BTs7k5y-BTs7d5Hnsn1bYPjRs0gfqnHmLnjb3Pjb1Ps7VTHYk0W0aiRFH0yPC5yuWgLKW0Hcvn1TznHf3rjn&ai=0_429147520_1_0&word=&ck=0.0.0.0.0.0.0.0&shh=baike.baidu.com)  [www.qiucheng-tech.com](http://www.baidu.com/baidu.php?url=Ks00000EAMrnlPLIyVQ77okPpeChwzuhhaFGcwbivRbRi04bVLVLrdEXY6Dc9CpNAqWiPAjkjumaZdwlLDLncUM59Cw3TbAN_ze4QGQxlO0UWP6D6a9nkT8CUSNHJSscUec9LeJZelXx2ZeGUY_hfGTMPsaMStw8hdPKoGwCOUK1sABiF6Yp8RnnLarBGHPDPMpMDqNiDcn97yRhOp5yzdUmHCBR.7R_jv5sfdF1ulampbf2pMpRt85R_nYQAeI34Pl6.U1Yz0ZDqiRFH0ZfqpyF10A-V5HDzPWc0Iybq0ZKGujYzn0KWpyfqP1c0mhbqn10k0AuY5H00TA6qn0KET1Ys0AFL5H00UMfqn0K1XWY0ThIYmyTqn0K8IM0qna3snj0snj0sn0K-ThTqn0KYTh7buHYvPj0znjn0mhwGujdaPHn3nDfsfbNanDmYPbuarDnvrHcdfbDYfHbkrj7An6KbmvPb5fK9TdqGuAnquj0VuLGCXZb0u1dLTv410ZFY5Hm4nfKkTA-b5H00TyPGujYs0A7B5HKxn0KsTjYs0AdYTjYs0AwbUL0qn0KzpWYs0ZwdT1Y4njfdnj0LPWmdrjDdnjD4njnL0A7W5HD0TA3qn0Ksmgwxuhk9u1Ys0AN1IjYs0ANYpyfqQHD0mgPsmvnqn0KdTA-8mvnqn0KhmLNY5H00mywhUA7M5HD0IvuzUvYq0AFY5H00XZPYIHY1njn4Pjfdn0Kzug7Y5HDvP104rjf4n1TYnHn0Tv-b5H0smhc1PjmYrjFhuj6vuAD0ULfqn6KETMKY5HcWnandr1DLr1nsc1Dsnj0WnHmzPjmLPHmsPBnsc108nan1c1Dsnj0WnanV0AVG5H00UgfqnW0vn6KVm1YznWRknjbdPj6dP6KVmdqhThqV5H00uA78IyF-gLK_my4GuZnqn0K9uZ745R-aRsK9uZ7Y5H00pgPWUjYs0Z7VIjYs0A7bgLPEIgFWuHYkranW0APzm1Y1Pj0k&us=newvui&xst=m1YKmWdaPHn3nDfsfbNanDmYPbuarDnvrHcdfbDYfHbkrj7An6715HRzPWb1PjcvrHf1rHD4rjnYg1DzPWFxnf7L5y-BTs7k5y-BTs7d5Hnsn1bYPjRs0gfqnHmLnjb3Pjb1Ps7VTHYk0W0aiRFH0yPC5yuWgLKW0Hcvn1TznHf3rjn&ai=0_429147520_1_0&word=&ck=0.0.0.0.0.0.0.0&shh=baike.baidu.com) |
| --- | --- | --- | --- |
|  | | | |
| 岔 搜索发现 |  | | |

<https://baike.baidu.com/item/IBS/10530572?fromModule=lemma_search-box>

3/4

2022/12/14 10:29

IBS (肠易激综合征) _百度百科

| [肠易激综合征吃什么药](https://www.baidu.com/s?word=%E8%82%A0%E6%98%93%E6%BF%80%E7%BB%BC%E5%90%88%E5%BE%81%E5%90%83%E4%BB%80%E4%B9%88%E8%8D%AF&tn=SE_baikepcxf02_fcetbk02&pos=baike_pc_turbo_1767&ori_sid=00bb34639a7e9090)  [robey+warshaw](https://www.baidu.com/s?word=robey%2Bwarshaw&tn=SE_baikepcxf02_fcetbk02&pos=baike_pc_turbo_1767&ori_sid=00bb34639a7e9090) |
| --- |

肠易激综合征的症状 珠海ibs

[编辑](javascript:;)

全日制英语

[ibs是什么意思](https://www.baidu.com/s?word=ibs%E6%98%AF%E4%BB%80%E4%B9%88%E6%84%8F%E6%80%9D&tn=SE_baikepcxf02_fcetbk02&pos=baike_pc_turbo_1767&ori_sid=00bb34639a7e9090)

[收藏](javascript:;)

英语学校

[IBS主要症状](https://www.baidu.com/s?word=IBS%E4%B8%BB%E8%A6%81%E7%97%87%E7%8A%B6&tn=SE_baikepcxf02_fcetbk02&pos=baike_pc_turbo_1767&ori_sid=00bb34639a7e9090)

ibs学校 ibs酒店

[回 讨论](https://baike.baidu.com/planet/talk?lemmaId=10530572)

小 播报

[赞](javascript:;)

新手上路

我有疑问

投诉建议

[成长任务](https://baike.baidu.com/usercenter/tasks#guide) [编辑规则](https://baike.baidu.com/help#main06)

[编辑入门](https://baike.baidu.com/help#main01) [内容质疑](javascript:void(0);)

[本人编辑](https://baike.baidu.com/item/%E7%99%BE%E5%BA%A6%E7%99%BE%E7%A7%91%EF%BC%9A%E6%9C%AC%E4%BA%BA%E8%AF%8D%E6%9D%A1%E7%BC%96%E8%BE%91%E6%9C%8D%E5%8A%A1/22442459?bk_fr=pcFooter) [官方贴吧](http://tieba.baidu.com/f?ie=utf-8&fr=bks0000&kw=%E7%99%BE%E5%BA%A6%E7%99%BE%E7%A7%91)

[在线客服](http://zhiqiu.baidu.com/baike/passport/html/baikechat.html)

[意见反馈](javascript:void(0);)

[举报不良信息](http://help.baidu.com/newadd?word=IBS&&submit_link=https%3A%2F%2Fbaike.baidu.com%2Fitem%2FIBS%2F10530572%3FfromModule%3Dlemma_search-box&prod_id=10&category=1) [投诉侵权信息](http://help.baidu.com/newadd?word=IBS&&submit_link=https%3A%2F%2Fbaike.baidu.com%2Fitem%2FIBS%2F10530572%3FfromModule%3Dlemma_search-box&prod_id=10&category=6)

[未通过词条申诉](http://help.baidu.com/newadd?word=IBS&&submit_link=https%3A%2F%2Fbaike.baidu.com%2Fitem%2FIBS%2F10530572%3FfromModule%3Dlemma_search-box&prod_id=10&category=2)

[封禁查询与解封](http://help.baidu.com/newadd?word=IBS&&submit_link=https%3A%2F%2Fbaike.baidu.com%2Fitem%2FIBS%2F10530572%3FfromModule%3Dlemma_search-box&prod_id=10&category=5)

©2022 Baidu [使用百度前必读](http://www.baidu.com/duty/) | [百科协议](http://help.baidu.com/question?prod_en=baike&class=89&id=1637) | [隐私政策](http://help.baidu.com/question?prod_id=10&class=690&id=1001779) | [百度百科合作平台](https://baike.baidu.com/operation/cooperation) | 京ICP证030173号

[京公网安备11000002000001号](http://www.beian.gov.cn/portal/registerSystemInfo?recordcode=11000002000001)

<https://baike.baidu.com/item/IBS/10530572?fromModule=lemma_search-box>

4/4
