# Supplementary material for: Assessment of medical information on irritable bowel syndrome information in Wikipedia and Baidu Encyclopedia: comparative study
Source: PeerJ. 2024 May 24;12:e17264. doi: 10.7717/peerj.17264 (PMC11129691; doi:10.7717/peerj.17264)
Supplement: Data S1 [file peerj-12-17264-s001.zip › σÄƒσoïμò░μì«/Baidu/Baidu-Chinese/2-Φéáμÿôμ┐Çτ╗╝σÉêσ╛ü∩╝êΦéáμÿôμ┐Çτ╗╝σÉêσ╛ü∩╝ë_τÖ╛σ║aτÖ╛τoæ.docx]

2022/12/14 10:30

[岔](https://baike.baidu.com/)

肠易激综合征

进入词条

[岔](https://baike.baidu.com/)

肠易激综合征(肠易激综合征) _百度百科

| [~~网页~~](https://www.baidu.com/) [~~新闻~~](http://news.baidu.com/) [~~贴吧~~](https://tieba.baidu.com/) [知道](https://zhidao.baidu.com/) | | | | | [网盘](https://pan.baidu.com/?from=1027327l) | [图片](http://image.baidu.com/) | [视频](http://v.baidu.com/)    播报 | | [地图](http://map.baidu.com/) | [文库](https://wenku.baidu.com/)  编辑 | | 百科  收藏 | [百度首页](http://www.baidu.com/) 赞 | [登录](javascript:;) |
| --- | --- | --- | --- | --- | --- | --- | --- | --- | --- | --- | --- | --- | --- | --- |
|  | | | | | | | | | | | | | | |
|  | |  | | | | | |  | | |  | |  | |
|  | | 肠易激综合征 | | | | | | 进入词条 | | | 全站搜索 | | [帮助](https://baike.baidu.com/help) | |
| 近期有不法分子冒充百度百科官方人员，以删除词条为由威胁并敲诈相关企业。在此严正声明：百度百科是免费编辑平台，绝不存在收费代编服务，请勿上当受骗！ [详情>>](https://baike.baidu.com/common/declaration) | | | | | | | | | | | | |  |  |
| [首页](https://baike.baidu.com/) 秒懂百科 特色百科 用户 知识专题 权威合作 [口下载百科APP](https://baike.baidu.com/wapui/subpage/baikeappdownload?sfrom=pc_lemmapage_navigation) [2 个](https://baike.baidu.com/usercenter) | | | | | | | | | | | | | | |
| 肠易激综合征是一个[多义词](https://baike.baidu.com/item/%E7%99%BE%E5%BA%A6%E7%99%BE%E7%A7%91%EF%BC%9A%E5%A4%9A%E4%B9%89%E8%AF%8D)，请在下列[义项](https://baike.baidu.com/item/%E4%B9%89%E9%A1%B9)上选择浏览([共3个义项](https://baike.baidu.com/item/%E8%82%A0%E6%98%93%E6%BF%80%E7%BB%BC%E5%90%88%E5%BE%81?force=1)) [展开 添加义项](javascript:;)      肠易激综合征可防可治，三种治疗方 …  05:25    肠易激综合征  [编辑](javascript:;)  [播报](javascript:;)  [上传视频](javascript:;)      什么是肠易激综合征？  02:22 | | | | | | | | | | | | | | |
| [女疊](javascript:void(0);)  收藏  [2411](javascript:void(0);) 556  本词条由[“科普中国”科学百科词条编写与应用工作项目](https://baike.baidu.com/science) 认证 。  肠易激综合征(irritable bowel syndrome， IBS)是一组持续或间歇发作，以腹痛、腹胀、排便习惯和(或)大便性状改变 为临床表现，而缺乏胃肠道结构和生化异常的肠道功能紊乱性疾病。罗马Ⅲ将其列为功能性肠病的一类，患者以中青年人为主， 发病年龄多见于20 ~ 50岁，女性较男性多见，有家族聚集倾向，常与其他胃肠道功能紊乱性疾病如功能性消化不良并存伴发。按 照大便的性状将IBS分为腹泻型、便秘型、混合型和不定型四种临床类型，我国以腹泻为主型多见。  外文名 irritable bowel syndrome  专家解读   \| 就诊科室 \| 消化内科 \| \| --- \| --- \| \| 多发群体 \| 20 ~ 50岁女性 \|   [肠易激综合征如何治疗？](javascript:;)  [对肠易激综合征的一般治疗包括建立良好的](javascript:;)  [生活习惯，饮食上避免产气的食物。](javascript:;)   \| 科普中国  审核  致力于权威的科学传播  本词条认证专家为  屠惠明 丨主任医师   \|  \| \| --- \|   无锡市第四人民医院 消化内  审核  韩英 丨主任医师   \|  \| \| --- \|   陆军总院 消化科 \| \| --- \| --- \| --- \|   多与精神因素、应激状态及肠功能…  常见病因 | | | | | | | | | | | | | | |
|  | | | | | | | | | | | | | | |
| 疾病概况 | 科普文章 (1) | | 科普视频 | [口](javascript:void(0);) | | | | | | | | | | |
| \|  \| \| --- \|  \| 权威合作编辑  [“科普中国”科学百科词条编](http://www.kepuchina.cn/)  “科普中国”是为我国科普信 建设塑造的全...  [什么是权威编辑](http://baike.bdimg.com/cms/static/cooperation/content.pdf) \| \| --- \|  \|  \| \| --- \|  \| 目录 \| 1 [病因](#_bookmark1)  2 [临床表现](#_bookmark2)  3 [检查](#_bookmark3)  4 [诊断](#_bookmark4)  5 [治疗](#_bookmark5) \| \| --- \| --- \|   基本信息   \| 资源提供  [科普中国](http://www.cast.org.cn/)  “科普中国”是中国科协... 提供资源类型： 资源  [什么是资源合作](http://baike.bdimg.com/cms/static/cooperation/content.pdf) \| \| --- \|   irritable bowel syndrome  多与精神因素、应激状态及肠功能紊乱有关 腹痛、腹胀、腹泻、便秘、烧心、恶心、呕吐  无  常见病因 常见症状  传染性  外文名 就诊科室  多发群体  消化内科  20 ~ 50岁女性  [播报编辑](javascript:;)  词条统计  病因  浏览次数： 4464651次  IBS的病因和发病机制尚不十分清楚，被认为是胃肠动力异常、内脏感觉异常、脑肠调控异常、炎症和精神心理等多种因素  编辑次数： 66次[历史版本](https://baike.baidu.com/historylist/%E8%82%A0%E6%98%93%E6%BF%80%E7%BB%BC%E5%90%88%E5%BE%81/8456761)  最近更新： [w_ou](https://baike.baidu.com/usercenter/userpage?uk=TY3CXj_hJSEcBfJBVP43Rg&from=lemma) ( 2018-08-11)  突出贡献榜  [haodf_hz](https://baike.baidu.com/usercenter/userpage?uk=FJEYwTDtnGuUTco98fnJhA&from=lemma)  [tougao16](https://baike.baidu.com/usercenter/userpage?uk=eNoJN598ZtdRfaWZCW2Q1Q&from=lemma)  共同作用的结果。  **1.**胃肠道动力紊乱  肠道动力变化是IBS 症状发生的重要病理生理基础。以腹泻为主的IBS患者呈肠道动力亢进的表现，小肠传输时间显著缩  短，结肠动力指数和高幅推进性收缩的均值和最大值均明显提高。便秘型IBS则正好相反，表现为肠道动力不足。 | | | | | | | | | | | | | | |

<https://baike.baidu.com/item/>肠易激综合征/8456761?fromModule=lemma_inlink

1/4

2022/12/14 10:30

肠易激综合征(肠易激综合征) _百度百科

| gjhxw  [岔](https://baike.baidu.com/)  [编辑](javascript:;)  [收藏](javascript:;)  小 播报  [赞](javascript:;)  **2.**内脏感觉异常  研究发现IBS患者多数具有对管腔(直肠)扩张感觉过敏的临床特征，其平均痛觉阈值下降，直肠扩张后的不适程度增强或 | | |
| --- | --- | --- |
| 有异常的内脏-躯体放射痛，提示脊髓水平对内脏感觉信号处理的异常。  **3.**中枢感觉异常  研究表明， IBS患者其内脏疼痛的中枢通路与正常人有所不同，且腹泻型IBS与便秘型IBS之间的大脑反应区也有所不同。  **4.**脑**-**肠轴调节异常  IBS患者存在中枢神经系统对肠道传入信号的处理及对肠神经系统的调节异常。  **5.**肠道感染与炎症反应  研究显示，急性肠道感染后发生IBS的几率大大增高，因此肠道急性感染被认为是诱发IBS的危险因素之一。肠道感染引起的 黏膜炎症反应，通透性增加及免疫功能激活与IBS发病的关系值得进一步研究。  **6.**精神心理因素  IBS患者常有焦虑、紧张、抑郁等心理异常。同时精神心理应激也可诱发或加重IBS症状，说明精神心理因素与IBS有密切的 关系。 | | [女疊 口](javascript:void(0);) |
| 临床表现 | [小 播报编辑](javascript:;) |  |
| IBS无特异性症状，但相对于器质性胃肠疾病，具有一些特点：起病缓慢，间歇性发作；病程长但全身健康状况不受影响； 症状的出现或加重常与精神因素或应激状态有关；白天明显，夜间睡眠后减轻。  **1.**症状  ( **1**)腹痛或腹部不适 是IBS的主要症状，伴有大便次数或形状的异常，腹痛多于排便后缓解，部分病人易在进食后出现， 腹痛可发生于腹部任何部位，局限性或弥漫性，疼痛性质多样。腹痛不会进行性加重，夜间睡眠后极少有痛醒者。  (**2**)腹泻 ①持续性或间歇性腹泻，粪量少，呈糊状，含大量黏液；②禁食72小时后症状消失；③夜间不出现，有别于器质 性疾患；④部分患者可因进食诱发；⑤患者可有腹泻与便秘交替现象。  (**3**)便秘 排便困难，大便干结，量少，可带较多黏液，便秘可间断或与腹泻相交替，常伴排便不尽感。  (**4**)腹胀 白天较重，尤其在午后，夜间睡眠后减轻。  (**5**)上胃肠道症状 近半数患者有胃烧灼感、恶心、呕吐等上胃肠道症状。  (**6**)肠外症状 背痛、头痛、心悸、尿频、尿急、性功能障碍等胃肠外表现较器质性肠病显著多见，部分病人尚有不同程度 的心理精神异常表现，如焦虑、抑郁、紧张等。  **2.**体征  通常无阳性发现，或仅有腹部轻压痛。部分患者有多汗，脉快，血压高等自主神经失调表现，有时可于腹部触及乙状结肠曲 或痛性肠襻。直肠指检可感到肛门痉挛、张力高，可有触痛。 | |  |
| 检查 | [小 播报编辑](javascript:;) |  |
| 旨在排除器质性病变，多次(至少3次)大便常规培养均阴性，便隐血试验阴性，血尿常规正常，血沉正常，甲状腺、肝、 胆、胰腺、肾功能正常。对于年龄40岁以上患者，除上述检查外，尚需进行结肠镜检查并进行黏膜活检以除外肠道感染性、肿瘤 性疾病等。钡剂灌肠X线检查和腹部超声检查，也常用来进行排除诊断。 | |  |
| 诊断 | [小 播报编辑](javascript:;) |  |
| IBS诊断标准以症状学为依据，诊断建立在排除器质性疾病的基础上，推荐采用目前国际公认的IBS罗马Ⅲ诊断标准：  反复发作的腹痛或不适(不适意味着感觉不舒服而非疼痛)，最近3个月内每个月至少有3天出现症状，合并以下2条或多 条：①排便后症状缓解；②发作时伴有排便频率改变；③发作时伴有大便性状(外观)改变。  诊断前症状出现至少6个月,近3个月符合以上标准。  以下症状对诊断具有支持意义，包括①排便频率异常(每周排便少于3次，或每日排便多于3次)；②粪便性状异常(干粪球 或硬粪，或糊状粪/稀水粪)；③排便费力；④排便急迫感、排便不尽、排黏液便以及腹胀。 | |  |
| 治疗 | [小 播报编辑](javascript:;) |  |
| 目前对IBS的治疗只限于对症处理。中华医学会消化病分会胃肠动力学组在《肠易激综合征诊断和治疗的共识意见》中提  出： “治疗目的是消除患者顾虑，改善症状，提高生活质量。治疗原则是建立在良好医患关系的基础上，根据主要症状类型进行症 | |  |

<https://baike.baidu.com/item/>肠易激综合征/8456761?fromModule=lemma_inlink

2/4

2022/12/14 10:30

肠易激综合征(肠易激综合征) _百度百科

| 状治疗和根据症状严重程度进行分级治疗。注意治疗措施的个体化和综合运用。 ”  小 播报  [编辑](javascript:;)  [收藏](javascript:;)  [赞](javascript:;)  [时岔謂](https://baike.baidu.com/)  **1.**调整饮食 | | | |
| --- | --- | --- | --- |
|  | | | |
| 详细了解病人的饮食习惯及其与症状的关系，避免敏感食物，避免过量的脂肪及刺激性食物如咖啡、浓茶、酒精等，并减少 产气食物(奶制品，大豆，扁豆等)的摄取。高纤维素食物(如麸糠)可刺激结肠运动，对改善便秘有明显效果。  **2.**心理和行为治疗  对病人进行耐心的解释工作，具体包括心理治疗，生物反馈疗法等，对于有失眠，焦虑等症状者，可适当予以镇静药。  **3.**药物治疗  ( **1**)解痉剂 目前使用较为普遍的是选择性肠道平滑肌钙离子通道拮抗剂，或离子通道调节剂。抗胆碱能药如阿托品、颠 茄、莨菪碱类也能改善腹痛症状，但需注意不良反应。  (**2**)通便剂 便秘可使用导泻药，可试用容积性泻剂如甲基纤维素和渗透性轻泻剂如聚乙二醇、乳果糖等。刺激性泻剂应慎 用。  (**3**)止泻剂 如洛哌丁胺或复方苯乙哌啶等，可改善腹泻，需注意便秘、腹胀等不良反应。轻症者可选用八面体蒙脱石等吸 附剂。  (**4**)促动力剂 适用于有腹胀和便秘型患者。常用的有西沙比利或莫沙必利等。  (**5**)内脏止痛剂 生长抑素及其类似物如奥曲肽，具有缓解躯体和内脏疼痛的作用。 5-HT3受体阻滞剂如阿洛司琼，能改善  腹泻型患者的腹痛及大便次数，可引起缺血性结肠炎等不良反应，使用时需注意。 5-HT4受体的部分激动剂因其存在增加心血管 缺血事件的风险，已被停止使用。  (**6**)抗精神病药 对腹痛症状重而上述治疗无效，尤其是具有明显精神症状的患者，适当予以镇静剂，抗抑郁药，抗焦虑药 有一定帮助。  (**7**)益生菌 能调整宿主肠道微生物群生态平衡，适用于伴有肠道菌群失调的IBS患者。  **4.**中医药治疗  中医将IBS分为大肠燥热证、寒热夹杂证、脾胃虚弱证、肝气乘脾证、肝郁气滞证等证型，通过辨证论治、针灸等方法，对 IBS的治疗取得了一定疗效，仍有待于今后进一步研究。 | | | [女](javascript:void(0);) [公](javascript:void(0);) [.](javascript:void(0);) [疊](javascript:void(0);) [口](javascript:void(0);) |
| 学术论文 | | 内容来自 |  |
| [Wang等. 中医治疗肠易激综合征的研究进展：肠易激综合征中医证候学特点的认](https://xueshu.baidu.com/usercenter/paper/show?paperid=78a927fc08b2691ebc68d605d8782574&tn=SE_baiduxueshu_c1gjeupa&ie=utf-8&site=baike) 《世界华人消化杂志》， 2010  [徐启旺，钱家鸣，窦君，余平. 细菌潜生体相关的肠易激综合征动物模型的建立及细菌潜生体在肠易激](https://xueshu.baidu.com/usercenter/paper/show?paperid=a3b9f8b3196ac7e7060e220463381710&tn=SE_baiduxueshu_c1gjeupa&ie=utf-8&site=baike) 《胃肠病学》， 2003  [肠易激综合征联合治疗对肠易激综合征病人心理精神因素的疗效评价．](https://xueshu.baidu.com/usercenter/paper/show?paperid=9b7dc75fd3592c42014a83aaa7e5b0ac&tn=SE_baiduxueshu_c1gjeupa&ie=utf-8&site=baike)陈艳. 《临床和实验医学杂志》， 2006  [陈达氏. 肠易激综合征患者的胃肠动力：肠易激综合征是否为一种动力紊乱．](https://xueshu.baidu.com/usercenter/paper/show?paperid=bbe2321662e895447e4461a48598b550&tn=SE_baiduxueshu_c1gjeupa&ie=utf-8&site=baike) 《vip》， 1994  [胡品津. 肠易激综合征诊治共识意见．](https://xueshu.baidu.com/usercenter/paper/show?paperid=bc9d014868c0ff198ec4f033c85d6ead&tn=SE_baiduxueshu_c1gjeupa&ie=utf-8&site=baike) 《 CNKI》， 2003  [查看全部](https://xueshu.baidu.com/s?wd=%E8%82%A0%E6%98%93%E6%BF%80%E7%BB%BC%E5%90%88%E5%BE%81+%E8%82%A0%E6%98%93%E6%BF%80%E7%BB%BC%E5%90%88%E5%BE%81&tn=SE_baiduxueshu_c1gjeupa&ie=utf-8&sc_from=pingtai6&site=baike) | | |  |
|  | | | |
| 猜你喜欢 | [幽门螺旋杆菌，吃益生菌有用吗?高活性益生菌](http://www.baidu.com/baidu.php?url=Ks00000EAMrnlPLIyGAY4b9NmgBNriocqp7ssdviP3k5x_WGkL4dsRYNizs9gBg55tnfcD06t02_AheBhNtmO3AwqyMeGjv8WBZlDPYws_JSEdqb8KfdQ1Jh-04LVaibwM_WsWRDRnPEZIyNcFG2me2WuLUMoghdgMLXWOzOiBCPeuvs_zYeRmVbI9PBC1w0dfkLzkXnB88nR3i6zIrZiGWb7Lfe.7D_NR2Ar5Od66xfHGt_Vzc2eQr1k_lX1uEooo3tdPHV2XgZJyAp7WW8e2O7f.U1Yk0ZDq_lUidezbdq8l1qgL0ZfqEJv1CtaVV_xgYe5RzoQjVQojVxx0eUrh1o60pyYqnHcvn6KdpHY0TA-b5Hcs0APGujYLn6KBpHY1njD0uMfqn0KspjYs0Aq15H00mMTqn0K8IjYs0ZPl5fKzuLw9u1Ys0A4vTjYsQW0snj0snj0s0ANzu1Ys0Zwzmyw-5H00mhwGujYznRNafWb1wbNAnbfkwDm3rDNjwjf3fRf4wDDYf1mzffKbmvPb5fK9TdqGuAnqTZnVuLGCXZb0u1dLTv410ZFY5Hn4P0KkTA-b5H00TyPGujYs0A7B5HKxn0KsTjYs0AdYTjYs0AwbUL0qn0KzpWYs0ZwdT1YvnWRsn103Pjf4PHf1P1n3rHbk0A7W5HT0TA3qn0Ksmgwxuhk9u1Ys0AN1IjY1n6K-IA-b5iYk0A71TAPW5H00IgKGUhPW5H00uhPdIjYs0A7buhk9u1Yk0ZIhThqV5fKBIjYk0ZF-TgfqnHmLnjb3Pjb3rj63P6K1pyfqnjKBmWnYP1DvmH-hn1F9ufKEIjYs0AqzTZfqnanscYwANansc10WnansQW0WnaPDw-fWnaPDw-f0pvbqn0KVIjYznjmz0AdW5HcLn1D3PWT1nWTd0AdWgvuzUvYqn0Kbmy4dmhNxTAk9Uh-bT1Ys0A7bTgbq8QMf8VpBdef0mywkIjYs0A-1mvsqn0KkUgfqn0K9u7q1ULNzmvRqnWnWIv-1uNqYULKxmv7WpARWgdqxgv41cLwGIAk-cvPYmLuzTNqCXNqGTvt0mLFW5HcvrHbs&us=newvui&ai=0_429414813_1_0&word=&ck=0.0.0.0.0.0.0.0&shh=baike.baidu.com)  [幽门螺旋杆菌哪些症状，感染幽门螺旋杆菌怎么办，幽门螺杆菌是](http://www.baidu.com/baidu.php?url=Ks00000EAMrnlPLIyGAY4b9NmgBNriocqp7ssdviP3k5x_WGkL4dsRYNizs9gBg55tnfcD06t02_AheBhNtmO3AwqyMeGjv8WBZlDPYws_JSEdqb8KfdQ1Jh-04LVaibwM_WsWRDRnPEZIyNcFG2me2WuLUMoghdgMLXWOzOiBCPeuvs_zYeRmVbI9PBC1w0dfkLzkXnB88nR3i6zIrZiGWb7Lfe.7D_NR2Ar5Od66xfHGt_Vzc2eQr1k_lX1uEooo3tdPHV2XgZJyAp7WW8e2O7f.U1Yk0ZDq_lUidezbdq8l1qgL0ZfqEJv1CtaVV_xgYe5RzoQjVQojVxx0eUrh1o60pyYqnHcvn6KdpHY0TA-b5Hcs0APGujYLn6KBpHY1njD0uMfqn0KspjYs0Aq15H00mMTqn0K8IjYs0ZPl5fKzuLw9u1Ys0A4vTjYsQW0snj0snj0s0ANzu1Ys0Zwzmyw-5H00mhwGujYznRNafWb1wbNAnbfkwDm3rDNjwjf3fRf4wDDYf1mzffKbmvPb5fK9TdqGuAnqTZnVuLGCXZb0u1dLTv410ZFY5Hn4P0KkTA-b5H00TyPGujYs0A7B5HKxn0KsTjYs0AdYTjYs0AwbUL0qn0KzpWYs0ZwdT1YvnWRsn103Pjf4PHf1P1n3rHbk0A7W5HT0TA3qn0Ksmgwxuhk9u1Ys0AN1IjY1n6K-IA-b5iYk0A71TAPW5H00IgKGUhPW5H00uhPdIjYs0A7buhk9u1Yk0ZIhThqV5fKBIjYk0ZF-TgfqnHmLnjb3Pjb3rj63P6K1pyfqnjKBmWnYP1DvmH-hn1F9ufKEIjYs0AqzTZfqnanscYwANansc10WnansQW0WnaPDw-fWnaPDw-f0pvbqn0KVIjYznjmz0AdW5HcLn1D3PWT1nWTd0AdWgvuzUvYqn0Kbmy4dmhNxTAk9Uh-bT1Ys0A7bTgbq8QMf8VpBdef0mywkIjYs0A-1mvsqn0KkUgfqn0K9u7q1ULNzmvRqnWnWIv-1uNqYULKxmv7WpARWgdqxgv41cLwGIAk-cvPYmLuzTNqCXNqGTvt0mLFW5HcvrHbs&us=newvui&ai=0_429414813_1_0&word=&ck=0.0.0.0.0.0.0.0&shh=baike.baidu.com)  [胃部发炎引起的怎么治幽门螺旋杆菌，恶心干呕，易导致口臭， …](http://www.baidu.com/baidu.php?url=Ks00000EAMrnlPLIyGAY4b9NmgBNriocqp7ssdviP3k5x_WGkL4dsRYNizs9gBg55tnfcD06t02_AheBhNtmO3AwqyMeGjv8WBZlDPYws_JSEdqb8KfdQ1Jh-04LVaibwM_WsWRDRnPEZIyNcFG2me2WuLUMoghdgMLXWOzOiBCPeuvs_zYeRmVbI9PBC1w0dfkLzkXnB88nR3i6zIrZiGWb7Lfe.7D_NR2Ar5Od66xfHGt_Vzc2eQr1k_lX1uEooo3tdPHV2XgZJyAp7WW8e2O7f.U1Yk0ZDq_lUidezbdq8l1qgL0ZfqEJv1CtaVV_xgYe5RzoQjVQojVxx0eUrh1o60pyYqnHcvn6KdpHY0TA-b5Hcs0APGujYLn6KBpHY1njD0uMfqn0KspjYs0Aq15H00mMTqn0K8IjYs0ZPl5fKzuLw9u1Ys0A4vTjYsQW0snj0snj0s0ANzu1Ys0Zwzmyw-5H00mhwGujYznRNafWb1wbNAnbfkwDm3rDNjwjf3fRf4wDDYf1mzffKbmvPb5fK9TdqGuAnqTZnVuLGCXZb0u1dLTv410ZFY5Hn4P0KkTA-b5H00TyPGujYs0A7B5HKxn0KsTjYs0AdYTjYs0AwbUL0qn0KzpWYs0ZwdT1YvnWRsn103Pjf4PHf1P1n3rHbk0A7W5HT0TA3qn0Ksmgwxuhk9u1Ys0AN1IjY1n6K-IA-b5iYk0A71TAPW5H00IgKGUhPW5H00uhPdIjYs0A7buhk9u1Yk0ZIhThqV5fKBIjYk0ZF-TgfqnHmLnjb3Pjb3rj63P6K1pyfqnjKBmWnYP1DvmH-hn1F9ufKEIjYs0AqzTZfqnanscYwANansc10WnansQW0WnaPDw-fWnaPDw-f0pvbqn0KVIjYznjmz0AdW5HcLn1D3PWT1nWTd0AdWgvuzUvYqn0Kbmy4dmhNxTAk9Uh-bT1Ys0A7bTgbq8QMf8VpBdef0mywkIjYs0A-1mvsqn0KkUgfqn0K9u7q1ULNzmvRqnWnWIv-1uNqYULKxmv7WpARWgdqxgv41cLwGIAk-cvPYmLuzTNqCXNqGTvt0mLFW5HcvrHbs&us=newvui&ai=0_429414813_1_0&word=&ck=0.0.0.0.0.0.0.0&shh=baike.baidu.com)  [item.jd.com](http://www.baidu.com/baidu.php?url=Ks00000EAMrnlPLIyGAY4b9NmgBNriocqp7ssdviP3k5x_WGkL4dsRYNizs9gBg55tnfcD06t02_AheBhNtmO3AwqyMeGjv8WBZlDPYws_JSEdqb8KfdQ1Jh-04LVaibwM_WsWRDRnPEZIyNcFG2me2WuLUMoghdgMLXWOzOiBCPeuvs_zYeRmVbI9PBC1w0dfkLzkXnB88nR3i6zIrZiGWb7Lfe.7D_NR2Ar5Od66xfHGt_Vzc2eQr1k_lX1uEooo3tdPHV2XgZJyAp7WW8e2O7f.U1Yk0ZDq_lUidezbdq8l1qgL0ZfqEJv1CtaVV_xgYe5RzoQjVQojVxx0eUrh1o60pyYqnHcvn6KdpHY0TA-b5Hcs0APGujYLn6KBpHY1njD0uMfqn0KspjYs0Aq15H00mMTqn0K8IjYs0ZPl5fKzuLw9u1Ys0A4vTjYsQW0snj0snj0s0ANzu1Ys0Zwzmyw-5H00mhwGujYznRNafWb1wbNAnbfkwDm3rDNjwjf3fRf4wDDYf1mzffKbmvPb5fK9TdqGuAnqTZnVuLGCXZb0u1dLTv410ZFY5Hn4P0KkTA-b5H00TyPGujYs0A7B5HKxn0KsTjYs0AdYTjYs0AwbUL0qn0KzpWYs0ZwdT1YvnWRsn103Pjf4PHf1P1n3rHbk0A7W5HT0TA3qn0Ksmgwxuhk9u1Ys0AN1IjY1n6K-IA-b5iYk0A71TAPW5H00IgKGUhPW5H00uhPdIjYs0A7buhk9u1Yk0ZIhThqV5fKBIjYk0ZF-TgfqnHmLnjb3Pjb3rj63P6K1pyfqnjKBmWnYP1DvmH-hn1F9ufKEIjYs0AqzTZfqnanscYwANansc10WnansQW0WnaPDw-fWnaPDw-f0pvbqn0KVIjYznjmz0AdW5HcLn1D3PWT1nWTd0AdWgvuzUvYqn0Kbmy4dmhNxTAk9Uh-bT1Ys0A7bTgbq8QMf8VpBdef0mywkIjYs0A-1mvsqn0KkUgfqn0K9u7q1ULNzmvRqnWnWIv-1uNqYULKxmv7WpARWgdqxgv41cLwGIAk-cvPYmLuzTNqCXNqGTvt0mLFW5HcvrHbs&us=newvui&ai=0_429414813_1_0&word=&ck=0.0.0.0.0.0.0.0&shh=baike.baidu.com) | | |

| 岔 搜索发现  [治疗肠易激的中成药](https://www.baidu.com/s?word=%E6%B2%BB%E7%96%97%E8%82%A0%E6%98%93%E6%BF%80%E7%9A%84%E4%B8%AD%E6%88%90%E8%8D%AF&tn=SE_baikepcxf02_fcetbk02&pos=baike_pc_turbo_1767&ori_sid=00bb34716a7c54d6) [肠激惹综合症吃什么药](https://www.baidu.com/s?word=%E8%82%A0%E6%BF%80%E6%83%B9%E7%BB%BC%E5%90%88%E7%97%87%E5%90%83%E4%BB%80%E4%B9%88%E8%8D%AF&tn=SE_baikepcxf02_fcetbk02&pos=baike_pc_turbo_1767&ori_sid=00bb34716a7c54d6) | [肝郁脾虚最好中成药](https://www.baidu.com/s?word=%E8%82%9D%E9%83%81%E8%84%BE%E8%99%9A%E6%9C%80%E5%A5%BD%E4%B8%AD%E6%88%90%E8%8D%AF&tn=SE_baikepcxf02_fcetbk02&pos=baike_pc_turbo_1767&ori_sid=00bb34716a7c54d6)  [肠易激综合征的症状特点](https://www.baidu.com/s?word=%E8%82%A0%E6%98%93%E6%BF%80%E7%BB%BC%E5%90%88%E5%BE%81%E7%9A%84%E7%97%87%E7%8A%B6%E7%89%B9%E7%82%B9&tn=SE_baikepcxf02_fcetbk02&pos=baike_pc_turbo_1767&ori_sid=00bb34716a7c54d6) | [中医治疗肠易激综合征](https://www.baidu.com/s?word=%E4%B8%AD%E5%8C%BB%E6%B2%BB%E7%96%97%E8%82%A0%E6%98%93%E6%BF%80%E7%BB%BC%E5%90%88%E5%BE%81&tn=SE_baikepcxf02_fcetbk02&pos=baike_pc_turbo_1767&ori_sid=00bb34716a7c54d6)  [肠易激综合征病因](https://www.baidu.com/s?word=%E8%82%A0%E6%98%93%E6%BF%80%E7%BB%BC%E5%90%88%E5%BE%81%E7%97%85%E5%9B%A0&tn=SE_baikepcxf02_fcetbk02&pos=baike_pc_turbo_1767&ori_sid=00bb34716a7c54d6) | [拉肚子拉水止泻小妙招](https://www.baidu.com/s?word=%E6%8B%89%E8%82%9A%E5%AD%90%E6%8B%89%E6%B0%B4%E6%AD%A2%E6%B3%BB%E5%B0%8F%E5%A6%99%E6%8B%9B&tn=SE_baikepcxf02_fcetbk02&pos=baike_pc_turbo_1767&ori_sid=00bb34716a7c54d6) [肠易激综合症怎么调理](https://www.baidu.com/s?word=%E8%82%A0%E6%98%93%E6%BF%80%E7%BB%BC%E5%90%88%E7%97%87%E6%80%8E%E4%B9%88%E8%B0%83%E7%90%86&tn=SE_baikepcxf02_fcetbk02&pos=baike_pc_turbo_1767&ori_sid=00bb34716a7c54d6) | | [肠应激性综合症](https://www.baidu.com/s?word=%E8%82%A0%E5%BA%94%E6%BF%80%E6%80%A7%E7%BB%BC%E5%90%88%E7%97%87&tn=SE_baikepcxf02_fcetbk02&pos=baike_pc_turbo_1767&ori_sid=00bb34716a7c54d6)  [肠易激综合症自愈方法](https://www.baidu.com/s?word=%E8%82%A0%E6%98%93%E6%BF%80%E7%BB%BC%E5%90%88%E7%97%87%E8%87%AA%E6%84%88%E6%96%B9%E6%B3%95&tn=SE_baikepcxf02_fcetbk02&pos=baike_pc_turbo_1767&ori_sid=00bb34716a7c54d6) | |
| --- | --- | --- | --- | --- | --- | --- |
| Q 新手上路 | | 邮 我有疑问 | | 目 投诉建议 | | |
| [成长任务](https://baike.baidu.com/usercenter/tasks#guide) [编辑入门](https://baike.baidu.com/help#main01) | | [内容质疑](javascript:void(0);) [在线客服](http://zhiqiu.baidu.com/baike/passport/html/baikechat.html) | | [举报不良信息](http://help.baidu.com/newadd?word=%E8%82%A0%E6%98%93%E6%BF%80%E7%BB%BC%E5%90%88%E5%BE%81&&submit_link=https%3A%2F%2Fbaike.baidu.com%2Fitem%2F%25E8%2582%25A0%25E6%2598%2593%25E6%25BF%2580%25E7%25BB%25BC%25E5%2590%2588%25E5%25BE%2581%2F8456761%3FfromModule%3Dlemma_inlink&prod_id=10&category=1) [未通过词条申诉](http://help.baidu.com/newadd?word=%E8%82%A0%E6%98%93%E6%BF%80%E7%BB%BC%E5%90%88%E5%BE%81&&submit_link=https%3A%2F%2Fbaike.baidu.com%2Fitem%2F%25E8%2582%25A0%25E6%2598%2593%25E6%25BF%2580%25E7%25BB%25BC%25E5%2590%2588%25E5%25BE%2581%2F8456761%3FfromModule%3Dlemma_inlink&prod_id=10&category=2) | | |
| [编辑规则](https://baike.baidu.com/help#main06) [本人编辑](https://baike.baidu.com/item/%E7%99%BE%E5%BA%A6%E7%99%BE%E7%A7%91%EF%BC%9A%E6%9C%AC%E4%BA%BA%E8%AF%8D%E6%9D%A1%E7%BC%96%E8%BE%91%E6%9C%8D%E5%8A%A1/22442459?bk_fr=pcFooter) | | [官方贴吧](http://tieba.baidu.com/f?ie=utf-8&fr=bks0000&kw=%E7%99%BE%E5%BA%A6%E7%99%BE%E7%A7%91) [意见反馈](javascript:void(0);) | | [投诉侵权信息](http://help.baidu.com/newadd?word=%E8%82%A0%E6%98%93%E6%BF%80%E7%BB%BC%E5%90%88%E5%BE%81&&submit_link=https%3A%2F%2Fbaike.baidu.com%2Fitem%2F%25E8%2582%25A0%25E6%2598%2593%25E6%25BF%2580%25E7%25BB%25BC%25E5%2590%2588%25E5%25BE%2581%2F8456761%3FfromModule%3Dlemma_inlink&prod_id=10&category=6) [封禁查询与解封](http://help.baidu.com/newadd?word=%E8%82%A0%E6%98%93%E6%BF%80%E7%BB%BC%E5%90%88%E5%BE%81&&submit_link=https%3A%2F%2Fbaike.baidu.com%2Fitem%2F%25E8%2582%25A0%25E6%2598%2593%25E6%25BF%2580%25E7%25BB%25BC%25E5%2590%2588%25E5%25BE%2581%2F8456761%3FfromModule%3Dlemma_inlink&prod_id=10&category=5) | |  |

<https://baike.baidu.com/item/>肠易激综合征/8456761?fromModule=lemma_inlink

3/4

2022/12/14 10:30

[女](javascript:void(0);) [疊](http://baike.baidu.com/l/WWoXYu7P) [口](javascript:void(0);)

©2022 Baidu 使用百度前必读 | 百科协议 | 隐私政策 | 百度百科合作平台 | 京ICP证030173号 [京公网安备11000002000001号](http://www.beian.gov.cn/portal/registerSystemInfo?recordcode=11000002000001)

肠易激综合征(肠易激综合征) _百度百科

[岔](https://baike.baidu.com/)

小 播报

[编辑](javascript:;)

[收藏](javascript:;)

[赞](javascript:;)

<https://baike.baidu.com/item/>肠易激综合征/8456761?fromModule=lemma_inlink

4/4
