# Supplementary material for: Assessment of medical information on irritable bowel syndrome information in Wikipedia and Baidu Encyclopedia: comparative study
Source: PeerJ. 2024 May 24;12:e17264. doi: 10.7717/peerj.17264 (PMC11129691; doi:10.7717/peerj.17264)
Supplement: Data S1 [file peerj-12-17264-s001.zip › σÄƒσoïμò░μì«/Baidu/Baidu-English/11-σèƒΦâ╜μÇoΦà╣ΦâÇ_τÖ╛σ║aτÖ╛τoæ.docx]

[疊口](javascript:void(0);)

| 功能性腹胀 | | \| [小播报](javascript:;) \| \| --- \| | \| [c编辑](javascript:;) \| \| --- \| | \| [上传视频](javascript:;) \| \| --- \| | . 收藏 [山 0](javascript:void(0);) 1 |  | |
| --- | --- | --- | --- | --- | --- | --- | --- | --- | --- | --- |
|  |  |  |  |  |  |  | 科普中国  致力于权威的科学传播 |
| 本词条由[“科普中国”科学百科词条编写与应用工作项目](https://baike.baidu.com/science) 认证 。  Functional bloating is a subjective sensation of repeated abdominal distention, unlike the feeling of fullness and discomfort that occurs after eating, with or without measurable increase in abdominal circumference, and is not part of other functional bowel diseases such as irritable bowel syndrome or functional gastroduodenal diseases such as functional dyspepsia. 10% ~ 30% of the general population can be affected by bloating, which is common in women and does not depend on age. Functional bloating is thought to be associated with obesity, aerophagia, polyphagia, diaphragmatic decline, protrusion of the spine, weakened abdominal muscle strength, and especially mental status, and it is an intermittent chronic process. | | | | | | 本词条认证专家为  韩英 丨主任医师  北京军区总医院 消化内科  审核 | |
|  |  |  |  |  |  |  | |
| 多发群体 女性 常见症状 腹胀，白天逐渐加重，尤其进食后，晚上减展…开 、 | | | | | |  |  |
| 常见发病 | 腹部 | 作者 | | | |  |  |

|  |
| --- |

[女](javascript:void(0);)

| 2022/12/14 10:51  [网页](https://www.baidu.com/) | [新闻](http://news.baidu.com/) | 功能性腹胀_百度百科  [贴吧](https://tieba.baidu.com/) [知道](https://zhidao.baidu.com/) [网盘](https://pan.baidu.com/?from=1027327l) [图片](http://image.baidu.com/) | [视频](http://v.baidu.com/) | [地图](http://map.baidu.com/) | [文库](https://wenku.baidu.com/) | 百科 | [百度首页](http://www.baidu.com/) [登录](javascript:;) |
| --- | --- | --- | --- | --- | --- | --- | --- |

| [岔](https://baike.baidu.com/) | \| 功能性腹胀 \| 进入词条 \| \| --- \| --- \| | \| 全站搜索 \| \| --- \| | [帮助](https://baike.baidu.com/help) | |
| --- | --- | --- | --- | --- | --- | --- | --- |
| 近期有不法分子冒充百度百科官方人员，以删除词条为由威胁并敲诈相关企业。在此严正声明：百度百科是免费编辑平台，绝不存在收费代编服务，请勿上当受骗！ [详情>>](https://baike.baidu.com/common/declaration) | | | | |
| [首页](https://baike.baidu.com/) 秒懂百科 特色百科 用户 知识专题 权威合作 | | [口下载百科APP](https://baike.baidu.com/wapui/subpage/baikeappdownload?sfrom=pc_lemmapage_navigation) | | [2 个](https://baike.baidu.com/usercenter) |

| \| 目录 \| 1 [病因](#_bookmark1)  2 [临床表现](#_bookmark2)  3 [诊断](#_bookmark3)  4 [鉴别诊断](#_bookmark4)  5 [治疗](#_bookmark5) \| \| --- \| --- \| | | | | | \|  \| \| --- \|   [“科普中国”科学百科词条编](http://www.kepuchina.cn/)  “科普中国”是为我国科普信 建设塑造的全...  权威合作编辑  [什么是权威编辑](http://baike.bdimg.com/cms/static/cooperation/content.pdf) |
| --- | --- | --- | --- | --- | --- | --- | --- | --- |
|  |  |  |  |  | 词条统计  浏览次数： 20067次  编辑次数： 2次[历史版本](https://baike.baidu.com/historylist/%E5%8A%9F%E8%83%BD%E6%80%A7%E8%85%B9%E8%83%80/22050389)  最近更新： [卫计委科普项目](https://baike.baidu.com/usercenter/userpage?uk=x54mmYyOgfn1hjDv9q0UTQ&from=lemma) ( 2017-11-2  突出贡献榜  [卫计委科普项目](https://baike.baidu.com/usercenter/userpage?uk=x54mmYyOgfn1hjDv9q0UTQ&from=lemma) |
|  | 基本信息  就诊科室  多发群体  常见发病部位 | 消化内科  女性  腹部 | 常见病因 常见症状  作 者 | 不明，可能与生理、心理因素有关  腹胀，白天逐渐加重，尤其进食后，晚上减轻；伴上 腹部疼痛、早饱和食物胃内滞留 |  |
| 病因 | | | [小 播报c编辑](javascript:;) | |  |
| At present, the pathophysiological mechanism of functional abdominal distension has not been fully elucidated, mainly related to physiological factors and psychopsychological factors. (1) Physiological factors include intestinal gas accumulation, abnormal sensorimotor function, food intolerance, fluid retention, weak abdominal wall muscles, etc.; (2) Mental and psychological factors include depression, insomnia, coping disorders, panic disorder, phobia, etc. | | | | |  |
| 临床表现 | | | [小 播报c编辑](javascript:;) | |  |
| The typical symptom is bloating, which gradually worsens during the day, especially after eating, and decreases at night. At the same time, it is accompanied by epigastric pain, early satiety and accumulation of food retention in the stomach. | | | | |  |
| 诊断 | | | [小 播报c编辑](javascript:;) | |  |
| Rome III diagnostic criteria for functional bloating are as follows: (1) recurrent feeling of distension or gross abdominal distension at least 3 days per month for 3 months; (2) insufficient evidence to diagnose functional dyspepsia, irritable bowel syndrome, or other functional gastrointestinal disorders. Pre-diagnosis symptoms have been present for at least 6 months and the above criteria have been met in the past 3 months. | | | | |  |

<https://baike.baidu.com/item/>功能性腹胀?fromModule=lemma_search-box

1/2

2022/12/14 10:51

[未通过词条申诉](http://help.baidu.com/newadd?word=%E5%8A%9F%E8%83%BD%E6%80%A7%E8%85%B9%E8%83%80&&submit_link=https%3A%2F%2Fbaike.baidu.com%2Fitem%2F%25E5%258A%259F%25E8%2583%25BD%25E6%2580%25A7%25E8%2585%25B9%25E8%2583%2580%3FfromModule%3Dlemma_search-box&prod_id=10&category=2)

[封禁查询与解封](http://help.baidu.com/newadd?word=%E5%8A%9F%E8%83%BD%E6%80%A7%E8%85%B9%E8%83%80&&submit_link=https%3A%2F%2Fbaike.baidu.com%2Fitem%2F%25E5%258A%259F%25E8%2583%25BD%25E6%2580%25A7%25E8%2585%25B9%25E8%2583%2580%3FfromModule%3Dlemma_search-box&prod_id=10&category=5)

功能性腹胀_百度百科

| 鉴别诊断  [小 播报c编辑](javascript:;)  [女 口](javascript:void(0);)  **1. Aerophagia**  **Patients often have nervousness, emotional instability or depression, the main symptoms of the digestive tract is belching or hiccups, patients feel comfortable after belching, in fact, swallowing a large amount of air at the same time as belching, so that the upper abdomen has a feeling of swelling or fullness. There were no obvious organic lesions on either a barium x-ray or gastroscopy.**  **2. Chronic atrophic gastritis**  **It is more common in middle-aged patients and above, and the main symptoms are dull epigastric pain, abdominal distension, loss of appetite, weight loss, anemia and other symptoms. Gastroscopy and mucosal biopsy histopathology can establish the diagnosis.**  **3. Stomach prosagging**  **It is more common in elongated, weak people, elderly people with loose abdominal walls, and people with maternal or chronic wasting diseases. Abdominal distension is generally mild when waking up in the morning, and the symptoms are aggravated when standing for too long to the evening and can also be accompanied by symptoms such as loss of appetite, nausea, belching, and weakness of the limbs. X-ray barium meal examination showed that the position of the stomach was significantly down, and the gastric contour was moved down below the iliac ridge connection on both sides, and the stomach was weak, which was conducive to the diagnosis of gastric prolapse.**  [小 播报c编辑](javascript:;)  治疗  At present, there are no effective prevention and treatment measures for this disease.  1. General treatment  Carry out health publicity and education for patients, remind patients to pay attention to regular exercise and weight reduction.  2. Adjust your diet  Avoid foods that produce gas, such as foods high in sugar, beans, or milk. If the symptoms worsen after eating daily food, fresh fruit or juice, it indicates lactose or fructose intolerance, and further examination or dietary exclusion tests are needed.  3. Medication  (1) Probiotics: ① adjust the imbalance of microecology and prevent diarrhea; ② Relieve lactose intolerance and promote nutrient absorption; ③ Metabolites can produce biological antagonism, resist bacterial virus infection, enhance human immunity, improve intestinal barrier function, relieve allergy; ④ Prevent and treat certain diseases, such as intestinal syndrome, respiratory infections, allergies, halitosis, gastric ulcers, etc.  (2) Gastrointestinal motility agents: gastrointestinal motility agents may have certain effects on some patients.  (3) Other drugs: such as pancreatic enzyme preparations, activated carbon (medicinal carbon), surface active substances, etc., may be effective.  内容来自  学术论文    ．年  [许卫华，王微，李妮矫，吕冉等. 加味三香汤治疗脾虚痰湿型功能性腹胀的临床观察．](https://xueshu.baidu.com/usercenter/paper/show?paperid=f0f40b5fd68eaaccf9e512e3c00c06be&tn=SE_baiduxueshu_c1gjeupa&ie=utf-8&site=baike) 《中华中医药杂志》， 2014  [张红英，王进海，李永等. 功能性腹胀发病机制的研究．](https://xueshu.baidu.com/usercenter/paper/show?paperid=88fe78d83cf1b232b63980fd172d1d18&tn=SE_baiduxueshu_c1gjeupa&ie=utf-8&site=baike) 《CNKI;WanFang》， 2013  [王峰，李小芳，周晓军. 大柴胡汤加减治疗功能性腹胀的效果．](https://xueshu.baidu.com/usercenter/paper/show?paperid=b7ab2264f510ed55e5ed55dec2ea31d8&tn=SE_baiduxueshu_c1gjeupa&ie=utf-8&site=baike) 《实用临床医药杂志》， 2015  [贾志新，冯五金等. 冯五金老中医温阳通调法治疗功能性腹胀经验体会．](https://xueshu.baidu.com/usercenter/paper/show?paperid=0ee83b9c1a54264f35af9ee2d1ad43d8&tn=SE_baiduxueshu_c1gjeupa&ie=utf-8&site=baike) 《世界中西医结合杂志》， 2015  [查看全部](https://xueshu.baidu.com/s?wd=intitle%3A%28%E5%8A%9F%E8%83%BD%E6%80%A7%E8%85%B9%E8%83%80%29&tn=SE_baiduxueshu_c1gjeupa&ie=utf-8&sc_from=pingtai6&site=baike) |
| --- |

| 岔 搜索发现  [肝病会引起腹胀吗](https://www.baidu.com/s?word=%E8%82%9D%E7%97%85%E4%BC%9A%E5%BC%95%E8%B5%B7%E8%85%B9%E8%83%80%E5%90%97&tn=SE_baikepcxf02_fcetbk02&pos=baike_pc_turbo_1767&ori_sid=00bb35b0b6a95974)  [肚子老是胀气怎么办](https://www.baidu.com/s?word=%E8%82%9A%E5%AD%90%E8%80%81%E6%98%AF%E8%83%80%E6%B0%94%E6%80%8E%E4%B9%88%E5%8A%9E&tn=SE_baikepcxf02_fcetbk02&pos=baike_pc_turbo_1767&ori_sid=00bb35b0b6a95974) [青岛搬家](https://www.baidu.com/s?word=%E9%9D%92%E5%B2%9B%E6%90%AC%E5%AE%B6&tn=SE_baikepcxf02_fcetbk02&pos=baike_pc_turbo_1767&ori_sid=00bb35b0b6a95974)  [英语该如何学习](https://www.baidu.com/s?word=%E8%8B%B1%E8%AF%AD%E8%AF%A5%E5%A6%82%E4%BD%95%E5%AD%A6%E4%B9%A0&tn=SE_baikepcxf02_fcetbk02&pos=baike_pc_turbo_1767&ori_sid=00bb35b0b6a95974) [米粉图片](https://www.baidu.com/s?word=%E7%B1%B3%E7%B2%89%E5%9B%BE%E7%89%87&tn=SE_baikepcxf02_fcetbk02&pos=baike_pc_turbo_1767&ori_sid=00bb35b0b6a95974)  [篆体字图片识别](https://www.baidu.com/s?word=%E7%AF%86%E4%BD%93%E5%AD%97%E5%9B%BE%E7%89%87%E8%AF%86%E5%88%AB&tn=SE_baikepcxf02_fcetbk02&pos=baike_pc_turbo_1767&ori_sid=00bb35b0b6a95974) [酒柜样式](https://www.baidu.com/s?word=%E9%85%92%E6%9F%9C%E6%A0%B7%E5%BC%8F&tn=SE_baikepcxf02_fcetbk02&pos=baike_pc_turbo_1767&ori_sid=00bb35b0b6a95974)  [怎么炒饭](https://www.baidu.com/s?word=%E6%80%8E%E4%B9%88%E7%82%92%E9%A5%AD&tn=SE_baikepcxf02_fcetbk02&pos=baike_pc_turbo_1767&ori_sid=00bb35b0b6a95974)  [二手包回收](https://www.baidu.com/s?word=%E4%BA%8C%E6%89%8B%E5%8C%85%E5%9B%9E%E6%94%B6&tn=SE_baikepcxf02_fcetbk02&pos=baike_pc_turbo_1767&ori_sid=00bb35b0b6a95974) |
| --- |

Q

新手上路 [成长任务](https://baike.baidu.com/usercenter/tasks#guide)

[编辑规则](https://baike.baidu.com/help#main06)

[编辑入门](https://baike.baidu.com/help#main01)

[本人编辑](https://baike.baidu.com/item/%E7%99%BE%E5%BA%A6%E7%99%BE%E7%A7%91%EF%BC%9A%E6%9C%AC%E4%BA%BA%E8%AF%8D%E6%9D%A1%E7%BC%96%E8%BE%91%E6%9C%8D%E5%8A%A1/22442459?bk_fr=pcFooter)

我有疑问 [内容质疑](javascript:void(0);) [官方贴吧](http://tieba.baidu.com/f?ie=utf-8&fr=bks0000&kw=%E7%99%BE%E5%BA%A6%E7%99%BE%E7%A7%91)

投诉建议

[举报不良信息](http://help.baidu.com/newadd?word=%E5%8A%9F%E8%83%BD%E6%80%A7%E8%85%B9%E8%83%80&&submit_link=https%3A%2F%2Fbaike.baidu.com%2Fitem%2F%25E5%258A%259F%25E8%2583%25BD%25E6%2580%25A7%25E8%2585%25B9%25E8%2583%2580%3FfromModule%3Dlemma_search-box&prod_id=10&category=1)

[在线客服](http://zhiqiu.baidu.com/baike/passport/html/baikechat.html) [意见反馈](javascript:void(0);)

[投诉侵权信息](http://help.baidu.com/newadd?word=%E5%8A%9F%E8%83%BD%E6%80%A7%E8%85%B9%E8%83%80&&submit_link=https%3A%2F%2Fbaike.baidu.com%2Fitem%2F%25E5%258A%259F%25E8%2583%25BD%25E6%2580%25A7%25E8%2585%25B9%25E8%2583%2580%3FfromModule%3Dlemma_search-box&prod_id=10&category=6)

©2022 Baidu [使用百度前必读](http://www.baidu.com/duty/) | [百科协议](http://help.baidu.com/question?prod_en=baike&class=89&id=1637) | [隐私政策](http://help.baidu.com/question?prod_id=10&class=690&id=1001779) | [百度百科合作平台](https://baike.baidu.com/operation/cooperation) | 京ICP证030173号

[京公网安备11000002000001号](http://www.beian.gov.cn/portal/registerSystemInfo?recordcode=11000002000001)

<https://baike.baidu.com/item/>功能性腹胀?fromModule=lemma_search-box

2/2
