# Supplementary material for: Assessment of medical information on irritable bowel syndrome information in Wikipedia and Baidu Encyclopedia: comparative study
Source: PeerJ. 2024 May 24;12:e17264. doi: 10.7717/peerj.17264 (PMC11129691; doi:10.7717/peerj.17264)
Supplement: Data S1 [file peerj-12-17264-s001.zip › σÄƒσoïμò░μì«/Baidu/Baidu-English/5-μ┐ÇΦ║üμÇoσñoΦéáτùçσÇÖτ╛ñ_τÖ╛σ║aτÖ╛τoæ.docx]

| 2022/12/14 10:41 | 激躁性大肠症候群_百度百科  **[网页](https://www.baidu.com/)** **[新闻](http://news.baidu.com/)** **[贴吧](https://tieba.baidu.com/)** **[知道](https://zhidao.baidu.com/)** **[网盘](https://pan.baidu.com/?from=1027327l)** **[图片](http://image.baidu.com/)** | **[视频](http://v.baidu.com/)** | **[地图](http://map.baidu.com/)** | **[文库](https://wenku.baidu.com/)** | **百科** | [百度首页](http://www.baidu.com/) [登录](javascript:;) |
| --- | --- | --- | --- | --- | --- | --- |

| [岔](https://baike.baidu.com/) | \| 激躁性大肠症候群 \| 进入词条 \| \| --- \| --- \| | \| 全站搜索 \| \| --- \| | [帮助](https://baike.baidu.com/help) |
| --- | --- | --- | --- | --- | --- | --- |
| 近期有不法分子冒充百度百科官方人员，以删除词条为由威胁并敲诈相关企业。在此严正声明：百度百科是免费编辑平台，绝不存在收费代编服务，请勿上当受骗！ [详情>>](https://baike.baidu.com/common/declaration) | | | |
| [首页](https://baike.baidu.com/) 秒懂百科 特色百科 用户 知识专题 权威合作 [口下载百科APP](https://baike.baidu.com/wapui/subpage/baikeappdownload?sfrom=pc_lemmapage_navigation) [2 个](https://baike.baidu.com/usercenter) | | | |

|  | . 收藏 [山 158](javascript:void(0);)  **性** **类**  **质** **别**  激躁性大肠症候群 [上传视频](javascript:;)   \| 小播报 \| \| --- \|  \| 编辑 \| \| --- \|  \| O讨论 \| \| --- \|   肠胃道疾病  Irritable Bowel Syndrome is the most common gastrointestinal disease in the clinic, although the disease is not fatal, but the patient often has great pain, and the doctor has a feeling of helplessness and frustration during treatment.  Irritable coli disease is common in young or middle-aged people, with a female/male ratio of 1:2. The main manifestations are chronic constipation, intermittent diarrhea, or both.  激躁性大肠症候群  医学  疾病  **中文名**  **外文名**  Irritable Bowel Syndrome | 13 | [激躁性大肠症候群的概述 张)](https://baike.baidu.com/pic/%E6%BF%80%E8%BA%81%E6%80%A7%E5%A4%A7%E8%82%A0%E7%97%87%E5%80%99%E7%BE%A4/4549268/1/0b55b319ebc4b74570b842efc5fc1e178a82151e?fr=lemma&fromModule=lemma_top-image&ct=single) |
| --- | --- | --- | --- | --- | --- | --- |
|  | \| 目录 \| 3 [病理生理](#_bookmark2)  1 [疾病类型](#_bookmark1) 2 [临床表现](#_bookmark4)  5 [治疗方式](#_bookmark5) 6 [预防保养](#_bookmark6)  4 [诊断要点](#_bookmark3) \| \| --- \| --- \| |  |  |
|  |  |  | \| **词条统计**    浏览次数： 247543次  编辑次数： 17次历史版本  最近更新： w_ou ( 2021-01-26)  **突出贡献榜**  qqq582311436  [女](javascript:void(0);) \| \| \| \| --- \| --- \| --- \| \| **1** \| [购买域名](https://cpro.baidu.com/cpro/ui/uijs.php?en=mywWUA71T1YsFh7sT7qGujYsFhPC5H0huAbqrauGTdq9TZ0qnauJp1YvrjI-mhcLuhwhujb4nyRsFh_qFRc4FRFKFRnzFRmzFRf1FRmzFRn1FRuaFhkdpvbqniuVmLKV5HbYnW0LFMDqn1TsrHfkPjKxmLKzFMFB5H0hTMnqniu1uyk_ugFxpyfqniu1pyfquH04nyf4uAmLuhFBPvRvrau1IA-b5H6hIjdYTAP_pyPouyf1gv9WFMwb5HD4rHTvn1nhIAYqnWm3rj01PH6kFMwVT1YkPWTsrH6dPW0vFMwd5gRkrHbLPWn1FMRqpZwYTZn-nYD-nbm-nbuBmy-ouiRzwyF9pywdFHF7mvqVFMmqnBuG5yn3m1FBnAPh&besl=6&c=news&cf=1&cvrq=1941531&eid_list=201577_202016_204226_204854_205526_207574_209357&expid=201577_202016_202257_202564_204854_205526_205809_207574_209394&fr=20&fv=0&haacp=1286&img_typ=0&itm=0&lu_idc=gzhxy&lukid=1&lus=e091d9df7fbb7e68&lust=63993786&luwtr=17492749210616066771&mscf=0&n=10&nttp=1&p=baidu&pbs=220093&sce=7&sr=72&ssp2=1&tpl=baiduCustITagLinkUnitRankCol&tsf=dtp:1&tu_type=0&u=%2Fitem%2F%25E6%25BF%2580%25E8%25BA%2581%25E6%2580%25A7%25E5%25A4%25A7%25E8%2582%25A0%25E7%2597%2587%25E5%2580%2599%25E7%25BE%25A4%2F4549268%3FfromModule%3Dsearch%2Dresult%5Flemma&uicf=lurecv&urlid=0&eot=1) \| **12** 电商台怎 \| \| **2** \| [37游戏平台](https://cpro.baidu.com/cpro/ui/uijs.php?en=mywWUA71T1YsFh7sT7qGujYsFhPC5H0huAbqrauGTdq9TZ0qnauJp1YvrjI-mhcLuhwhujb4nyRsFh_qn1T-wjn-fYR-fYm-fWT-f1m-fbf-fYn-fH6hUZNopHYzFhdWTAYqrHc1nj0hTHY1P104PjDYn7qWTZchThcqnauzT1YkFMP-UAk-T-qGujYkFMPGujd-njbkuj-buWIhmhcLuHm3FMPYpyfqrauY5gwsmvkGmvV-ujPxpAnhIAfqnHb4P1m1nzuYUHYzPW63njndrjDhIAd15HDvP104rjRvnjmhIZRqIHD4rHTvn1nhIHdCIZwsTzR1fiRzwBRzwhF9pyV-FHF7mh7GuZR-nbNWUvYhIWYzFhbqmHfzuWTzrjn&besl=6&c=news&cf=1&cvrq=3623810&eid_list=201577_202016_204226_204854_205526_207574_209357&expid=201577_202016_202257_202564_204854_205526_205809_207574_209394&fr=20&fv=0&haacp=188&img_typ=0&itm=0&lu_idc=gzhxy&lukid=2&lus=e091d9df7fbb7e68&lust=63993786&luwtr=676781759077005289&mscf=0&n=10&nttp=1&p=baidu&pbs=220093&sce=7&sr=72&ssp2=1&tpl=baiduCustITagLinkUnitRankCol&tsf=dtp:1&tu_type=0&u=%2Fitem%2F%25E6%25BF%2580%25E8%25BA%2581%25E6%2580%25A7%25E5%25A4%25A7%25E8%2582%25A0%25E7%2597%2587%25E5%2580%2599%25E7%25BE%25A4%2F4549268%3FfromModule%3Dsearch%2Dresult%5Flemma&uicf=lurecv&urlid=0&eot=1) \| **13** sci论文投稿 \| \| **3**  **4**  **5** \| [国际期货](https://cpro.baidu.com/cpro/ui/uijs.php?en=mywWUA71T1YsFh7sT7qGujYsFhPC5H0huAbqrauGTdq9TZ0qnauJp1YvrjI-mhcLuhwhujb4nyRsFh_qFRc4FRuKFRFjFRPKFRnvFRwKFRFaFRmdFhkdpvbqnzuVmLKV5HDsrjb1Pzuk5HnLnjbYnHfsgvPsTBuzmWYsFMF15HDhTvN_UANzgv-b5HDhTv-b5yRsrH7brywhPvuBmWI-PW6hTLwGujY3FMfqIZKWUA-WpvNbndqCmzuYujYkrHbLPWn1FMwV5Hcvrj6sn1R3niuYUgnqnHmLnjb3PHmsPBuYIHddnHb4P1m1nzud5y9YIZK1FHPKFHFAFHFAmh7GpvR-nbNBmy-bIiRzwyPEUiuv5HchpHYkn1bkmWmdrf&besl=6&c=news&cf=1&cvrq=1621391&eid_list=201577_202016_204226_204854_205526_207574_209357&expid=201577_202016_202257_202564_204854_205526_205809_207574_209394&fr=20&fv=0&haacp=2073&img_typ=0&itm=0&lu_idc=gzhxy&lukid=3&lus=e091d9df7fbb7e68&lust=63993786&luwtr=14608826967881669318&mscf=0&n=10&nttp=1&p=baidu&pbs=220093&sce=7&sr=72&ssp2=1&tpl=baiduCustITagLinkUnitRankCol&tsf=dtp:1&tu_type=0&u=%2Fitem%2F%25E6%25BF%2580%25E8%25BA%2581%25E6%2580%25A7%25E5%25A4%25A7%25E8%2582%25A0%25E7%2597%2587%25E5%2580%2599%25E7%25BE%25A4%2F4549268%3FfromModule%3Dsearch%2Dresult%5Flemma&uicf=lurecv&urlid=0&eot=1) [csgo电脑配置](https://cpro.baidu.com/cpro/ui/uijs.php?en=mywWUA71T1YsFh7sT7qGujYsFhPC5H0huAbqrauGTdq9TZ0qnauJp1YvrjI-mhcLuhwhujb4nyRsFh_qmLPMUzNaPiN7PzNjPaNDPaNjPiN7PaNDPBNjnzu_IyVG5HfhUyPsUHYLrjcsniuk5HnLnjbYnHfsgvPsTBuzmWYsFMF15HDhTvN_UANzgv-b5HDhTv-b5yRsrH7brywhPvuBmWI-PW6hTLwGujY3FMfqIZKWUA-WpvNbndqCmzuYujYkrHbLPWn1FMwV5Hcvrj6sn1R3niuYUgnqnHmLnjb3PHmsPBuYIHddnHb4P1m1nzud5y9YIZK1FHPKFHFAFHFAmh7GpvR-nbNBmy-bIiRzwyPEUiuv5HchpHdBnWfsPW6kns&besl=6&c=news&cf=1&cvrq=3472465&eid_list=201577_202016_204226_204854_205526_207574_209357&expid=201577_202016_202257_202564_204854_205526_205809_207574_209394&fr=20&fv=0&haacp=611&img_typ=0&itm=0&lu_idc=gzhxy&lukid=4&lus=e091d9df7fbb7e68&lust=63993786&luwtr=2357911270216075011&mscf=0&n=10&nttp=1&p=baidu&pbs=220093&sce=7&sr=72&ssp2=1&tpl=baiduCustITagLinkUnitRankCol&tsf=dtp:1&tu_type=0&u=%2Fitem%2F%25E6%25BF%2580%25E8%25BA%2581%25E6%2580%25A7%25E5%25A4%25A7%25E8%2582%25A0%25E7%2597%2587%25E5%2580%2599%25E7%25BE%25A4%2F4549268%3FfromModule%3Dsearch%2Dresult%5Flemma&uicf=lurecv&urlid=0&eot=1) [战队logo设计](https://cpro.baidu.com/cpro/ui/uijs.php?en=mywWUA71T1YsFh7sT7qGujYsFhPC5H0huAbqrauGTdq9TZ0qnauJp1YvrjI-mhcLuhwhujb4nyRsFh_qFRfdFRFDFRcvFRf1UAqMUzNjriN7raNafzNjPBu_IyVG5HRhUyPsUHY4PHc1Pauk5HnLnjbYnHfsgvPsTBuzmWYsFMF15HDhTvN_UANzgv-b5HDhTv-b5yRsrH7brywhPvuBmWI-PW6hTLwGujY3FMfqIZKWUA-WpvNbndqCmzuYujYkrHbLPWn1FMwV5Hcvrj6sn1R3niuYUgnqnHmLnjb3PHmsPBuYIHddnHb4P1m1nzud5y9YIZK1FHPKFHFAFHFAmh7GpvR-nbNBmy-bIiRzwyPEUiuv5HchpHY3mWR3ujPBn6&besl=6&c=news&cf=1&cvrq=1756705&eid_list=201577_202016_204226_204854_205526_207574_209357&expid=201577_202016_202257_202564_204854_205526_205809_207574_209394&fr=20&fv=0&haacp=707&img_typ=0&itm=0&lu_idc=gzhxy&lukid=5&lus=e091d9df7fbb7e68&lust=63993786&luwtr=1863292750894598650&mscf=0&n=10&nttp=1&p=baidu&pbs=220093&sce=7&sr=72&ssp2=1&tpl=baiduCustITagLinkUnitRankCol&tsf=dtp:1&tu_type=0&u=%2Fitem%2F%25E6%25BF%2580%25E8%25BA%2581%25E6%2580%25A7%25E5%25A4%25A7%25E8%2582%25A0%25E7%2597%2587%25E5%2580%2599%25E7%25BE%25A4%2F4549268%3FfromModule%3Dsearch%2Dresult%5Flemma&uicf=lurecv&urlid=0&eot=1) \| **16** 网络工口程师  **14** 虚拟[货疊币](javascript:void(0);)平  **15** 图书批发网 \| \| **6** \| [自己创建个网](https://cpro.baidu.com/cpro/ui/uijs.php?en=mywWUA71T1YsFh7sT7qGujYsFhPC5H0huAbqrauGTdq9TZ0qnauJp1YvrjI-mhcLuhwhujb4nyRsFh_qFRfLFRfYFRFjFRFKFRcYFRcYFRFDFRD3FRc3FRmvFRPDFRm3FRfdFRF7FhkdpvbqPBuVmLKV5HDzn1cYnzuk5HnLnjbYnHfsgvPsTBuzmWYsFMF15HDhTvN_UANzgv-b5HDhTv-b5yRsrH7brywhPvuBmWI-PW6hTLwGujY3FMfqIZKWUA-WpvNbndqCmzuYujYkrHbLPWn1FMwV5Hcvrj6sn1R3niuYUgnqnHmLnjb3PHmsPBuYIHddnHb4P1m1nzud5y9YIZK1FHPKFHFAFHFAmh7GpvR-nbNBmy-bIiRzwyPEUiuv5HchpHd9Pj6snjTkms&besl=6&c=news&cf=1&cvrq=2235876&eid_list=201577_202016_204226_204854_205526_207574_209357&expid=201577_202016_202257_202564_204854_205526_205809_207574_209394&fr=20&fv=0&haacp=1001&img_typ=0&itm=0&lu_idc=gzhxy&lukid=6&lus=e091d9df7fbb7e68&lust=63993786&luwtr=6556233252601032990&mscf=0&n=10&nttp=1&p=baidu&pbs=220093&sce=7&sr=72&ssp2=1&tpl=baiduCustITagLinkUnitRankCol&tsf=dtp:1&tu_type=0&u=%2Fitem%2F%25E6%25BF%2580%25E8%25BA%2581%25E6%2580%25A7%25E5%25A4%25A7%25E8%2582%25A0%25E7%2597%2587%25E5%2580%2599%25E7%25BE%25A4%2F4549268%3FfromModule%3Dsearch%2Dresult%5Flemma&uicf=lurecv&urlid=0&eot=1) \| **17** 出版社自费 \| \| **7** \| [游戏盒子](https://cpro.baidu.com/cpro/ui/uijs.php?en=mywWUA71T1YsFh7sT7qGujYsFhPC5H0huAbqrauGTdq9TZ0qnauJp1YvrjI-mhcLuhwhujb4nyRsFh_qFRf1FRP7FRPAFRcLFRFKFRfsFRfLFRf1FhkdpvbqPzuVmLKV5HTzrH0dFMDqn1TsrHfkPjKxmLKzFMFB5H0hTMnqniu1uyk_ugFxpyfqniu1pyfquH04nyf4uAmLuhFBPvRvrau1IA-b5H6hIjdYTAP_pyPouyf1gv9WFMwb5HD4rHTvn1nhIAYqnWm3rj01PH6kFMwVT1YkPWTsrH6dPW0vFMwd5gRkrHbLPWn1FMRqpZwYTZn-nYD-nbm-nbuBmy-ouiRzwyF9pywdFHF7mvqVFMmqnBuG5H6dnyRvnHRL&besl=6&c=news&cf=1&cvrq=3228426&eid_list=201577_202016_204226_204854_205526_207574_209357&expid=201577_202016_202257_202564_204854_205526_205809_207574_209394&fr=20&fv=0&haacp=271&img_typ=0&itm=0&lu_idc=gzhxy&lukid=7&lus=e091d9df7fbb7e68&lust=63993786&luwtr=13245032438307475642&mscf=0&n=10&nttp=1&p=baidu&pbs=220093&sce=7&sr=72&ssp2=1&tpl=baiduCustITagLinkUnitRankCol&tsf=dtp:1&tu_type=0&u=%2Fitem%2F%25E6%25BF%2580%25E8%25BA%2581%25E6%2580%25A7%25E5%25A4%25A7%25E8%2582%25A0%25E7%2597%2587%25E5%2580%2599%25E7%25BE%25A4%2F4549268%3FfromModule%3Dsearch%2Dresult%5Flemma&uicf=lurecv&urlid=0&eot=1) \| **18** vr消防演练 \| \| **8** \| [哈佛大学申请](https://cpro.baidu.com/cpro/ui/uijs.php?en=mywWUA71T1YsFh7sT7qGujYsFhPC5H0huAbqrauGTdq9TZ0qnauJp1YvrjI-mhcLuhwhujb4nyRsFh_qFRc4FRu7FRcLFRmsFRcYFRm1FRfkFRDLFRn4FRNKFRnLFRNaFRPjFRmdFRFjFRu7FhkdpvbqrauVmLKV5HbknjDvFMDqn1TsrHfkPjKxmLKzFMFB5H0hTMnqniu1uyk_ugFxpyfqniu1pyfquH04nyf4uAmLuhFBPvRvrau1IA-b5H6hIjdYTAP_pyPouyf1gv9WFMwb5HD4rHTvn1nhIAYqnWm3rj01PH6kFMwVT1YkPWTsrH6dPW0vFMwd5gRkrHbLPWn1FMRqpZwYTZn-nYD-nbm-nbuBmy-ouiRzwyF9pywdFHF7mvqVFMmqnBuG5HDsPhn3nym1&besl=6&c=news&cf=1&cvrq=2024363&eid_list=201577_202016_204226_204854_205526_207574_209357&expid=201577_202016_202257_202564_204854_205526_205809_207574_209394&fr=20&fv=0&haacp=870&img_typ=0&itm=0&lu_idc=gzhxy&lukid=8&lus=e091d9df7fbb7e68&lust=63993786&luwtr=7841139350953723636&mscf=0&n=10&nttp=1&p=baidu&pbs=220093&sce=7&sr=72&ssp2=1&tpl=baiduCustITagLinkUnitRankCol&tsf=dtp:1&tu_type=0&u=%2Fitem%2F%25E6%25BF%2580%25E8%25BA%2581%25E6%2580%25A7%25E5%25A4%25A7%25E8%2582%25A0%25E7%2597%2587%25E5%2580%2599%25E7%25BE%25A4%2F4549268%3FfromModule%3Dsearch%2Dresult%5Flemma&uicf=lurecv&urlid=0&eot=1) \| **19** 价格便宜的 \| \| **9** \| [无人机反制](https://cpro.baidu.com/cpro/ui/uijs.php?en=mywWUA71T1YsFh7sT7qGujYsFhPC5H0huAbqrauGTdq9TZ0qnauJp1YvrjI-mhcLuhwhujb4nyRsFh_qFRP7FRw7FRn3FRPaFRFaFRuKFRcLFRcYFRfvFRnvFhkdpvbqriuVmLKV5H6sP1bvFMDqn1TsrHfkPjKxmLKzFMFB5H0hTMnqniu1uyk_ugFxpyfqniu1pyfquH04nyf4uAmLuhFBPvRvrau1IA-b5H6hIjdYTAP_pyPouyf1gv9WFMwb5HD4rHTvn1nhIAYqnWm3rj01PH6kFMwVT1YkPWTsrH6dPW0vFMwd5gRkrHbLPWn1FMRqpZwYTZn-nYD-nbm-nbuBmy-ouiRzwyF9pywdFHF7mvqVFMmqnBuG5yczm1bvPyR3&besl=6&c=news&cf=1&cvrq=1415934&eid_list=201577_202016_204226_204854_205526_207574_209357&expid=201577_202016_202257_202564_204854_205526_205809_207574_209394&fr=20&fv=0&haacp=904&img_typ=0&itm=0&lu_idc=gzhxy&lukid=9&lus=e091d9df7fbb7e68&lust=63993786&luwtr=685006414410405008&mscf=0&n=10&nttp=1&p=baidu&pbs=220093&sce=7&sr=72&ssp2=1&tpl=baiduCustITagLinkUnitRankCol&tsf=dtp:1&tu_type=0&u=%2Fitem%2F%25E6%25BF%2580%25E8%25BA%2581%25E6%2580%25A7%25E5%25A4%25A7%25E8%2582%25A0%25E7%2597%2587%25E5%2580%2599%25E7%25BE%25A4%2F4549268%3FfromModule%3Dsearch%2Dresult%5Flemma&uicf=lurecv&urlid=0&eot=1) \| **20** csgo网站开 \| \| **10** \| [网络安全培训](https://cpro.baidu.com/cpro/ui/uijs.php?en=mywWUA71T1YsFh7sT7qGujYsFhPC5H0huAbqrauGTdq9TZ0qnauJp1YvrjI-mhcLuhwhujb4nyRsFh_qFRPDFRm3FRnzFRRLFRcsFRczFRn3FR7aFRndFRRsFRfkFRcdFhkdpvbqnH0hUyPsUHYknW0vP1DhTHY1P104PjDYn7qWTZchThcqnauzT1YkFMP-UAk-T-qGujYkFMPGujd-njbkuj-buWIhmhcLuHm3FMPYpyfqrauY5gwsmvkGmvV-ujPxpAnhIAfqnHb4P1m1nzuYUHYzPW63njndrjDhIAd15HDvP104rjRvnjmhIZRqIHD4rHTvn1nhIHdCIZwsTzR1fiRzwBRzwhF9pyV-FHF7mh7GuZR-nbNWUvYhIWYzFhbqPAckP179PHc&besl=6&c=news&cf=1&cvrq=3292150&eid_list=201577_202016_204226_204854_205526_207574_209357&expid=201577_202016_202257_202564_204854_205526_205809_207574_209394&fr=20&fv=0&haacp=1162&img_typ=0&itm=0&lu_idc=gzhxy&lukid=10&lus=e091d9df7fbb7e68&lust=63993786&luwtr=2249689388344567100&mscf=0&n=10&nttp=1&p=baidu&pbs=220093&sce=7&sr=72&ssp2=1&tpl=baiduCustITagLinkUnitRankCol&tsf=dtp:1&tu_type=0&u=%2Fitem%2F%25E6%25BF%2580%25E8%25BA%2581%25E6%2580%25A7%25E5%25A4%25A7%25E8%2582%25A0%25E7%2597%2587%25E5%2580%2599%25E7%25BE%25A4%2F4549268%3FfromModule%3Dsearch%2Dresult%5Flemma&uicf=lurecv&urlid=0&eot=1) \| **21** 怎么创建小 \| \| **11** \| [亚马逊图书](https://cpro.baidu.com/cpro/ui/uijs.php?en=mywWUA71T1YsFh7sT7qGujYsFhPC5H0huAbqrauGTdq9TZ0qnauJp1YvrjI-mhcLuhwhujb4nyRsFh_qFRfkFRnLFRnzFRNDFRfkFRcLFRPDFRFjFRPKFRR4FhkdpvbqnHDhUyPsUHY3nj0dPiuk5HnLnjbYnHfsgvPsTBuzmWYsFMF15HDhTvN_UANzgv-b5HDhTv-b5yRsrH7brywhPvuBmWI-PW6hTLwGujY3FMfqIZKWUA-WpvNbndqCmzuYujYkrHbLPWn1FMwV5Hcvrj6sn1R3niuYUgnqnHmLnjb3PHmsPBuYIHddnHb4P1m1nzud5y9YIZK1FHPKFHFAFHFAmh7GpvR-nbNBmy-bIiRzwyPEUiuv5HchpHYvuHFBm1T3n0&besl=6&c=news&cf=1&cvrq=3335536&eid_list=201577_202016_204226_204854_205526_207574_209357&expid=201577_202016_202257_202564_204854_205526_205809_207574_209394&fr=20&fv=0&haacp=219&img_typ=0&itm=0&lu_idc=gzhxy&lukid=11&lus=e091d9df7fbb7e68&lust=63993786&luwtr=718744168689952326&mscf=0&n=10&nttp=1&p=baidu&pbs=220093&sce=7&sr=72&ssp2=1&tpl=baiduCustITagLinkUnitRankCol&tsf=dtp:1&tu_type=0&u=%2Fitem%2F%25E6%25BF%2580%25E8%25BA%2581%25E6%2580%25A7%25E5%25A4%25A7%25E8%2582%25A0%25E7%2597%2587%25E5%2580%2599%25E7%25BE%25A4%2F4549268%3FfromModule%3Dsearch%2Dresult%5Flemma&uicf=lurecv&urlid=0&eot=1) \| **22** 供应链管理 \| |
|  | 疾病类型  [小 播报编辑](javascript:;)  There are three types of irritable coli disease:  (1) Chronic painless intermittent diarrhea: Many people have gastrointestinal problems, such as diarrhea, constipation, flatulence, etc., but cannot find out the cause.  (2) Chronic intermittent abdominal pain and constipation: Patients with spastic colitis complain of chronic abdominal pain and constipation.  (3) Diarrhea and constipation alternately: Some patients can have the above two manifestations, constipation and diarrhea alternately.  临床表现  [小 播报编辑](javascript:;) |  |  |
|  | Typical patients may have intermittent watery diarrhea for years or months, often worsening in the early morning or after breakfast, and feeling normal the rest of the time after excreting 3-4 loose stools with large amounts of mucus. Diarrhea all day, or always at night, is rare. Diarrhea can last for weeks or months and then disappear automatically for a while. Some patients excrete "pencil-like" mushy stools without diarrhea. Another typical presentation is chronic abdominal pain with constipation, or constipation alternating with diarrhea. These patients complain of intermittent lower abdominal cramps, which are relieved by exhaust or defecation. Irritable colorectal disease also often has heartburn, obvious bloating, back pain, weakness, fatigue, palpitations, etc. |  |  |
|  | 病理生理  [小 播报编辑](javascript:;) |  |  |
|  | The basic pathophysiological abnormality in irritable bowel disease is altered bowel motility. Patients with colonic spasm (abdominal pain and constipation) have increased colonic motility at rest.  In contrast, patients with predominantly diarrhea had decreased colonic motility at rest. Some patients with irritable bowel disease may have evidence of significant psychosis. Common depression, hysteria, obsessive-compulsive disorder.  Mental stimulation can also make symptoms worse. However, it should be noted that the pressure in the colon can increase in normal people during acute stress reactions.  This suggests that mental stimulation may be a nonspecific trigger of irritable bowel disease, as in other diseases, with many etiologies. |  |  |
|  |  |  |  |
|  | 诊断要点  [小 播报编辑](javascript:;) |  |  |
|  | 1. Have the above symptoms of gastrointestinal dysfunction and accompanied by systemic symptoms. 2. The patient's symptoms often change with mood. 3. Total gastrointestinal imaging or gastroscopy, rectoscopy are normal to exclude organic lesions. 3. Etiology and pathogenesis: 1. Feeling external evil: Among the six immoralities, cold, dampness, heat, and heat are common, and there are especially many people who feel dampness and evil and cause diarrhea.  2. Injuries caused by diet: mostly excessive diet, overnight food stop; or not fat and sweet, dull stomach and spleen; Or cold and unclean, hurting the spleen and stomach.  3. Emotional disorders: liver depression multiplies the spleen 4. Body weakness and long-term disease: spleen deficiency and kidney |  |  |
|  | 治疗方式  [小 播报编辑](javascript:;) |  |  |

<https://baike.baidu.com/item/>激躁性大肠症候群/4549268?fromModule=search-result_lemma 1/2

2022/12/14 10:41

[口](javascript:void(0);)

Cold and dampness are the main pathological factors of diarrhea, and spleen deficiency and dampness are the key to its pathogenesis. Solid treatment should be based on the principle of transporting the spleen and removing dampness. Dialectical governance

1. Cold and wet spleen: disperse cold and dampness, strengthen the spleen and stop diarrhea

2. Damp heat bet: clear heat and dampness, thick intestine and stop diarrhea

3. Food stagnation: eliminate food stagnation, harmonize the spleen and stomach

4. Liver qi multiplies the spleen: suppresses the liver and supports the spleen, harmonizes the spleen and stomach

5. Spleen deficiency and dampness: strengthen the spleen and invigorate qi, luck stops diarrhea

6. Spleen and kidney yang deficiency: warm spleen and kidney, solid intestine to stop diarrhea

预防保养

1. Pay attention to food hygiene and live regularly

2. Do not eat raw and cold, or fat and greasy, or drink excessively

3. Pay attention to emotional factors

4. Pay attention to keeping warm and do not get wet and cold

[词条图册 更多图册 >](https://baike.baidu.com/pic/%E6%BF%80%E8%BA%81%E6%80%A7%E5%A4%A7%E8%82%A0%E7%97%87%E5%80%99%E7%BE%A4/4549268?fr=lemma)

[概述图册(1)](https://baike.baidu.com/pic/%E6%BF%80%E8%BA%81%E6%80%A7%E5%A4%A7%E8%82%A0%E7%97%87%E5%80%99%E7%BE%A4/4549268/1/0b55b319ebc4b74570b842efc5fc1e178a82151e?fr=lemma)

[女](javascript:void(0);)

[小 播报编辑](javascript:;)

激躁性大肠症候群_百度百科

| 猜你喜欢    [躁郁自测--抑郁自评量表SDS，标准版](http://www.baidu.com/baidu.php?url=Ks00000EAMrnlPLIyCNSmyXz9UGTU52obwY0vDioEMchaZhpNgYYofrKouLvcTqto0hdwElFKg6Iv-oyLybb3CA9pGUy9OdhJ5mFJfWFJbzMFvrZabGsfmVVjXuet92gcBQVas355TwhaU0ZSy2M2gpeb7htKt3pj6losOyuKSs19s0ze-hysXcjXvATQ-OBUJ5nT0dRNCBD-JM7CdFnktr_Jh_3.7Y_NR2Ar5Od66E89WtJPgKwzl526eZKfHZf68Hfuxwe3h2SMowJ32rgwuuuY4PlhGv-5QWdQjPakYeVdB6.U1Yk0ZDqE2HRlVjRV5r1GVpB8EQc86KY5IHJYOHgdQQB0A-V5HDzPWc0Iybq0ZKGujYzn0KWpyfqP1c0mhbqn10k0AuY5H00TA6qn0KET1Ys0AFL5H00UMfqn0K1XWY0ThIYmyTqn0K8IM0qna3snj0snj0sn0K-ThTqn0KYTh7buHYs0AFbpyfqnW77fbc4nYu7wWFDnRwArj97fYfYrD7DrRwKPDnvnbD0uAPWujY0mgPxpywW5gK1QyIlpZ940AqW5HD0u1dLTv41IZc0TMfqn1bY0Z7spyfqn0Kkmv-b5H00mycqn7ts0ZKs5H00Ugws5H00uAwETjYk0ZFJ5H00IZN15HnsPHf4PW6vP1TzPWcknH6krjb0mynqnfKsUWYs0ZK9I7qhUA7M5H00ugPY5H00ugwGujYVnfK9TLKWm1Ys0ZNspy4Wm1Ys0AuWIgfqn0K9uAu_myTqnfKLuMFEUHY0mMfqnfKzug7Y5HDvP104rjRvPHbdrHR0Tv-b5H0smhc1PHD4rHc4mHw-PWD0ULfqnfKETMKY5HcWnanknanzc1b1PWTdnWbsnan1rH0sc1n4nj08nan1c1cWnanV0AVG5H00UgfqnW0vn6KVm1YzPWfdn16sn1mkn0KVmdqhThqV5H00uA78IyF-gLK_my4GuZnqn0K9uZ745UAGdroyCogD_XL5d6K9uZ7Y5H00pgPWUjYs0Z7VIjYs0A7bgLPEIgFWuHYznzPYpgw_uNqkIyNzXiPxgdqxUMnWIA-YUARWUhksgvkY0APzm1Yvn1TsP6&us=newvui&ai=0_429389852_1_0&word=&ck=0.0.0.0.0.0.0.0&shh=baike.baidu.com)  [躁郁自测，抑郁测量表，网上的测评试题 评分标准，综合心理，](http://www.baidu.com/baidu.php?url=Ks00000EAMrnlPLIyCNSmyXz9UGTU52obwY0vDioEMchaZhpNgYYofrKouLvcTqto0hdwElFKg6Iv-oyLybb3CA9pGUy9OdhJ5mFJfWFJbzMFvrZabGsfmVVjXuet92gcBQVas355TwhaU0ZSy2M2gpeb7htKt3pj6losOyuKSs19s0ze-hysXcjXvATQ-OBUJ5nT0dRNCBD-JM7CdFnktr_Jh_3.7Y_NR2Ar5Od66E89WtJPgKwzl526eZKfHZf68Hfuxwe3h2SMowJ32rgwuuuY4PlhGv-5QWdQjPakYeVdB6.U1Yk0ZDqE2HRlVjRV5r1GVpB8EQc86KY5IHJYOHgdQQB0A-V5HDzPWc0Iybq0ZKGujYzn0KWpyfqP1c0mhbqn10k0AuY5H00TA6qn0KET1Ys0AFL5H00UMfqn0K1XWY0ThIYmyTqn0K8IM0qna3snj0snj0sn0K-ThTqn0KYTh7buHYs0AFbpyfqnW77fbc4nYu7wWFDnRwArj97fYfYrD7DrRwKPDnvnbD0uAPWujY0mgPxpywW5gK1QyIlpZ940AqW5HD0u1dLTv41IZc0TMfqn1bY0Z7spyfqn0Kkmv-b5H00mycqn7ts0ZKs5H00Ugws5H00uAwETjYk0ZFJ5H00IZN15HnsPHf4PW6vP1TzPWcknH6krjb0mynqnfKsUWYs0ZK9I7qhUA7M5H00ugPY5H00ugwGujYVnfK9TLKWm1Ys0ZNspy4Wm1Ys0AuWIgfqn0K9uAu_myTqnfKLuMFEUHY0mMfqnfKzug7Y5HDvP104rjRvPHbdrHR0Tv-b5H0smhc1PHD4rHc4mHw-PWD0ULfqnfKETMKY5HcWnanknanzc1b1PWTdnWbsnan1rH0sc1n4nj08nan1c1cWnanV0AVG5H00UgfqnW0vn6KVm1YzPWfdn16sn1mkn0KVmdqhThqV5H00uA78IyF-gLK_my4GuZnqn0K9uZ745UAGdroyCogD_XL5d6K9uZ7Y5H00pgPWUjYs0Z7VIjYs0A7bgLPEIgFWuHYznzPYpgw_uNqkIyNzXiPxgdqxUMnWIA-YUARWUhksgvkY0APzm1Yvn1TsP6&us=newvui&ai=0_429389852_1_0&word=&ck=0.0.0.0.0.0.0.0&shh=baike.baidu.com)  [健康测试，心理测试，心理抑郁，全方位解读，图表分析，针对 …](http://www.baidu.com/baidu.php?url=Ks00000EAMrnlPLIyCNSmyXz9UGTU52obwY0vDioEMchaZhpNgYYofrKouLvcTqto0hdwElFKg6Iv-oyLybb3CA9pGUy9OdhJ5mFJfWFJbzMFvrZabGsfmVVjXuet92gcBQVas355TwhaU0ZSy2M2gpeb7htKt3pj6losOyuKSs19s0ze-hysXcjXvATQ-OBUJ5nT0dRNCBD-JM7CdFnktr_Jh_3.7Y_NR2Ar5Od66E89WtJPgKwzl526eZKfHZf68Hfuxwe3h2SMowJ32rgwuuuY4PlhGv-5QWdQjPakYeVdB6.U1Yk0ZDqE2HRlVjRV5r1GVpB8EQc86KY5IHJYOHgdQQB0A-V5HDzPWc0Iybq0ZKGujYzn0KWpyfqP1c0mhbqn10k0AuY5H00TA6qn0KET1Ys0AFL5H00UMfqn0K1XWY0ThIYmyTqn0K8IM0qna3snj0snj0sn0K-ThTqn0KYTh7buHYs0AFbpyfqnW77fbc4nYu7wWFDnRwArj97fYfYrD7DrRwKPDnvnbD0uAPWujY0mgPxpywW5gK1QyIlpZ940AqW5HD0u1dLTv41IZc0TMfqn1bY0Z7spyfqn0Kkmv-b5H00mycqn7ts0ZKs5H00Ugws5H00uAwETjYk0ZFJ5H00IZN15HnsPHf4PW6vP1TzPWcknH6krjb0mynqnfKsUWYs0ZK9I7qhUA7M5H00ugPY5H00ugwGujYVnfK9TLKWm1Ys0ZNspy4Wm1Ys0AuWIgfqn0K9uAu_myTqnfKLuMFEUHY0mMfqnfKzug7Y5HDvP104rjRvPHbdrHR0Tv-b5H0smhc1PHD4rHc4mHw-PWD0ULfqnfKETMKY5HcWnanknanzc1b1PWTdnWbsnan1rH0sc1n4nj08nan1c1cWnanV0AVG5H00UgfqnW0vn6KVm1YzPWfdn16sn1mkn0KVmdqhThqV5H00uA78IyF-gLK_my4GuZnqn0K9uZ745UAGdroyCogD_XL5d6K9uZ7Y5H00pgPWUjYs0Z7VIjYs0A7bgLPEIgFWuHYznzPYpgw_uNqkIyNzXiPxgdqxUMnWIA-YUARWUhksgvkY0APzm1Yvn1TsP6&us=newvui&ai=0_429389852_1_0&word=&ck=0.0.0.0.0.0.0.0&shh=baike.baidu.com)  [bdcs.lhwljy.top](http://www.baidu.com/baidu.php?url=Ks00000EAMrnlPLIyCNSmyXz9UGTU52obwY0vDioEMchaZhpNgYYofrKouLvcTqto0hdwElFKg6Iv-oyLybb3CA9pGUy9OdhJ5mFJfWFJbzMFvrZabGsfmVVjXuet92gcBQVas355TwhaU0ZSy2M2gpeb7htKt3pj6losOyuKSs19s0ze-hysXcjXvATQ-OBUJ5nT0dRNCBD-JM7CdFnktr_Jh_3.7Y_NR2Ar5Od66E89WtJPgKwzl526eZKfHZf68Hfuxwe3h2SMowJ32rgwuuuY4PlhGv-5QWdQjPakYeVdB6.U1Yk0ZDqE2HRlVjRV5r1GVpB8EQc86KY5IHJYOHgdQQB0A-V5HDzPWc0Iybq0ZKGujYzn0KWpyfqP1c0mhbqn10k0AuY5H00TA6qn0KET1Ys0AFL5H00UMfqn0K1XWY0ThIYmyTqn0K8IM0qna3snj0snj0sn0K-ThTqn0KYTh7buHYs0AFbpyfqnW77fbc4nYu7wWFDnRwArj97fYfYrD7DrRwKPDnvnbD0uAPWujY0mgPxpywW5gK1QyIlpZ940AqW5HD0u1dLTv41IZc0TMfqn1bY0Z7spyfqn0Kkmv-b5H00mycqn7ts0ZKs5H00Ugws5H00uAwETjYk0ZFJ5H00IZN15HnsPHf4PW6vP1TzPWcknH6krjb0mynqnfKsUWYs0ZK9I7qhUA7M5H00ugPY5H00ugwGujYVnfK9TLKWm1Ys0ZNspy4Wm1Ys0AuWIgfqn0K9uAu_myTqnfKLuMFEUHY0mMfqnfKzug7Y5HDvP104rjRvPHbdrHR0Tv-b5H0smhc1PHD4rHc4mHw-PWD0ULfqnfKETMKY5HcWnanknanzc1b1PWTdnWbsnan1rH0sc1n4nj08nan1c1cWnanV0AVG5H00UgfqnW0vn6KVm1YzPWfdn16sn1mkn0KVmdqhThqV5H00uA78IyF-gLK_my4GuZnqn0K9uZ745UAGdroyCogD_XL5d6K9uZ7Y5H00pgPWUjYs0Z7VIjYs0A7bgLPEIgFWuHYznzPYpgw_uNqkIyNzXiPxgdqxUMnWIA-YUARWUhksgvkY0APzm1Yvn1TsP6&us=newvui&ai=0_429389852_1_0&word=&ck=0.0.0.0.0.0.0.0&shh=baike.baidu.com) |
| --- |

| 岔 搜索发现  [激躁性大肠症候群](https://www.baidu.com/s?word=%E6%BF%80%E8%BA%81%E6%80%A7%E5%A4%A7%E8%82%A0%E7%97%87%E5%80%99%E7%BE%A4&tn=SE_baikepcxf02_fcetbk02&pos=baike_pc_turbo_1767&ori_sid=00bb350b8f61098b)  [大肠激躁症能喝酸奶吗](https://www.baidu.com/s?word=%E5%A4%A7%E8%82%A0%E6%BF%80%E8%BA%81%E7%97%87%E8%83%BD%E5%96%9D%E9%85%B8%E5%A5%B6%E5%90%97&tn=SE_baikepcxf02_fcetbk02&pos=baike_pc_turbo_1767&ori_sid=00bb350b8f61098b) | [肠炎的症状](https://www.baidu.com/s?word=%E8%82%A0%E7%82%8E%E7%9A%84%E7%97%87%E7%8A%B6&tn=SE_baikepcxf02_fcetbk02&pos=baike_pc_turbo_1767&ori_sid=00bb350b8f61098b)  [大肠激躁症可以自愈](https://www.baidu.com/s?word=%E5%A4%A7%E8%82%A0%E6%BF%80%E8%BA%81%E7%97%87%E5%8F%AF%E4%BB%A5%E8%87%AA%E6%84%88&tn=SE_baikepcxf02_fcetbk02&pos=baike_pc_turbo_1767&ori_sid=00bb350b8f61098b) | [激躁性大肠要吃药吗](https://www.baidu.com/s?word=%E6%BF%80%E8%BA%81%E6%80%A7%E5%A4%A7%E8%82%A0%E8%A6%81%E5%90%83%E8%8D%AF%E5%90%97&tn=SE_baikepcxf02_fcetbk02&pos=baike_pc_turbo_1767&ori_sid=00bb350b8f61098b) [狂躁症](https://www.baidu.com/s?word=%E7%8B%82%E8%BA%81%E7%97%87&tn=SE_baikepcxf02_fcetbk02&pos=baike_pc_turbo_1767&ori_sid=00bb350b8f61098b) | [肠梗阻症状及治疗](https://www.baidu.com/s?word=%E8%82%A0%E6%A2%97%E9%98%BB%E7%97%87%E7%8A%B6%E5%8F%8A%E6%B2%BB%E7%96%97&tn=SE_baikepcxf02_fcetbk02&pos=baike_pc_turbo_1767&ori_sid=00bb350b8f61098b)  [暴躁症](https://www.baidu.com/s?word=%E6%9A%B4%E8%BA%81%E7%97%87&tn=SE_baikepcxf02_fcetbk02&pos=baike_pc_turbo_1767&ori_sid=00bb350b8f61098b) | [运动治好了我的肠易激](https://www.baidu.com/s?word=%E8%BF%90%E5%8A%A8%E6%B2%BB%E5%A5%BD%E4%BA%86%E6%88%91%E7%9A%84%E8%82%A0%E6%98%93%E6%BF%80&tn=SE_baikepcxf02_fcetbk02&pos=baike_pc_turbo_1767&ori_sid=00bb350b8f61098b) [狂躁症吃什么药](https://www.baidu.com/s?word=%E7%8B%82%E8%BA%81%E7%97%87%E5%90%83%E4%BB%80%E4%B9%88%E8%8D%AF&tn=SE_baikepcxf02_fcetbk02&pos=baike_pc_turbo_1767&ori_sid=00bb350b8f61098b) |
| --- | --- | --- | --- | --- |

| Q | 新手上路 [成长任务](https://baike.baidu.com/usercenter/tasks#guide)  [编辑规则](https://baike.baidu.com/help#main06) | [编辑入门](https://baike.baidu.com/help#main01)  [本人编辑](https://baike.baidu.com/item/%E7%99%BE%E5%BA%A6%E7%99%BE%E7%A7%91%EF%BC%9A%E6%9C%AC%E4%BA%BA%E8%AF%8D%E6%9D%A1%E7%BC%96%E8%BE%91%E6%9C%8D%E5%8A%A1/22442459?bk_fr=pcFooter) |  | 我有疑问 [内容质疑](javascript:void(0);) [官方贴吧](http://tieba.baidu.com/f?ie=utf-8&fr=bks0000&kw=%E7%99%BE%E5%BA%A6%E7%99%BE%E7%A7%91) | 投诉建议  [举报不良信息](http://help.baidu.com/newadd?word=%E6%BF%80%E8%BA%81%E6%80%A7%E5%A4%A7%E8%82%A0%E7%97%87%E5%80%99%E7%BE%A4&&submit_link=https%3A%2F%2Fbaike.baidu.com%2Fitem%2F%25E6%25BF%2580%25E8%25BA%2581%25E6%2580%25A7%25E5%25A4%25A7%25E8%2582%25A0%25E7%2597%2587%25E5%2580%2599%25E7%25BE%25A4%2F4549268%3FfromModule%3Dsearch-result_lemma&prod_id=10&category=1) [投诉侵权信息](http://help.baidu.com/newadd?word=%E6%BF%80%E8%BA%81%E6%80%A7%E5%A4%A7%E8%82%A0%E7%97%87%E5%80%99%E7%BE%A4&&submit_link=https%3A%2F%2Fbaike.baidu.com%2Fitem%2F%25E6%25BF%2580%25E8%25BA%2581%25E6%2580%25A7%25E5%25A4%25A7%25E8%2582%25A0%25E7%2597%2587%25E5%2580%2599%25E7%25BE%25A4%2F4549268%3FfromModule%3Dsearch-result_lemma&prod_id=10&category=6)  [在线客服](http://zhiqiu.baidu.com/baike/passport/html/baikechat.html) [意见反馈](javascript:void(0);) |
| --- | --- | --- | --- | --- | --- |

©2022 Baidu [使用百度前必读](http://www.baidu.com/duty/) | [百科协议](http://help.baidu.com/question?prod_en=baike&class=89&id=1637) | [隐私政策](http://help.baidu.com/question?prod_id=10&class=690&id=1001779) | [百度百科合作平台](https://baike.baidu.com/operation/cooperation) | 京ICP证030173号 [京公网安备11000002000001号](http://www.beian.gov.cn/portal/registerSystemInfo?recordcode=11000002000001)

<https://baike.baidu.com/item/>激躁性大肠症候群/4549268?fromModule=search-result_lemma

[未通过词条申诉](http://help.baidu.com/newadd?word=%E6%BF%80%E8%BA%81%E6%80%A7%E5%A4%A7%E8%82%A0%E7%97%87%E5%80%99%E7%BE%A4&&submit_link=https%3A%2F%2Fbaike.baidu.com%2Fitem%2F%25E6%25BF%2580%25E8%25BA%2581%25E6%2580%25A7%25E5%25A4%25A7%25E8%2582%25A0%25E7%2597%2587%25E5%2580%2599%25E7%25BE%25A4%2F4549268%3FfromModule%3Dsearch-result_lemma&prod_id=10&category=2)

[封禁查询与解封](http://help.baidu.com/newadd?word=%E6%BF%80%E8%BA%81%E6%80%A7%E5%A4%A7%E8%82%A0%E7%97%87%E5%80%99%E7%BE%A4&&submit_link=https%3A%2F%2Fbaike.baidu.com%2Fitem%2F%25E6%25BF%2580%25E8%25BA%2581%25E6%2580%25A7%25E5%25A4%25A7%25E8%2582%25A0%25E7%2597%2587%25E5%2580%2599%25E7%25BE%25A4%2F4549268%3FfromModule%3Dsearch-result_lemma&prod_id=10&category=5)

2/2
