# Supplementary material for: Assessment of medical information on irritable bowel syndrome information in Wikipedia and Baidu Encyclopedia: comparative study
Source: PeerJ. 2024 May 24;12:e17264. doi: 10.7717/peerj.17264 (PMC11129691; doi:10.7717/peerj.17264)
Supplement: Data S1 [file peerj-12-17264-s001.zip › σÄƒσoïμò░μì«/Baidu/Baidu-English/6-σñoΦéáμ┐ÇΦ║üτùç_τÖ╛σ║aτÖ╛τoæ.docx]

| 2022/12/14 10:41  [网页](https://www.baidu.com/) | [新闻](http://news.baidu.com/) | 大肠激躁症_百度百科  [贴吧](https://tieba.baidu.com/) [知道](https://zhidao.baidu.com/) [网盘](https://pan.baidu.com/?from=1027327l) [图片](http://image.baidu.com/) | [视频](http://v.baidu.com/) | [地图](http://map.baidu.com/) | [文库](https://wenku.baidu.com/) | 百科 | [百度首页](http://www.baidu.com/) [登录](javascript:;) |
| --- | --- | --- | --- | --- | --- | --- | --- |

| [岔](https://baike.baidu.com/) | \| 大肠激躁症 \| 进入词条 \| \| --- \| --- \| | \| 全站搜索 \| \| --- \| | [帮助](https://baike.baidu.com/help) |
| --- | --- | --- | --- | --- | --- | --- |
| 近期有不法分子冒充百度百科官方人员，以删除词条为由威胁并敲诈相关企业。在此严正声明：百度百科是免费编辑平台，绝不存在收费代编服务，请勿上当受骗！ [详情>>](https://baike.baidu.com/common/declaration) | | | |
| [首页](https://baike.baidu.com/) 秒懂百科 特色百科 用户 知识专题 权威合作 [口下载百科APP](https://baike.baidu.com/wapui/subpage/baikeappdownload?sfrom=pc_lemmapage_navigation) [2 个](https://baike.baidu.com/usercenter) | | | |

| 大肠激躁症 | \| [小播报](javascript:;) \| \| --- \| | \| [c编辑](javascript:;) \| \| --- \| | \| [上传视频](javascript:;) \| \| --- \| | . 收藏 [山 34](javascript:void(0);) 1 | \| 词条统计  浏览次数： 41855次  编辑次数： 4次[历史版本](https://baike.baidu.com/historylist/%E5%A4%A7%E8%82%A0%E6%BF%80%E8%BA%81%E7%97%87/3109976)  最近更新： [yghm](https://baike.baidu.com/usercenter/userpage?uk=CwPuzWXvk8Fzpi5IqCzNNA&from=lemma) ( 2012-06-01)  突出贡献榜  [crocosmia](https://baike.baidu.com/usercenter/userpage?uk=3R4HDrhSyqNd4nVPAZPnWQ&from=lemma) \| \| --- \| |
| --- | --- | --- | --- | --- | --- | --- | --- | --- | --- |
| Constipation-Irritable Bowel Syndrome, or C-IBS. Irritable bowel disorder is simply a syndrome manifested by abnormal function of the intestine. Symptoms may include abdominal pain, changes in bowel habits, gas, bloating, a feeling of incomplete bowel relief, or mucus in the stool. Almost every adult has experienced one or more of these conditions. However, when these symptoms occur together, persist, and have considerable life distress, they tend to be recognized as a dysfunctional condition. | | | | |  |
| \| 目录 \| ▪ [(2)饮食因素](#_bookmark1)  1 [简介](#_bookmark4)  2 [病因及致病机转](#_bookmark5)  ▪ ( 1)肠道蠕动功能障 碍  3 [诊断](#_bookmark6)  ▪ [(3)神经异常](#_bookmark2)  ▪ [(4)感觉异常](#_bookmark3)  ▪ ( 5)肠道与大脑之讯  息交流异常 \| \| --- \| --- \| | | | | |  |
|  |  |  |  |  | \| **1** 哈佛大学申请 **12** 网络工程师  **2** 电商平台怎么 **13** 自己创建个  **3** 37游戏平台 **14** 网络安全培  **4** 虚拟货币平台 **15** 无人机反制  **5** csgo电脑配置 **16** 价格便宜的  **6** 亚马逊图书 **17** sci论文投稿  **7** 图书批发网 **18** vr消防演练  **8** 购买域名 **19** csgo网站开  **9** 游戏盒子 **20** 国际期货  **10** 出版社自费出 **21** 怎么创建小  **11** 战队logo设计 **22** 供应链管理 \| \| --- \| |
| [小 播报c编辑](javascript:;)  简介 | | | | |  |
| Intestinal irritability is not fatal, but it is rarely cured and can occur or progress throughout life. Often there are no guidelines for defecation habits – sometimes constipation, sometimes diarrhea, and sometimes both in the short term. The difference between irritable bowel disease and organic lesions is that its symptoms cannot be explained by specific lesions, or even clearly explained by pathophysiology. Although the patient's intestine has obvious functional abnormalities, the current technology cannot accurately measure it, and there is a lack of appropriate diagnostic tools and related examinations. Therefore, clinically, the presence of irritable bowel disorder can only be diagnosed by the symptoms described by the patient. | | | | |  |
|  |  |  |  |  | [女疊 口](javascript:void(0);) |
| 病因及致病机转  [小 播报c编辑](javascript:;) | | | | |  |
| The following are possible causes of bowel irritability, but the current conclusive is still inconclusive:  (1) Intestinal peristalsis dysfunction  This theory advocates that some patients have slow intestinal peristalsis, some are faster, and some patients alternate between fast and slow, and the pattern is changeable. At present, this theory has yet to be confirmed by the results of the study of intestinal peristalsis.  (2) Dietary factors  If a specific drug is used to cause constipation in normal volunteers, they can also develop symptoms of irritation of the bowel. The reduction of fiber intake can also have a similar effect. However, no studies have confirmed that the amount of fiber consumed by patients with irritable bowel is different from that of normal people. Therefore, the lack of fiber does not seem to be a clear cause of disease. Although many patients believe that the onset of irritable bowel disorder is related to specific foods, this is not easy to confirm by objective methods. In fact, feeding itself stimulates intestinal peristalsis. In summary, food intake, especially eating large amounts of food at one time, can stimulate symptoms in a non-specific way in patients with irritable bowel disorder, but it has not been confirmed to be related to specific foods.   1. Neurological abnormalities   Another theory is that symptoms stem from abnormalities in the intestinal nervous system rather than the gut itself. Many current studies have focused on the interintestinal plexus, also known as the "brain of the gut". The study of the interintestinal plexus and its association with the central nervous system has opened up a new field: gastrointestinal neurology. Scholars believe that understanding the physiological characteristics and chemical composition of the interintestinal plexus will help solve the mystery of irritable colorectal disorder. The interintestinal plexus is quite complex and contains most of the nerve transmitters contained in the central nervous system. For example, the electrical activity of the intestines during sleep is the same frequency as that of the brain, and patients often have abnormal REM sleep. However, the interintestinal plexus has not been proven to be the main cause of irritable bowel disorder.  (4) Paresthesia  Since the 1980s , the theory of paresthesias has also been proposed and attracted widespread attention. Many studies have found that when a balloon is placed in the rectum or sigmoid colon and starts pumping, people with irritable bowel disorder feel discomfort or pain at a smaller amount or pressure than normal. Similar conditions can occur in the small intestine and even in the esophagus. However, the threshold of skin pain in patients with irritable bowel disorder is no different from that of normal people, or even higher, so there is a theory of "intestinal hypersensitivity". This may explain why patients with irritable bowel disorder are oversensitive to normal stimuli, resulting in abnormal reflexes and bowel movements. But what is the cause of oversensitivity of the intestines?  The following conditions are thought to cause intestinal hypersensitivity:  -Damage to the intestinal mucosa (such as inflammation, infection, or exposure to toxic chemicals)  - Hyperextension of the intestinal muscles | | | | |  |
|  |  |  |  |  |  |

<https://baike.baidu.com/item/>大肠激躁症/3109976?fromModule=search-result_lemma 1/3

2022/12/14 10:41

[女疊口](javascript:void(0);)

大肠激躁症_百度百科

- Leakage at the junction of spinal nerves

- A precipitating signal is transmitted from a higher nerve center through the spinal nerves

(5) Abnormal communication between the intestine and the brain

In addition to integrating the previous theory, this theory also takes into account psychological and physiological factors, which can also seriously affect the formation of symptoms.

[小 播报c编辑](javascript:;)

诊断

The challenge for clinicians in the diagnosis of irritable bowel syndrome is how to analyze the symptoms of patients and recognize the subtypes of irritable bowel syndrome. The Manning Criteria identified six symptoms that are more common in irritable bowel disorder than in structural celiac disease:

I. Abdominal pain relief after defecation

II. When the abdominal pain begins, the stool becomes soft

III. More frequent defecation at the onset of abdominal pain

IV. Bloating

V. Loosen mucus

VI. Feeling that the stool is not clean

In 1999, a group of gastroenterologists who were highly interested in the research of irritable bowel syndrome formulated the Rome II Criteria based on the Manning Criteria and Rome Criteria, which has become the most widely accepted and applied diagnostic criteria:

※ In the past 12 months, abdominal pain or discomfort occurred for at least 12 weeks (not necessarily consecutive), and at least two of the following characteristics were included:

I. Abdominal discomfort relieved by defecation

II. Change in frequency of defecation

III. Changes in Stool Morphology

※ One or more of the following symptoms occur at least a quarter of the time when symptoms are present, and can be used to distinguish different types of irritable bowel syndrome. These symptoms are not necessary for a diagnosis, but the more present they are, the more convincing doctors are for the diagnosis of irritable bowel disorder:

I. Abnormal bowel movement frequency (more than three times a day or less than three times a week)

II. Abnormal stool patterns (hard lumps or loose/watery stools)

III. Abnormal defecation pattern (forced, urgent or feeling that the stool is not clean)

IV. Drain mucus

V. Gas or feeling bloated

Note: Patients must have been free of symptoms of structural or metabolic disease causing these symptoms for twelve consecutive weeks

It is worth noting that if the patient has the following symptoms, it is a warning sign of an organic disease, and appropriate examinations must be arranged in a step:

I. Bloody stool or discharge from the anus

II. Weight Loss

III. Persistent diarrhea

IV. Recent, unrelieved abdominal distension

V. anemia

VI. Fever

For example, enteritis, particularly erosive enteritis, may meet five Manning Criteria criteria. However, its bloody stool pattern, fever, weight loss, and anemia can be used as a differential.

| 岔 搜索发现  [激躁性大肠要吃药吗](https://www.baidu.com/s?word=%E6%BF%80%E8%BA%81%E6%80%A7%E5%A4%A7%E8%82%A0%E8%A6%81%E5%90%83%E8%8D%AF%E5%90%97&tn=SE_baikepcxf02_fcetbk02&pos=baike_pc_turbo_1767&ori_sid=00bb350c8f9b552a)  [大肠激躁症如何治疗](https://www.baidu.com/s?word=%E5%A4%A7%E8%82%A0%E6%BF%80%E8%BA%81%E7%97%87%E5%A6%82%E4%BD%95%E6%B2%BB%E7%96%97&tn=SE_baikepcxf02_fcetbk02&pos=baike_pc_turbo_1767&ori_sid=00bb350c8f9b552a) [激躁性大肠症怎么治愈](https://www.baidu.com/s?word=%E6%BF%80%E8%BA%81%E6%80%A7%E5%A4%A7%E8%82%A0%E7%97%87%E6%80%8E%E4%B9%88%E6%B2%BB%E6%84%88&tn=SE_baikepcxf02_fcetbk02&pos=baike_pc_turbo_1767&ori_sid=00bb350c8f9b552a)  [大肠激躁症能喝酸奶吗](https://www.baidu.com/s?word=%E5%A4%A7%E8%82%A0%E6%BF%80%E8%BA%81%E7%97%87%E8%83%BD%E5%96%9D%E9%85%B8%E5%A5%B6%E5%90%97&tn=SE_baikepcxf02_fcetbk02&pos=baike_pc_turbo_1767&ori_sid=00bb350c8f9b552a) [大肠激躁症会背痛](https://www.baidu.com/s?word=%E5%A4%A7%E8%82%A0%E6%BF%80%E8%BA%81%E7%97%87%E4%BC%9A%E8%83%8C%E7%97%9B&tn=SE_baikepcxf02_fcetbk02&pos=baike_pc_turbo_1767&ori_sid=00bb350c8f9b552a)  [大肠激躁症吃什么药](https://www.baidu.com/s?word=%E5%A4%A7%E8%82%A0%E6%BF%80%E8%BA%81%E7%97%87%E5%90%83%E4%BB%80%E4%B9%88%E8%8D%AF&tn=SE_baikepcxf02_fcetbk02&pos=baike_pc_turbo_1767&ori_sid=00bb350c8f9b552a) [部队文职是什么工作](https://www.baidu.com/s?word=%E9%83%A8%E9%98%9F%E6%96%87%E8%81%8C%E6%98%AF%E4%BB%80%E4%B9%88%E5%B7%A5%E4%BD%9C&tn=SE_baikepcxf02_fcetbk02&pos=baike_pc_turbo_1767&ori_sid=00bb350c8f9b552a)  [大肠激躁症可以自愈](https://www.baidu.com/s?word=%E5%A4%A7%E8%82%A0%E6%BF%80%E8%BA%81%E7%97%87%E5%8F%AF%E4%BB%A5%E8%87%AA%E6%84%88&tn=SE_baikepcxf02_fcetbk02&pos=baike_pc_turbo_1767&ori_sid=00bb350c8f9b552a) [免费兼职](https://www.baidu.com/s?word=%E5%85%8D%E8%B4%B9%E5%85%BC%E8%81%8C&tn=SE_baikepcxf02_fcetbk02&pos=baike_pc_turbo_1767&ori_sid=00bb350c8f9b552a)  [激躁性大肠症放屁多](https://www.baidu.com/s?word=%E6%BF%80%E8%BA%81%E6%80%A7%E5%A4%A7%E8%82%A0%E7%97%87%E6%94%BE%E5%B1%81%E5%A4%9A&tn=SE_baikepcxf02_fcetbk02&pos=baike_pc_turbo_1767&ori_sid=00bb350c8f9b552a) |
| --- |

<https://baike.baidu.com/item/>大肠激躁症/3109976?fromModule=search-result_lemma 2/3

2022/12/14 10:41 大肠激躁症_百度百科

[女疊 口](javascript:void(0);)

Q 新手上路

我有疑问

投诉建议

[成长任务](https://baike.baidu.com/usercenter/tasks#guide) [编辑规则](https://baike.baidu.com/help#main06)

[编辑入门](https://baike.baidu.com/help#main01) [内容质疑](javascript:void(0);)

[本人编辑](https://baike.baidu.com/item/%E7%99%BE%E5%BA%A6%E7%99%BE%E7%A7%91%EF%BC%9A%E6%9C%AC%E4%BA%BA%E8%AF%8D%E6%9D%A1%E7%BC%96%E8%BE%91%E6%9C%8D%E5%8A%A1/22442459?bk_fr=pcFooter) [官方贴吧](http://tieba.baidu.com/f?ie=utf-8&fr=bks0000&kw=%E7%99%BE%E5%BA%A6%E7%99%BE%E7%A7%91)

[在线客服](http://zhiqiu.baidu.com/baike/passport/html/baikechat.html)

[意见反馈](javascript:void(0);)

[举报不良信息](http://help.baidu.com/newadd?word=%E5%A4%A7%E8%82%A0%E6%BF%80%E8%BA%81%E7%97%87&&submit_link=https%3A%2F%2Fbaike.baidu.com%2Fitem%2F%25E5%25A4%25A7%25E8%2582%25A0%25E6%25BF%2580%25E8%25BA%2581%25E7%2597%2587%2F3109976%3FfromModule%3Dsearch-result_lemma&prod_id=10&category=1) [投诉侵权信息](http://help.baidu.com/newadd?word=%E5%A4%A7%E8%82%A0%E6%BF%80%E8%BA%81%E7%97%87&&submit_link=https%3A%2F%2Fbaike.baidu.com%2Fitem%2F%25E5%25A4%25A7%25E8%2582%25A0%25E6%25BF%2580%25E8%25BA%2581%25E7%2597%2587%2F3109976%3FfromModule%3Dsearch-result_lemma&prod_id=10&category=6)

[未通过词条申诉](http://help.baidu.com/newadd?word=%E5%A4%A7%E8%82%A0%E6%BF%80%E8%BA%81%E7%97%87&&submit_link=https%3A%2F%2Fbaike.baidu.com%2Fitem%2F%25E5%25A4%25A7%25E8%2582%25A0%25E6%25BF%2580%25E8%25BA%2581%25E7%2597%2587%2F3109976%3FfromModule%3Dsearch-result_lemma&prod_id=10&category=2)

[封禁查询与解封](http://help.baidu.com/newadd?word=%E5%A4%A7%E8%82%A0%E6%BF%80%E8%BA%81%E7%97%87&&submit_link=https%3A%2F%2Fbaike.baidu.com%2Fitem%2F%25E5%25A4%25A7%25E8%2582%25A0%25E6%25BF%2580%25E8%25BA%2581%25E7%2597%2587%2F3109976%3FfromModule%3Dsearch-result_lemma&prod_id=10&category=5)

©2022 Baidu [使用百度前必读](http://www.baidu.com/duty/) | [百科协议](http://help.baidu.com/question?prod_en=baike&class=89&id=1637) | [隐私政策](http://help.baidu.com/question?prod_id=10&class=690&id=1001779) | [百度百科合作平台](https://baike.baidu.com/operation/cooperation) | 京ICP证030173号

[京公网安备11000002000001号](http://www.beian.gov.cn/portal/registerSystemInfo?recordcode=11000002000001)

<https://baike.baidu.com/item/>大肠激躁症/3109976?fromModule=search-result_lemma

3/3
