# Supplementary material for: Assessment of medical information on irritable bowel syndrome information in Wikipedia and Baidu Encyclopedia: comparative study
Source: PeerJ. 2024 May 24;12:e17264. doi: 10.7717/peerj.17264 (PMC11129691; doi:10.7717/peerj.17264)
Supplement: Data S1 [file peerj-12-17264-s001.zip › σÄƒσoïμò░μì«/Baidu/Baidu-English/9-μàóμÇoσèƒΦâ╜μÇoΣ╛┐τoÿ_τÖ╛σ║aτÖ╛τoæ.docx]

| 2022/12/14 10:48  [慢性功能性便秘的概述图 张)](https://baike.baidu.com/pic/%E6%85%A2%E6%80%A7%E5%8A%9F%E8%83%BD%E6%80%A7%E4%BE%BF%E7%A7%98/8952323/1/6c224f4a20a4462333086e729b22720e0df3d7e4?fr=lemma&fromModule=lemma_top-image&ct=single)   \| 词条统计  浏览次数： 244558次  编辑次数： 18次[历史版本](https://baike.baidu.com/historylist/%E6%85%A2%E6%80%A7%E5%8A%9F%E8%83%BD%E6%80%A7%E4%BE%BF%E7%A7%98/8952323)  最近更新： [涵宇Q](https://baike.baidu.com/usercenter/userpage?uk=sAKxvCB7Bi22BNyXo0TMfQ&from=lemma) ( 2021-12-04)  突出贡献榜  [bianmi8](https://baike.baidu.com/usercenter/userpage?uk=fn9sdkW-KBqeK3DkiYmx7w&from=lemma) \| \| --- \|   [女](javascript:void(0);)  [疊](javascript:void(0);)  [dna亲子鉴定](https://cpro.baidu.com/cpro/ui/uijs.php?en=mywWUA71T1YsFh7sT7qGujYsFhPC5H0huAbqrauGTdq9TZ0qnauJp1YzmHcYmWT1mvRvuW01nWPWFh_quA49FRnLFRfLFRfLFRf1FRFjFRm3FRcvFRD3FRcvFRRsFRn4FRf4FRnLFR77FhkdpvbqnHchUyPsUHYknHD4njbhTHY1P104PjDYn7qWTZchThcqnauzT1YkFMP-UAk-T-qGujYkFMPGujY1m1nzuWK-PWPWmWTzPjF9FMPYpyfqrauY5gwsmvkGmvV-ujPxpAnhIAfqnHb4P1m1nzuYUHYzPW63njndrjDhIAd15HDvP104rjmsrHmhIZRqIHD4rHTvn1nhIHdCIZwsTzR1fiRzwBRzwhF9pyV-FHF7mh7GuZR-nbNWUvYhIWYzFhbquH9-mHb3PHT&besl=6&c=news&cf=1&cvrq=1893597&eid_list=201577_203434_207574_208118_209357&expid=201577_202257_202564_203434_205809_207574_208118_209394&fr=20&fv=0&haacp=348&img_typ=0&itm=0&lu_idc=gzhxy&lukid=12&lus=3c32f0e63cb7242a&lust=63993970&luwtr=84518820883097642&mscf=0&n=10&nttp=1&p=baidu&pbs=220093&sce=7&sr=72&ssp2=1&tpl=baiduCustITagLinkUnitRankCol&tsf=dtp:1&tu_type=0&u=%2Fitem%2F%25E6%2585%25A2%25E6%2580%25A7%25E5%258A%259F%25E8%2583%25BD%25E6%2580%25A7%25E4%25BE%25BF%25E7%25A7%2598%3FfromModule%3Dlemma%5Fsearch%2Dbox&uicf=lurecv&urlid=0&eot=1)  去德国口留学  [螺杆式家用](https://cpro.baidu.com/cpro/ui/uijs.php?en=mywWUA71T1YsFh7sT7qGujYsFhPC5H0huAbqrauGTdq9TZ0qnauJp1YzmHcYmWT1mvRvuW01nWPWFh_qFRnzFRwDFRc3FRPaFRPKFRFDFRFjFRfzFRf1FRn1FRcdFRRLFRPjFRwDFhkdpvbqnHfhUyPsUHY4PWTvriuk5HnLnjbYnHfsgvPsTBuzmWYsFMF15HDhTvN_UANzgv-b5HDhTv-b5HPWn1FhnARvnvPBP1cYnhDhTLwGujY3FMfqIZKWUA-WpvNbndqCmzuYujYkrHbLPWn1FMwV5Hcvrj6sn1R3niuYUgnqnHmLnjb3PW04PBuYIHddnHb4P1m1nzud5y9YIZK1FHPKFHFAFHFAmh7GpvR-nbNBmy-bIiRzwyPEUiuv5HchpHY4uWIhuW6zrf&besl=6&c=news&cf=1&cvrq=1618597&eid_list=201577_203434_207574_208118_209357&expid=201577_202257_202564_203434_205809_207574_208118_209394&fr=20&fv=0&haacp=547&img_typ=0&itm=0&lu_idc=gzhxy&lukid=14&lus=3c32f0e63cb7242a&lust=63993970&luwtr=7205834330722693679&mscf=0&n=10&nttp=1&p=baidu&pbs=220093&sce=7&sr=72&ssp2=1&tpl=baiduCustITagLinkUnitRankCol&tsf=dtp:1&tu_type=0&u=%2Fitem%2F%25E6%2585%25A2%25E6%2580%25A7%25E5%258A%259F%25E8%2583%25BD%25E6%2580%25A7%25E4%25BE%25BF%25E7%25A7%2598%3FfromModule%3Dlemma%5Fsearch%2Dbox&uicf=lurecv&urlid=0&eot=1) [vr消防演练](https://cpro.baidu.com/cpro/ui/uijs.php?en=mywWUA71T1YsFh7sT7qGujYsFhPC5H0huAbqrauGTdq9TZ0qnauJp1YzmHcYmWT1mvRvuW01nWPWFh_qIMc-fYm-wbc-fWT-f10-wjD-wDf-f1D-fWThUZNopHYkPiuVmLKV5HD1rjczrauk5HnLnjbYnHfsgvPsTBuzmWYsFMF15HDhTvN_UANzgv-b5HDhTv-b5HPWn1FhnARvnvPBP1cYnhDhTLwGujY3FMfqIZKWUA-WpvNbndqCmzuYujYkrHbLPWn1FMwV5Hcvrj6sn1R3niuYUgnqnHmLnjb3PW04PBuYIHddnHb4P1m1nzud5y9YIZK1FHPKFHFAFHFAmh7GpvR-nbNBmy-bIiRzwyPEUiuv5HchpHYknv79PARzP6&besl=6&c=news&cf=1&cvrq=4705752&eid_list=201577_203434_207574_208118_209357&expid=201577_202257_202564_203434_205809_207574_208118_209394&fr=20&fv=0&haacp=1024&img_typ=0&itm=0&lu_idc=gzhxy&lukid=15&lus=3c32f0e63cb7242a&lust=63993970&luwtr=754962655298315806&mscf=0&n=10&nttp=1&p=baidu&pbs=220093&sce=7&sr=72&ssp2=1&tpl=baiduCustITagLinkUnitRankCol&tsf=dtp:1&tu_type=0&u=%2Fitem%2F%25E6%2585%25A2%25E6%2580%25A7%25E5%258A%259F%25E8%2583%25BD%25E6%2580%25A7%25E4%25BE%25BF%25E7%25A7%2598%3FfromModule%3Dlemma%5Fsearch%2Dbox&uicf=lurecv&urlid=0&eot=1) [无人机反制](https://cpro.baidu.com/cpro/ui/uijs.php?en=mywWUA71T1YsFh7sT7qGujYsFhPC5H0huAbqrauGTdq9TZ0qnauJp1YzmHcYmWT1mvRvuW01nWPWFh_qFRP7FRw7FRn3FRPaFRFaFRuKFRcLFRcYFRfvFRnvFhkdpvbqnHmhUyPsUHY3nHnsPzuk5HnLnjbYnHfsgvPsTBuzmWYsFMF15HDhTvN_UANzgv-b5HDhTv-b5HPWn1FhnARvnvPBP1cYnhDhTLwGujY3FMfqIZKWUA-WpvNbndqCmzuYujYkrHbLPWn1FMwV5Hcvrj6sn1R3niuYUgnqnHmLnjb3PW04PBuYIHddnHb4P1m1nzud5y9YIZK1FHPKFHFAFHFAmh7GpvR-nbNBmy-bIiRzwyPEUiuv5HchpHd-uHRsm1czrf&besl=6&c=news&cf=1&cvrq=1415933&eid_list=201577_203434_207574_208118_209357&expid=201577_202257_202564_203434_205809_207574_208118_209394&fr=20&fv=0&haacp=904&img_typ=0&itm=0&lu_idc=gzhxy&lukid=16&lus=3c32f0e63cb7242a&lust=63993970&luwtr=685006414410405008&mscf=0&n=10&nttp=1&p=baidu&pbs=220093&sce=7&sr=72&ssp2=1&tpl=baiduCustITagLinkUnitRankCol&tsf=dtp:1&tu_type=0&u=%2Fitem%2F%25E6%2585%25A2%25E6%2580%25A7%25E5%258A%259F%25E8%2583%25BD%25E6%2580%25A7%25E4%25BE%25BF%25E7%25A7%2598%3FfromModule%3Dlemma%5Fsearch%2Dbox&uicf=lurecv&urlid=0&eot=1) [自己创建个](https://cpro.baidu.com/cpro/ui/uijs.php?en=mywWUA71T1YsFh7sT7qGujYsFhPC5H0huAbqrauGTdq9TZ0qnauJp1YzmHcYmWT1mvRvuW01nWPWFh_qFRfLFRfYFRFjFRFKFRcYFRcYFRFDFRD3FRc3FRmvFRPDFRm3FRfdFRF7FhkdpvbqnHThUyPsUHYknWn1PjThTHY1P104PjDYn7qWTZchThcqnauzT1YkFMP-UAk-T-qGujYkFMPGujY1m1nzuWK-PWPWmWTzPjF9FMPYpyfqrauY5gwsmvkGmvV-ujPxpAnhIAfqnHb4P1m1nzuYUHYzPW63njndrjDhIAd15HDvP104rjmsrHmhIZRqIHD4rHTvn1nhIHdCIZwsTzR1fiRzwBRzwhF9pyV-FHF7mh7GuZR-nbNWUvYhIWYzFhbqPv7hnHIBuW0&besl=6&c=news&cf=1&cvrq=2235877&eid_list=201577_203434_207574_208118_209357&expid=201577_202257_202564_203434_205809_207574_208118_209394&fr=20&fv=0&haacp=1001&img_typ=0&itm=0&lu_idc=gzhxy&lukid=17&lus=3c32f0e63cb7242a&lust=63993970&luwtr=6556233252601032990&mscf=0&n=10&nttp=1&p=baidu&pbs=220093&sce=7&sr=72&ssp2=1&tpl=baiduCustITagLinkUnitRankCol&tsf=dtp:1&tu_type=0&u=%2Fitem%2F%25E6%2585%25A2%25E6%2580%25A7%25E5%258A%259F%25E8%2583%25BD%25E6%2580%25A7%25E4%25BE%25BF%25E7%25A7%2598%3FfromModule%3Dlemma%5Fsearch%2Dbox&uicf=lurecv&urlid=0&eot=1) [csgo电脑配](https://cpro.baidu.com/cpro/ui/uijs.php?en=mywWUA71T1YsFh7sT7qGujYsFhPC5H0huAbqrauGTdq9TZ0qnauJp1YzmHcYmWT1mvRvuW01nWPWFh_qmLPMUzNaPiN7PzNjPaNDPaNjPiN7PaNDPBNjnzu_IyVG5HD3FhdWTAYqP16zP1fhTHY1P104PjDYn7qWTZchThcqnauzT1YkFMP-UAk-T-qGujYkFMPGujY1m1nzuWK-PWPWmWTzPjF9FMPYpyfqrauY5gwsmvkGmvV-ujPxpAnhIAfqnHb4P1m1nzuYUHYzPW63njndrjDhIAd15HDvP104rjmsrHmhIZRqIHD4rHTvn1nhIHdCIZwsTzR1fiRzwBRzwhF9pyV-FHF7mh7GuZR-nbNWUvYhIWYzFhbqn1nvuhDduym&besl=6&c=news&cf=1&cvrq=3472466&eid_list=201577_203434_207574_208118_209357&expid=201577_202257_202564_203434_205809_207574_208118_209394&fr=20&fv=0&haacp=611&img_typ=0&itm=0&lu_idc=gzhxy&lukid=18&lus=3c32f0e63cb7242a&lust=63993970&luwtr=2357911270216075011&mscf=0&n=10&nttp=1&p=baidu&pbs=220093&sce=7&sr=72&ssp2=1&tpl=baiduCustITagLinkUnitRankCol&tsf=dtp:1&tu_type=0&u=%2Fitem%2F%25E6%2585%25A2%25E6%2580%25A7%25E5%258A%259F%25E8%2583%25BD%25E6%2580%25A7%25E4%25BE%25BF%25E7%25A7%2598%3FfromModule%3Dlemma%5Fsearch%2Dbox&uicf=lurecv&urlid=0&eot=1) [自己建个网](https://cpro.baidu.com/cpro/ui/uijs.php?en=mywWUA71T1YsFh7sT7qGujYsFhPC5H0huAbqrauGTdq9TZ0qnauJp1YzmHcYmWT1mvRvuW01nWPWFh_qFRfLFRfYFRFjFRFKFRFDFRD3FRc3FRmvFRPDFRm3FRfdFRF7FhkdpvbqnHbhUyPsUHYdPW0snBuk5HnLnjbYnHfsgvPsTBuzmWYsFMF15HDhTvN_UANzgv-b5HDhTv-b5HPWn1FhnARvnvPBP1cYnhDhTLwGujY3FMfqIZKWUA-WpvNbndqCmzuYujYkrHbLPWn1FMwV5Hcvrj6sn1R3niuYUgnqnHmLnjb3PW04PBuYIHddnHb4P1m1nzud5y9YIZK1FHPKFHFAFHFAmh7GpvR-nbNBmy-bIiRzwyPEUiuv5HchpHYYmW9WmWD1P6&besl=6&c=news&cf=1&cvrq=2719613&eid_list=201577_203434_207574_208118_209357&expid=201577_202257_202564_203434_205809_207574_208118_209394&fr=20&fv=0&haacp=1125&img_typ=0&itm=0&lu_idc=gzhxy&lukid=19&lus=3c32f0e63cb7242a&lust=63993970&luwtr=2471349030649590347&mscf=0&n=10&nttp=1&p=baidu&pbs=220093&sce=7&sr=72&ssp2=1&tpl=baiduCustITagLinkUnitRankCol&tsf=dtp:1&tu_type=0&u=%2Fitem%2F%25E6%2585%25A2%25E6%2580%25A7%25E5%258A%259F%25E8%2583%25BD%25E6%2580%25A7%25E4%25BE%25BF%25E7%25A7%2598%3FfromModule%3Dlemma%5Fsearch%2Dbox&uicf=lurecv&urlid=0&eot=1) [机器人展会](https://cpro.baidu.com/cpro/ui/uijs.php?en=mywWUA71T1YsFh7sT7qGujYsFhPC5H0huAbqrauGTdq9TZ0qnauJp1YzmHcYmWT1mvRvuW01nWPWFh_qFRFaFRuKFRnvFRmLFRn3FRPaFRfdFRc4FRFaFRRkFhkdpvbqnW0hUyPsUHYLPHb1riuk5HnLnjbYnHfsgvPsTBuzmWYsFMF15HDhTvN_UANzgv-b5HDhTv-b5HPWn1FhnARvnvPBP1cYnhDhTLwGujY3FMfqIZKWUA-WpvNbndqCmzuYujYkrHbLPWn1FMwV5Hcvrj6sn1R3niuYUgnqnHmLnjb3PW04PBuYIHddnHb4P1m1nzud5y9YIZK1FHPKFHFAFHFAmh7GpvR-nbNBmy-bIiRzwyPEUiuv5HchpHYLrjbdPjRLP6&besl=6&c=news&cf=1&cvrq=2360884&eid_list=201577_203434_207574_208118_209357&expid=201577_202257_202564_203434_205809_207574_208118_209394&fr=20&fv=0&haacp=1509&img_typ=0&itm=0&lu_idc=gzhxy&lukid=20&lus=3c32f0e63cb7242a&lust=63993970&luwtr=717749295403362800&mscf=0&n=10&nttp=1&p=baidu&pbs=220093&sce=7&sr=72&ssp2=1&tpl=baiduCustITagLinkUnitRankCol&tsf=dtp:1&tu_type=0&u=%2Fitem%2F%25E6%2585%25A2%25E6%2580%25A7%25E5%258A%259F%25E8%2583%25BD%25E6%2580%25A7%25E4%25BE%25BF%25E7%25A7%2598%3FfromModule%3Dlemma%5Fsearch%2Dbox&uicf=lurecv&urlid=0&eot=1) [什么叫云计](https://cpro.baidu.com/cpro/ui/uijs.php?en=mywWUA71T1YsFh7sT7qGujYsFhPC5H0huAbqrauGTdq9TZ0qnauJp1YzmHcYmWT1mvRvuW01nWPWFh_qFRPKFRczFRn1FRcYFRFDFRfsFRfYFRnvFRFjFRnvFRPaFRR1FhkdpvbqnWDhUyPsUHYLP1b3nzuk5HnLnjbYnHfsgvPsTBuzmWYsFMF15HDhTvN_UANzgv-b5HDhTv-b5HPWn1FhnARvnvPBP1cYnhDhTLwGujY3FMfqIZKWUA-WpvNbndqCmzuYujYkrHbLPWn1FMwV5Hcvrj6sn1R3niuYUgnqnHmLnjb3PW04PBuYIHddnHb4P1m1nzud5y9YIZK1FHPKFHFAFHFAmh7GpvR-nbNBmy-bIiRzwyPEUiuv5HchpHYsnhFhnjKBu0&besl=6&c=news&cf=1&cvrq=2481698&eid_list=201577_203434_207574_208118_209357&expid=201577_202257_202564_203434_205809_207574_208118_209394&fr=20&fv=0&haacp=894&img_typ=0&itm=0&lu_idc=gzhxy&lukid=21&lus=3c32f0e63cb7242a&lust=63993970&luwtr=2262954635798908161&mscf=0&n=10&nttp=1&p=baidu&pbs=220093&sce=7&sr=72&ssp2=1&tpl=baiduCustITagLinkUnitRankCol&tsf=dtp:1&tu_type=0&u=%2Fitem%2F%25E6%2585%25A2%25E6%2580%25A7%25E5%258A%259F%25E8%2583%25BD%25E6%2580%25A7%25E4%25BE%25BF%25E7%25A7%2598%3FfromModule%3Dlemma%5Fsearch%2Dbox&uicf=lurecv&urlid=0&eot=1) [动物焚烧炉](https://cpro.baidu.com/cpro/ui/uijs.php?en=mywWUA71T1YsFh7sT7qGujYsFhPC5H0huAbqrauGTdq9TZ0qnauJp1YzmHcYmWT1mvRvuW01nWPWFh_qFRcvFR7AFRP7FRNAFRcLFRf4FRn4FRfdFRnzFR7AFhkdpvbqnWchUyPsUHYdnHnsnauk5HnLnjbYnHfsgvPsTBuzmWYsFMF15HDhTvN_UANzgv-b5HDhTv-b5HPWn1FhnARvnvPBP1cYnhDhTLwGujY3FMfqIZKWUA-WpvNbndqCmzuYujYkrHbLPWn1FMwV5Hcvrj6sn1R3niuYUgnqnHmLnjb3PW04PBuYIHddnHb4P1m1nzud5y9YIZK1FHPKFHFAFHFAmh7GpvR-nbNBmy-bIiRzwyPEUiuv5HchpHY1rAuBuWT3P0&besl=6&c=news&cf=1&cvrq=3128930&eid_list=201577_203434_207574_208118_209357&expid=201577_202257_202564_203434_205809_207574_208118_209394&fr=20&fv=0&haacp=386&img_typ=0&itm=0&lu_idc=gzhxy&lukid=22&lus=3c32f0e63cb7242a&lust=63993970&luwtr=751581624084886335&mscf=0&n=10&nttp=1&p=baidu&pbs=220093&sce=7&sr=72&ssp2=1&tpl=baiduCustITagLinkUnitRankCol&tsf=dtp:1&tu_type=0&u=%2Fitem%2F%25E6%2585%25A2%25E6%2580%25A7%25E5%258A%259F%25E8%2583%25BD%25E6%2580%25A7%25E4%25BE%25BF%25E7%25A7%2598%3FfromModule%3Dlemma%5Fsearch%2Dbox&uicf=lurecv&urlid=0&eot=1)  **1**  **2**  **3**  **4**  **5**  **6**  **7**  **8**  **9**  **10**  **11**  **12**  **13**  **14**  **15**  **16**  **17**  **18**  **19**  **20**  **21**  **22**   \| [自己怎样建网](https://cpro.baidu.com/cpro/ui/uijs.php?en=mywWUA71T1YsFh7sT7qGujYsFhPC5H0huAbqrauGTdq9TZ0qnauJp1YzmHcYmWT1mvRvuW01nWPWFh_qFRfLFRfYFRFjFRFKFRfYFRmdFRfkFRm4FRFDFRD3FRPDFRm3FRfdFRF7FhkdpvbqniuVmLKV5Hm4P1RsFMDqn1TsrHfkPjKxmLKzFMFB5H0hTMnqniu1uyk_ugFxpyfqniu1pyfqnvn1nhmsuHm1mvcLnWfzmiu1IA-b5H6hIjdYTAP_pyPouyf1gv9WFMwb5HD4rHTvn1nhIAYqnWm3rj01PH6kFMwVT1YkPWTsrH6vnjbvFMwd5gRkrHbLPWn1FMRqpZwYTZn-nYD-nbm-nbuBmy-ouiRzwyF9pywdFHF7mvqVFMmqnBuG5H63nHckmHR3&besl=6&c=news&cf=1&cvrq=2061742&eid_list=201577_203434_207574_208118_209357&expid=201577_202257_202564_203434_205809_207574_208118_209394&fr=20&fv=0&haacp=2362&img_typ=0&itm=0&lu_idc=gzhxy&lukid=1&lus=3c32f0e63cb7242a&lust=63993970&luwtr=6786335543231135880&mscf=0&n=10&nttp=1&p=baidu&pbs=220093&sce=7&sr=72&ssp2=1&tpl=baiduCustITagLinkUnitRankCol&tsf=dtp:1&tu_type=0&u=%2Fitem%2F%25E6%2585%25A2%25E6%2580%25A7%25E5%258A%259F%25E8%2583%25BD%25E6%2580%25A7%25E4%25BE%25BF%25E7%25A7%2598%3FfromModule%3Dlemma%5Fsearch%2Dbox&uicf=lurecv&urlid=0&eot=1)  [焊缝无损检测](https://cpro.baidu.com/cpro/ui/uijs.php?en=mywWUA71T1YsFh7sT7qGujYsFhPC5H0huAbqrauGTdq9TZ0qnauJp1YzmHcYmWT1mvRvuW01nWPWFh_qFRFKFRc3FRcLFRNjFRP7FRw7FRPaFRmsFRFjFRNjFRczFRRzFhkdpvbqnBuVmLKV5HDknjnvrauk5HnLnjbYnHfsgvPsTBuzmWYsFMF15HDhTvN_UANzgv-b5HDhTv-b5HPWn1FhnARvnvPBP1cYnhDhTLwGujY3FMfqIZKWUA-WpvNbndqCmzuYujYkrHbLPWn1FMwV5Hcvrj6sn1R3niuYUgnqnHmLnjb3PW04PBuYIHddnHb4P1m1nzud5y9YIZK1FHPKFHFAFHFAmh7GpvR-nbNBmy-bIiRzwyPEUiuv5HchpHYYryR4uycLns&besl=6&c=news&cf=1&cvrq=2411344&eid_list=201577_203434_207574_208118_209357&expid=201577_202257_202564_203434_205809_207574_208118_209394&fr=20&fv=0&haacp=1596&img_typ=0&itm=0&lu_idc=gzhxy&lukid=2&lus=3c32f0e63cb7242a&lust=63993970&luwtr=2196606844417253483&mscf=0&n=10&nttp=1&p=baidu&pbs=220093&sce=7&sr=72&ssp2=1&tpl=baiduCustITagLinkUnitRankCol&tsf=dtp:1&tu_type=0&u=%2Fitem%2F%25E6%2585%25A2%25E6%2580%25A7%25E5%258A%259F%25E8%2583%25BD%25E6%2580%25A7%25E4%25BE%25BF%25E7%25A7%2598%3FfromModule%3Dlemma%5Fsearch%2Dbox&uicf=lurecv&urlid=0&eot=1)  [价格便宜的香](https://cpro.baidu.com/cpro/ui/uijs.php?en=mywWUA71T1YsFh7sT7qGujYsFhPC5H0huAbqrauGTdq9TZ0qnauJp1YzmHcYmWT1mvRvuW01nWPWFh_qFRFjFRwaFRc3FRmkFRckFRR1FRfzFRPaFRcdFRnYFRPAFRR1FRc3FRwaFRcLFRu7FRP7FRmkFRnvFRmLFhkdpvbqnzuVmLKV5HT4PWDsFMDqn1TsrHfkPjKxmLKzFMFB5H0hTMnqniu1uyk_ugFxpyfqniu1pyfqnvn1nhmsuHm1mvcLnWfzmiu1IA-b5H6hIjdYTAP_pyPouyf1gv9WFMwb5HD4rHTvn1nhIAYqnWm3rj01PH6kFMwVT1YkPWTsrH6vnjbvFMwd5gRkrHbLPWn1FMRqpZwYTZn-nYD-nbm-nbuBmy-ouiRzwyF9pywdFHF7mvqVFMmqnBuG5HndPWFbP1Ih&besl=6&c=news&cf=1&cvrq=2363942&eid_list=201577_203434_207574_208118_209357&expid=201577_202257_202564_203434_205809_207574_208118_209394&fr=20&fv=0&haacp=1750&img_typ=0&itm=0&lu_idc=gzhxy&lukid=3&lus=3c32f0e63cb7242a&lust=63993970&luwtr=18274635138362951998&mscf=0&n=10&nttp=1&p=baidu&pbs=220093&sce=7&sr=72&ssp2=1&tpl=baiduCustITagLinkUnitRankCol&tsf=dtp:1&tu_type=0&u=%2Fitem%2F%25E6%2585%25A2%25E6%2580%25A7%25E5%258A%259F%25E8%2583%25BD%25E6%2580%25A7%25E4%25BE%25BF%25E7%25A7%2598%3FfromModule%3Dlemma%5Fsearch%2Dbox&uicf=lurecv&urlid=0&eot=1)  [电脑主机多少](https://cpro.baidu.com/cpro/ui/uijs.php?en=mywWUA71T1YsFh7sT7qGujYsFhPC5H0huAbqrauGTdq9TZ0qnauJp1YzmHcYmWT1mvRvuW01nWPWFh_qFRcdFRRLFRnYFRfYFRfvFRmLFRFaFRuKFRcvFRRsFRn4FRf4FRnLFR77FhkdpvbqPauVmLKV5HmLnjnLFMDqn1TsrHfkPjKxmLKzFMFB5H0hTMnqniu1uyk_ugFxpyfqniu1pyfqnvn1nhmsuHm1mvcLnWfzmiu1IA-b5H6hIjdYTAP_pyPouyf1gv9WFMwb5HD4rHTvn1nhIAYqnWm3rj01PH6kFMwVT1YkPWTsrH6vnjbvFMwd5gRkrHbLPWn1FMRqpZwYTZn-nYD-nbm-nbuBmy-ouiRzwyF9pywdFHF7mvqVFMmqnBuG5H63P1Phnymv&besl=6&c=news&cf=1&cvrq=2978018&eid_list=201577_203434_207574_208118_209357&expid=201577_202257_202564_203434_205809_207574_208118_209394&fr=20&fv=0&haacp=217&img_typ=0&itm=0&lu_idc=gzhxy&lukid=4&lus=3c32f0e63cb7242a&lust=63993970&luwtr=6400949698628764850&mscf=0&n=10&nttp=1&p=baidu&pbs=220093&sce=7&sr=72&ssp2=1&tpl=baiduCustITagLinkUnitRankCol&tsf=dtp:1&tu_type=0&u=%2Fitem%2F%25E6%2585%25A2%25E6%2580%25A7%25E5%258A%259F%25E8%2583%25BD%25E6%2580%25A7%25E4%25BE%25BF%25E7%25A7%2598%3FfromModule%3Dlemma%5Fsearch%2Dbox&uicf=lurecv&urlid=0&eot=1)  [蓄热式焚烧炉](https://cpro.baidu.com/cpro/ui/uijs.php?en=mywWUA71T1YsFh7sT7qGujYsFhPC5H0huAbqrauGTdq9TZ0qnauJp1YzmHcYmWT1mvRvuW01nWPWFh_qFRfsFRN7FRn3FRn3FRPKFRFDFRcLFRf4FRn4FRfdFRnzFR7AFhkdpvbqPiuVmLKV5HRvnHDvFMDqn1TsrHfkPjKxmLKzFMFB5H0hTMnqniu1uyk_ugFxpyfqniu1pyfqnvn1nhmsuHm1mvcLnWfzmiu1IA-b5H6hIjdYTAP_pyPouyf1gv9WFMwb5HD4rHTvn1nhIAYqnWm3rj01PH6kFMwVT1YkPWTsrH6vnjbvFMwd5gRkrHbLPWn1FMRqpZwYTZn-nYD-nbm-nbuBmy-ouiRzwyF9pywdFHF7mvqVFMmqnBuG5H7-rjm4PHR3&besl=6&c=news&cf=1&cvrq=2461324&eid_list=201577_203434_207574_208118_209357&expid=201577_202257_202564_203434_205809_207574_208118_209394&fr=20&fv=0&haacp=1686&img_typ=0&itm=0&lu_idc=gzhxy&lukid=5&lus=3c32f0e63cb7242a&lust=63993970&luwtr=2270768104828311148&mscf=0&n=10&nttp=1&p=baidu&pbs=220093&sce=7&sr=72&ssp2=1&tpl=baiduCustITagLinkUnitRankCol&tsf=dtp:1&tu_type=0&u=%2Fitem%2F%25E6%2585%25A2%25E6%2580%25A7%25E5%258A%259F%25E8%2583%25BD%25E6%2580%25A7%25E4%25BE%25BF%25E7%25A7%2598%3FfromModule%3Dlemma%5Fsearch%2Dbox&uicf=lurecv&urlid=0&eot=1)  [高温隔热材料](https://cpro.baidu.com/cpro/ui/uijs.php?en=mywWUA71T1YsFh7sT7qGujYsFhPC5H0huAbqrauGTdq9TZ0qnauJp1YzmHcYmWT1mvRvuW01nWPWFh_qFRc3FRwAFRP7FRnzFRc3FRmYFRn3FRn3FRczFRnYFRnkFRPAFhkdpvbqPBuVmLKV5HTsnjmLFMDqn1TsrHfkPjKxmLKzFMFB5H0hTMnqniu1uyk_ugFxpyfqniu1pyfqnvn1nhmsuHm1mvcLnWfzmiu1IA-b5H6hIjdYTAP_pyPouyf1gv9WFMwb5HD4rHTvn1nhIAYqnWm3rj01PH6kFMwVT1YkPWTsrH6vnjbvFMwd5gRkrHbLPWn1FMRqpZwYTZn-nYD-nbm-nbuBmy-ouiRzwyF9pywdFHF7mvqVFMmqnBuG5yRdPHTLmvf1&besl=6&c=news&cf=1&cvrq=2582209&eid_list=201577_203434_207574_208118_209357&expid=201577_202257_202564_203434_205809_207574_208118_209394&fr=20&fv=0&haacp=1183&img_typ=0&itm=0&lu_idc=gzhxy&lukid=6&lus=3c32f0e63cb7242a&lust=63993970&luwtr=2200308873988409662&mscf=0&n=10&nttp=1&p=baidu&pbs=220093&sce=7&sr=72&ssp2=1&tpl=baiduCustITagLinkUnitRankCol&tsf=dtp:1&tu_type=0&u=%2Fitem%2F%25E6%2585%25A2%25E6%2580%25A7%25E5%258A%259F%25E8%2583%25BD%25E6%2580%25A7%25E4%25BE%25BF%25E7%25A7%2598%3FfromModule%3Dlemma%5Fsearch%2Dbox&uicf=lurecv&urlid=0&eot=1)  [哈佛大学申请](https://cpro.baidu.com/cpro/ui/uijs.php?en=mywWUA71T1YsFh7sT7qGujYsFhPC5H0huAbqrauGTdq9TZ0qnauJp1YzmHcYmWT1mvRvuW01nWPWFh_qFRc4FRu7FRcLFRmsFRcYFRm1FRfkFRDLFRn4FRNKFRnLFRNaFRPjFRmdFRFjFRu7FhkdpvbqPzuVmLKV5HbknHnLFMDqn1TsrHfkPjKxmLKzFMFB5H0hTMnqniu1uyk_ugFxpyfqniu1pyfqnvn1nhmsuHm1mvcLnWfzmiu1IA-b5H6hIjdYTAP_pyPouyf1gv9WFMwb5HD4rHTvn1nhIAYqnWm3rj01PH6kFMwVT1YkPWTsrH6vnjbvFMwd5gRkrHbLPWn1FMRqpZwYTZn-nYD-nbm-nbuBmy-ouiRzwyF9pywdFHF7mvqVFMmqnBuG5H04nhwbuHwW&besl=6&c=news&cf=1&cvrq=2024364&eid_list=201577_203434_207574_208118_209357&expid=201577_202257_202564_203434_205809_207574_208118_209394&fr=20&fv=0&haacp=870&img_typ=0&itm=0&lu_idc=gzhxy&lukid=7&lus=3c32f0e63cb7242a&lust=63993970&luwtr=7841139350953723636&mscf=0&n=10&nttp=1&p=baidu&pbs=220093&sce=7&sr=72&ssp2=1&tpl=baiduCustITagLinkUnitRankCol&tsf=dtp:1&tu_type=0&u=%2Fitem%2F%25E6%2585%25A2%25E6%2580%25A7%25E5%258A%259F%25E8%2583%25BD%25E6%2580%25A7%25E4%25BE%25BF%25E7%25A7%2598%3FfromModule%3Dlemma%5Fsearch%2Dbox&uicf=lurecv&urlid=0&eot=1)  [俄语口语学习](https://cpro.baidu.com/cpro/ui/uijs.php?en=mywWUA71T1YsFh7sT7qGujYsFhPC5H0huAbqrauGTdq9TZ0qnauJp1YzmHcYmWT1mvRvuW01nWPWFh_qFRcvFRNDFRf1FRNAFRFAFRwKFRf1FRNAFRfkFRDLFRPAFRcsFhkdpvbqrauVmLKV5HRLnWbzFMDqn1TsrHfkPjKxmLKzFMFB5H0hTMnqniu1uyk_ugFxpyfqniu1pyfqnvn1nhmsuHm1mvcLnWfzmiu1IA-b5H6hIjdYTAP_pyPouyf1gv9WFMwb5HD4rHTvn1nhIAYqnWm3rj01PH6kFMwVT1YkPWTsrH6vnjbvFMwd5gRkrHbLPWn1FMRqpZwYTZn-nYD-nbm-nbuBmy-ouiRzwyF9pywdFHF7mvqVFMmqnBuG5yRzmhFhmHRL&besl=6&c=news&cf=1&cvrq=2822133&eid_list=201577_203434_207574_208118_209357&expid=201577_202257_202564_203434_205809_207574_208118_209394&fr=20&fv=0&haacp=727&img_typ=0&itm=0&lu_idc=gzhxy&lukid=8&lus=3c32f0e63cb7242a&lust=63993970&luwtr=2414741795559411788&mscf=0&n=10&nttp=1&p=baidu&pbs=220093&sce=7&sr=72&ssp2=1&tpl=baiduCustITagLinkUnitRankCol&tsf=dtp:1&tu_type=0&u=%2Fitem%2F%25E6%2585%25A2%25E6%2580%25A7%25E5%258A%259F%25E8%2583%25BD%25E6%2580%25A7%25E4%25BE%25BF%25E7%25A7%2598%3FfromModule%3Dlemma%5Fsearch%2Dbox&uicf=lurecv&urlid=0&eot=1)  [新概念英语网](https://cpro.baidu.com/cpro/ui/uijs.php?en=mywWUA71T1YsFh7sT7qGujYsFhPC5H0huAbqrauGTdq9TZ0qnauJp1YzmHcYmWT1mvRvuW01nWPWFh_qFRfsFRnzFRc3FRndFRnYFRN7FRf1FRDzFRf1FRNAFRPDFRm3FRFAFRP7FhkdpvbqriuVmLKV5HDsPj0dnzuk5HnLnjbYnHfsgvPsTBuzmWYsFMF15HDhTvN_UANzgv-b5HDhTv-b5HPWn1FhnARvnvPBP1cYnhDhTLwGujY3FMfqIZKWUA-WpvNbndqCmzuYujYkrHbLPWn1FMwV5Hcvrj6sn1R3niuYUgnqnHmLnjb3PW04PBuYIHddnHb4P1m1nzud5y9YIZK1FHPKFHFAFHFAmh7GpvR-nbNBmy-bIiRzwyPEUiuv5HchpHd9mvwhrjP9u0&besl=6&c=news&cf=1&cvrq=3212251&eid_list=201577_203434_207574_208118_209357&expid=201577_202257_202564_203434_205809_207574_208118_209394&fr=20&fv=0&haacp=1071&img_typ=0&itm=0&lu_idc=gzhxy&lukid=9&lus=3c32f0e63cb7242a&lust=63993970&luwtr=6912353898211953940&mscf=0&n=10&nttp=1&p=baidu&pbs=220093&sce=7&sr=72&ssp2=1&tpl=baiduCustITagLinkUnitRankCol&tsf=dtp:1&tu_type=0&u=%2Fitem%2F%25E6%2585%25A2%25E6%2580%25A7%25E5%258A%259F%25E8%2583%25BD%25E6%2580%25A7%25E4%25BE%25BF%25E7%25A7%2598%3FfromModule%3Dlemma%5Fsearch%2Dbox&uicf=lurecv&urlid=0&eot=1)  [战队logo设计](https://cpro.baidu.com/cpro/ui/uijs.php?en=mywWUA71T1YsFh7sT7qGujYsFhPC5H0huAbqrauGTdq9TZ0qnauJp1YzmHcYmWT1mvRvuW01nWPWFh_qFRfdFRFDFRcvFRf1UAqMUzNjriN7raNafzNjPBu_IyVG5HDsFhdWTAYqrHm3PjnhTHY1P104PjDYn7qWTZchThcqnauzT1YkFMP-UAk-T-qGujYkFMPGujY1m1nzuWK-PWPWmWTzPjF9FMPYpyfqrauY5gwsmvkGmvV-ujPxpAnhIAfqnHb4P1m1nzuYUHYzPW63njndrjDhIAd15HDvP104rjmsrHmhIZRqIHD4rHTvn1nhIHdCIZwsTzR1fiRzwBRzwhF9pyV-FHF7mh7GuZR-nbNWUvYhIWYzFhbqmH0zuW9Bm1b&besl=6&c=news&cf=1&cvrq=1756706&eid_list=201577_203434_207574_208118_209357&expid=201577_202257_202564_203434_205809_207574_208118_209394&fr=20&fv=0&haacp=707&img_typ=0&itm=0&lu_idc=gzhxy&lukid=10&lus=3c32f0e63cb7242a&lust=63993970&luwtr=1863292750894598650&mscf=0&n=10&nttp=1&p=baidu&pbs=220093&sce=7&sr=72&ssp2=1&tpl=baiduCustITagLinkUnitRankCol&tsf=dtp:1&tu_type=0&u=%2Fitem%2F%25E6%2585%25A2%25E6%2580%25A7%25E5%258A%259F%25E8%2583%25BD%25E6%2580%25A7%25E4%25BE%25BF%25E7%25A7%2598%3FfromModule%3Dlemma%5Fsearch%2Dbox&uicf=lurecv&urlid=0&eot=1)  [10万级无尘车](https://cpro.baidu.com/cpro/ui/uijs.php?en=mywWUA71T1YsFh7sT7qGujYsFhPC5H0huAbqrauGTdq9TZ0qnauJp1YzmHcYmWT1mvRvuW01nWPWFh_qnH0-fYf-wWc-fbn-fWm-fYR-wDR-fWn-fbR-fWn-fWR-fbn-wHfhUZNopHYkniuVmLKV5HT3nW6LFMDqn1TsrHfkPjKxmLKzFMFB5H0hTMnqniu1uyk_ugFxpyfqniu1pyfqnvn1nhmsuHm1mvcLnWfzmiu1IA-b5H6hIjdYTAP_pyPouyf1gv9WFMwb5HD4rHTvn1nhIAYqnWm3rj01PH6kFMwVT1YkPWTsrH6vnjbvFMwd5gRkrHbLPWn1FMRqpZwYTZn-nYD-nbm-nbuBmy-ouiRzwyF9pywdFHF7mvqVFMmqnBuG5HIbPjNWPvcd&besl=6&c=news&cf=1&cvrq=3846261&eid_list=201577_203434_207574_208118_209357&expid=201577_202257_202564_203434_205809_207574_208118_209394&fr=20&fv=0&haacp=369&img_typ=0&itm=0&lu_idc=gzhxy&lukid=11&lus=3c32f0e63cb7242a&lust=63993970&luwtr=7318665999692295159&mscf=0&n=10&nttp=1&p=baidu&pbs=220093&sce=7&sr=72&ssp2=1&tpl=baiduCustITagLinkUnitRankCol&tsf=dtp:1&tu_type=0&u=%2Fitem%2F%25E6%2585%25A2%25E6%2580%25A7%25E5%258A%259F%25E8%2583%25BD%25E6%2580%25A7%25E4%25BE%25BF%25E7%25A7%2598%3FfromModule%3Dlemma%5Fsearch%2Dbox&uicf=lurecv&urlid=0&eot=1) \| \| --- \| | 慢性功能性便秘_百度百科  [网页](https://www.baidu.com/) [新闻](http://news.baidu.com/) [贴吧](https://tieba.baidu.com/) [知道](https://zhidao.baidu.com/) [网盘](https://pan.baidu.com/?from=1027327l) [图片](http://image.baidu.com/) | [视频](http://v.baidu.com/) | [地图](http://map.baidu.com/) | [文库](https://wenku.baidu.com/) | 百科 | [百度首页](http://www.baidu.com/) [登录](javascript:;) |
| --- | --- | --- | --- | --- | --- | --- | --- | --- |

| [岔](https://baike.baidu.com/) | \| 慢性功能性便秘 \| 进入词条 \| \| --- \| --- \| | \| 全站搜索 \| \| --- \| | [帮助](https://baike.baidu.com/help) |
| --- | --- | --- | --- | --- | --- | --- |
| 近期有不法分子冒充百度百科官方人员，以删除词条为由威胁并敲诈相关企业。在此严正声明：百度百科是免费编辑平台，绝不存在收费代编服务，请勿上当受骗！ [详情>>](https://baike.baidu.com/common/declaration) | | | |
| [首页](https://baike.baidu.com/) 秒懂百科 特色百科 用户 知识专题 权威合作 [口下载百科APP](https://baike.baidu.com/wapui/subpage/baikeappdownload?sfrom=pc_lemmapage_navigation) [2 个](https://baike.baidu.com/usercenter) | | | |

| . 收藏 [山 16](javascript:void(0);) 17  慢性功能性便秘 [上传视频](javascript:;)   \| 小播报 \| \| --- \|  \| !编辑 \| \| --- \|  \| 讨论 \| \| --- \|   常见病  Chronic functional constipation is a common disease, frequently-occurring disease. It mainly refers to the decrease in the frequency of defecation, the decrease in the amount of feces, the dryness of feces, and the difficulty of defecation. Constipation must be judged based on the properties of the stool, my usual bowel habits, and whether there is any difficulty in defecation. If it exceeds 6 months, it is chronic constipation. | | | | | | |
| --- | --- | --- | --- | --- | --- | --- | --- | --- | --- |
| 多发群体  常见病因 | 老人，女性  饮食因素，精神压力 | | 西医学名 所属科室 | | 慢性功能性[便秘](https://baike.baidu.com/item/%E4%BE%BF%E7%A7%98/332148?fromModule=lemma_inlink)  [内科](https://baike.baidu.com/item/%E5%86%85%E7%A7%91/274870?fromModule=lemma_inlink) - 消化内科 | |
| 相关视频 | | 1.4万播放  01:24 | | | | 查看全部 > |
| 8085播放  01:21 | |  |  |  |  |  |
|  |  | 有一种便秘叫“慢性功能性便  秘”，你要知道！ | | | |  |
| 如何治疗便秘？ | |  |  |  |  |  |
| \| 目录 \| 1 [诊断标准](#_bookmark4) 2 [病因病机](#_bookmark5) 3 [形成原因](#_bookmark6)  7 [辨证施治要点](#_bookmark7) 8 [慢性便秘治疗](#_bookmark8) 9 [不同病症区别](#_bookmark9)   \| 4 [食疗方法](#_bookmark1) \| \| --- \| \| 5 [治疗便秘方法](#_bookmark2) \| \| 6 [缓解便秘妙招](#_bookmark3) \| \| \| --- \| --- \| --- \| --- \| --- \| | | | | | | |
| 诊断标准 | | | | [小 播报!编辑](javascript:;) | | |
| Diagnostic criteria: 2 or more of the following symptoms in a continuous or intermittent manner for at least 3 months in the past year: (1) Laborious bowel movements in less than 1/4 of the time; (2) Less than 1/4 of the time there is fecal dry knots; (3) Less than 1/4 of the time there is a feeling of incomplete bowel movements; (4) obstruction or anorectal obstruction during defecation in less than 1/4 of the time; (5) Less than 1/4 of the time requires manual assistance in defecation; (6) Less than 3 bowel movements per week in less than 1/4 of the time. absence of loose stools and failure to meet diagnostic criteria for irritable bowel syndrome;It is also necessary to exclude intestinal or systemic organic causes and constipation due to drug factors. | | | | | | |
| 病因病机 | | | | [小 播报!编辑](javascript:;) | | |
| "Treatise on Febrile Diseases" divides constipation into yang knot, yin knot, and spleen contraction. Tang Rongchuan's "Blood Syndrome" says: "When the lungs move heat to the large intestine, there will be constipation. The occurrence of constipation is closely related to the large intestine, spleen, stomach, lung, liver, kidney and other viscera. "Internal Classics" says: "The large intestine is the official of conduction, and the changes come out." It is also proposed that "the lungs and the large intestine are exterior and interior." Spleen and stomach are the oceans of transporting and transforming water and grains. The spleen governs transport and transformation, the stomach governs harmony and descending. This also depends on the function of the liver governing catharsis. If the liver is stagnant and qi stagnant, the internal qi will be blocked, and if the qi stagnation is not enough, the large intestine will fail. Kidney governs both defecation and defecation. Insufficient kidney qi leads to weak conduction in the large intestine and difficulty in excreting stool. It is generally believed that excessive heat in the body, or eating too much fat, sweet and delicious food, can easily cause gastrointestinal heat accumulation, consume body fluid, and make it difficult to pass stool; Abnormal descending, internal stop of dross; exogenous cold evil, overeating cold and cold, leading to yin and cold, stagnation of gastrointestinal tract, and inability to conduct dross; fatigue due to diet, old age and physical weakness, serious illness and postpartum, all may appear due to different constitutions Deficiency of qi and depletion of yang, deficiency of yin and lack of blood, etc., lead to weak conduction in the large intestine, or intestinal obstruction, resulting in constipation. Western medicine divides functional constipation into three types: slow transit type, outlet obstruction type, and mixed type. Traditional Chinese medicine believes that slow transit constipation is mostly constipation due to deficiency syndrome, such as old age and infirmity, long-term illness after childbirth, and weak body; or eating fatigue, overeating raw and cold, feeling cold and damp, and hurting the spleen and stomach, all of which can cause lung, spleen, The kidneys are weak. If the spleen is deficient, the movement and transformation will be weak, and the essence of water and grain will not  It has to be transferred, and the dregs are unable to move in the large intestine. Deficiency of the lung leads to abnormal circulation and descending, the lung and the large intestine are on the outside and inside, and the large intestine is thus unfavorable to the movement of qi, and its operation is blocked. Hua Tuo's "Zhongzang Jing" says: "The large intestine is also the organ of the lungs. It is the director of transmission and the official of the prison. If the lung disease lasts for a long time, it can be passed down to the large intestine." : "The reason why the large intestine can conduct is that it is the organ of the lung. The lung qi is transmitted, so it can conduct." The kidney governs water and controls the two stools. Kidney deficiency will cause the intestines to lose moisture, open and close poorly, and dregs will be blocked. Jin Yuan's "Lan Shi Mi Zang · Stool Dryness" said: "The husband's kidney governs the five liquids, and the body fluid moistens the stool as usual. ... There are also old people who are weak and dry due to insufficient body fluid." Empirical constipation. Such as worry and anger, liver depression and qi stagnation, overeating raw, cold, fatty, sweet, greasy, spicy food, or addicted to tobacco and alcohol, exogenous pathogenic factors, depending on the constitution, the pathogens are transformed from heat or cold, resulting in or accumulated heat in the stomach, Or stagnation of yin and cold, or stagnation of qi, so that the internal qi is blocked, the stomach qi does not descend, and the large intestine loses conduction. For example, the "Nei Jing" says: "The reversal of Taiyin will cause abdominal distension and then unfavorable", mentioning that constipation is related to the cold of the spleen and stomach; , so the pain makes it difficult to close”, which explains the pathological basis of constipation caused by accumulated heat in the intestines and stomach. The liver governs catharsis and helps conduction in the large intestine. If the stagnation of liver qi is stagnant, the qi movement in the large intestine will be unfavorable and the qi of the organs will not flow. For example, "Synopsis of the Golden Chamber · Constipation" states: "A person with secret qi will stagnate in the qi, and things won't work."  Mixed constipation is mostly a combination of deficiency and excess, or constipation with deficiency and excess. It is originally deficiency, and its standard is excess.  The etiology of constipation is complex, and various syndromes may appear concurrently or transform into each other during the development of the disease. Syndrome differentiation should take deficiency and excess as the key link, and yin and yang, qi and blood as the order. Its disease location is in the large intestine, and the pathogenesis is related to the spleen, stomach, lung, liver and kidney. Western medicine disease classification and TCM syndrome classification have their inherent laws to follow, but because of the variability and complexity of the disease itself, it should not be rigidly adhered to. During the treatment, the syndrome should be examined to seek the cause, and the cause should be treated. | | | | | | |

<https://baike.baidu.com/item/>慢性功能性便秘?fromModule=lemma_search-box

1/5

2022/12/14 10:48

慢性功能性便秘_百度百科

|  | | | |
| --- | --- | --- | --- |
| 形成原因 | | [小 播报编辑](javascript:;) | |
| 1. Dietary factors: Some people eat too little food, too refined food, insufficient cellulose and water in the food, which cannot stimulate the intestinal tract to a certain extent, slow intestinal peristalsis, and cannot push food residues to the rectum in time. The residence time in the intestine is prolonged, and the excessive water absorption makes the stool dry.  2. Drug effects: Long-term abuse of laxatives reduces the excitability of nerve sensory cells in the intestinal wall. Even if there is a sufficient amount of feces in the intestines, normal peristalsis and defecation reflex cannot be produced, resulting in intractable defecation difficulties.  3. Diseases of the large intestine: diseases such as allergic colitis, diverticulitis of the large intestine, and Hirschsprung's disease can cause spasm and abnormal movement of the large intestine, making it difficult to pass stool and defecate.  4. Delaying the time of defecation: Some people regard defecation as irrelevant, which can be done sooner or later, and ignore the habit of regular defecation, so that the nerve cells on the rectal wall become slow in responding to the pressure sensation generated after feces enter the rectum, making the feces It stays in the rectum for a long time without causing the feeling of defecation, forming habitual difficulty in defecation.  5. Insufficient defecation power: defecation not only requires the relaxation of the anal sphincter, the levator ani muscle is pulled upward and outward, but also the diaphragm must be lowered, the abdominal muscles contracted, and breath-holding force is required to push the feces out. Old and weak, bedridden for a long time, postpartum, etc., may have insufficient defecation motivation due to weakened diaphragm, abdominal muscle, and anal sphincter contraction and lower abdominal pressure.  6. Mental factors: Strong mental stimulation, panic, emotional stress, anxiety, or high concentration on a certain job will make the desire to defecate disappear, resulting in difficulty in defecation. In addition, nervous system disorders, endocrine disorders, vitamin deficiency, etc. can also cause difficulty in defecation [1]. | | | |
|  | 食疗方法  [小 播报编辑](javascript:;)  What to eat more for patients with constipation  1. Drink at least 8 glasses of water a day, especially when eating high-fiber foods, you should pay more attention to drinking water;  2. Eat more fresh vegetables and increase the fiber intake in the diet;  3. Add bran, wheat bran, etc. every day to expand the volume of feces, promote intestinal peristalsis, and reduce the occurrence of constipation;  4. Get up every morning on an empty stomach and drink warm water with honey water, honey has a lubricating effect on the intestines;  5. Increase the supply of B vitamin food, try to choose natural, unprocessed food, such as coarse grains, beans,  Yeast, etc., to enhance the tension of the intestinal tract;  6. Eat more celery, walnut kernels, ripe bananas, walnuts, pomelo, apples, grapefruit, brown rice, carrots, sweet potatoes, etc.  治疗便秘方法  [小 播报编辑](javascript:;)  Therapeutic method  What to eat more for patients with constipation  1. Drink at least 8 glasses of water a day, especially when eating high-fiber foods, you should pay more attention to drinking water;  2. Eat more fresh vegetables and increase the fiber intake in the diet;  3. Add bran, wheat bran, etc. every day to expand the volume of feces, promote intestinal peristalsis, and reduce the occurrence of constipation;  4. Get up every morning on an empty stomach and drink warm water with honey water, honey has a lubricating effect on the intestines;  5. Increase the supply of B vitamin food, try to choose natural, unprocessed food, such as coarse grains, beans,  Yeast, etc., to enhance the tension of the intestinal tract;  6. Eat more celery, walnut kernels, ripe bananas, walnuts, pomelo, apples, grapefruit, brown rice, carrots, sweet potatoes, etc.  Treatment for constipation  Remedy 1: Roasted and ground sesame (black sesame is the best) 2~3 tablespoons, mixed with warm water (after boiling water is warm) 200~  300 ml plus 3 to 5 tablespoons of 10-day mature acacia honey, made into a paste and taken orally, once in the morning and once in the evening. Treat Habits With This Remedy  50 cases of sexual constipation, the fastest effect is 2 days, the longest is more than 10 days, 40 cases are effective in 2-10 days, 10 cases are effective in more than 10 days,  All 50 cases achieved good results.  Remedy 2: 60 grams of honey, 30 grams each morning and evening, drink with cold boiled water. Suitable for the elderly, pregnant women with constipation and  Habitual constipation.  Recipe 3: 60 grams of honey, 6 grams of royal jelly, mix them thoroughly, and take them with warm water twice a day in the morning and evening.  Habitual constipation.  Remedy 4: Grind 150 grams of bee pollen, add 250 grams of honey to make a paste, take 1 tablespoon every morning and evening on an empty stomach, suitable for habitual constipation.  Recipe 5: 10-day mature acacia flower honey, appropriate amount of white radish, first wash and cut the white radish into slices, dip in honey and eat raw, several times a day. Most suitable for adolescents with constipation.  Remedy 6: Proper amount of honey and bananas, peel the bananas and eat them raw with honey, several times a day. Most suitable for the elderly and habitual constipation. | | [女疊 口](javascript:void(0);) |
|  |  |  |  |
|  |  | |  |
|  |  |  |  |
|  | 缓解便秘妙招  [小 播报编辑](javascript:;)  Skipping rope relieves constipation | |  |

<https://baike.baidu.com/item/>慢性功能性便秘?fromModule=lemma_search-box

2/5

2022/12/14 10:48

慢性功能性便秘_百度百科

|  | |  |  | |
| --- | --- | --- | --- | --- |
| Women are more likely to experience constipation than men. First of all, because women eat less, finer, and more refined food, their intestinal peristalsis is slower. Second, women exercise less, and regular exercise can make the intestines move better.  The best way to prevent constipation is to vibrate the internal organs by skipping rope. Bounce can stimulate bones and muscles, promote blood circulation, and also strengthen the immune function of the lymphatic system, which is very important for relieving constipation.  When people with constipation walk, they can increase the rotation of the waist and crotch as much as possible, and walk like a model, which can massage the abdominal cavity, strengthen the viscera, especially the peristalsis of the stomach, and promote the absorption of nutrients and waste disposal. It has a particularly obvious curative effect on constipation caused by gastrointestinal dysfunction and indigestion.  The bathroom is decorated in light yellow to help defecation  A new Japanese study found that some yellow decorations in the bathroom are helpful for defecation. Yellow is a warm tone, which has the effect of relaxing and relieving tension. When people defecate, the muscles of the body are in a state of tension and contraction. At this time, they maintain a happy mood, relax the muscles, and help defecation. Choose warm colors in the bathroom, such as light yellow, light green, light blue, light orange, etc., which can help you calm down, while purple and red colors are easy to stimulate the nerves, which is not conducive to mental relaxation. In a bathroom with a relatively narrow space, the yellow color can also have the effect of broadening the field of vision, making the whole bathroom more soft, spacious and bright.  Drink vinegar to prevent constipation  The acidic component of vinegar is similar to the digestive juice in the stomach, so vinegar can stimulate the stomach and promote intestinal peristalsis. The resulting enhanced sense of defecation can prevent the stool from staying in the body for a long time and dry out, which has a good effect on preventing constipation. When a person is full, the intestines are filled, and vinegar is not easy to irritate the stomach, so it is best to eat vinegar for laxatives in the morning and evening when you are empty stomach.  When eating, do not exceed 1 tablespoon each time, but not less than half a tablespoon, and no more than 3 times a day.  After eating, immediately drink a glass of warm water. Because the concentration of raw vinegar is too high, if you eat too much, it may cause certain damage to the gastric mucosa, so you must master the amount of vinegar you consume.  You can also add an equal amount of honey to a tablespoon of mature vinegar, stir evenly with warm or cold water and drink it.  Raisins for Constipation  The saying that raisins can cure constipation has not been recorded in ancient medical books, and there are very few related reports so far, and there are two opposite views on it: It is reported that raisins contain fiber and tartaric acid, which can allow excrement to pass through the rectum quickly. A small handful can prevent and treat constipation; some people think that eating more raisins is "easy to generate internal heat", which will aggravate constipation instead.  However, there is no reliable experimental or clinical research to prove these two views. Experiments have shown that grape seed oil extracted from grape seeds can have a laxative effect on animals.  However, most of the raisins sold in the market are seedless, and the chance of curing constipation is very small. Even if it is effective occasionally, it is only a case. It does not mean that this method is also effective for other people, and it is not suitable to follow suit.  drink a glass of water after waking up  A glass of boiled water in the morning can replenish the water lost by the body's metabolism, and it can also promote excretion and prevent constipation. It can also thin the blood and promote blood circulation, which is especially important for women who lose weight. You can also use light salt water instead of plain water.  Take a bath and rub your stomach skillfully to cure constipation  When taking a bath, massage the abdomen in a clockwise direction with the palm of your hand. At the same time, the abdomen bulges and retracts to breathe heavily, and shower the abdomen, which can treat chronic constipation and prevent hemorrhoids. For nervous constipation, it is necessary to flush with hot water at 40°C for about 3 minutes along the intestines, and then flush with warm water at 25°C for 10 seconds, repeating 5 times, which can increase the peristalsis of the large intestine.  Walnut Honey Tea for Constipation  Walnut honey tea is a folk prescription commonly used for constipation, and it has a good effect on treating constipation. The specific method is to use 5 grams of walnuts and 1 gram of honey, brew tea with warm boiled water and drink it, which can achieve the effect of moistening the intestines and defecating. | | | | [女疊 口](javascript:void(0);) |
| 辨证施治要点 | | [小 播报编辑](javascript:;) | |  |
| 1. Pay attention to spleen Yin, fluid increase boat travel  By creek "army play" cloud: "the Yin of the spleen and soil were injured, transfer the officer of dereliction of duty". In Plain Questions, "The spleen is insufficient, which makes the nine orifics obstructed". "Ruijing" exegesis: "Lack of disease in the middle, so that the nine orifices are not passable, with the weak temper of the four zang are weak and qi is not good. "The thought from synopsis, spleen Yin deficiency, not to line the stomach fluid, intestinal embellish, namely the formation of spleen about card, lead to constipation. "Spleen and Stomach Theory" says, "The stomach is the source of protection, and the spleen is the source of camp", "the spleen in four seasons is strong and immune to pathogens", when camp is Yin and spleen Yin is sufficient, the spleen transfer function is normal, the subtle can be transformed, and the dfecal can be transmitted. Patients with long-term habitual constipation, especially elderly patients, are mostly deficient in spleen qi or spleen Yin, or both qi and Yin deficiency. Treatment should be protect spleen Gas spleen Yin, runchang purge. Invigorating spleen qi with Sijunzi soup, nourishing spleen Yin with salvia miltiorrhizae soup.  2. Drop and stomach, stagnant since the division  "In the stomach, the sea of the five zangfu organs is also, the water and grain are all into the stomach, and the five zangfu organs are all endowed with qi in the stomach." "The stomach is always qi, and the person who has no stomach qi says inverse, and the inverse dies. Li Gao also said: "If the spleen and stomach are injured internally, all diseases are caused by birth. ", stomach to pass down and stomach gas and the polluted air is reduced, dregs to discharge the large intestine. Through the fall can be used in bitter orange, magnolias officinalis, Raphani seeds, and stomach can be used in ginger, jujube, licorice. | \|  \| \| --- \| | | |  |
|  | | | |  |

<https://baike.baidu.com/item/>慢性功能性便秘?fromModule=lemma_search-box

3/5

2022/12/14 10:48

慢性功能性便秘_百度百科

| 3. Examine the cause and use the drug differently  In the treatment of functional constipation, the prescription should be selected according to the evidence, and the drug should be applied according to the person. Spleen deficiency type constipation, large dose of raw white surgery 30 ~ 60g to strengthen the spleen and replenish laxation; blood deficiency and intestinal dryness with large doses of raw white peony, hemp seeds to replenish blood and moisturize the intestines and laxation; kidney yin deficiency reuse He Shou Wu, raw land to nourish the kidney to moisturize and laxation; kidney yang deficiency reuse cistanche to warm up kidney yang laxation; spleen deficiency reuse raw astragalus 30 ~ 50g; phlegm fever lung and lung loss add almonds, scutellaria baicalensis, Lilou ren to promote lung heat and laxation; large intestine real heat people use rhubarb, knotweed to clear heat and laxative; people who have been ill for a long time use peach kernels, Wine rhubarb to activate blood circulation and dissolve stool; those with liver depression and stagnation reuse tulips and citrus fruits to relieve liver qi and laxation.  [小 播报编辑](javascript:;)  慢性便秘治疗  First of all, we should strengthen scientific life management, maintain a good mental and psychological state, pay attention to high-speed diet, eat more fiber-rich foods, drink enough water, develop the habit of defecating on time, participate in appropriate physical exercises, etc., and also carry out abdominal breathing exercises or massage.  1. Drug treatment: (1) drug treatment can be considered when the efficacy is not achieved by the above methods, for STC patients, the first choice is prokinetics, cisapride as a total gastrointestinal prokinetic agent, effective for some STC patients. A new specific enterotropic motility drug, procal carbride, is a benzofuran compound that acts specifically  5-HT4 receptor is expected to be an ideal drug for the treatment of CFC. Soffer et al. [10] reported that misoprostol can be used to treat intractable constipation. Some Chinese medicines may have a motility-boosting effect and can also be applied selectively. (2) Commonly used laxatives: (1) volumetric laxatives: magnesium sulfate, sodium sulfate, methylcellulose, agar, etc.; (2) Irritating laxatives: senna, castor oil, diester phenol, etc.; (3) Fecal softeners: liquid paraffin, lactulose, etc.; (4) Rectal administration: glycerol suppository, kaiser dew, etc. Long-term abuse of laxatives leading to laxative bowel disease should be avoided.  2. Biofeedback defecation behavior therapy: Biofeedback therapy is a training method to correct uncoordinated defecation behavior, mainly used to treat sphincter incoordination and pelvic floor muscles, external sphincter defecation paradoxical contraction caused by FOOC, some people report its efficacy up to 96%, the method has no drug side effects, low cost, non-invasive and other advantages compared with drug treatment, this therapy has been carried out in China.  3. Surgical treatment: For severe chronic constipation that is ineffective through the above treatment, seriously affecting the quality of life of patients and even nutritional disorders, surgical treatment can be taken. Mainly for STC patients, subtotal colonectomy and ileorectal anastomosis can be used, and anorectal sphincterectomy can be performed for FOOC patients. Although this surgery can restore the rhythmic peristalsis of the intestinal tube and shorten the total gastrointestinal passage time, it must strictly grasp the indications, and also pay attention to postoperative complications such as diarrhea, fecal incontinence, and even recurrence.  [小 播报编辑](javascript:;)  不同病症区别  Functional constipation (functional constipation) and pelvic floor dysfunction (pelvic floor dyssynergia): the former refers to chronic constipation without evidence of organic disease, some patients with functional constipation have the performance of pelvic floor disorder, the latter needs to be accompanied by the performance of pelvic floor disorder, That is, it meets the diagnostic criteria of Rome II for functional constipation, and it also needs to have an objective basis for pelvic floor disorders.  (1) There is evidence of anorectal manometry, electromyography, or X-ray examination, showing that the pelvic floor muscles are inappropriately contracted or unable to relax during repeated defecation movements.  (2) The rectum can undergo sufficient propulsive contraction during force rowing.  (3) And there is evidence of poor stool discharge.  [词条图册 更多图册 >](https://baike.baidu.com/pic/%E6%85%A2%E6%80%A7%E5%8A%9F%E8%83%BD%E6%80%A7%E4%BE%BF%E7%A7%98/8952323?fr=lemma)   \| 参考资料 \| \| \| \| --- \| --- \| --- \| \| 1  [引起大便困难的原因](https://baike.baidu.com/reference/8952323/d534pBFZSmjcEux9giMW6puudTdzEyH3Qc73SKqfBYVAb3IJ8b0oLE7ELq4JPFO5FhM0DGVph4mT48aDuy_6PiAcvcK_QhVZ6-5zfRlYGbFEpK2ToKRYkA)  ．新浪健康[引用日期2014-09-14]  学术论文 \| 内容来自 \|  \|   [于金源，孙长岗，尹国富. 从肝论治慢性功能性便秘．](https://xueshu.baidu.com/usercenter/paper/show?paperid=c159fab5fe04c6d800eee2482893d9d2&tn=SE_baiduxueshu_c1gjeupa&ie=utf-8&site=baike) 《WanFang》， 2004  [金洵，丁义江，王玲玲等. 针刺治疗慢性功能性便秘疗效观察．](https://xueshu.baidu.com/usercenter/paper/show?paperid=08ea5c676b12a77cebaea0b09289a4ff&tn=SE_baiduxueshu_c1gjeupa&ie=utf-8&site=baike) 《CNKI;WanFang》， 2010  [王丽娟，王玲玲. 麦粒灸结合针刺治疗慢性功能性便秘随机对照研究．](https://xueshu.baidu.com/usercenter/paper/show?paperid=a83daa8478be44b3f132313ad0939954&tn=SE_baiduxueshu_c1gjeupa&ie=utf-8&site=baike) 《CNKI;WanFang》， 2011  [施永敏，周永香，马文芳等. 老年慢性功能性便秘的干预护理．](https://xueshu.baidu.com/usercenter/paper/show?paperid=3d1a2ef60c684f1493b52e22a91f78c8&tn=SE_baiduxueshu_c1gjeupa&ie=utf-8&site=baike) 《实用临床医药杂志》， 2005  [郭荣，丁义江，张建淮. 慢性功能性便秘诊断和中医证型的量化研究．](https://xueshu.baidu.com/usercenter/paper/show?paperid=d18bbb7c4b527ec929dd0b6efe923447&tn=SE_baiduxueshu_c1gjeupa&ie=utf-8&site=baike) 《 CNKI》， 2011  [查看全部](https://xueshu.baidu.com/s?wd=intitle%3A%28%E6%85%A2%E6%80%A7%E5%8A%9F%E8%83%BD%E6%80%A7%E4%BE%BF%E7%A7%98%29&tn=SE_baiduxueshu_c1gjeupa&ie=utf-8&sc_from=pingtai6&site=baike) |
| --- | --- | --- | --- | --- | --- | --- |
|  |

<https://baike.baidu.com/item/>慢性功能性便秘?fromModule=lemma_search-box

4/5

2022/12/14 10:48

[女](http://baike.baidu.com/l/WWoXYu7P)  [口](javascript:void(0);)

慢性功能性便秘_百度百科

| 岔 搜索发现  [功能性便秘怎么治疗](https://www.baidu.com/s?word=%E5%8A%9F%E8%83%BD%E6%80%A7%E4%BE%BF%E7%A7%98%E6%80%8E%E4%B9%88%E6%B2%BB%E7%96%97&tn=SE_baikepcxf02_fcetbk02&pos=baike_pc_turbo_1767&ori_sid=00bb3586ac9751ad)  [为什么老是便秘](https://www.baidu.com/s?word=%E4%B8%BA%E4%BB%80%E4%B9%88%E8%80%81%E6%98%AF%E4%BE%BF%E7%A7%98&tn=SE_baikepcxf02_fcetbk02&pos=baike_pc_turbo_1767&ori_sid=00bb3586ac9751ad) | [便秘的解决方法](https://www.baidu.com/s?word=%E4%BE%BF%E7%A7%98%E7%9A%84%E8%A7%A3%E5%86%B3%E6%96%B9%E6%B3%95&tn=SE_baikepcxf02_fcetbk02&pos=baike_pc_turbo_1767&ori_sid=00bb3586ac9751ad)  [预防便秘的方法有哪些](https://www.baidu.com/s?word=%E9%A2%84%E9%98%B2%E4%BE%BF%E7%A7%98%E7%9A%84%E6%96%B9%E6%B3%95%E6%9C%89%E5%93%AA%E4%BA%9B&tn=SE_baikepcxf02_fcetbk02&pos=baike_pc_turbo_1767&ori_sid=00bb3586ac9751ad) | [慢性便秘的症状](https://www.baidu.com/s?word=%E6%85%A2%E6%80%A7%E4%BE%BF%E7%A7%98%E7%9A%84%E7%97%87%E7%8A%B6&tn=SE_baikepcxf02_fcetbk02&pos=baike_pc_turbo_1767&ori_sid=00bb3586ac9751ad)  [突然便秘怎么办](https://www.baidu.com/s?word=%E7%AA%81%E7%84%B6%E4%BE%BF%E7%A7%98%E6%80%8E%E4%B9%88%E5%8A%9E&tn=SE_baikepcxf02_fcetbk02&pos=baike_pc_turbo_1767&ori_sid=00bb3586ac9751ad) | [闭合性粉刺怎么排出来](https://www.baidu.com/s?word=%E9%97%AD%E5%90%88%E6%80%A7%E7%B2%89%E5%88%BA%E6%80%8E%E4%B9%88%E6%8E%92%E5%87%BA%E6%9D%A5&tn=SE_baikepcxf02_fcetbk02&pos=baike_pc_turbo_1767&ori_sid=00bb3586ac9751ad) [便秘怎么办快速解决](https://www.baidu.com/s?word=%E4%BE%BF%E7%A7%98%E6%80%8E%E4%B9%88%E5%8A%9E%E5%BF%AB%E9%80%9F%E8%A7%A3%E5%86%B3&tn=SE_baikepcxf02_fcetbk02&pos=baike_pc_turbo_1767&ori_sid=00bb3586ac9751ad) | [乳果糖治疗便秘](https://www.baidu.com/s?word=%E4%B9%B3%E6%9E%9C%E7%B3%96%E6%B2%BB%E7%96%97%E4%BE%BF%E7%A7%98&tn=SE_baikepcxf02_fcetbk02&pos=baike_pc_turbo_1767&ori_sid=00bb3586ac9751ad)  [便秘有什么危害](https://www.baidu.com/s?word=%E4%BE%BF%E7%A7%98%E6%9C%89%E4%BB%80%E4%B9%88%E5%8D%B1%E5%AE%B3&tn=SE_baikepcxf02_fcetbk02&pos=baike_pc_turbo_1767&ori_sid=00bb3586ac9751ad) |
| --- | --- | --- | --- | --- |

Q 新手上路

我有疑问

投诉建议

[成长任务](https://baike.baidu.com/usercenter/tasks#guide) [编辑规则](https://baike.baidu.com/help#main06)

[编辑入门](https://baike.baidu.com/help#main01) [内容质疑](javascript:void(0);)

[本人编辑](https://baike.baidu.com/item/%E7%99%BE%E5%BA%A6%E7%99%BE%E7%A7%91%EF%BC%9A%E6%9C%AC%E4%BA%BA%E8%AF%8D%E6%9D%A1%E7%BC%96%E8%BE%91%E6%9C%8D%E5%8A%A1/22442459?bk_fr=pcFooter) [官方贴吧](http://tieba.baidu.com/f?ie=utf-8&fr=bks0000&kw=%E7%99%BE%E5%BA%A6%E7%99%BE%E7%A7%91)

[在线客服](http://zhiqiu.baidu.com/baike/passport/html/baikechat.html)

[意见反馈](javascript:void(0);)

[举报不良信息](http://help.baidu.com/newadd?word=%E6%85%A2%E6%80%A7%E5%8A%9F%E8%83%BD%E6%80%A7%E4%BE%BF%E7%A7%98&&submit_link=https%3A%2F%2Fbaike.baidu.com%2Fitem%2F%25E6%2585%25A2%25E6%2580%25A7%25E5%258A%259F%25E8%2583%25BD%25E6%2580%25A7%25E4%25BE%25BF%25E7%25A7%2598%3FfromModule%3Dlemma_search-box&prod_id=10&category=1) [投诉侵权信息](http://help.baidu.com/newadd?word=%E6%85%A2%E6%80%A7%E5%8A%9F%E8%83%BD%E6%80%A7%E4%BE%BF%E7%A7%98&&submit_link=https%3A%2F%2Fbaike.baidu.com%2Fitem%2F%25E6%2585%25A2%25E6%2580%25A7%25E5%258A%259F%25E8%2583%25BD%25E6%2580%25A7%25E4%25BE%25BF%25E7%25A7%2598%3FfromModule%3Dlemma_search-box&prod_id=10&category=6)

[未通过词条申诉](http://help.baidu.com/newadd?word=%E6%85%A2%E6%80%A7%E5%8A%9F%E8%83%BD%E6%80%A7%E4%BE%BF%E7%A7%98&&submit_link=https%3A%2F%2Fbaike.baidu.com%2Fitem%2F%25E6%2585%25A2%25E6%2580%25A7%25E5%258A%259F%25E8%2583%25BD%25E6%2580%25A7%25E4%25BE%25BF%25E7%25A7%2598%3FfromModule%3Dlemma_search-box&prod_id=10&category=2)

[封禁查询与解封](http://help.baidu.com/newadd?word=%E6%85%A2%E6%80%A7%E5%8A%9F%E8%83%BD%E6%80%A7%E4%BE%BF%E7%A7%98&&submit_link=https%3A%2F%2Fbaike.baidu.com%2Fitem%2F%25E6%2585%25A2%25E6%2580%25A7%25E5%258A%259F%25E8%2583%25BD%25E6%2580%25A7%25E4%25BE%25BF%25E7%25A7%2598%3FfromModule%3Dlemma_search-box&prod_id=10&category=5)

©2022 Baidu [使用百度前必读](http://www.baidu.com/duty/) | [百科协议](http://help.baidu.com/question?prod_en=baike&class=89&id=1637) | [隐私政策](http://help.baidu.com/question?prod_id=10&class=690&id=1001779) | [百度百科合作平台](https://baike.baidu.com/operation/cooperation) | 京ICP证030173号

[京公网安备11000002000001号](http://www.beian.gov.cn/portal/registerSystemInfo?recordcode=11000002000001)

<https://baike.baidu.com/item/>慢性功能性便秘?fromModule=lemma_search-box

5/5
