# Supplementary material for: Assessment of medical information on irritable bowel syndrome information in Wikipedia and Baidu Encyclopedia: comparative study
Source: PeerJ. 2024 May 24;12:e17264. doi: 10.7717/peerj.17264 (PMC11129691; doi:10.7717/peerj.17264)
Supplement: Data S1 [file peerj-12-17264-s001.zip › σÄƒσoïμò░μì«/Baidu/Baidu-English/10-σèƒΦâ╜μÇoΦà╣μ│╗_τÖ╛σ║aτÖ╛τoæ.docx]

| 2022/12/14 10:50  [网页](https://www.baidu.com/) | [新闻](http://news.baidu.com/) | 功能性腹泻_百度百科  [贴吧](https://tieba.baidu.com/) [知道](https://zhidao.baidu.com/) [网盘](https://pan.baidu.com/?from=1027327l) [图片](http://image.baidu.com/) | [视频](http://v.baidu.com/) | [地图](http://map.baidu.com/) | [文库](https://wenku.baidu.com/) | 百科 | [百度首页](http://www.baidu.com/) [登录](javascript:;) |
| --- | --- | --- | --- | --- | --- | --- | --- |

| [岔](https://baike.baidu.com/) | \| 功能性腹泻 \| 进入词条 \| \| --- \| --- \| | \| 全站搜索 \| \| --- \| | [帮助](https://baike.baidu.com/help) |
| --- | --- | --- | --- | --- | --- | --- |
| 近期有不法分子冒充百度百科官方人员，以删除词条为由威胁并敲诈相关企业。在此严正声明：百度百科是免费编辑平台，绝不存在收费代编服务，请勿上当受骗！ [详情>>](https://baike.baidu.com/common/declaration) | | | |
| [首页](https://baike.baidu.com/) 秒懂百科 特色百科 用户 知识专题 权威合作 [口下载百科APP](https://baike.baidu.com/wapui/subpage/baikeappdownload?sfrom=pc_lemmapage_navigation) [2 个](https://baike.baidu.com/usercenter) | | | |

| 功能性腹泻 | \| [小播报](javascript:;) \| \| --- \| | \| [c编辑](javascript:;) \| \| --- \| | \| [上传视频](javascript:;) \| \| --- \| | . 收藏 [山 59](javascript:void(0);) 24 |  | |
| --- | --- | --- | --- | --- | --- | --- | --- | --- | --- |
|  |  |  |  |  |  | 科普中国  致力于权威的科学传播 |
| Functional diarrhea refers to a syndrome in which loose or watery stools are persistent or recurrent, without symptoms of abdominal pain or abdominal discomfort. Diarrhea without bacterial, viral, or parasitic infections, generally caused by gastrointestinal dysfunction. The occurrence of this disease may be related to mental factors, intestinal motor sensory dysfunction, autonomic dysfunction, etc. Various examinations have not found organic lesions causing diarrhea.  外文名 functional diarrhea 常见病因 不明，可能与精神因素、肠运动感觉功能异展…开 、  就诊科室 消化内科 常见症状 不伴有腹痛或腹部不适的少量多次的排便 | | | | | 本词条认证专家为  韩英 丨主任医师   \|  \| \| --- \|   北京军区总医院 消化内科  审核 | |
|  |  |  |  |  |  | |

| \| 目录 \| 1 [病因](#_bookmark1)  2 [临床表现](#_bookmark2)  3 [检查](#_bookmark3)  4 [诊断](#_bookmark4)  5 [治疗](#_bookmark5) \| \| --- \| --- \| | | | 常见病因 | 不明，可能与精神因素、肠运动感觉功能异常、自主 神经功能紊乱等有关  不伴有腹痛或腹部不适的少量多次的排便 | [女疊 口](javascript:void(0);)   \| 词条统计  浏览次数： 147939次  编辑次数： 8次[历史版本](https://baike.baidu.com/historylist/%E5%8A%9F%E8%83%BD%E6%80%A7%E8%85%B9%E6%B3%BB/1309776)  最近更新： [卫计委科普项目](https://baike.baidu.com/usercenter/userpage?uk=x54mmYyOgfn1hjDv9q0UTQ&from=lemma) ( 2017-10-0  3)  突出贡献榜  [andnage](https://baike.baidu.com/usercenter/userpage?uk=sbIszXoccXLdwjYitVh0pw&from=lemma) \| \| --- \| |
| --- | --- | --- | --- | --- | --- | --- | --- | --- |
|  | 基本信息  外文名  就诊科室 | functional diarrhea  消化内科 |  |  |  |
|  |  |  | 常见症状 |  |  |
| 病因 | | |  |  |  |
|  |  |  |  | [小 播报c编辑](javascript:;) |  |
| Organic lesions that have diarrhea symptoms but have not been found to cause diarrhea after various examinations are called functional diarrhea. Its etiology is complex, including irritable bowel syndrome of unknown cause and secondary enteral lactase deficiency with a known cause. Although the cause is still unknown, studies have proved that due to mental stress, it can cause changes in colonic motility and diarrhea and other symptoms.  临床表现  [小 播报c编辑](javascript:;)  Patients with functional diarrhea often present with a small number of frequent defecation without abdominal pain or abdominal discomfort, but they need to be differentiated from "pseudodiarrhea", which is characterized by frequent defecation and a sense of urgency to defecate, but every Every time I defecate.  检查  [小 播报c编辑](javascript:;)  Patients with suspected diarrhea should have CBC, erythrocyte sedimentation rate (ESR), and c-reactive protein (CRP) tests. Measurement of serum ferritin and folate can help differentiate it from small bowel malabsorption. If suspicion of IBD or small bowel malabsorption is high, imaging of the gastrointestinal tract or gastrointestinal endoscopy should be performed. In patients with severe watery diarrhea, diarrhea due to endocrine diseases, such as intestinal vasoactive peptide (VIP) tumors and 5-HIAA, should be excluded.。  诊断  [小 播报c编辑](javascript:;)  To exclude organic lesions, functional diarrhea can be considered for diagnosis when the following conditions are met: (1) at least 75% of bowel movements are loose stool or watery stool without abdominal pain; (2) symptoms have been present for at least 6 months before diagnosis, and the above diagnostic criteria have been met in the past 3 months.  It is important to note that diarrhea is only a non-specific intestinal symptom, and there are many causes of diarrhea. Functional diarrhea must be distinguished from diarrhea caused by intestinal organic diseases such as intestinal infectious diseases (chronic bacterial dysentery, intestinal tuberculosis, parasitic infectious diarrhea, etc.), IBD, radiation enteritis, colon tumors, small bowel malabsorption, and VIP tumors. Endoscopy, blood biochemistry, and other related tests help to make a differential diagnosis.  治疗  [小 播报c编辑](javascript:;) | | | | | |

<https://baike.baidu.com/item/>功能性腹泻?fromModule=lemma_search-box

1/2

2022/12/14 10:50

| 岔 搜索发现  [功能性胃病](https://www.baidu.com/s?word=%E5%8A%9F%E8%83%BD%E6%80%A7%E8%83%83%E7%97%85&tn=SE_baikepcxf02_fcetbk02&pos=baike_pc_turbo_1767&ori_sid=00bb359fb29db277)  [治拉肚子的偏方](https://www.baidu.com/s?word=%E6%B2%BB%E6%8B%89%E8%82%9A%E5%AD%90%E7%9A%84%E5%81%8F%E6%96%B9&tn=SE_baikepcxf02_fcetbk02&pos=baike_pc_turbo_1767&ori_sid=00bb359fb29db277) | [腹泻的症状](https://www.baidu.com/s?word=%E8%85%B9%E6%B3%BB%E7%9A%84%E7%97%87%E7%8A%B6&tn=SE_baikepcxf02_fcetbk02&pos=baike_pc_turbo_1767&ori_sid=00bb359fb29db277)  [腹泻的治疗方法](https://www.baidu.com/s?word=%E8%85%B9%E6%B3%BB%E7%9A%84%E6%B2%BB%E7%96%97%E6%96%B9%E6%B3%95&tn=SE_baikepcxf02_fcetbk02&pos=baike_pc_turbo_1767&ori_sid=00bb359fb29db277) | [肠易激综合征会自愈吗](https://www.baidu.com/s?word=%E8%82%A0%E6%98%93%E6%BF%80%E7%BB%BC%E5%90%88%E5%BE%81%E4%BC%9A%E8%87%AA%E6%84%88%E5%90%97&tn=SE_baikepcxf02_fcetbk02&pos=baike_pc_turbo_1767&ori_sid=00bb359fb29db277) [腹泻出血怎么回事](https://www.baidu.com/s?word=%E8%85%B9%E6%B3%BB%E5%87%BA%E8%A1%80%E6%80%8E%E4%B9%88%E5%9B%9E%E4%BA%8B&tn=SE_baikepcxf02_fcetbk02&pos=baike_pc_turbo_1767&ori_sid=00bb359fb29db277) | [拉肚子拉水止泻小妙招](https://www.baidu.com/s?word=%E6%8B%89%E8%82%9A%E5%AD%90%E6%8B%89%E6%B0%B4%E6%AD%A2%E6%B3%BB%E5%B0%8F%E5%A6%99%E6%8B%9B&tn=SE_baikepcxf02_fcetbk02&pos=baike_pc_turbo_1767&ori_sid=00bb359fb29db277) [功能性腹泻怎么治](https://www.baidu.com/s?word=%E5%8A%9F%E8%83%BD%E6%80%A7%E8%85%B9%E6%B3%BB%E6%80%8E%E4%B9%88%E6%B2%BB&tn=SE_baikepcxf02_fcetbk02&pos=baike_pc_turbo_1767&ori_sid=00bb359fb29db277) | [什么是腹泻](https://www.baidu.com/s?word=%E4%BB%80%E4%B9%88%E6%98%AF%E8%85%B9%E6%B3%BB&tn=SE_baikepcxf02_fcetbk02&pos=baike_pc_turbo_1767&ori_sid=00bb359fb29db277)  [腹胀便秘怎么治疗](https://www.baidu.com/s?word=%E8%85%B9%E8%83%80%E4%BE%BF%E7%A7%98%E6%80%8E%E4%B9%88%E6%B2%BB%E7%96%97&tn=SE_baikepcxf02_fcetbk02&pos=baike_pc_turbo_1767&ori_sid=00bb359fb29db277) |
| --- | --- | --- | --- | --- |
|  | | | | |

[口](javascript:void(0);)

功能性腹泻_百度百科

| Patients with functional diarrhea should be treated with individualized treatment and detailed explanations of the patient's diarrhea due to stressful events can help relieve anxiety.  1. Dietary therapy  Ask the patient about their eating habits in detail. Pasta, dairy products, citrus fruits, eggs, onions, caffeine, and alcohol may cause symptoms in patients with functional diarrhea. Therefore, restricting these foods will help relieve the patient's diarrhea symptoms.  2. Drug treatment  If lifestyle changes are ineffective, medication may be used. Commonly used antidiarrheal drugs are loperamide and phenethylpiperidine, which exert antidiarrheal effects by binding to opioid receptors and preventing the release of acetylcholine from cholinergic nerve endings. Loperamide also has a non-opioid effect to achieve antidiarrheal purposes. | |
| --- | --- |
| 学术论文 | 内容来自 |
| [李琳，李岩. 肠道菌群失调与功能性腹泻．](https://xueshu.baidu.com/usercenter/paper/show?paperid=620c4c0ed0fb8d935b7f80c469bb44f7&tn=SE_baiduxueshu_c1gjeupa&ie=utf-8&site=baike) 《胃肠病学和肝病学杂志》， 2014  [李红梅，梁浩，唐湖泉，李祥. 匹维溴铵对小鼠功能性腹泻及便秘的治疗作用．](https://xueshu.baidu.com/usercenter/paper/show?paperid=46d6c5256b6e170df77584bf8cb7973a&tn=SE_baiduxueshu_c1gjeupa&ie=utf-8&site=baike) 《世界华人消化杂志》， 1998  [李琳，李岩. 整肠生联合常乐康治疗伴肠道菌群失调功能性腹泻的疗效观察．](https://xueshu.baidu.com/usercenter/paper/show?paperid=c33ca1f2e7997d97344df9f36f247db2&tn=SE_baiduxueshu_c1gjeupa&ie=utf-8&site=baike) 《 CNKI》， 2014  [许继宗，李玉华，张喆，张波. 体感五行音乐疗法联合参苓白术散治疗功能性腹泻疗效观察．](https://xueshu.baidu.com/usercenter/paper/show?paperid=7fdf45e8c158297022072a08332931db&tn=SE_baiduxueshu_c1gjeupa&ie=utf-8&site=baike) 《 CNKI》， 2012  [马秀丽，李正军. 参苓白术散联合理中汤治疗慢性功能性腹泻35例．](https://xueshu.baidu.com/usercenter/paper/show?paperid=9c80887f160c6afc1bd5970093ae7264&tn=SE_baiduxueshu_c1gjeupa&ie=utf-8&site=baike) 《陕西中医》， 2011  [查看全部](https://xueshu.baidu.com/s?wd=intitle%3A%28%E5%8A%9F%E8%83%BD%E6%80%A7%E8%85%B9%E6%B3%BB%29&tn=SE_baiduxueshu_c1gjeupa&ie=utf-8&sc_from=pingtai6&site=baike) | |

| 猜你喜欢 | [腹泻贴多少钱一盒零售，潮流新品，好货热卖，更](http://www.baidu.com/baidu.php?url=Ks00000EAMrnlPLIyW15gPJnEGUHQh0d6RV8AxERo18JrH4TgzWQNa7o-Mo8nKInb4TnBiU0XRBadvSqfgxzTGduEewgRz917ijYbinXTwsqJjr1d_a_S8ckmupJriveDbdws1fdMMsxBIaFjGRvjnAdARf00iWPMBPrtL2WRHzmreq-KBafsauqN7sGOFEIp2KF9DvYzaQ5qd0iqiDDf2TB8XMb.Db_iHF8xnhA94wEYL_SNK-deQbfHgI3ynDgg6msw5I7AMHdey5Z_otIv8EWj3q-Xek8dqTUAMHz4rMG34nheuztIdMugbzTEZF83e5ZGzIUvZO-OtZeOL3Xho1Ce5ZHOkxWqqgYFlk_IIU7Na9WWstxU9zxgjbS8aB17I4RAgg_8w9zxyPrMjbSVaSPQnYPyZWqubltXQjkSyMHz4rMG34nheuztIdMugbzTEZF83e5ZGzmTMHvGYTjGo_5Z4mThe1vmTheu8s4PLoEs4PLMY3Thed33ThedqTrHI4enrHIlqhZF8qX1jbLXXejbLXL_s4PvOv3The1LmThed_s4PvSEjbLXXOjbLUthZF8qptrHI4qhZF8qIhZF8vymThe1tTrHIEqTrHIEzmThe1L3Thexl3ThedlTrHI4e_rHIEenrHIexPHReiM-kl-9h9mzyUO7f0.U1Yk0ZDq8pUDLPjR8QMf86KY5UB4YQonSUU6zIMZoV2e8VjK3toU0A-V5HDzPWc0Iybq0ZKGujYzn0KWpyfqP1c0mhbqn10k0AuY5H00TA6qn0KET1Ys0AFL5H00UMfqn0K1XWY0ThIYmyTqn0K8IM0qna3snj0snj0sn0K-ThTqn0KYTh7buHYs0AFbpyfqnW77fbc4nYu7wWFDnRwArj97fYfYrD7DrRwKPDnvnbD0uAPWujY0mgPxpywW5gK1QyIlpZ940ATqILP8TsKzIjY1rHf0TgKGujYs0Z7Wpyfqn0K9mWYsg100TZ0qn0KVIZ0qn0KbuAqs5H00ThCqn0KYIgnqnHTdPWm4nH0YPWnvnjTLPjRzPjc0mynqnfKsUWYs0ZK9I7qhUA7M5H00ugPY5H00ugwGujYVnfK9TLKWm1Ys0ZNspy4Wm1Ys0AuWIgfqn0K9uAu_myTqnfKLuMFEUHY0mMfqnfKzug7Y5HDvP104rjmkrHbzPHm0Tv-b5H0smhc1PyDsmWFWPAN9njc0ULfqn0KETMKY5H0WnaPDw-fWnansc10Wna3sc10WwDuRc10WwDuR0AVG5H00UgfqnW0vn6KVm1YzPWTYnW64nH6vn0KVmdqhThqV5H00uA78IyF-gLK_my4GuZnqn0K9uZ745UB4YQonSfK9uZ7Y5H00pgPWUjYs0Z7VIjYs0A7bgLPEIgFWuHYznzPkIyNzXNqkIyNzXNqsmzPxgdqxTAP8TzPkIyNzXiP-TvdVT-qvnfKWThnqPH0knj6&us=newvui&ai=0_429107157_1_1&word=&ck=0.0.0.0.0.0.0.0&shh=baike.baidu.com)…  [腹泻贴多少钱一盒零售，购物上淘宝，优选材质，用的舒心!在线](http://www.baidu.com/baidu.php?url=Ks00000EAMrnlPLIyW15gPJnEGUHQh0d6RV8AxERo18JrH4TgzWQNa7o-Mo8nKInb4TnBiU0XRBadvSqfgxzTGduEewgRz917ijYbinXTwsqJjr1d_a_S8ckmupJriveDbdws1fdMMsxBIaFjGRvjnAdARf00iWPMBPrtL2WRHzmreq-KBafsauqN7sGOFEIp2KF9DvYzaQ5qd0iqiDDf2TB8XMb.Db_iHF8xnhA94wEYL_SNK-deQbfHgI3ynDgg6msw5I7AMHdey5Z_otIv8EWj3q-Xek8dqTUAMHz4rMG34nheuztIdMugbzTEZF83e5ZGzIUvZO-OtZeOL3Xho1Ce5ZHOkxWqqgYFlk_IIU7Na9WWstxU9zxgjbS8aB17I4RAgg_8w9zxyPrMjbSVaSPQnYPyZWqubltXQjkSyMHz4rMG34nheuztIdMugbzTEZF83e5ZGzmTMHvGYTjGo_5Z4mThe1vmTheu8s4PLoEs4PLMY3Thed33ThedqTrHI4enrHIlqhZF8qX1jbLXXejbLXL_s4PvOv3The1LmThed_s4PvSEjbLXXOjbLUthZF8qptrHI4qhZF8qIhZF8vymThe1tTrHIEqTrHIEzmThe1L3Thexl3ThedlTrHI4e_rHIEenrHIexPHReiM-kl-9h9mzyUO7f0.U1Yk0ZDq8pUDLPjR8QMf86KY5UB4YQonSUU6zIMZoV2e8VjK3toU0A-V5HDzPWc0Iybq0ZKGujYzn0KWpyfqP1c0mhbqn10k0AuY5H00TA6qn0KET1Ys0AFL5H00UMfqn0K1XWY0ThIYmyTqn0K8IM0qna3snj0snj0sn0K-ThTqn0KYTh7buHYs0AFbpyfqnW77fbc4nYu7wWFDnRwArj97fYfYrD7DrRwKPDnvnbD0uAPWujY0mgPxpywW5gK1QyIlpZ940ATqILP8TsKzIjY1rHf0TgKGujYs0Z7Wpyfqn0K9mWYsg100TZ0qn0KVIZ0qn0KbuAqs5H00ThCqn0KYIgnqnHTdPWm4nH0YPWnvnjTLPjRzPjc0mynqnfKsUWYs0ZK9I7qhUA7M5H00ugPY5H00ugwGujYVnfK9TLKWm1Ys0ZNspy4Wm1Ys0AuWIgfqn0K9uAu_myTqnfKLuMFEUHY0mMfqnfKzug7Y5HDvP104rjmkrHbzPHm0Tv-b5H0smhc1PyDsmWFWPAN9njc0ULfqn0KETMKY5H0WnaPDw-fWnansc10Wna3sc10WwDuRc10WwDuR0AVG5H00UgfqnW0vn6KVm1YzPWTYnW64nH6vn0KVmdqhThqV5H00uA78IyF-gLK_my4GuZnqn0K9uZ745UB4YQonSfK9uZ7Y5H00pgPWUjYs0Z7VIjYs0A7bgLPEIgFWuHYznzPkIyNzXNqkIyNzXNqsmzPxgdqxTAP8TzPkIyNzXiP-TvdVT-qvnfKWThnqPH0knj6&us=newvui&ai=0_429107157_1_1&word=&ck=0.0.0.0.0.0.0.0&shh=baike.baidu.com)  [下单，省时省力。你要的好货尽在淘宝网，安心享受网购乐趣!](http://www.baidu.com/baidu.php?url=Ks00000EAMrnlPLIyW15gPJnEGUHQh0d6RV8AxERo18JrH4TgzWQNa7o-Mo8nKInb4TnBiU0XRBadvSqfgxzTGduEewgRz917ijYbinXTwsqJjr1d_a_S8ckmupJriveDbdws1fdMMsxBIaFjGRvjnAdARf00iWPMBPrtL2WRHzmreq-KBafsauqN7sGOFEIp2KF9DvYzaQ5qd0iqiDDf2TB8XMb.Db_iHF8xnhA94wEYL_SNK-deQbfHgI3ynDgg6msw5I7AMHdey5Z_otIv8EWj3q-Xek8dqTUAMHz4rMG34nheuztIdMugbzTEZF83e5ZGzIUvZO-OtZeOL3Xho1Ce5ZHOkxWqqgYFlk_IIU7Na9WWstxU9zxgjbS8aB17I4RAgg_8w9zxyPrMjbSVaSPQnYPyZWqubltXQjkSyMHz4rMG34nheuztIdMugbzTEZF83e5ZGzmTMHvGYTjGo_5Z4mThe1vmTheu8s4PLoEs4PLMY3Thed33ThedqTrHI4enrHIlqhZF8qX1jbLXXejbLXL_s4PvOv3The1LmThed_s4PvSEjbLXXOjbLUthZF8qptrHI4qhZF8qIhZF8vymThe1tTrHIEqTrHIEzmThe1L3Thexl3ThedlTrHI4e_rHIEenrHIexPHReiM-kl-9h9mzyUO7f0.U1Yk0ZDq8pUDLPjR8QMf86KY5UB4YQonSUU6zIMZoV2e8VjK3toU0A-V5HDzPWc0Iybq0ZKGujYzn0KWpyfqP1c0mhbqn10k0AuY5H00TA6qn0KET1Ys0AFL5H00UMfqn0K1XWY0ThIYmyTqn0K8IM0qna3snj0snj0sn0K-ThTqn0KYTh7buHYs0AFbpyfqnW77fbc4nYu7wWFDnRwArj97fYfYrD7DrRwKPDnvnbD0uAPWujY0mgPxpywW5gK1QyIlpZ940ATqILP8TsKzIjY1rHf0TgKGujYs0Z7Wpyfqn0K9mWYsg100TZ0qn0KVIZ0qn0KbuAqs5H00ThCqn0KYIgnqnHTdPWm4nH0YPWnvnjTLPjRzPjc0mynqnfKsUWYs0ZK9I7qhUA7M5H00ugPY5H00ugwGujYVnfK9TLKWm1Ys0ZNspy4Wm1Ys0AuWIgfqn0K9uAu_myTqnfKLuMFEUHY0mMfqnfKzug7Y5HDvP104rjmkrHbzPHm0Tv-b5H0smhc1PyDsmWFWPAN9njc0ULfqn0KETMKY5H0WnaPDw-fWnansc10Wna3sc10WwDuRc10WwDuR0AVG5H00UgfqnW0vn6KVm1YzPWTYnW64nH6vn0KVmdqhThqV5H00uA78IyF-gLK_my4GuZnqn0K9uZ745UB4YQonSfK9uZ7Y5H00pgPWUjYs0Z7VIjYs0A7bgLPEIgFWuHYznzPkIyNzXNqkIyNzXNqsmzPxgdqxTAP8TzPkIyNzXiP-TvdVT-qvnfKWThnqPH0knj6&us=newvui&ai=0_429107157_1_1&word=&ck=0.0.0.0.0.0.0.0&shh=baike.baidu.com)  [simba.taobao.com](http://www.baidu.com/baidu.php?url=Ks00000EAMrnlPLIyW15gPJnEGUHQh0d6RV8AxERo18JrH4TgzWQNa7o-Mo8nKInb4TnBiU0XRBadvSqfgxzTGduEewgRz917ijYbinXTwsqJjr1d_a_S8ckmupJriveDbdws1fdMMsxBIaFjGRvjnAdARf00iWPMBPrtL2WRHzmreq-KBafsauqN7sGOFEIp2KF9DvYzaQ5qd0iqiDDf2TB8XMb.Db_iHF8xnhA94wEYL_SNK-deQbfHgI3ynDgg6msw5I7AMHdey5Z_otIv8EWj3q-Xek8dqTUAMHz4rMG34nheuztIdMugbzTEZF83e5ZGzIUvZO-OtZeOL3Xho1Ce5ZHOkxWqqgYFlk_IIU7Na9WWstxU9zxgjbS8aB17I4RAgg_8w9zxyPrMjbSVaSPQnYPyZWqubltXQjkSyMHz4rMG34nheuztIdMugbzTEZF83e5ZGzmTMHvGYTjGo_5Z4mThe1vmTheu8s4PLoEs4PLMY3Thed33ThedqTrHI4enrHIlqhZF8qX1jbLXXejbLXL_s4PvOv3The1LmThed_s4PvSEjbLXXOjbLUthZF8qptrHI4qhZF8qIhZF8vymThe1tTrHIEqTrHIEzmThe1L3Thexl3ThedlTrHI4e_rHIEenrHIexPHReiM-kl-9h9mzyUO7f0.U1Yk0ZDq8pUDLPjR8QMf86KY5UB4YQonSUU6zIMZoV2e8VjK3toU0A-V5HDzPWc0Iybq0ZKGujYzn0KWpyfqP1c0mhbqn10k0AuY5H00TA6qn0KET1Ys0AFL5H00UMfqn0K1XWY0ThIYmyTqn0K8IM0qna3snj0snj0sn0K-ThTqn0KYTh7buHYs0AFbpyfqnW77fbc4nYu7wWFDnRwArj97fYfYrD7DrRwKPDnvnbD0uAPWujY0mgPxpywW5gK1QyIlpZ940ATqILP8TsKzIjY1rHf0TgKGujYs0Z7Wpyfqn0K9mWYsg100TZ0qn0KVIZ0qn0KbuAqs5H00ThCqn0KYIgnqnHTdPWm4nH0YPWnvnjTLPjRzPjc0mynqnfKsUWYs0ZK9I7qhUA7M5H00ugPY5H00ugwGujYVnfK9TLKWm1Ys0ZNspy4Wm1Ys0AuWIgfqn0K9uAu_myTqnfKLuMFEUHY0mMfqnfKzug7Y5HDvP104rjmkrHbzPHm0Tv-b5H0smhc1PyDsmWFWPAN9njc0ULfqn0KETMKY5H0WnaPDw-fWnansc10Wna3sc10WwDuRc10WwDuR0AVG5H00UgfqnW0vn6KVm1YzPWTYnW64nH6vn0KVmdqhThqV5H00uA78IyF-gLK_my4GuZnqn0K9uZ745UB4YQonSfK9uZ7Y5H00pgPWUjYs0Z7VIjYs0A7bgLPEIgFWuHYznzPkIyNzXNqkIyNzXNqsmzPxgdqxTAP8TzPkIyNzXiP-TvdVT-qvnfKWThnqPH0knj6&us=newvui&ai=0_429107157_1_1&word=&ck=0.0.0.0.0.0.0.0&shh=baike.baidu.com) |
| --- | --- |

[女](javascript:void(0);)

Q 新手上路 我有疑问 投诉建议

[成长任务](https://baike.baidu.com/usercenter/tasks#guide) [编辑规则](https://baike.baidu.com/help#main06)

[编辑入门](https://baike.baidu.com/help#main01) [内容质疑](javascript:void(0);)

[本人编辑](https://baike.baidu.com/item/%E7%99%BE%E5%BA%A6%E7%99%BE%E7%A7%91%EF%BC%9A%E6%9C%AC%E4%BA%BA%E8%AF%8D%E6%9D%A1%E7%BC%96%E8%BE%91%E6%9C%8D%E5%8A%A1/22442459?bk_fr=pcFooter) [官方贴吧](http://tieba.baidu.com/f?ie=utf-8&fr=bks0000&kw=%E7%99%BE%E5%BA%A6%E7%99%BE%E7%A7%91)

[在线客服](http://zhiqiu.baidu.com/baike/passport/html/baikechat.html)

[意见反馈](javascript:void(0);)

[举报不良信息](http://help.baidu.com/newadd?word=%E5%8A%9F%E8%83%BD%E6%80%A7%E8%85%B9%E6%B3%BB&&submit_link=https%3A%2F%2Fbaike.baidu.com%2Fitem%2F%25E5%258A%259F%25E8%2583%25BD%25E6%2580%25A7%25E8%2585%25B9%25E6%25B3%25BB%3FfromModule%3Dlemma_search-box&prod_id=10&category=1) [投诉侵权信息](http://help.baidu.com/newadd?word=%E5%8A%9F%E8%83%BD%E6%80%A7%E8%85%B9%E6%B3%BB&&submit_link=https%3A%2F%2Fbaike.baidu.com%2Fitem%2F%25E5%258A%259F%25E8%2583%25BD%25E6%2580%25A7%25E8%2585%25B9%25E6%25B3%25BB%3FfromModule%3Dlemma_search-box&prod_id=10&category=6)

[未通过词条申诉](http://help.baidu.com/newadd?word=%E5%8A%9F%E8%83%BD%E6%80%A7%E8%85%B9%E6%B3%BB&&submit_link=https%3A%2F%2Fbaike.baidu.com%2Fitem%2F%25E5%258A%259F%25E8%2583%25BD%25E6%2580%25A7%25E8%2585%25B9%25E6%25B3%25BB%3FfromModule%3Dlemma_search-box&prod_id=10&category=2)

[封禁查询与解封](http://help.baidu.com/newadd?word=%E5%8A%9F%E8%83%BD%E6%80%A7%E8%85%B9%E6%B3%BB&&submit_link=https%3A%2F%2Fbaike.baidu.com%2Fitem%2F%25E5%258A%259F%25E8%2583%25BD%25E6%2580%25A7%25E8%2585%25B9%25E6%25B3%25BB%3FfromModule%3Dlemma_search-box&prod_id=10&category=5)

©2022 Baidu [使用百度前必读](http://www.baidu.com/duty/) | [百科协议](http://help.baidu.com/question?prod_en=baike&class=89&id=1637) | [隐私政策](http://help.baidu.com/question?prod_id=10&class=690&id=1001779) | [百度百科合作平台](https://baike.baidu.com/operation/cooperation) | 京ICP证030173号

[京公网安备11000002000001号](http://www.beian.gov.cn/portal/registerSystemInfo?recordcode=11000002000001)

<https://baike.baidu.com/item/>功能性腹泻?fromModule=lemma_search-box

2/2
