# Supplementary material for: Assessment of medical information on irritable bowel syndrome information in Wikipedia and Baidu Encyclopedia: comparative study
Source: PeerJ. 2024 May 24;12:e17264. doi: 10.7717/peerj.17264 (PMC11129691; doi:10.7717/peerj.17264)
Supplement: Data S1 [file peerj-12-17264-s001.zip › σÄƒσoïμò░μì«/Baidu/Baidu-English/1-IBS∩╝êΦéáμÿôμ┐Çτ╗╝σÉêσ╛ü∩╝ë_τÖ╛σ║aτÖ╛τoæ.docx]

2022/12/14 10:29

[网页](https://www.baidu.com/) [新闻](http://news.baidu.com/) [贴吧](https://tieba.baidu.com/) [知道](https://zhidao.baidu.com/) [网盘](https://pan.baidu.com/?from=1027327l) [图片](http://image.baidu.com/) [视频](http://v.baidu.com/) [地图](http://map.baidu.com/) [文库](https://wenku.baidu.com/) 百科 [百度首页](http://www.baidu.com/) [登录](javascript:;)

[小播报](javascript:;)

[c编辑](javascript:;)

[O讨论](https://baike.baidu.com/planet/talk?lemmaId=10530572&fromModule=lemma_right-issue-btn)

IBS

| IBS | 进入词条 |
| --- | --- |

[岔](https://baike.baidu.com/)

小 播报

[夕 编辑](javascript:;)

[回 讨论](https://baike.baidu.com/planet/talk?lemmaId=10530572)

[让 收藏](javascript:;)

[凸 赞](javascript:;)

IBS (肠易激综合征) _百度百科

[岔](https://baike.baidu.com/) 进入词条 [帮助](https://baike.baidu.com/help)

近期有不法分子冒充百度百科官方人员，以删除词条为由威胁并敲诈相关企业。在此严正声明：百度百科是免费编辑平台，绝不存在收费代编服务，请勿上当受骗！ [详情>>](https://baike.baidu.com/common/declaration)

[首页](https://baike.baidu.com/)

秒懂百科

特色百科

用户

知识专题

权威合作

[口下载百科APP](https://baike.baidu.com/wapui/subpage/baikeappdownload?sfrom=pc_lemmapage_navigation) [2 个](https://baike.baidu.com/usercenter)

**IBS**是一个[多义词](https://baike.baidu.com/item/%E7%99%BE%E5%BA%A6%E7%99%BE%E7%A7%91%EF%BC%9A%E5%A4%9A%E4%B9%89%E8%AF%8D)，请在下列[义项](https://baike.baidu.com/item/%E4%B9%89%E9%A1%B9)上选择浏览([共6个义项](https://baike.baidu.com/item/IBS?force=1)) [展开 、添加义项 +](javascript:;)

| [训上传视频](javascript:;) |
| --- |

肠易激综合征

什么是肠易激综合征？

02:22

| . 收藏 [山 178](javascript:void(0);) 吐 108  IBS is Irritable Bowel Syndrome (Irritable Bowel Syndrome, IBS) is a common functional Bowel disease, abdominal pain or discomfort as the main symptoms, defecation can change the good, often accompanied by Bowel habit change, lack of explain symptoms of abnormal morphology and biochemistry.  IBS is Irritable Bowel Syndrome (Irritable Bowel Syndrome, IBS) is a common functional Bowel disease, abdominal pain or discomfort as the main symptoms, defecation can change the good, often accompanied by Bowel habit change, lack of explain symptoms of abnormal morphology and biochemistry.Epidemiological studies have reported all over the world show that IBS is a frequently-occurring disease worldwide.Western population prevalence was 10% -20%, one of our community by Roman Ⅱ standard epidemiological survey population IBS prevalence was 5.7%, 22% of them had the symptoms of IBS and attendance.  肠易激综合症  irritable bowel syndrome,IBS  西医学名  外文名   \| 目录 \| 1 [病因](#_bookmark1)  2 [诊断](#_bookmark2)  3 [肠易激综合征](#_bookmark3)  4 [鉴别诊断](#_bookmark4)  5 [治疗](#_bookmark5) \| \| --- \| --- \|   口  病因  [小 播报c编辑](javascript:;)  The general cause is the result of the interaction between the body's stress response and psychological factors, different individuals may involve genetic, environmental, psychological, social and gastrointestinal infections and other factors, resulting in gastrointestinal motility changes, brain-gut axis interaction disorders, autonomic and hormonal changes, accompanied by mental disorders (such as panic, anxiety, post-traumatic stress disorder, etc.), sleep disorders and psychological coping disorders, stress life events can often lead to aggravation of symptoms, but the exact connection between psychological factors and IBS is not very clear Clear. Studies have found that 1/3 of IBS patients have a history of gastrointestinal infection, and the influence of psychopsychological factors on the pathogenesis of IBS has been emphasized at home and abroad, and more attention has been paid to the role of neuropeptides and related receptor functions in the pathogenesis of IBS; in recent years, people have strengthened the research on the link between IBS and inflammatory bowel disease (IBD), and a few scholars even believe that IBS is the early manifestation of IBD.  诊断  [小 播报c编辑](javascript:;)  Symptomatology is still used as clinical diagnosis and research  肠易激综合征  [小 播报c编辑](javascript:;)     \| Abdominal pain, abdominal distention, diarrhea, constipation  IBS    常见症状  英文简称  IBS的概述图(2张)[疊](javascript:void(0);) \| \| --- \| \|  \| \| 词条统计 \| \| 浏览次数： 871420次  编辑次数： 51次[历史版本](https://baike.baidu.com/historylist/IBS/10530572)  最近更新： [w_ou](https://baike.baidu.com/usercenter/userpage?uk=TY3CXj_hJSEcBfJBVP43Rg&from=lemma) ( 2021-01-26)  突出贡献榜  [shmily32123](https://baike.baidu.com/usercenter/userpage?uk=5Iw5RYbIVJ8rf4-PMPKeGQ&from=lemma) \|  \| **1** aci营养师 **12** 自己创建个  **2** sci文献 **13** 手机空号  **3** 营养师报考资 **14** 大专生怎么  **4** 二级心理咨询 **15** 意大利留学  **5** 电商怎么做 **16** 征文投稿  **6** 快速学日语 **17** 如何治脸瘫  **7** 战队logo设计 **18** 9733游戏平  **8** 哈佛大学申请 **19** 芝加哥留学  **9** 哈佛大学入学 **20** 怎么成为插  **10** 价格便宜的香 **21** 华师在职研  **11** 千锋教育 **22** 哈佛大学宿 \| \| --- \|   1) General criteria for IBS:  Have experienced recurrent abdominal pain or discomfort for at least 3 days each month in the last 3 months with 2 or more of the following:  (1) Improvement of symptoms after defecation.  (2) Accompanied by changes in the frequency of bowel movements.  (3) Accompanied by changes in fecal properties. |
| --- | --- | --- | --- | --- | --- | --- | --- |

<https://baike.baidu.com/item/IBS/10530572?fromModule=lemma_search-box>

1/4

2022/12/14 10:29

IBS (肠易激综合征) _百度百科

| 诊断标准建立于患者至少在诊断前的6个月内出现症状，并在最近的3个月持续存在，在观察期间疼痛(不适)症状的频率至  [岔](https://baike.baidu.com/)  小 播报  [编辑](javascript:;)  [回 讨论](https://baike.baidu.com/planet/talk?lemmaId=10530572)  [收藏](javascript:;)  [赞](javascript:;)  少一周2天。 |
| --- |
| 2) The following general symptoms of IBS support the diagnosis:  (1) Abnormal bowel frequency: a. ≤ 3 bowel movements per week or b. > 3 bowel movements per day.  (2) Abnormal fecal properties: c. lumpy stool/hard stool or d. loose stool/loose stool.  (3) Laborious bowel movements.  (4) Urgency to defecate or incomplete bowel movements.  (5) Discharge mucus.  (6) Bloating.  3) IBS subtypes are divided into the following subtypes according to the characteristics of feces:  (1) IBS constipation type (IBS-C): the proportion of hard or lumpy stool ≥ 25%, and the proportion of loose stool (mushy stool) or watery stool <25%.  (2) IBS diarrhea type (IBS-D): the proportion of loose stools (mushy stools) or watery stools ≥ 25%, and the proportion of hard or lumpy stools <25%.  (3) Mixed IBS (IBS-M): the proportion of hard or lumpy stools > 25%, and the proportion of loose stools (mushy stools) or watery stools ≥ 25%.  (4) Indeterminate IBS (IBS-U): The properties of feces do not meet any of the above criteria of IBS-C, D, M.  In addition to the need to focus on fecal traits in IBS, symptoms such as strained, urgency, and incomplete bowel movements should also be noted, and in most cases fecal traits (from  Dilapidated watery diarrhea to induration stool) can reflect the transit time of the bowel tube.  Previous key points emphasized in diagnosis were: reduced abdominal pain after defecation, increased frequency of stool in abdominal pain, and loose stool and marked bloating during onset of abdominal pain. It's still in use, but it's in  The value in differentiating organic bowel disease is limited and appropriate testing is required at the right time. Determining the diagnosis of IBS usually requires a detailed history and physical examination, depending on the patient  , targeted laboratory and auxiliary examinations. In order to explain all the symptoms as much as possible by the organic disease itself, mild organic diseases can also be associated with IBS. |
| 鉴别诊断  [小 播报编辑](javascript:;)  [女疊 口](javascript:void(0);)    The main diseases that need to be differentiated from IBS are inflammatory bowel disease, colorectal tumors, and IBS· D intolerance to lactose, bacterial overgrowth of small intestines, parasitic infection and other identification.  [小 播报编辑](javascript:;)  治疗  Individualized comprehensive treatment is emphasized here, that is, it should include psychopsychological and behavioral intervention, dietary modification and drug treatment, and the patient's treatment method and drug selection response  Symptomatic treatment varies from person to person. There are three categories of recommendations for treatment by the IBS expert group in Rome III:  1. Psychotherapy  Psychobehavioral therapy should be considered in patients with severe and stubborn symptoms that do not respond to general treatment and medical therapy. These include psychotherapy, cognitive therapy, hypnotherapy, and biofeedback  Wait.  2. Diet adjustment  Poor eating habits and dietary structure can exacerbate the symptoms of IBS. Therefore, a healthy, balanced diet can help reduce symptoms of gastrointestinal disorders. IBS patients  It is advisable to avoid:  (1) excessive diet;  (2) heavy alcohol consumption;  (3) caffeine;  (4) high-fat diet;  (5) Some vegetables with "gas-production" effect;  (6) Refined food and artificial food (constipation), sorbitol and fructose (diarrhea);  (7) Foods that are not tolerated (varies from individual to individual).  Increasing dietary fiber is mainly used in IBS patients with predominantly constipation, and the method of increasing fiber intake should be individualized.  3. Drug treatment  Mainly for patients with significant symptoms, due to the complex and variable symptoms of IBS patients and the complex relationship with the central and enteric nervous systems, the drug has efficacy and safety  Limitations can only work to a certain extent. Deschute is the representative gastrointestinal selective calcium antagonist among the most popular of all kinds of smooth muscle spasmolytics, used in dozens of countries;  Rowe (introduced with the Rome III standard) was so popular that its effectiveness and safety limited its use;In recent years, great attention has been paid to probiotic treatment |

<https://baike.baidu.com/item/IBS/10530572?fromModule=lemma_search-box>

2/4

2022/12/14 10:29

IBS (肠易激综合征) _百度百科

| [岔](https://baike.baidu.com/)  感性的药物(作用于各级神经调节的内脏敏感性)为研制的重点，现有的低剂量三环类和5羟色胺再摄取抑制剂治疗抗抑郁药有 一定的调节内脏敏感性，其长期治疗仍被强调，尤其有较顽固症状者。  小 播报  [编辑](javascript:;)  [回 讨论](https://baike.baidu.com/planet/talk?lemmaId=10530572)  [收藏](javascript:;)  [赞](javascript:;) |
| --- |
| Other non-drug treatments include psychotherapy, hypnotherapy, biofeedback therapy, behavioral therapy, etc., which have not been promoted by the IBS expert group in Rome III  Recommended, but cross-cutting with the above, also shows a certain efficacy, further research is needed.  Irritable bowel syndrome (IBS) is a common functional gastrointestinal condition characterized by abdominal pain or discomfort that improves with bowel movements, often accompanied by bowel habits and bowel movements  Morphological changes, constipation, and diarrhea may alternate, but morphological and biochemical abnormalities that explain symptoms are lacking. In the past, it was called "spastic colitis", "mucinous colitis" and "instability."  colitis" and so on. The patients of the disease are mostly young and middle-aged people aged 20-40, and most common in women; it has a tendency to recurrent attacks and is often prolonged. In terms of drug treatment, Western medicine does not have ideals  Drugs.  The incidence of irritable bowel syndrome in China is about 10%-20%, and the patients are mainly young and middle-aged. The exact mechanism of pathogenesis is still unclear, but psychopsychological factors contribute to the onset  is an important factor that seriously affects the quality of life of patients. Patients should seek medical attention promptly, and antispasmodics can be selected under the guidance of the doctor to relieve the corresponding symptoms; and antidiarrheal agents/laxatives should be improved  Symptoms of diarrhea/constipation; or modification of the beneficial gut flora with probiotics to reduce abdominal discomfort. Only by adjusting the diet structure and improving the emotional state can the goal of gradual recovery be achieved  Target. [1][词条图册 更多图册 >](https://baike.baidu.com/pic/IBS/10530572?fr=lemma)   \|  \| \| \| --- \| --- \| \| 概述图册(2) \| \| \|  \|  \|  \| **NRN** \| \| \| \| \| \| \| \| \| \| \| \| --- \| --- \| --- \| --- \| --- \| --- \| --- \| --- \| --- \| --- \| --- \| \|  \| ▪ \| [STV](http://baike.baidu.com/view/444125.htm) \| ▪ \| [HBC](http://baike.baidu.com/searchword/?word=HBC&pic=1&sug=1&enc=utf8) \| ▪ \| [RAB](http://baike.baidu.com/view/1082772.htm) \| ▪ [IBC](http://baike.baidu.com/view/1518618.htm) \| ▪ \| [TBC](http://baike.baidu.com/view/713117.htm) \| ▪ [ABS](http://baike.baidu.com/view/8910.htm) \| \|  \| ▪ \| [YBC](http://baike.baidu.com/view/609803.htm) \| ▪ \| [RFC](http://baike.baidu.com/view/6108.htm) \| ▪ \| [QR](http://baike.baidu.com/view/124747.htm) \| ▪ [LF](http://baike.baidu.com/view/3446937.htm) \| ▪ \| [IBS](http://baike.baidu.com/view/601942.htm) \| ▪ [CRT](http://baike.baidu.com/view/1190.htm) \| \|  \| ▪ \| [YBS](http://baike.baidu.com/searchword/?word=YBS&pic=1&sug=1&enc=utf8) \| ▪ \| [SBC](http://baike.baidu.com/view/259912.htm) \| ▪ \| [BSN](http://baike.baidu.com/view/805622.htm) \| ▪ [SBS](http://baike.baidu.com/view/306571.htm) \| ▪ \| [SF](http://baike.baidu.com/view/22900.htm) \| ▪ [KNB](http://baike.baidu.com/searchword/?word=KNB&pic=1&sug=1&enc=utf8) \| \| 加盟电台 \| ▪  ▪  ▪  ▪ \| [MRO](http://baike.baidu.com/view/981647.htm)  [BSS](http://baike.baidu.com/view/453125.htm)  [RNB](http://baike.baidu.com/view/1031667.htm)  [MBC](http://baike.baidu.com/view/335494.htm) \| ▪  ▪  ▪  ▪ \| [FBC](http://baike.baidu.com/view/1653990.htm)  [RSK](http://baike.baidu.com/searchword/?word=RSK&pic=1&sug=1&enc=utf8)  [RKC](http://baike.baidu.com/searchword/?word=RKC&pic=1&sug=1&enc=utf8)  [ROK](http://baike.baidu.com/searchword/?word=ROK&pic=1&sug=1&enc=utf8) \| ▪  ▪  ▪ \| [MBS](http://baike.baidu.com/view/254906.htm)  [RCC](http://baike.baidu.com/view/1193496.htm)  [KBC](http://baike.baidu.com/view/818218.htm) \| ▪ [ABC](http://baike.baidu.com/view/5544.htm)  ▪ [KRY](http://baike.baidu.com/view/1581361.htm)  ▪ [NBC](http://baike.baidu.com/view/495074.htm) \| ▪  ▪  ▪ \| [OBC](http://baike.baidu.com/view/1443013.htm)  [JRT](http://baike.baidu.com/searchword/?word=JRT&pic=1&sug=1&enc=utf8)  [RKK](http://baike.baidu.com/searchword/?word=RKK&pic=1&sug=1&enc=utf8) \| ▪ [KBS](http://baike.baidu.com/view/84140.htm)  ▪ [RNC](http://baike.baidu.com/view/364500.htm)  ▪ [MRT](http://baike.baidu.com/view/1336474.htm) \| \| 原加盟电台 \| ▪ \| [CRK](http://baike.baidu.com/searchword/?word=CRK&pic=1&sug=1&enc=utf8) \|  \|  \|  \|  \|  \|  \|  \|  \|   参考资料    1 [加强大众科学认知肠易激综合征](https://baike.baidu.com/reference/10530572/266ftLVUuWJg1qDmaGdi8vSYeg9ImFQsvjUX2KTEq6scwAIJTwGmbZIw3mbPh0grgnwgp1kRhALnIBi8uAWGrE0SgspBbNsgWs7rY4nZqQxUx54) ．新华网[引用日期2015-04-22]  学术论文  内容来自    [李红缨，高丽，李宁秀. IBS-QOL专用量表在肠易激综合征患者中的运用．](https://xueshu.baidu.com/usercenter/paper/show?paperid=e8ae296528f4be36f74ad73fa71e4e44&tn=SE_baiduxueshu_c1gjeupa&ie=utf-8&site=baike) 《中国循证医学杂志》， 2004  [王世勇，陈曦，高权国. 艾灸治疗肠道易激综合征(IBS)30例临床观察．](https://xueshu.baidu.com/usercenter/paper/show?paperid=8f3562abecd2d4f54d67fa2d6b880780&tn=SE_baiduxueshu_c1gjeupa&ie=utf-8&site=baike) 《CNKI;WanFang》， 2003  [樊冬梅，刘凤斌，杨晓军等. 便秘型肠易激综合征(IBS)从脾论治的病机探微．](https://xueshu.baidu.com/usercenter/paper/show?paperid=b5eca34bd35010aa17e44ad5b7197e4a&tn=SE_baiduxueshu_c1gjeupa&ie=utf-8&site=baike) 《吉林中医药》， 2006  [李敏雅. 陆维宏辨证治疗肠易激综合征(IBS)经验拾萃．](https://xueshu.baidu.com/usercenter/paper/show?paperid=698afb956aebfc4359183ee1181f329b&tn=SE_baiduxueshu_c1gjeupa&ie=utf-8&site=baike) 《浙江中医药大学学报》， 2009  [康宁宁，潘迪，谭悦，付钰. 益生菌联合情志疗法对腹泻型肠易激综合征(IBS-D)患者的疗效及生存](https://xueshu.baidu.com/usercenter/paper/show?paperid=9dc3d60b304047ee76d8df11851e63ae&tn=SE_baiduxueshu_c1gjeupa&ie=utf-8&site=baike)  《VIP》， 2016  [查看全部](https://xueshu.baidu.com/s?wd=IBS+%E8%82%A0%E6%98%93%E6%BF%80%E7%BB%BC%E5%90%88%E5%BE%81&tn=SE_baiduxueshu_c1gjeupa&ie=utf-8&sc_from=pingtai6&site=baike) |

| 猜你喜欢 | [vvs欧洲供应商凯信贸易](http://www.baidu.com/baidu.php?url=Ks00000EAMrnlPLIyWswfF5Pk8AMjjS_PrGalQaSjuJY8_SUc2m93To1RXXexAu-OjKJmq-LAHr3tR97s3ViQcfrI4H_8SjSTdceMNV9WovmDCPi5JN6Uz5uJXYbCkkKBC-kz98ovfF7jB3Ju2PZhwwA9lS1GiiTBFDvqVIg6migruRXMVYNkTnV5zDqszEZe1gu4pOVXx1RWyaaQBuM8UGYq7Fw.Db_iIoQS9tNSaPtim3Y2pMpRt85R_nYQ7xu83tN0.U1Yk0ZDqiRFH0ZfqpyF10A-V5HDzPWc0Iybq0ZKGujYzn0KWpyfqP1c0mhbqn10k0AuY5H00TA6qn0KET1Ys0AFL5H00UMfqn0K1XWY0ThIYmyTqn0K8IM0qna3snj0snj0sn0K-ThTqn0KYTh7buHYdPH0znjD0mhwGujdaPHn3nDfsfbNanDmYPbuarDnvrHcdfbDYfHbkrj7An6KbmvPb5fK9TdqGuAnquj0VuLGCXZb0u1dLTv410ZFY5Hm4nfKkTA-b5H00TyPGujYs0A7B5HKxn0KsTjYs0AdYTjYs0AwbUL0qn0KzpWYs0ZwdT1YYnHR3rH0kPWc1rH6znHcsn10v0A7W5HD0TA3qn0Ksmgwxuhk9u1Ys0AN1IjYs0ANYpyfqQHD0mgPsmvnqn0KdTA-8mvnqn0KhmLNY5H00mywhUA7M5HD0IvuzUvYq0AFY5H00XZPYIHY1nHDYn163nfKzug7Y5HDvP104rjf4n1TYnHn0Tv-b5H0smhc1PjmYrjFhuj6vuAD0ULfqn0KETMKY5H0WnaPDw-fWnansc10Wna3sc10WwDuRc10WwDuR0AVG5H00UgfqnW0vn6KVm1YznjcLP1mvn1cLP0KVmdqhThqV5H00uA78IyF-gLK_my4GuZnqn0K9uZ745R-aRsK9uZ7Y5H00pgPWUjYs0Z7VIjYs0A7bgLPEIgFWuHYkranW0APzm1YzPjcvP0&us=newvui&xst=m1YKmWdaPHn3nDfsfbNanDmYPbuarDnvrHcdfbDYfHbkrj7An6715HRzPWb1PjcvrHf1rHD4rjnYg1DzPWFxn07L5y-BTs7k5y-BTs7d5HnknHf1rj6k0gfqnHmLnjb3Pjb1Ps7VTHYk0W0aiRFH0yPC5yuWgLKW0Hnkn1D3PHRzPj6&ai=0_429217685_1_0&word=&ck=0.0.0.0.0.0.0.0&shh=baike.baidu.com)  [ibs德国直采，原厂报价单报价，折扣优惠，可提供报关单，欧洲 工业品上游供应商，经营65家代理品牌， 2486家真实成交20000 … www.kaixinbusiness.com](http://www.baidu.com/baidu.php?url=Ks00000EAMrnlPLIyWswfF5Pk8AMjjS_PrGalQaSjuJY8_SUc2m93To1RXXexAu-OjKJmq-LAHr3tR97s3ViQcfrI4H_8SjSTdceMNV9WovmDCPi5JN6Uz5uJXYbCkkKBC-kz98ovfF7jB3Ju2PZhwwA9lS1GiiTBFDvqVIg6migruRXMVYNkTnV5zDqszEZe1gu4pOVXx1RWyaaQBuM8UGYq7Fw.Db_iIoQS9tNSaPtim3Y2pMpRt85R_nYQ7xu83tN0.U1Yk0ZDqiRFH0ZfqpyF10A-V5HDzPWc0Iybq0ZKGujYzn0KWpyfqP1c0mhbqn10k0AuY5H00TA6qn0KET1Ys0AFL5H00UMfqn0K1XWY0ThIYmyTqn0K8IM0qna3snj0snj0sn0K-ThTqn0KYTh7buHYdPH0znjD0mhwGujdaPHn3nDfsfbNanDmYPbuarDnvrHcdfbDYfHbkrj7An6KbmvPb5fK9TdqGuAnquj0VuLGCXZb0u1dLTv410ZFY5Hm4nfKkTA-b5H00TyPGujYs0A7B5HKxn0KsTjYs0AdYTjYs0AwbUL0qn0KzpWYs0ZwdT1YYnHR3rH0kPWc1rH6znHcsn10v0A7W5HD0TA3qn0Ksmgwxuhk9u1Ys0AN1IjYs0ANYpyfqQHD0mgPsmvnqn0KdTA-8mvnqn0KhmLNY5H00mywhUA7M5HD0IvuzUvYq0AFY5H00XZPYIHY1nHDYn163nfKzug7Y5HDvP104rjf4n1TYnHn0Tv-b5H0smhc1PjmYrjFhuj6vuAD0ULfqn0KETMKY5H0WnaPDw-fWnansc10Wna3sc10WwDuRc10WwDuR0AVG5H00UgfqnW0vn6KVm1YznjcLP1mvn1cLP0KVmdqhThqV5H00uA78IyF-gLK_my4GuZnqn0K9uZ745R-aRsK9uZ7Y5H00pgPWUjYs0Z7VIjYs0A7bgLPEIgFWuHYkranW0APzm1YzPjcvP0&us=newvui&xst=m1YKmWdaPHn3nDfsfbNanDmYPbuarDnvrHcdfbDYfHbkrj7An6715HRzPWb1PjcvrHf1rHD4rjnYg1DzPWFxn07L5y-BTs7k5y-BTs7d5HnknHf1rj6k0gfqnHmLnjb3Pjb1Ps7VTHYk0W0aiRFH0yPC5yuWgLKW0Hnkn1D3PHRzPj6&ai=0_429217685_1_0&word=&ck=0.0.0.0.0.0.0.0&shh=baike.baidu.com) |  | [德国ibs的产品，原装进口，欢迎询价 优势供应ibs的产品，报价快，货期稳定，品质保证，质优价 提供报关单和原产地证明，期待您的来电!](http://www.baidu.com/baidu.php?url=Ks00000EAMrnlPLIyVQ77okPpeChwzuhhaFGcwbivRbRi04bVLVLrdEXY6Dc9CpNAqWiPAjkjumaZdwlLDLncUM59Cw3TbAN_ze4QGQxlO0UWP6D6a9nkT8CUSNHJSscUec9LeJZelXx2ZeGUY_hfGTMPsaMStw8hdPKoGwCOUK1sABiF6Yp8RnnLarBGHPDPMpMDqNiDcn97yRhOp5yzdUmHCBR.7R_jv5sfdF1ulampbf2pMpRt85R_nYQAeI34Pl6.U1Yz0ZDqiRFH0ZfqpyF10A-V5HDzPWc0Iybq0ZKGujYzn0KWpyfqP1c0mhbqn10k0AuY5H00TA6qn0KET1Ys0AFL5H00UMfqn0K1XWY0ThIYmyTqn0K8IM0qna3snj0snj0sn0K-ThTqn0KYTh7buHYvPj0znjn0mhwGujdaPHn3nDfsfbNanDmYPbuarDnvrHcdfbDYfHbkrj7An6KbmvPb5fK9TdqGuAnquj0VuLGCXZb0u1dLTv410ZFY5Hm4nfKkTA-b5H00TyPGujYs0A7B5HKxn0KsTjYs0AdYTjYs0AwbUL0qn0KzpWYs0ZwdT1Y4njfdnj0LPWmdrjDdnjD4njnL0A7W5HD0TA3qn0Ksmgwxuhk9u1Ys0AN1IjYs0ANYpyfqQHD0mgPsmvnqn0KdTA-8mvnqn0KhmLNY5H00mywhUA7M5HD0IvuzUvYq0AFY5H00XZPYIHY1njn4Pjfdn0Kzug7Y5HDvP104rjf4n1TYnHn0Tv-b5H0smhc1PjmYrjFhuj6vuAD0ULfqn6KETMKY5HcWnandr1DLr1nsc1Dsnj0WnHmzPjmLPHmsPBnsc108nan1c1Dsnj0WnanV0AVG5H00UgfqnW0vn6KVm1YznWRknjbdPj6dP6KVmdqhThqV5H00uA78IyF-gLK_my4GuZnqn0K9uZ745R-aRsK9uZ7Y5H00pgPWUjYs0Z7VIjYs0A7bgLPEIgFWuHYkranW0APzm1Y1Pj0k&us=newvui&xst=m1YKmWdaPHn3nDfsfbNanDmYPbuarDnvrHcdfbDYfHbkrj7An6715HRzPWb1PjcvrHf1rHD4rjnYg1DzPWFxnf7L5y-BTs7k5y-BTs7d5Hnsn1bYPjRs0gfqnHmLnjb3Pjb1Ps7VTHYk0W0aiRFH0yPC5yuWgLKW0Hcvn1TznHf3rjn&ai=0_429147520_1_0&word=&ck=0.0.0.0.0.0.0.0&shh=baike.baidu.com)  [www.qiucheng-tech.com](http://www.baidu.com/baidu.php?url=Ks00000EAMrnlPLIyVQ77okPpeChwzuhhaFGcwbivRbRi04bVLVLrdEXY6Dc9CpNAqWiPAjkjumaZdwlLDLncUM59Cw3TbAN_ze4QGQxlO0UWP6D6a9nkT8CUSNHJSscUec9LeJZelXx2ZeGUY_hfGTMPsaMStw8hdPKoGwCOUK1sABiF6Yp8RnnLarBGHPDPMpMDqNiDcn97yRhOp5yzdUmHCBR.7R_jv5sfdF1ulampbf2pMpRt85R_nYQAeI34Pl6.U1Yz0ZDqiRFH0ZfqpyF10A-V5HDzPWc0Iybq0ZKGujYzn0KWpyfqP1c0mhbqn10k0AuY5H00TA6qn0KET1Ys0AFL5H00UMfqn0K1XWY0ThIYmyTqn0K8IM0qna3snj0snj0sn0K-ThTqn0KYTh7buHYvPj0znjn0mhwGujdaPHn3nDfsfbNanDmYPbuarDnvrHcdfbDYfHbkrj7An6KbmvPb5fK9TdqGuAnquj0VuLGCXZb0u1dLTv410ZFY5Hm4nfKkTA-b5H00TyPGujYs0A7B5HKxn0KsTjYs0AdYTjYs0AwbUL0qn0KzpWYs0ZwdT1Y4njfdnj0LPWmdrjDdnjD4njnL0A7W5HD0TA3qn0Ksmgwxuhk9u1Ys0AN1IjYs0ANYpyfqQHD0mgPsmvnqn0KdTA-8mvnqn0KhmLNY5H00mywhUA7M5HD0IvuzUvYq0AFY5H00XZPYIHY1njn4Pjfdn0Kzug7Y5HDvP104rjf4n1TYnHn0Tv-b5H0smhc1PjmYrjFhuj6vuAD0ULfqn6KETMKY5HcWnandr1DLr1nsc1Dsnj0WnHmzPjmLPHmsPBnsc108nan1c1Dsnj0WnanV0AVG5H00UgfqnW0vn6KVm1YznWRknjbdPj6dP6KVmdqhThqV5H00uA78IyF-gLK_my4GuZnqn0K9uZ745R-aRsK9uZ7Y5H00pgPWUjYs0Z7VIjYs0A7bgLPEIgFWuHYkranW0APzm1Y1Pj0k&us=newvui&xst=m1YKmWdaPHn3nDfsfbNanDmYPbuarDnvrHcdfbDYfHbkrj7An6715HRzPWb1PjcvrHf1rHD4rjnYg1DzPWFxnf7L5y-BTs7k5y-BTs7d5Hnsn1bYPjRs0gfqnHmLnjb3Pjb1Ps7VTHYk0W0aiRFH0yPC5yuWgLKW0Hcvn1TznHf3rjn&ai=0_429147520_1_0&word=&ck=0.0.0.0.0.0.0.0&shh=baike.baidu.com) |
| --- | --- | --- | --- |
|  | | | |
| 岔 搜索发现 |  | | |

<https://baike.baidu.com/item/IBS/10530572?fromModule=lemma_search-box>

3/4

2022/12/14 10:29

IBS (肠易激综合征) _百度百科

| [肠易激综合征吃什么药](https://www.baidu.com/s?word=%E8%82%A0%E6%98%93%E6%BF%80%E7%BB%BC%E5%90%88%E5%BE%81%E5%90%83%E4%BB%80%E4%B9%88%E8%8D%AF&tn=SE_baikepcxf02_fcetbk02&pos=baike_pc_turbo_1767&ori_sid=00bb34639a7e9090)  [robey+warshaw](https://www.baidu.com/s?word=robey%2Bwarshaw&tn=SE_baikepcxf02_fcetbk02&pos=baike_pc_turbo_1767&ori_sid=00bb34639a7e9090) |
| --- |

肠易激综合征的症状 珠海ibs

[编辑](javascript:;)

全日制英语

[ibs是什么意思](https://www.baidu.com/s?word=ibs%E6%98%AF%E4%BB%80%E4%B9%88%E6%84%8F%E6%80%9D&tn=SE_baikepcxf02_fcetbk02&pos=baike_pc_turbo_1767&ori_sid=00bb34639a7e9090)

[收藏](javascript:;)

英语学校

[IBS主要症状](https://www.baidu.com/s?word=IBS%E4%B8%BB%E8%A6%81%E7%97%87%E7%8A%B6&tn=SE_baikepcxf02_fcetbk02&pos=baike_pc_turbo_1767&ori_sid=00bb34639a7e9090)

ibs学校 ibs酒店

[回 讨论](https://baike.baidu.com/planet/talk?lemmaId=10530572)

小 播报

[赞](javascript:;)

新手上路

我有疑问

投诉建议

[成长任务](https://baike.baidu.com/usercenter/tasks#guide) [编辑规则](https://baike.baidu.com/help#main06)

[编辑入门](https://baike.baidu.com/help#main01) [内容质疑](javascript:void(0);)

[本人编辑](https://baike.baidu.com/item/%E7%99%BE%E5%BA%A6%E7%99%BE%E7%A7%91%EF%BC%9A%E6%9C%AC%E4%BA%BA%E8%AF%8D%E6%9D%A1%E7%BC%96%E8%BE%91%E6%9C%8D%E5%8A%A1/22442459?bk_fr=pcFooter) [官方贴吧](http://tieba.baidu.com/f?ie=utf-8&fr=bks0000&kw=%E7%99%BE%E5%BA%A6%E7%99%BE%E7%A7%91)

[在线客服](http://zhiqiu.baidu.com/baike/passport/html/baikechat.html)

[意见反馈](javascript:void(0);)

[举报不良信息](http://help.baidu.com/newadd?word=IBS&&submit_link=https%3A%2F%2Fbaike.baidu.com%2Fitem%2FIBS%2F10530572%3FfromModule%3Dlemma_search-box&prod_id=10&category=1) [投诉侵权信息](http://help.baidu.com/newadd?word=IBS&&submit_link=https%3A%2F%2Fbaike.baidu.com%2Fitem%2FIBS%2F10530572%3FfromModule%3Dlemma_search-box&prod_id=10&category=6)

[未通过词条申诉](http://help.baidu.com/newadd?word=IBS&&submit_link=https%3A%2F%2Fbaike.baidu.com%2Fitem%2FIBS%2F10530572%3FfromModule%3Dlemma_search-box&prod_id=10&category=2)

[封禁查询与解封](http://help.baidu.com/newadd?word=IBS&&submit_link=https%3A%2F%2Fbaike.baidu.com%2Fitem%2FIBS%2F10530572%3FfromModule%3Dlemma_search-box&prod_id=10&category=5)

©2022 Baidu [使用百度前必读](http://www.baidu.com/duty/) | [百科协议](http://help.baidu.com/question?prod_en=baike&class=89&id=1637) | [隐私政策](http://help.baidu.com/question?prod_id=10&class=690&id=1001779) | [百度百科合作平台](https://baike.baidu.com/operation/cooperation) | 京ICP证030173号

[京公网安备11000002000001号](http://www.beian.gov.cn/portal/registerSystemInfo?recordcode=11000002000001)

<https://baike.baidu.com/item/IBS/10530572?fromModule=lemma_search-box>

4/4
