# Supplementary material for: Assessment of medical information on irritable bowel syndrome information in Wikipedia and Baidu Encyclopedia: comparative study
Source: PeerJ. 2024 May 24;12:e17264. doi: 10.7717/peerj.17264 (PMC11129691; doi:10.7717/peerj.17264)
Supplement: Data S1 [file peerj-12-17264-s001.zip › σÄƒσoïμò░μì«/Baidu/Baidu-English/7-ΦéáΦ║üτùç_τÖ╛σ║aτÖ╛τoæ.docx]

| 2022/12/14 10:42  [网页](https://www.baidu.com/) | [新闻](http://news.baidu.com/) | 肠躁症_百度百科  [贴吧](https://tieba.baidu.com/) [知道](https://zhidao.baidu.com/) [网盘](https://pan.baidu.com/?from=1027327l) | [图片](http://image.baidu.com/) | [视频](http://v.baidu.com/) | [地图](http://map.baidu.com/) | [文库](https://wenku.baidu.com/) | 百科 | [百度首页](http://www.baidu.com/) [登录](javascript:;) |
| --- | --- | --- | --- | --- | --- | --- | --- | --- |

| [时岔謂](https://baike.baidu.com/) | \| 肠躁症 \| 进入词条 \| \| --- \| --- \| | \| 全站搜索 \| \| --- \| | [帮助](https://baike.baidu.com/help) |
| --- | --- | --- | --- | --- | --- | --- |
| 近期有不法分子冒充百度百科官方人员，以删除词条为由威胁并敲诈相关企业。在此严正声明：百度百科是免费编辑平台，绝不存在收费代编服务，请勿上当受骗！ [详情>>](https://baike.baidu.com/common/declaration) | | | |
| [首页](https://baike.baidu.com/) 秒懂百科 特色百科 用户 知识专题 权威合作 [口下载百科APP](https://baike.baidu.com/wapui/subpage/baikeappdownload?sfrom=pc_lemmapage_navigation) [2 个](https://baike.baidu.com/usercenter) | | | |

| 肠躁症 | \| [小播报](javascript:;) \| \| --- \| | \| [编辑](javascript:;) \| \| --- \| | \| [上传视频](javascript:;) \| \| --- \| | . 收藏 [山 1627](javascript:void(0);) 1093 | [肠躁症的概述图(1张)](https://baike.baidu.com/pic/%E8%82%A0%E8%BA%81%E7%97%87/86605/1/e1fe9925bc315c60daebf9fe8bb1cb13495477a1?fr=lemma&fromModule=lemma_top-image&ct=single) |
| --- | --- | --- | --- | --- | --- | --- | --- | --- |
| Irritable bowel disorder is the most common digestive system disorder, at least 10~15% of adults occur, most cases are caused by excessive stress, greatly affected by emotions. | | | | |  |
| \| 别 名 \| 肠躁症 \| 中医病名  外文名 \| 结肠痉挛  Irritable Bowel Syndrome \| \| --- \| --- \| --- \| --- \| | | | | |  |
| 英文名称： Irritable Bowel Syndrome  中文名称： 肠躁症(躁性大肠症候群)  简称缩写： IBS  Explanation of terms: Also known as colonic spasm, it is the most common digestive system disorder, occurring at least 10~15% of adults, and about 90% of cases are related to psychological stress. The main symptoms are abdominal pain, gastrointestinal discomfort, constipation, or diarrhea. Within the past 12 months, continuous or intermittent, with the above symptoms for more than 12 weeks.  [词条图册 更多图册 >](https://baike.baidu.com/pic/%E8%82%A0%E8%BA%81%E7%97%87/86605?fr=lemma) | | | | |  |
|  |  |  |  |  | \| 词条统计  浏览次数： 90686次  编辑次数： 8次[历史版本](https://baike.baidu.com/historylist/%E8%82%A0%E8%BA%81%E7%97%87/86605)  最近更新： [sunnyahuman](https://baike.baidu.com/usercenter/userpage?uk=jT098525sw_zxnRbYmbzKg&from=lemma) ( 2016-01-25  突出贡献榜  [tjh19931014](https://baike.baidu.com/usercenter/userpage?uk=bVuWOcBh-AnT5qKsLfFDSw&from=lemma) \| \| --- \| |
|  |  |  |  |  | [出版社自费出](https://cpro.baidu.com/cpro/ui/uijs.php?en=mywWUA71T1YsFh7sT7qGujYsFhPC5H0huAbqrauGTdq9TZ0qnauJp1d-uj0suyPBrjFWnAu-Pj63Fh_qFRc1FRmvFRcsFRRvFRn4FRRLFRfLFRfYFRcLFRfkFRc1FRmvFRPKFRR4FhkdpvbqniuVmLKV5HDknjR1PBuk5HnLnjbYnHfsgvPsTBuzmWYsFMF15HDhTvN_UANzgv-b5HDhTv-b5H63uHfsuWFWmW9-m10suyfhTLwGujY3FMfqIZKWUA-WpvNbndqCmzuYujYkrHbLPWn1FMwV5Hcvrj6sn1R3niuYUgnqnHmLnjb3PHmkPBuYIHddnHb4P1m1nzud5y9YIZK1FHPKFHFAFHFAmh7GpvR-nbNBmy-bIiRzwyPEUiuv5HchpHYkujmkmW0dm6&besl=6&c=news&cf=1&cvrq=7189029&eid_list=200214_202016_204854_205526_209357&expid=200214_202016_202257_202564_204854_205526_205809_206503_209394&fr=20&fv=0&haacp=618&img_typ=0&itm=0&lu_idc=gzhxy&lukid=1&lus=88e40f2cb8ec00ed&lust=63993790&luwtr=7476068094432150857&mscf=0&n=10&nttp=1&p=baidu&pbs=220093&sce=7&sr=72&ssp2=1&tpl=baiduCustITagLinkUnitRankCol&tsf=dtp:1&tu_type=0&u=%2Fitem%2F%25E8%2582%25A0%25E8%25BA%2581%25E7%2597%2587%2F86605%3FfromModule%3Dsearch%2Dresult%5Flemma&uicf=lurecv&urlid=0&eot=1)  **12** 自己创建个  **13** 虚拟货币平  **14** vr消防演练  **15** 亚马逊女图书  **16** 购买域子名  **17** 价格便宜的    **20**  **21** 怎么创口建小  **22** 供应链管理  **1**  **2**  **3**  **4**  **5**  **6**  **7**  **8**  **9**  **10**  **11**  [csgo网站开](https://cpro.baidu.com/cpro/ui/uijs.php?en=mywWUA71T1YsFh7sT7qGujYsFhPC5H0huAbqrauGTdq9TZ0qnauJp1d-uj0suyPBrjFWnAu-Pj63Fh_qmLPMUzNjwaNAraNDPiNawiNawBNKfiNjwBN7Pau_IyVG5HcsFhdWTAYqnHcknjbkFMDqn1TsrHfkPjKxmLKzFMFB5H0hTMnqniu1uyk_ugFxpyfqniu1pyfqrj9-PjKhnhPBrANWnjK-uau1IA-b5H6hIjdYTAP_pyPouyf1gv9WFMwb5HD4rHTvn1nhIAYqnWm3rj01PH6kFMwVT1YkPWTsrH6dPWDvFMwd5gRkrHbLPWn1FMRqpZwYTZn-nYD-nbm-nbuBmy-ouiRzwyF9pywdFHF7mvqVFMmqnBuG5HwBP103nHnd&besl=6&c=news&cf=1&cvrq=4489244&eid_list=200214_202016_204854_205526_209357&expid=200214_202016_202257_202564_204854_205526_205809_206503_209394&fr=20&fv=0&haacp=624&img_typ=0&itm=0&lu_idc=gzhxy&lukid=20&lus=88e40f2cb8ec00ed&lust=63993790&luwtr=2267704484931445128&mscf=0&n=10&nttp=1&p=baidu&pbs=220093&sce=7&sr=72&ssp2=1&tpl=baiduCustITagLinkUnitRankCol&tsf=dtp:1&tu_type=0&u=%2Fitem%2F%25E8%2582%25A0%25E8%25BA%2581%25E7%2597%2587%2F86605%3FfromModule%3Dsearch%2Dresult%5Flemma&uicf=lurecv&urlid=0&eot=1)  [游戏盒子](https://cpro.baidu.com/cpro/ui/uijs.php?en=mywWUA71T1YsFh7sT7qGujYsFhPC5H0huAbqrauGTdq9TZ0qnauJp1d-uj0suyPBrjFWnAu-Pj63Fh_qFRf1FRP7FRPAFRcLFRFKFRfsFRfLFRf1FhkdpvbqnBuVmLKV5HTzrH0dFMDqn1TsrHfkPjKxmLKzFMFB5H0hTMnqniu1uyk_ugFxpyfqniu1pyfqrj9-PjKhnhPBrANWnjK-uau1IA-b5H6hIjdYTAP_pyPouyf1gv9WFMwb5HD4rHTvn1nhIAYqnWm3rj01PH6kFMwVT1YkPWTsrH6dPWDvFMwd5gRkrHbLPWn1FMRqpZwYTZn-nYD-nbm-nbuBmy-ouiRzwyF9pywdFHF7mvqVFMmqnBuG5HnLn1T4PHuB&besl=6&c=news&cf=1&cvrq=3228426&eid_list=200214_202016_204854_205526_209357&expid=200214_202016_202257_202564_204854_205526_205809_206503_209394&fr=20&fv=0&haacp=271&img_typ=0&itm=0&lu_idc=gzhxy&lukid=2&lus=88e40f2cb8ec00ed&lust=63993790&luwtr=13245032438307475642&mscf=0&n=10&nttp=1&p=baidu&pbs=220093&sce=7&sr=72&ssp2=1&tpl=baiduCustITagLinkUnitRankCol&tsf=dtp:1&tu_type=0&u=%2Fitem%2F%25E8%2582%25A0%25E8%25BA%2581%25E7%2597%2587%2F86605%3FfromModule%3Dsearch%2Dresult%5Flemma&uicf=lurecv&urlid=0&eot=1)  [网络安全培训](https://cpro.baidu.com/cpro/ui/uijs.php?en=mywWUA71T1YsFh7sT7qGujYsFhPC5H0huAbqrauGTdq9TZ0qnauJp1d-uj0suyPBrjFWnAu-Pj63Fh_qFRPDFRm3FRnzFRRLFRcsFRczFRn3FR7aFRndFRRsFRfkFRcdFhkdpvbqnzuVmLKV5HDznjmLniuk5HnLnjbYnHfsgvPsTBuzmWYsFMF15HDhTvN_UANzgv-b5HDhTv-b5H63uHfsuWFWmW9-m10suyfhTLwGujY3FMfqIZKWUA-WpvNbndqCmzuYujYkrHbLPWn1FMwV5Hcvrj6sn1R3niuYUgnqnHmLnjb3PHmkPBuYIHddnHb4P1m1nzud5y9YIZK1FHPKFHFAFHFAmh7GpvR-nbNBmy-bIiRzwyPEUiuv5HchpHY4m1uWPHPbr0&besl=6&c=news&cf=1&cvrq=3292150&eid_list=200214_202016_204854_205526_209357&expid=200214_202016_202257_202564_204854_205526_205809_206503_209394&fr=20&fv=0&haacp=1162&img_typ=0&itm=0&lu_idc=gzhxy&lukid=3&lus=88e40f2cb8ec00ed&lust=63993790&luwtr=2249689388344567100&mscf=0&n=10&nttp=1&p=baidu&pbs=220093&sce=7&sr=72&ssp2=1&tpl=baiduCustITagLinkUnitRankCol&tsf=dtp:1&tu_type=0&u=%2Fitem%2F%25E8%2582%25A0%25E8%25BA%2581%25E7%2597%2587%2F86605%3FfromModule%3Dsearch%2Dresult%5Flemma&uicf=lurecv&urlid=0&eot=1)  [国际期货](https://cpro.baidu.com/cpro/ui/uijs.php?en=mywWUA71T1YsFh7sT7qGujYsFhPC5H0huAbqrauGTdq9TZ0qnauJp1d-uj0suyPBrjFWnAu-Pj63Fh_qFRc4FRuKFRFjFRPKFRnvFRwKFRFaFRmdFhkdpvbqPauVmLKV5HDsrjb1Pzuk5HnLnjbYnHfsgvPsTBuzmWYsFMF15HDhTvN_UANzgv-b5HDhTv-b5H63uHfsuWFWmW9-m10suyfhTLwGujY3FMfqIZKWUA-WpvNbndqCmzuYujYkrHbLPWn1FMwV5Hcvrj6sn1R3niuYUgnqnHmLnjb3PHmkPBuYIHddnHb4P1m1nzud5y9YIZK1FHPKFHFAFHFAmh7GpvR-nbNBmy-bIiRzwyPEUiuv5HchpHdbrH99uHwBuf&besl=6&c=news&cf=1&cvrq=1621391&eid_list=200214_202016_204854_205526_209357&expid=200214_202016_202257_202564_204854_205526_205809_206503_209394&fr=20&fv=0&haacp=2073&img_typ=0&itm=0&lu_idc=gzhxy&lukid=4&lus=88e40f2cb8ec00ed&lust=63993790&luwtr=14608826967881669318&mscf=0&n=10&nttp=1&p=baidu&pbs=220093&sce=7&sr=72&ssp2=1&tpl=baiduCustITagLinkUnitRankCol&tsf=dtp:1&tu_type=0&u=%2Fitem%2F%25E8%2582%25A0%25E8%25BA%2581%25E7%2597%2587%2F86605%3FfromModule%3Dsearch%2Dresult%5Flemma&uicf=lurecv&urlid=0&eot=1)  [电商平台怎么](https://cpro.baidu.com/cpro/ui/uijs.php?en=mywWUA71T1YsFh7sT7qGujYsFhPC5H0huAbqrauGTdq9TZ0qnauJp1d-uj0suyPBrjFWnAu-Pj63Fh_qFRcdFRRLFRn4FRPjFRnvFRFDFRPjFRD3FRfYFRmdFRn1FRcYFRfLFRmvFhkdpvbqPiuVmLKV5H6sP1T3FMDqn1TsrHfkPjKxmLKzFMFB5H0hTMnqniu1uyk_ugFxpyfqniu1pyfqrj9-PjKhnhPBrANWnjK-uau1IA-b5H6hIjdYTAP_pyPouyf1gv9WFMwb5HD4rHTvn1nhIAYqnWm3rj01PH6kFMwVT1YkPWTsrH6dPWDvFMwd5gRkrHbLPWn1FMRqpZwYTZn-nYD-nbm-nbuBmy-ouiRzwyF9pywdFHF7mvqVFMmqnBuG5HI-PhRsrA7W&besl=6&c=news&cf=1&cvrq=3247861&eid_list=200214_202016_204854_205526_209357&expid=200214_202016_202257_202564_204854_205526_205809_206503_209394&fr=20&fv=0&haacp=3601&img_typ=0&itm=0&lu_idc=gzhxy&lukid=5&lus=88e40f2cb8ec00ed&lust=63993790&luwtr=7777852140969087504&mscf=0&n=10&nttp=1&p=baidu&pbs=220093&sce=7&sr=72&ssp2=1&tpl=baiduCustITagLinkUnitRankCol&tsf=dtp:1&tu_type=0&u=%2Fitem%2F%25E8%2582%25A0%25E8%25BA%2581%25E7%2597%2587%2F86605%3FfromModule%3Dsearch%2Dresult%5Flemma&uicf=lurecv&urlid=0&eot=1)  [战队logo设计](https://cpro.baidu.com/cpro/ui/uijs.php?en=mywWUA71T1YsFh7sT7qGujYsFhPC5H0huAbqrauGTdq9TZ0qnauJp1d-uj0suyPBrjFWnAu-Pj63Fh_qFRfdFRFDFRcvFRf1UAqMUzNjriN7raNafzNjPBu_IyVG5HmhUyPsUHY4PHc1Pauk5HnLnjbYnHfsgvPsTBuzmWYsFMF15HDhTvN_UANzgv-b5HDhTv-b5H63uHfsuWFWmW9-m10suyfhTLwGujY3FMfqIZKWUA-WpvNbndqCmzuYujYkrHbLPWn1FMwV5Hcvrj6sn1R3niuYUgnqnHmLnjb3PHmkPBuYIHddnHb4P1m1nzud5y9YIZK1FHPKFHFAFHFAmh7GpvR-nbNBmy-bIiRzwyPEUiuv5HchpHd9uyPBmHP-u0&besl=6&c=news&cf=1&cvrq=1756705&eid_list=200214_202016_204854_205526_209357&expid=200214_202016_202257_202564_204854_205526_205809_206503_209394&fr=20&fv=0&haacp=707&img_typ=0&itm=0&lu_idc=gzhxy&lukid=6&lus=88e40f2cb8ec00ed&lust=63993790&luwtr=1863292750894598650&mscf=0&n=10&nttp=1&p=baidu&pbs=220093&sce=7&sr=72&ssp2=1&tpl=baiduCustITagLinkUnitRankCol&tsf=dtp:1&tu_type=0&u=%2Fitem%2F%25E8%2582%25A0%25E8%25BA%2581%25E7%2597%2587%2F86605%3FfromModule%3Dsearch%2Dresult%5Flemma&uicf=lurecv&urlid=0&eot=1)  [无人机反制](https://cpro.baidu.com/cpro/ui/uijs.php?en=mywWUA71T1YsFh7sT7qGujYsFhPC5H0huAbqrauGTdq9TZ0qnauJp1d-uj0suyPBrjFWnAu-Pj63Fh_qFRP7FRw7FRn3FRPaFRFaFRuKFRcLFRcYFRfvFRnvFhkdpvbqPzuVmLKV5H6sP1bvFMDqn1TsrHfkPjKxmLKzFMFB5H0hTMnqniu1uyk_ugFxpyfqniu1pyfqrj9-PjKhnhPBrANWnjK-uau1IA-b5H6hIjdYTAP_pyPouyf1gv9WFMwb5HD4rHTvn1nhIAYqnWm3rj01PH6kFMwVT1YkPWTsrH6dPWDvFMwd5gRkrHbLPWn1FMRqpZwYTZn-nYD-nbm-nbuBmy-ouiRzwyF9pywdFHF7mvqVFMmqnBuG5HmvnhwbujuW&besl=6&c=news&cf=1&cvrq=1415934&eid_list=200214_202016_204854_205526_209357&expid=200214_202016_202257_202564_204854_205526_205809_206503_209394&fr=20&fv=0&haacp=904&img_typ=0&itm=0&lu_idc=gzhxy&lukid=7&lus=88e40f2cb8ec00ed&lust=63993790&luwtr=685006414410405008&mscf=0&n=10&nttp=1&p=baidu&pbs=220093&sce=7&sr=72&ssp2=1&tpl=baiduCustITagLinkUnitRankCol&tsf=dtp:1&tu_type=0&u=%2Fitem%2F%25E8%2582%25A0%25E8%25BA%2581%25E7%2597%2587%2F86605%3FfromModule%3Dsearch%2Dresult%5Flemma&uicf=lurecv&urlid=0&eot=1)  [csgo电脑配置](https://cpro.baidu.com/cpro/ui/uijs.php?en=mywWUA71T1YsFh7sT7qGujYsFhPC5H0huAbqrauGTdq9TZ0qnauJp1d-uj0suyPBrjFWnAu-Pj63Fh_qmLPMUzNaPiN7PzNjPaNDPaNjPiN7PaNDPBNjnzu_IyVG5H6hUyPsUHYLrjcsniuk5HnLnjbYnHfsgvPsTBuzmWYsFMF15HDhTvN_UANzgv-b5HDhTv-b5H63uHfsuWFWmW9-m10suyfhTLwGujY3FMfqIZKWUA-WpvNbndqCmzuYujYkrHbLPWn1FMwV5Hcvrj6sn1R3niuYUgnqnHmLnjb3PHmkPBuYIHddnHb4P1m1nzud5y9YIZK1FHPKFHFAFHFAmh7GpvR-nbNBmy-bIiRzwyPEUiuv5HchpHdWmhubryuBu0&besl=6&c=news&cf=1&cvrq=3472465&eid_list=200214_202016_204854_205526_209357&expid=200214_202016_202257_202564_204854_205526_205809_206503_209394&fr=20&fv=0&haacp=611&img_typ=0&itm=0&lu_idc=gzhxy&lukid=8&lus=88e40f2cb8ec00ed&lust=63993790&luwtr=2357911270216075011&mscf=0&n=10&nttp=1&p=baidu&pbs=220093&sce=7&sr=72&ssp2=1&tpl=baiduCustITagLinkUnitRankCol&tsf=dtp:1&tu_type=0&u=%2Fitem%2F%25E8%2582%25A0%25E8%25BA%2581%25E7%2597%2587%2F86605%3FfromModule%3Dsearch%2Dresult%5Flemma&uicf=lurecv&urlid=0&eot=1)  [哈佛大学申请](https://cpro.baidu.com/cpro/ui/uijs.php?en=mywWUA71T1YsFh7sT7qGujYsFhPC5H0huAbqrauGTdq9TZ0qnauJp1d-uj0suyPBrjFWnAu-Pj63Fh_qFRc4FRu7FRcLFRmsFRcYFRm1FRfkFRDLFRn4FRNKFRnLFRNaFRPjFRmdFRFjFRu7FhkdpvbqriuVmLKV5HbknjDvFMDqn1TsrHfkPjKxmLKzFMFB5H0hTMnqniu1uyk_ugFxpyfqniu1pyfqrj9-PjKhnhPBrANWnjK-uau1IA-b5H6hIjdYTAP_pyPouyf1gv9WFMwb5HD4rHTvn1nhIAYqnWm3rj01PH6kFMwVT1YkPWTsrH6dPWDvFMwd5gRkrHbLPWn1FMRqpZwYTZn-nYD-nbm-nbuBmy-ouiRzwyF9pywdFHF7mvqVFMmqnBuG5HwhPhmkPvcv&besl=6&c=news&cf=1&cvrq=2024363&eid_list=200214_202016_204854_205526_209357&expid=200214_202016_202257_202564_204854_205526_205809_206503_209394&fr=20&fv=0&haacp=870&img_typ=0&itm=0&lu_idc=gzhxy&lukid=9&lus=88e40f2cb8ec00ed&lust=63993790&luwtr=7841139350953723636&mscf=0&n=10&nttp=1&p=baidu&pbs=220093&sce=7&sr=72&ssp2=1&tpl=baiduCustITagLinkUnitRankCol&tsf=dtp:1&tu_type=0&u=%2Fitem%2F%25E8%2582%25A0%25E8%25BA%2581%25E7%2597%2587%2F86605%3FfromModule%3Dsearch%2Dresult%5Flemma&uicf=lurecv&urlid=0&eot=1)  [图书批发网](https://cpro.baidu.com/cpro/ui/uijs.php?en=mywWUA71T1YsFh7sT7qGujYsFhPC5H0huAbqrauGTdq9TZ0qnauJp1d-uj0suyPBrjFWnAu-Pj63Fh_qFRPDFRFjFRPKFRR4FRndFRuKFRcLFRDzFRPDFRm3FhkdpvbqnH0hUyPsUHYLrHfzPiuk5HnLnjbYnHfsgvPsTBuzmWYsFMF15HDhTvN_UANzgv-b5HDhTv-b5H63uHfsuWFWmW9-m10suyfhTLwGujY3FMfqIZKWUA-WpvNbndqCmzuYujYkrHbLPWn1FMwV5Hcvrj6sn1R3niuYUgnqnHmLnjb3PHmkPBuYIHddnHb4P1m1nzud5y9YIZK1FHPKFHFAFHFAmh7GpvR-nbNBmy-bIiRzwyPEUiuv5HchpHd9mWN-rAPWuf&besl=6&c=news&cf=1&cvrq=4468518&eid_list=200214_202016_204854_205526_209357&expid=200214_202016_202257_202564_204854_205526_205809_206503_209394&fr=20&fv=0&haacp=317&img_typ=0&itm=0&lu_idc=gzhxy&lukid=10&lus=88e40f2cb8ec00ed&lust=63993790&luwtr=639862747281857404&mscf=0&n=10&nttp=1&p=baidu&pbs=220093&sce=7&sr=72&ssp2=1&tpl=baiduCustITagLinkUnitRankCol&tsf=dtp:1&tu_type=0&u=%2Fitem%2F%25E8%2582%25A0%25E8%25BA%2581%25E7%2597%2587%2F86605%3FfromModule%3Dsearch%2Dresult%5Flemma&uicf=lurecv&urlid=0&eot=1)  [网络工程师](https://cpro.baidu.com/cpro/ui/uijs.php?en=mywWUA71T1YsFh7sT7qGujYsFhPC5H0huAbqrauGTdq9TZ0qnauJp1d-uj0suyPBrjFWnAu-Pj63Fh_qFRPDFRm3FRnzFRRLFRc4FRDYFRc1FRPjFRPKFRDvFhkdpvbqnHDhUyPsUHYkn1Dkrj0hTHY1P104PjDYn7qWTZchThcqnauzT1YkFMP-UAk-T-qGujYkFMPGujY3rARYnAmzmvc3uynsnANbFMPYpyfqrauY5gwsmvkGmvV-ujPxpAnhIAfqnHb4P1m1nzuYUHYzPW63njndrjDhIAd15HDvP104rjRvnHmhIZRqIHD4rHTvn1nhIHdCIZwsTzR1fiRzwBRzwhF9pyV-FHF7mh7GuZR-nbNWUvYhIWYzFhbqPvf4uyPWuj0&besl=6&c=news&cf=1&cvrq=3661677&eid_list=200214_202016_204854_205526_209357&expid=200214_202016_202257_202564_204854_205526_205809_206503_209394&fr=20&fv=0&haacp=1439&img_typ=0&itm=0&lu_idc=gzhxy&lukid=11&lus=88e40f2cb8ec00ed&lust=63993790&luwtr=750531414731215287&mscf=0&n=10&nttp=1&p=baidu&pbs=220093&sce=7&sr=72&ssp2=1&tpl=baiduCustITagLinkUnitRankCol&tsf=dtp:1&tu_type=0&u=%2Fitem%2F%25E8%2582%25A0%25E8%25BA%2581%25E7%2597%2587%2F86605%3FfromModule%3Dsearch%2Dresult%5Flemma&uicf=lurecv&urlid=0&eot=1) |
| [概述图册(1)](https://baike.baidu.com/pic/%E8%82%A0%E8%BA%81%E7%97%87/86605/1/e1fe9925bc315c60daebf9fe8bb1cb13495477a1?fr=lemma) | | | | |  |
| 内容来自  学术论文 | | | | |  |
| [陶诗秀. 肠躁症的饮食治疗．](https://xueshu.baidu.com/usercenter/paper/show?paperid=23553da467c75c47d21d877926ce9136&tn=SE_baiduxueshu_c1gjeupa&ie=utf-8&site=baike) 《vip》， 2016  [陶诗秀. 肠躁症的饮食治疗．](https://xueshu.baidu.com/usercenter/paper/show?paperid=1v7w0gm0986r0c60gk7a02t0bw523816&tn=SE_baiduxueshu_c1gjeupa&ie=utf-8&site=baike) 《现代养生》， 2016  [欣欣. 肠躁症又是什么?．](https://xueshu.baidu.com/usercenter/paper/show?paperid=5bab0c6b648452a9c14f7306233e6bef&tn=SE_baiduxueshu_c1gjeupa&ie=utf-8&site=baike) 《健康之家》， 2016  [偏头痛与肠躁症有关．](https://xueshu.baidu.com/usercenter/paper/show?paperid=df50af3f4c2e994f84d1022899d79669&tn=SE_baiduxueshu_c1gjeupa&ie=utf-8&site=baike) 《vip》， 2017  [查看全部](https://xueshu.baidu.com/s?wd=intitle%3A%28%E8%82%A0%E8%BA%81%E7%97%87%29&tn=SE_baiduxueshu_c1gjeupa&ie=utf-8&sc_from=pingtai6&site=baike) | | | | | |

| 岔 搜索发现  [胶囊胃镜](https://www.baidu.com/s?word=%E8%83%B6%E5%9B%8A%E8%83%83%E9%95%9C&tn=SE_baikepcxf02_fcetbk02&pos=baike_pc_turbo_1767&ori_sid=00bb350e90098018)  [肠躁症](https://www.baidu.com/s?word=%E8%82%A0%E8%BA%81%E7%97%87&tn=SE_baikepcxf02_fcetbk02&pos=baike_pc_turbo_1767&ori_sid=00bb350e90098018) | [胃肠胀气](https://www.baidu.com/s?word=%E8%83%83%E8%82%A0%E8%83%80%E6%B0%94&tn=SE_baikepcxf02_fcetbk02&pos=baike_pc_turbo_1767&ori_sid=00bb350e90098018)  [肠燥症症状](https://www.baidu.com/s?word=%E8%82%A0%E7%87%A5%E7%97%87%E7%97%87%E7%8A%B6&tn=SE_baikepcxf02_fcetbk02&pos=baike_pc_turbo_1767&ori_sid=00bb350e90098018) | [肠道功能紊乱的症状](https://www.baidu.com/s?word=%E8%82%A0%E9%81%93%E5%8A%9F%E8%83%BD%E7%B4%8A%E4%B9%B1%E7%9A%84%E7%97%87%E7%8A%B6&tn=SE_baikepcxf02_fcetbk02&pos=baike_pc_turbo_1767&ori_sid=00bb350e90098018) [肠燥症怎么办](https://www.baidu.com/s?word=%E8%82%A0%E7%87%A5%E7%97%87%E6%80%8E%E4%B9%88%E5%8A%9E&tn=SE_baikepcxf02_fcetbk02&pos=baike_pc_turbo_1767&ori_sid=00bb350e90098018) | [肠炎的治法](https://www.baidu.com/s?word=%E8%82%A0%E7%82%8E%E7%9A%84%E6%B2%BB%E6%B3%95&tn=SE_baikepcxf02_fcetbk02&pos=baike_pc_turbo_1767&ori_sid=00bb350e90098018)  [教师资格证考试时间](https://www.baidu.com/s?word=%E6%95%99%E5%B8%88%E8%B5%84%E6%A0%BC%E8%AF%81%E8%80%83%E8%AF%95%E6%97%B6%E9%97%B4&tn=SE_baikepcxf02_fcetbk02&pos=baike_pc_turbo_1767&ori_sid=00bb350e90098018) | [肚子胀痛快速解决办法](https://www.baidu.com/s?word=%E8%82%9A%E5%AD%90%E8%83%80%E7%97%9B%E5%BF%AB%E9%80%9F%E8%A7%A3%E5%86%B3%E5%8A%9E%E6%B3%95&tn=SE_baikepcxf02_fcetbk02&pos=baike_pc_turbo_1767&ori_sid=00bb350e90098018) [中专学校](https://www.baidu.com/s?word=%E4%B8%AD%E4%B8%93%E5%AD%A6%E6%A0%A1&tn=SE_baikepcxf02_fcetbk02&pos=baike_pc_turbo_1767&ori_sid=00bb350e90098018) |
| --- | --- | --- | --- | --- |

Q

新手上路 [成长任务](https://baike.baidu.com/usercenter/tasks#guide)

[编辑规则](https://baike.baidu.com/help#main06)

邮 我有疑问

目 投诉建议

[编辑入门](https://baike.baidu.com/help#main01) [内容质疑](javascript:void(0);)

[在线客服](http://zhiqiu.baidu.com/baike/passport/html/baikechat.html) [意见反馈](javascript:void(0);)

[举报不良信息](http://help.baidu.com/newadd?word=%E8%82%A0%E8%BA%81%E7%97%87&&submit_link=https%3A%2F%2Fbaike.baidu.com%2Fitem%2F%25E8%2582%25A0%25E8%25BA%2581%25E7%2597%2587%2F86605%3FfromModule%3Dsearch-result_lemma&prod_id=10&category=1) [投诉侵权信息](http://help.baidu.com/newadd?word=%E8%82%A0%E8%BA%81%E7%97%87&&submit_link=https%3A%2F%2Fbaike.baidu.com%2Fitem%2F%25E8%2582%25A0%25E8%25BA%2581%25E7%2597%2587%2F86605%3FfromModule%3Dsearch-result_lemma&prod_id=10&category=6)

[本人编辑](https://baike.baidu.com/item/%E7%99%BE%E5%BA%A6%E7%99%BE%E7%A7%91%EF%BC%9A%E6%9C%AC%E4%BA%BA%E8%AF%8D%E6%9D%A1%E7%BC%96%E8%BE%91%E6%9C%8D%E5%8A%A1/22442459?bk_fr=pcFooter) [官方贴吧](http://tieba.baidu.com/f?ie=utf-8&fr=bks0000&kw=%E7%99%BE%E5%BA%A6%E7%99%BE%E7%A7%91)

©2022 Baidu [使用百度前必读](http://www.baidu.com/duty/) | [百科协议](http://help.baidu.com/question?prod_en=baike&class=89&id=1637) | [隐私政策](http://help.baidu.com/question?prod_id=10&class=690&id=1001779) | [百度百科合作平台](https://baike.baidu.com/operation/cooperation) | 京ICP证030173号 [京公网安备11000002000001号](http://www.beian.gov.cn/portal/registerSystemInfo?recordcode=11000002000001)

[未通过词条申诉](http://help.baidu.com/newadd?word=%E8%82%A0%E8%BA%81%E7%97%87&&submit_link=https%3A%2F%2Fbaike.baidu.com%2Fitem%2F%25E8%2582%25A0%25E8%25BA%2581%25E7%2597%2587%2F86605%3FfromModule%3Dsearch-result_lemma&prod_id=10&category=2)

[封禁查询与解封](http://help.baidu.com/newadd?word=%E8%82%A0%E8%BA%81%E7%97%87&&submit_link=https%3A%2F%2Fbaike.baidu.com%2Fitem%2F%25E8%2582%25A0%25E8%25BA%2581%25E7%2597%2587%2F86605%3FfromModule%3Dsearch-result_lemma&prod_id=10&category=5)

<https://baike.baidu.com/item/>肠躁症/86605?fromModule=search-result_lemma

1/ 1
