# Supplementary material for: Assessment of medical information on irritable bowel syndrome information in Wikipedia and Baidu Encyclopedia: comparative study
Source: PeerJ. 2024 May 24;12:e17264. doi: 10.7717/peerj.17264 (PMC11129691; doi:10.7717/peerj.17264)
Supplement: Data S1 [file peerj-12-17264-s001.zip › σÄƒσoïμò░μì«/Baidu/Baidu-English/2-Φéáμÿôμ┐Çτ╗╝σÉêσ╛ü∩╝êΦéáμÿôμ┐Çτ╗╝σÉêσ╛ü∩╝ë_τÖ╛σ║aτÖ╛τoæ.docx]

2022/12/14 10:30

[岔](https://baike.baidu.com/)

肠易激综合征

进入词条

[岔](https://baike.baidu.com/)

肠易激综合征(肠易激综合征) _百度百科

| ~~[网页](https://www.baidu.com/) [新闻](http://news.baidu.com/) [贴吧](https://tieba.baidu.com/)~~  [知道](https://zhidao.baidu.com/) | | | | | [网盘](https://pan.baidu.com/?from=1027327l) | [图片](http://image.baidu.com/) | [视频](http://v.baidu.com/)    播报 | | [地图](http://map.baidu.com/) | [文库](https://wenku.baidu.com/)  编辑 | | 百科  收藏 | [百度首页](http://www.baidu.com/) 赞 | [登录](javascript:;) |
| --- | --- | --- | --- | --- | --- | --- | --- | --- | --- | --- | --- | --- | --- | --- |
|  | | | | | | | | | | | | | | |
|  | |  | | | | | |  | | |  | |  | |
|  | | 肠易激综合征 | | | | | | 进入词条 | | | 全站搜索 | | [帮助](https://baike.baidu.com/help) | |
| 近期有不法分子冒充百度百科官方人员，以删除词条为由威胁并敲诈相关企业。在此严正声明：百度百科是免费编辑平台，绝不存在收费代编服务，请勿上当受骗！ [详情>>](https://baike.baidu.com/common/declaration) | | | | | | | | | | | | |  |  |
| [首页](https://baike.baidu.com/) 秒懂百科 特色百科 用户 知识专题 权威合作 [口下载百科APP](https://baike.baidu.com/wapui/subpage/baikeappdownload?sfrom=pc_lemmapage_navigation) [2 个](https://baike.baidu.com/usercenter) | | | | | | | | | | | | | | |
| 肠易激综合征是一个[多义词](https://baike.baidu.com/item/%E7%99%BE%E5%BA%A6%E7%99%BE%E7%A7%91%EF%BC%9A%E5%A4%9A%E4%B9%89%E8%AF%8D)，请在下列[义项](https://baike.baidu.com/item/%E4%B9%89%E9%A1%B9)上选择浏览([共3个义项](https://baike.baidu.com/item/%E8%82%A0%E6%98%93%E6%BF%80%E7%BB%BC%E5%90%88%E5%BE%81?force=1)) [展开 添加义项](javascript:;)      肠易激综合征可防可治，三种治疗方 …  05:25    肠易激综合征  [编辑](javascript:;)  [播报](javascript:;)  [上传视频](javascript:;)      什么是肠易激综合征？  02:22 | | | | | | | | | | | | | | |
| [女疊](javascript:void(0);)  收藏  [2411](javascript:void(0);) 556  外文名 irritable bowel syndrome  专家解读   \| 就诊科室 \| 消化内科 \| \| --- \| --- \| \| 多发群体 \| 20 ~ 50岁女性 \|   [肠易激综合征如何治疗？](javascript:;)  [对肠易激综合征的一般治疗包括建立良好的](javascript:;)  [生活习惯，饮食上避免产气的食物。](javascript:;)   \| 科普中国  本词条由[“科普中国”科学百科词条编写与应用工作项目](https://baike.baidu.com/science) 认证 。  Irritable bowel syndrome (IBS) is a group of persistent or intermittent episodes of intestinal dysfunction characterized by abdominal pain, bloating, bowel habits, and/or changes in stool characteristics, and lack of gastrointestinal structures and biochemical abnormalities. Rome III classified it as a category of functional bowel disease, patients are mainly young and middle-aged, the age of onset is more common in 20 ~ 50 years old, women are more common than men, there is a tendency to family aggregation, often coexisting with other gastrointestinal dysfunction diseases such as functional dyspepsia. According to the characteristics of stool, IBS is divided into four clinical types: diarrhea, constipation, mixed and amorphous, and diarrhea is more common in China.  审核  致力于权威的科学传播  本词条认证专家为  屠惠明 丨主任医师   \|  \| \| --- \|   无锡市第四人民医院 消化内  审核  韩英 丨主任医师   \|  \| \| --- \|   陆军总院 消化科 \| \| --- \| --- \| --- \|   多与精神因素、应激状态及肠功能…  常见病因 | | | | | | | | | | | | | | |
|  | | | | | | | | | | | | | | |
| 疾病概况 | 科普文章 (1) | | 科普视频 | [口](javascript:void(0);) | | | | | | | | | | |
| \|  \| \| --- \|  \| 权威合作编辑  [“科普中国”科学百科词条编](http://www.kepuchina.cn/)  “科普中国”是为我国科普信 建设塑造的全...  [什么是权威编辑](http://baike.bdimg.com/cms/static/cooperation/content.pdf) \| \| --- \|  \|  \| \| --- \|  \| 目录 \| 1 [病因](#_bookmark1)  2 [临床表现](#_bookmark2)  3 [检查](#_bookmark3)  4 [诊断](#_bookmark4)  5 [治疗](#_bookmark5) \| \| --- \| --- \|   基本信息   \| 资源提供  [科普中国](http://www.cast.org.cn/)  “科普中国”是中国科协... 提供资源类型： 资源  [什么是资源合作](http://baike.bdimg.com/cms/static/cooperation/content.pdf) \| \| --- \|   irritable bowel syndrome  多与精神因素、应激状态及肠功能紊乱有关 腹痛、腹胀、腹泻、便秘、烧心、恶心、呕吐  无  常见病因 常见症状  传染性  外文名 就诊科室  多发群体  消化内科  20 ~ 50岁女性  [播报编辑](javascript:;)  词条统计  病因  浏览次数： 4464651次  The etiology and pathogenesis of IBS are not well understood, but are thought to be a variety of factors such as abnormal gastrointestinal motility, visceral paresthesia, cerebral and gastrointestinal regulation abnormalities, inflammation, and psychological  The result of a joint action.  1. Gastrointestinal motility disorders  Changes in intestinal motility are an important pathophysiology underpinning the development of IBS symptoms. IBS patients with diarrhea showed intestinal hypermotility, and the small intestinal transit time was significantly reduced  Short, colonic motility index and high propulsive contraction were significantly increased in mean and maximum. Constipation-type IBS is the opposite, manifested by insufficient intestinal motility. | | | | | | | | | | | | | | |

<https://baike.baidu.com/item/>肠易激综合征/8456761?fromModule=lemma_inlink

1/4

2022/12/14 10:30

肠易激综合征(肠易激综合征) _百度百科

| gjhxw  [岔](https://baike.baidu.com/)  [编辑](javascript:;)  [收藏](javascript:;)  小 播报  [赞](javascript:;)  **2.**内脏感觉异常  研究发现IBS患者多数具有对管腔(直肠)扩张感觉过敏的临床特征，其平均痛觉阈值下降，直肠扩张后的不适程度增强或 | | |
| --- | --- | --- |
| Abnormal visceral-somatic radiating pain suggests abnormal sensory signal processing at the spinal cord level.  **3. Central paresthesia**  **Studies have shown that the central pathway of visceral pain in IBS patients is different from that of normal people, and the brain response area between diarrheal IBS and constipated IBS is also different.**  **4. Abnormal regulation of the brain-gut axis**  **Patients with IBS have abnormalities in the central nervous system's processing of intestinal afferent signals and regulation of the enteric nervous system.**  **5. Intestinal infection and inflammatory response**  **Studies have shown that the incidence of IBS after acute intestinal infection is greatly increased, so acute intestinal infection is considered to be one of the risk factors for IBS. The relationship between mucosal inflammation, increased permeability and immune function activation caused by intestinal infection and the incidence of IBS deserves further study.**  **6. Psychopsychological factors**  **IBS patients often have psychological abnormalities such as anxiety, nervousness, and depression. At the same time, mental and psychological stress can also induce or aggravate the symptoms of IBS, indicating that mental and psychological factors are closely related to IBS.** | | [女疊 口](javascript:void(0);) |
| 临床表现 | [小 播报编辑](javascript:;) |  |
| IBS has no specific symptoms, but has some features relative to organic gastrointestinal disease: slow onset, intermittent onset; The course of the disease is long but the general health condition is not affected; The onset or worsening of symptoms is often related to psychiatric factors or stressful states; It is noticeable during the day and relieves after sleep at night.  1. Symptoms  (1) Abdominal pain or abdominal discomfort is the main symptom of IBS, accompanied by abnormal stool frequency or shape, abdominal pain is more than relieved after defecation, some patients are prone to appear after eating, abdominal pain can occur in any part of the abdomen, localized or diffuse, pain nature is diverse. Abdominal pain does not worsen progressively, and people rarely wake up after nighttime sleep.  (2) Diarrhea (1) Persistent or intermittent diarrhea, small feces, mushy, containing a large amount of mucus; (2) Symptoms disappear after 72 hours of fasting; (3) It does not appear at night, which is different from organic diseases; (4) Some patients can be induced by eating; (5) Patients may have diarrhea and constipation alternating.  (3) Constipation Difficulty in defecation, dry stool, less amount, can bring more mucus, constipation can be interrupted or alternating with diarrhea, often accompanied by incomplete defecation.  (4) Abdominal distension is heavier during the day, especially in the afternoon, and relieved after sleep at night.  (5) Upper gastrointestinal symptoms Nearly half of the patients have upper gastrointestinal symptoms such as heartburn, nausea, and vomiting.  (6) Intestinal symptoms Back pain, headache, palpitations, frequent urination, urgency, sexual dysfunction and other extragastrointestinal manifestations are significantly more common than organic bowel diseases, and some patients still have different degrees of psychological and mental abnormalities, such as anxiety, depression, nervousness, etc.  2. Signs  There are usually no positive findings, or only mild abdominal tenderness. Some patients have autonomic disorders such as hyperhidrosis, fast pulse, high blood pressure, and sometimes palpation of sigmoid flexure or painful intestinal loops in the abdomen. Digital rectal examination may feel spasm, high tension, and tenderness. | |  |
| 检查 | [小 播报编辑](javascript:;) |  |
| To rule out organic disease, multiple (at least 3) stool routine cultures were negative, fecal occult blood test was negative, hematuria routine was normal, erythrocyte sedimentation rate was normal, and thyroid, liver, gallbladder, pancreas, and kidney functions were normal. For patients over the age of 40, in addition to the above examinations, colonoscopy and mucosal biopsy should be performed to exclude intestinal infection and neoplastic diseases. Barium enema X-ray examination and abdominal ultrasonography are also commonly used to exclude the diagnosis. | |  |
| 诊断 | [小 播报编辑](javascript:;) |  |
| The diagnostic criteria for IBS are symptomatic-based, the diagnosis is based on the exclusion of organic diseases, and the currently internationally recognized IBS Rome III diagnostic criteria are recommended:  Recurrent episodes of abdominal pain or malaise (discomfort means feeling unwell rather than painful), with symptoms occurring at least 3 days a month in the last 3 months, combined with two or more of the following: (1) relief of symptoms after bowel movements; (2) changes in bowel frequency at the onset of the attack, and (3) changes in stool characteristics (appearance) at the onset.  Pre-diagnosis symptoms have been present for at least 6 months and the above criteria have been met in the past 3 months.  The following symptoms are supportive of the diagnosis, including (1) abnormal bowel movements (less than 3 bowel movements per week or more than 3 times a day), ;(2) abnormal stool characteristics (dry or hard stool, or mushy/dilute stool), ;(3) laborious bowel movements, and (4) urgency, incomplete bowel movements, mucus stool, and bloating. | |  |
| 治疗 | [小 播报编辑](javascript:;) |  |
| Treatment of IBS is limited to symptomatic management. The Gastrointestinal Kinetics Group of the Gastroenterology Branch of the Chinese Medical Association proposed in the "Consensus Opinions on the Diagnosis and Treatment of Irritable Bowel Syndrome": "The purpose of treatment is to eliminate patients' concerns, improve symptoms and improve quality of life. The principle of treatment is based on a good doctor-patient relationship, symptomatic treatment according to the main symptom type and graded treatment according to symptom severity. Pay attention to the individualization and comprehensive use of treatment measures. ” | |  |

<https://baike.baidu.com/item/>肠易激综合征/8456761?fromModule=lemma_inlink

2/4

2022/12/14 10:30

肠易激综合征(肠易激综合征) _百度百科

| 状治疗和根据症状严重程度进行分级治疗。注意治疗措施的个体化和综合运用。 ”  小 播报  [编辑](javascript:;)  [收藏](javascript:;)  [赞](javascript:;)  [时岔謂](https://baike.baidu.com/)  **1.**调整饮食 | | | |
| --- | --- | --- | --- |
|  | | | |
| Learn more about the patient's eating habits and their relationship with symptoms, avoid sensitive foods, avoid excessive fat and irritating foods such as coffee, strong tea, alcohol, etc., and reduce the intake of gas-producing foods (dairy products, soybeans, lentils, etc.). High-fiber foods (such as bran) can stimulate colon movement and have a significant effect on improving constipation.  2. Psychological and behavioral therapy  Patiently explain the patient, including psychotherapy, biofeedback therapy, etc., and sedative drugs can be given appropriately for those with insomnia, anxiety and other symptoms.  3. Medication  (1) Antispasmodic agents At present, selective intestinal smooth muscle calcium channel antagonists or ion channel modulators are more commonly used. Anticholinergics such as atropine, belladonna, and hyoscyamine can also improve abdominal pain symptoms, but adverse effects should be noted.  (2) Laxatives can be used for constipation, and volumetric laxatives such as methylcellulose and osmotic laxatives such as polyethylene glycol, lactulose, etc. can be tried. Stimulant laxatives should be used with caution.  (3) Antidiarrheal agents such as loperamide or compound phenethylpiperidine can improve diarrhea, and it is necessary to pay attention to adverse reactions such as constipation and bloating. Patients with mild disease can choose octahedral montmorillonite and other adjectives.  (4) Prokinetic agents are suitable for patients with bloating and constipation. Commonly used are cisapyride or mosapride.  (5) Visceral analgesics Somatostatin and its analogues such as octreotide have the effect of relieving somatic and visceral pain. 5-HT3 receptor blockers such as alosetron can improve  Abdominal pain and frequency of stool in patients with diarrhea type can cause adverse reactions such as ischemic colitis, and attention should be paid to when using. Some agonists of the 5-HT4 receptor have been discontinued due to their increased risk of cardiovascular ischemic events.  (6) Antipsychotics for patients with severe abdominal pain symptoms and the above treatment is ineffective, especially patients with obvious mental symptoms, appropriate sedatives, antidepressants, anxiolytics have a certain help.  (7) Probiotics can adjust the ecological balance of the host intestinal microbiota and are suitable for IBS patients with intestinal dysbacteriosis.  4. Traditional Chinese medicine treatment  Traditional Chinese medicine divides IBS into large intestine dry heat evidence, cold and heat inclusion evidence, spleen and stomach weakness certificate, liver qi multiplied by spleen evidence, liver depression and qi stagnation and other types of evidence, through dialectical treatment, acupuncture and other methods, the treatment of IBS has achieved certain curative effects, still need to be further studied in the future. | | | [女](javascript:void(0);) [公](javascript:void(0);) [.](javascript:void(0);) [疊](javascript:void(0);) [口](javascript:void(0);) |
| 学术论文 | | 内容来自 |  |
| [Wang等. 中医治疗肠易激综合征的研究进展：肠易激综合征中医证候学特点的认](https://xueshu.baidu.com/usercenter/paper/show?paperid=78a927fc08b2691ebc68d605d8782574&tn=SE_baiduxueshu_c1gjeupa&ie=utf-8&site=baike) 《世界华人消化杂志》， 2010  [徐启旺，钱家鸣，窦君，余平. 细菌潜生体相关的肠易激综合征动物模型的建立及细菌潜生体在肠易激](https://xueshu.baidu.com/usercenter/paper/show?paperid=a3b9f8b3196ac7e7060e220463381710&tn=SE_baiduxueshu_c1gjeupa&ie=utf-8&site=baike) 《胃肠病学》， 2003  [肠易激综合征联合治疗对肠易激综合征病人心理精神因素的疗效评价．](https://xueshu.baidu.com/usercenter/paper/show?paperid=9b7dc75fd3592c42014a83aaa7e5b0ac&tn=SE_baiduxueshu_c1gjeupa&ie=utf-8&site=baike)陈艳. 《临床和实验医学杂志》， 2006  [陈达氏. 肠易激综合征患者的胃肠动力：肠易激综合征是否为一种动力紊乱．](https://xueshu.baidu.com/usercenter/paper/show?paperid=bbe2321662e895447e4461a48598b550&tn=SE_baiduxueshu_c1gjeupa&ie=utf-8&site=baike) 《vip》， 1994  [胡品津. 肠易激综合征诊治共识意见．](https://xueshu.baidu.com/usercenter/paper/show?paperid=bc9d014868c0ff198ec4f033c85d6ead&tn=SE_baiduxueshu_c1gjeupa&ie=utf-8&site=baike) 《 CNKI》， 2003  [查看全部](https://xueshu.baidu.com/s?wd=%E8%82%A0%E6%98%93%E6%BF%80%E7%BB%BC%E5%90%88%E5%BE%81+%E8%82%A0%E6%98%93%E6%BF%80%E7%BB%BC%E5%90%88%E5%BE%81&tn=SE_baiduxueshu_c1gjeupa&ie=utf-8&sc_from=pingtai6&site=baike) | | |  |
|  | | | |
| 猜你喜欢 | [幽门螺旋杆菌，吃益生菌有用吗?高活性益生菌](http://www.baidu.com/baidu.php?url=Ks00000EAMrnlPLIyGAY4b9NmgBNriocqp7ssdviP3k5x_WGkL4dsRYNizs9gBg55tnfcD06t02_AheBhNtmO3AwqyMeGjv8WBZlDPYws_JSEdqb8KfdQ1Jh-04LVaibwM_WsWRDRnPEZIyNcFG2me2WuLUMoghdgMLXWOzOiBCPeuvs_zYeRmVbI9PBC1w0dfkLzkXnB88nR3i6zIrZiGWb7Lfe.7D_NR2Ar5Od66xfHGt_Vzc2eQr1k_lX1uEooo3tdPHV2XgZJyAp7WW8e2O7f.U1Yk0ZDq_lUidezbdq8l1qgL0ZfqEJv1CtaVV_xgYe5RzoQjVQojVxx0eUrh1o60pyYqnHcvn6KdpHY0TA-b5Hcs0APGujYLn6KBpHY1njD0uMfqn0KspjYs0Aq15H00mMTqn0K8IjYs0ZPl5fKzuLw9u1Ys0A4vTjYsQW0snj0snj0s0ANzu1Ys0Zwzmyw-5H00mhwGujYznRNafWb1wbNAnbfkwDm3rDNjwjf3fRf4wDDYf1mzffKbmvPb5fK9TdqGuAnqTZnVuLGCXZb0u1dLTv410ZFY5Hn4P0KkTA-b5H00TyPGujYs0A7B5HKxn0KsTjYs0AdYTjYs0AwbUL0qn0KzpWYs0ZwdT1YvnWRsn103Pjf4PHf1P1n3rHbk0A7W5HT0TA3qn0Ksmgwxuhk9u1Ys0AN1IjY1n6K-IA-b5iYk0A71TAPW5H00IgKGUhPW5H00uhPdIjYs0A7buhk9u1Yk0ZIhThqV5fKBIjYk0ZF-TgfqnHmLnjb3Pjb3rj63P6K1pyfqnjKBmWnYP1DvmH-hn1F9ufKEIjYs0AqzTZfqnanscYwANansc10WnansQW0WnaPDw-fWnaPDw-f0pvbqn0KVIjYznjmz0AdW5HcLn1D3PWT1nWTd0AdWgvuzUvYqn0Kbmy4dmhNxTAk9Uh-bT1Ys0A7bTgbq8QMf8VpBdef0mywkIjYs0A-1mvsqn0KkUgfqn0K9u7q1ULNzmvRqnWnWIv-1uNqYULKxmv7WpARWgdqxgv41cLwGIAk-cvPYmLuzTNqCXNqGTvt0mLFW5HcvrHbs&us=newvui&ai=0_429414813_1_0&word=&ck=0.0.0.0.0.0.0.0&shh=baike.baidu.com)  [幽门螺旋杆菌哪些症状，感染幽门螺旋杆菌怎么办，幽门螺杆菌是](http://www.baidu.com/baidu.php?url=Ks00000EAMrnlPLIyGAY4b9NmgBNriocqp7ssdviP3k5x_WGkL4dsRYNizs9gBg55tnfcD06t02_AheBhNtmO3AwqyMeGjv8WBZlDPYws_JSEdqb8KfdQ1Jh-04LVaibwM_WsWRDRnPEZIyNcFG2me2WuLUMoghdgMLXWOzOiBCPeuvs_zYeRmVbI9PBC1w0dfkLzkXnB88nR3i6zIrZiGWb7Lfe.7D_NR2Ar5Od66xfHGt_Vzc2eQr1k_lX1uEooo3tdPHV2XgZJyAp7WW8e2O7f.U1Yk0ZDq_lUidezbdq8l1qgL0ZfqEJv1CtaVV_xgYe5RzoQjVQojVxx0eUrh1o60pyYqnHcvn6KdpHY0TA-b5Hcs0APGujYLn6KBpHY1njD0uMfqn0KspjYs0Aq15H00mMTqn0K8IjYs0ZPl5fKzuLw9u1Ys0A4vTjYsQW0snj0snj0s0ANzu1Ys0Zwzmyw-5H00mhwGujYznRNafWb1wbNAnbfkwDm3rDNjwjf3fRf4wDDYf1mzffKbmvPb5fK9TdqGuAnqTZnVuLGCXZb0u1dLTv410ZFY5Hn4P0KkTA-b5H00TyPGujYs0A7B5HKxn0KsTjYs0AdYTjYs0AwbUL0qn0KzpWYs0ZwdT1YvnWRsn103Pjf4PHf1P1n3rHbk0A7W5HT0TA3qn0Ksmgwxuhk9u1Ys0AN1IjY1n6K-IA-b5iYk0A71TAPW5H00IgKGUhPW5H00uhPdIjYs0A7buhk9u1Yk0ZIhThqV5fKBIjYk0ZF-TgfqnHmLnjb3Pjb3rj63P6K1pyfqnjKBmWnYP1DvmH-hn1F9ufKEIjYs0AqzTZfqnanscYwANansc10WnansQW0WnaPDw-fWnaPDw-f0pvbqn0KVIjYznjmz0AdW5HcLn1D3PWT1nWTd0AdWgvuzUvYqn0Kbmy4dmhNxTAk9Uh-bT1Ys0A7bTgbq8QMf8VpBdef0mywkIjYs0A-1mvsqn0KkUgfqn0K9u7q1ULNzmvRqnWnWIv-1uNqYULKxmv7WpARWgdqxgv41cLwGIAk-cvPYmLuzTNqCXNqGTvt0mLFW5HcvrHbs&us=newvui&ai=0_429414813_1_0&word=&ck=0.0.0.0.0.0.0.0&shh=baike.baidu.com)  [胃部发炎引起的怎么治幽门螺旋杆菌，恶心干呕，易导致口臭， …](http://www.baidu.com/baidu.php?url=Ks00000EAMrnlPLIyGAY4b9NmgBNriocqp7ssdviP3k5x_WGkL4dsRYNizs9gBg55tnfcD06t02_AheBhNtmO3AwqyMeGjv8WBZlDPYws_JSEdqb8KfdQ1Jh-04LVaibwM_WsWRDRnPEZIyNcFG2me2WuLUMoghdgMLXWOzOiBCPeuvs_zYeRmVbI9PBC1w0dfkLzkXnB88nR3i6zIrZiGWb7Lfe.7D_NR2Ar5Od66xfHGt_Vzc2eQr1k_lX1uEooo3tdPHV2XgZJyAp7WW8e2O7f.U1Yk0ZDq_lUidezbdq8l1qgL0ZfqEJv1CtaVV_xgYe5RzoQjVQojVxx0eUrh1o60pyYqnHcvn6KdpHY0TA-b5Hcs0APGujYLn6KBpHY1njD0uMfqn0KspjYs0Aq15H00mMTqn0K8IjYs0ZPl5fKzuLw9u1Ys0A4vTjYsQW0snj0snj0s0ANzu1Ys0Zwzmyw-5H00mhwGujYznRNafWb1wbNAnbfkwDm3rDNjwjf3fRf4wDDYf1mzffKbmvPb5fK9TdqGuAnqTZnVuLGCXZb0u1dLTv410ZFY5Hn4P0KkTA-b5H00TyPGujYs0A7B5HKxn0KsTjYs0AdYTjYs0AwbUL0qn0KzpWYs0ZwdT1YvnWRsn103Pjf4PHf1P1n3rHbk0A7W5HT0TA3qn0Ksmgwxuhk9u1Ys0AN1IjY1n6K-IA-b5iYk0A71TAPW5H00IgKGUhPW5H00uhPdIjYs0A7buhk9u1Yk0ZIhThqV5fKBIjYk0ZF-TgfqnHmLnjb3Pjb3rj63P6K1pyfqnjKBmWnYP1DvmH-hn1F9ufKEIjYs0AqzTZfqnanscYwANansc10WnansQW0WnaPDw-fWnaPDw-f0pvbqn0KVIjYznjmz0AdW5HcLn1D3PWT1nWTd0AdWgvuzUvYqn0Kbmy4dmhNxTAk9Uh-bT1Ys0A7bTgbq8QMf8VpBdef0mywkIjYs0A-1mvsqn0KkUgfqn0K9u7q1ULNzmvRqnWnWIv-1uNqYULKxmv7WpARWgdqxgv41cLwGIAk-cvPYmLuzTNqCXNqGTvt0mLFW5HcvrHbs&us=newvui&ai=0_429414813_1_0&word=&ck=0.0.0.0.0.0.0.0&shh=baike.baidu.com)  [item.jd.com](http://www.baidu.com/baidu.php?url=Ks00000EAMrnlPLIyGAY4b9NmgBNriocqp7ssdviP3k5x_WGkL4dsRYNizs9gBg55tnfcD06t02_AheBhNtmO3AwqyMeGjv8WBZlDPYws_JSEdqb8KfdQ1Jh-04LVaibwM_WsWRDRnPEZIyNcFG2me2WuLUMoghdgMLXWOzOiBCPeuvs_zYeRmVbI9PBC1w0dfkLzkXnB88nR3i6zIrZiGWb7Lfe.7D_NR2Ar5Od66xfHGt_Vzc2eQr1k_lX1uEooo3tdPHV2XgZJyAp7WW8e2O7f.U1Yk0ZDq_lUidezbdq8l1qgL0ZfqEJv1CtaVV_xgYe5RzoQjVQojVxx0eUrh1o60pyYqnHcvn6KdpHY0TA-b5Hcs0APGujYLn6KBpHY1njD0uMfqn0KspjYs0Aq15H00mMTqn0K8IjYs0ZPl5fKzuLw9u1Ys0A4vTjYsQW0snj0snj0s0ANzu1Ys0Zwzmyw-5H00mhwGujYznRNafWb1wbNAnbfkwDm3rDNjwjf3fRf4wDDYf1mzffKbmvPb5fK9TdqGuAnqTZnVuLGCXZb0u1dLTv410ZFY5Hn4P0KkTA-b5H00TyPGujYs0A7B5HKxn0KsTjYs0AdYTjYs0AwbUL0qn0KzpWYs0ZwdT1YvnWRsn103Pjf4PHf1P1n3rHbk0A7W5HT0TA3qn0Ksmgwxuhk9u1Ys0AN1IjY1n6K-IA-b5iYk0A71TAPW5H00IgKGUhPW5H00uhPdIjYs0A7buhk9u1Yk0ZIhThqV5fKBIjYk0ZF-TgfqnHmLnjb3Pjb3rj63P6K1pyfqnjKBmWnYP1DvmH-hn1F9ufKEIjYs0AqzTZfqnanscYwANansc10WnansQW0WnaPDw-fWnaPDw-f0pvbqn0KVIjYznjmz0AdW5HcLn1D3PWT1nWTd0AdWgvuzUvYqn0Kbmy4dmhNxTAk9Uh-bT1Ys0A7bTgbq8QMf8VpBdef0mywkIjYs0A-1mvsqn0KkUgfqn0K9u7q1ULNzmvRqnWnWIv-1uNqYULKxmv7WpARWgdqxgv41cLwGIAk-cvPYmLuzTNqCXNqGTvt0mLFW5HcvrHbs&us=newvui&ai=0_429414813_1_0&word=&ck=0.0.0.0.0.0.0.0&shh=baike.baidu.com) | | |

| 岔 搜索发现  [治疗肠易激的中成药](https://www.baidu.com/s?word=%E6%B2%BB%E7%96%97%E8%82%A0%E6%98%93%E6%BF%80%E7%9A%84%E4%B8%AD%E6%88%90%E8%8D%AF&tn=SE_baikepcxf02_fcetbk02&pos=baike_pc_turbo_1767&ori_sid=00bb34716a7c54d6) [肠激惹综合症吃什么药](https://www.baidu.com/s?word=%E8%82%A0%E6%BF%80%E6%83%B9%E7%BB%BC%E5%90%88%E7%97%87%E5%90%83%E4%BB%80%E4%B9%88%E8%8D%AF&tn=SE_baikepcxf02_fcetbk02&pos=baike_pc_turbo_1767&ori_sid=00bb34716a7c54d6) | [肝郁脾虚最好中成药](https://www.baidu.com/s?word=%E8%82%9D%E9%83%81%E8%84%BE%E8%99%9A%E6%9C%80%E5%A5%BD%E4%B8%AD%E6%88%90%E8%8D%AF&tn=SE_baikepcxf02_fcetbk02&pos=baike_pc_turbo_1767&ori_sid=00bb34716a7c54d6)  [肠易激综合征的症状特点](https://www.baidu.com/s?word=%E8%82%A0%E6%98%93%E6%BF%80%E7%BB%BC%E5%90%88%E5%BE%81%E7%9A%84%E7%97%87%E7%8A%B6%E7%89%B9%E7%82%B9&tn=SE_baikepcxf02_fcetbk02&pos=baike_pc_turbo_1767&ori_sid=00bb34716a7c54d6) | [中医治疗肠易激综合征](https://www.baidu.com/s?word=%E4%B8%AD%E5%8C%BB%E6%B2%BB%E7%96%97%E8%82%A0%E6%98%93%E6%BF%80%E7%BB%BC%E5%90%88%E5%BE%81&tn=SE_baikepcxf02_fcetbk02&pos=baike_pc_turbo_1767&ori_sid=00bb34716a7c54d6)  [肠易激综合征病因](https://www.baidu.com/s?word=%E8%82%A0%E6%98%93%E6%BF%80%E7%BB%BC%E5%90%88%E5%BE%81%E7%97%85%E5%9B%A0&tn=SE_baikepcxf02_fcetbk02&pos=baike_pc_turbo_1767&ori_sid=00bb34716a7c54d6) | [拉肚子拉水止泻小妙招](https://www.baidu.com/s?word=%E6%8B%89%E8%82%9A%E5%AD%90%E6%8B%89%E6%B0%B4%E6%AD%A2%E6%B3%BB%E5%B0%8F%E5%A6%99%E6%8B%9B&tn=SE_baikepcxf02_fcetbk02&pos=baike_pc_turbo_1767&ori_sid=00bb34716a7c54d6) [肠易激综合症怎么调理](https://www.baidu.com/s?word=%E8%82%A0%E6%98%93%E6%BF%80%E7%BB%BC%E5%90%88%E7%97%87%E6%80%8E%E4%B9%88%E8%B0%83%E7%90%86&tn=SE_baikepcxf02_fcetbk02&pos=baike_pc_turbo_1767&ori_sid=00bb34716a7c54d6) | | [肠应激性综合症](https://www.baidu.com/s?word=%E8%82%A0%E5%BA%94%E6%BF%80%E6%80%A7%E7%BB%BC%E5%90%88%E7%97%87&tn=SE_baikepcxf02_fcetbk02&pos=baike_pc_turbo_1767&ori_sid=00bb34716a7c54d6)  [肠易激综合症自愈方法](https://www.baidu.com/s?word=%E8%82%A0%E6%98%93%E6%BF%80%E7%BB%BC%E5%90%88%E7%97%87%E8%87%AA%E6%84%88%E6%96%B9%E6%B3%95&tn=SE_baikepcxf02_fcetbk02&pos=baike_pc_turbo_1767&ori_sid=00bb34716a7c54d6) | |
| --- | --- | --- | --- | --- | --- | --- |
| Q 新手上路 | | 邮 我有疑问 | | 目 投诉建议 | | |
| [成长任务](https://baike.baidu.com/usercenter/tasks#guide) [编辑入门](https://baike.baidu.com/help#main01) | | [内容质疑](javascript:void(0);) [在线客服](http://zhiqiu.baidu.com/baike/passport/html/baikechat.html) | | [举报不良信息](http://help.baidu.com/newadd?word=%E8%82%A0%E6%98%93%E6%BF%80%E7%BB%BC%E5%90%88%E5%BE%81&&submit_link=https%3A%2F%2Fbaike.baidu.com%2Fitem%2F%25E8%2582%25A0%25E6%2598%2593%25E6%25BF%2580%25E7%25BB%25BC%25E5%2590%2588%25E5%25BE%2581%2F8456761%3FfromModule%3Dlemma_inlink&prod_id=10&category=1) [未通过词条申诉](http://help.baidu.com/newadd?word=%E8%82%A0%E6%98%93%E6%BF%80%E7%BB%BC%E5%90%88%E5%BE%81&&submit_link=https%3A%2F%2Fbaike.baidu.com%2Fitem%2F%25E8%2582%25A0%25E6%2598%2593%25E6%25BF%2580%25E7%25BB%25BC%25E5%2590%2588%25E5%25BE%2581%2F8456761%3FfromModule%3Dlemma_inlink&prod_id=10&category=2) | | |
| [编辑规则](https://baike.baidu.com/help#main06) [本人编辑](https://baike.baidu.com/item/%E7%99%BE%E5%BA%A6%E7%99%BE%E7%A7%91%EF%BC%9A%E6%9C%AC%E4%BA%BA%E8%AF%8D%E6%9D%A1%E7%BC%96%E8%BE%91%E6%9C%8D%E5%8A%A1/22442459?bk_fr=pcFooter) | | [官方贴吧](http://tieba.baidu.com/f?ie=utf-8&fr=bks0000&kw=%E7%99%BE%E5%BA%A6%E7%99%BE%E7%A7%91) [意见反馈](javascript:void(0);) | | [投诉侵权信息](http://help.baidu.com/newadd?word=%E8%82%A0%E6%98%93%E6%BF%80%E7%BB%BC%E5%90%88%E5%BE%81&&submit_link=https%3A%2F%2Fbaike.baidu.com%2Fitem%2F%25E8%2582%25A0%25E6%2598%2593%25E6%25BF%2580%25E7%25BB%25BC%25E5%2590%2588%25E5%25BE%2581%2F8456761%3FfromModule%3Dlemma_inlink&prod_id=10&category=6) [封禁查询与解封](http://help.baidu.com/newadd?word=%E8%82%A0%E6%98%93%E6%BF%80%E7%BB%BC%E5%90%88%E5%BE%81&&submit_link=https%3A%2F%2Fbaike.baidu.com%2Fitem%2F%25E8%2582%25A0%25E6%2598%2593%25E6%25BF%2580%25E7%25BB%25BC%25E5%2590%2588%25E5%25BE%2581%2F8456761%3FfromModule%3Dlemma_inlink&prod_id=10&category=5) | |  |

<https://baike.baidu.com/item/>肠易激综合征/8456761?fromModule=lemma_inlink

3/4

2022/12/14 10:30

[女](javascript:void(0);) [疊](http://baike.baidu.com/l/WWoXYu7P) [口](javascript:void(0);)

©2022 Baidu 使用百度前必读 | 百科协议 | 隐私政策 | 百度百科合作平台 | 京ICP证030173号 [京公网安备11000002000001号](http://www.beian.gov.cn/portal/registerSystemInfo?recordcode=11000002000001)

肠易激综合征(肠易激综合征) _百度百科

[岔](https://baike.baidu.com/)

小 播报

[编辑](javascript:;)

[收藏](javascript:;)

[赞](javascript:;)

<https://baike.baidu.com/item/>肠易激综合征/8456761?fromModule=lemma_inlink

4/4
