# Supplementary material for: Assessment of medical information on irritable bowel syndrome information in Wikipedia and Baidu Encyclopedia: comparative study
Source: PeerJ. 2024 May 24;12:e17264. doi: 10.7717/peerj.17264 (PMC11129691; doi:10.7717/peerj.17264)
Supplement: Data S1 [file peerj-12-17264-s001.zip › σÄƒσoïμò░μì«/Baidu/Baidu-English/8-σèƒΦâ╜μÇoΣ╛┐τoÿ_τÖ╛σ║aτÖ╛τoæ.docx]

功能性便秘_百度百科

2022/12/14 10:43

[贴吧](https://tieba.baidu.com/) [知道](https://zhidao.baidu.com/) [网盘](https://pan.baidu.com/?from=1027327l) [图片](http://image.baidu.com/)

[百度首页](http://www.baidu.com/) [登录](javascript:;)

[新闻](http://news.baidu.com/)

[视频](http://v.baidu.com/)

[地图](http://map.baidu.com/)

[文库](https://wenku.baidu.com/)

百科

[网页](https://www.baidu.com/)

[岔](https://baike.baidu.com/)

| 功能性便秘 | 进入词条 |
| --- | --- |

| 全站搜索 |
| --- |

[帮助](https://baike.baidu.com/help)

[首页](https://baike.baidu.com/)

秒懂百科

特色百科

用户

知识专题

权威合作

[口下载百科APP](https://baike.baidu.com/wapui/subpage/baikeappdownload?sfrom=pc_lemmapage_navigation)

[2 个](https://baike.baidu.com/usercenter)

| 功能性便秘   \| [小播报](javascript:;) \| \| --- \|   本词条由[好大夫在线](http://www.haodf.com/)提供内容并参与编辑 。 | | \| [a锁定](https://baike.baidu.com/item/%E7%99%BE%E5%BA%A6%E7%99%BE%E7%A7%91%EF%BC%9A%E9%94%81%E5%AE%9A%E8%AF%8D%E6%9D%A1) \| \| --- \| | \| [上传视频](javascript:;) \| \| --- \| | | . 收藏 [山 89](javascript:void(0);) | | 39 | [女疊](javascript:void(0);)    [口](javascript:void(0);)   \| 权威合作编辑  [好大夫在线](http://www.haodf.com/)   \|  \| \| --- \|   好大夫在线创立于2006年， 中国领先的医疗信息...  [什么是权威编辑](http://baike.bdimg.com/cms/static/cooperation/content.pdf) \| \| --- \| --- \|  \| 词条统计  浏览次数： 226250次  编辑次数： 28次[历史版本](https://baike.baidu.com/historylist/%E5%8A%9F%E8%83%BD%E6%80%A7%E4%BE%BF%E7%A7%98/10429990)  最近更新： [壹统华夏](https://baike.baidu.com/usercenter/userpage?uk=APFSTtvNubDnCpx97W4cKg&from=lemma) ( 2017-07-12)  突出贡献榜  [haodf_hz](https://baike.baidu.com/usercenter/userpage?uk=FJEYwTDtnGuUTco98fnJhA&from=lemma)  [映雪红梅](https://baike.baidu.com/usercenter/userpage?uk=jwrCcN88dHc2oS1Q_wzHig&from=lemma) \| \| --- \|  \| **1** 自己创建个网 **12** 价格便宜的  **2** csgo电脑配置 **13** 自己怎样建  **3** 哈佛大学申请 **14** 螺杆式家用  **4** 战队logo设计 **15** 电脑主机多  **5** vr消防演练 **16** 俄语口语学  **6** 去德国留学 **17** 动物焚烧炉  **7** 无人机反制 **18** 自己建个网  **8** dna亲子鉴定多 **19** MC服务器  **9** 10万级无尘车 **20** GTX显卡排名  **10** 新概念英语网 **21** 机器人展会  **11** 高温隔热材料 **22** 什么叫云计 \| \| --- \| |
| --- | --- | --- | --- | --- | --- | --- | --- | --- | --- | --- | --- | --- | --- | --- | --- |
| \| 郭晓峰 (主任医师) 山西省人民医院消化科 \| \| --- \| | | | | | | |  |  |
| Constipation is a common clinical symptom characterized by dry stools, difficulty defecating, and decreased stool weight and frequency. With the aging of society, the change of modern life rhythm and eating habits, the change of disease spectrum and other effects on diseases, constipation has become one of the important factors affecting the quality of life of modern people, and it is closely related to the incidence of colorectal cancer. Constipation, which can be caused by many causes such as neurogenic, systemic diseases, etc., is called secondary constipation. If constipation does not have organic lesions that cause constipation, it is called functional constipation, which was previously considered simple constipation, habitual constipation or idiopathic constipation. Laxative bowel disease and colon melanosis caused by laxative abuse in patients with constipation have attracted much attention because colon melanosis is associated with colon cancer, so the treatment of functional constipation is receiving more and more attention. | | | | | | |  |  |
| 内科-消化内科  便秘  粪便干结，排便困难  中文名  表 现  所属科室  发病原因  暂不明确 | | | | | | |  |  |
| 相关视频 | 7385播放  04:31 | | | 7002播放  01:23 | | 查看全部 >  >  5653播放 01:04  肠道百科-功能性便秘的诊断 标准是什么？ |  |  |
| 18万播放  05:11 |  |  |  |  |  |  |  |  |
|  | 让专家来为你解释一下，功能 性便秘“罗马标准” \|央视网 | | |  |  |  |  |  |
|  |  |  |  | 什么叫功能性便秘？有以下几 种表现，可不要忽视了 | |  |  |  |
| 发生便秘怎么办？ |  |  |  |  |  |  |  |  |
| \| 目录 \| 1 [疾病介绍](#_bookmark1)  ▪ [药物治疗](#_bookmark6)  ▪ [外科治疗](#_bookmark7)  ▪ [生物反馈](#_bookmark8)  ▪ [高电位治疗](#_bookmark9)  6 [诊断鉴别](#_bookmark10)  ▪ [辅助检查](#_bookmark11)  ▪ [鉴别诊断](#_bookmark12)  7 [疾病治疗](#_bookmark13)  ▪ [饮食治疗](#_bookmark14)  ▪ [养成定时排便习惯](#_bookmark15)  2 [疾病分类](#_bookmark2)  3 [发病原因](#_bookmark3)  4 [发病机制](#_bookmark4)  5 [临床表现](#_bookmark5) \| \| --- \| --- \| | | | | | | |  |  |
| 疾病介绍  [小 播报](javascript:;)    Functional constipation refers to chronic constipation that lacks an organic cause, has no structural abnormalities or metabolic disorders, and excludes irritable bowel syndrome. Patients with functional constipation may present with hard stools, difficulty defecating, incomplete bowel movements, and decreased bowel frequency. Table 1 lists the Rome III diagnostic criteria for functional constipation, which have been symptomatic for at least 6 months prior to diagnosis and have met these criteria for the last 3 months.  Table 1 Rome III functional <a href="#" data-lemmaid="332148" >  Diagnostic criteria for constipation</a>  1. Must meet 2 or more of the following:  (1) At least 25% of bowel movements feel strained  (2) At least 25% of bowel movements are dry bulbous or hard stools  (3) At least 25% of bowel movements have a feeling of anorectal obstruction or obstruction  (4) At least 25% of bowel movements require manual help (such as finger assistance, pelvic floor support)  (5) 3 times per week < stool  2. Loose stools rarely appear when laxatives are not used  3. There is insufficient evidence to diagnose IBS  The main diagnostic points of irritable bowel syndrome with constipation (IBS-C) patients have abdominal pain and/or abdominal distension, which is closely related to defecation frequency and changes in fecal form during the attack. | | | | | | | | |

<https://baike.baidu.com/item/>功能性便秘?fromModule=lemma_search-box

1/7

2022/12/14 10:43 功能性便秘_百度百科

[女疊口](javascript:void(0);)

The concept of constipation proposed in 1999 refers to the small amount of stool, too hard and difficult to drain, and combined with some special symptoms such as prolonged straining, rectal bloating, incomplete bowel movements, and even the need for manual help to defecate, less than 2 bowel movements in 1 week or no intention to defecate for a long time. More quantitative, easier to understand than international standards.

疾病分类

[小 播报](javascript:;)

In order to facilitate the selection of clinical treatment of functional constipation regimens and drugs, most of them are classified according to the kinetic characteristics of the colon, which are divided into slow transit constipation, functional outlet obstructive constipation and mixed constipation. This classification is based on the characteristics of colonic or anorectal motility disorders.

(1) Slow transit constipation: It is the most common type, which refers to the colon motility disorder, so that the contents remain in the colon or the colon through slow constipation, colon manometry shows that colonic motility is reduced, resulting in slow advancement of colonic content and slow emptying. It may also be accompanied by gastrointestinal disorders due to other autonomic abnormalities, such as retarded gastric emptying or small bowel dyskinesia. Patients complain of low frequency of bowel movements, hard stools, and lack of defecation. Delayed passage of the colon with scintigraphy or radiopaque markers establishes the diagnosis. Therefore, some people call it colonic weakness, which is the most common type of functional constipation. Promotility agents are preferred for treatment.

(2) Outlet obstructive constipation: has normal colonic transmission function, due to abnormal function of the anus and rectum (non-organic lesions) such as absent defecation reflex, pelvic floor muscle spasm syndrome or sphincter incoordination during defecation. These include striated muscle dysfunction, abnormal rectal smooth muscle motility, rectal sensory impairment, sphincter incoordination, and pelvic floor spasm syndrome. Patients complain of difficulty defecating, anorectal obstruction, and the need for hand assistance during defecation. It is more common in children, women and the elderly. Biofeedback therapy is an option.

(3) Mixed constipation: with the characteristics of slow transmission of the colon, there are also abnormal function of the anus and rectum, or both are atypical, and the treatment varies from person to person. This type may develop due to slow-transit constipation, and it is also thought that long-term outlet obstruction affects colonic weakness secondary to colon emptying.

发病原因

[小 播报](javascript:;)

The etiology of functional constipation is not well understood and may be multifactorial. Studies have shown that the high incidence of functional constipation in the elderly is associated with food intake, senile gastrointestinal function such as decreased intestinal secretion of digestive juices, weakened bowel tone peristalsis, and low muscle tone involved in bowel movement. Some patients who complain of functional constipation may have significant food factors, such as a low-residue diet. Adding 30g/d of plant fiber to food can significantly increase intestinal peristalsis, called the cellulose-like effect. Mental and psychological factors also predominate, patients with functional constipation are depressed, anxiety is significantly increased, and patients with functional constipation have autonomic dysfunction. Patients with functional constipation may be accompanied by pangastric dysfunction, such as emptying of the gallbladder and stomach and slow movement of the small intestine.

(1) Due to poor eating habits, the mechanical or chemical stimulation contained in food is insufficient (such as cellulose in vegetables) or the amount of photography is too small, especially the lack of food with a large number of residues. The intestinal stimulation is insufficient, and the reflex pupal movement is weakened, resulting in constipation.

(2) After the total peristalsis of the colon, the fecal mass enters the rectum, causing a defecation reflex. However, when the intention to defecate is often ignored, inappropriate defecation occasions and postures, and frequent use of laxatives or colon washing, etc., can cause the sensitivity of the bowel reflex to be weakened. As a result, although a fecal mass enters, it is not enough to cause effective nerve impulses, so the defecation reflex is not produced, resulting in constipation.

(3) Mental depression or excessive excitement, so that conditioned reflexes are impaired, the inhibition of parasympathetic nerves by the high-level small center is strengthened, and the sympathetic effect of the thoracolumbar branch distributed in the intestinal wall is strengthened, resulting in constipation. A significant proportion of patients with functional constipation have a pre-existing psychological disorder.

(4) Bad living habits and lack of sleep. Persistent high mental tension, etc., can also cause abnormal peristalsis or convulsive contraction of the colon, resulting in constipation. (1) Too little or too refined food, lack of fiber residue stimulation of colon movement. (2) Pregnancy: Smooth muscle motility is reduced in the third trimester of pregnancy, which may be caused by the action of progesterone. (3) Changes in the pattern of life. (4) Certain drugs: such as opium, morphine, codeine, anticholinergic and ganglion blockers, sedatives, antidepressants, certain antacids (calcium carbonate, aluminum hydroxide), etc. In addition, regular use of alcohol sausages and laxatives can reduce the sensitivity of the intestines, causing or aggravating constipation.

[小 播报](javascript:;)

发病机制

Constipation can be seen as the ultimate symptomatic manifestation of different pathophysiologic processes. The defecation process requires peripheral nerve excitation, which transmits impulses to the primary defecation center and cerebral cortex, causing coordinated movements of the colon, rectum, and sphincters and pelvic floor muscles. Obstruction at any one of these steps can lead to constipation.

1 Colon Peristals are the most important in the form of colonic movement. It is composed of some contraction waves that are stable forward. There is also a type of peristalsis that goes on very quickly and advances very far, that is, group peristalsis. Group peristalsis is common postprandial due to the duodeno-colon reflex. The movement of intestinal contents is determined by the postprandial pressure gradient of various parts of the colon, and group peristalsis is necessary to maintain normal bowel function.

2 Rectal canal When defecating normally, when feces enter the rectum, the intention to defecate occurs, the internal sphincter relaxes, and the external sphincter ring that surrounds it forms a dilating effect, rectal contraction makes the pressure in the rectal cavity exceed the pressure of the canal At the same time, the defecation reflex occurs, and the internal sphincter relaxes so that the feces are discharged. Dysfecation caused by intraanal pressure exceeding rectal pressure is a common motility disorder for outlet obstructive constipation. Fecal angiography in patients with pelvic floor spasm syndrome shows that the anorectal angle is reduced, does not increase when defecation is forced, and the depth of the anterior process of the pelvic floor rectum is related to the time of rectal emptying. Puborectal muscle spasm syndrome electromyography presents with paradoxical puborectal muscle contraction. Another important pathophysiology is pelvic floor dysfunction, characterized by a normal or slight slowing of the colon, but prolonged retention of fecal residues in the rectum, the main defect of which is the inability to expel its contents from the rectum. There are many other names for this functional deficit (outlet obstruction, stool difficulty, laxity, pelvic floor dyssynergy). This inferential pathophysiology that leads to the inability to excrete stool from the rectum is poorly understood. The simplest possible classifications are: (1) high muscle tension (relaxation is not); Incomplete loosening of the pelvic floor and constriction of the pelvic floor and external sphincter when attempting to defecate. (2) Muscle hypotonia, sometimes accompanied by excessive lowering of the megarectum and pelvic floor. These syndromes are multifactorial and some are not well understood.

3. Pathological changes of the muscular layer and intermuscular plexus of the intestinal wall Many research data show that the colon wall of patients with constipation has pathological changes such as muscle fibrosis, muscle atrophy, degeneration of the intermuscular plexus of the intestinal wall, deformation, and reduced number.

4. Changes in neurotransmitters in the intestinal wall

临床表现

[小 播报](javascript:;)

<https://baike.baidu.com/item/>功能性便秘?fromModule=lemma_search-box

2/7

| 2022/12/14 10:43  功能性便秘_百度百科  [女疊 口](javascript:void(0);)    Due to excessive stagnation of the fecal mass in the sigmoid colon and rectum, patients sometimes have a feeling of distension in the left lower abdomen, and often have symptoms such as poor desire and bowel movements after tenesmus. Hemorrhoids often appear as a secondary symptom of constipation. In patients who are accustomed to using laxatives or bowel washing, due to the disturbance of gastrointestinal motor function, epigastric fullness and discomfort, belching, nausea, abdominal pain, abdominal sound, exhaust and other complaints may occur. Long-term constipation may cause mild "toxemia" symptoms in some patients, such as loss of appetite, bitter mouth, malaise, dizziness, fatigue, and body aches. As for those who cause mild anemia and malnutrition, it is rare. In a few cases, there is dull pain and swelling sensation in the buttocks and back of the thighs, which is caused by the compression of the anterior branches of the third, fourth and fifth spinal nerve roots by the fecal mass. For neurasthenic symptoms such as headache, tiredness, insomnia, etc., it is not so much a consequence of functional constipation as a cause of it.  Fecal traits are often a characteristic complaint of patients. The feces excreted by people with rectal constipation are mostly coarse and lumpy, while colonic constipation is mostly small, similar to sheep feces. The mechanical irritation of hard stool causes the rectal mucosa to secrete mucus, often covering the surface and between the gaps of hard stool, and sometimes discharged as mucus membrane. People with constipation sometimes have sudden abdominal pain during bowel movements. The initiation of hard stools, followed by foul-smelling loose stools, is called "pseudodiarrhea".  Most patients have inconspicuous signs. In spastic constipation, the convulsive constriction of the bowel is often seen. In rectal constipation, a fecal mass can often be palpated in the lower left gum, a solid fecal mass can be touched during digital examination, and after defecation, digital examination shows that the intestinal wall is not easy to touch due to ampulla dilation.  In the Roman Standard Definition, bowel movement frequency is only one of the six basic characteristics, including force, hard stools, and incomplete bowel movements. The symptoms included in the patient definition are (second in importance  Order): Force, excessively hard stools, urgent and ineffective stools, low frequency of stools, and incomplete bowel movements. Symptom assessment should include a history of specific symptoms. Complete prescription and over-the-counter medications must be listed. Constipation side effects are widespread among commonly used medications. Most people with constipation often apply their own over-the-counter medications to relieve their symptoms. Population studies have shown that laxative use and abuse are 7% and 4%, respectively.  [小 播报](javascript:;)  诊断鉴别  Ancillary examination  (1) Gastrointestinal X-ray examination to understand the motor function status of barium according to its operation in the gastrointestinal tract. In patients with slowed tone constipation, barium may be seen to travel significantly after reaching the colon, with prolonged arrest in the left colon, particularly showing a dilated rectal ampulla. In patients with constipation, barium in the colon can be divided into small pieces, and it can be seen that as a result of reverse peristalsis, barium that reaches the descending colon or sigmoid colon can sometimes retrograde to the transverse colon. The greater significance of gastrointestinal x-ray examination is to exclude constipation caused by organic lesions such as tumors, tuberculosis, megacolonia, and obstruction, which is very important to establish the diagnosis of functional constipation.  (2) Proctoscopy, sigmoidoscopy and fiberoptic colonoscopy can directly diagnose the state of the intestinal mucosa, and biopsy is taken if necessary. In patients with functional constipation, the colonic mucosa, especially the rectal mucosa, often has varying degrees of inflammatory changes due to the retention and irritation of hard feces. It is manifested as hyperemia, edema, vascular ambiguity, etc. In patients with contracture constipation, spastic contractions of the bowel may sometimes be seen on colonoscopy, in addition to inflammatory changes. Manifested by the intestinal wall converging into the lumen, the intestinal lumen contraction and narrowing, difficulty in advancing the colonoscopy, and the patient feels abdominal pain. It is relieved by a short pause of contracture, the intestinal lumen is opened, and the abdominal pain disappears.  (3) Fecal angiography is a method that combines morphology and dynamics to evaluate the function of the anorectal area. X-ray imaging technology was used to measure the anorectal angle, suprarectal distance, and B shame distance of each phase of sitting, lifting, forbearance, and forceful defecation. It is used to diagnose anatomic malformations (rectal prolapse, rectal protrusion, etc.) and local dysfunction of the distal intestinal part (functional outlet obstruction, rectal weakness, etc.), which is of great value in constipation and can provide a basis for the selection of treatment.  (4) Anorectal manometry is also very helpful for the etiology and treatment of functional constipation. and rectal manometry plays an important role in the diagnosis of chronic constipation and distinguishes end-stage constipation from other types of constipation. Commonly used parameters are internal sphincter pressure and length, maximum compression pressure, rectal sensitivity, and rectal anus  Door reflections, etc.  (5) Anorectal sensory examination Measuring sensation by electrical stimulation. The energized probe is in contact with the mucosa, and the upper, middle and lower sphincter is measured separately, and the amount of current is gradually increased until the patient has a burning or tingling sensation, the threshold is recorded, and the average threshold is calculated. The normal value is 2. 0—7． 3mA。 Rectal sensitivity is measured by balloon dilation. Kamm et al. reported that the current stimulation method is more precise, avoiding differences in balloon pressure, rectal diameter, and compliance. The results measured by the two methods correlated significantly. However, the latter is acceptable and reproducible.  (vi) sphincter electromyography A needle or column electrode is inserted into the subcutaneous bundle of the external sphincter to record the electrical activity. The most common EMG change in patients with constipation is paradoxical contraction of the puborectal muscle. EMG can distinguish between pelvic floor voluntary muscle group muscles and neurological dysfunction, and 77% of patients have pelvic floor muscles that cannot relax during bowel movements, which is of great significance for the diagnosis of outlet obstructive constipation.  鉴别诊断  The diagnosis of functional constipation depends on the medical history, analysis of the cause of constipation, and digital examination can make a diagnosis of constipation. If necessary, barium x-rays of the gastrointestinal tract or / and colonoscopy may be performed to exclude organic diseases and confirm the diagnosis of functional constipation.  First of all, it is necessary to clarify the true meaning of the patient's constipation, many people mistakenly believe that only 1 bowel movement per day is normal, and some people mistakenly think that incomplete bowel movements due to internal hemorrhoidal prolapse, causing foreign body sensation. The physical properties of the stool should be clarified when asked whether the stool is dry and hard, because some patients actually only have slightly dry formed stools when they answer "dry stools". There are also some patients with chronic constipation, who often take laxatives to defecate, and if they do not ask in detail, they can mistakenly think that the stool is normal. Therefore, only natural bowel movements (non-laxative bowel movements) less than 3 times a week, or dry and hard stools, or stools that are not dry and hard and difficult to excrete, accompanied by discomfort, can be considered constipation.  The time of onset has a certain significance for diagnosis, and the onset of disease in juveniles suggests that the cause is related to congenital factors, while the recent onset is mostly caused by intestinal organic lesions or dietary environmental factors.  Patients with painful bowel movements indicate lesions near the canal, while those with painless bowel movements accompanied by blood and mucus are mostly intraluminal lesions in the node and rectum.  Poor eating habits such as low intake, low water drinking, partial eating, not liking vegetables and bad bowel habits such as often ignoring the desire to defecate can often directly prompt the preliminary diagnosis, such as shop clerks, textile factory female workers, due to the intention to drink less water or even no water at work and cause chronic constipation. Escalating laxative abuse is another major cause of intractable constipation that is difficult to correct, and it is important to ask in detail about the type of medication, how to use it, the time of start and end, and the effect of medication. Long-term use of a drug that can cause constipation due to other diseases is a cause that is often missed.  A history of abdominal and perineal surgery should be documented and the relationship with constipation should be inquired. Some of the more specific manifestations such as prolonged bowel movements, repeated excessive straining, rectal fullness, incomplete defecation, and hand-assisted defecation (i.e., fingers inserted into the anus or vagina to assist with defecation) often indicate pelvic floor outlet lesions. The physical properties of the stool can sometimes help determine the location of the lesion, and long-term chestnut-like dry and hard stools suggest that constipation may be colonic, while soft stools are difficult to pass and the fecal mass is thinner, suggesting that the cause of constipation may be rectal and pelvic floor.  Since constipation is not an independent disease, but a group of symptoms caused by multiple causes, the diagnosis of constipation should focus on the diagnosis of the cause, rather than the diagnosis of symptoms, such as "slow constipation", "habitual constipation" and so on. Symptomatic diagnosis alone is incomplete or even dangerous, and there is a possibility of misdiagnosis and missed diagnosis of major lesions. Recipients should routinely conduct a comprehensive and systematic examination of the patient, especially when the characteristic manifestations of the primary disease causing constipation are not obvious, but the first manifestation of constipation symptoms. The author has seen several cases of patients diagnosed with "chronic constipation", who did not undergo routine examination, and occurred blood in the stool and intestinal obstruction while waiting for special examination, and were easily diagnosed as rectal cancer and colon cancer after routine examination. In the past, there were also patients with constipation who did not respond well to surgery, and were finally diagnosed with diabetes and systemic sclerosis.  Therefore, the term constipation should not become an independent diagnosis, under which possible causes should be listed. For patients with a primary disease that is difficult to identify for a while, known major organic lesions must be ruled out first. Consider functional tests such as intestinal transit, anorectal dynamology, fecal angiography, pelvic floor electromyography, etc. only if known organic lesions are not found after a thorough systematic examination.  Colonic transport function test: It is a method of using radiopaque X-ray markers to take regular abdominal radiographs after oral administration to track the operation of the colon and track the operation of the colon.  Anorectal kinetic examination: The use of pressure measurement device to check the functional status of the internal and external sphincters, pelvic floor, rectum and the coordination between them is of great significance to determine whether constipation is related to the dysfunction of the above structures.  Pelvic floor electromyography: electrophysiological techniques are used to examine the functional status of the striated muscles such as the pelvic floor muscles, puborectal muscles, and external sphincters, as well as the functional status of their innervated nerves. Due to the high requirements of this technique for examiners and the difficulty of judging the examination results, it is currently only used to observe whether there is abnormal discharge of the pelvic floor striated muscle during simulated bowel movements. Those who use needle electrodes, because of traumatic examination, are easy to induce protective reflexes and cause false positives, especially when using multiple needle electrodes at the same time, inexperienced people often make mistakes in judgment, which should attract attention.  Fecal angiography: After barium is injected into the rectum, colon (and sometimes by mouth to observe the small intestine), the patient sits on a toilet that is easy to seep X-rays, and during the process of defecation, multiple films or videos are taken to observe imaging changes in the canal and rectum.  The examiner should read the film in person, combined with clinical data and other examination results, and cannot be diagnosed by imaging data alone. Histology: biopsy should be performed when Hirschsprung disease is suspected. In the past, it was often taken 2 ~ 3cm above the tooth line, but some people think that it is better to take the material 1 ~ 1 5cm above the tooth line, because the material taken from the high part may miss the "ultra-short section of megacolon".  Generally, for patients with symptoms of constipation, if he complains of constipation, three levels of problems should be solved in diagnosis. The first level is symptom diagnosis. That is, whether the patient's complaint meets the definition of constipation, that is, natural stool frequency reduction or difficulty in discharge, accompanied by uncomfortable symptoms, only if the definition is met can it be determined that he has constipation symptoms. The second level is functional diagnostics. That is, through the intestinal transport function test, it is divided into normal intestinal transport type (total intestinal passage time ≤3 days) or intestinal slow transit type (total intestinal passage time &gt;3 days). The normal transport type is mainly manifested as outlet constipation, and abnormalities of the internal sphincter, external sphincter (including puborectal muscle and levator muscle), rectum, internal genital organs, and urinary organs can be found through anorectal dynamics, pelvic floor electrophysiology, and fecal angiography. The third level is the diagnosis of etiology. That is, gradually exclude according to the etiological classification table and determine the most important cause.  Special examination and reference values for constipation: (1) Colon transit test: subjects should refrain from taking laxatives and other drugs that affect intestinal function from 3 days before the examination. The daily examination consists of 20 marker capsules, 2 capsules, 1 plain abdominal film every 24 hours. Normal patients should excrete 80% of the marker within 72 hours. (2) Fecal angiography: normal: the rectangular force discharge is increased compared with resting, and it should be ≥ 90 degrees, and the anus is the smallest. The supraanal distance should be ≥ resting, but the supraanal distance must be 30 mm ≤ (35 mm &lt; for multiparous women). Both the B shame distance and the small shame distance are negative. Sacral straight spacing ≤ about 10mm, or 20mm and uniform. Barium drainage was smooth and no abnormalities were found. (3) pressure measurement: left lying position, do not do digital examination before manometry. First, the balloon or probe is placed in the canal, the resting pressure and maximum constriction pressure of the canal are measured, and then the balloon is sent into the ampulla of the rectum to measure the rectal resting pressure, and the catheter is connected to the drag device to measure the length of sphincter function. Change the double capsule catheter, the large sac is placed in the ampulla of the rectum, the small sac or probe is placed in the canal, and the large sac is rapidly inflated 50 ~ 100ml. Normal is a decrease in pressure and a duration greater than 30 seconds, and a positive anorectal suppression reflex. (4) Rectal sensory function and compliance determination: the maximum tolerated capacity minus rectal sensory threshold is volume change (v), the maximum tolerated capacity pressure minus rectal sensory threshold pressure is pressure change (P), V/P is rectal compliance. (5) Balloon forcing test: Place the balloon in the ampulla of the rectum, inject 50ml of warm water, and instruct the subject to take a habitual defecation position and discharge the balloon as soon as possible. Normal discharge within 5 minutes. (6) Pelvic floor electromyography examination: needle electrodes are punctured to the puborectal muscle and the deep or superficial part of the external sphincter muscle, and the myoelectric activity of the subject at rest, mild contraction, forced contraction and defecation is recorded. Analyze changes in waveform, amplitude, and frequency.  In middle-aged and older patients, bowel habits – regular, gradual onset of intractable constipation must be given timely and thorough examinations to rule out colon cancer. When intractable constipation begins at a young age, the possibility of an overly long colon and Hirschsprung disease should be considered.  Constipation as one of the symptoms can be seen in various diseases caused by the lack of bowel motility. For example, cachexia, weakness, pregnancy, ascites, huge compression, chronic, and scleroplegia caused by long-term chronic wasting diseases can often cause weakness of abdominal muscles, box muscles, levator muscles and smooth muscles, which may cause constipation. Spinal cord and cauda equina injuries often cause impaired defecation reflexes. fissures, hemorrhoids, perianal inflammation, etc. can cause spasm of the sphincter and transient stenosis of the anus, etc., which can cause constipation. As for lead, arsenic, mercury, phosphorus and other poisoning, the use of calcium carbonate, aluminum hydroxide, atropine, opium and other drugs, intestinal amine stenosis caused by various reasons, etc., although constipation can occur, it often cannot cover up the main manifestations of the primary disease, so it is often not difficult to distinguish from functional constipation. |
| --- |

<https://baike.baidu.com/item/>功能性便秘?fromModule=lemma_search-box

3/7

2022/12/14 10:43 功能性便秘_百度百科

[女](javascript:void(0);) [疊口](http://baike.baidu.com/l/WWoXYu7P)

[小 播报](javascript:;)

疾病治疗

The treatment of functional constipation should take comprehensive measures and holistic treatment to improve or restore normal defecation and achieve the purpose of relieving various symptoms. At the same time, we should also consider whether the therapeutic drug can be used for a long time, how safe it is and whether the patient can be expected to have good tolerance to the drug.

The fundamental treatment is to remove the cause. For patients with functional constipation, reasonable diet and living habits should be established. Correct bad habits, adjust dietary content, increase vegetables and fruits rich in fiber and vitamin, appropriate intake of coarse and slag grains, such as standard flour, potato, corn, barley rice, etc. Oil food, cold water, honey are helpful for the prevention and treatment of constipation.

Reasonable arrange work and life, do take rests. Proper sports and sports activities, especially the exercise of abdominal muscles, are beneficial to the improvement of gastrointestinal function. For long-term mental work, sedentary office less activity is more beneficial.

Develop good bowel movement. Establish the habit of defecation on time every day, so that the defecation movement of the rectum produces conditioning. "In those with neurasthenia, placebo may be administered appropriately to modulate autonomic central function." Patients with anal fissure, perianal infection and adnexitis should be treated in time to eliminate their reflex effect on bowel movement and constipation.

<https://baike.baidu.com/item/>功能性便秘?fromModule=lemma_search-box

4/7

2022/12/14 10:43 功能性便秘_百度百科

[女](http://baike.baidu.com/l/WWoXYu7P)  [疊口](javascript:void(0);)

The etiology and pathogenesis of constipation should be treated, and the principles of conservative treatment for patients with constipation without organic lesions are: (1) increase the intake of dietary fiber; (2) develop regular bowel habits; (3) avoid the use of laxatives; (4) individualize treatment.

饮食治疗

Dietary dietary fiber can change the nature of feces and bowel habits, and the fiber itself is not absorbed, which can swell the feces and stimulate colonic motility. This may be more effective for people with constipation who consume less dietary fiber. Patients with intestinal obstruction or megacolon and neurological constipation can not use increasing dietary fiber to achieve the purpose of laxation, should reduce intestinal content, and regular bowel movements.

The diet should choose vegetables and fruits rich in crude fiber and foods rich in B vitamins, such as whole grains and beans. Sesame seeds, honey, pine nuts, almonds, mountain mushrooms, walnuts, bamboo shoots, potatoes, radish, bananas, silver fungus, peanuts, corn, spinach, knapkins, celery, wheat bran, buckwheat, sunflower seeds, vegetable oil, figs, water chestnuts and other foods and mulberry seeds, cassia seeds, raw shou wu, angelica, hemp seeds, plum kernels, cistanche and other medicinal and food products. Avoid alcohol, tobacco, strong tea, coffee, garlic, peppers and other irritating foods. In terms of food treatment, the following dietary remedies can be selected:

1 500g of black sesame seeds, 250g of walnuts and 100g of cottony sugar. First remove impurities from black sesame seeds and walnut kernels, dry them, stir-fry them, grind them into fine powder, mix them well, put them in bottles and jars, and set aside. 2 times/d, 15d/time, or chew 15g in the morning and evening. This dietary remedy is suitable for all types of functional constipation.

2 Cypress kernel stewed pork heart 20g cypress kernel, 1 pork heart (about 500g). Soak the pork hearts in clean water for a while, wash them and cut them into thin slices. Wash the cypress kernels and place in a bowl. Add an appropriate amount of water to a casserole, put it on the fire, add pork heart slices, boil over high heat, cook cooking wine, add chopped green onion, ginger slices and cypress kernels, change to low heat and simmer for 1h, wait for the pig heart to rot, stop the fire, add refined salt, monosodium glutamate, five-spice powder a little each, mix evenly. Serve as a dish. This dietary remedy is suitable for blood deficiency and constipation.

3 Sanren porridge cypress kernel 20g, pine nut kernel 15g, Yu Li ren 20g, glutinous rice 100g. First break the plum kernels, put them in a pot, add water and cook for 20min, remove the residue and take the juice. Crush the cypress kernels and pine nuts, remove the coat, put them into a casserole pot with the washed glutinous rice, add an appropriate amount of water, first boil over high heat, slowly add the plum kernel frying sauce, and simmer over low heat to make a thick porridge. Divide 2 times in the morning and evening. This dietary remedy is suitable for all types of functional constipation.

4 Astragalus hemp seed honey drink honey 20g of astragalus, 10g of hemp seed, 15g of honey. First crush the raw hemp seeds, put them into a pot with honey and seared astragalus, add water and cook for 30min, remove the slag, take the thick juice, add honey while warm, and mix well. Take every morning on an empty stomach. This dietary remedy is especially suitable for qi-deficient constipation.

5 Senna cassia tea senna 3g, cassia 30g. Put senna leaves and cassia seeds in a covered cup, brew with boiling water, cover, and simmer for 15min before drinking. When tea, drink frequently, generally brewed 2 times. This dietary remedy is particularly suitable for thermostatic constipation. [1]

养成定时排便习惯

Regular bowel movements prevent stool buildup, which is especially important in patients with fecal impaction. Note that before training, it is advisable to cleanse the intestines, which can be cleaned with normal saline, 2 times a day, for 3 days. After colon cleansing, plain abdominal radiographs were taken to confirm that there was no fecal impaction in the intestine. In recent years, oral electrolyte balance solution has also been reported, which can achieve the purpose of colon cleansing. After colon cleansing, laxatives can be given, and the number of poops should reach at least 1 time/day. Patients are encouraged to relieve their bowel movements after breakfast or, if they still do not defecate, to relieve themselves again after dinner. Restore normal bowel habits. Once postprandial defecation occurs regularly and lasts for more than 2 ~ 3 months, laxatives can be gradually discontinued. If there is 2 ~ 3 days of stool relief during the process, it is still necessary to cleanse the intestine to avoid fecal impaction again. This method of cleansing the intestines, taking laxatives and training bowel habits is often used to treat functional constipation, and its success rate can reach 70 ~ 80%, but there are many recurrences. For patients with constipation with rectal sphincter dysfunction, Biofeedback can be used to correct inappropriate contractions of the pelvic floor muscles and extraanal dilators during defecation, which has been successful in children and adults with functional constipation, but is less effective in patients with constipation who are depressed.

药物治疗

Drug therapy can be considered when the above methods fail to achieve curative effect. For STC patients, prokinetic agents are the first choice. As a prokinetic agent for the whole gastrointestinal tract, cisapride is effective for some STC patients. A new type of specific intestinal motility drug, prucalopride, has recently come out. This drug is a benzofuran compound that specifically acts on 5-HT4 receptors. It is expected to become an ideal drug for the treatment of functional constipation. Commonly used laxatives are: ① volumetric laxatives: magnesium sulfate, sodium sulfate, methylcellulose, agar, etc.; ② stimulant laxatives: senna, castor oil, diesterphenidin, etc.; ③ stool softener: liquid Paraffin, lactulose, etc.; ④ Intrarectal administration: glycerin suppository, Kaisailu, etc. Long-term abuse of laxatives should be avoided leading to laxative enteropathy.

1. Bulk laxatives (cellulose) can accelerate the transit of the colon or the whole intestinal tract, absorb water, make the stool soft and easy to pass, and relieve constipation and the urgency of defecation; soluble fibers such as pectin, plantain, and oat bran help maintain Moisture in feces; and insoluble cellulose such as plant cellulose and lignin can increase the amount of stool.

The advantages of cellulose preparations are that they are economical, safe, and applicable to medical institutions at all levels; however, flatulence may occur when a large amount of cellulose preparations are ingested, and should be used with caution in patients with colonic fatigue.

After supplementing cellulose, it will not be effective immediately. After 7-10 days of application, the dosage should be appropriately increased or decreased according to the specific situation.

2. Salt laxatives (magnesium sulfate) Oral magnesium sulfate is not easy to absorb in the intestines, and stays in the intestinal cavity to form a hypertonic state. The catharsis effect is strong and rapid. Generally, watery or semi-fluid stools can be discharged after 2-6 hours of oral administration . It can cause serious adverse reactions and should be used with caution in clinical practice. At present, it is usually used for bowel preparation before examinations such as colonoscopy or barium enema.

3. Stimulant diarrhea (fanna, cascara, phenolphthalein, castor oil, etc.) Long-term use of stimulant laxatives can damage the patient's enteric nervous system, and it is likely to be irreversible.

Phenolphthalein: After oral administration, it forms soluble sodium salts in the intestines, stimulates the colonic mucosa to promote peristalsis; and prevents intestinal juice from being absorbed by the intestinal wall, thereby causing catharsis. Generally, semi-fluid soft stools can be discharged 4-8 hours after medication, and catharsis is related to the acidity of the liquid in the intestinal cavity. It is contraindicated for appendicitis, intestinal bleeding, heart and kidney insufficiency, hypertension, intestinal obstruction, infants and pregnant women. Clinical application 1-4 tablets each time, take before going to bed. Those who prepare the bowel before colonoscopy, X-ray examination or surgery should take it 8 hours in advance.

Bisacodyl: After oral administration, the products decomposed by intestinal bacteria and the drug itself have a strong stimulating effect on the wall, which can increase intestinal peristalsis and promote defecation; at the same time, it can inhibit the absorption of NA, CA2 and water in the colon, so that The volume of the intestinal lumen increases, causing reflex defecation. Clinically, it is more effective for acute and chronic constipation. It can also be used for intestinal emptying before childbirth, before surgery, abdominal X-ray examination or endoscopy, and to restore normal bowel habits after surgery and postpartum. It can cause abdominal pain and occasionally severe abdominal cramps after taking it. It is contraindicated for patients with acute abdomen, spastic constipation, severe hard stools, anal rupture or hemorrhoid ulcers, and should be used with caution by pregnant women.

<https://baike.baidu.com/item/>功能性便秘?fromModule=lemma_search-box

5/7

2022/12/14 10:43 功能性便秘_百度百科

[女疊口](javascript:void(0);)

**4**、渗透性泻剂(聚乙二醇4000)、乳果糖等

乳果糖：是人工合成双糖，在胃及小肠内不被分解和吸收，到达结肠后，通过渗透作用使水和电解质保留于肠腔内；并被肠 道正常菌群分解为乳酸和乙酸等，并进一步提高唱腔内渗透压，产生导泻作用；阻断氨的吸收；其酸性代谢产物能刺激肠黏膜， 增加肠蠕动，促进排便。由于乳果糖在体内分解产生气体，故部分患者会有腹胀、排气增多等胃肠胀气表现。用量过大会产生恶 心、腹胀、 [腹泻](https://baike.baidu.com/item/%E8%85%B9%E6%B3%BB/2193261?fromModule=lemma_inlink)和[低钾血症](https://baike.baidu.com/item/%E4%BD%8E%E9%92%BE%E8%A1%80%E7%97%87/5085460?fromModule=lemma_inlink)、[高钠血症](https://baike.baidu.com/item/%E9%AB%98%E9%92%A0%E8%A1%80%E7%97%87/2379282?fromModule=lemma_inlink)等。禁用于胃肠道阻塞、 [糖尿病](https://baike.baidu.com/item/%E7%B3%96%E5%B0%BF%E7%97%85/100969?fromModule=lemma_inlink)或低糖饮食者。慢性便秘患者治疗剂量为每天1-2次，每 次5-10g，及俩美好以每日保持2-3次软便为宜。临床用于慢性功能性便秘，包括老人、儿童、婴儿和孕妇各个年龄组的患者，安 全性高。对于[肝性脑病](https://baike.baidu.com/item/%E8%82%9D%E6%80%A7%E8%84%91%E7%97%85/2827515?fromModule=lemma_inlink)患者，应用乳果糖后，不仅具有保持大便通畅的作用，还可减少氨的吸收，有利于[肝性脑病](https://baike.baidu.com/item/%E8%82%9D%E6%80%A7%E8%84%91%E7%97%85/2827515?fromModule=lemma_inlink)的恢复。

**5.**促动力药(西沙必利)是临床上广泛应用的胃肠道促动力药，属于苯二氮卓类药物，其促动力效应直接作用于上段结肠。 它曾用于便秘的治疗，但疗效并不肯定。对于结肠乏力即STC患者，选用促动剂改善肠神经和特异选择性作用于结肠平滑肌的促 动力药，如5— HT4受体激动剂，西沙必利，普卡必利(procaloprid)，以及5— HT4部分激动剂，特异作用结肠的替加色罗 (tegaserod)等，后者多用于CIBS。此外，米索前列醇(misoprost01)，阿片类拮抗剂纳络酮(naloxone)也可改善某些患者的便秘症 状，但对功能和梗阻型便秘的排便功能，尚未能证实其确切疗效。

6.润滑性泻剂(开塞露 液状石蜡)

开塞露(含硫酸镁、甘油、丙二醇)：能润滑并刺激肠壁，软化大便，使其易于排出，成人20ml/次，主要适用于硬结便患 者，尤其是老年症患者。

液状石蜡：在肠道内不被吸收或消化，润滑肠壁，使粪便易于排出。对年老体弱、长期卧床的便秘患者使用是应注意其有引 起脂质性吸入性[肺炎](https://baike.baidu.com/item/%E8%82%BA%E7%82%8E/1083485?fromModule=lemma_inlink)的可能，长期服用可致脂溶性维生素缺乏。成人15-30ml/次，用药后6-8小时产生效果，一般于睡前服用。

**7.**微生态制剂:含有双歧杆菌、乳酸杆菌、肠球菌等肠道正常菌群。是一种良好的微生态调节制剂，直接补充正常生理性菌 群，改善肠道微生态环境。但应避免与抗生素合用。

调节肠道微生态的制剂还有米雅BM、丽珠肠乐等。可作为便秘的辅助治疗。

**8.**中药泻剂 就中医而言，便秘分为实秘、虚秘。热秘以清热润肠为主，可服麻仁丸；气秘应理气导滞，以苏子降气汤加味。 虚秘又分[气虚](https://baike.baidu.com/item/%E6%B0%94%E8%99%9A/4363275?fromModule=lemma_inlink)，以益气润肠为主，用补中益气汤加减；血虚则宜养血润燥，四物汤可用；寒凝则应温通开秘，以温脾汤加味。

临床上常用的中药制剂应注意，制剂中大都含有大黄、芦荟等刺激性泻剂成分的药物，故不主张长时间的应用。

简言之，在慢性便秘治疗中，选用不恰当的泻剂或泻剂应用剂量不合理等，均可能引起患者脱水、电解质平衡紊乱等到不良 反应。对有[高血压](https://baike.baidu.com/item/%E9%AB%98%E8%A1%80%E5%8E%8B/195863?fromModule=lemma_inlink)、[心脏病](https://baike.baidu.com/item/%E5%BF%83%E8%84%8F%E7%97%85/1441350?fromModule=lemma_inlink)、[糖尿病](https://baike.baidu.com/item/%E7%B3%96%E5%B0%BF%E7%97%85/100969?fromModule=lemma_inlink)、肾功能不全合并便秘的患者，应选用安全的通便药物，如聚乙二醇4000。

外科治疗

当应用轻泻药、纤维和促动力药进行的积极的、延长疗程的结肠惰性治疗失败时，其治疗应是全结肠切除伴回-直肠吻合术。 应告诉患者，该手术是设计用来治疗便秘症状(排便困难或频率稀少)。其他症状(腹痛和腹胀)可能不会缓解。结肠切除到骶骨岬水 平，在末端回肠和直肠上端之间进行吻合。进入骶前区时需仔细保留交感神经。

回-直肠吻合较回肠-乙状结肠吻合更为成功。如果任何部位留下乙状结肠，便秘可能复发，相反，吻合口低于距肛门边缘7 ~ 10厘米水平可能导致无法接受的高排便频率，有时甚至[大便失禁](https://baike.baidu.com/item/%E5%A4%A7%E4%BE%BF%E5%A4%B1%E7%A6%81/6048753?fromModule=lemma_inlink)。回-直肠吻合术后仍持续便秘的患者可能有盆腔底功能异常。

**1**、排空异常的外科治疗

切断耻骨直肠肌的后纤维被认为可能对排便时该肌肉呈矛盾收缩的患者有益。然而并非如此，不论是切断耻骨直肠肌的后部 或侧面都令人失望。将耻骨直肠肌肉纤维在中线任何一边切断， 7名患者中仅1人症状改善，而将侧面肌肉切断在15名患者中仅3 人症状改善。

**2**、会阴下降综合征

会阴下降综合征患者也会发生便秘，这种患者排便时无止境地摒力但直肠不能完全排空。可以观察到会阴明显鼓出坐骨结节 平面，这种会阴异常下降可能继发于分娩，或是排便时长时间摒力造成骶神经损伤。不完全排空导致更摒力，对神经的牵拉更 强，以及肛门外括约肌和耻骨直肠肌的进行性去神经支配。这种情况会造成[大便失禁](https://baike.baidu.com/item/%E5%A4%A7%E4%BE%BF%E5%A4%B1%E7%A6%81/6048753?fromModule=lemma_inlink)，因而增加患者的痛苦。手术不能纠正该问 题。最佳的治疗方法是生物反馈，尽管成功率只有50%。

**3**、造口术

患者有时因便秘而要求作造口。造口是个好的选择，因其能回复。再次，仔细选择患者极为重要。结肠造口容许作结肠冲洗 的可能性，但一些作者报道因造口近端的持续结肠惰性或更全面的动力紊乱，导致效果不满意。

最近描述的一种称为"自制结肠导管"的手术可能是对某些患者的解决方法。通过在中点横断乙状结肠，将之用作为自制结肠 导管。该手术成功地降低患者的排便时间，增加排便次数。该手术是可逆的，但复杂。

因此，在许多诉有便秘的患者中只有一小部分将从手术中得益，可能是占经高度选择的转诊病人的5%。

生物反馈

生物反馈治疗的实质是利用声音和影像的反馈，刺激训练病人正确地控制肛门外括约肌的舒缩，达到正常排便。生物反馈治 疗法是一种纠正不协调排便行为的训练法，主要用于治疗肛门括约肌失协调和盆底肌、肛门外括约肌排便时矛盾性收缩导致的 FOOC，有人报告其疗效可达96%，该法与药物治疗相比具有无药物副作用、成本低、非创伤性等优点，目前国内已开展此项疗 法。生物反馈疗法对功能性便秘有确定的疗效，无副作用，治疗费用低。 Faliakou等报道对100例功能性便秘病人65%为结肠慢

<https://baike.baidu.com/item/>功能性便秘?fromModule=lemma_search-box

6/7

2022/12/14 10:43

功能性便秘_百度百科

| 传输， 59%为反常性盆底肌痉挛)历时4年的研究结果显示，生物反馈疗法对慢传输型、出口梗阻型、混合型便秘病人均有效。  Glia等对26例功能性便秘病人10例为结肠慢传输， 16 例为反常性盆底肌痉挛进行生物反馈治疗， 6个月的随访结果表明，生物反  馈疗法对出口梗阻型便秘病人有较好疗效。  **1**、生物反馈疗法的具体步骤  生物反馈疗法强调动员病人大脑的调控功能，强调医生与病人之间良好的沟通，这一思想贯穿生物反馈疗法的各个步骤。  首先，在治疗前，要向病人详细讲解人体结肠、直肠、肛门和盆底肌的正常解剖和生理功能，讲解正常排便的机制；还要向  病人讲解清楚生物反馈治疗的机理和目的以及生物反馈仪器的使用。将治疗仪与病人连接好后，安排病人坐或躺在治疗仪和治疗  师的右侧，面对治疗仪和治疗师。向病人讲解清楚仪器上所显示的曲线的意义，并指出病人在静息、屏气和用力排便时的异常所  在。耐心告诉病人如何调控括约肌的舒缩，鼓励其尝试，病人的每一次尝试都会在仪器上显示，一旦有正确的活动，仪器便会以  悦耳的声音和动感的图象刺激病人，治疗师亦会给予鼓励。最后，病人在无治疗师帮助的情况下，面对仪器自行练习，直至连续  三次正常排便出现为止。  **2**、生物反馈疗法的时间安排  行生物反馈治疗者绝大多数为门诊病人，一般安排病人每周治疗2次，持续5周以上。  **3**、生物反馈疗法的几种形式  ( **1**) 肌电图介导的生物反馈方式(EMG－basedbiofeedbackmethod)是目前最为常用的生物反馈方式。有两种系统较为常  用：带有温度和呼吸传感器的大型治疗系统(SRS Orion  PC－12)和便携式家用小型治疗系统(U－ControlT“EMGHome Trainer)，为CTD－SYSNETICS公司生产。  (**2**) 压力测定介导的生物反馈方式(manometrybasedbiofeedback method)其机理为使用肛门括约肌探头进行括约肌压力  测定，通过压力变化行生物反馈治疗。  (**3**) 其他生物反馈方式 Fleshman等发明了一种可以上下摆动，同时也可以发出声音信号的光棒来训练病人。首先，插人直  肠带电极的塞子，记录静息、屏息及用力排塞时的肌肉活动，然后指导病人控制肌肉的活动。  超短波、短波、水疗、矿泉水浴、按摩等理疗方法作为辅助治疗可有帮助。  高电位治疗 | | | | | |
| --- | --- | --- | --- | --- | --- |
| 临床上无理想治疗方法 ，目前广泛采用的常规导泻剂虽然有效 ，但均有不同程度的副作用 ，如干扰肠道正常活动和吸收 ， 降低肠壁感受细胞的应激性等，还可造成病人对[药物依赖](https://baike.baidu.com/item/%E8%8D%AF%E7%89%A9%E4%BE%9D%E8%B5%96/11034384?fromModule=lemma_inlink)性 ，长期使用可造成便秘的恶性循环。而高电位治疗器治疗功能性便秘 避免了以往治法的弊端 ，完全突破了以泻治秘的常规疗法 ，取得满意效果 ，高于常规导泻方法。在总便次数、软便次数的增加 及无便日、硬便次数、排便时间减少的五项指标上，无论是治疗期 ，还是停疗期均较常规导泻法有显著性差异(P< 0 . 0 1 )。从 高电位的角度探讨其治疗机理，包括以下四方面： (1 )刺激作用(振动效果) ：高电位的正负相位变化 ，即是刺激 ，它对活跃细胞 ，调节神经机能等都有影响； (2 )电离作用 ：施加高电位因电离的作用 ，膜的通透性增加 ，提高了失神经肌纤维膜对钾的通透 性； (3 )植物神经的调节作用：电位负荷可以减轻副交感神经的紧张和调节植物神经的功能。 (4)水束分解作用 ：在人体内起着运 输营养、氧、排泄废物的水，负荷高电位后活动加剧。上述四方面的作用最终达到调节肠道的功能 ，加强肠管节率性推进 ，促 进肠蠕动而排便 ，且以软便为主。另外 ，高电位治疗器治疗时 ，仅个别患者有发热感、疲乏感，在降低电压 ，缩短治疗时间后 消失。 [2-3] | | | | | [女 口](javascript:void(0);) |
| 参考资料 | | | | | |
| 1  中华中医药学会。 便秘诊疗指南。 中国中医药现代远程教育， 2011年09卷 第17期  2  周晓娜，刘培茹，张莉。 生物反馈治疗老年慢性功能性便秘的肛门直肠动力学改变。 中国老年学杂志， 2008年28卷 第03期  3  李延青，于岩波 .功能性便秘的诊断与治疗 .中国实用内科杂志 2011年31卷02期 | | | | | |
| 岔 搜索发现  [老人便秘怎么回事](https://www.baidu.com/s?word=%E8%80%81%E4%BA%BA%E4%BE%BF%E7%A7%98%E6%80%8E%E4%B9%88%E5%9B%9E%E4%BA%8B&tn=SE_baikepcxf02_fcetbk02&pos=baike_pc_turbo_1767&ori_sid=00bb353b9ad746fa)  [为什么老是便秘](https://www.baidu.com/s?word=%E4%B8%BA%E4%BB%80%E4%B9%88%E8%80%81%E6%98%AF%E4%BE%BF%E7%A7%98&tn=SE_baikepcxf02_fcetbk02&pos=baike_pc_turbo_1767&ori_sid=00bb353b9ad746fa) | [便秘的主要原因](https://www.baidu.com/s?word=%E4%BE%BF%E7%A7%98%E7%9A%84%E4%B8%BB%E8%A6%81%E5%8E%9F%E5%9B%A0&tn=SE_baikepcxf02_fcetbk02&pos=baike_pc_turbo_1767&ori_sid=00bb353b9ad746fa)  [功能行便秘](https://www.baidu.com/s?word=%E5%8A%9F%E8%83%BD%E8%A1%8C%E4%BE%BF%E7%A7%98&tn=SE_baikepcxf02_fcetbk02&pos=baike_pc_turbo_1767&ori_sid=00bb353b9ad746fa) | [便秘会有什么症状](https://www.baidu.com/s?word=%E4%BE%BF%E7%A7%98%E4%BC%9A%E6%9C%89%E4%BB%80%E4%B9%88%E7%97%87%E7%8A%B6&tn=SE_baikepcxf02_fcetbk02&pos=baike_pc_turbo_1767&ori_sid=00bb353b9ad746fa)  [功能性便秘如何治](https://www.baidu.com/s?word=%E5%8A%9F%E8%83%BD%E6%80%A7%E4%BE%BF%E7%A7%98%E5%A6%82%E4%BD%95%E6%B2%BB&tn=SE_baikepcxf02_fcetbk02&pos=baike_pc_turbo_1767&ori_sid=00bb353b9ad746fa) | [经常便秘怎么办](https://www.baidu.com/s?word=%E7%BB%8F%E5%B8%B8%E4%BE%BF%E7%A7%98%E6%80%8E%E4%B9%88%E5%8A%9E&tn=SE_baikepcxf02_fcetbk02&pos=baike_pc_turbo_1767&ori_sid=00bb353b9ad746fa)  [预防便秘的方法有哪些](https://www.baidu.com/s?word=%E9%A2%84%E9%98%B2%E4%BE%BF%E7%A7%98%E7%9A%84%E6%96%B9%E6%B3%95%E6%9C%89%E5%93%AA%E4%BA%9B&tn=SE_baikepcxf02_fcetbk02&pos=baike_pc_turbo_1767&ori_sid=00bb353b9ad746fa) | [长期便秘的解决方法](https://www.baidu.com/s?word=%E9%95%BF%E6%9C%9F%E4%BE%BF%E7%A7%98%E7%9A%84%E8%A7%A3%E5%86%B3%E6%96%B9%E6%B3%95&tn=SE_baikepcxf02_fcetbk02&pos=baike_pc_turbo_1767&ori_sid=00bb353b9ad746fa)  [便秘并发症](https://www.baidu.com/s?word=%E4%BE%BF%E7%A7%98%E5%B9%B6%E5%8F%91%E7%97%87&tn=SE_baikepcxf02_fcetbk02&pos=baike_pc_turbo_1767&ori_sid=00bb353b9ad746fa) | |

| Q | 新手上路 [成长任务](https://baike.baidu.com/usercenter/tasks#guide)  [编辑规则](https://baike.baidu.com/help#main06) | [编辑入门](https://baike.baidu.com/help#main01)  [本人编辑](https://baike.baidu.com/item/%E7%99%BE%E5%BA%A6%E7%99%BE%E7%A7%91%EF%BC%9A%E6%9C%AC%E4%BA%BA%E8%AF%8D%E6%9D%A1%E7%BC%96%E8%BE%91%E6%9C%8D%E5%8A%A1/22442459?bk_fr=pcFooter) |  | 我有疑问 [内容质疑](javascript:void(0);) [官方贴吧](http://tieba.baidu.com/f?ie=utf-8&fr=bks0000&kw=%E7%99%BE%E5%BA%A6%E7%99%BE%E7%A7%91) | 投诉建议  [举报不良信息](http://help.baidu.com/newadd?word=%E5%8A%9F%E8%83%BD%E6%80%A7%E4%BE%BF%E7%A7%98&&submit_link=https%3A%2F%2Fbaike.baidu.com%2Fitem%2F%25E5%258A%259F%25E8%2583%25BD%25E6%2580%25A7%25E4%25BE%25BF%25E7%25A7%2598%3FfromModule%3Dlemma_search-box&prod_id=10&category=1) [投诉侵权信息](http://help.baidu.com/newadd?word=%E5%8A%9F%E8%83%BD%E6%80%A7%E4%BE%BF%E7%A7%98&&submit_link=https%3A%2F%2Fbaike.baidu.com%2Fitem%2F%25E5%258A%259F%25E8%2583%25BD%25E6%2580%25A7%25E4%25BE%25BF%25E7%25A7%2598%3FfromModule%3Dlemma_search-box&prod_id=10&category=6)  [在线客服](http://zhiqiu.baidu.com/baike/passport/html/baikechat.html) [意见反馈](javascript:void(0);) |
| --- | --- | --- | --- | --- | --- |

©2022 Baidu [使用百度前必读](http://www.baidu.com/duty/) | [百科协议](http://help.baidu.com/question?prod_en=baike&class=89&id=1637) | [隐私政策](http://help.baidu.com/question?prod_id=10&class=690&id=1001779) | [百度百科合作平台](https://baike.baidu.com/operation/cooperation) | 京ICP证030173号 [京公网安备11000002000001号](http://www.beian.gov.cn/portal/registerSystemInfo?recordcode=11000002000001)

<https://baike.baidu.com/item/>功能性便秘?fromModule=lemma_search-box

[未通过词条申诉](http://help.baidu.com/newadd?word=%E5%8A%9F%E8%83%BD%E6%80%A7%E4%BE%BF%E7%A7%98&&submit_link=https%3A%2F%2Fbaike.baidu.com%2Fitem%2F%25E5%258A%259F%25E8%2583%25BD%25E6%2580%25A7%25E4%25BE%25BF%25E7%25A7%2598%3FfromModule%3Dlemma_search-box&prod_id=10&category=2)

[封禁查询与解封](http://help.baidu.com/newadd?word=%E5%8A%9F%E8%83%BD%E6%80%A7%E4%BE%BF%E7%A7%98&&submit_link=https%3A%2F%2Fbaike.baidu.com%2Fitem%2F%25E5%258A%259F%25E8%2583%25BD%25E6%2580%25A7%25E4%25BE%25BF%25E7%25A7%2598%3FfromModule%3Dlemma_search-box&prod_id=10&category=5)

7/7
