# Supplementary material for: Assessment of medical information on irritable bowel syndrome information in Wikipedia and Baidu Encyclopedia: comparative study
Source: PeerJ. 2024 May 24;12:e17264. doi: 10.7717/peerj.17264 (PMC11129691; doi:10.7717/peerj.17264)
Supplement: Data S1 [file peerj-12-17264-s001.zip › σÄƒσoïμò░μì«/Baidu/Baidu-English/4-μÿôμ┐Çτ╗ôΦéá_τÖ╛σ║aτÖ╛τoæ.docx]

| 2022/12/14 10:40  [网页](https://www.baidu.com/) | [新闻](http://news.baidu.com/) | 易激结肠_百度百科  [贴吧](https://tieba.baidu.com/) [知道](https://zhidao.baidu.com/) [网盘](https://pan.baidu.com/?from=1027327l) | [图片](http://image.baidu.com/) | [视频](http://v.baidu.com/) | [地图](http://map.baidu.com/) | [文库](https://wenku.baidu.com/) | 百科 | [百度首页](http://www.baidu.com/) [登录](javascript:;) |
| --- | --- | --- | --- | --- | --- | --- | --- | --- |

| [岔](https://baike.baidu.com/) | \| 易激结肠 \| 进入词条 \| \| --- \| --- \| | \| 全站搜索 \| \| --- \| | [帮助](https://baike.baidu.com/help) |
| --- | --- | --- | --- | --- | --- | --- |
| 近期有不法分子冒充百度百科官方人员，以删除词条为由威胁并敲诈相关企业。在此严正声明：百度百科是免费编辑平台，绝不存在收费代编服务，请勿上当受骗！ [详情>>](https://baike.baidu.com/common/declaration) | | | |
| [首页](https://baike.baidu.com/) 秒懂百科 特色百科 用户 知识专题 权威合作 [口下载百科APP](https://baike.baidu.com/wapui/subpage/baikeappdownload?sfrom=pc_lemmapage_navigation) [2 个](https://baike.baidu.com/usercenter) | | | |

| 易激结肠  医学疾病 | \| [小播报](javascript:;) \| \| --- \| | \| [c编辑](javascript:;) \| \| --- \| | \| [讨论](https://baike.baidu.com/planet/talk?lemmaId=17002016&fromModule=lemma_right-issue-btn) \| \| --- \| | \| [上传视频](javascript:;) \| \| --- \| | . 收藏 [山 0](javascript:void(0);) 3 | \| 词条统计  浏览次数： 5609次  编辑次数： 8次[历史版本](https://baike.baidu.com/historylist/%E6%98%93%E6%BF%80%E7%BB%93%E8%82%A0/17002016)  最近更新： [杨家将1206](https://baike.baidu.com/usercenter/userpage?uk=_99_yWI_eYDMb13pqBkNew&from=lemma) ( 2022-06-05)  突出贡献榜  [hnta933](https://baike.baidu.com/usercenter/userpage?uk=OCtpcvTM1OlG7i9SsNPcdg&from=lemma) \| \| --- \| |
| --- | --- | --- | --- | --- | --- | --- | --- | --- | --- | --- | --- |
| 本词条缺少概述图，补充相关内容使词条更完整，还能快速升级，赶紧来编辑吧！  Irritable bowel syndrome (IBS) is a complex syndrome related to changes in gastrointestinal function, with chronic or recurrent abdominal pain, diarrhea, abnormal bowel habits and stool characteristics as the main symptoms, and the absence of gastrointestinal structural or biochemical abnormalities. sign. | | | | | |  |
|  |  |  |  |  |  | \| **1** csgo电脑配置 **12** 购买域名  **2** 电商平台怎么 **13** sci论文投稿  **3** 网络安全培训 **14** 37游戏平台  **4** 无人机反制 **15** 价格便宜的  **5** 自己创建个网 **16** vr消防演练  **6** csgo网站开箱 **17** 哈佛大学申  **7** 亚马逊图书 **18** 网络工程师  **8** 图书批发网 **19** 战队logo设  **9** 出版社自费出 **20** 虚拟货币平  **10** 国际期货 **21** 怎么创建小  **11** 游戏盒子 **22** 供应链管理 \| \| --- \| |
| irritable bowel syndrome  易激结肠  外文名  **ICD**号  中文名  K63.8 | | | | | |  |
| \| 目录 \| ▪ [肠运动异常](#_bookmark1)  ▪ [甲状腺疾病](#_bookmark9)  16 [易激结肠的治疗](#_bookmark10)  ▪ [治疗原则](#_bookmark11)  ▪ [生活和饮食调节](#_bookmark12)  ▪ [精神治疗](#_bookmark13)  ▪ [药物治疗](#_bookmark14)  ▪ 乙状结肠镜或纤维结 肠镜检查  ▪ [肠道动力检查](#_bookmark15)  1 [基本信息](#_bookmark16) 2 [疾病名称](#_bookmark17) 3 [英文名称](#_bookmark18) 4 [别名](#_bookmark19)  5 [分类](#_bookmark20)  6 [ICD号](#_bookmark21) 7 [流行病学](#_bookmark22)  8 [病因](#_bookmark23)  14 [诊断](#_bookmark24)  15 [鉴别诊断](#_bookmark25)  ▪ [慢性细菌感染](#_bookmark26)  ▪ [慢性阿米巴痢疾](#_bookmark27)  ▪ [血吸虫感染](#_bookmark28)  ▪ [吸收不良综合征](#_bookmark29)  ▪ [肠肿瘤](#_bookmark30)  ▪ [溃疡性结肠炎](#_bookmark31)  ▪ [克罗恩病](#_bookmark32)  ▪ [乳糖酶缺乏](#_bookmark33)  ▪ [胃肠道内分泌肿瘤](#_bookmark34)  17 [预后](#_bookmark35)  18 [易激结肠的预防](#_bookmark36) 19 [相关药品](#_bookmark37)  20 [相关检查](#_bookmark38)  ▪ [感觉异常](#_bookmark2)  ▪ [分泌异常](#_bookmark3)  10 易激结肠的临床  表现  ▪ [症状](#_bookmark4)  ▪ [体征](#_bookmark5)  11 易激结肠的并发 症  12 [实验室检查](#_bookmark6)  ▪ [精神、神经因素](#_bookmark39)  ▪ [肠道刺激因素](#_bookmark40)  9 [发病机制](#_bookmark41)  13 [辅助检查](#_bookmark7)  ▪ [X线钡灌肠检查](#_bookmark8) \| \| --- \| --- \| | | | | | |  |
|  |  |  |  |  |  | [女疊 口](javascript:void(0);) |
| 基本信息  [小 播报c编辑](javascript:;) | | | | | |  |
| Often coexisting with other functional gastrointestinal disorders such as gastroesophageal reflux disease (GERD) and functional dyspepsia (FD). Examination is required to rule out this can cause this  Some symptoms of organic diseases. The disease belongs to gastrointestinal dysfunction diseases, including abdominal pain, abdominal distention, defecation habits and abnormal stool characteristics, mucous stool, persistent or intermittent attacks  and lack of morphological and biochemical abnormalities that can explain the syndrome. It is characterized by irritability of intestinal function.  疾病名称  [小 播报c编辑](javascript:;)  易激结肠  英文名称  [小 播报c编辑](javascript:;)  irritable bowel syndrome  [小 播报c编辑](javascript:;)  别名  [；肠应激综合征；过敏性肠综合征；过敏性大肠综合征； ； ；黏液性结肠炎； 肠易激综](https://baike.baidu.com/item/%E8%82%A0%E6%98%93%E6%BF%80%E7%BB%BC%E5%90%88%E5%BE%81/8456761?fromModule=lemma_inlink)  [合征；应激性结肠综合征](https://baike.baidu.com/item/%E8%82%A0%E6%98%93%E6%BF%80%E7%BB%BC%E5%90%88%E5%BE%81/8456761?fromModule=lemma_inlink)  分类  [小 播报c编辑](javascript:;)  消化科 > 肠道疾病 > 大肠疾病  [小 播报c编辑](javascript:;)  ICD号  K63.8 | | | | | | |

<https://baike.baidu.com/item/>易激结肠?fromModule=search-result_lemma

1/6

2022/12/14 10:40

[女疊口](javascript:void(0);)

流行病学

易激结肠_百度百科

[小 播报c编辑](javascript:;)

IBS is one of the most common digestive disorders. According to Western statistics, IBS accounts for about 14% ~ 22% of the adult population, and women are 1.38 times that of men, of which only 50%.

IBS patients seek medical attention. Other data show that the prevalence in European and American populations is about 7.1% ~ 13.6%. In 1994, China's adult population survey (non-random) in 9 provinces and cities in the north and south of China was found

The incidence of abdominal pain and relief after stool was 22.1% more than 6 times a year. According to Peking Union Medical College Hospital, the results of the survey of the general population aged 18~70 in urban and rural areas of Beijing in 1996 have

The prevalence of IBS symptoms in people who meet Manning's criteria is 7.01%, and it is more common in young people aged 18~30, of which only 20% seek medical treatment.

病因

The cause of IBS is unknown. It is currently thought to be related to the following factors.

Psychiatric, neurological factors

The incidence of psychiatric abnormalities in IBS patients is significantly higher than that of ordinary people. Studies have shown that mental stress can change the MMC of the intestine, and mental stimulation is more likely to cause intestinal motility disorders in IBS patients than normal people. Modern neurophysiology believes that the intestines of IBS patients have increased sensitivity to tension and multiple stimuli. However, it is not clear whether this is due to abnormalities in the intestinal wall plexus and its receptors or afferent nerve pathways, or abnormalities in the regulation of the bowel by the central nervous system. In addition, studies have found that stress can cause functional enteromotility disorder in rats, and it has been found that the release of some gastrointestinal hormones increases after stress, indicating that neuroendocrine regulation is involved in the response process of intestinal dysfunction caused by stress. The above relationship between mental and neurological factors and IBS supports the current view that IBS is a physical and mental disease type of gastrointestinal disease.

Intestinal irritating factors

Certain factors in the intestine may alter bowel function and aggravate the pre-existing irritable colon. These stimuli include external foods, drugs, microorganisms, etc., and may also include certain internal substances produced during digestion. Experiments have found that the intestinal tract sensitized by intraluminal antigen stimulation can significantly induce intestinal contractile activity and produce diarrhea in rats. Some analysts believe that when certain irritants act on the intestine many times, they may change the sensorimotor function of the intestine and sensitivity to stimuli, so that the intestinal tube produces "irritability". It has been reported that the ileum in IBS is very sensitive to the secretion of perfusion bile acids, but may not be diagnosed with bile acid malabsorption. Short- or medium-chain fatty acids may reach the right colon in patients with limited absorption capacity or rapid movement in the small intestine, causing high pressure waves passing rapidly through the right colon, which are extremely effective in advancing colonic contents and may cause pain and diarrhea. Whether these intestinal irritants are the cause or cause in IBS is still undetermined.

[小 播报c编辑](javascript:;)

[小 播报c编辑](javascript:;)

发病机制

Abnormal bowel movements

The main pathogenesis of IBS is abnormal bowel motor function. Studies have found that patients with IBS have increased discreted clustered constrictions (DCCs) and prolonged propagated constrictions (PPCs), which are consistent with spasmodic pain. In patients with diarrheal IBS, the number of migrating motor complex (MMc) during the day is increased, and the cycle is shortened; More jejunal constriction in stage II and after meals; The colon shows a large number of rapid and progressive contractions; rapid passage of the proximal colon and positively correlated with stool weight; Cholinergic stimulation increases a number of motility indicators of the desigmoid colon. In contrast, patients with constipated IBS have prolonged proximal colonic passage time and significantly slowed down emptying; Reduction of high-amplitude propulsive contraction; The contraction frequency and contraction time of the desigmoid colon in the basal state decreased, and the responsiveness to cholinergic stimulation decreased, while the percentage of contraction time of the proximal colon increased significantly, manifested as incoordination. Pressure in the canal rises, and the sphincter is retarded in response to rectal dilation. Abnormal contraction of the external sphincter during defecation is associated with dysfecation in IBS.

IBS motility abnormalities are not limited to the intestine, esophagus, stomach, biliary tract have power disorders, so that it is called "gastrointestinal asthma (asthma of gut)". Current research on IBS dynamics is not fully consistent, and some even lead to the opposite. It shows that the dynamic disorder of IBS is very complex, it is not only a certain abnormality of a certain intestinal segment of power, but also has a problem of coordination between them.

paresthesia

People with IBS have a lower threshold of abdominal pain than normal people and therefore develop an excessive feeling of standard colonic dilation. The synergistic effect of this paresthesia with cluster movement abnormalities is a major factor in the development of crampy pain in IBS. Mental stress and anxiety worsen the pain sensation when the colon is dilated. Conversely, the perception of bowel dilation is reduced in the relaxed state. Abnormal rectal and sensations cause incomplete bowel movements, even abdominal pain before bowel movements. Excessive sensation in the rectum and anus is accompanied by excessive reflex movements in the rectum. That is, incomplete bowel movements cause increased motor response, resulting in increased bowel frequency, but not with increased bowel weight.

Abnormal secretion

In patients with IBS, the mucosa of the small intestine reacts to the secretion of irritating substances is enhanced. Increased mucus secretion by the colonic mucosa.

易激结肠的临床表现

[小 播报c编辑](javascript:;)

症状

symptom

(1) Abdominal pain, abdominal discomfort: often along the intestinal tube discomfort or abdominal pain, can develop into colic, lasting a few minutes to hours, relieved after exhaust and defecation. Some foods such as crude fiber vegetables, coarse fruits, strong condiments, wine, cold drinks, etc., can induce abdominal pain. However, abdominal pain does not worsen. No seizures during sleep.

(2) Diarrhea or unformed stool: often after meals, especially after breakfast, multiple bowel movements. It can also occur the rest of the time, but not at night. Occasionally stool up to 10 or more. However, the amount of stool per time is small, and the total amount rarely exceeds the normal range. Sometimes the stool is only 1 ~ 2 times, but it is not formed. Diarrhea or unformed stools sometimes alternate with normal stools or constipation.

(3) Constipation: 1 ~ 2 times a week, occasionally once in more than 10 days. Laxatives are required for early on, intermittent, and late sustainability.

(4) Abnormal bowel process: patients often have symptoms such as difficulty defecating, incomplete bowel movements or urgency.

(5) Mucus stool: stool often carries a small amount of mucus. However, occasionally a large amount of mucus or mucus casts are discharged.

(6) Abdominal distention: obvious during the day, reduced after night sleep, generally abdominal circumference does not increase.

2/6

| 体征  The cecum and sigmoid colon are often palpable, and the cecum is usually inflated with inflated bowel sensation; the sigmoid colon often has a cord-like spasm or palpation of the fecal mass. The bowel may be mildly tender,  However, the tenderness is not fixed, and the pain disappears with continuous compression. Some patients have pain on digital examination and a feeling of increased sphincter tone. | | | | |
| --- | --- | --- | --- | --- |
| 易激结肠的并发症 | | | [小 播报c编辑](javascript:;) | |
| The onset or worsening of symptoms is often related to psychiatric factors or some stressful state. Some patients have symptoms of various functional disorders of the upper gastrointestinal tract and outside the intestine. It can also be accompanied by psychological essence  Abnormal manifestations of God, such as depression, suspiciousness, nervousness, anxiety, hostility, etc. | | | | |
| 实验室检查 | | [小 播报c编辑](javascript:;) | | |
| Stool is watery, soft or hard, and may be mucus. There are no other abnormalities. | | | | |
| 辅助检查 | [小 播报c编辑](javascript:;) | | | |
|  | | | | |
| X-ray barium enema examination  Often no abnormalities were found. In a small number of cases, "line sign" occurs due to intestinal spasm. Other nonspecific manifestations may include deepening or increasing of the colonic pouch.  Sigmoidoscopy or fiberoptic colonoscopy  There was no abnormality in the mucosa observed with the naked eye, and there was no abnormality in the biopsy. However, it can cause cramping, pain when the lens is inserted, or pain when it is inflated. If splenic area syndrome is suspected, 100-200ml of gas can be slowly injected during the examination, and then the mirror can be pulled out quickly, and the patient is asked to sit up. After 5-10 minutes, left upper abdominal pain will appear and radiate to the left shoulder. Objective signs of splenic area syndrome.  Some doctors put a balloon in the rectum, and the patient experienced pain after inflating it. When allergic colon patients have abdominal pain, the pressure of the air sac is significantly lower than that of normal people.  bowel motility test  Distinct and imperfect from esophagus and stomach.  (1) Bowel transit time check:  ①Hydrogen breath test method: the principle is that sugars that cannot be absorbed in the small intestine, such as lactulose, are fermented by bacteria in the colon to release hydrogen gas, which is then exhaled through the lungs. Therefore, after taking lactulose orally, the expired hydrogen was collected after a certain period of time (10-15 minutes), and the concentration of expired hydrogen was measured by gas chromatography. According to the change of the hydrogen concentration in the exhaled gas, the mouth-blind transit time was calculated. When the breath hydrogen concentration is higher than 50% of the base value or higher than 4~10ppm level, it is the peak value, and the time from oral administration of lactulose to the peak value is the mouth-blind transit time. Some factors affect the test results, such as taking lactulose on an empty stomach. For different patients, the digestive phase is different at that time, resulting in inhomogeneity in the results of oral-blind passage time. Therefore, lactulose should be taken at the same time as the test meal. Because the digestive period is stopped immediately after eating, the activities of the digestive period are replaced. In this way, the conditions of the subjects are the same. If given with a liquid test meal, it represents the transit time of the liquid, and if given with the solid, it represents the transit time of the solid. The composition of the trial meal should be a prescribed meal similar to that of the ordinary meal; the amount of exercise will affect the motivation, so the same amount of exercise should be prescribed; drugs will affect the breath test, and it is required to stop taking anticholinergic drugs, calcium ion channel blockers, nitroglycerin, and sedation 48 hours before the test Drugs and psychotropic drugs, no antibiotics within 1 month; In addition, the influence of gastric emptying function and digestive tract bacteria should also be considered.  ② Radionuclide scanning method:  A. Determination of small intestine transit time: usually mark the test meal with 99mTC, and count it under the gamma camera after the test meal (nuclide scanning of front and rear body positions to correct errors). If two radionuclides are used, one is used for gastric emptying and the other is used to measure the oral-blind transit time, and the small intestinal transit time can be deduced from this.  B. Determination of colon transit time: inject radionuclide-labeled liquid into cecum or orally orally disintegrate radionuclide capsule located in cecum. Thus the filling of the colon and the transit time of each segment are measured, the disadvantage of which is a non-physiological marker. In contrast, the oral capsule method is closer to the physiological situation.  ③ X-ray-opaque marker method: taking one or more kinds of X-ray-opaque markers after oral administration (with a certain interval of time) and taking regular radiographs. The bony landmarks on the plain film and the moving direction of the landmarks on the continuous radiographs are used to judge the position of the landmarks on the plain film, and to calculate the total gastrointestinal transit time, oral-blind transit time, total colon and each segment of colon transit time .  (2) Pressure measurement:  ①Measurement of small intestinal pressure: insert the pressure collecting tube or the catheter with miniature pressure sensor into the duodenum through the stomach to the upper part of the jejunum (guide wire is required under X-ray, and can be inserted through an endoscope), and the digestive space of the small intestine can be measured. Dynamic activity (number of contractions, contraction amplitude and dynamic index) during the period of digestion and digestion. There are many influencing factors, the air bubbles in the catheter affect the accuracy of pressure measurement; the intestinal lumen does not disappear when the intestinal tract shrinks, which will affect the accuracy of the recording; various drugs affect the accuracy, and the technique and cooperation during intubation and measurement are also important influences factor. It is very important to standardize the instrument, otherwise it will cause measurement errors.  ②Colon pressure measurement: send the guide wire through the biopsy hole of the colonoscope, then put the pressure catheter into the guide wire, and send it to the colon under the guidance of X-rays, and record the dynamic activity of fasting and after meals or after administration . The influencing factors are as follows: fasting and meal activities are not consistent; drug effects are obvious; the results of different methods vary greatly; the insertion technique requires lightness and speed, otherwise the results will be affected. | | | | [女疊 口](javascript:void(0);) |
|  |  |  |  |  |
|  |  |  |  |  |
| [小 播报c编辑](javascript:;)  诊断 | | | |  |

<https://baike.baidu.com/item/>易激结肠?fromModule=search-result_lemma

3/6

2022/12/14 10:40 易激结肠_百度百科

[女](javascript:void(0);) [疊口](http://baike.baidu.com/l/WWoXYu7P)

Symptoms of a functional intestinal disorder are diagnosed, and after various possible organic lesions are excluded. Diagnostic criteria for irritable colonic symptoms are not uniform,

And constantly revised. The 1992 Rome standard is currently widely used internationally:

1. Symptoms persist or recur for more than 3 months.

2. Must have the following symptoms

(1) Abdominal pain or abdominal discomfort, and has the following characteristics: relief after defecation; and/or accompanied by changes in stool characteristics.

(2) Abnormal bowel movements occur at least 25% of the time, with at least the following 2 types: changes in stool frequency (> 3 times / day or < 3 times / week); changes in stool characteristics (hard: lumps (or) loose: watery stool); changes in the bowel process (strenuous or urgent or incomplete bowel movements); mucus discharge, accompanied by flatulence or bloating.

According to its main manifestations, irritable colon can be divided into different types, generally divided into diarrhea-predominant (IBS-D) and constipation-predominant (IBS-C) two categories, as well as various mixed types.

鉴别诊断

[小 播报c编辑](javascript:;)

Chronic bacterial infections

Multiple positive findings on routine stool and culture, as well as adequate and effective systemic antibiotic therapy, significantly improve symptoms and confirm the diagnosis.

Chronic amoebic dysentery

Multiple stool attempts to find amoeba and metronidazole test can confirm the diagnosis.

Schistosomiasis infection

Patients in schistosomiasis-endemic areas can undergo sigmomorphoscopy, take the rectal mucosa to look for schistosomiasis eggs, or identify them by fecal hatching and other methods.

Malabsorption syndrome

There is diarrhea, but there is often fat and undigested food in the stool.

Intestinal tumors

Benign small tumors of the small intestine can develop diarrhea and intermittent episodes of partial ileus. Colon tumors can also present with symptoms similar to functional diseases of the intestine. Especially for the elderly, attention should be paid to. Barium radiographs or colonoscopy may be done to confirm the diagnosis.

Ulcerative colitis

There are abnormal manifestations such as fever, pus and bloody stools. This can be identified by barium radiography or colonoscopy.

Crohn's disease

There are often systemic symptoms such as fever, anemia, and weakness. Barium radiography or colonoscopy can be used to identify the difference.

Lactase deficiency

A lactose tolerance test can be used to differentiate. Lactase deficiency is congenital and acquired. The clinical manifestation is severe diarrhea after eating dairy products, and the stool contains a lot of foam and lactose, lactic acid. Symptoms can be improved by removing milk or dairy products from food. Kefir is broken down by lactic acid bacteria and can be consumed by such patients.

Gastrointestinal endocrine tumors

Gastrinomas can present with severe diarrhea and stubborn ulcer disease, and serum gastrin levels are extremely high, and general treatment is ineffective. Vasoactive intestinal peptide tumors (Vipoma) also cause severe diarrhea; Serum VIP levels are elevated.

Thyroid disease

Diarrhea can occur with hyperthyroidism. Hyperparathyroidism can lead to constipation. Thyroid and parathyroid function tests can be used to differentiate.

易激结肠的治疗

[小 播报c编辑](javascript:;)

Principles of treatment

IBS has complex etiology, many symptoms and easy to recur, and cannot rely solely on specific drug treatment, and comprehensive systemic treatment needs to be used according to different individuals.

Life and diet regulation

Avoid predisposing factors, choose a diet that is easy to digest, less fat, and avoid irritating and sensitive foods. For constipation and bloating, you can eat more fiber-rich but not easy to produce gas, and avoid overeating and snacking. Patients with predominantly diarrhea should eat less foods containing crude fiber.

Psychiatric treatment

Mental status is closely related to intestinal symptoms. Medical workers should explain the nature and precautions of the disease to patients with sympathy and responsibility, and should relieve the patient's mentality of many doubts, so that they can eliminate their fear and enhance their confidence in overcoming the disease. Sedation and antidepressants should be used if necessary. It can be used to dededetamine 50mg, 3 times / d, or fluoxidine hydrochloride (Prozae) 20mg/d, or with haloperthioxine / dimethylamine propylene / dimethylamine propene (Dianshen), 2 tablets a day in the morning, oral administration, to relieve its mental abnormalities, so that abdominal pain and other discomfort can be relieved. You can also choose amitriptyline 25mg, 2 times / d, doxepin 25mg, 2 ~ 3 times / d, poor sleep patients take diazepam (diazepam) and so on

4/6

2022/12/14 10:40 易激结肠_百度百科

[女](http://baike.baidu.com/l/WWoXYu7P)  [疊口](javascript:void(0);)

Medication

Although it can reduce symptoms, it cannot prevent recurrence, so drugs should be used rationally and drug abuse should be avoided.

(1) Anticholinergic drugs: such as scopolamine (654-2), benatidine (gastric rehabilitation), etc., due to more adverse reactions, gradually replaced by other drugs. At present, it is recommended to use mebevirin (malpivanlin) 10mg; Dicyclovirine 10 mg; Pyrifen-olfactory ammonium (prifiniumbromide) 30 mg; Oral administration 3 times / day. Citropium bromide 50mg was also used before meals to achieve good results.

(2) Acetamide hydrochloride (acebutyramide hydrochloride): has a single anti-exercise, anticholinergic effect without anesthesia, the dosage is 2mg, 3 ~ 4 times / d. Clonidine (clonidine) 0.3 ~ 0.4mg, 3 times / d, oral, can promote the absorption of liquid substances in the small intestine, enhance the absorption of electrolytes by the colon, slow down the transit time of the small intestine, and have a better antidiarrheal effect.

(3) Calcium channel blockers: nifedipine (heartache) 10 ~ 20mg or verapamil (isopamide) 40mg, 3 times / day oral, can inhibit gastrocolic reflex, relieve abdominal pain, reduce stool.

(4) Opioid antidiarrheal agent: loperamide (Yimeng) acts on the opioid receptors of the intestinal wall, blocks the release of acetylcholine and prostaglandins, inhibits intestinal peristalsis, and increases water and electrolyte absorption. Each oral administration of 2mg, 2 ~ 3 times / d, the daily dosage should not exceed 10mg. After the formation of the stool, the dosage can be gradually reduced until the drug is stopped. Some patients experience side effects such as dry mouth, bloating, and even pseudo-intestinal obstruction. Also taken, this drug is a petidine derivative, in addition to antidiarrheal effect, but also has the effect of stimulating the central nervous system, large doses of analgesia and euphoria, long-term use is dependent.

(5) Microecological regulator: through microbiology technology, the normal flora in the human body is isolated, after pure culture, industrial production, made into probiotic products, and then returned to the human body according to the original path, adjust microecological disorders, and achieve the role of preventing and treating diseases and enhancing immune function. In addition to probiotic products (Probiotics), microecological regulators also have beneficial bacteria growth promotion substances called prebiotcs. After taking probiotic preparations, they enter the intestine, quickly colonize the intestinal mucosa, and rapidly multiply to form a biological barrier, decompose glucose to produce lactic acid, reduce intestinal pH, inhibit the reproduction and growth of pathogenic bacteria, correct intestinal flora imbalance, restore and maintain the stability of the intestinal microbial ecosystem, and change intestinal motor function. Common probiotic preparations are:

A. Oral bifidobacterium triple viable bacteria preparation (Pefican): It is composed of bifidobacteria, acidophilus, fecal streptococcus, etc., and is resistant to antibiotics and chemotherapy drugs, so that after taking antibiotics and chemotherapy drugs, the intestinal flora is balanced and some symptoms caused by dysbacteriosis are eliminated. Take 2 ~ 4 capsules each time, 2 ~ 3 times/d.

B. Lactobacillus acidophilus (Le Tor): mainly contains Lactobacillus acidophilus and its metabolites, which has the effect of inhibiting the growth of intestinal pathogenic bacteria and preventing the adhesion of bacteria, viruses and intestinal villi. Take 1 capsule each time, oral 3 ~ 4 times.

C. Juketon: mainly contains three kinds of lactic acid bacteria, Lactobacillus acidophilus, Lactobacillus lactis and Streptococcus lactis, which are resistant to a variety of antibiotics. 2 capsules each time, 3 times / d.

D. Christconn (gastrferm) is mainly active Enterococcus faecalis and can tolerate a variety of antibiotics. 1 ~ 2 capsules each time, 2 ~ 3 times/d.

E. Clostridium caseinate (Mia BBM): Uterine bacteria (caseic acid bacteria) is Clostridium anaerobic spore, which is not affected by gastric acid, bile, etc. in the body, and is a normal flora in the body, which can prevent the colonization of harmful bacteria and correct the disorders of intestinal flora, 2 tablets per serving (including 40mg of uterine bacteria), 3 times / d. In addition, there are bifidobacteria (Lizhu entero), Bacillus licheniformus (whole intestine), bifidobacteria (rejuvenation), lactobacillin, lactase birth, etc., all of which are such drugs.

(1) Dietary adjustment: eat foods that soften and expand fecal volume, such as foods with high crude fiber, drink more water in moderation, and defecate regularly.

(2) Cisapride (Prebosch): By stimulating the serotonin 4 (5-HT4) receptors of preganglionic neurons, and acting on the ganglion cells of the intestinal muscular plexus to release acetylcholine, increase gastrointestinal propulsion, and promote bowel movements. 5 ~ 10mg each time, 3 times / d half an hour before meals.

(3) Lactulose: oral administration of 15ml/d can increase the frequency of stool, make the feces soft, and relieve the difficulty of defecation. However, those with allergies should be used with caution.

(4) Oral mannitol 2 ~ 4g, 3 times / d; Or take glucomannan 1g, 3 times / d.

Pay attention to the relationship between mood and abdominal pain, suggesting therapy or topical warmth, physiotherapy, massage, or occlusion if necessary. In recent years, it has been reported that calcium channel blockers have a good effect, have a relaxing effect on gastrointestinal smooth muscle, especially on the esophagus and colon, and patients with abdominal pain can try nifedipine (heartache), and antispasmodic analgesics. Selective gastrointestinal calcium channel inhibitors can relieve gastrointestinal smooth muscle spasm and inhibit postprandial colonic motor response. It has a good treatment effect on abdominal pain in IBS patients, and also has a certain effect on diarrhea and constipation. Commonly used selective calcium channel antagonists include peveromide (deschate) 5mg/time, 3 times/d; Dicyclovirine (dicyclic amine) 10 ~ 20mg/time, 3 ~ 4 times/d; Citropium bromide 50 mg / time, 3 times / day, orally.

The gonadotropin leuprolide acetate (1euprolide acetate) is a gonadotropin-releasing hormone booster. Some authors have found that menstruating women are more likely to develop IBS symptoms, so they can try this drug. The 6-month trial found that this drug has a significant improvement effect on abdominal pain, nausea, vomiting and other symptoms in IBS patients.

Octreotide is a long-acting synthetic octapeptide somatostatin, which has inhibitory effect on most other hormones such as cholecystokin and endorphins, so it can be tried to treat IBS, but it is expensive.

Serotonin has a variety of effects on intestinal motor function in vitro, enhancing contractions or promoting relaxation, but has not been studied in humans.

(1) Hexagonal montmorillonite (Smecta): is a natural mineral, is a double octahedral montmorillonite, the drug is a layered striated structure, has strong coverage, has a strong fixation and scavenging power for viruses, bacteria and toxins, prevents intestinal epithelial cell damage, and can absorb intestinal gas, reduce intestinal sensitivity. This drug is not absorbed by cells and does not enter the blood circulation, so it has no toxic side effects and can be used in conjunction with antibiotics and probiotic preparations. Hexagonal montmorillonite (Smecta) 3g per bag, pour 50ml of warm water and stir well for oral administration, each time take 1 ~ 2 bags; Also use 1 ~ 3 bags mixed with 50 ~ 100ml warm water to retain enema, 1 ~ 3 times / d.

(2) Gonadotropin leuprolide acetate leuprolide acetate [leuprolide acetate (1eupron)]: is a gonadotropin-releasing hormone accelerator. Some people use IBS to treat abdominal pain, nausea, vomiting and other symptoms have been significantly improved.

(3) Sodium chromium copper (naloxone and natrexem): is an opiate antagonist, can make intestinal peristalsis hyperactive, good application for constipated IBS patients.

(4) Application of autonomic drugs: such as oryzanol 20 ~ 60mg each time, 3 times / d; It can also be used calcium pantothenate, cinnarizine (cinnammonazine) and so on.

Dialectical treatment or according to the candidate with hemp seed soup, seven flavors of white shusan, etc., can also try to strengthen the spleen and benefit the intestines. It has been reported that acupuncture treatment also has some efficacy.

<https://baike.baidu.com/item/>易激结肠?fromModule=search-result_lemma

5/6

2022/12/14 10:40

易激结肠_百度百科

|  | [小 播报c编辑](javascript:;)  预后  No life-threatening, can maintain a healthy life for a long time  易激结肠的预防  [小 播报c编辑](javascript:;)  Reduce adverse irritation to the digestive tract, avoid food allergies and eat less food that can produce gas in the digestive tract. Excessively spicy, sweet, sour, rough and other irritating foods should be avoided. Eat more digestible, nutritious food. Patients with constipation should take in more fiber-rich foods and fruits. For those with a history of allergies, avoid eating foods that may cause allergies. Those suspected of being lactose intolerant should avoid consuming large amounts of milk and milk products. It is advisable to chew slowly, quit smoking, and drink less carbonated beverages.  If the patient has obvious mental and nervous factors, we should start with eliminating the patient's doubts and fears to prevent the occurrence or aggravation of the disease.  相关药品  [小 播报c编辑](javascript:;)  Lactulose, nitroglycerin, glycerin, haloperithioxane, haloperithioxane/dimethylamine propene, amitriptyline, doxepin, diazepam, scopolamine, benatidine, mebevirine, dicyclovirine, citropium bromide, acetaminolol, clonidine, nifedipine, verapamil, loperamide, glucose, acidophilus, lactic acid bacteria, polycoton, Christiecon, Clostridium caseinate, mia, oxygen, bacillus licheniformis, lactobacin, cisapride, mannitol, acetic acid, leuprolide, octreotide, growth inhibition Vegetarian, montmorillonite, oryzanol, calcium pantothenate, cinnarizine  相关检查  [小 播报c编辑](javascript:;)  Serotonin |  |
| --- | --- | --- |

| 岔 搜索发现  [哪里有钢格板厂家](https://www.baidu.com/s?word=%E5%93%AA%E9%87%8C%E6%9C%89%E9%92%A2%E6%A0%BC%E6%9D%BF%E5%8E%82%E5%AE%B6&tn=SE_baikepcxf02_fcetbk02&pos=baike_pc_turbo_1767&ori_sid=00bb350b8f4234b8)  [机器人教育加盟哪家好](https://www.baidu.com/s?word=%E6%9C%BA%E5%99%A8%E4%BA%BA%E6%95%99%E8%82%B2%E5%8A%A0%E7%9B%9F%E5%93%AA%E5%AE%B6%E5%A5%BD&tn=SE_baikepcxf02_fcetbk02&pos=baike_pc_turbo_1767&ori_sid=00bb350b8f4234b8) [跑车租车](https://www.baidu.com/s?word=%E8%B7%91%E8%BD%A6%E7%A7%9F%E8%BD%A6&tn=SE_baikepcxf02_fcetbk02&pos=baike_pc_turbo_1767&ori_sid=00bb350b8f4234b8)  [孕妇护肤品哪种好](https://www.baidu.com/s?word=%E5%AD%95%E5%A6%87%E6%8A%A4%E8%82%A4%E5%93%81%E5%93%AA%E7%A7%8D%E5%A5%BD&tn=SE_baikepcxf02_fcetbk02&pos=baike_pc_turbo_1767&ori_sid=00bb350b8f4234b8)  [礼堂椅生产批发厂家](https://www.baidu.com/s?word=%E7%A4%BC%E5%A0%82%E6%A4%85%E7%94%9F%E4%BA%A7%E6%89%B9%E5%8F%91%E5%8E%82%E5%AE%B6&tn=SE_baikepcxf02_fcetbk02&pos=baike_pc_turbo_1767&ori_sid=00bb350b8f4234b8)  [机房用空调](https://www.baidu.com/s?word=%E6%9C%BA%E6%88%BF%E7%94%A8%E7%A9%BA%E8%B0%83&tn=SE_baikepcxf02_fcetbk02&pos=baike_pc_turbo_1767&ori_sid=00bb350b8f4234b8)  [折叠自行车](https://www.baidu.com/s?word=%E6%8A%98%E5%8F%A0%E8%87%AA%E8%A1%8C%E8%BD%A6&tn=SE_baikepcxf02_fcetbk02&pos=baike_pc_turbo_1767&ori_sid=00bb350b8f4234b8)  [女](javascript:void(0);)  [口](javascript:void(0);) |
| --- |

投诉建议

Q

我有疑问

新手上路 [成长任务](https://baike.baidu.com/usercenter/tasks#guide)

[编辑规则](https://baike.baidu.com/help#main06)

[在线客服](http://zhiqiu.baidu.com/baike/passport/html/baikechat.html)

[编辑入门](https://baike.baidu.com/help#main01) [内容质疑](javascript:void(0);)

[本人编辑](https://baike.baidu.com/item/%E7%99%BE%E5%BA%A6%E7%99%BE%E7%A7%91%EF%BC%9A%E6%9C%AC%E4%BA%BA%E8%AF%8D%E6%9D%A1%E7%BC%96%E8%BE%91%E6%9C%8D%E5%8A%A1/22442459?bk_fr=pcFooter) [官方贴吧](http://tieba.baidu.com/f?ie=utf-8&fr=bks0000&kw=%E7%99%BE%E5%BA%A6%E7%99%BE%E7%A7%91)

[举报不良信息](http://help.baidu.com/newadd?word=%E6%98%93%E6%BF%80%E7%BB%93%E8%82%A0&&submit_link=https%3A%2F%2Fbaike.baidu.com%2Fitem%2F%25E6%2598%2593%25E6%25BF%2580%25E7%25BB%2593%25E8%2582%25A0%3FfromModule%3Dsearch-result_lemma&prod_id=10&category=1) [投诉侵权信息](http://help.baidu.com/newadd?word=%E6%98%93%E6%BF%80%E7%BB%93%E8%82%A0&&submit_link=https%3A%2F%2Fbaike.baidu.com%2Fitem%2F%25E6%2598%2593%25E6%25BF%2580%25E7%25BB%2593%25E8%2582%25A0%3FfromModule%3Dsearch-result_lemma&prod_id=10&category=6)

[未通过词条申诉](http://help.baidu.com/newadd?word=%E6%98%93%E6%BF%80%E7%BB%93%E8%82%A0&&submit_link=https%3A%2F%2Fbaike.baidu.com%2Fitem%2F%25E6%2598%2593%25E6%25BF%2580%25E7%25BB%2593%25E8%2582%25A0%3FfromModule%3Dsearch-result_lemma&prod_id=10&category=2) [封禁查询与解封](http://help.baidu.com/newadd?word=%E6%98%93%E6%BF%80%E7%BB%93%E8%82%A0&&submit_link=https%3A%2F%2Fbaike.baidu.com%2Fitem%2F%25E6%2598%2593%25E6%25BF%2580%25E7%25BB%2593%25E8%2582%25A0%3FfromModule%3Dsearch-result_lemma&prod_id=10&category=5)

[意见反馈](javascript:void(0);)

©2022 Baidu [使用百度前必读](http://www.baidu.com/duty/) | [百科协议](http://help.baidu.com/question?prod_en=baike&class=89&id=1637) | [隐私政策](http://help.baidu.com/question?prod_id=10&class=690&id=1001779) | [百度百科合作平台](https://baike.baidu.com/operation/cooperation) | 京ICP证030173号

[京公网安备11000002000001号](http://www.beian.gov.cn/portal/registerSystemInfo?recordcode=11000002000001)

<https://baike.baidu.com/item/>易激结肠?fromModule=search-result_lemma

6/6
