# Supplementary material for: Assessment of medical information on irritable bowel syndrome information in Wikipedia and Baidu Encyclopedia: comparative study
Source: PeerJ. 2024 May 24;12:e17264. doi: 10.7717/peerj.17264 (PMC11129691; doi:10.7717/peerj.17264)
Supplement: Data S1 [file peerj-12-17264-s001.zip › σÄƒσoïμò░μì«/Baidu/Baidu-Chinese/7-ΦéáΦ║üτùç_τÖ╛σ║aτÖ╛τoæ.pdf]

网页 新闻 贴吧 知道 网盘 图片 视频 地图 文库 百科 | 百度首页 登录

Baidu百科 肠躁症 进入词条 全站搜索 帮助

近期有不法分子冒充百度百科官方人员，以删除词条为由威胁并敲诈相关企业。在此严正声明：百度百科是免费编辑平台，绝不存在收费代编服务，请勿上当受骗！ 详情>>

首页 秒懂百科 特色百科 用户 知识专题 权威合作 下载百科APP | 个

收藏 | 1627 | 1093

肠躁症

播报 编辑 上传视频

肠躁症是最常见的消化系统失调，至少百分之10~15%的成人发生，大多数病例都是因为压力过大而造成，受情绪影响较大。

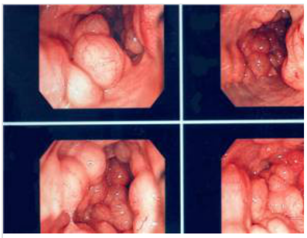
肠躁症的概述图（1张）

别　名	肠躁症	中医病名	结肠痉挛
		外文名	Irritable Bowel Syndrome

英文名称：Irritable Bowel Syndrome

中文名称：肠躁症（躁性大肠症候群）

简称缩写：IBS

名词解释：又称为结肠痉挛，是最常见的消化系统失调，至少百分之10~15%的成人发生，约90%的病例与心理压力有关。主要症状为腹痛，肠胃不适，便秘，或下痢。近12个月内，连续或间歇性，有以上症状达12周以上。

词条统计

浏览次数：90686次

编辑次数：8次历史版本

最近更新：sunnyahuman （2016-01-25

突出贡献榜

tjh19931014

词条图册

更多图册 >

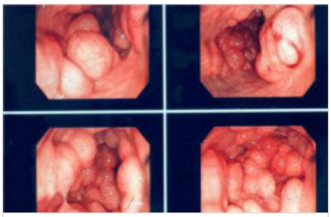
概述图册 (1)

1 出版社自费出
2 游戏盒子
3 网络安全培训
4 国际期货
5 电商平台怎么
6 战队logo设计
7 无人机反制
8 csgo电脑配置
9 哈佛大学申请
10 图书批发网
11 网络工程师
12 自己创建个[illegible]
13 虚拟货币平[illegible]
14 vr[illegible]
15 亚[illegible]
16 购[illegible]
17 价[illegible]
18 37[illegible]
19 sci[illegible]
20 csg[illegible]
21 怎[illegible]
22 供应[illegible]

学术论文

内容来自 Baidu学术

陶诗秀. 肠躁症的饮食治疗. 《vip》，2016

陶诗秀. 肠躁症的饮食治疗. 《现代养生》，2016

欣欣. 肠躁症又是什么?. 《健康之家》，2016

偏头痛与肠躁症有关. 《vip》，2017

查看全部 >

搜索发现

胶囊胃镜 胃肠胀气 肠道功能紊乱的症状 肠炎的治法 肚子胀痛快速解决办法
肠躁症 肠燥症症状 肠燥症怎么办 教师资格证考试时间 中专学校

新手上路：成长任务 编辑入门 编辑规则 本人编辑 NEW
我有疑问：内容质疑 在线客服 官方贴吧 意见反馈
投诉建议：举报不良信息 未通过词条申诉 投诉侵权信息 封禁查询与解封

秒懂百科 金灿荣 详解2022年俄乌冲突

©2022 Baidu 使用百度前必读 | 百科协议 | 隐私政策 | 百度百科合作平台 | 京ICP证030173号
京公网安备11000002000001号
