# Supplementary material for: Assessment of medical information on irritable bowel syndrome information in Wikipedia and Baidu Encyclopedia: comparative study
Source: PeerJ. 2024 May 24;12:e17264. doi: 10.7717/peerj.17264 (PMC11129691; doi:10.7717/peerj.17264)

Baidu百科 IBS 进入词条 播报 编辑 讨论 收藏 赞

IBS 进入词条 全站搜索 帮助

近期有不法分子冒充百度百科官方人员，以删除词条为由威胁并敲诈相关企业。在此严正声明：百度百科是免费编辑平台，绝不存在收费代编服务，请勿上当受骗！ 详情>>

首页 秒懂百科 特色百科 用户 知识专题 权威合作 下载百科APP 个

IBS是一个多义词，请在下列义项上选择浏览（共6个义项） 展开 添加义项 +

IBS

播报 编辑 讨论 上传视频

肠易激综合征

什么是肠易激综合征? 02:22

秒懂 一看就懂的视频

收藏 178 108

IBS即肠易激综合征(Irritable Bowel Syndrome，IBS)是一种常见的功能性肠病，以腹痛或腹部不适为主要症状，排便后可改善，常伴有排便习惯改变，缺乏解释症状的形态学和生化学异常。

世界各地流行病学研究报道显示IBS是一种世界范围内的多发病。西方国家人群患病率达10%—20%，我国的一项按罗马Ⅱ标准的流行病学调查显示社区人群IBS患病率为5.7%，其中22%曾因IBS症状而就诊。

西医学名	肠易激综合症	常见症状	腹痛，腹胀，腹泻，便秘
外文名	irritable bowel syndrome,IBS	英文简称	IBS

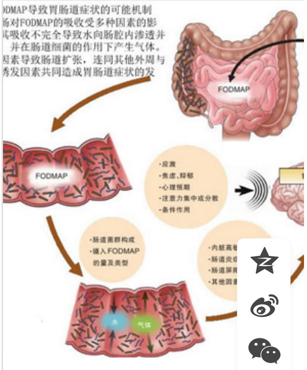

IBS的概述图（2张

词条统计

浏览次数：871420次

编辑次数：51次历史版本

最近更新：w_ou （2021-01-26）

突出贡献榜

shmily32123

1 aci营养师 12 自己创建个
2 sci文献 13 手机空号
3 营养师报考资 14 大专生怎么
4 二级心理咨询 15 意大利留学
5 电商怎么做 16 征文投稿
6 快速学日语 17 如何治脸瘫
7 战队logo设计 18 9733游戏平
8 哈佛大学申请 19 芝加哥留学
9 哈佛大学入学 20 怎么成为插
10 价格便宜的香 21 华师在职研
11 千锋教育 22 哈佛大学宿

秒懂百科 金灿荣 详解2022年俄乌冲突

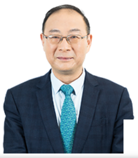

目录

1 病因
2 诊断
3 肠易激综合征
4 鉴别诊断
5 治疗

病因

播报 编辑

一般的病因是机体应激反应与心理因素相互作用的结果，不同的个体都可能涉及遗传、环境、心理、社会和胃肠感染等因素，导致胃肠动力改变、脑—肠轴相互作用的紊乱、自主神经和激素的变化等，伴有精神障碍（如恐慌、焦虑、创伤后应激紊乱等）、睡眠障碍和心理应对障碍的患者，应激性生活事件常可导致症状的加重，但对心理因素与IBS之间的确切联系还不十分清楚。研究发现有1/3的IBS患者有胃肠道感染史，国内外都强调精神心理因素对IBS发病的影响，更加重视神经肽类和相关受体功能在IBS发病机制中的作用；近些年人们加强了IBS与炎症性肠病（IBD）之间的联系研究，少数学者甚至认为IBS为IBD的前期表现。

诊断

播报 编辑

依旧以症状学作为临床诊断和研究。

肠易激综合征

播报 编辑

1）IBS的一般标准 在最近的3个月内，每个月至少有3天出现反复发作的腹痛或不适症状，并具有下列中的2项或2项以上：

①排便后症状改善。

②伴随排便频率的改变。

③伴随粪便性状的改变。

2）IBS的一般下列症状可支持诊断：

①异常的排便频率：a.每周≤3次排便或b.每天>3次排便。

②异常的粪便性状：c.块状便/硬便或d.松散便/稀水便。

③排便费力。

④排便急迫感或排便不尽感。

⑤排出黏液。

⑥腹胀。

3）IBS亚型 依据粪便的性状分为以下亚型：

①IBS便秘型（IBS-C）：硬便或块状便排便比例≥25%，稀便（糊状便）或水样便排便比例<25%。

②IBS腹泻型（IBS - D）：稀便（糊状便）或水样便排便比例≥25%，硬便或块状便排便比例<25%。

③混合型IBS(IBS-M)：硬便或块状便排便比例>25%，稀便（糊状便）或水样便排便比例≥25%。

④不确定型IBS（IBS-U）：粪便的性状不符合上述IBS-C，D，M之中的任一标准。

在IBS分型时除需注重粪便性状外，还应注意到患者的排便费力、急迫感和排便不尽感等症状，在多数情况下粪便性状（从稀水样泻到硬结便）能够反映肠管的转运时间。

既往强调诊断的要点是：排便后腹痛减轻，腹痛时伴大便次数增多；腹痛发作时大便变稀，明显腹胀。现依旧在使用，但在鉴别器质性肠病上价值有限，需要适时作恰当的检查。确定IBS的诊断通常需要进行详细的采集病史和体格检查，根据不同患者的需求，有针对性地进行实验室和辅助检查。以便尽可能以器质性疾病本身解释所有的症状，轻的器质病也可以与IBS同在。

鉴别诊断

播报 编辑

需要与IBS鉴别诊断的疾病主要有炎症性肠病、结直肠肿瘤，还要注意IBS·D与乳糖不耐受、小肠细菌过度生长、寄生虫感染等鉴别。

治疗

播报 编辑

在此强调个体化的综合治疗，即应包括精神心理行为干预治疗、饮食调整和药物治疗，患者的治疗方法和对药物的选择应因人而异对症处理。制定罗马Ⅲ中IBS专家组对治疗的推荐意见有三类：

1、**心理治疗**

症状严重而顽固，经一般治疗和药物治疗无效者应考虑予心理行为治疗。包括心理治疗、认知治疗、催眠疗法、生物反馈等。

2、**饮食调整**

不良的饮食习惯和膳食结构可以加剧IBS的症状。因此，健康、平衡的饮食可有助于减轻患者的胃肠功能紊乱症状。IBS患者宜避免：

①过度饮食；

②大量饮酒；

③咖啡因；

④高脂饮食；

⑤某些具有“产气”作用的蔬菜类等；

⑥精加工食粮和人工食品(便秘者)，山梨醇及果糖(腹泻者)；

⑦不耐受的食物(因不同个体而异)。

增加膳食纤维主要用于便秘为主的IBS患者，增加纤维摄入量的方法应个体化。

3、**药物治疗**

主要针对症状显著的患者，由于IBS患者症状复杂多变且与中枢和肠神经系统间复杂的关系，药物都存在有效性和安全性的限制，只能一定程度起作用。得舒特为代表胃肠选择性钙离子拮抗剂在各类平滑肌解痉剂中最欢迎，在数十个国家使用；替加色罗（随罗马Ⅲ标准一同推出）曾风光无限，其有效性和安全性的限制了它的使用；近几年对益生菌治疗很重视，具有调节内脏敏

秒懂百科
金灿荣
详解2022年俄乌冲突

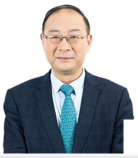

Bai du 百科 播报 编辑 讨论 收藏 赞

其它非药物治疗有心理专科治疗、催眠治疗、生物反馈治疗、行为治疗等，这些现虽未被罗马Ⅲ中IBS专家组对治疗的推荐，但与上述有交叉，也显出一定的疗效，需要进一步研究。

肠易激综合征（IBS）是一种常见的功能性胃肠病，以腹痛或腹部不适为主要症状，排便后可改善，常伴排便习惯及大便性状改变，便秘和腹泻可交替出现，但缺乏可解释症状的形态学和生化异常。过去称为“痉挛性结肠炎”“黏液性结肠炎”及“不稳定性结肠炎”等。该病患者以20-40岁中青年居多，女性多见；具有反复发作的倾向，常迁延难愈。在药物治疗方面，西医暂无理想的药物。

肠易激综合征在中国的发病率在10%-20%左右，患者以中青年为主。确切的致病机制仍不明确，但精神心理因素是导致发病的重要因素，严重影响患者生活质量。患者应及时就医，可在医生指导下选用解痉剂缓解相应症状；以及止泻剂/导泻剂对应改善腹泻/便秘的症状；或通过益生菌调整肠道有益菌群，减轻腹部不适症状。调整饮食结构，改善情绪状态，才能实现逐渐康复的目的。 [1]

词条图册

更多图册 >

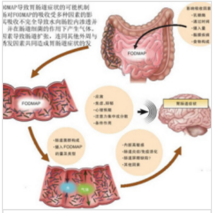

概述图册 (2)

NRN

加盟电台	STV	HBC	RAB	IBC	TBC	ABS
	YBC	RFC	QR	LF	IBS	CRT
	YBS	SBC	BSN	SBS	SF	KNB
	MRO	FBC	MBS	ABC	OBC	KBS
	BSS	RSK	RCC	KRY	JRT	RNC
	RNB	RKC	KBC	NBC	RKK	MRT
	MBC	ROK				
原加盟电台	CRK					

参考资料

1 加强大众科学认知肠易激综合征 . 新华网[引用日期2015-04-22]

学术论文

内容来自 Bai du 学术

李红缨，高丽，李宁秀. IBS-QOL专用量表在肠易激综合征患者中的运用. 《中国循证医学杂志》，2004

王世勇，陈曦，高权国. 艾灸治疗肠道易激综合征(IBS)30例临床观察. 《CNKI;WanFang》，2003

樊冬梅，刘凤斌，杨晓军等. 便秘型肠易激综合征(IBS)从脾论治的病机探微. 《吉林中医药》，2006

李敏雅. 陆维宏辨证治疗肠易激综合征(IBS)经验拾萃. 《浙江中医药大学学报》，2009

康宁宁，潘迪，谭悦，付钰. 益生菌联合情志疗法对腹泻型肠易激综合征(IBS-D)患者的疗效及生存... 《VIP》，2016

查看全部 >

猜你喜欢

vvs欧洲供应商凯信贸易
ibs德国直采，原厂报价单报价，折扣优惠，可提供报关单，欧洲工业品上游供应商，经营65家代理品牌，2486家真实成交20000...
www.kaixinbusiness.com

德国ibs的产品，原装进口，欢迎洽谈
优势供应ibs的产品，报价快，货期稳定[illegible]证，质优价[illegible]
提供报关单和原产地证明，期待您的来电
www.qiucheng-tech.com

金灿荣 谨解2022年俄乌冲突

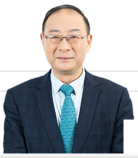

搜索发现

Baidu百科

播报 编辑 讨论 收藏 赞

新手上路
成长任务 编辑入门
编辑规则 本人编辑 NEW

我有疑问
内容质疑 在线客服
官方贴吧 意见反馈

投诉建议
举报不良信息 未通过词条申诉
投诉侵权信息 封禁查询与解封

©2022 Baidu 使用百度前必读 | 百科协议 | 隐私政策 | 百度百科合作平台 | 京ICP证030173号
京公网安备11000002000001号

秒懂百科
金灿荣
详解2022年俄乌冲突

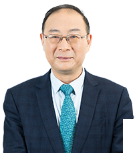

Supplement: Data S1 [file peerj-12-17264-s001.zip › σÄƒσoïμò░μì«/Baidu/Baidu-Chinese/1-IBS∩╝êΦéáμÿôμ┐Çτ╗╝σÉêσ╛ü∩╝ë_τÖ╛σ║aτÖ╛τoæ.pdf]
