# Supplementary material for: Assessment of medical information on irritable bowel syndrome information in Wikipedia and Baidu Encyclopedia: comparative study
Source: PeerJ. 2024 May 24;12:e17264. doi: 10.7717/peerj.17264 (PMC11129691; doi:10.7717/peerj.17264)

近期有不法分子冒充百度百科官方人员，以删除词条为由威胁并敲诈相关企业。在此严正声明：百度百科是免费编辑平台，绝不存在收费代编服务，请勿上当受骗！ 详情>>

激躁性大肠症候群

肠胃道疾病

激燥性大肠症(Irritable Bowel Syndrome) 是临床上最常见到的肠胃道疾病，虽然该病不是致命的疾病，但病人常有很大的痛苦，并且医师在治疗时有爱莫能助和灰心丧气的感觉。

激躁性大肠症常见于青年或中年人，女/男 比例1：2。主要表现是慢性便秘，间歇性腹泻或两者均有。

中文名	激躁性大肠候群	性　质	医学
外文名	Irritable Bowel Syndrome	类　别	疾病

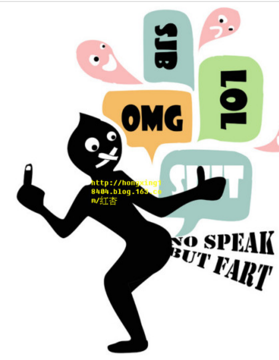

激躁性大肠症候群的概述图（张）

词条统计
浏览次数：247543次
编辑次数：17次历史版本
最近更新：w_ou （2021-01-26）
突出贡献榜
qqq582311436

目录

1 疾病类型
2 临床表现
3 病理生理
4 诊断要点
5 治疗方式
6 预防保养

疾病类型

激燥性大肠症有三种：

(一) 慢性无痛间歇性腹泻： 很多人都有胃肠毛病，如腹泻、便秘、胀气等却又查不出原因所在。

(二) 慢性间歇性腹痛与便秘：痉挛性结肠炎的病人主诉有慢性腹痛和便秘。

(三) 腹泻与便秘交替出现： 还有一些病人可出现上述两种表现，便秘与腹泻交替出现。

临床表现

典型病人可数年或数月内间断出现水样腹泻，常在清晨或早餐后腹泻加重，在排泄3-4次带大量黏液的稀便后，病人其余时间均感觉正常。整天腹泻，或总是夜间腹泻者很少见。腹泻可持续几周或数月，随后自动消失一段时间。有些病人无腹泻而排泄"铅笔样"糊状大便。 病人另一种典型的表现是慢性腹痛，伴便秘，或者便秘与腹泻交替出现。这些病人主诉间断性下腹部绞痛，排气或排便后可缓解。激躁性大肠症还常有胃灼热、明显腹胀、背痛、软弱、疲乏、心悸等。

病理生理

激躁性大肠症的基本病理生理异常是肠运动改变。结肠痉挛的病人（腹痛和便秘）静止时结肠动力增加。

与此相反，主要表现为腹泻的病人静止时结肠动力减弱。某些激燥性大肠症病人可有显著精神异常的证据。常见的有忧郁、歇斯底里、强迫症。

精神刺激也可使症状加重。然而应该注意是正常人在急性压力反应时，结肠内压力可升高。

这提示精神刺激可能是激躁性大肠症的非特异性诱发因素，同在其他疾病中一样，和许多病因一起致病。

诊断要点

1.有上述胃肠功能失常的症状及伴有全身性官能症状。 2.患者的症状常随情绪而变化。 3.全消化道摄影检查或胃镜、直肠镜检查正常，排除器质性病变。 三、病因病机： 1.感受外邪：六淫之中以寒、湿、暑、热为常见，又以感受湿邪致泻者尤多。

2.饮食所伤：多为饮食过量，宿食内停；或不节肥甘，呆胃滞脾；或生冷不洁，有伤脾胃。

3.情志失调：肝郁乘脾 4.体虚久病：脾虚及肾

治疗方式

1 购买域名
2 37游戏平台
3 国际期货
4 csgo电脑配置
5 战队logo设计
6 自己创建个网
7 游戏盒子
8 哈佛大学申请
9 无人机反制
10 网络安全培训
11 亚马逊图书
12 电[illegible]
13 sci[illegible]
14 虚[illegible]
15 图[illegible]
16 网[illegible]
17 出版社自费
18 vr消防演练
19 价格便宜的[illegible]
20 csgo网站开[illegible]
21 怎么创建小[illegible]
22 供应链管理[illegible]

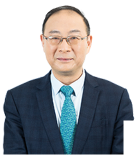

寒湿为泄泻的主要病理因素，脾虚湿盛是其发病关键。固治疗当以运脾祛湿为原则。辩证施治

1.寒湿困脾：散寒化湿，健脾止泻

2.湿热下注：清热利湿，厚肠止泻

3.食滞肠胃：消食导滞、调和脾胃

4.肝气乘脾：抑肝扶脾、调和脾胃

5.脾虚湿盛：健脾益气、运中止泻

6.脾肾阳虚：温补脾肾、固肠止泻

预防保养

播报 编辑

1.注意饮食卫生，起居有常

2.勿过食生冷、或肥甘厚腻、或饮酒无度

3.注意情志因素

4.注意保暖，切勿受湿受凉

词条图册

更多图册 >

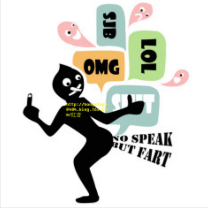

概述图册 (1)

猜你喜欢

躁郁自测--抑郁自评量表SDS，标准版

躁郁自测，抑郁测量表，网上的测评试题 评分标准，综合心理，健康测试，心理测试，心理抑郁，全方位解读，图表分析，针对...

bdcs.lhwljy.top

搜索发现

激躁性大肠症候群 肠炎的症状 激躁性大肠要吃药吗 肠梗阻症状及治疗 运动治好了我的肠易激
大肠激躁症能喝酸奶吗 大肠激躁症可以自愈 狂躁症 暴躁症 狂躁症吃什么药

新手上路
成长任务 编辑入门
编辑规则 本人编辑 NEW

我有疑问
内容质疑 在线客服
官方贴吧 意见反馈

投诉建议
举报不良信息 未通过词条申诉
投诉侵权信息 封禁查询与解封

©2022 Baidu 使用百度前必读 | 百科协议 | 隐私政策 | 百度百科合作平台 | 京ICP证030173号
京公网安备11000002000001号

秒懂百科
金灿荣
详解2022年俄乌冲突

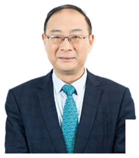

Supplement: Data S1 [file peerj-12-17264-s001.zip › σÄƒσoïμò░μì«/Baidu/Baidu-Chinese/5-μ┐ÇΦ║üμÇoσñoΦéáτùçσÇÖτ╛ñ_τÖ╛σ║aτÖ╛τoæ.pdf]
